# Molecular crowding effects on protein stability in a bacterial proteome

**DOI:** 10.1038/s41598-026-35990-9

**Published:** 2026-01-21

**Authors:** Kate McKeever, Eugene T. Dillon, Kieran Wynne, Gerard Cagney

**Affiliations:** 1 BiOrbic - Bioeconomy Research Centre, Ireland, University College Dublin, Belfield, Dublin 4, Ireland; 2 UCD Conway Institute, University College Dublin, Belfield, Dublin 4, Ireland; 3 School of Biomolecular and Biomedical Science, University College Dublin, Belfield, Dublin 4, Ireland; 4 Systems Biology Ireland, University College Dublin, Belfield, Dublin 4, Ireland

**Keywords:** Biochemistry, Biological techniques, Biophysics, Computational biology and bioinformatics

## Abstract

Molecular crowding refers to the restriction of space available for molecular mobility in solution due to the presence of other macromolecules. Crowding can influence biological phenomena such as protein stability and folding, diffusion, enzyme kinetics, molecular interactions, and phase separation. However, the relative contributions of entropic (excluded volume) and enthalpic (direct chemical interactions) is still an open question. In contrast to biochemical studies that employ dilute solutions containing a small number of reactants, the cellular environment is considered to be highly crowded, with up to 40% of cell volume occupied by many thousands of proteins and other large biomolecules. We examined the protein stability effects of widely used molecular crowders in a bacterial proteome (*Cupriavidus necator*) using the Thermal Proteome Profiling (TPP) method, whereby melting temperature is estimated based on protein denaturation and aggregation over a heat gradient. While all six tested compounds (Ficoll 70, Ficoll 400, dextran 40, dextran 86, PEG 1, PEG 8) reduced the global mean melting temperature, significantly increased or decreased stability was observed among distinct subsets of the proteome, with some individual proteins showing sensitivity to multiple crowding agents. Proteins displaying enhanced stability in the presence of crowding agents were more likely to be annotated as hydrophobic, to show classic enzyme-like properties, or propensity for protein interactions. Our data favour a direct binding/preferential exclusion model of enhanced stability, rather than alternative models based on viscosity or crowding.

**Supplementary Information:**

The online version contains supplementary material available at 10.1038/s41598-026-35990-9.

## Introduction

The structure and function of protein molecules are intrinsically linked. While they can potentially fold into any of a vast number of three-dimensional conformations, proteins in their biologically active states generally assemble into defined or ‘native’ configurations. The stability of native proteins is influenced by many biophysical factors that affect the thermodynamic state including temperature, pH, protein interactions (oligomerization or ligand binding), or the presence of cosolvents^1^. Proteins in solution exist in an equilibrium between folded and unfolded (or denatured) states, and the transition between states can be described by a sigmoid function. For example, proteins typically show a sigmoid response when stepwise increases in temperature lead to aggregation, with the midpoint of the S-curve (the temperature where 50% of the protein population has precipitated) termed the melting temperature, or T_m_. The recently described Thermal Proteome Profiling, or TPP, method (an extension of the CETSA or Cellular Thermal Shift Assay that analyzes single proteins) uses isobaric mass tagging combined with high resolution high mass accuracy LCMS to capture irreversible denaturation/aggregation behaviour (as opposed to reversible unfolding) on a proteome-wide scale^2^. Using this method, we determined the relative protein stability—estimated by calculating T_m_ from a progressive heat gradient—for hundreds of proteins in the presence or absence of macromolecular crowding agents.

Several classes of molecule are known to influence protein conformation, either as promoters of stability (termed osmolytes if they are of low molecular mass) or of instability (known as denaturing agents). Stabilizing osmolyte cosolvents include many naturally-occurring small, neutral molecules, for example trimethylamine *N*-oxide (TMAO), betaine, or glycine. Conversely, urea or guanidinium chloride promote denaturation. The precise mechanisms by which these compounds exert their effects are still under active study^3^. Some models of protein conformation stability propose that protein stabilizers such as TMAO act indirectly by influencing water structure via hydrogen bonding^4^. In contrast, denaturants are proposed to interact favourably with the polypeptide backbone and amino acid sidechains, thereby disrupting the native protein conformation^5^.

In addition to small molecule cosolvents, cells contain large quantities of macromolecules that are proposed to impact both protein folding equilibria and interactions with other molecules^6^. The concept of molecular crowding was introduced by Alexander Ogston, Allen Minton and others to describe how the very high concentrations of macromolecules encountered under in vivo conditions, e.g. > 150 mg/ml. This can result in effects that are not evident from in vitro studies employing dilute solutions, notably volume exclusion, i.e. the occupation of significant proportions of cell volume by ‘crowders’ restricts the space available to proteins and other solute molecules and raises their effective concentration. Various models that attempt to explain how osmolytes and macromolecular crowders influence the biochemistry of living cells have been presented. These incorporate both entropic and enthalpic thermodynamic components^7^. The former refer to purely physical effects arising from spatial restrictions, diffusion limits etc., while the latter incorporate specific interactions between the crowding agents and proteins. Another set of models place less emphasis on physical crowding effects, but rather propose a mechanism based on preferential exclusion from protein surfaces, termed the ‘osmophobic effect’^8^.

Here we investigated the effect of three structurally distinct classes of molecular crowding agent (three polymer types, each represented by two molecular mass variants) on protein conformation at proteome scale using the TPP approach. We examined the proteome of *Cupriavidus necator* (previously known as *Ralstonia eutropa*). This organism is of interest as an agent of the green economy due to its potential for carbon sequestration, hydrogen oxidation, and bioplastic production^9^. Overall, we found that while the global mean T_m_ is reduced in the presence of molecular crowding agents, subsets of proteins show alternatively stabilized or destabilized effects, suggesting that the stability of proteins within cells is highly complex and protein-specific. We also found that proteins stabilized by molecular crowding agents were enriched for annotated biophysical and biochemical features including increased hydrophobicity, a tendency to have enzyme-like properties, or to engage in protein interactions. Finally, we found moderate evidence that these reagents stabilize proteins by favouring a preferential exclusion mechanism rather than by physical crowding.

## Experimental procedures

### Growth of bacterial cells

*Cupriavidus necator* H16 (DSM 428; ATCC 17699), kindly provided by Kevin O’Connor (BiOrbic Institute, University College Dublin), was described by Pohlmann and coworkers^10^. *C. necator* cells were grown overnight at 30 °C on Miller’s Lysogeny Broth (LB) with shaking at 200 rpm. Overnight cultures were used to inoculate 50 mL of J-minimal medium (JMM) supplemented with 1 mM sodium gluconate (Sigma-Aldrich, S2054) as the carbon source to an initial OD_600_ of 0.05. Cultures were incubated at 30 °C, 200 rpm for 48 h and harvested by centrifugation at 10,000 × g for 10 min at 4 °C. Pelleted cells were washed twice with PBS and stored at −20 °C until further analysis.

Bacterial pellets were resuspended in 2 mL lysis buffer (50 µg/mL lysozyme (Thermo Fisher, 90,082), 0.8% IGEPAL CA-630 (Sigma-Aldrich, I8896), 250U/mL benzonase (Millipore, E1014), 1 mM MgCl2 (Sigma-Aldrich, 20303 M), 1 complete, EDTA-free protease inhibitor cocktail tablet (Thermo Fisher, 11,836,170,001) per 10 mL PBS (pH 7.4)). Suspensions were incubated at room temperature with shaking at 1000 rpm for 20 min, followed by three freeze–thaw cycles (−80 °C for 20 min, 25 °C for 5 min). Lysates were centrifuged at 20,000 × g, 4 °C for 20 min. Protein concentration was estimated using a Bradford assay (Pierce 23,200).

### Treatment with crowding agents

Stock solutions of Ficoll 70 (Sigma-Aldrich, F2878), Ficoll 400 (Sigma-Aldrich, F4375), dextran 40 (Sigma-Aldrich, 31,389), dextran 86 (Sigma-Aldrich, 31,390), PEG 1 (Sigma-Aldrich, 1,546,489), PEG 8 (Sigma-Aldrich, 89,510) were prepared at 300 mg/mL in PBS. Protein lysates were diluted to 1 µg/µL with PBS. For each treatment, 50 µL protein lysate and 50 µL of crowder stock was aliquoted into ten 0.2 mL PCR tubes, creating ten 100 µL subsamples per condition. This yielded final concentrations of 50 µg of protein and 150 mg/mL crowder per 100µL subsample. Protein/crowder mixtures were allowed to incubate for 20 min at room temperature. A no-crowder control was prepared by substituting 50 µL PBS for the crowder stock.

### Thermal proteome profiling

A heat gradient (30–70 °C) was applied using a LongGene A200 gradient thermal cycler for 3 min, and filtered through a MilliporeSigma MultiScreenHTS Durapore 96-well filter plate (Milllipore 10,448,023). The specific temperatures reached for each fraction were: 30, 34, 38, 43, 47, 52, 56, 60, 66, and 70 °C. 20 µL of PBS was used to wash through any remaining sample. Protein clean-up and digestion were carried out using Sera-Mag Speed Beads, according to the method of Hughes and coworkers^11^. Briefly, a mixture of hydrophilic and hydrophobic Sera-Mag Speed Beads (ThermoFischer Scientific 4515-2105-050,250, 6515-2105-050,250) were mixed (1:1) and 5µL of bead stock (100 µg/µL) added to each protein sample. The samples were shaken at 1000 rpm on a 1.5 mL Eppendorf Thermomixer 5350 for 15 min, washed 3 times on a magnetic rack with 80% ethanol before adding 50µL of digestion buffer (5 mM choloacetamide, Sigma C0267; 1.25 mM Tris(2-carboxyethyl) phosphine hydrochloride (TCEP), Biotium 91,049; 1 µg Trypsin/LysC, Pierce A41007; in 100 mM HEPES pH 8, Sigma H4034). Samples were digested overnight at 37 °C at 1000 rpm on the same Thermomixer. The peptide-containing supernatant was recovered by placing samples on the magnetic rack for 2 min to allow beads to attach to the magnet. The supernatant was retrieved, and beads were washed with 20 µL HPLC-grade water, vortexed, recovered, and pooled with the recovered supernatant. Samples were dried by SpeedVac and stored at -20 oC until further processing. Peptides were resuspended in HPLC-grade water. Each subsample, corresponding to a temperature point, was isobarically labelled using TMT-10plex (Thermo Fisher Scientific, 90,110) reagent at a molar ratio of 1:1 for 1 h at room temperature with shaking (1000 rpm). Reactions were quenched with 8 µL of 5% hydroxylamine (Sigma-Aldrich, 5470). TMT-labelled subsamples were combined into a single tube per condition, acidified, and desalted using StageTips. Eluted peptides were dried and stored at −80 °C until LC–MS analysis.

### Liquid chromatography-mass spectrometry

LC–MS was performed on a Thermo Scientific Q Exactive mass spectrometer connected to a Dionex Ultimate 3000 (RSLCnano) chromatography system. Tryptic peptides were resuspended in 0.1% formic acid. Each sample was loaded onto a fused silica emitter: 75 μm ID, pulled using a laser puller (Sutter Instruments P2000), packed with Reprocil Pur C18 reverse phase media (1.9 μm). LCMS Buffer A comprised 5% LCMS-grade acetonitrile in LCMS grade water with 0.1% formic acid, while Buffer B comprised 80% LCMS-grade acetonitrile in LCMS grade water with 0.1% formic acid. Samples were separated by an increasing acetonitrile gradient (0–40% Buffer B) over 300 min at a flow rate of 250 nl/min.

The mass spectrometer was operated in positive ion mode with a capillary temperature of 250 °C, and with a potential of 2300 V applied to the frit. All data was acquired with the mass spectrometer operating in automatic data dependent switching mode. MS1 parameters: resolution (70,000), scan range (350–1400 m/z), AGC target (3e6). MS2 parameters: resolution (35,000), fixed first mass 100 m/z, AGC target (2e5), Isolation window 1.2 m/z, maximum injection time 250 ms. The Q Exactive was set to select the 12 most intense ions prior to MS/MS analysis using HCD. The raw data was searched against the *Cupriavidus necator* (H16 strain) subset of the Uniprot Swissprot database (6614 entries; www.uniprot.org/proteomes/UP000008210) using the search engine Maxquant (release 2.0.3.0) using TMT 10plex workflow parameters^12,13^.

### Data analysis

For TPP analysis, melting curves derived from aggregated protein data were produced by normalizing mass spectral signal intensities (LFQ) to the lowest temperature (30 °C) and analyzing the transformed data with the TPP Bioconductor package using the highest stringency settings^14^. Protein annotations were obtained from the Uniprot database^15^. Other online resources or algorithms used for data analysis included the Multiple Protein Profiler^16^ and DAVID^17^.

### Experimental design and statistical rationale

Two technical replicates were used to assess the reproducibility of the LCMS platform; subsequently a single replicate was used for thermal proteomics (TPP) experiments, mainly dues to the cost of TMT reagent. Proteins whose melting temperature difference in the presence or absence of macromolecular crowding agent (ΔT_m_) elicited Z-scores of ≥ 1.96 or ≤ - −1.96 (α = 0.05) were considered significantly stabilized or destabilized, respectively.

## Results

### Thermal proteomics investigation of molecular crowding in a bacterial lysate

**Fig. 1:**
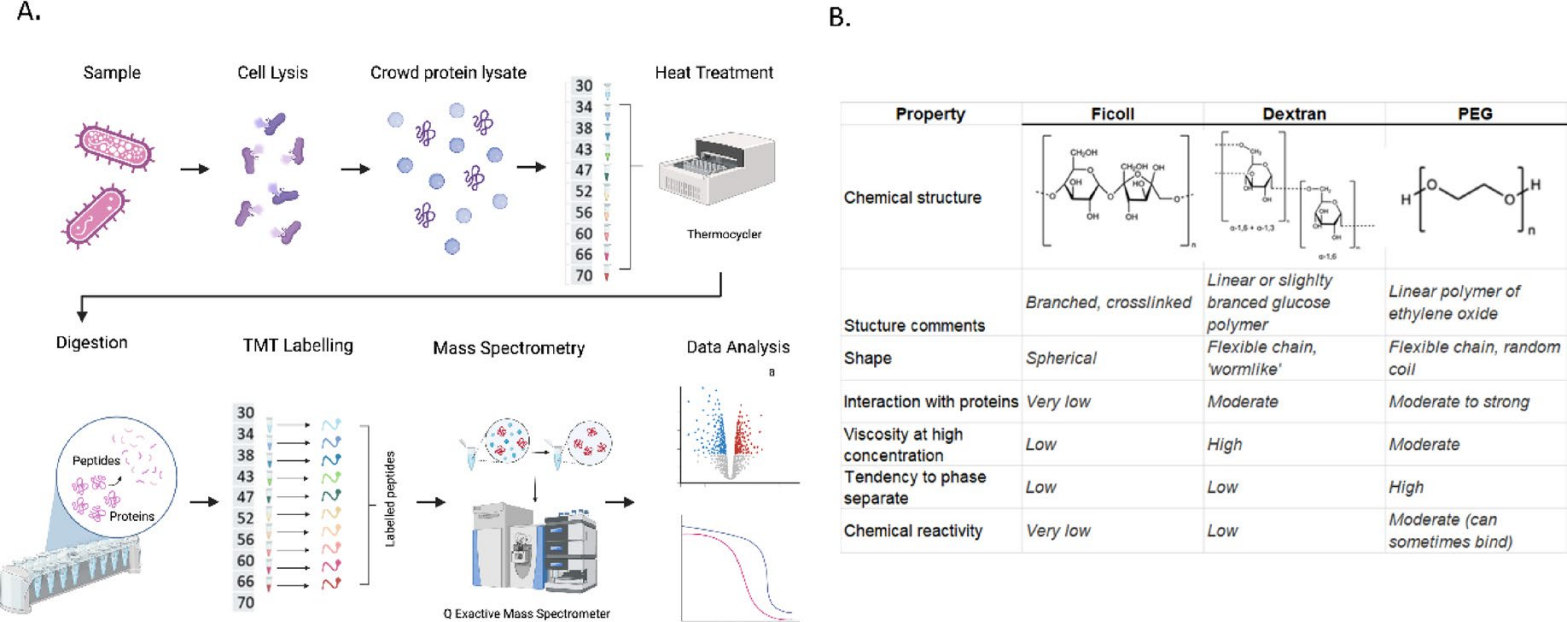
Thermal proteomics investigation of molecular crowding in a bacterial lysate. (**A**) Overview of the experimental approach. *C.necator* cells were grown in complete media, the cells lysed, and the resulting lysates individual treated with six compounds commonly used to experimentally induce molecular crowding effects. The effects on protein aggregation/denaturation were then monitored using a thermal gradient, with levels of denatured protein being determined using peptide TMT-labelling and LCMS (‘Thermal Proteome Profiling’). Melting temperature (T_m_) and other stability parameters were determined using the TPP R package. (**B**) Comparative properties of the six molecular crowding reagents used in the study.

The TPP approach combines a heat gradient step with a mass spectrometry readout to quantify the progressive aggregation of multiple proteins (i.e. a proteome sample) in a single analysis (Fig. 1A). Briefly, an overnight *C.necator* culture was lysed under mild conditions (0.8% IGEPAL, lysozyme, benzonase), and individual crowding agents were introduced at 150 mg/ml with the protein concentration of each sample adjusted to 0.5 µg/ml. This means that for the thermal gradient, protein was present at concentrations typical of a proteomics or biochemistry experiments, with the added crowding agents bringing the overall macromolecule concentration to levels typically observed in living bacterial cells^17–19^. The samples were divided into ten equal 100µL fractions, each heated to a single temperature point within the thermal gradient (30–70 °C). Increasing proportions of protein aggregate at higher temperatures, with the soluble fractions recovered, digested with trypsin, and the peptides labelled using isobaric TMT mass tagging. Following analysis by high resolution mass spectrometry, the proteins were identified and quantified, permitting thermal denaturation/aggregation curves to be constructed using the TMT mass spectral signals.

PEG, Ficoll, and dextran are frequently used in molecular crowding studies since at high concentrations they occupy large proportions of the available volume, thereby mimicking crowded cell environments. While they are considered to be chemically inert, no single reagent is likely to be completely unreactive. Additionally, each reagent has distinguishing properties such as monomer molecular mass, intrinsic viscosity, polymer topology (e.g. linear, branched), and chemical reactivity (Fig. 1B). We reasoned that by using all three reagents in TPP experiments investigating their effects on protein stability in a bacterial lysate, we could identify proteins that are globally sensitive to crowding, as well as proteins that respond specifically to individual reagents. We also used two MW variants within each crowding agent class: PEG 1 and PEG 8, Ficoll 40 and Ficoll 70, dextran 40 and dextran 86. These variant pairs should exhibit similar chemical reactivities, but maintain distinguishing properties in terms of excluded volume (higher MW is predicted to generate more crowded environments), and viscosity (higher MW is predicted to produce more viscous solutions at equal concentrations).

### Quality control of TPP experiments

**Fig. 2:**
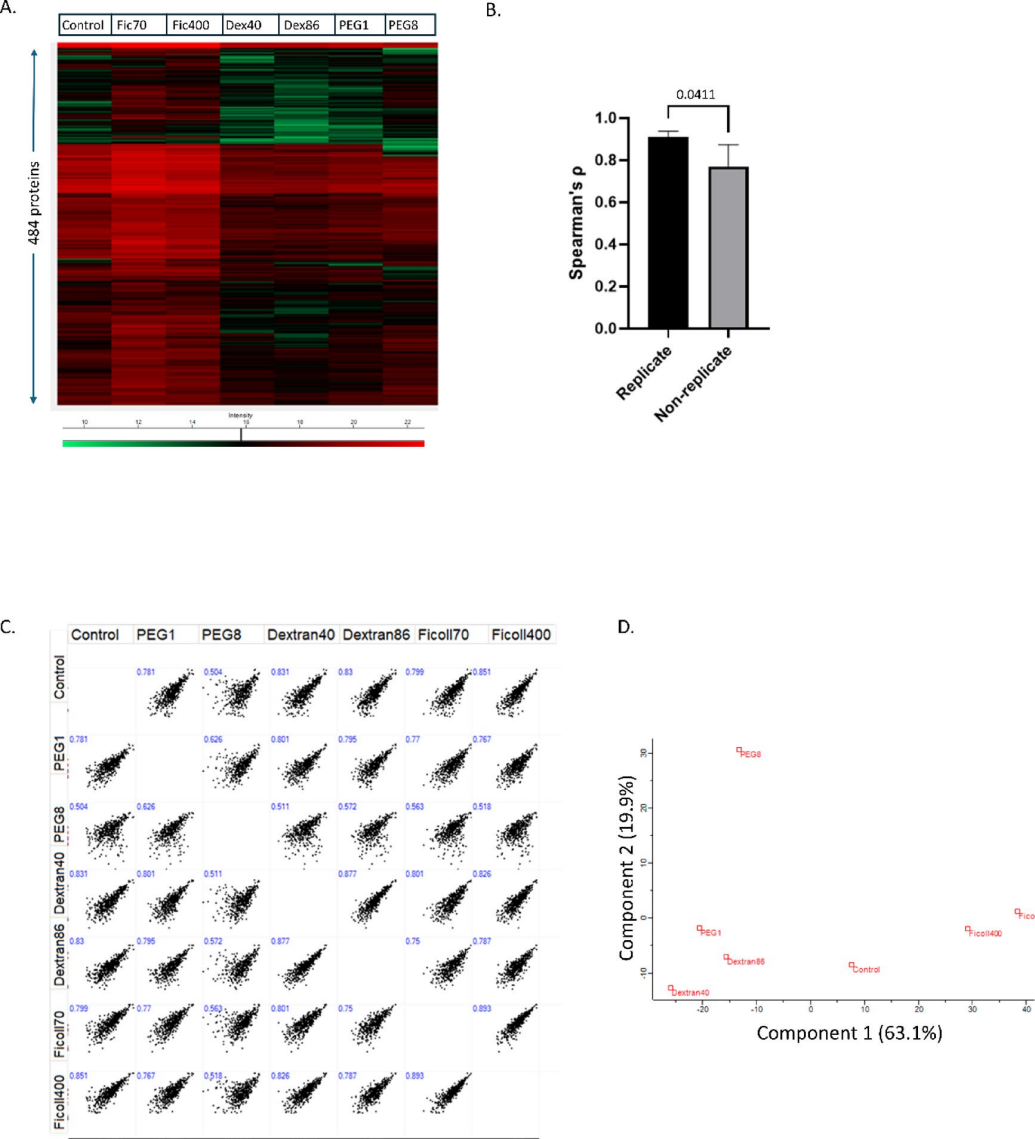
Quality control of the TPP experiments. (**A**) Protein expression levels (determined using the LFQ function of the MaxQuant program) for 484 proteins passing the quality control criteria of the TPP R package were analyzed using two-dimensional hierarchical clustering (Perseus software). ‘Intensity’ refers to the LFQ values from MaxQuant. (**B**) Spearman’s ρ was determined for all technical replicate and all non-replicate pairs, the former showing significantly higher correlation (Mann Whitney test). (**C**) Spearman’s ρ was also determined for all pairwise treatments with crowding reagents. (**D**) Principal Component Analysis of protein expression levels for all samples.

In total, 1746 proteins were identified across all samples using the MaxQuant program (excluding 19 reverse database matches, and 8 proteins classed as contaminants; Supplementary Table S1). Thermal proteome parameters were determined for 484 proteins using the most stringent settings of the TPP R package (Supplementary Table S2). This proportion of successfully analyzed proteins (28%) is similar to other published TPP studies but raises the question of whether the subset is biased in some way, for example in favour of more abundant or more stable proteins. While we could not rule out a bias in favour of stable proteins, we compared protein abundance of both sets using mass spectrometry signal and found no significant differences (data not shown). Relative protein expression levels were determined using the LFQ values from the MaxQuant program. Proteins at the lowest temperature in the thermal assay were subjected to hierarchical clustering. Most treatments displayed broadly similar overall expression profiles, with only minor individual differences apparent at whole dataset scale (Fig. 2A).

In order to assess experimental variability, the TPP-LCMS analysis was initially carried out in duplicate (n = 2 technical replicates). When the correlation of protein expression profiles among these direct replicates was compared to non-replicate pairs, the former were found to be significantly higher (Mann Whiteny test, *p* = 0.0411), confirming that the experimental platform was reproducible (Fig. 2B). All subsequent analysis were performed using the first of these replicates. We next examined correlation between samples in more detail by comparing expression profiles for all pairwise experimental treatments (Fig. 2C). Two crowding agent pairs differing only in MW were highly correlated: Ficoll 70 and Ficoll 400 (Spearman’s ρ = 0.89); dextran 40 and dextran 86 (Spearman’s ρ = 0.88). PEG 1 and PEG 8 showed lower correlation with each other (Spearman’s ρ = 0.63), while PEG8 in particular showed the lowest correlation overall with other treatments. This agreed with Principal Component Analysis, where PEG8 was a significant outlier, accounting for 30% of the variance in the second principal component (Fig. 2D).

### Thermal proteomics of the C.necator response to addition of molecular crowding agents

**Fig. 3:**
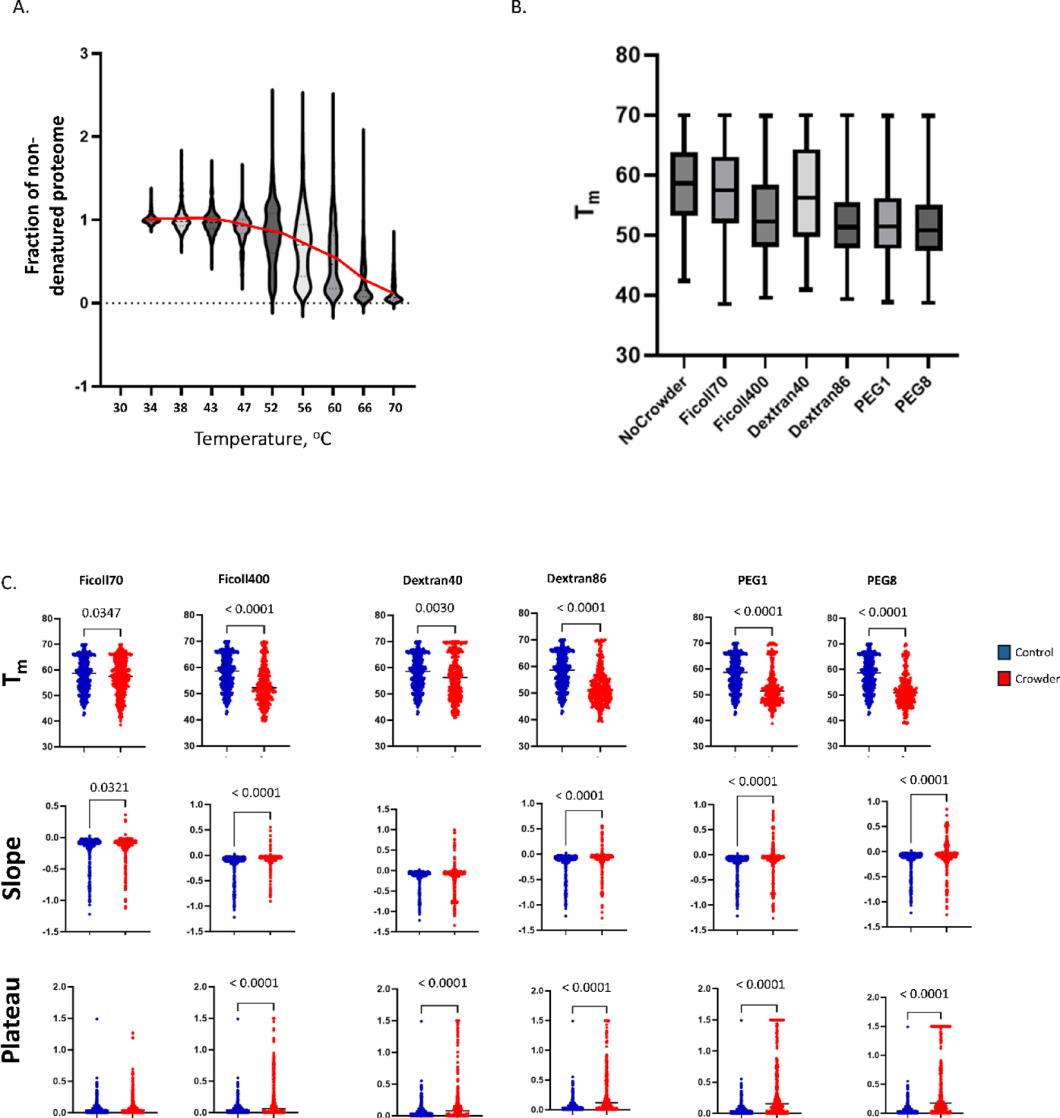
Thermal proteomics analysis of the *C.necator* response to addition of molecular crowding agents. (**A**) Analysis of the fraction of aggregated/denatured protein at each step of the thermal gradient. Values were calculated using TMT-mass tag signals for each data point, normalized to the lowest temperature setting (30 °C). (**B**) Distribution of melting temperature values observed for each crowding agent treatment and control. (**C**) Comparison of the distributions of melting temperature (top), slope (middle), and post-transition plateau (bottom) values for *C.necator* proteins in the presence or absence of crowding agents (Mann Whitney test). Note that the y-axis scales for slope values are not all equivalent.

In TPP experiments, as the proteome is subjected to an increasing temperature gradient, resulting in decreasing proportions of native or folded proteins in the sample (Fig. 3A). The resulting denaturing or aggregating behaviour of the samples in the presence or absence of crowding agents can be fitted to a sigmoid curve using an R package developed by Franken and coworkers^14^. Four parameters are typically calculated from these curves for each protein. The melting temperature (T_m_) reflects the stability of the protein, and is equivalent to the temperature where 50% of the protein is present in soluble form, calculated using the inflection point of the curve. While T_m_ is a widely employed measure of protein relative stability, it should be noted that other parameters describing reversible and kinetic aspects of the unfolding process are not measured using the TPP assay. These include denaturant half concentration and kinetic stability with unfolding rate. Nevertheless, a protein showing a higher T_m_ in one condition compared to another can be considered to be more stable in that condition. When the overall distribution of T_m_ values for all crowding treatments are compared, we found that the mean melting temperatures shifted downwards, suggesting that on average the presence of molecular crowding agents has a destabilizing effect (Fig. 3B). We also observed increased average post-transition plateau values (Fig. 3C). The former may potentially imply a slower rate of unfolding/denaturation in the presence of crowding agents, while the latter may imply that the unfolding/aggregation reactions are not as complete in the presence of crowder as they are in native conditions.

### Crowding agents exhibit concerted stabilizing or destabilizing effects on individual proteins

**Fig. 4:**
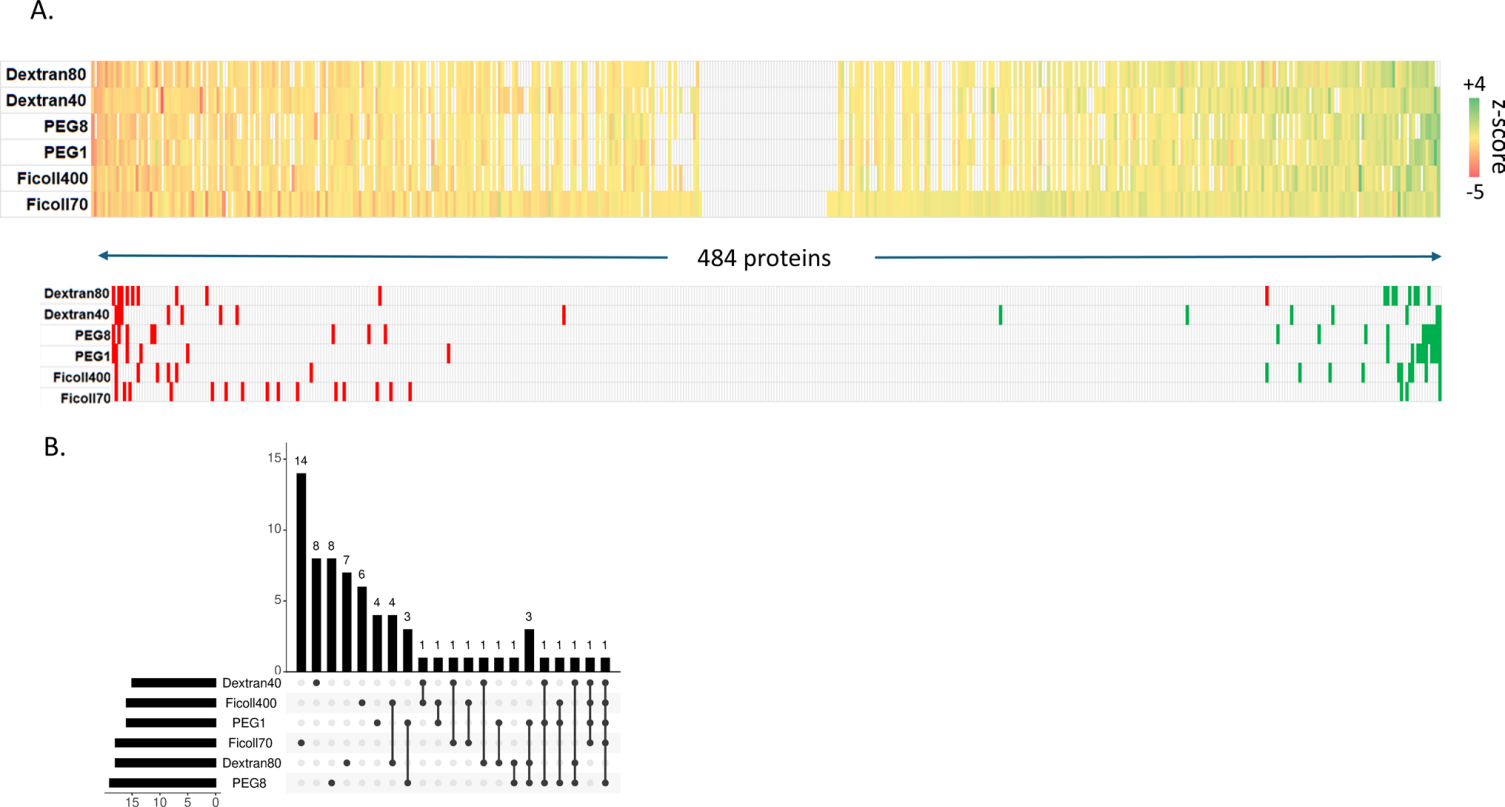

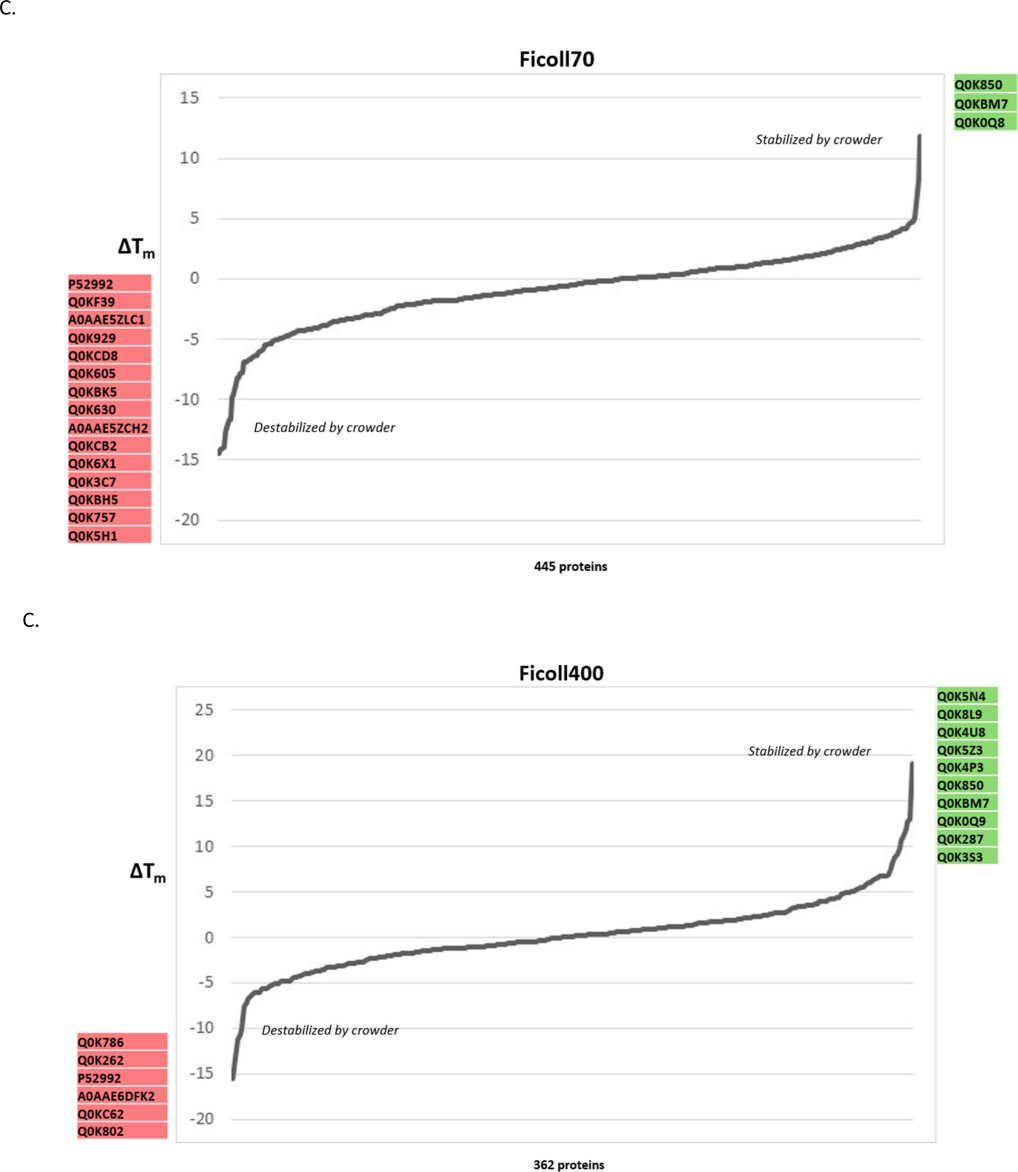

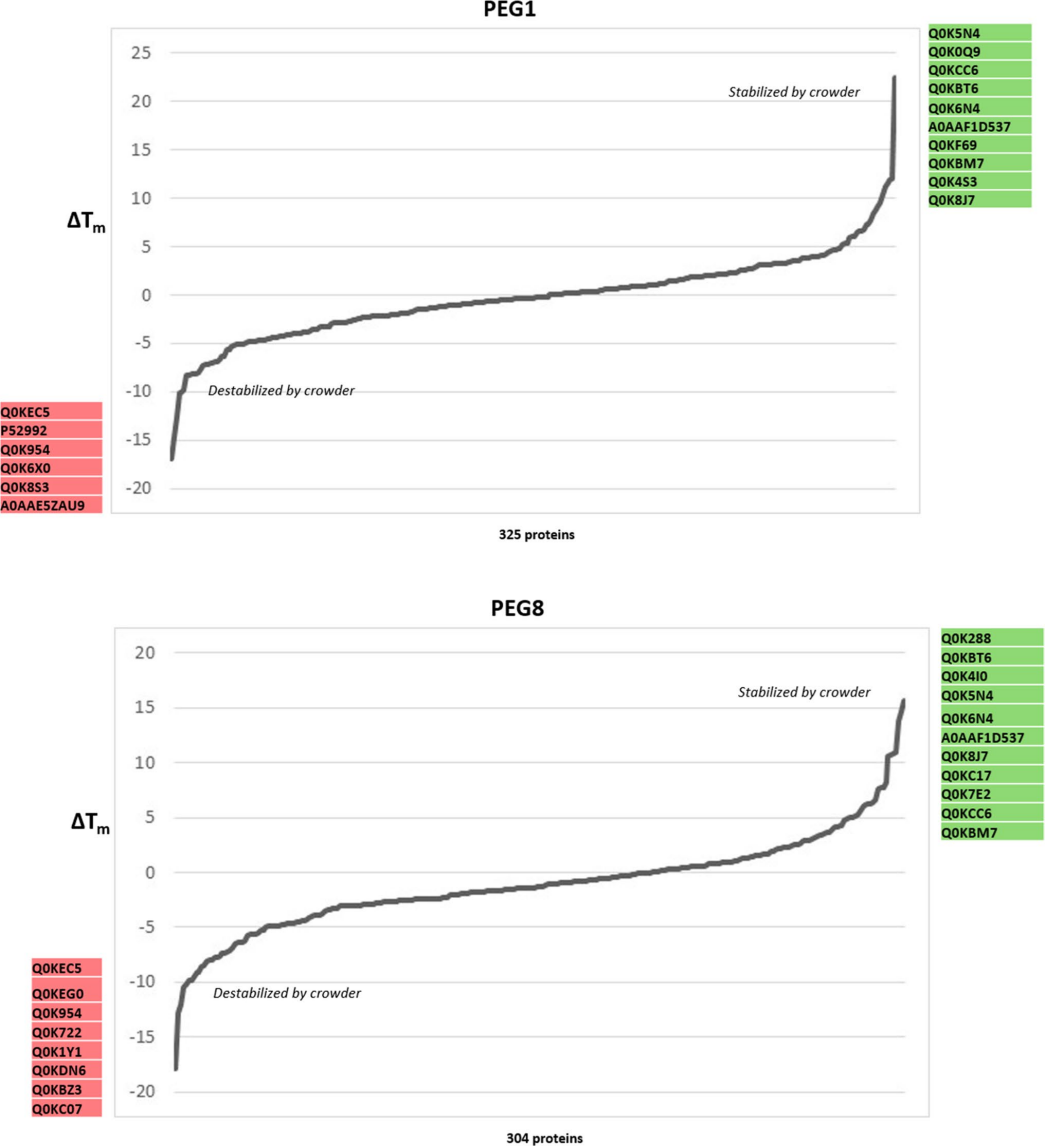

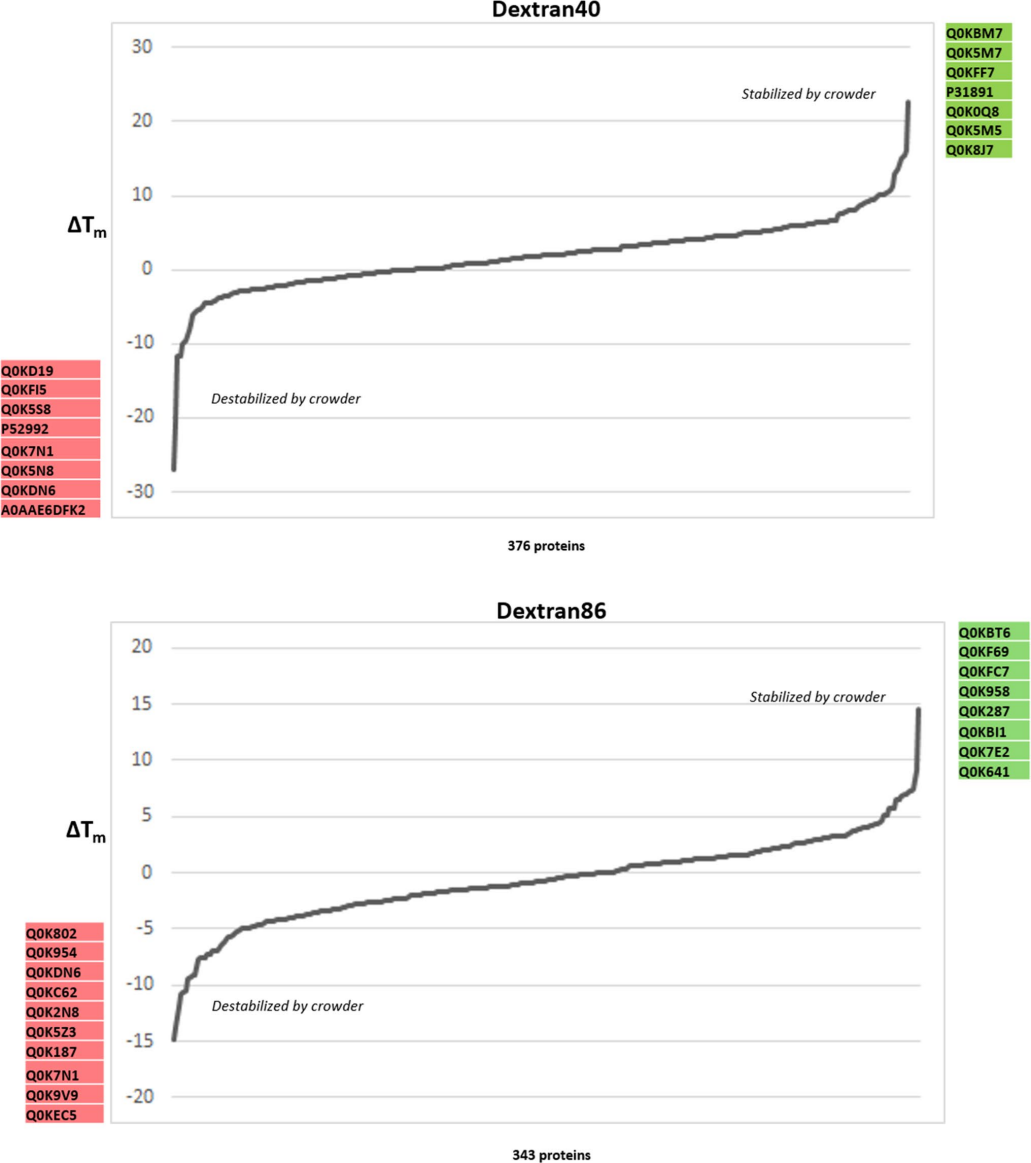

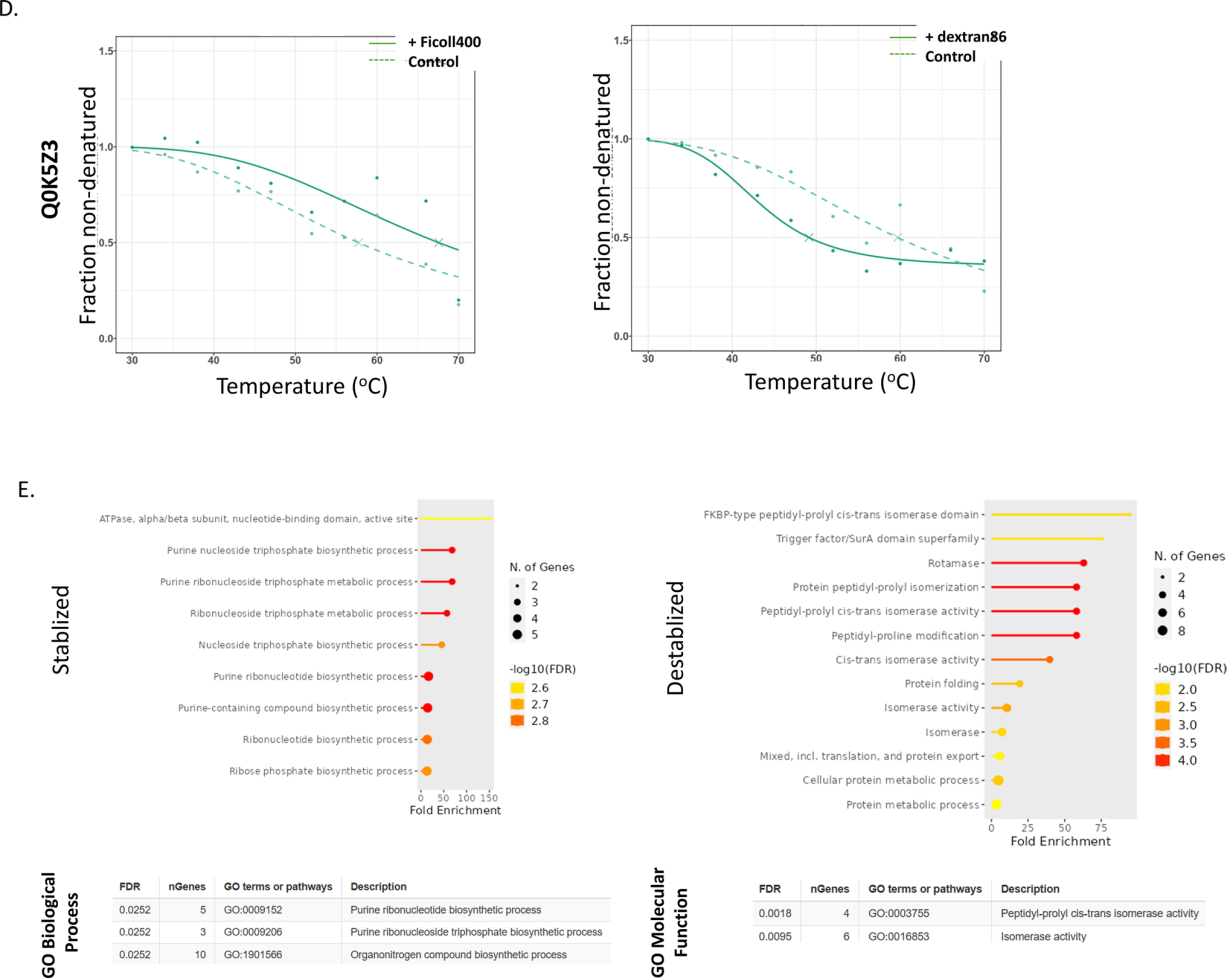
Crowding agents exhibit concerted stabilization or destabilization effects on individual proteins. (**A**) Heat map of z-score values quantifying the stabilization (positive score, green) or destabilization (negative score, red) of individual crowding agents on melting temperature (above). Individual proteins determined to be stabilized (green) or destabilized (red) based on differences in the T_m_ values in the absence and presence of macromolecular crowding agents are highlighted (below). (**B**) Lollipop plot showing the frequency of proteins found to show stabilizing or destabilizing effects for different combinations of crowding agents. (**C**) Rank plots showing the distribution of ΔT_m_ values for individual crowding agents. Uniprot accession codes for significantly stabilized or destabilized proteins are listed by the axes. (**D**) Comparative melting curves for the universal stress protein Q0K5Z3, which shows stabilizing behaviour in the presence of Ficoll 400 and destabilizing behaviour in the presence of dextran 86. (**E**) Gene Ontology keyword enrichment analysis for the set of stabilizing and destabilizing proteins.

We examined ΔT_m_ values (the difference in T_m_ observed before and after the addition of crowding agent, as determined by the TPP procedure) in order to ask: i) which proteins are influenced by the presence of these agents; ii) does the presence of a crowding agent result in a stabilizing or destabilizing effect? Z-scores were calculated for each protein, with those above 1.96 or below −1.96 deemed significant (stabilized or destabilized, respectively; α = 0.01) (Fig. 4A). Sixty-nine protein-crowder pairings were deemed significant: 30 stabilizing, 39 destabilizing (Supplementary Table S3). Among these, 47 proteins were affected by a single crowding agent, 14 by two crowders, six by three crowders, and one protein each affected by either four or five crowding agents (Fig. 4B). Rank order plots of ΔT_m_ for each crowding agent are broadly similar, with most proteins minimally affected by the presence of the agent while a smaller number of proteins at the ends of the plot were found to be more strongly affected (Fig. 4C). While all the plots are broadly symmetric, there is a notable skewness toward the left side of the plot, indicating that proteins are slightly more likely to show reduced than elevated T_m_ under our experimental conditions. Interestingly, these trends are visible across all six crowding agent treatments.

The protein stabilized by five crowding agent treatments (all except dextran80) was alkyl hydroperoxide reductase (Q0KBM7), an antioxidant protein involved in stress response, for which no information appears in the current literature. The protein destabilized in by four crowding treatments, dihydrolipoyl dehydrogenase (P52992), participates in several core metabolic pathways including pyruvate and 2-ketoglutarate production. The three proteins destabilized by three crowding agents are: a DUF5666 domain-containing protein of unknown function (Q0KEC5), the PilH response regulator (Q0KDN6) that may be involved in environmental response and biofilm formation, and a 3’–5’ exonuclease (Q0K954). The three proteins stabilized by three crowding agents are: ferredoxin (Q0K8J7), which is involved in many metabolic pathways as an electron transfer agent, an ABC-type transporter of the HAAT family (Q0K5N4), and an ABC transporter substrate-binding protein (Q0KBT6). No information could be found in the literature addressing the stability of these proteins although as a general class, proteins that respond to stress often employ structural instability or conformational flexibility as part of their response mechanism^20^.

It is notable that with one exception, all the proteins affected by more than one crowding agent, were either exclusively stabilized or exclusively destabilized by the multiple agents. The exception to this is the universal stress protein (Q0K5Z3), stabilized in the presence of Ficoll 400 and destabilized by dextran 86 (Fig. 4D). The exact role of universal stress proteins is unclear, but they are involved in response to diverse stresses including nutrient stress, temperature variation, high salt/osmolarity stress, or oxidative stress in many bacteria. Overall, examination of the sets of stabilized and destabilized proteins show that they play a wide range of functional roles with the cell, with no clearly identifiable trends emerging. Formal Gene Ontology enrichment analysis confirms this impression, finding only overrepresentation for purine ribonucleotide biosynthesis (five proteins stabilized) and isomerase activity (four proteins destabilized) (Fig. 4E).

### Biophysical and functional properties of proteins stabilized or destabilized by molecular crowders

**Fig. 5:**
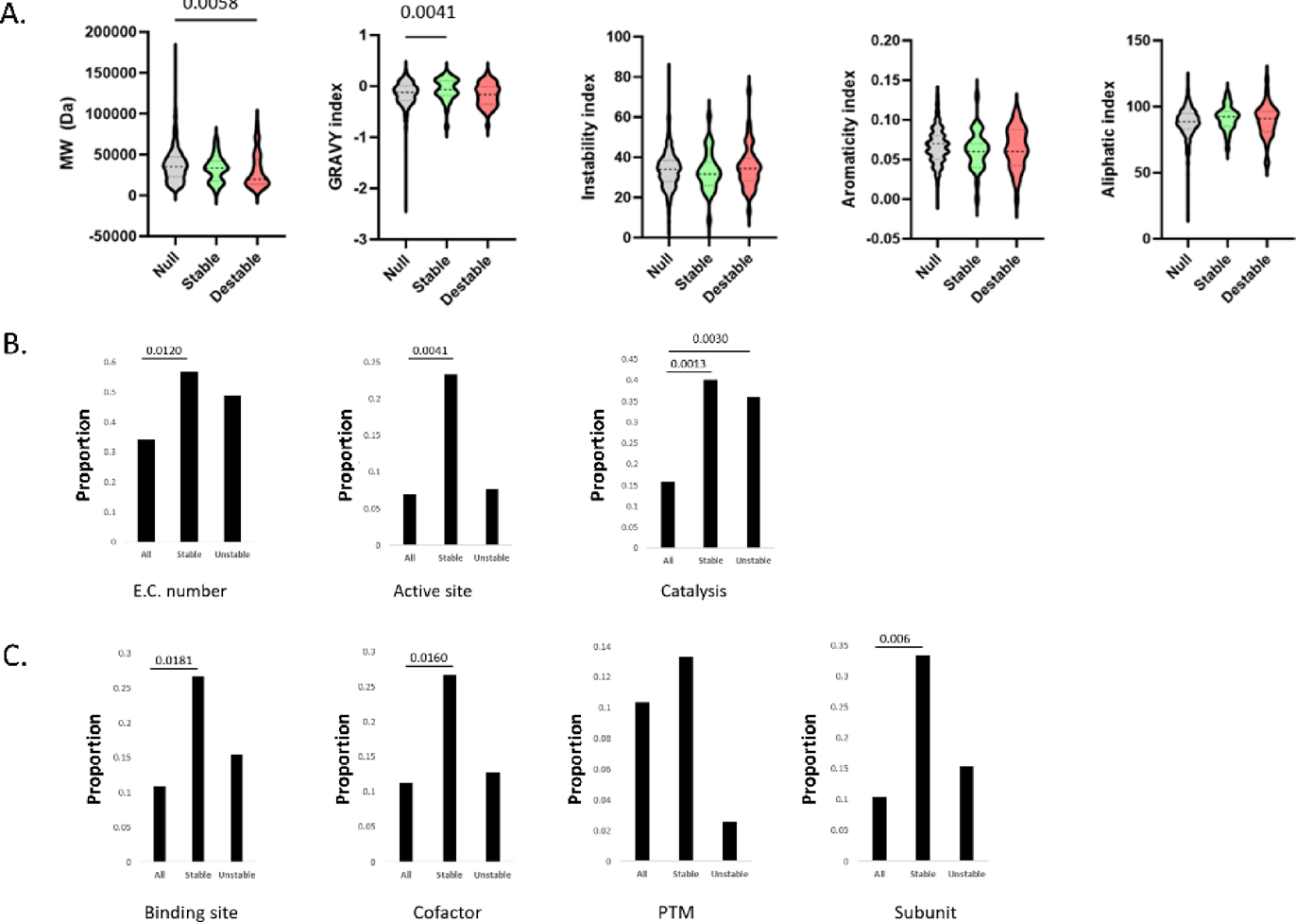
Database-annotated biophysical and functional properties associated with stabilized or destabilized proteins. (**A**) Analysis of global biophysical properties among the sets of stabilized, destabilized, and null proteins. (**B**) Keyword analysis of enzyme-like properties. (**C**) Keyword analysis of protein interaction and modification properties.

We next asked if any biophysical or other properties might be enriched among the sets of stabilized or destabilized proteins. The excluded volume effect is predicted to have a stronger stabilizing effect on larger proteins because the relative change in available volume between the folded and unfolded state is greater for larger molecules. Consistent with this, we found that destabilized proteins are smaller on average than proteins unaffected by crowding, while stabilized proteins have higher GRAVY indices (i.e. are more hydrophobic) (Fig. 5A). Perhaps surprisingly, no association was found for stabilized or destabilized proteins and the properties of instability, aromaticity, aliphaticity, nor the content of any single amino acid (data not shown). The protein size finding is in agreement with predictions from computational models of the *E.coli* cytoplasm that found that macromolecular crowding destabilizes smaller proteins due to increased surface exposure and fewer stabilizing intramolecular interactions. The literature is conflicted however, with a recent review outlining the diversity of experimental findings and context-specific nature of molecular crowding^21^. Analysis of annotated feature enrichments from the Uniprot database suggest preferential stabilization of enzymatic proteins by crowding agents (Fig. 5B). This set of stabilized proteins were more likely to have an Enzyme Commission number, have an active site or catalytic properties (according to analysis of data from the Uniprot database), while destabilized proteins also showed enrichment in catalytic properties (Fig. 5B). There are many reports in the literature of enzyme conformation and activity being influence by the presence of molecular crowding agents, with the findings generally supportive of stabilization based on excluded volume effects ^19,22–26^. Finally, we found that stabilized proteins are enriched in annotated keywords suggestive of involvement in protein interactions (‘binding site’, ‘cofactor’, ‘subunit’), as well as the presence of post-translational modifications (PTMs; Fig. 5C). Interestingly, destabilized proteins displayed the opposite trend, being less likely to have annotated PTMs. There is widespread support in the literature for molecular crowding influencing protein–protein interactions^27^. Both predictive modelling^28^ and large-scale in vivo analysis of bacterial cells^29^ also support a major role for macromolecular crowding on protein interactions.

### Potential mechanisms of stabilization or destabilization by molecular crowders

**Fig. 6:**
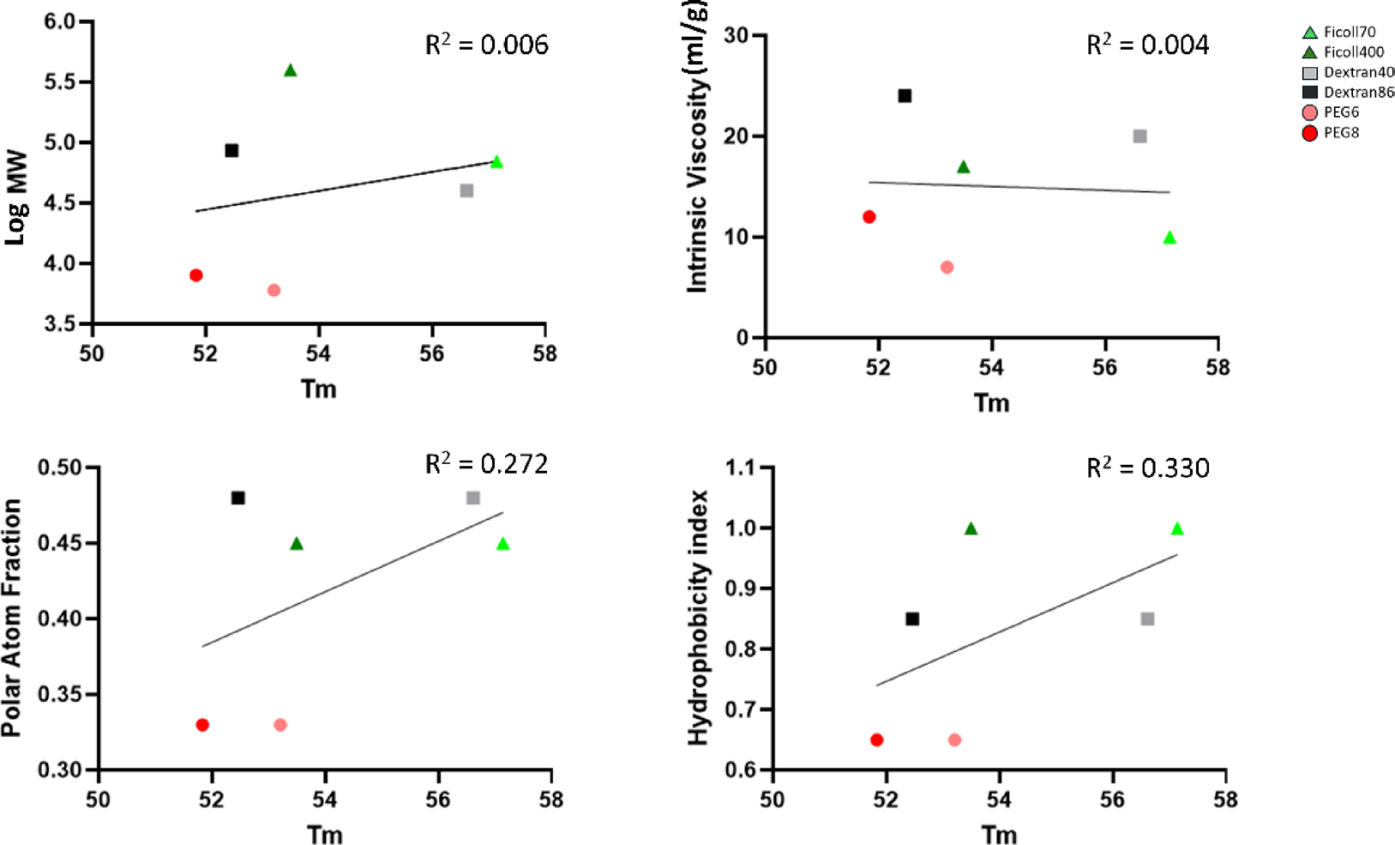
Potential mechanisms of protein stabilization or destabilization by molecular crowders. Correlation analysis of the physico-chemical properties of crowding reagents and the melting temperatures measured in the TPP experiments.

A recent proteomics study of the effect of osmolytes on protein stability found no evidence in favour of crowding or viscosity-based promotion of protein stability, but did find strong correlation with properties associated with a preferential exclusion mechanism, including polar surface area fraction (negative correlation) and free energy change after transfer to the osmolyte (positive correlation)^30^. Preferential exclusion posits that unfavourable interactions between osmolytes and the protein backbone discourages unstable conformational states^31^. Classical osmolytes tend to be naturally occurring low MW organic compounds. In contrast, our work focused on high MW polymeric compounds that are not normally found in the cell. The parameters that Pepelnjak and coworkers used in their analysis (polar surface area, transfer free energy) do not have direct equivalents for high MW polymers like Ficoll, dextran, or PEG. However, we wondered if our experiments provided evidence in support or against these models. In agreement with Pepelnjak and workers, we failed to find evidence of correlation between melting temperature and either MW (or mass concentration) or intrinsic viscosity (Fig. 6). In part agreement with the preferential exclusion model however, we found modest correlation between melting temperature and both the polar atom fraction (R^2^ = 0.272) and the hydrophobicity index (R^2^ = 0.330) for the six compounds tested.

## Discussion

In order to investigate the effects of macromolecular crowding on the proteome, we sought to generate conditions that mimicked the intracellular effects of crowding as closely as possible. However, the introduction of high MW compounds into living cells without compromising cell integrity is challenging. We therefore optimized a procedure where a model bacterial cell is lysed under mild conditions, and the released native proteins are treated with crowding agents at concentrations reported to elicit macromolecular crowding effects (150 mg/ml)^18,32,33^. Protein concentrations were maintained at levels typically employed in proteomics experiments (1 mg/ml), so that when crowding agents were added, the net macromolecule concentration was in line with native conditions^34^. During TPP, the protein/crowding agent mixture is subject to a thermal gradient, with progressive aggregation of individual proteins monitored by mass spectrometry^35^. While this approach allows the relative stability of proteins to be measured on a proteome-wide scale, it comes with caveats. We followed the methodology recommended in TPP methodology papers^2^. However, apparent T_m_ depends strongly on the heating rate (held at 3 min here), and once the proteins reach the temperatures specified in the experiment, they subsequently spend several minutes at room temperature as they are processed in the filtration steps to remove aggregated protein. While these conditions are sufficient to induce a globally detectable T_m_ changes (Fig. 3A), it is possible that there is insufficient time at elevated temperature for some proteins to achieve complete denaturation. Furthermore, the room temperature handling steps provide an opportunity for denatured proteins to refold, potentially complicating the interpretation of T_m_ values. This is a recognized limitation of the TPP method, as the measured ‘stability’ reflects not just the initial unfolding but also the ability of a protein to resist *irreversible* aggregation, as well as its propensity to *reversibly* refold^36^. Furthermore, Tm can be influenced by other factors independent of the temperature, including the protein concentration, and the pH and ionic composition of the buffer (Chan 1995)^37^. Overall however, our approach offers two advantages: first, the aggregation-based T_m_ parameter can be compared between two conditions; second, the approach permits protein stability measurements to be made in a proteome context.

Based on many types of evidence ranging from computer modelling to protein biochemistry experiments to in vivo tracking of fluorescent proteins, a various models have been presented to explain the impact of macromolecular crowding on protein stability^7^. In thermodynamic terms, these models attempt to capture aspects of both the entropic (mainly excluded volume or spatial constraints) and enthalpic (mainly specific interactions) contributions to protein conformation^38^. The diversity of crowding biomolecules present in the cell—including other proteins, nucleic acids, metabolites—makes this experimentally challenging. So-called ‘inert’ crowding agents are often employed in investigation of macromolecular crowding agent effects in an attempt to isolate the entropic component of their thermodynamic effect on protein stability. However, studies of model proteins using a variety of approaches including fluorescent and single molecule spectroscopy, molecular dynamics simulation, differential scanning calorimetry, and nuclear magnetic resonance report that these reagents can in fact engage in chemical interactions with protein^39–41^. Furthermore, many workers have suggested that the in vitro studies of protein function that have historically predominated in biochemistry are a poor surrogate for the dense in vivo conditions in a living cell^42,43^. While in vitro analyses using crowding agents in theory offer a description of the entropic effects of molecular crowders on a single protein species, it is quite far from the physico-chemical reality of a living cell. In reality, no experimental system is likely to reflect the diversity of the molecular environment encountered in even a single cell type (e.g. organelles and other compartments, hydrophobic lipid membranes, pH distributions, solute flux).

We therefore employed a compromise model, where experimental molecular crowding agents were added to a protein lysate obtained from a bacterial cell. This represents a midpoint that, while not an enclosed living cell, represents the entirety of the bacterial proteome at the macromolecular crowding concentrations similar to those encountered in a cell. A strength of this approach is that a large portion of the expressed proteome can be investigated in a single experiment; on the other hand, the sheer complexity of the cellular environment may mask underlying effects. For example, we observed a general trend whereby the addition of molecular crowding agents lowered the mean T_m_ across all measured proteins (F). In reality, this observation includes sets of proteins that showing elevated T_m_ and sets showing lowered T_m_. These confounding effects, as well as the difficulties in isolating enthalpy and entropy contributions (reviewed by Pastore and Temussi^21^), may limit the general conclusions that can be drawn from our study. However, we believe that the approach of studying protein stability effects in a proteome context has value in highlighting individual proteins showing such effects. These merit further investigation using approaches like differential scanning calorimetry in order to validate the observations and investigate underlying mechanisms.

One surprising result was that, on average, melting temperatures were reduced by the addition of all six crowding agents. According to classical molecular crowding theory, the addition of Ficoll, dextran or PEG at high concentrations should increase average protein stability based on the excluded volume effect, which would stabilize proteins by favouring folded conformations. Several factors may explain our observation. Firstly, most earlier studies used single model proteins, while the environment of a living cell is vastly more complex. When averaged across a proteome, melting temperature may decrease upon addition of a crowding agent, masking a more complicated situation where some proteins are stabilized with others destabilized, dependent on how they engage both with the specific reagent, and with the background proteome. In support of this, the recent proteome level study of osmolyte effects found a similar mixture of stabilizing and destabilizing effects^30^. A second possibility is that the presence of crowding agents may negatively influence protein interactions that are important for maintenance of protein stability, for example chaperone interactions. Miklos and coworkers^44^ found that physiological crowding agents (i.e. proteins) could exert mild destabilizing effects on barley chymotrypsin inhibitor 2, a small globular protein, partly through the destabilizing effects of nonspecific interactions.

As mentioned above, a recent TPP analysis of the effects of osmolytes on the proteome used physico-chemical properties of the osmolyte molecules to distinguish between two broad mechanisms of stability—a crowding/viscosity model, and a preferential exclusion model. We were unable to repeat their analysis exactly, due to the polymeric nature of the crowding agents employed, in contrast to osmolytes which are typically monomeric. However, we found moderate evidence that a similar preferential exclusion mechanism may explain the global crowding effects on our bacterial proteome. While there are many differences between the two studies, including the class of stabilizing/destabilizing reagent, different proteome substrates, and different scoring system for stabilization, congruence between both studies suggests that osmolytes and high MW crowding agents may share features of the preferential exclusion mechanism proposed by Street and coworkers^31^.

The use of cell lysates only approximates the native conditions in a living cell since: a) low but functionally efficacious quantities of additives are present (e.g. IGEPAL); b) subcellular organelle and compartment structures are disrupted, even in prokaryotic cells. Another experimental feature that deviates from real life cellular conditions is the use of a single crowding agent. This can result in potentially high levels of interactions involving specific chemistries associated with individual crowding agents. This situation contrasts with normal cell environments where the presence of many different macromolecules lowers the overall concentration any single chemical moiety, hence lowering any overall enthalpic contribution to protein instability. Interestingly, Dewavrin and coworkers^45^ used a collagen polymerization model to show that the presence of multiple crowding agents of different MW acted synergistically in generating higher excluded volume than any single reagent. Finally, the TPP method produces discrete measurements for each protein species—in other words, it does not address local conformation effects at the domain, motif, or single amino acid level. Other approaches such as the Limited Proteolysis method are capable of mapping stability effects at sub-protein resolution and might provide further insights into the nature of the interaction with molecular crowding agents^46^.

## Supplementary Information

Below is the link to the electronic supplementary material.

Supplementary Material 1

Supplementary Material 2

## Data Availability

The mass spectrometry proteomics data have been deposited to the ProteomeXchange Consortium via the PRIDE partner repository with the data set identifier PXD067043. Two supplementary files, one containing all the mass spectrometry outputs (‘SupplementaryTables’), and one containing all the output files from the TPP R script (‘TPPData’) are available on the publisher website or on request from the authors.

## Abbreviations

ACG: Automatic gain control
CETSA: Cellular thermal shift assay
EDTA: Ethylenediaminetetraacetic
HCD: High-energy collision dissociation
HEPES: N-2-hydroxyethylpiperazine-N'-2-ethanesulfonic acid
LCMS: Liquid chromatography-mass spectrometry
LFQ: Label-free quantitation
PBS: Phosphate-buffered saline
PEG: Polyethylene glycol
PTM: Post-translational modification
TMAO: Trimethylamine N-oxide
TMT: Tandem mass tag
TPP: Thermal proteome profiling
