# Supplementary figures and images for: Molecular crowding effects on protein stability in a bacterial proteome (part 2 of 2)
Source: Sci Rep. 2026 Jan 21;16:5908. doi: 10.1038/s41598-026-35990-9 (PMC12894976; doi:10.1038/s41598-026-35990-9)

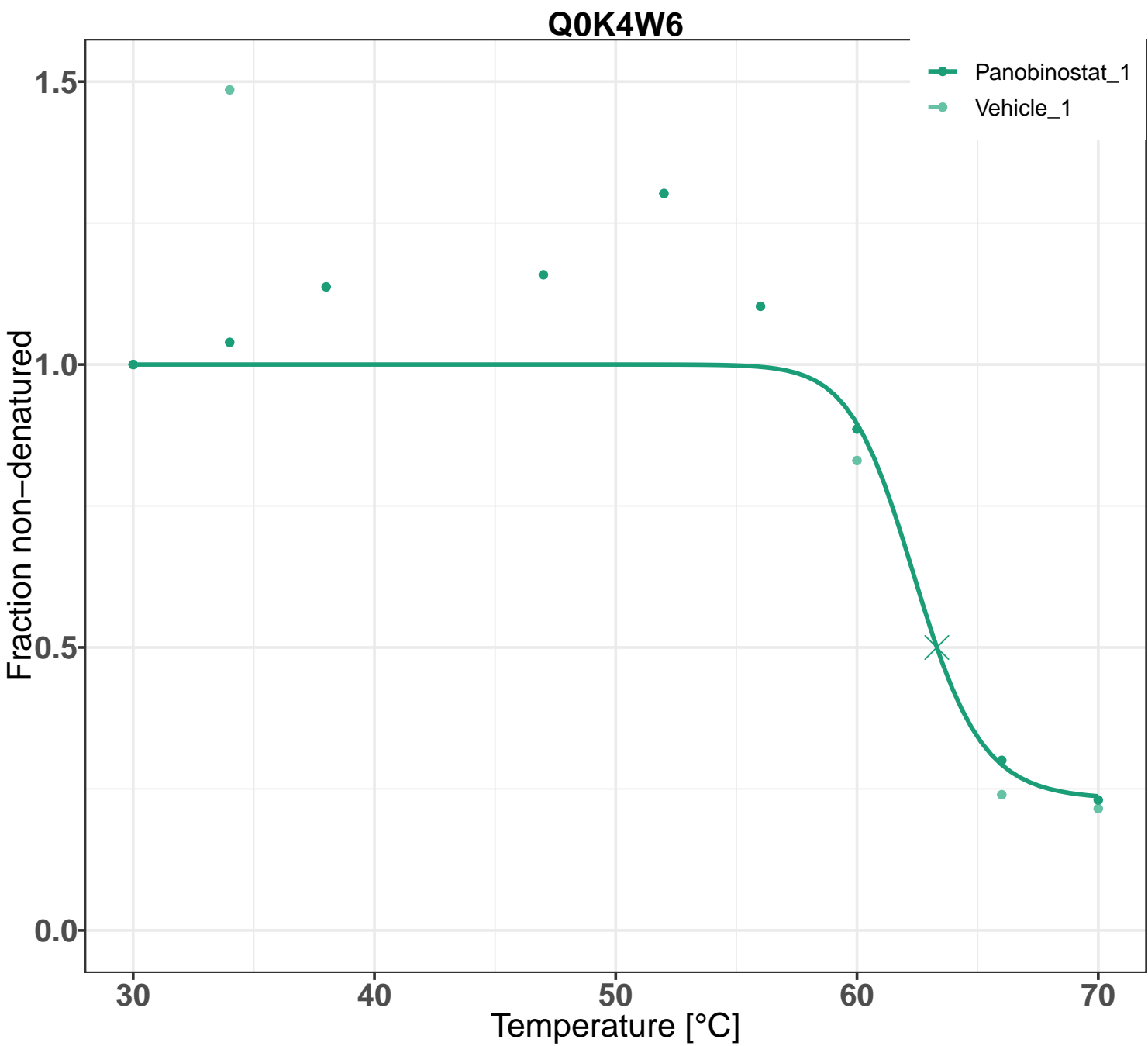

|                | meltPoint | slope | plateau | R2   |
|----------------|-----------|-------|---------|------|
| Panobinostat_1 | 63.31     | -0.14 | 0.23    | 0.55 |
| Vehicle_1      | -         | -     | -       | -    |

Supplement: Supplementary file 2 — Supplementary Material 2 [file 41598_2026_35990_MOESM2_ESM.zip › AllTheTPPData/D40vD86/Panobinostat_Vignette/Melting_Curves/meltCurve_Q0K4W6.pdf]

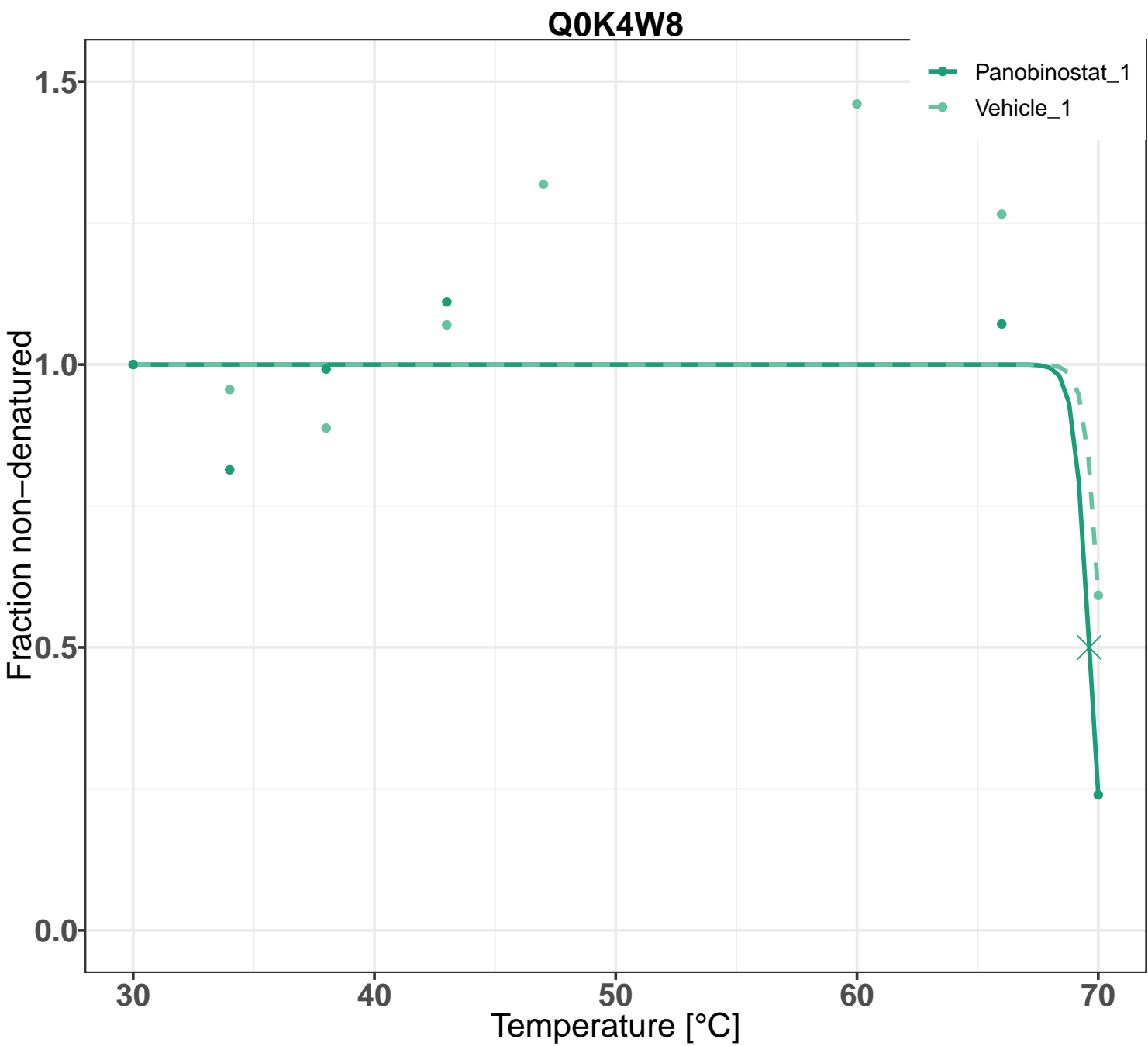

|                | meltPoint | slope | plateau | R2    |
|----------------|-----------|-------|---------|-------|
| Panobinostat_1 | 69.62     | -0.77 | 0       | -0.06 |
| Vehicle_1      | -         | -     | 0       | -0.19 |

Supplement: Supplementary file 2 — Supplementary Material 2 [file 41598_2026_35990_MOESM2_ESM.zip › AllTheTPPData/D40vD86/Panobinostat_Vignette/Melting_Curves/meltCurve_Q0K4W8.pdf]

# Q0K4Z4

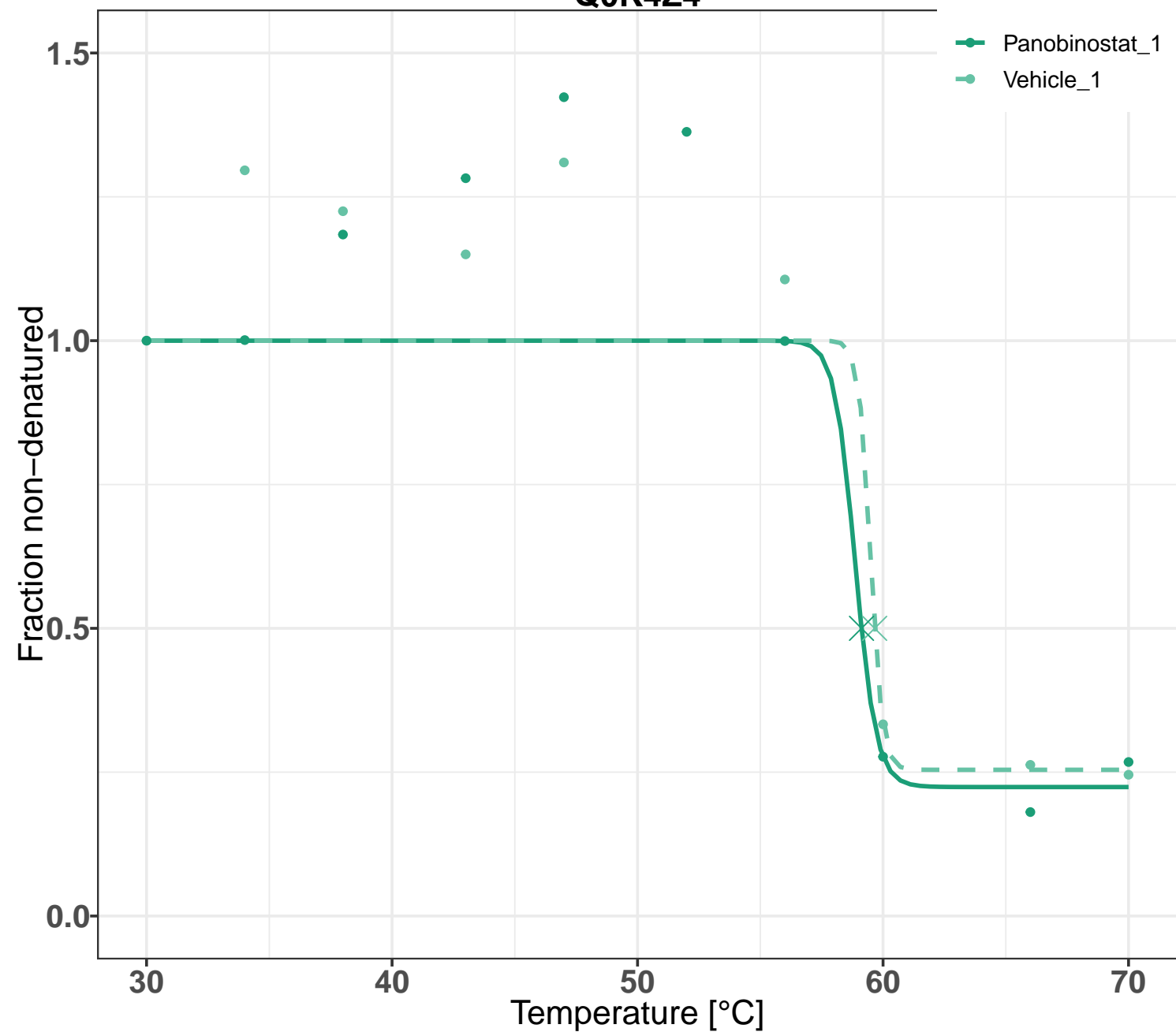

|                | meltPoint | slope | plateau | R2   |
|----------------|-----------|-------|---------|------|
| Panobinostat_1 | 59.12     | -0.46 | 0.22    | 0.79 |
| Vehicle_1      | 59.66     | -0.78 | 0.25    | 0.68 |

Supplement: Supplementary file 2 — Supplementary Material 2 [file 41598_2026_35990_MOESM2_ESM.zip › AllTheTPPData/D40vD86/Panobinostat_Vignette/Melting_Curves/meltCurve_Q0K4Z4.pdf]

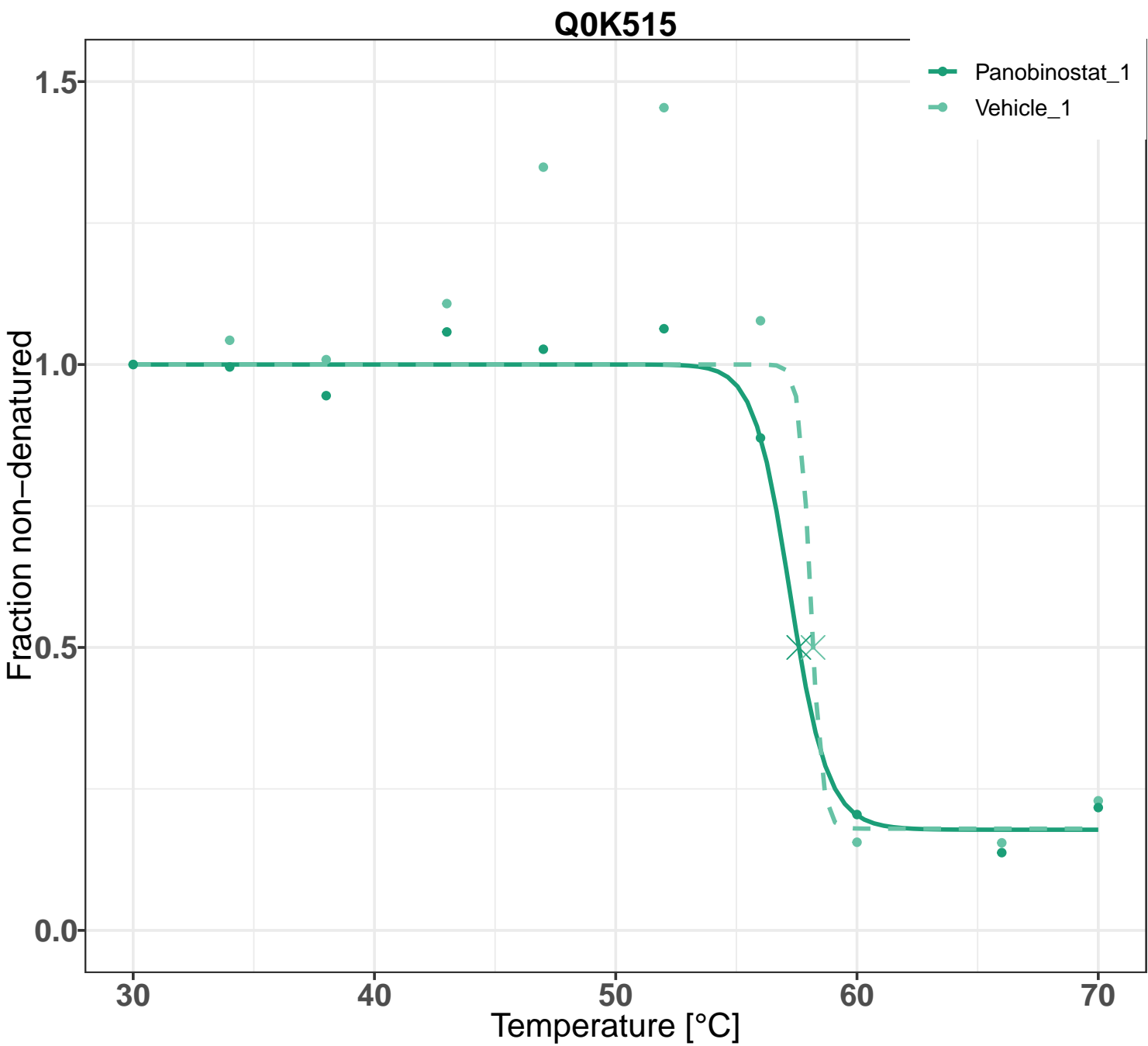

|                | meltPoint | slope | plateau | R2   |
|----------------|-----------|-------|---------|------|
| Panobinostat_1 | 57.59     | -0.27 | 0.18    | 0.99 |
| Vehicle_1      | 58.18     | -0.88 | 0.18    | 0.84 |

Supplement: Supplementary file 2 — Supplementary Material 2 [file 41598_2026_35990_MOESM2_ESM.zip › AllTheTPPData/D40vD86/Panobinostat_Vignette/Melting_Curves/meltCurve_Q0K515.pdf]

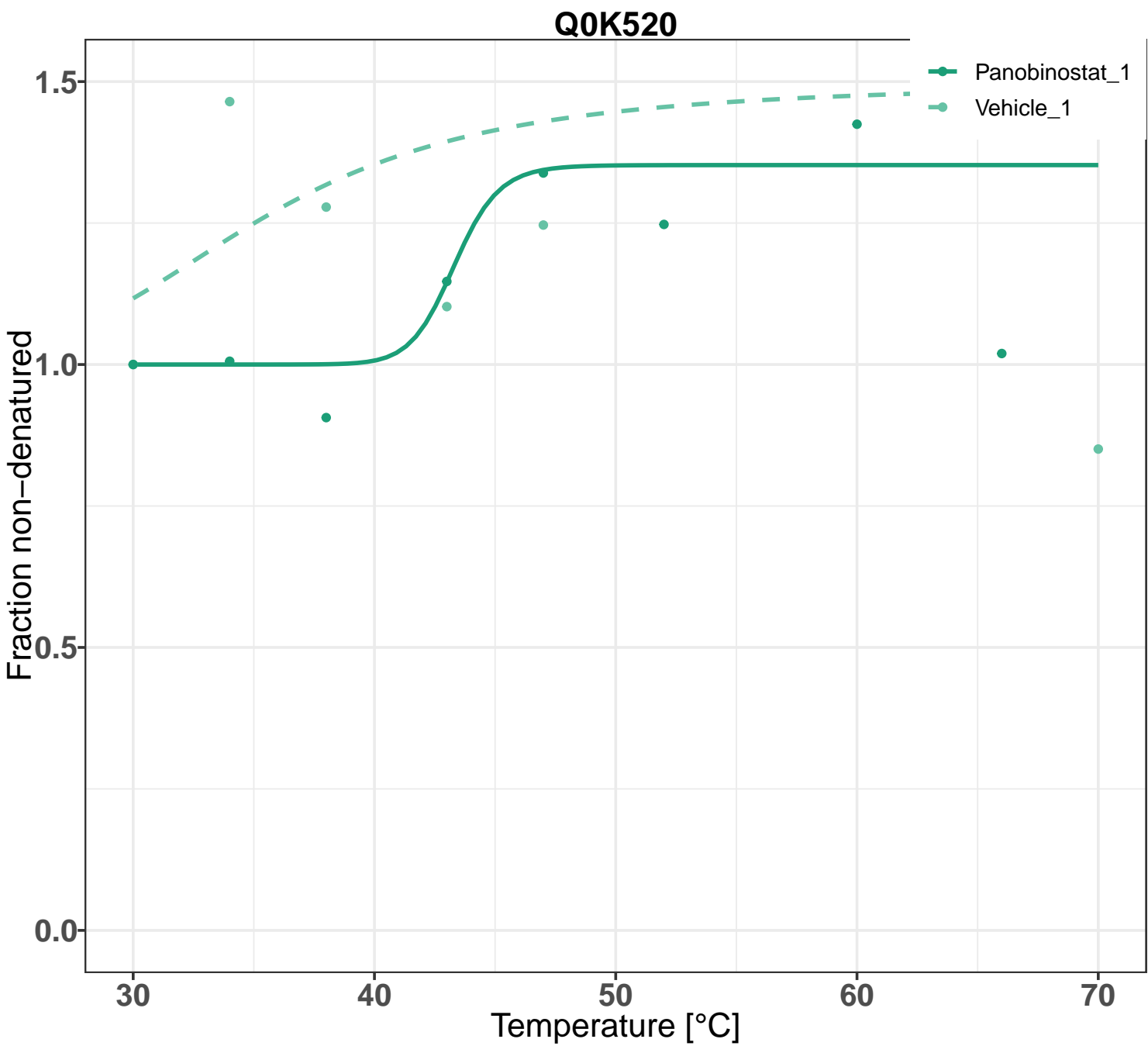

|                | meltPoint | slope | plateau | R2   |
|----------------|-----------|-------|---------|------|
| Panobinostat_1 | —         | 0.096 | 1.35    | 0.59 |
| Vehicle_1      | —         | 0.027 | 1.5     | 0.14 |

Supplement: Supplementary file 2 — Supplementary Material 2 [file 41598_2026_35990_MOESM2_ESM.zip › AllTheTPPData/D40vD86/Panobinostat_Vignette/Melting_Curves/meltCurve_Q0K520.pdf]

# Q0K548

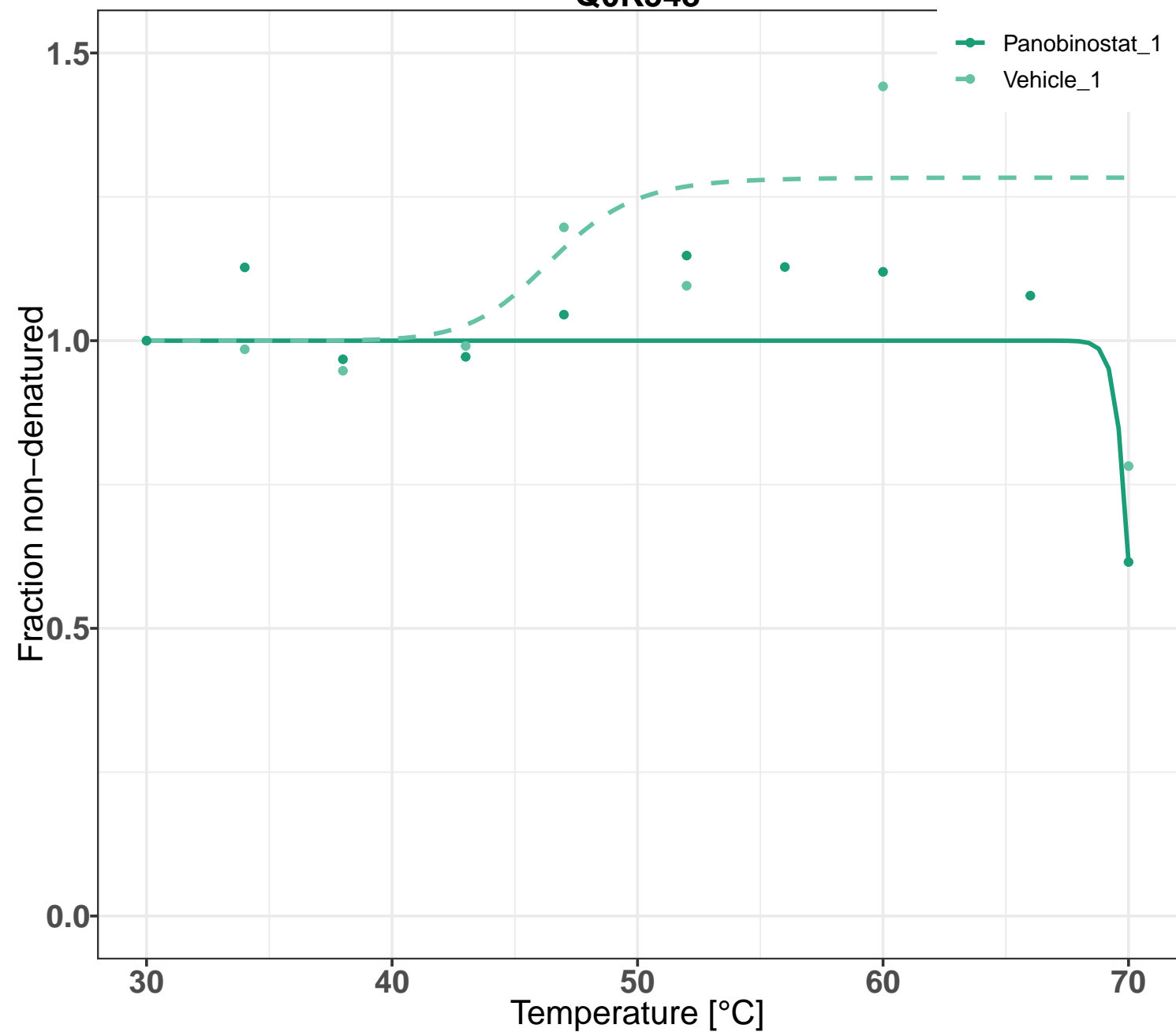

|                | meltPoint | slope | plateau | R2   |
|----------------|-----------|-------|---------|------|
| Panobinostat_1 | –         | –     | 0       | 0.65 |
| Vehicle_1      | –         | 0.042 | 1.28    | 0.31 |

Supplement: Supplementary file 2 — Supplementary Material 2 [file 41598_2026_35990_MOESM2_ESM.zip › AllTheTPPData/D40vD86/Panobinostat_Vignette/Melting_Curves/meltCurve_Q0K548.pdf]

# Q0K557

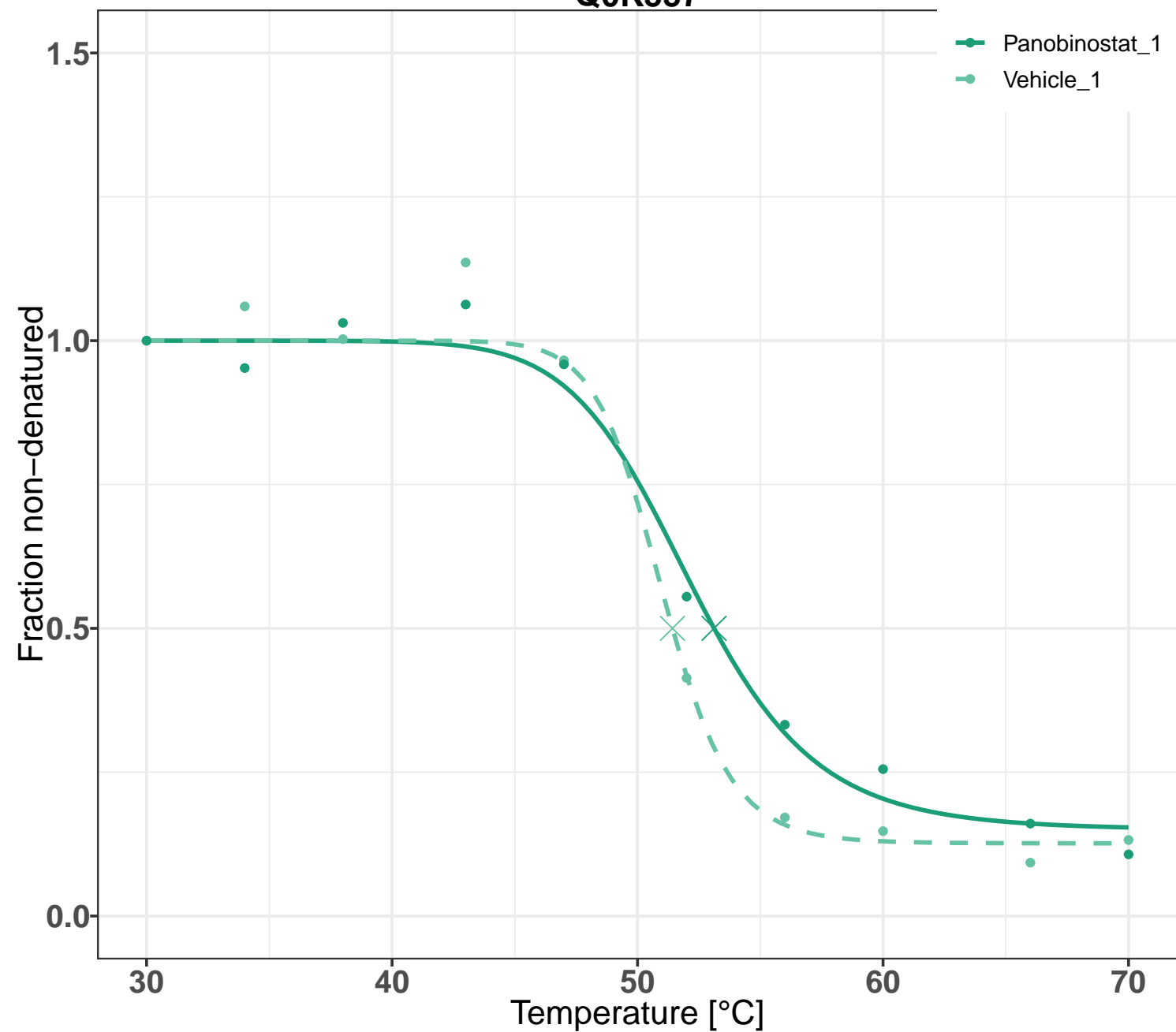

meltPoint

slope

plateau

R2

Panobinostat\_1

53.12

-0.085

0.15

0.99

Vehicle\_1

51.42

-0.16

0.13

0.99

Supplement: Supplementary file 2 — Supplementary Material 2 [file 41598_2026_35990_MOESM2_ESM.zip › AllTheTPPData/D40vD86/Panobinostat_Vignette/Melting_Curves/meltCurve_Q0K557.pdf]

# Q0K565

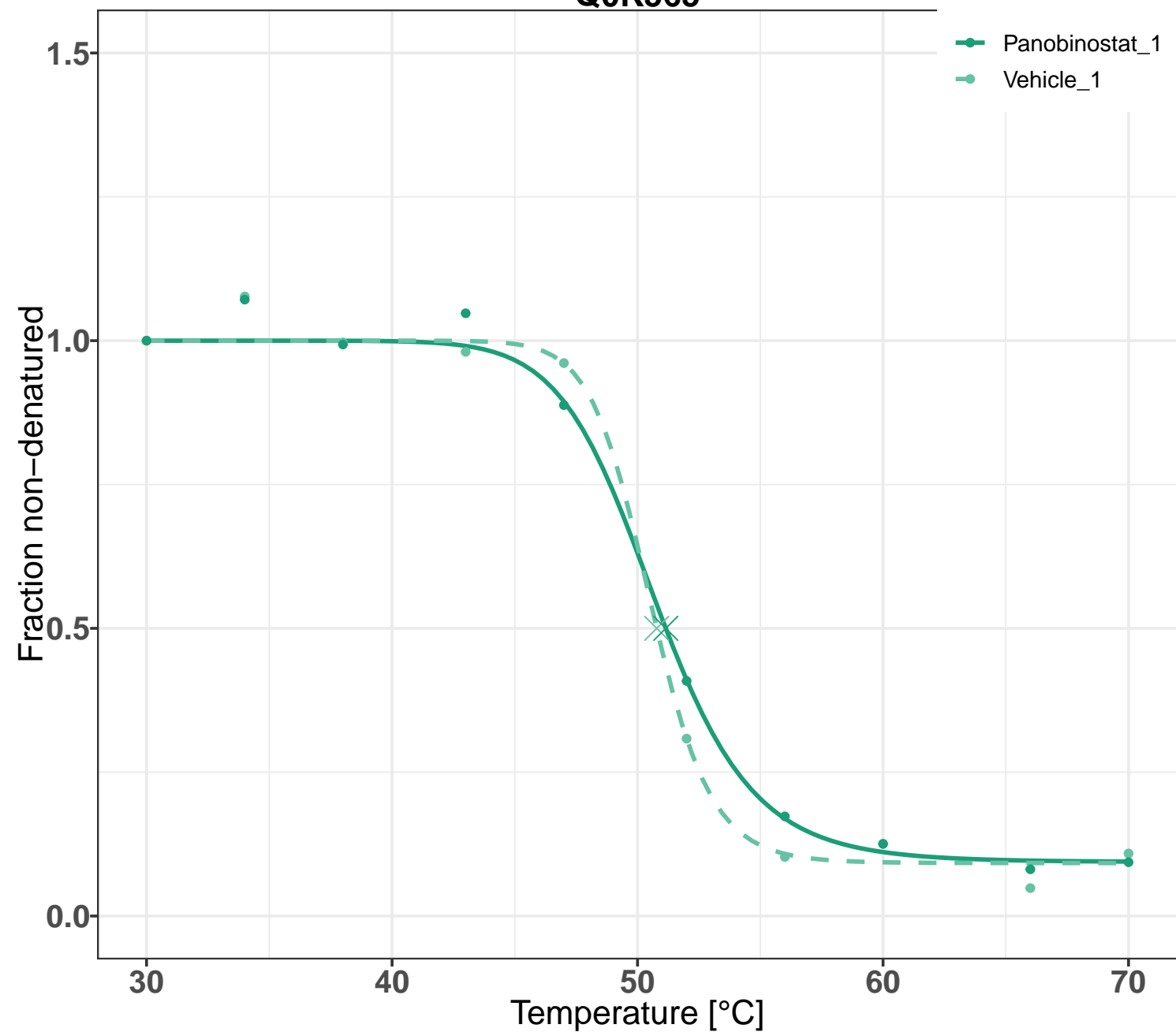

meltPoint

slope

plateau

R2

Panobinostat\_1

51.15

-0.11

0.09

1

Vehicle\_1

50.78

-0.19

0.09

1

Supplement: Supplementary file 2 — Supplementary Material 2 [file 41598_2026_35990_MOESM2_ESM.zip › AllTheTPPData/D40vD86/Panobinostat_Vignette/Melting_Curves/meltCurve_Q0K565.pdf]

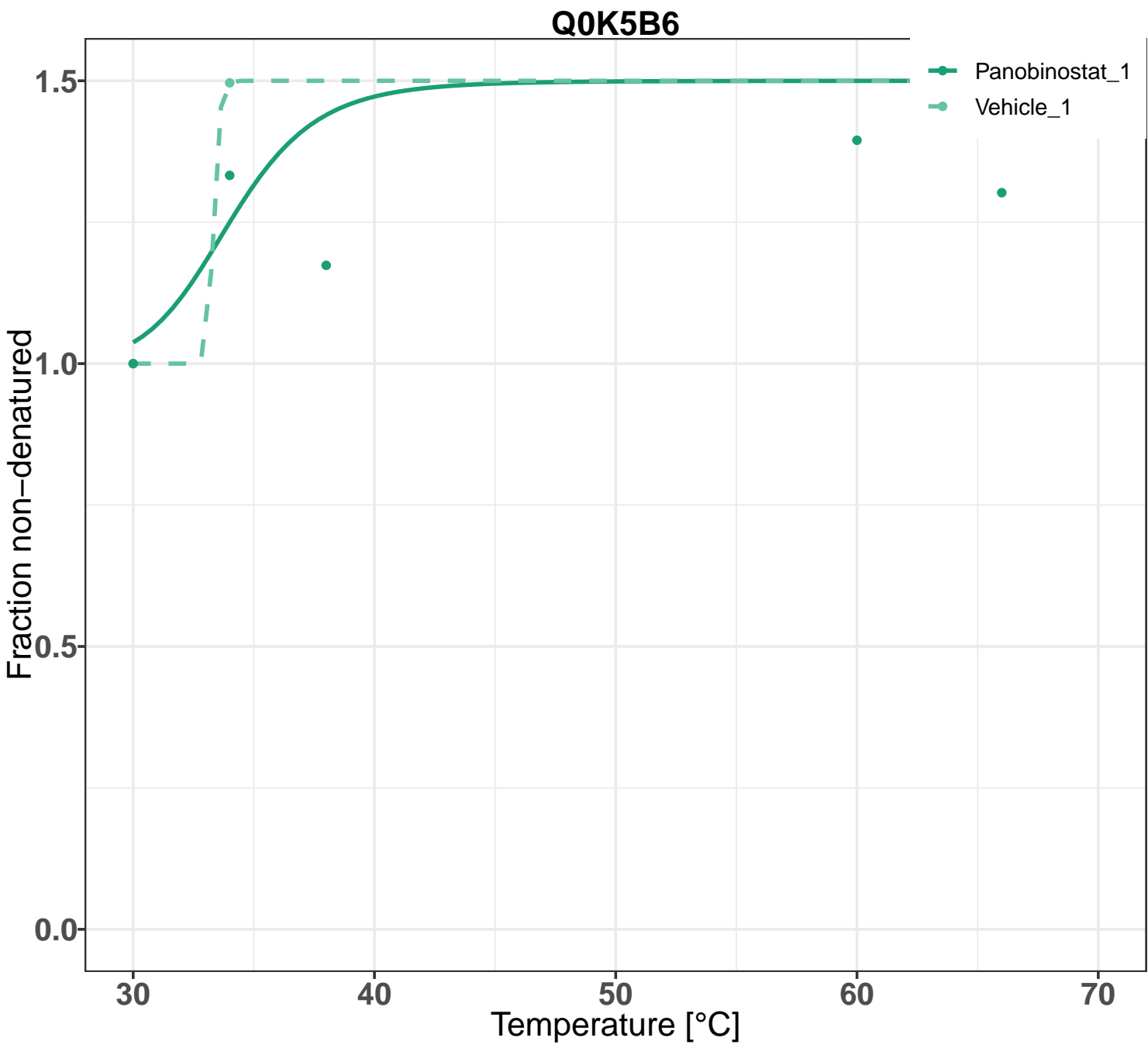

|                | meltPoint | slope | plateau | R2    |
|----------------|-----------|-------|---------|-------|
| Panobinostat_1 | –         | 0.07  | 1.5     | –0.07 |
| Vehicle_1      | –         | 0.94  | 1.5     | –0.7  |

Supplement: Supplementary file 2 — Supplementary Material 2 [file 41598_2026_35990_MOESM2_ESM.zip › AllTheTPPData/D40vD86/Panobinostat_Vignette/Melting_Curves/meltCurve_Q0K5B6.pdf]

Q0K5E7

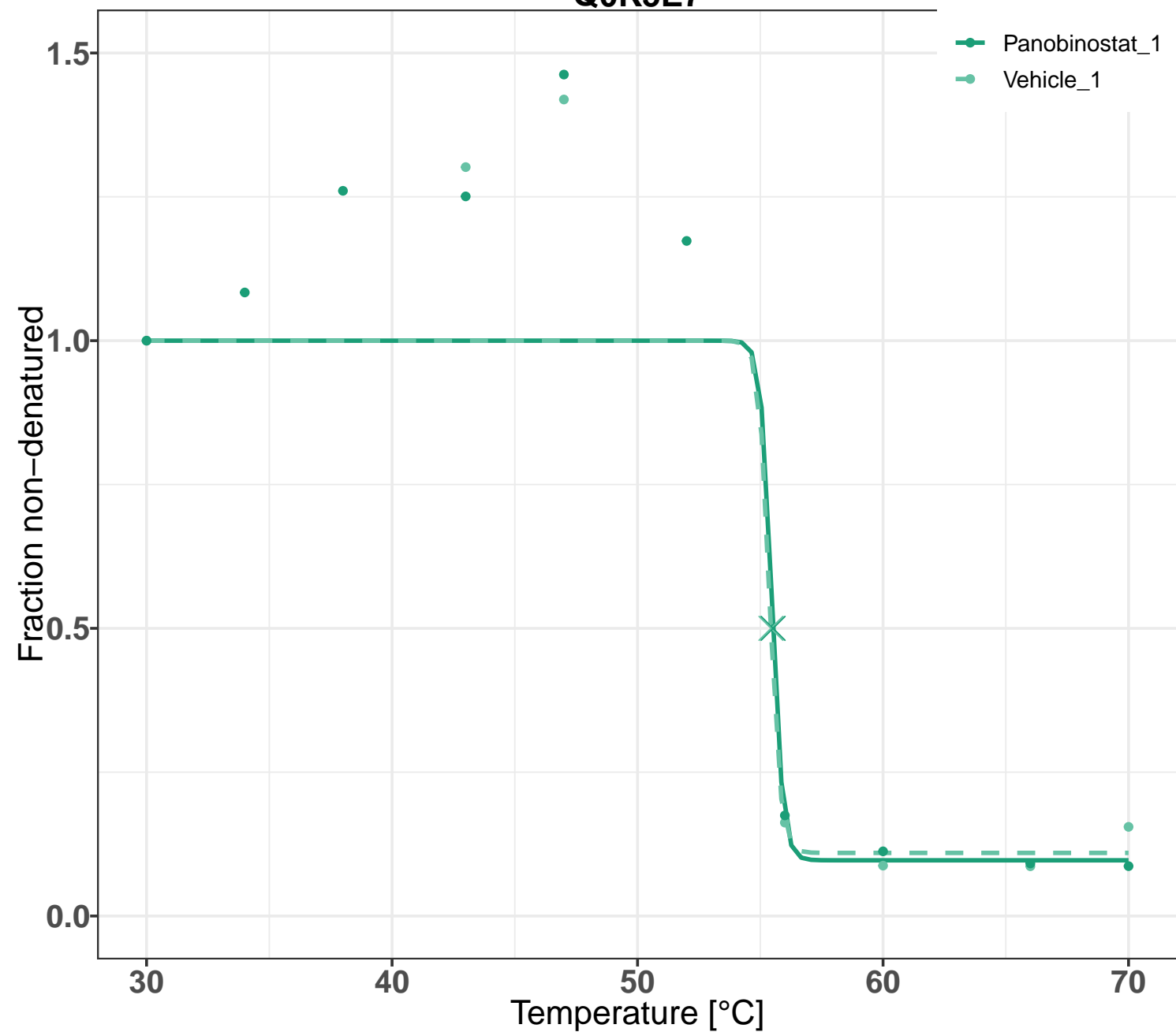

meltPoint

slope

plateau

R2

Panobinostat\_1

55.52

-1

0.1

0.87

Vehicle\_1

55.44

-1

0.11

0.71

Supplement: Supplementary file 2 — Supplementary Material 2 [file 41598_2026_35990_MOESM2_ESM.zip › AllTheTPPData/D40vD86/Panobinostat_Vignette/Melting_Curves/meltCurve_Q0K5E7.pdf]

# Q0K5G4

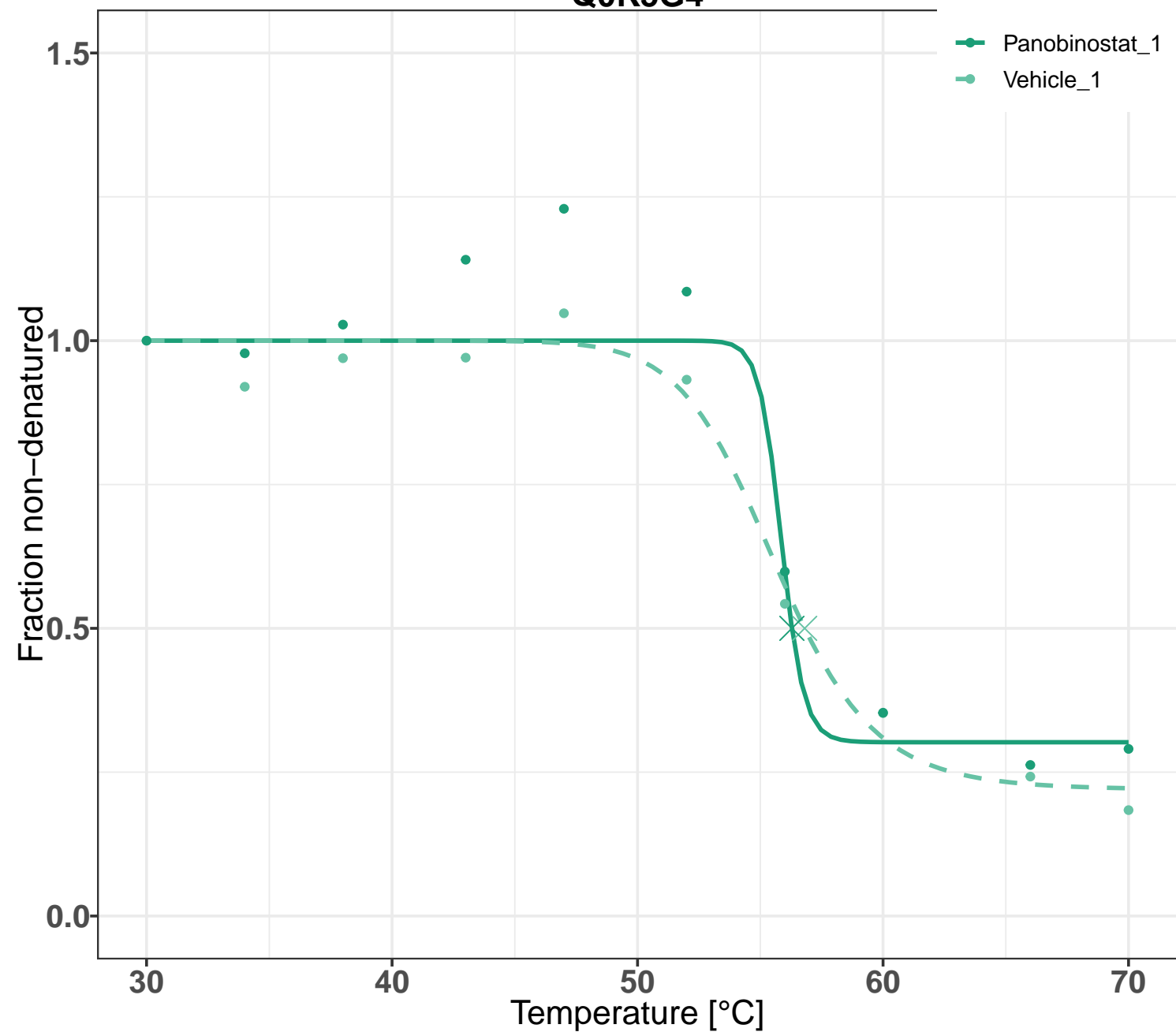

|                | meltPoint | slope  | plateau | R2   |
|----------------|-----------|--------|---------|------|
| Panobinostat_1 | 56.29     | -0.38  | 0.3     | 0.93 |
| Vehicle_1      | 56.81     | -0.099 | 0.22    | 0.98 |

Supplement: Supplementary file 2 — Supplementary Material 2 [file 41598_2026_35990_MOESM2_ESM.zip › AllTheTPPData/D40vD86/Panobinostat_Vignette/Melting_Curves/meltCurve_Q0K5G4.pdf]

# Q0K5H1

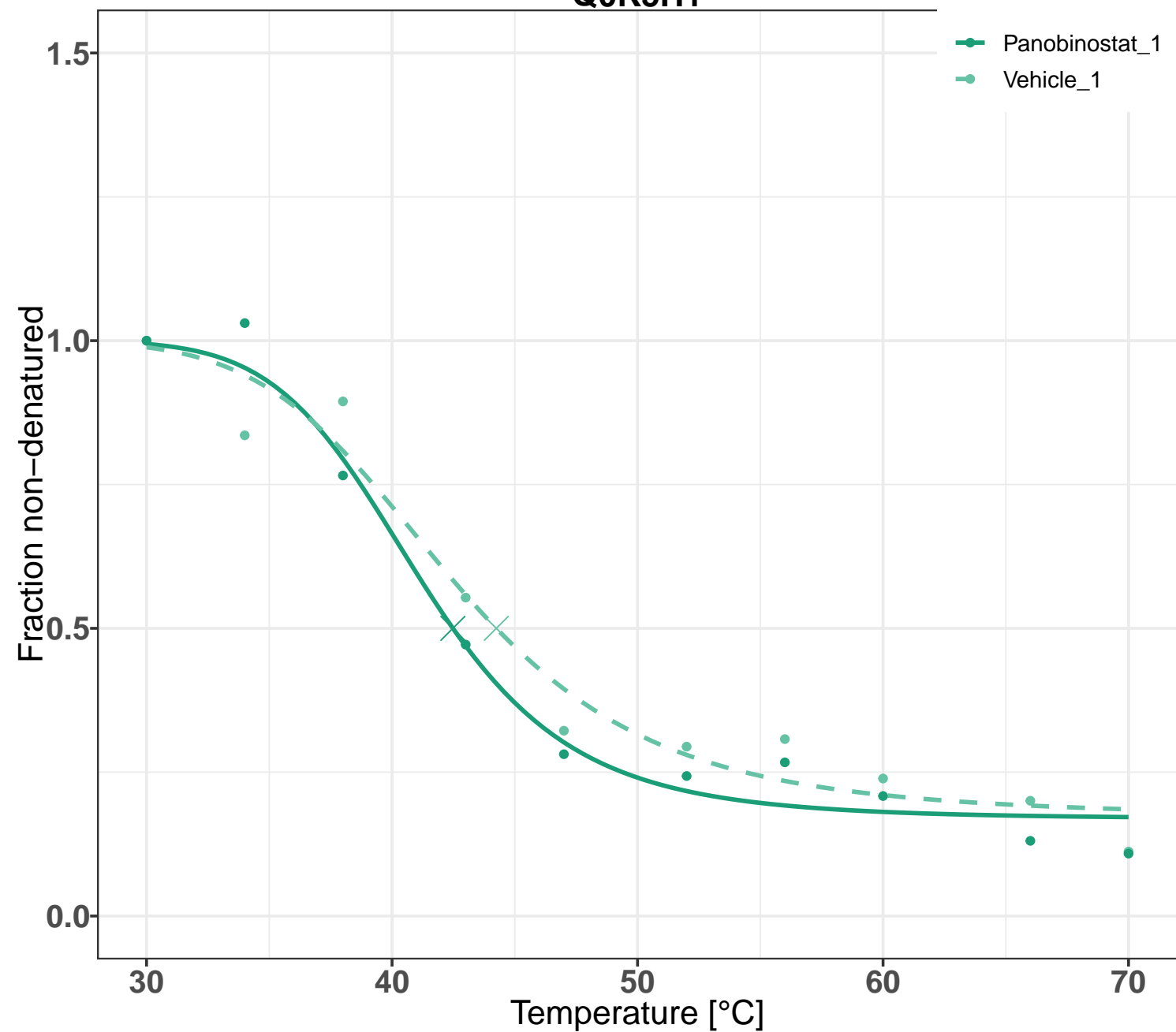

**meltPoint**

**slope**

**plateau**

**R2**

**Panobinostat\_1**

**42.48**

**-0.069**

**0.17**

**0.98**

**Vehicle\_1**

**44.25**

**-0.052**

**0.17**

**0.96**

Supplement: Supplementary file 2 — Supplementary Material 2 [file 41598_2026_35990_MOESM2_ESM.zip › AllTheTPPData/D40vD86/Panobinostat_Vignette/Melting_Curves/meltCurve_Q0K5H1.pdf]

# Q0K5H6

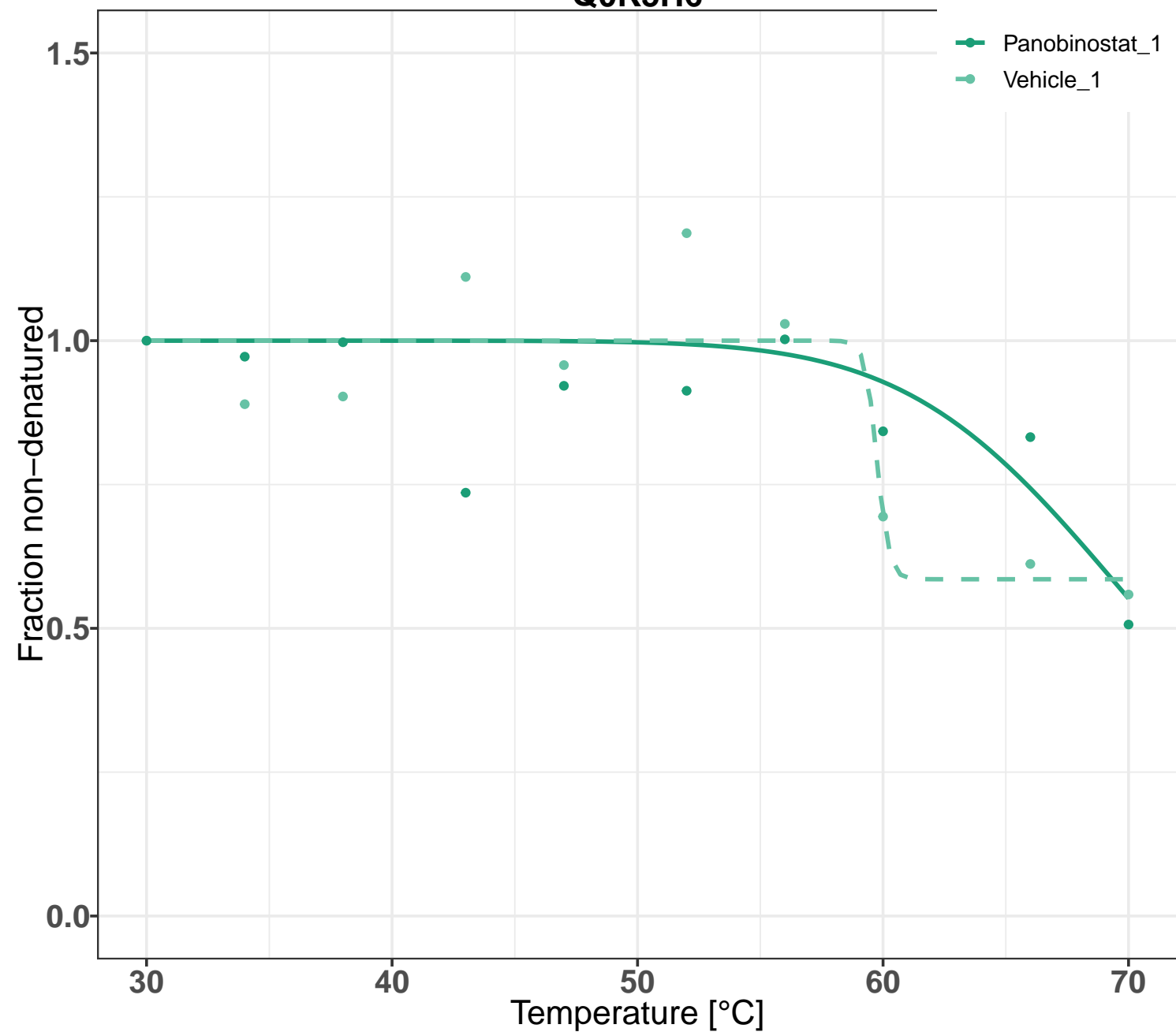

meltPoint

slope

plateau

R2

Panobinostat\_1

—

-0.05

0

0.54

Vehicle\_1

—

-0.43

0.59

0.82

Supplement: Supplementary file 2 — Supplementary Material 2 [file 41598_2026_35990_MOESM2_ESM.zip › AllTheTPPData/D40vD86/Panobinostat_Vignette/Melting_Curves/meltCurve_Q0K5H6.pdf]

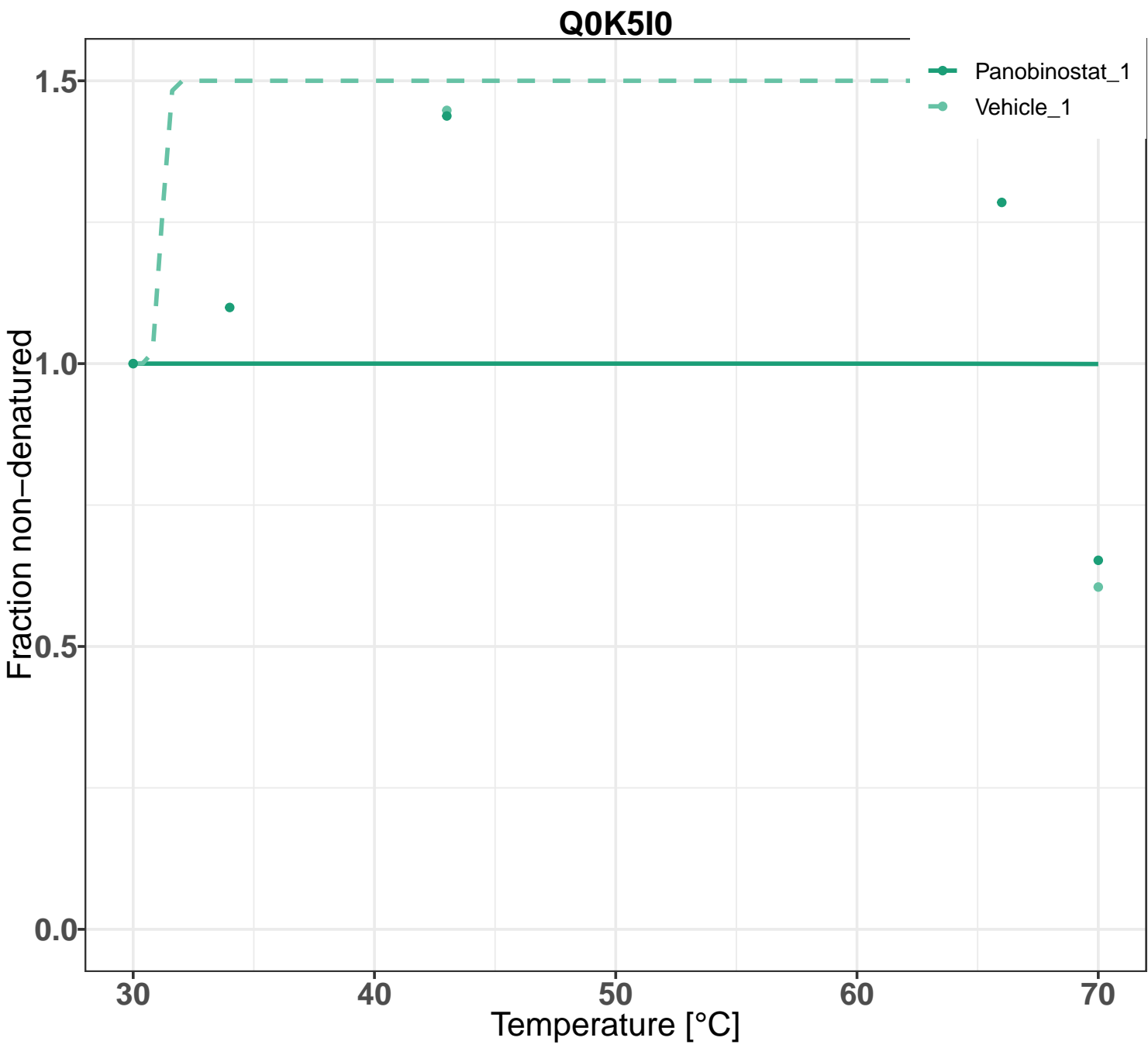

|                | meltPoint | slope | plateau | R2    |
|----------------|-----------|-------|---------|-------|
| Panobinostat_1 | –         | –     | 0.09    | –1.05 |
| Vehicle_1      | –         | 1     | 1.5     | –0.35 |

Supplement: Supplementary file 2 — Supplementary Material 2 [file 41598_2026_35990_MOESM2_ESM.zip › AllTheTPPData/D40vD86/Panobinostat_Vignette/Melting_Curves/meltCurve_Q0K5I0.pdf]

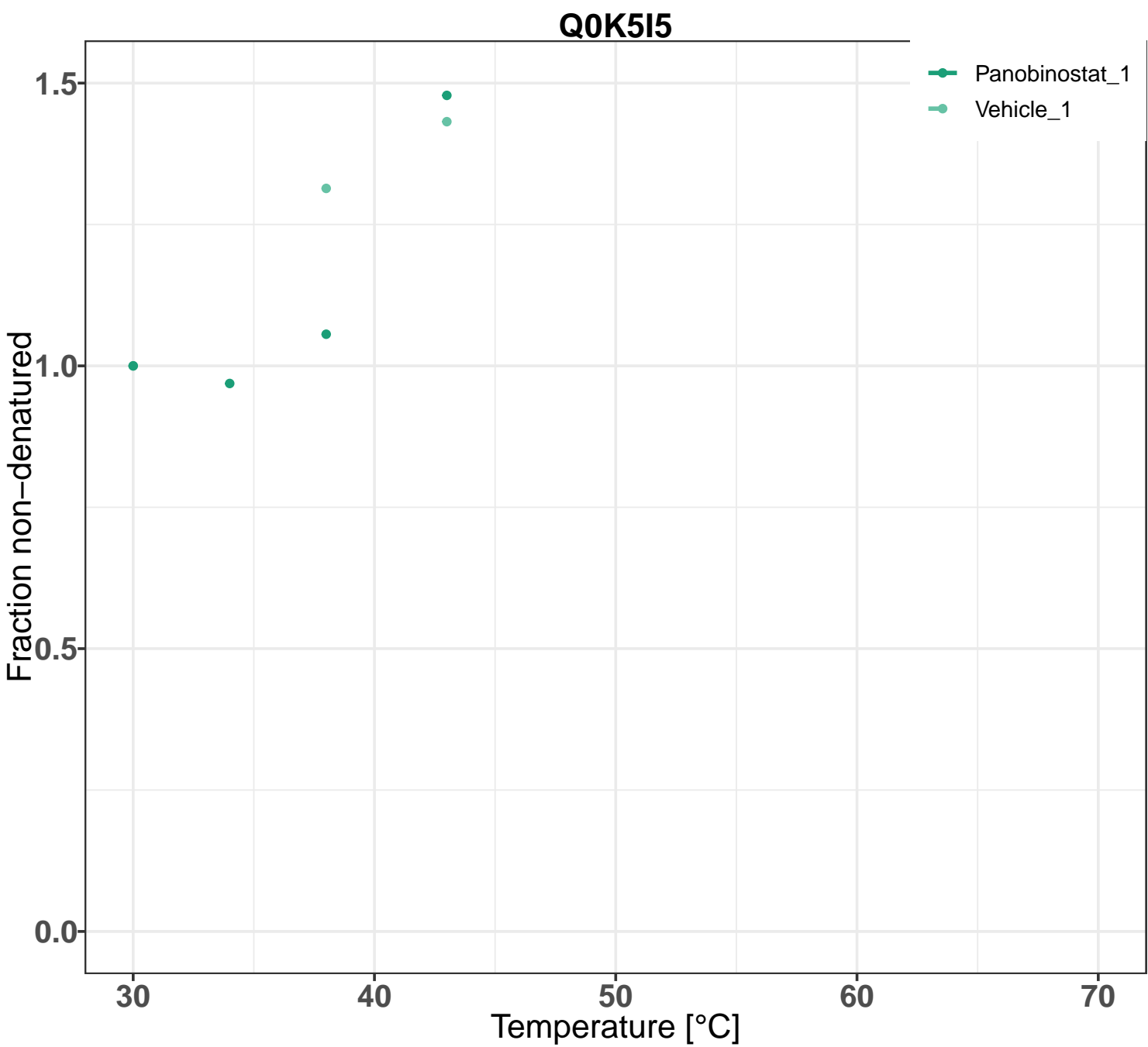

|                | meltPoint | slope | plateau | R2 |
|----------------|-----------|-------|---------|----|
| Panobinostat_1 | —         | —     | —       | —  |
| Vehicle_1      | —         | —     | —       | —  |

Supplement: Supplementary file 2 — Supplementary Material 2 [file 41598_2026_35990_MOESM2_ESM.zip › AllTheTPPData/D40vD86/Panobinostat_Vignette/Melting_Curves/meltCurve_Q0K5I5.pdf]

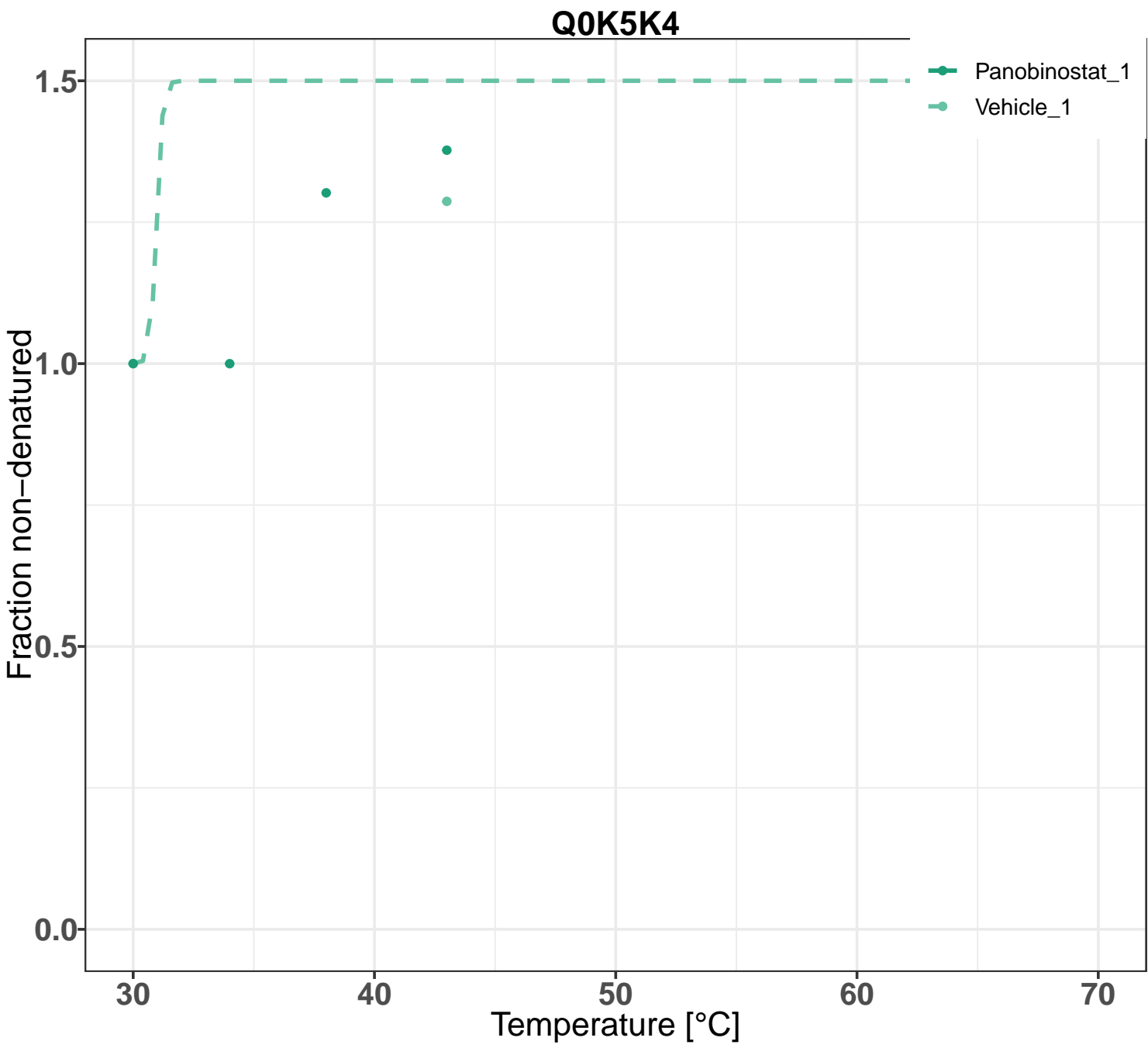

|                | meltPoint | slope | plateau | R2    |
|----------------|-----------|-------|---------|-------|
| Panobinostat_1 | -         | -     | -       | -     |
| Vehicle_1      | -         | 1     | 1.5     | -0.67 |

Supplement: Supplementary file 2 — Supplementary Material 2 [file 41598_2026_35990_MOESM2_ESM.zip › AllTheTPPData/D40vD86/Panobinostat_Vignette/Melting_Curves/meltCurve_Q0K5K4.pdf]

# Q0K5M5

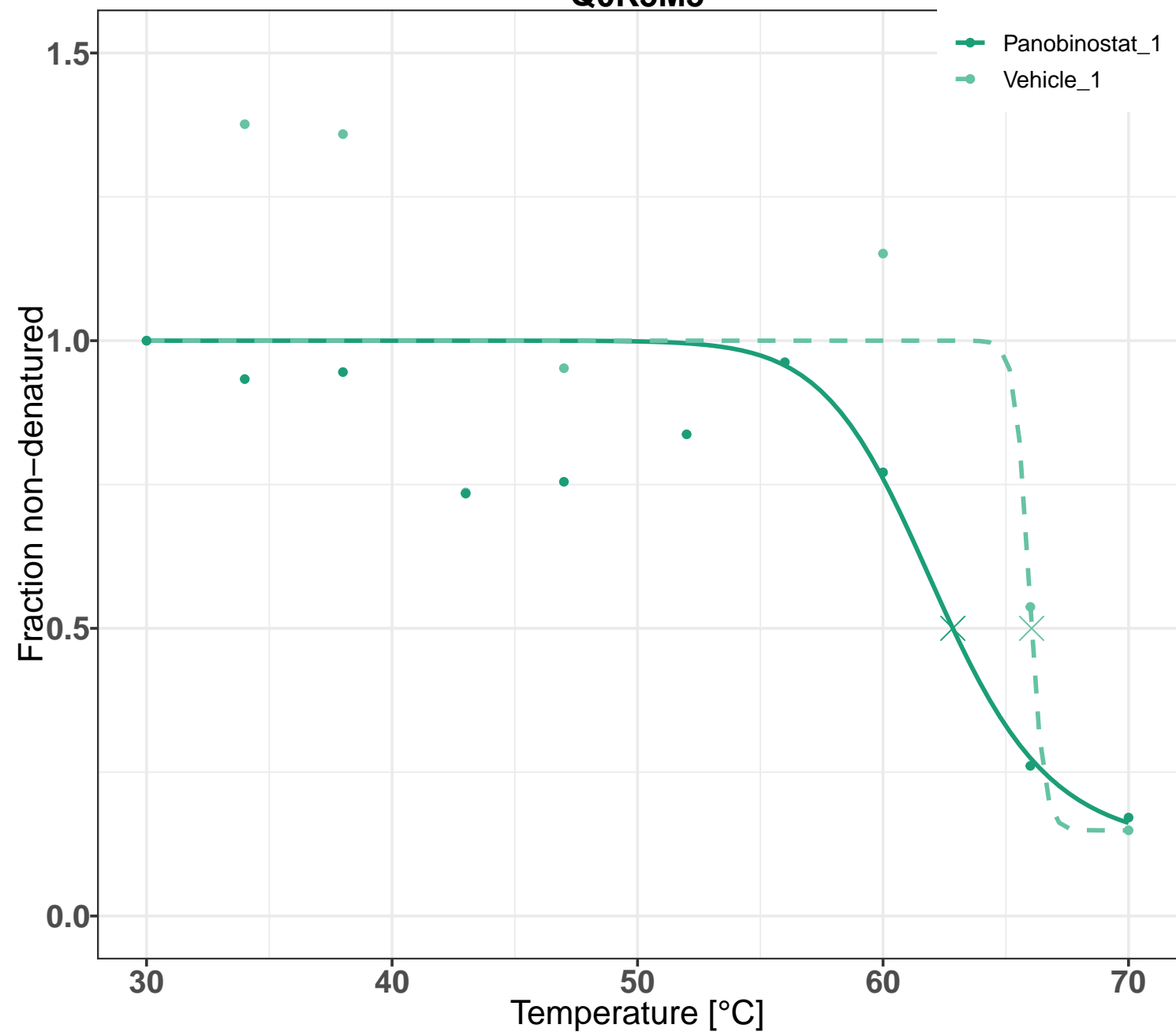

|                | meltPoint | slope  | plateau | R2   |
|----------------|-----------|--------|---------|------|
| Panobinostat_1 | 62.84     | -0.095 | 0.12    | 0.78 |
| Vehicle_1      | 66.05     | -0.73  | 0.15    | 0.44 |

Supplement: Supplementary file 2 — Supplementary Material 2 [file 41598_2026_35990_MOESM2_ESM.zip › AllTheTPPData/D40vD86/Panobinostat_Vignette/Melting_Curves/meltCurve_Q0K5M5.pdf]

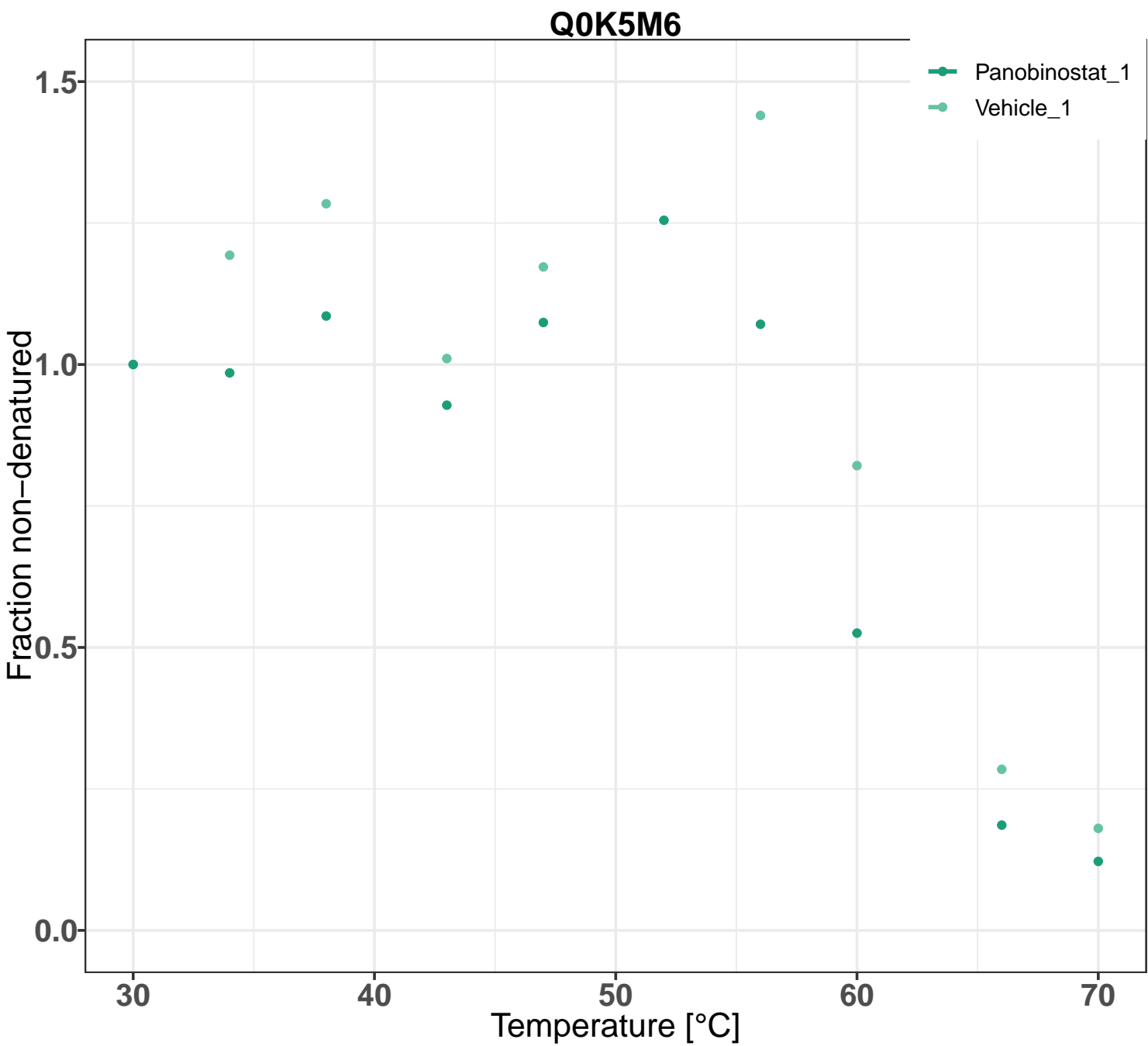

|                | meltPoint | slope | plateau | R2 |
|----------------|-----------|-------|---------|----|
| Panobinostat_1 | —         | —     | —       | —  |
| Vehicle_1      | —         | —     | —       | —  |

Supplement: Supplementary file 2 — Supplementary Material 2 [file 41598_2026_35990_MOESM2_ESM.zip › AllTheTPPData/D40vD86/Panobinostat_Vignette/Melting_Curves/meltCurve_Q0K5M6.pdf]

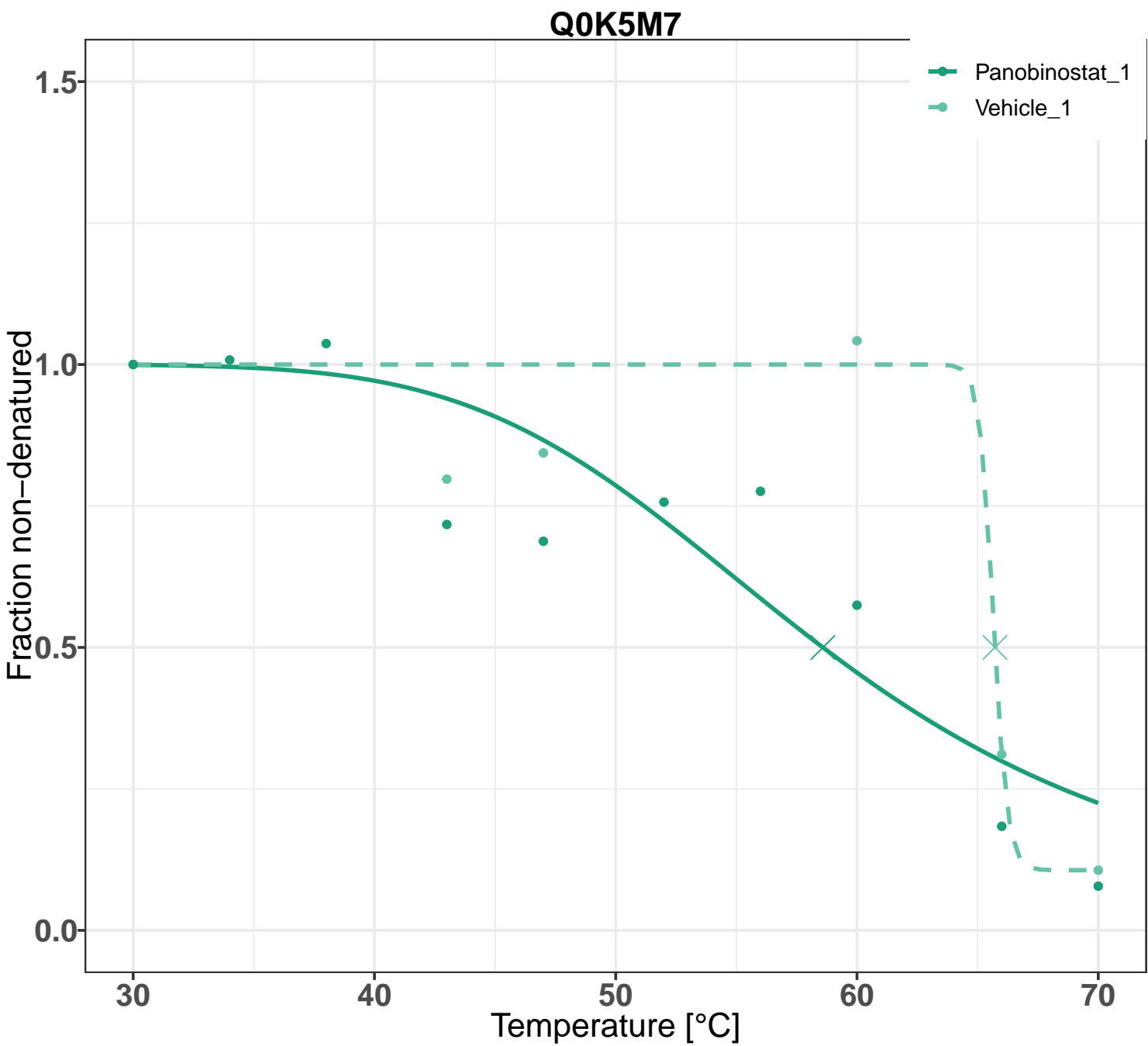

|                | meltPoint | slope  | plateau | R2   |
|----------------|-----------|--------|---------|------|
| Panobinostat_1 | 58.59     | -0.035 | 0       | 0.82 |
| Vehicle_1      | 65.72     | -0.78  | 0.11    | 0.45 |

Supplement: Supplementary file 2 — Supplementary Material 2 [file 41598_2026_35990_MOESM2_ESM.zip › AllTheTPPData/D40vD86/Panobinostat_Vignette/Melting_Curves/meltCurve_Q0K5M7.pdf]

# Q0K5M8

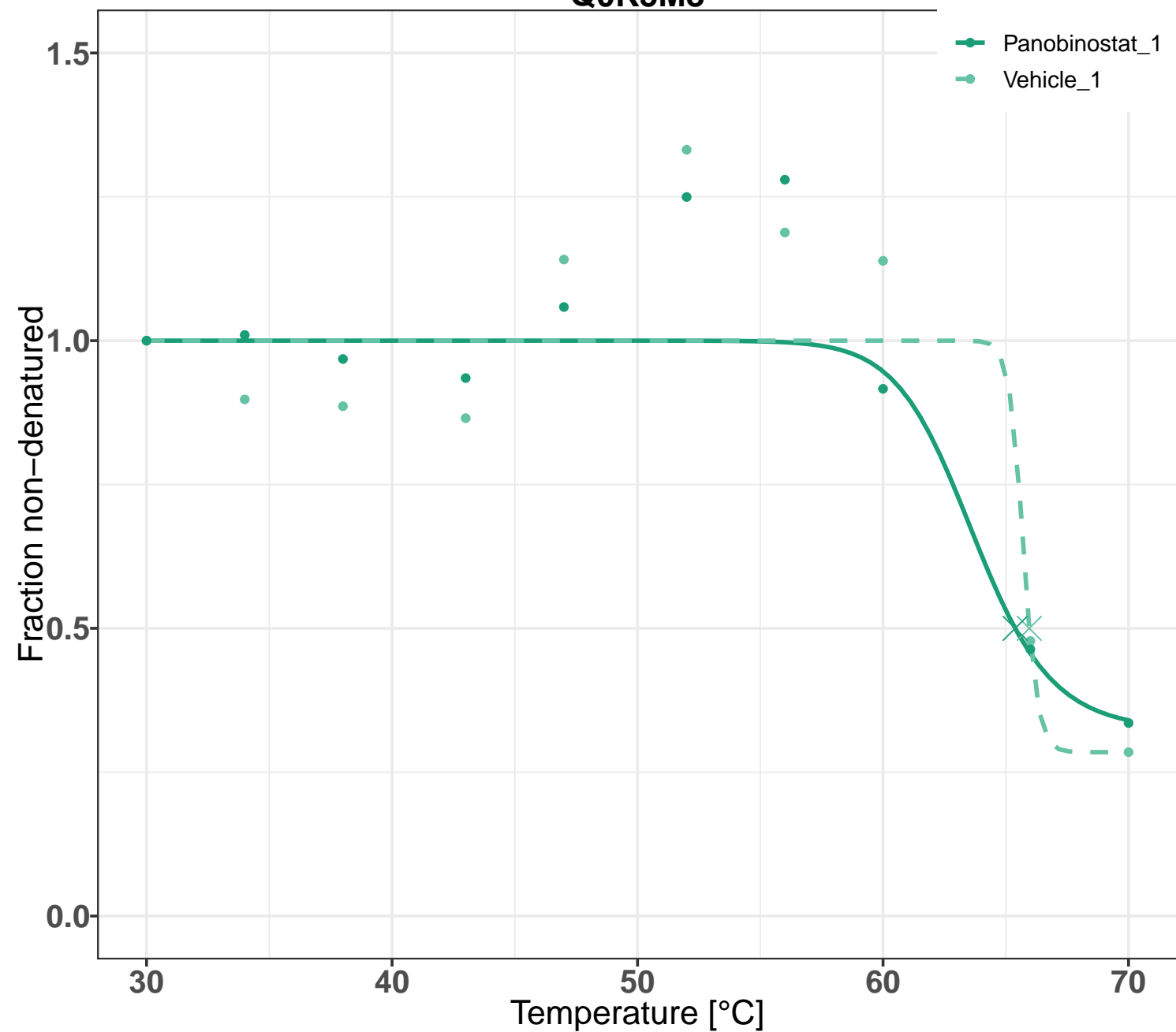

meltPoint

slope

plateau

R2

Panobinostat\_1

65.38

-0.11

0.32

0.82

Vehicle\_1

65.96

-0.62

0.28

0.76

Supplement: Supplementary file 2 — Supplementary Material 2 [file 41598_2026_35990_MOESM2_ESM.zip › AllTheTPPData/D40vD86/Panobinostat_Vignette/Melting_Curves/meltCurve_Q0K5M8.pdf]

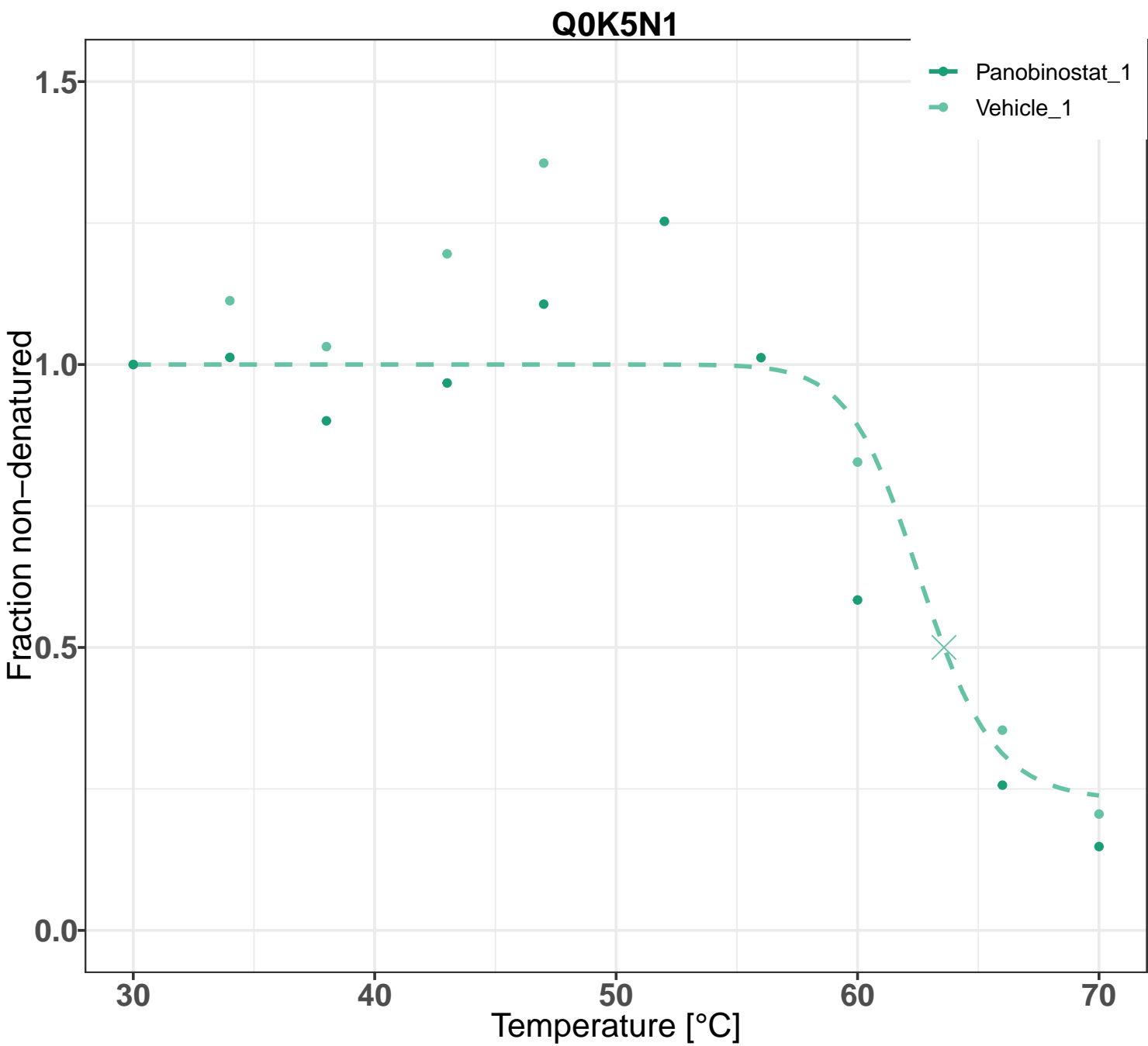

|                | meltPoint | slope | plateau | R2   |
|----------------|-----------|-------|---------|------|
| Panobinostat_1 | –         | –     | –       | –    |
| Vehicle_1      | 63.58     | –0.13 | 0.23    | 0.56 |

Supplement: Supplementary file 2 — Supplementary Material 2 [file 41598_2026_35990_MOESM2_ESM.zip › AllTheTPPData/D40vD86/Panobinostat_Vignette/Melting_Curves/meltCurve_Q0K5N1.pdf]

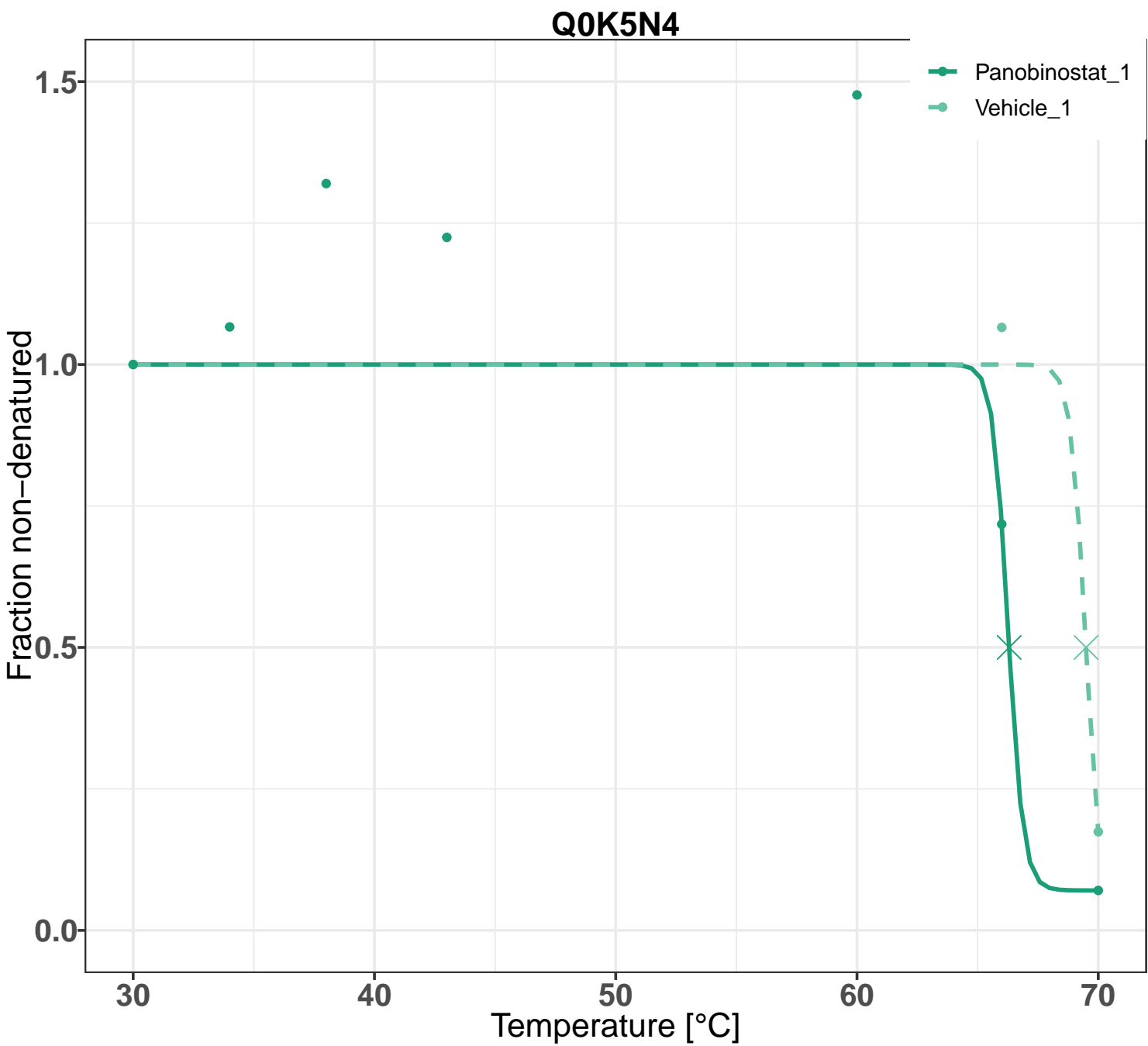

|                | meltPoint | slope | plateau | R2    |
|----------------|-----------|-------|---------|-------|
| Panobinostat_1 | 66.31     | -0.74 | 0.07    | 0.22  |
| Vehicle_1      | 69.5      | -0.78 | 0       | -0.67 |

Supplement: Supplementary file 2 — Supplementary Material 2 [file 41598_2026_35990_MOESM2_ESM.zip › AllTheTPPData/D40vD86/Panobinostat_Vignette/Melting_Curves/meltCurve_Q0K5N4.pdf]

# Q0K5N8

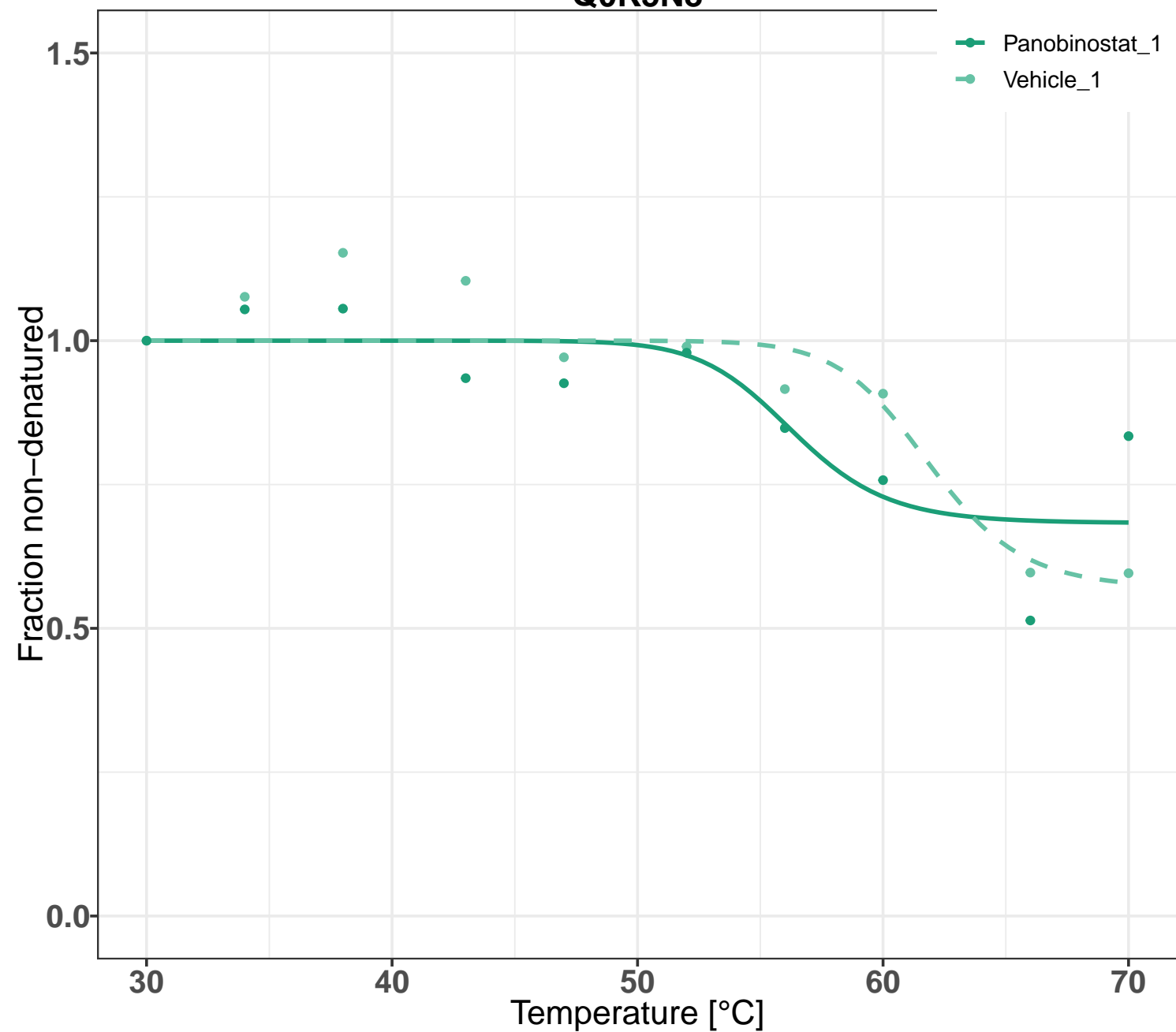

meltPoint

slope

plateau

R2

Panobinostat\_1

—

−0.041

0.68

0.71

Vehicle\_1

—

−0.057

0.57

0.86

Supplement: Supplementary file 2 — Supplementary Material 2 [file 41598_2026_35990_MOESM2_ESM.zip › AllTheTPPData/D40vD86/Panobinostat_Vignette/Melting_Curves/meltCurve_Q0K5N8.pdf]

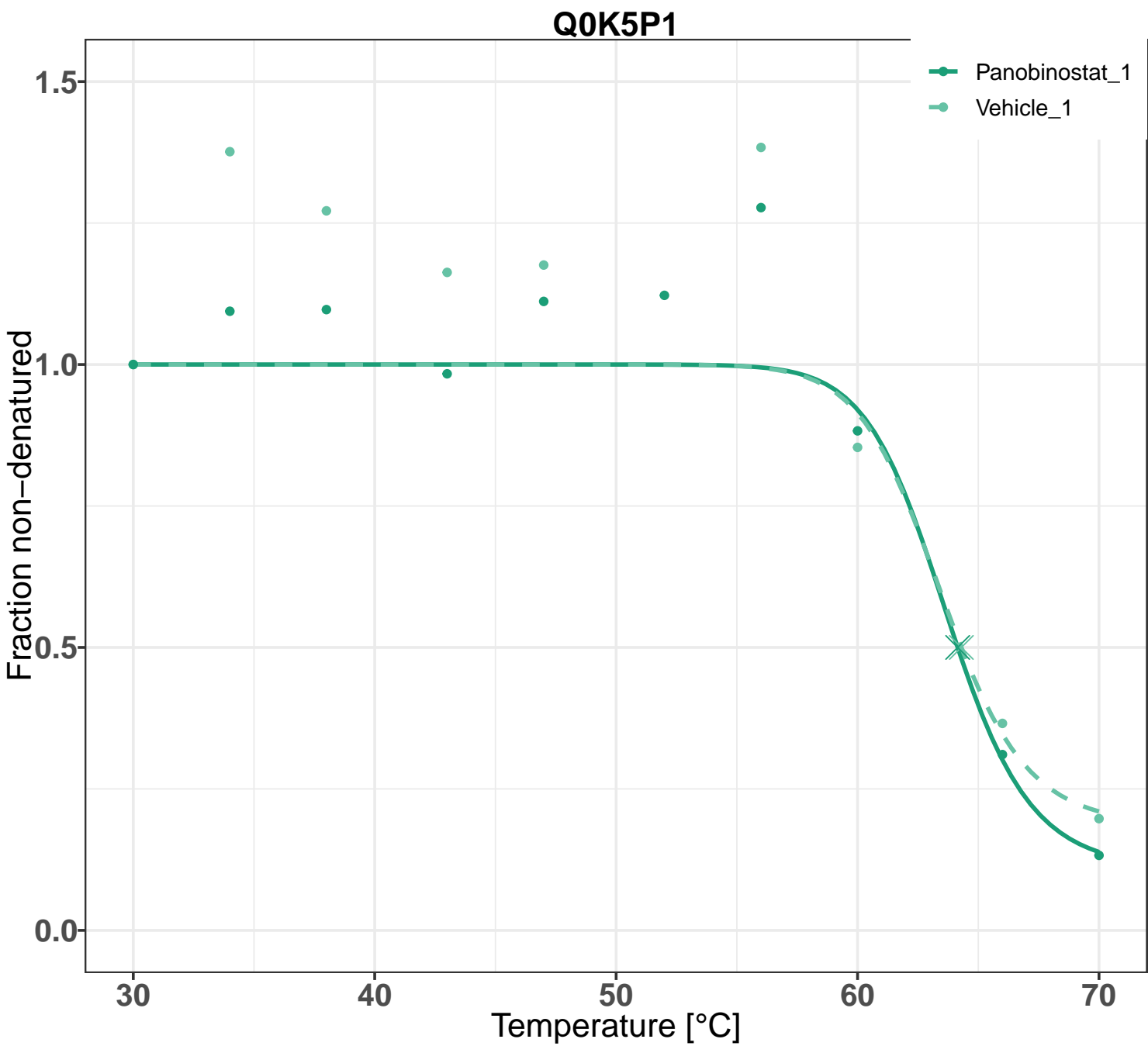

|                | meltPoint | slope | plateau | R2   |
|----------------|-----------|-------|---------|------|
| Panobinostat_1 | 64.15     | -0.13 | 0.11    | 0.9  |
| Vehicle_1      | 64.3      | -0.12 | 0.18    | 0.59 |

Supplement: Supplementary file 2 — Supplementary Material 2 [file 41598_2026_35990_MOESM2_ESM.zip › AllTheTPPData/D40vD86/Panobinostat_Vignette/Melting_Curves/meltCurve_Q0K5P1.pdf]

# Q0K5P3

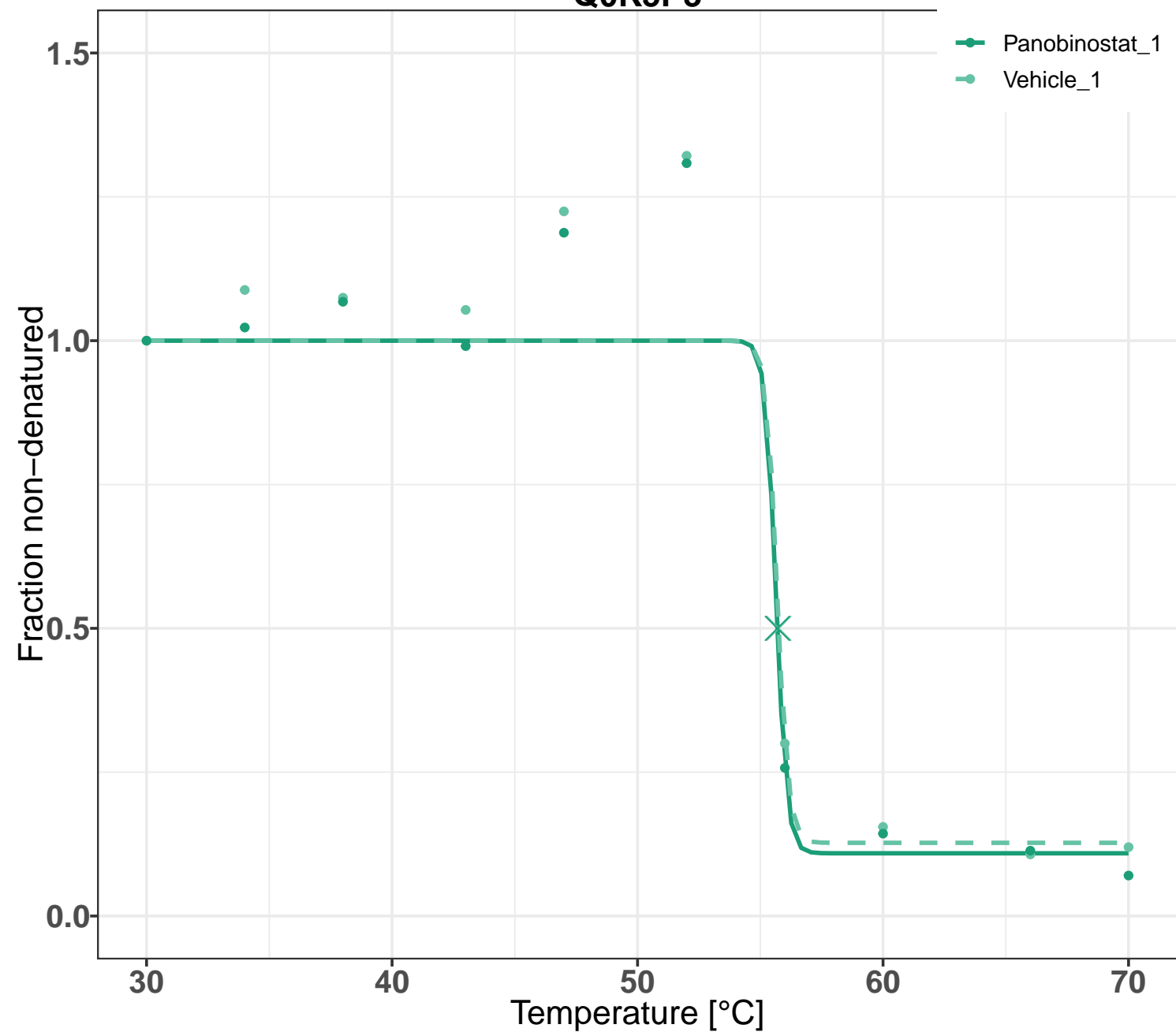

**meltPoint**

**slope**

**plateau**

**R2**

**Panobinostat\_1**

**55.69**

**-1**

**0.11**

**0.94**

**Vehicle\_1**

**55.75**

**-0.98**

**0.13**

**0.93**

Supplement: Supplementary file 2 — Supplementary Material 2 [file 41598_2026_35990_MOESM2_ESM.zip › AllTheTPPData/D40vD86/Panobinostat_Vignette/Melting_Curves/meltCurve_Q0K5P3.pdf]

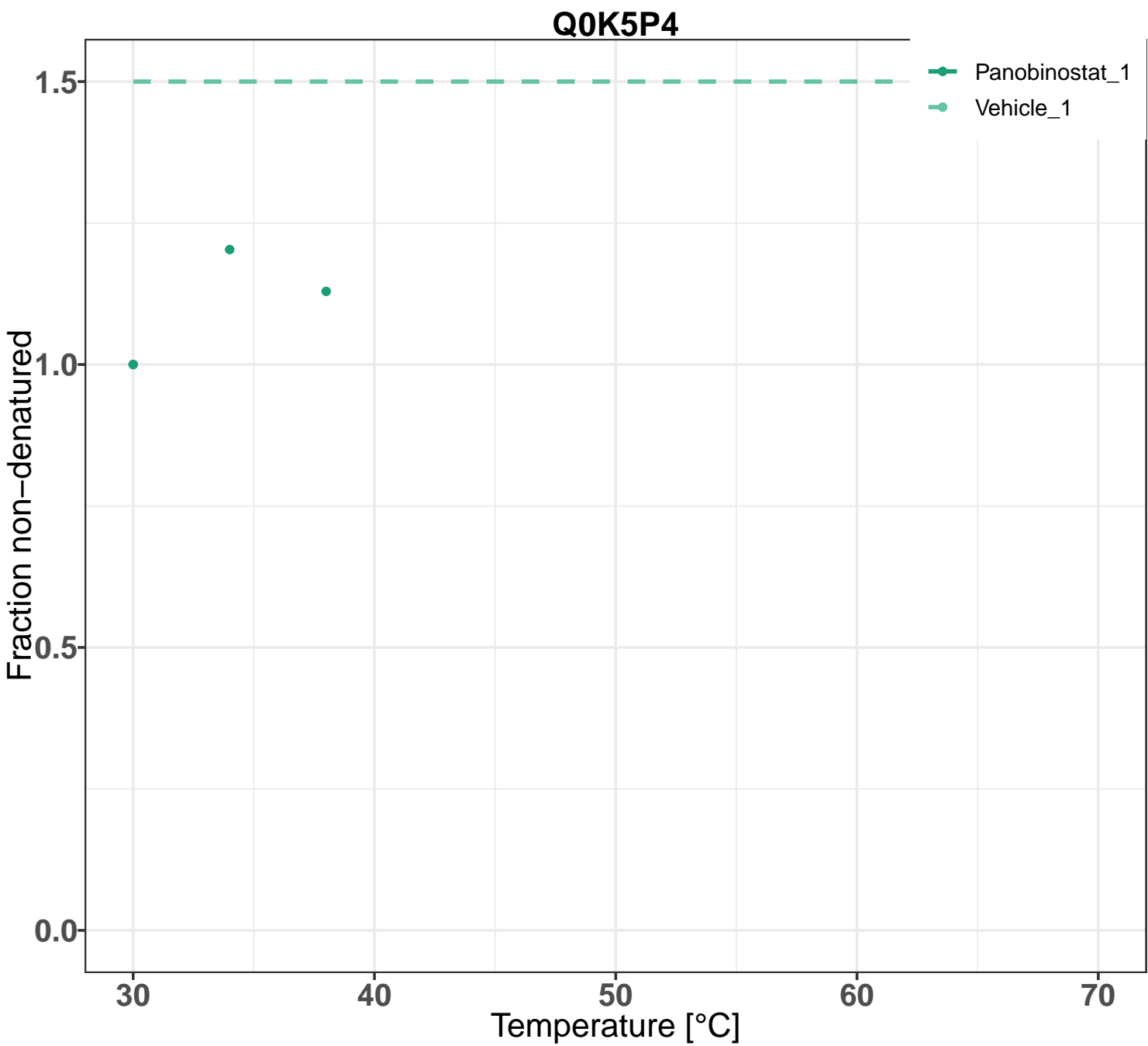

|                | meltPoint | slope | plateau | R2    |
|----------------|-----------|-------|---------|-------|
| Panobinostat_1 | –         | –     | –       | –     |
| Vehicle_1      | –         | –     | 1.5     | –1.02 |

Supplement: Supplementary file 2 — Supplementary Material 2 [file 41598_2026_35990_MOESM2_ESM.zip › AllTheTPPData/D40vD86/Panobinostat_Vignette/Melting_Curves/meltCurve_Q0K5P4.pdf]

# Q0K5P5

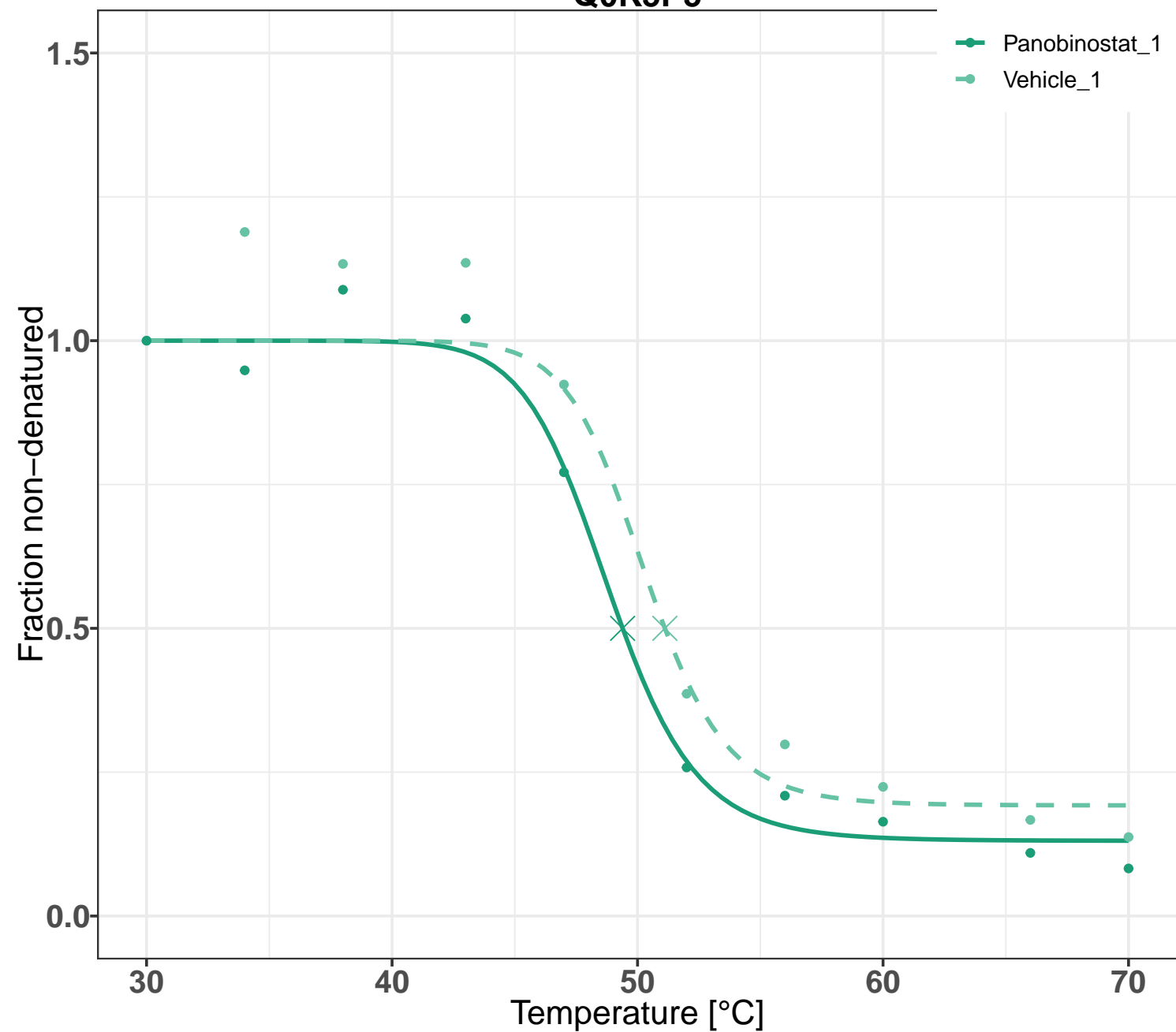

**meltPoint**

**slope**

**plateau**

**R2**

**Panobinostat\_1**

**49.39**

**-0.12**

**0.13**

**0.99**

**Vehicle\_1**

**51.11**

**-0.12**

**0.19**

**0.95**

Supplement: Supplementary file 2 — Supplementary Material 2 [file 41598_2026_35990_MOESM2_ESM.zip › AllTheTPPData/D40vD86/Panobinostat_Vignette/Melting_Curves/meltCurve_Q0K5P5.pdf]

# Q0K5R9

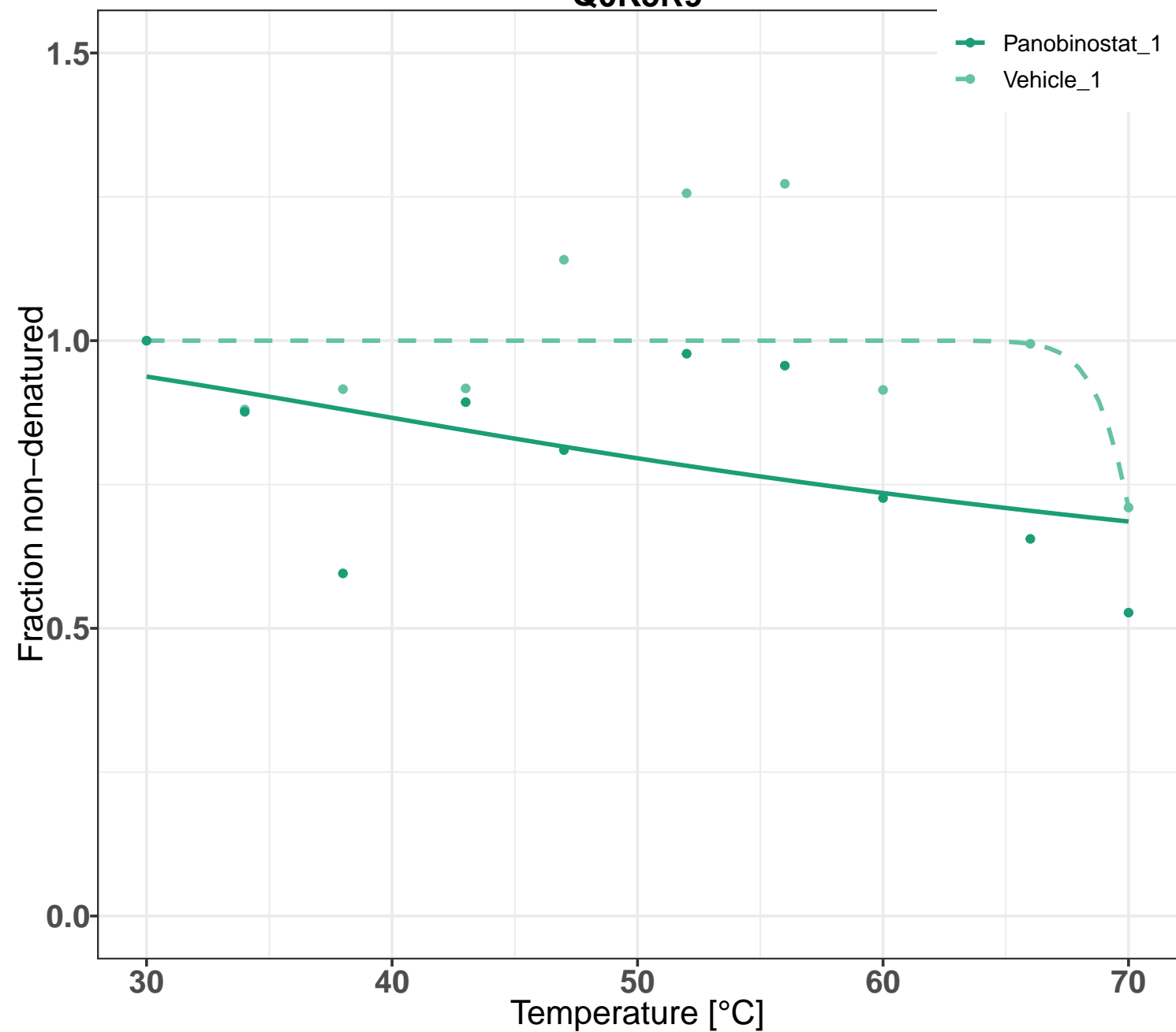

|                | meltPoint | slope   | plateau | R2   |
|----------------|-----------|---------|---------|------|
| Panobinostat_1 | –         | –0.0074 | 0       | 0.23 |
| Vehicle_1      | –         | –       | 0       | 0.3  |

Supplement: Supplementary file 2 — Supplementary Material 2 [file 41598_2026_35990_MOESM2_ESM.zip › AllTheTPPData/D40vD86/Panobinostat_Vignette/Melting_Curves/meltCurve_Q0K5R9.pdf]

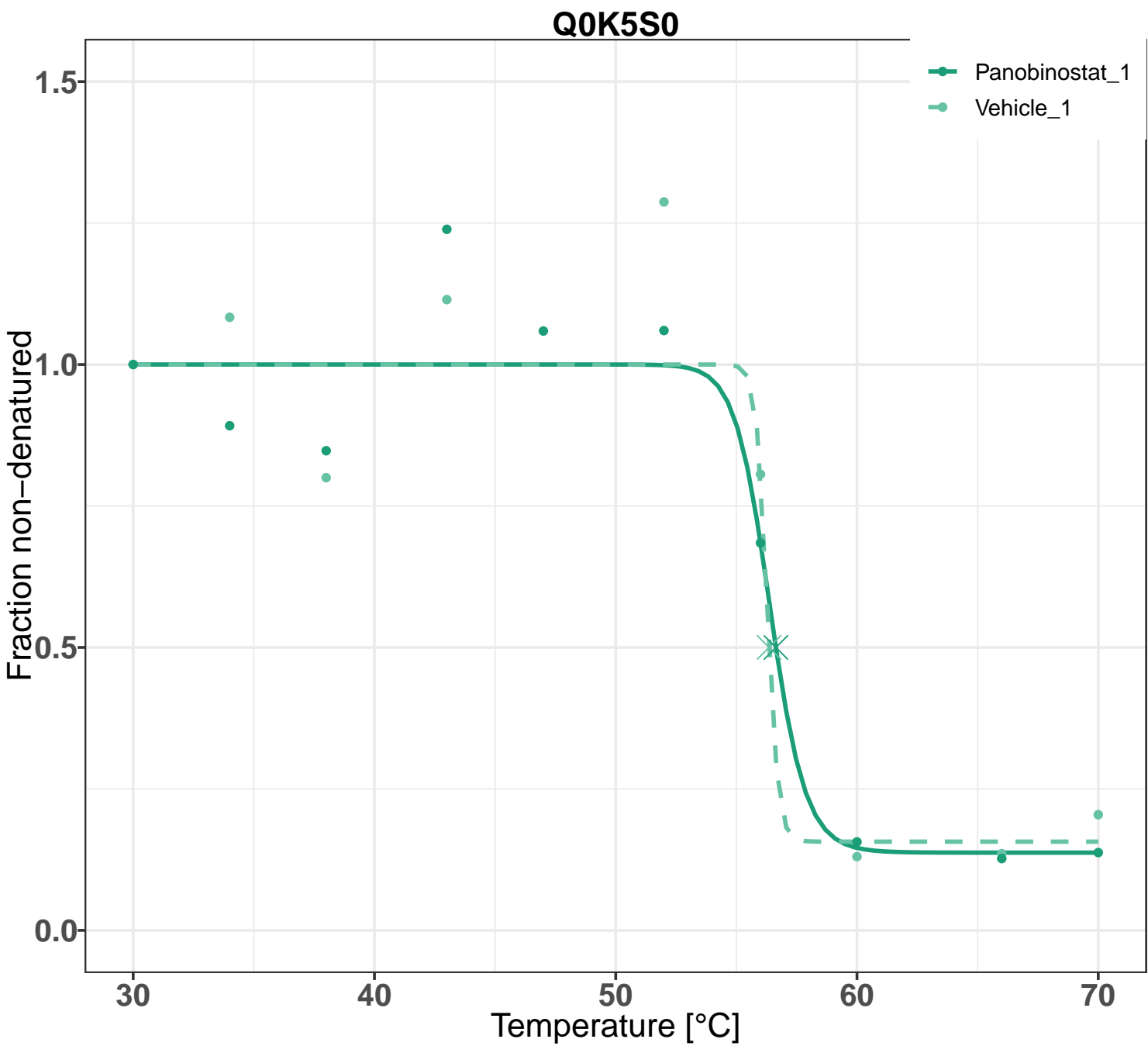

|                | meltPoint | slope | plateau | R2   |
|----------------|-----------|-------|---------|------|
| Panobinostat_1 | 56.64     | -0.3  | 0.14    | 0.94 |
| Vehicle_1      | 56.36     | -0.94 | 0.16    | 0.81 |

Supplement: Supplementary file 2 — Supplementary Material 2 [file 41598_2026_35990_MOESM2_ESM.zip › AllTheTPPData/D40vD86/Panobinostat_Vignette/Melting_Curves/meltCurve_Q0K5S0.pdf]

# Q0K5S4

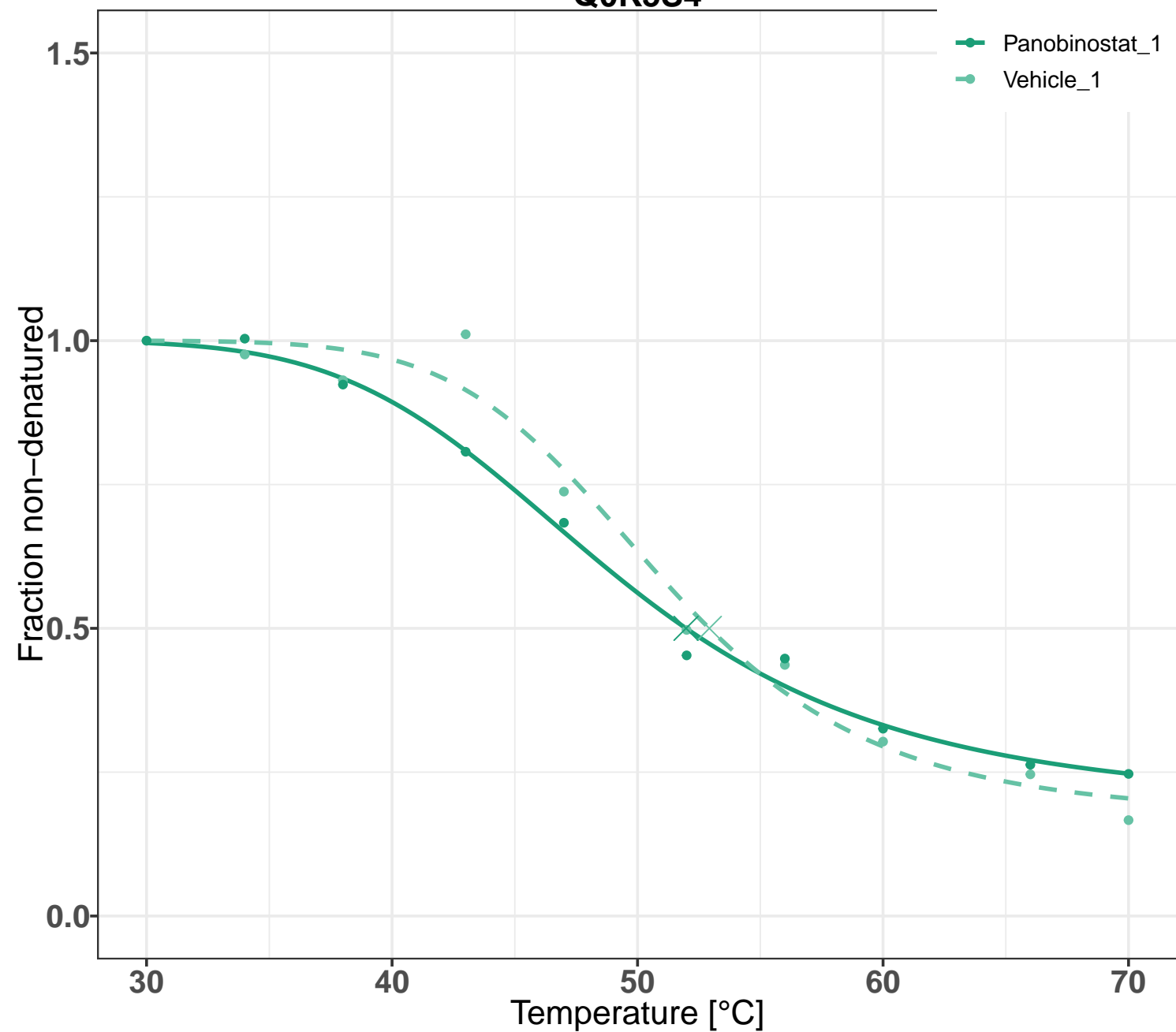

meltPoint

slope

plateau

R2

Panobinostat\_1

51.97

-0.037

0.18

0.99

Vehicle\_1

52.92

-0.048

0.17

0.98

Supplement: Supplementary file 2 — Supplementary Material 2 [file 41598_2026_35990_MOESM2_ESM.zip › AllTheTPPData/D40vD86/Panobinostat_Vignette/Melting_Curves/meltCurve_Q0K5S4.pdf]

# Q0K5S5

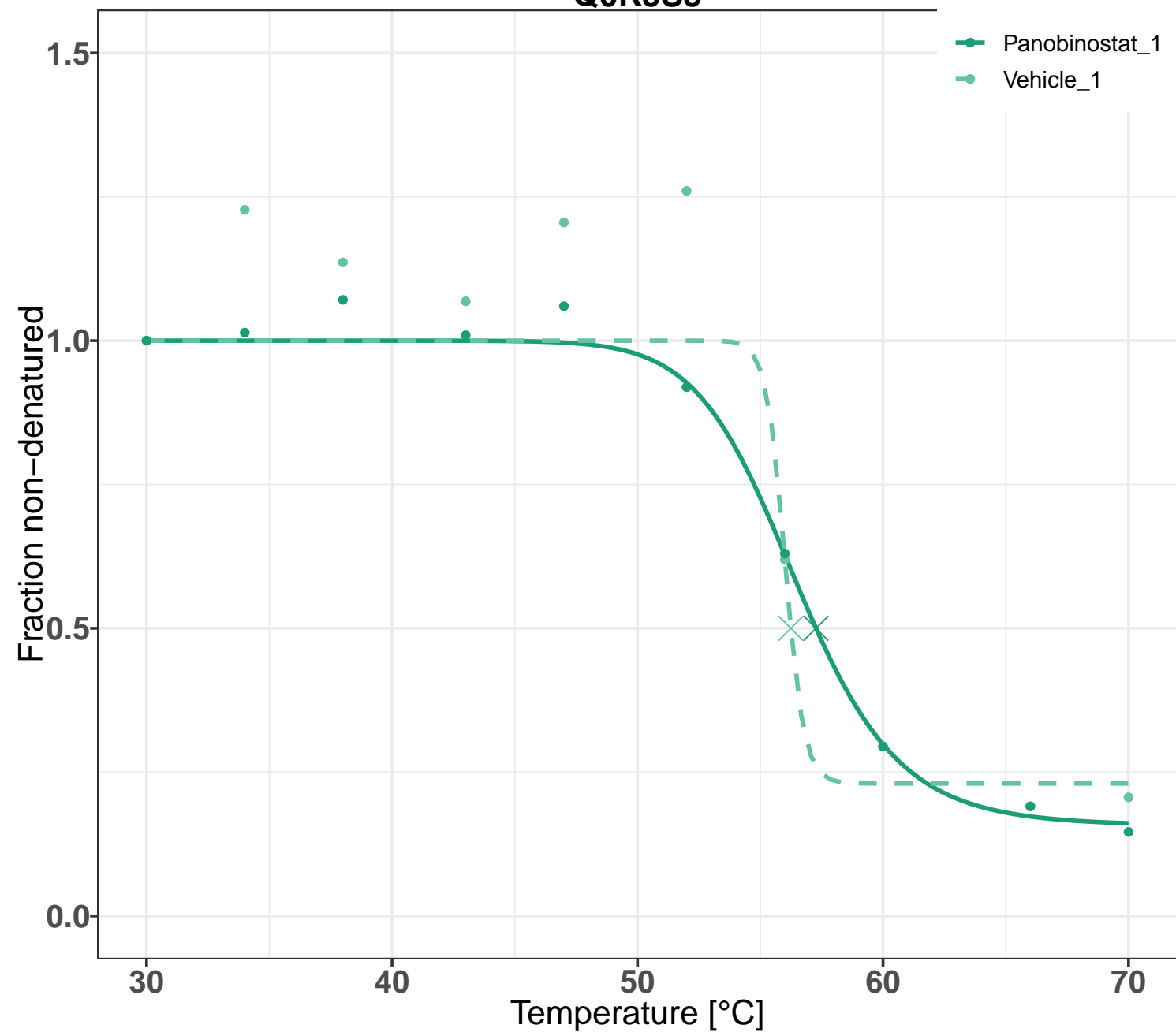

meltPoint

slope

plateau

R2

Panobinostat\_1

57.27

-0.1

0.16

0.99

Vehicle\_1

56.24

-0.5

0.23

0.89

Supplement: Supplementary file 2 — Supplementary Material 2 [file 41598_2026_35990_MOESM2_ESM.zip › AllTheTPPData/D40vD86/Panobinostat_Vignette/Melting_Curves/meltCurve_Q0K5S5.pdf]

# Q0K5S8

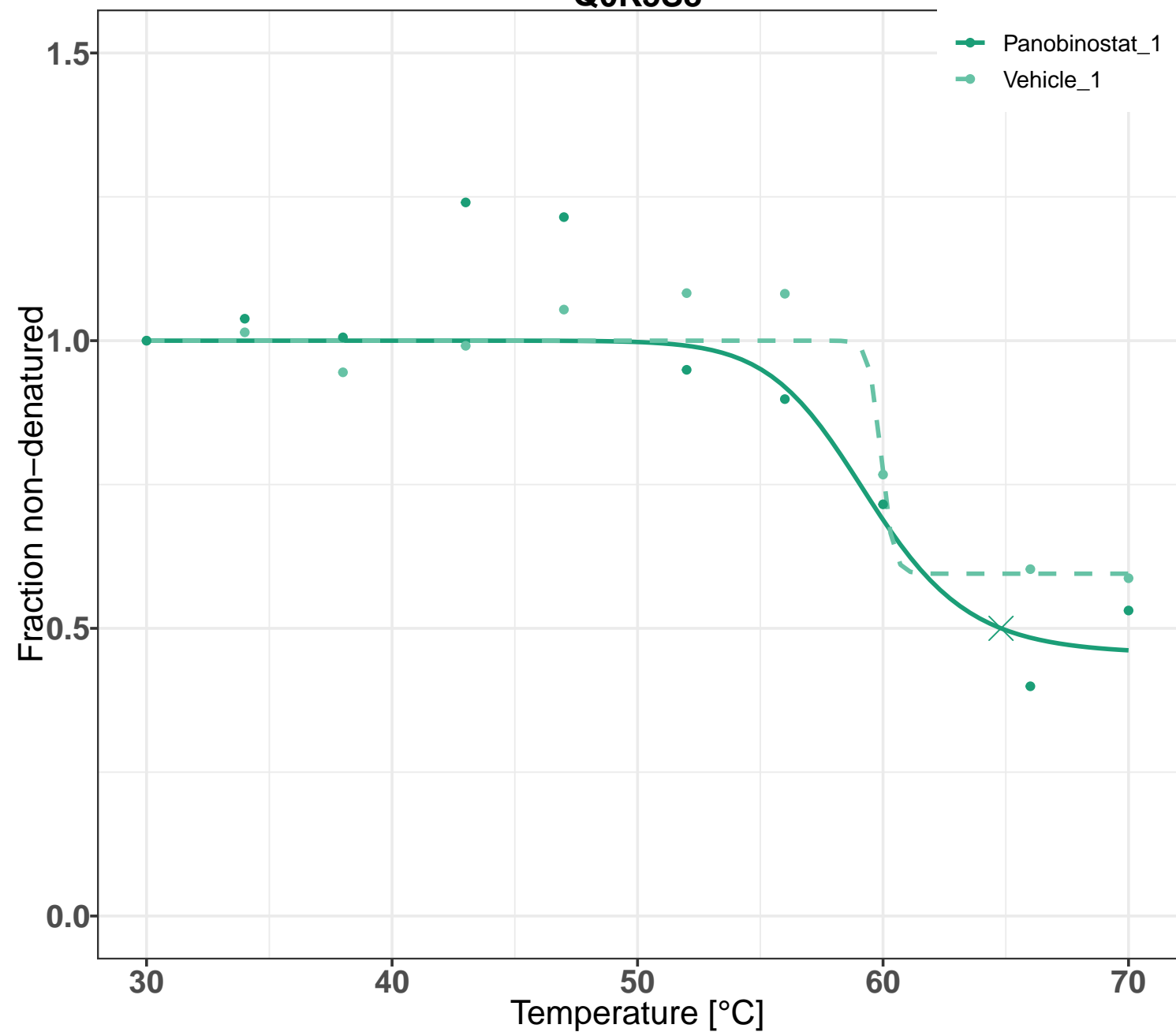

meltPoint

slope

plateau

R2

Panobinostat\_1

64.8

-0.067

0.45

0.82

Vehicle\_1

-

-0.42

0.59

0.94

Supplement: Supplementary file 2 — Supplementary Material 2 [file 41598_2026_35990_MOESM2_ESM.zip › AllTheTPPData/D40vD86/Panobinostat_Vignette/Melting_Curves/meltCurve_Q0K5S8.pdf]

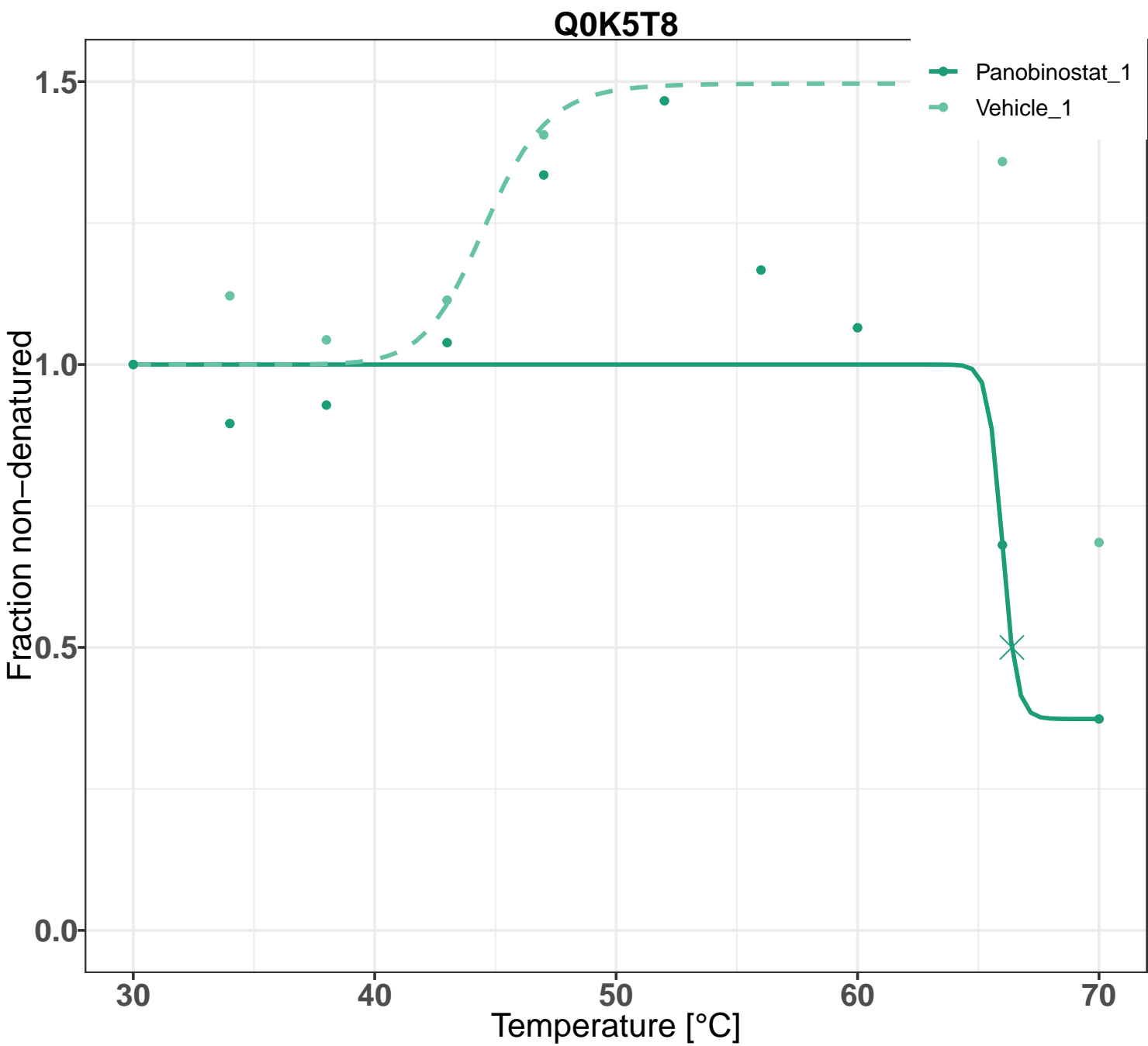

|                | meltPoint | slope | plateau | R2   |
|----------------|-----------|-------|---------|------|
| Panobinostat_1 | 66.39     | -0.54 | 0.37    | 0.57 |
| Vehicle_1      | -         | 0.096 | 1.5     | 0.27 |

Supplement: Supplementary file 2 — Supplementary Material 2 [file 41598_2026_35990_MOESM2_ESM.zip › AllTheTPPData/D40vD86/Panobinostat_Vignette/Melting_Curves/meltCurve_Q0K5T8.pdf]

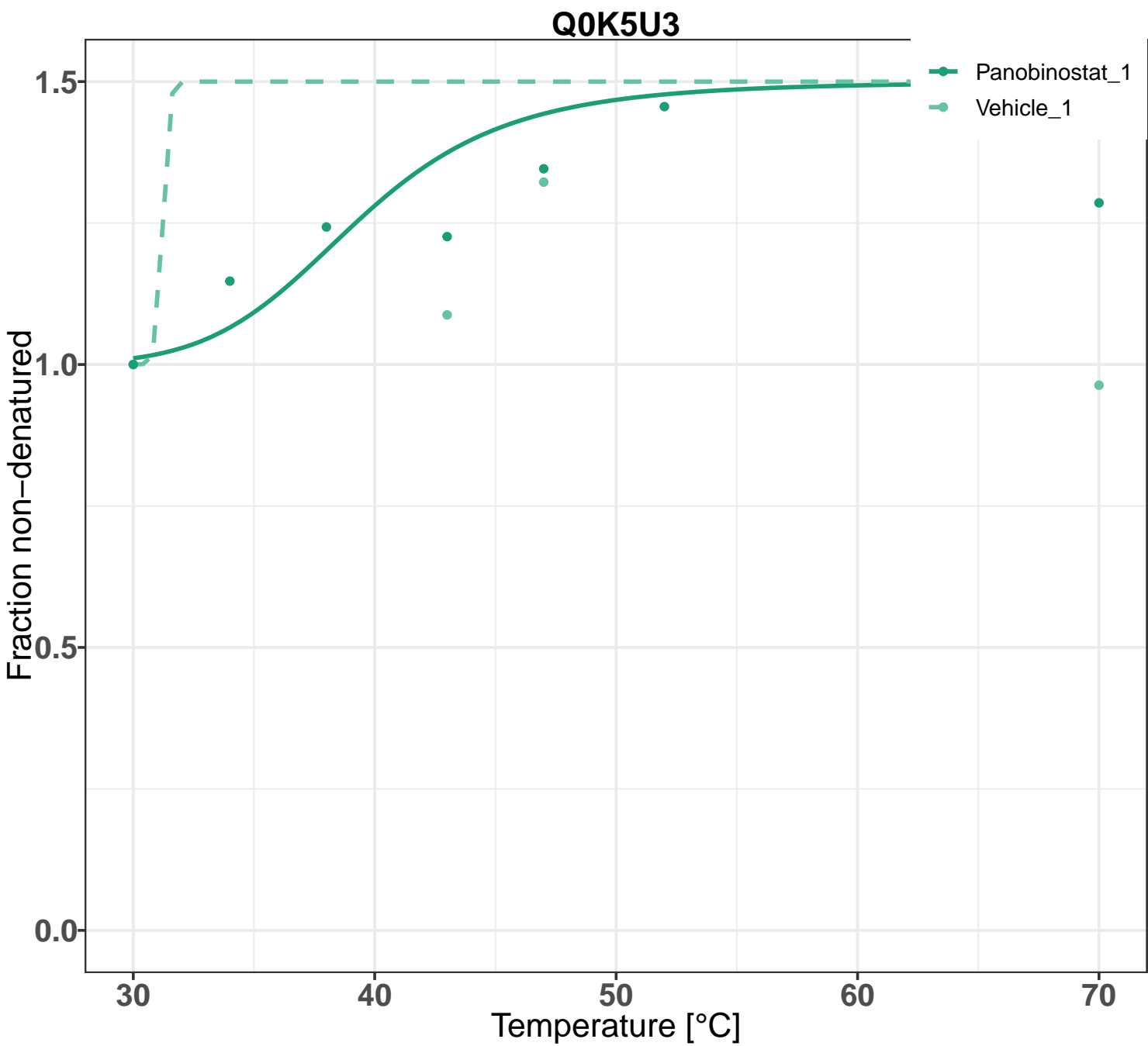

|                | meltPoint | slope | plateau | R2    |
|----------------|-----------|-------|---------|-------|
| Panobinostat_1 | —         | 0.04  | 1.5     | 0.39  |
| Vehicle_1      | —         | 1     | 1.5     | -0.12 |

Supplement: Supplementary file 2 — Supplementary Material 2 [file 41598_2026_35990_MOESM2_ESM.zip › AllTheTPPData/D40vD86/Panobinostat_Vignette/Melting_Curves/meltCurve_Q0K5U3.pdf]

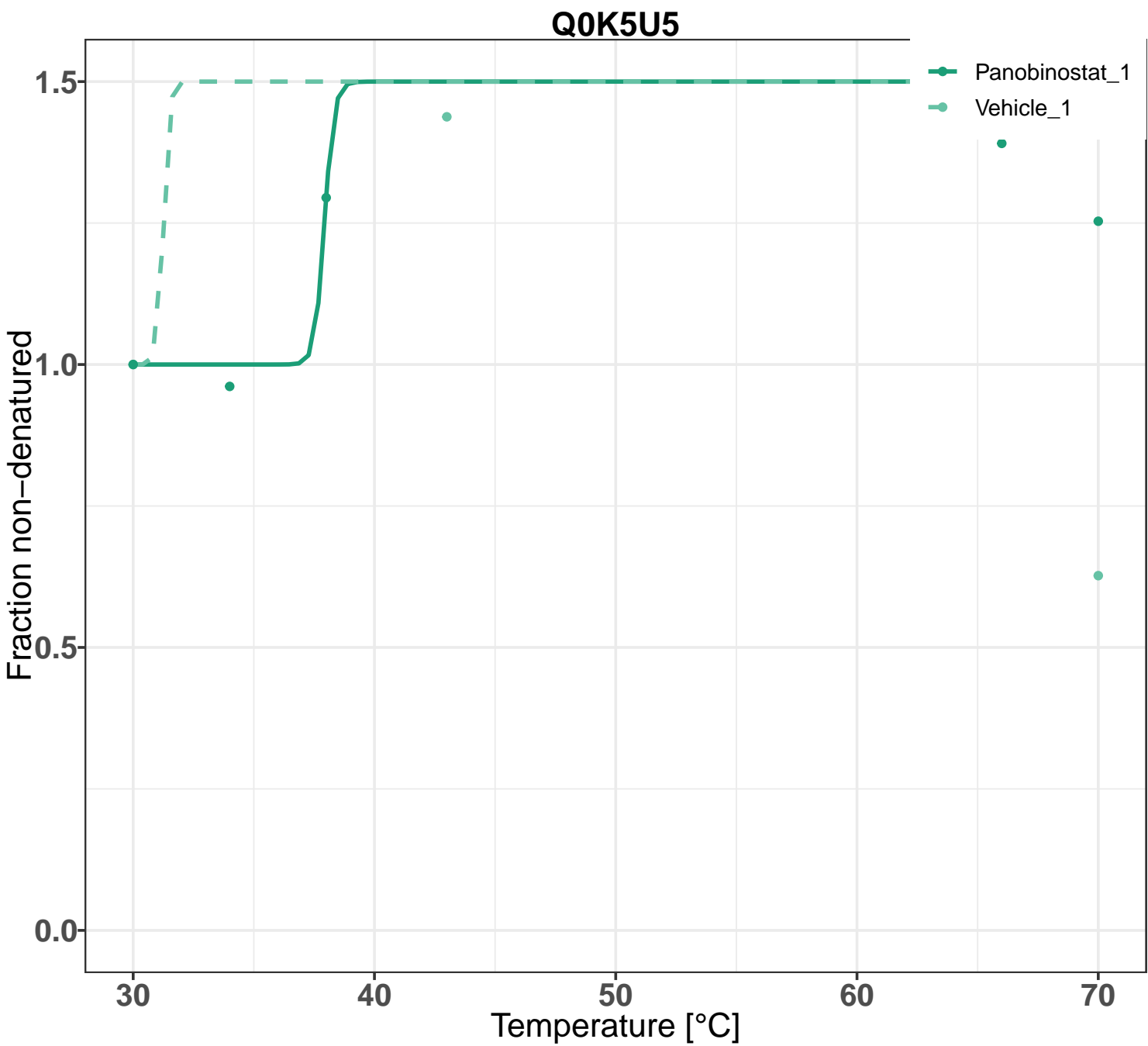

|                | meltPoint | slope | plateau | R2    |
|----------------|-----------|-------|---------|-------|
| Panobinostat_1 | –         | 0.63  | 1.5     | 0.27  |
| Vehicle_1      | –         | 1     | 1.5     | –0.31 |

Supplement: Supplementary file 2 — Supplementary Material 2 [file 41598_2026_35990_MOESM2_ESM.zip › AllTheTPPData/D40vD86/Panobinostat_Vignette/Melting_Curves/meltCurve_Q0K5U5.pdf]

# Q0K5U7

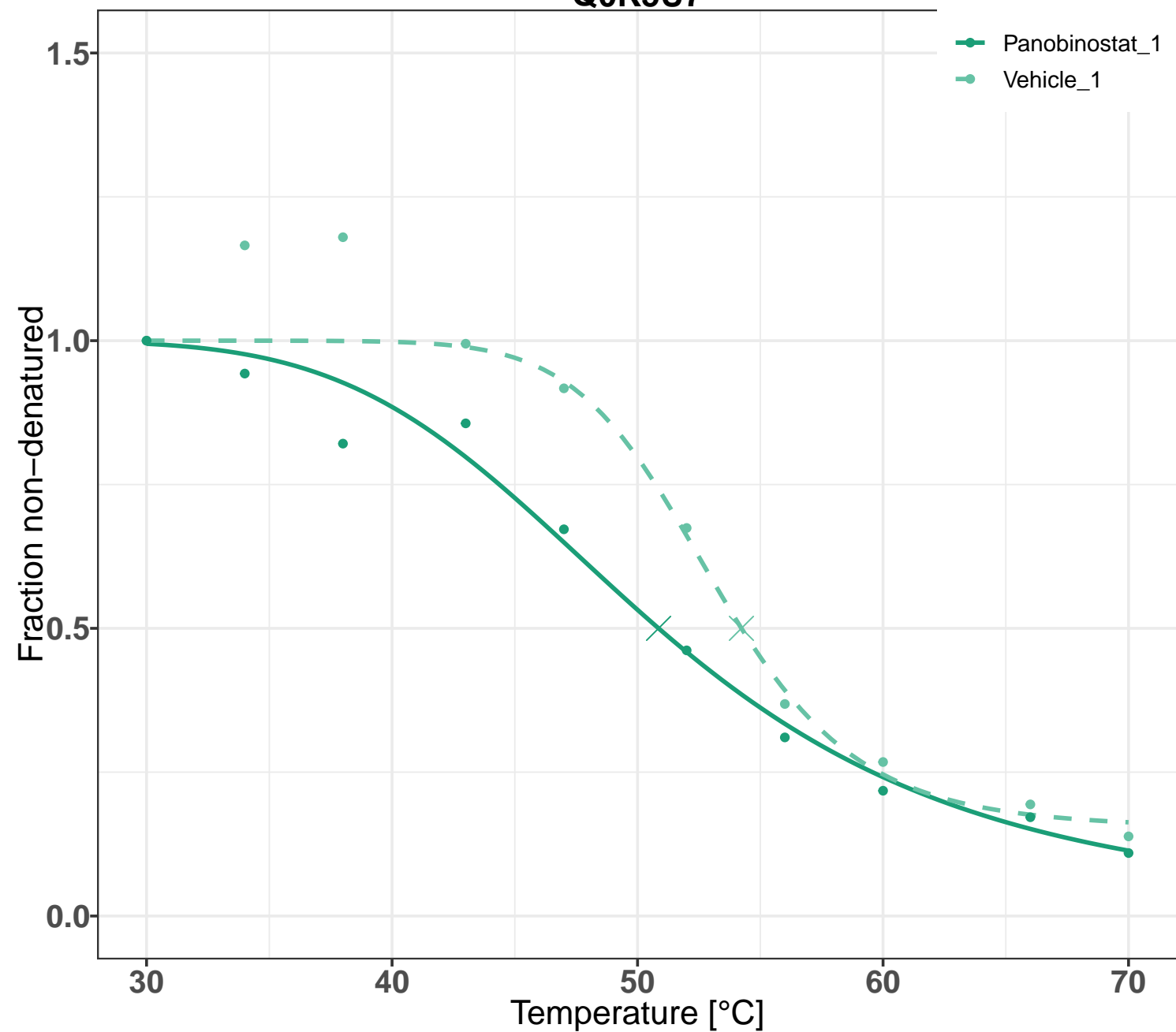

|                | meltPoint | slope  | plateau | R2   |
|----------------|-----------|--------|---------|------|
| Panobinostat_1 | 50.86     | -0.039 | 0       | 0.98 |
| Vehicle_1      | 54.23     | -0.074 | 0.15    | 0.96 |

Supplement: Supplementary file 2 — Supplementary Material 2 [file 41598_2026_35990_MOESM2_ESM.zip › AllTheTPPData/D40vD86/Panobinostat_Vignette/Melting_Curves/meltCurve_Q0K5U7.pdf]

# Q0K5V0

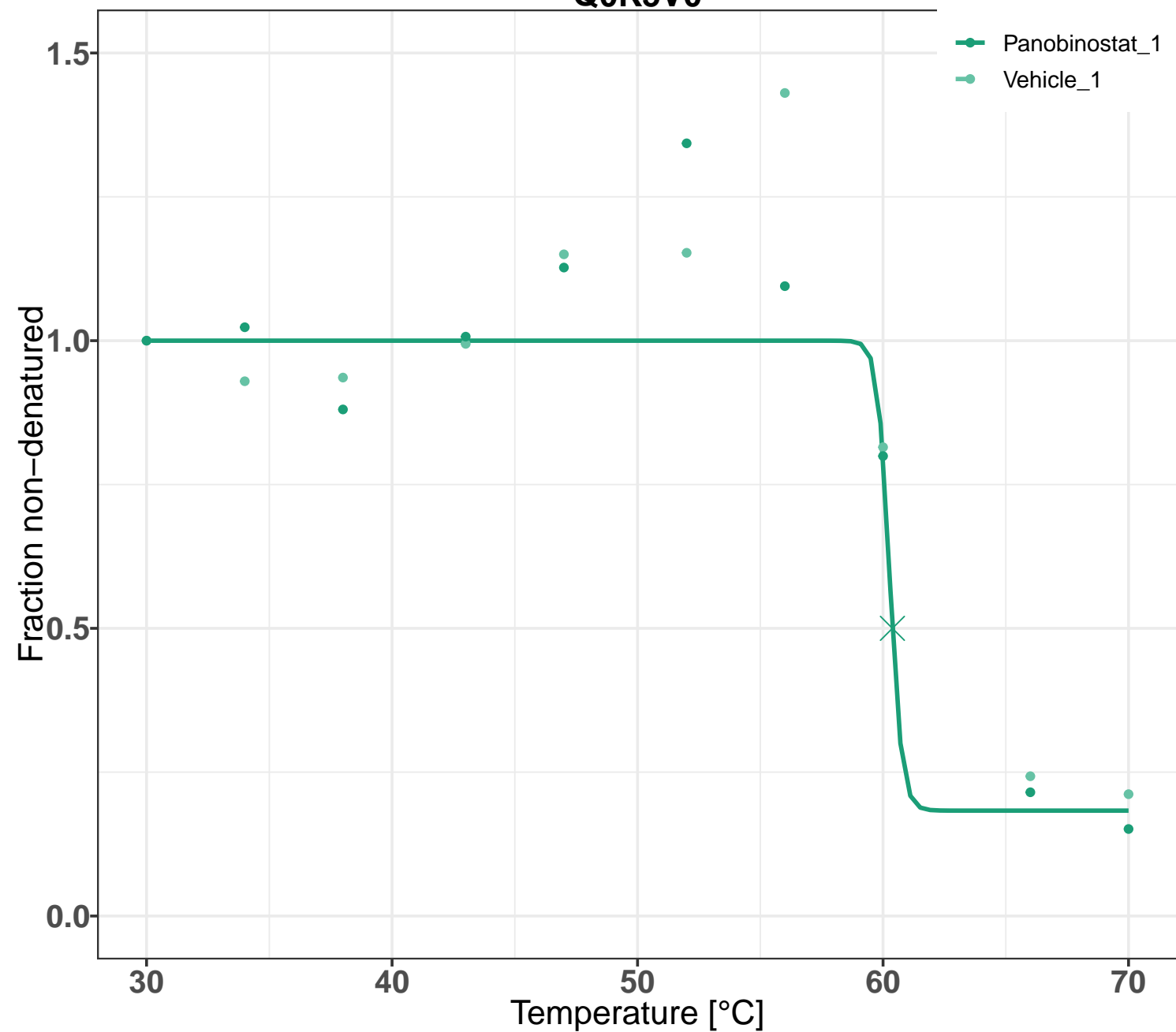

meltPoint

slope

plateau

R2

Panobinostat\_1

60.38

-0.84

0.18

0.88

Vehicle\_1

-

-

-

-

Supplement: Supplementary file 2 — Supplementary Material 2 [file 41598_2026_35990_MOESM2_ESM.zip › AllTheTPPData/D40vD86/Panobinostat_Vignette/Melting_Curves/meltCurve_Q0K5V0.pdf]

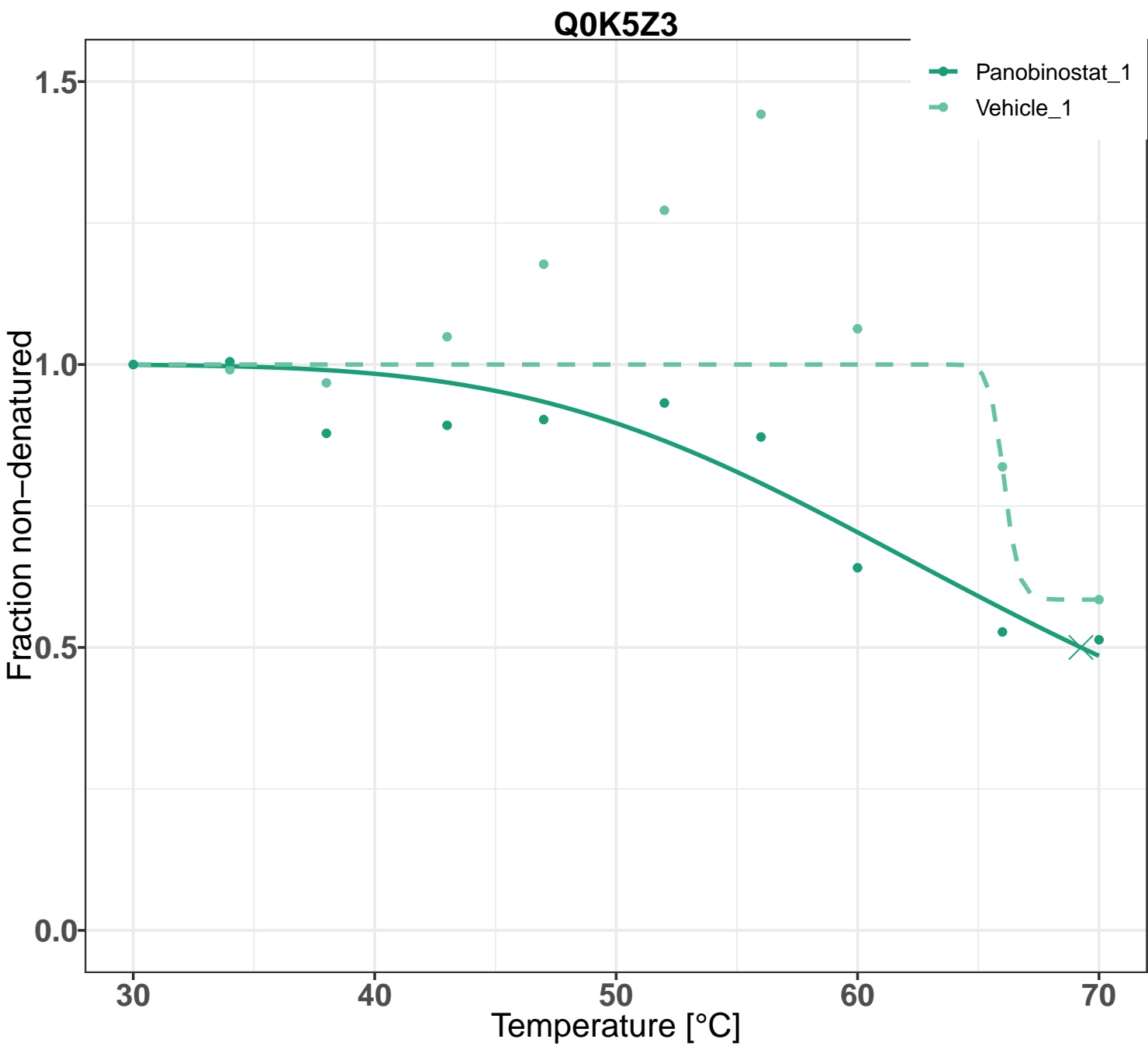

|                | meltPoint | slope  | plateau | R2   |
|----------------|-----------|--------|---------|------|
| Panobinostat_1 | 69.25     | -0.023 | 0       | 0.88 |
| Vehicle_1      | -         | -0.36  | 0.58    | 0.38 |

Supplement: Supplementary file 2 — Supplementary Material 2 [file 41598_2026_35990_MOESM2_ESM.zip › AllTheTPPData/D40vD86/Panobinostat_Vignette/Melting_Curves/meltCurve_Q0K5Z3.pdf]

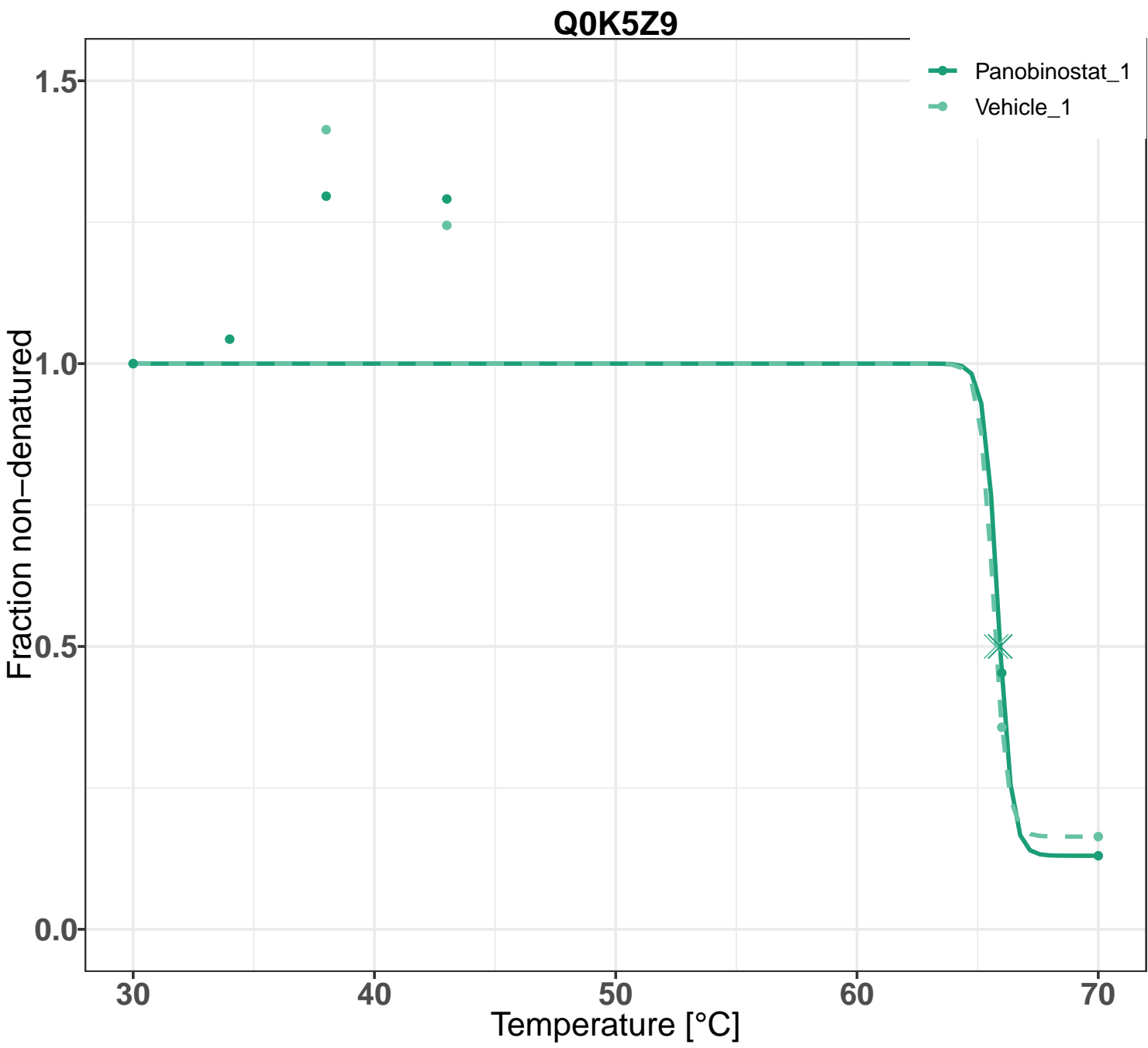

|                | meltPoint | slope | plateau | R2   |
|----------------|-----------|-------|---------|------|
| Panobinostat_1 | 65.93     | -0.75 | 0.13    | 0.14 |
| Vehicle_1      | 65.77     | -0.73 | 0.16    | -0.2 |

Supplement: Supplementary file 2 — Supplementary Material 2 [file 41598_2026_35990_MOESM2_ESM.zip › AllTheTPPData/D40vD86/Panobinostat_Vignette/Melting_Curves/meltCurve_Q0K5Z9.pdf]

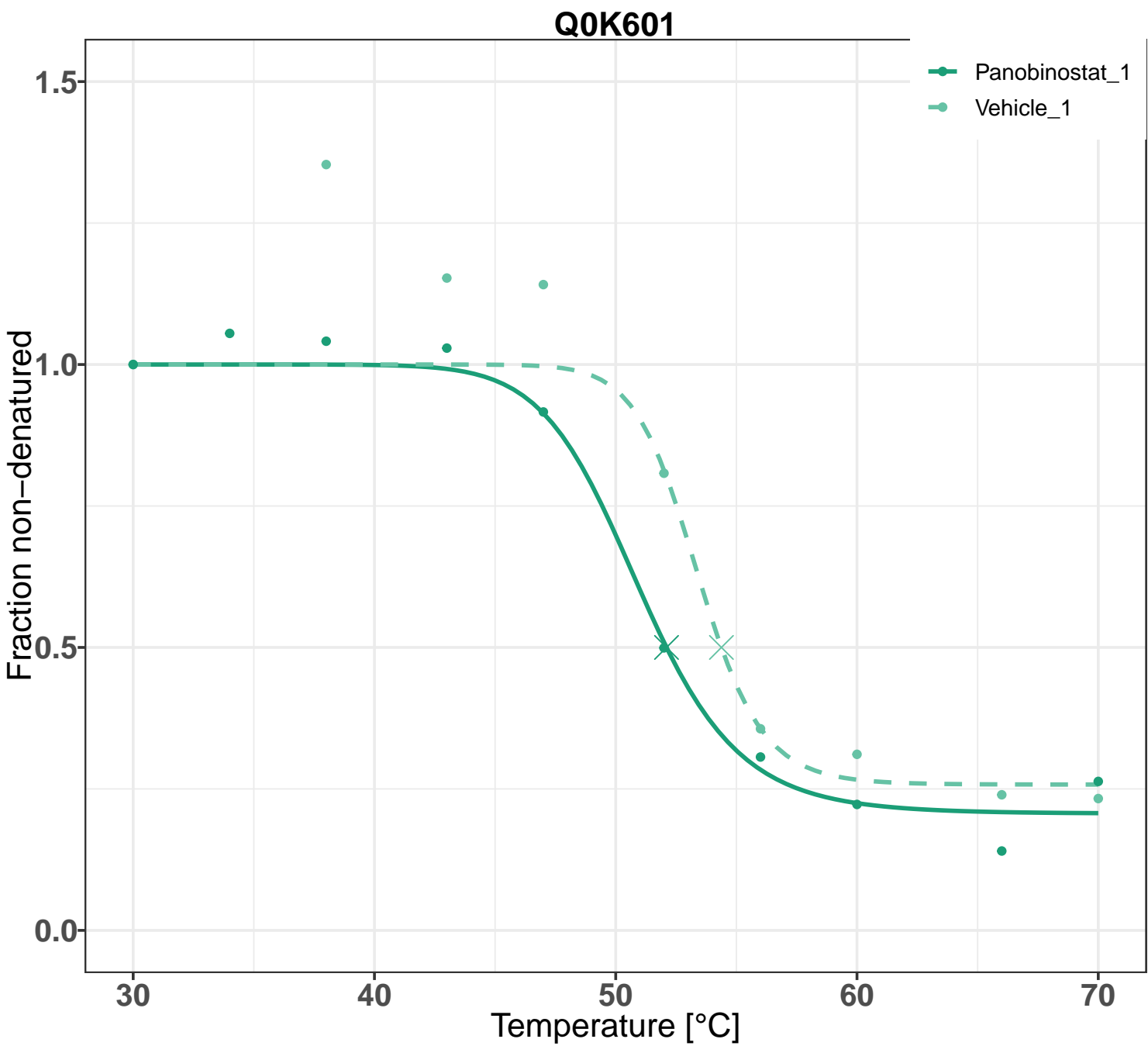

|                | meltPoint | slope  | plateau | R2   |
|----------------|-----------|--------|---------|------|
| Panobinostat_1 | 52.1      | -0.097 | 0.21    | 0.99 |
| Vehicle_1      | 54.38     | -0.14  | 0.26    | 0.8  |

Supplement: Supplementary file 2 — Supplementary Material 2 [file 41598_2026_35990_MOESM2_ESM.zip › AllTheTPPData/D40vD86/Panobinostat_Vignette/Melting_Curves/meltCurve_Q0K601.pdf]

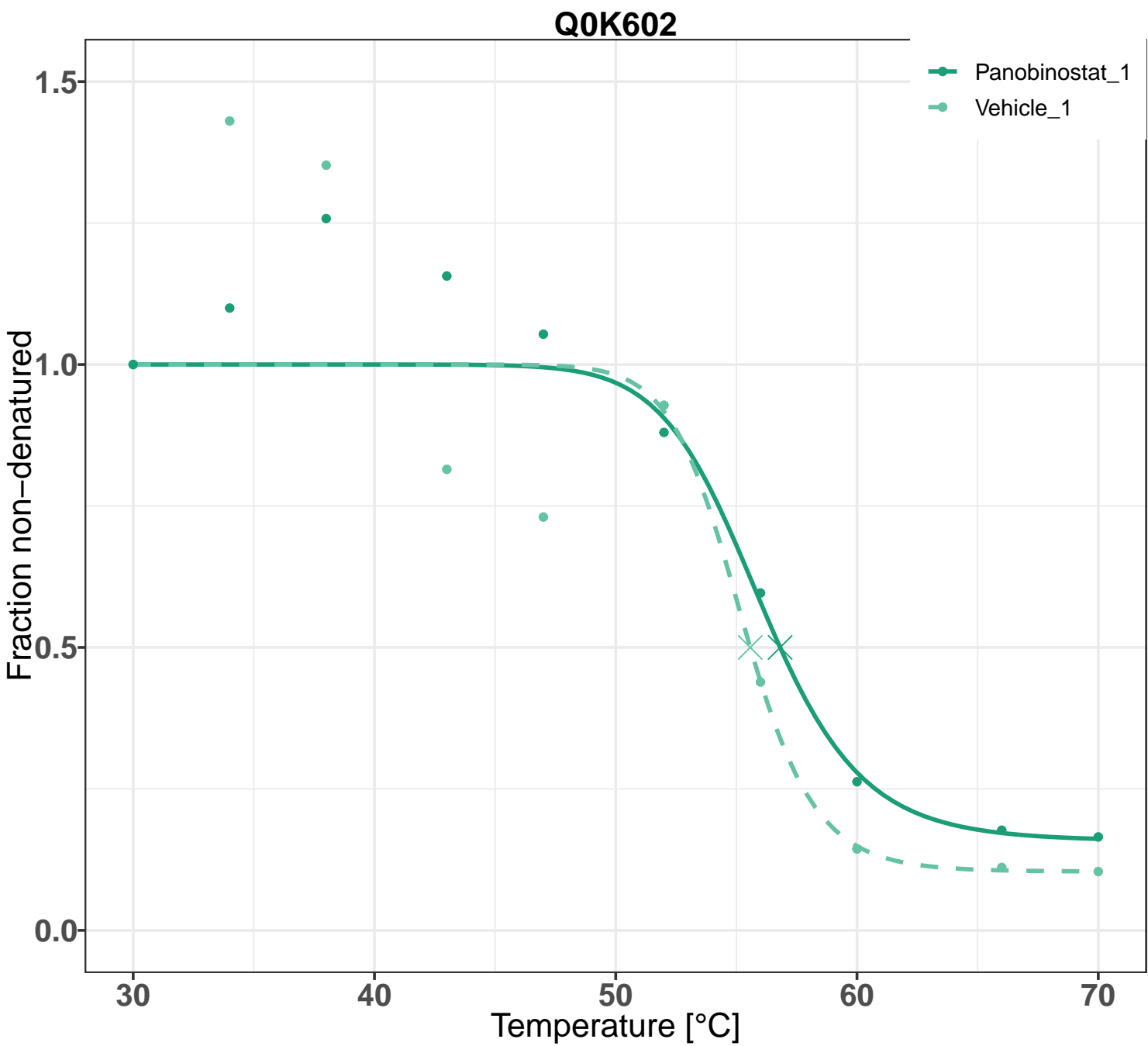

|                | meltPoint | slope | plateau | R2   |
|----------------|-----------|-------|---------|------|
| Panobinostat_1 | 56.81     | -0.1  | 0.16    | 0.94 |
| Vehicle_1      | 55.58     | -0.15 | 0.1     | 0.81 |

Supplement: Supplementary file 2 — Supplementary Material 2 [file 41598_2026_35990_MOESM2_ESM.zip › AllTheTPPData/D40vD86/Panobinostat_Vignette/Melting_Curves/meltCurve_Q0K602.pdf]

# Q0K603

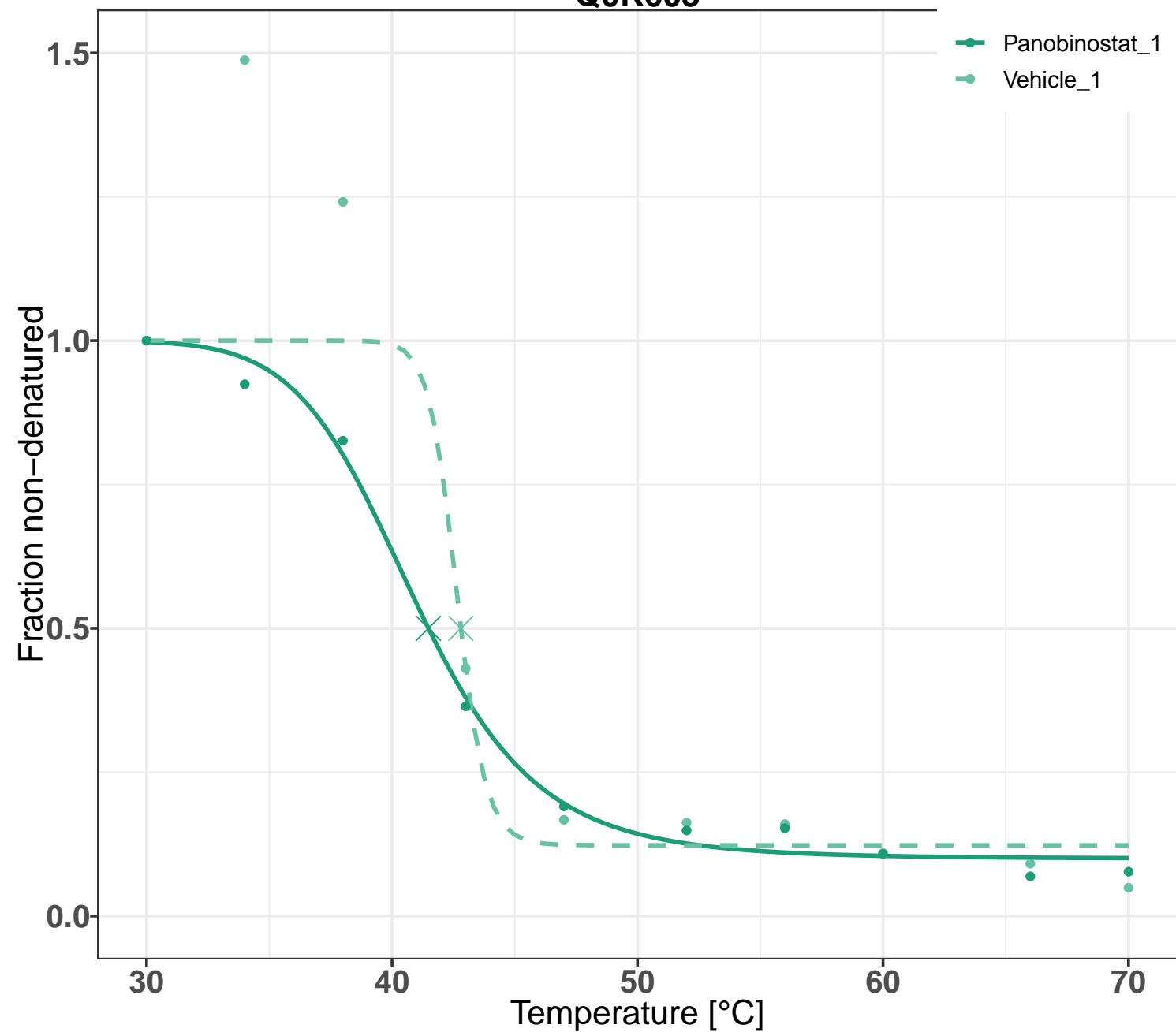

**meltPoint**

**slope**

**plateau**

**R2**

**Panobinostat\_1**

**41.48**

**-0.092**

**0.1**

**0.99**

**Vehicle\_1**

**42.8**

**-0.38**

**0.12**

**0.88**

Supplement: Supplementary file 2 — Supplementary Material 2 [file 41598_2026_35990_MOESM2_ESM.zip › AllTheTPPData/D40vD86/Panobinostat_Vignette/Melting_Curves/meltCurve_Q0K603.pdf]

# Q0K604

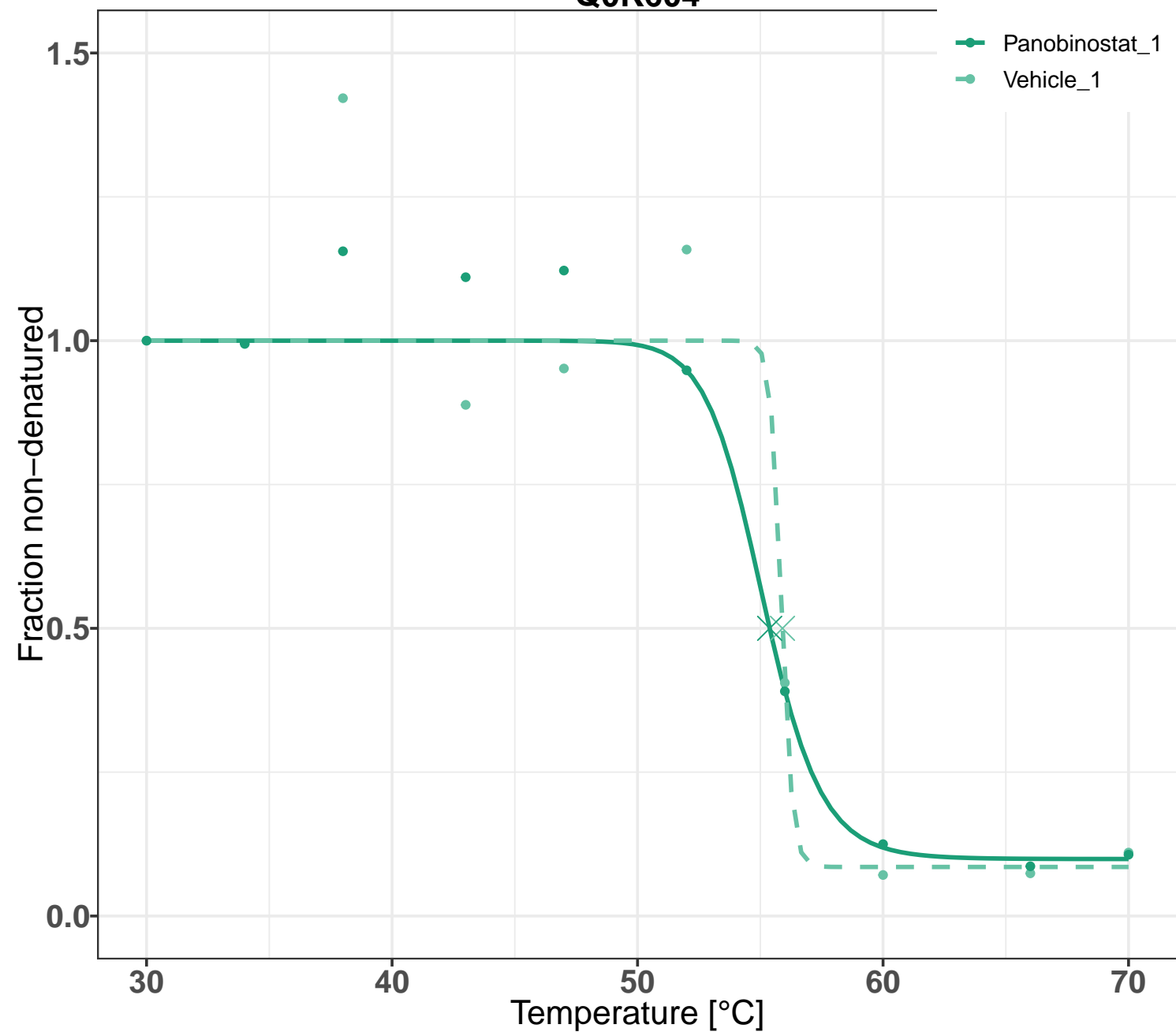

meltPoint

slope

plateau

R2

Panobinostat\_1

55.38

-0.19

0.1

0.97

Vehicle\_1

55.9

-1

0.09

0.74

Supplement: Supplementary file 2 — Supplementary Material 2 [file 41598_2026_35990_MOESM2_ESM.zip › AllTheTPPData/D40vD86/Panobinostat_Vignette/Melting_Curves/meltCurve_Q0K604.pdf]

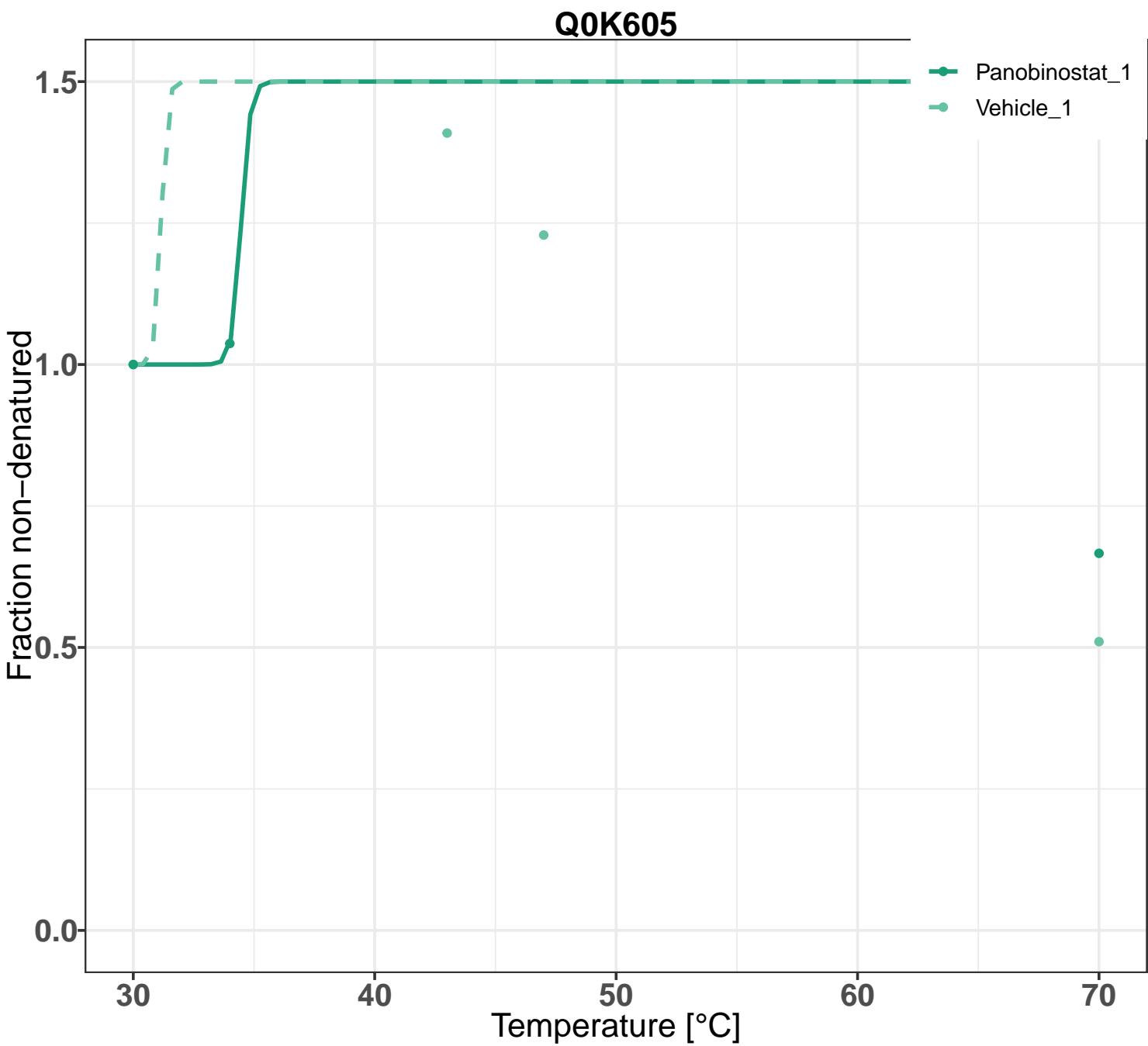

|                | meltPoint | slope | plateau | R2    |
|----------------|-----------|-------|---------|-------|
| Panobinostat_1 | –         | 0.67  | 1.5     | –0.16 |
| Vehicle_1      | –         | 1     | 1.5     | –0.6  |

Supplement: Supplementary file 2 — Supplementary Material 2 [file 41598_2026_35990_MOESM2_ESM.zip › AllTheTPPData/D40vD86/Panobinostat_Vignette/Melting_Curves/meltCurve_Q0K605.pdf]

# Q0K610

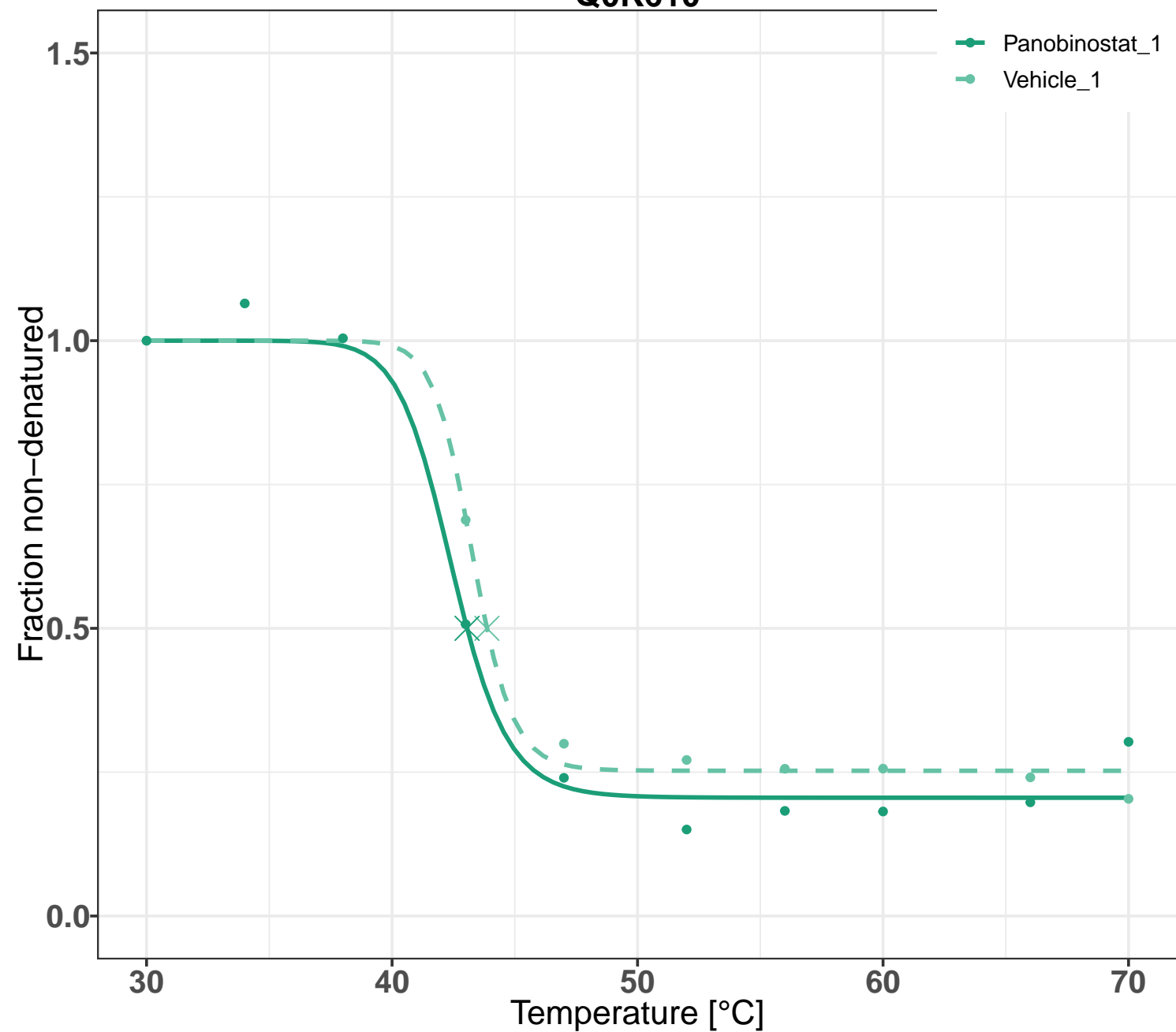

meltPoint

slope

plateau

R2

Panobinostat\_1

43.06

-0.18

0.21

0.99

Vehicle\_1

43.86

-0.23

0.25

0.65

Supplement: Supplementary file 2 — Supplementary Material 2 [file 41598_2026_35990_MOESM2_ESM.zip › AllTheTPPData/D40vD86/Panobinostat_Vignette/Melting_Curves/meltCurve_Q0K610.pdf]

# Q0K611

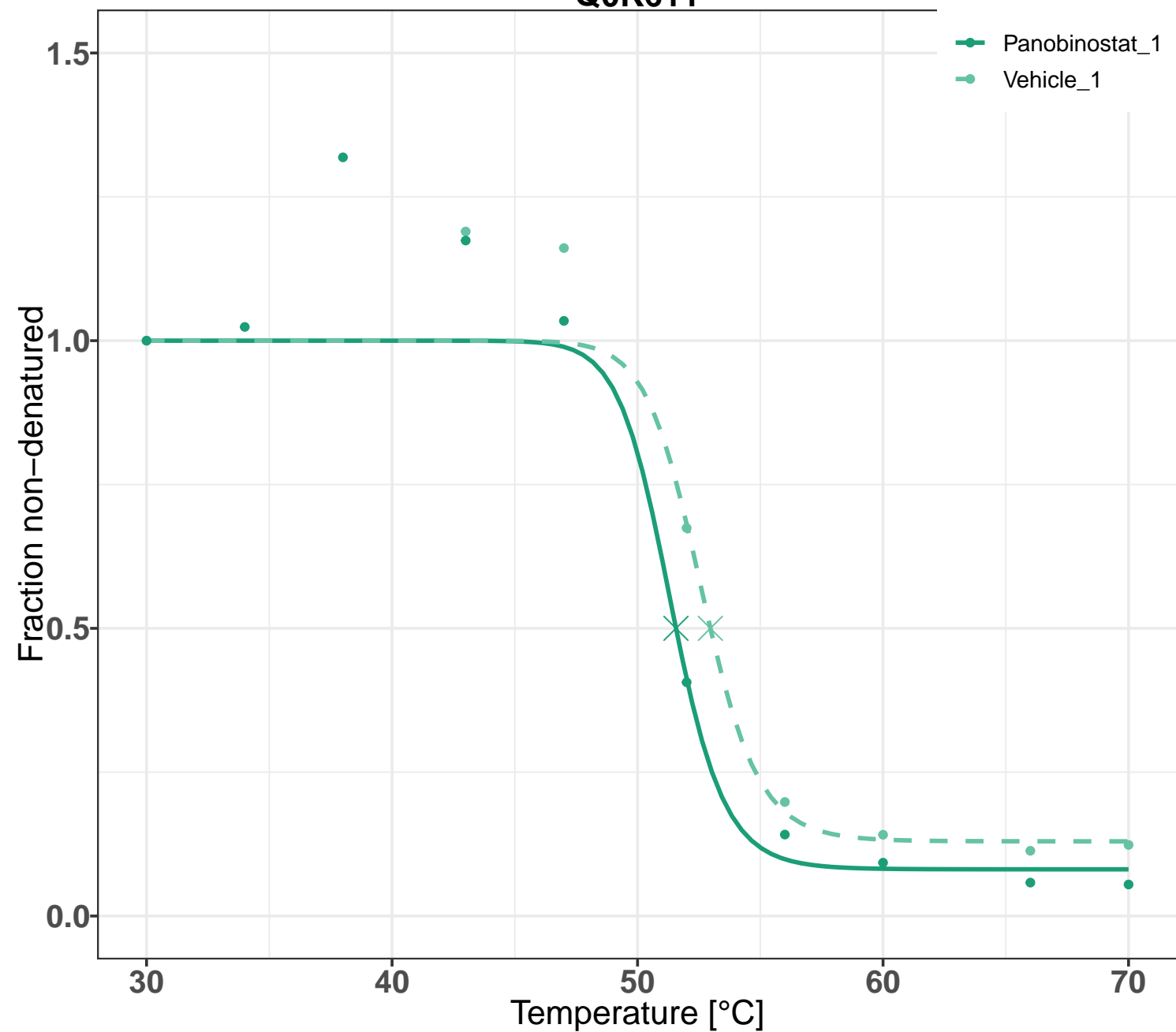

meltPoint

slope

plateau

R2

Panobinostat\_1

51.57

-0.21

0.08

0.94

Vehicle\_1

52.97

-0.19

0.13

0.78

Supplement: Supplementary file 2 — Supplementary Material 2 [file 41598_2026_35990_MOESM2_ESM.zip › AllTheTPPData/D40vD86/Panobinostat_Vignette/Melting_Curves/meltCurve_Q0K611.pdf]

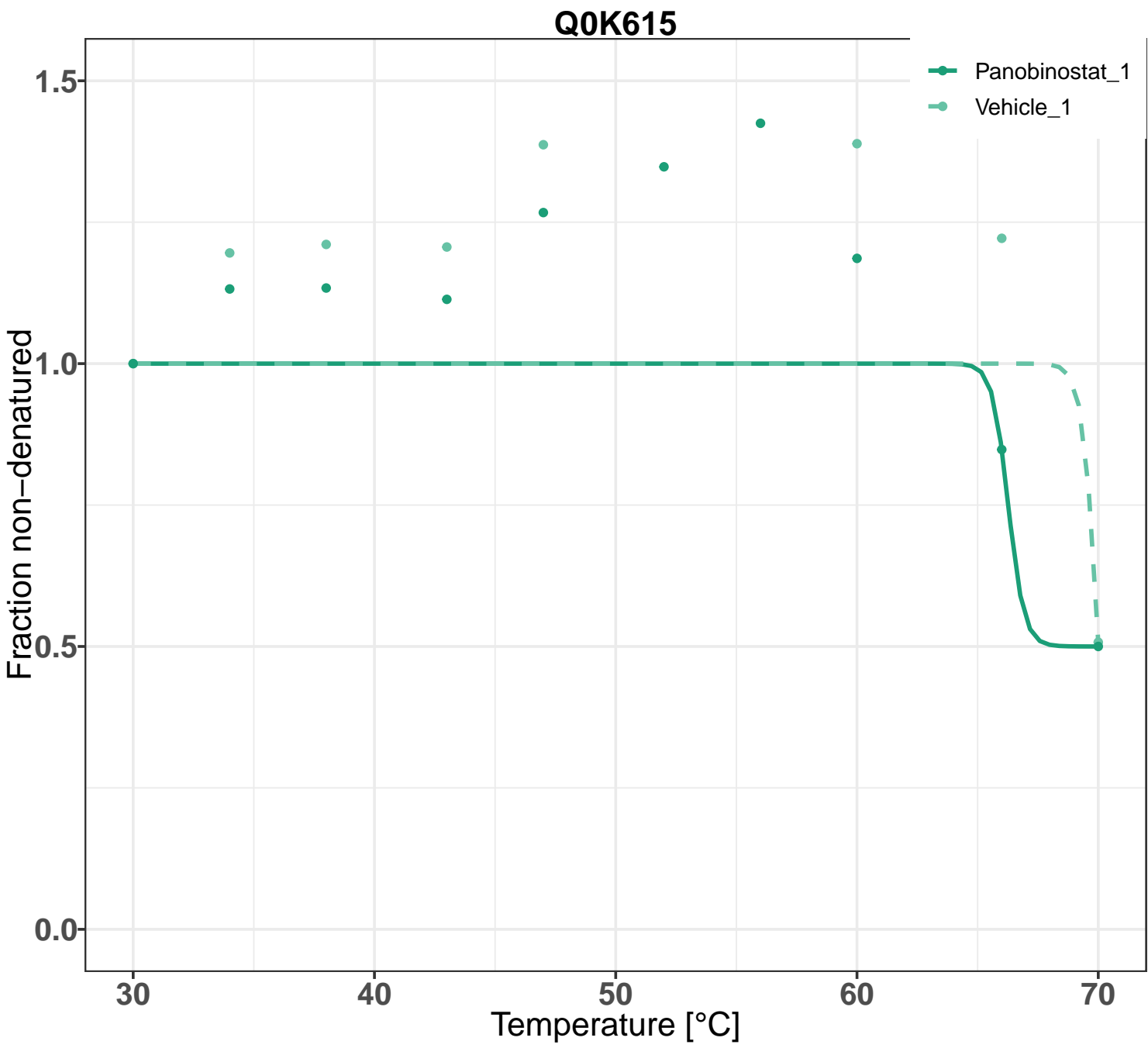

|                | meltPoint | slope | plateau | R2   |
|----------------|-----------|-------|---------|------|
| Panobinostat_1 | –         | –0.38 | 0.5     | 0.29 |
| Vehicle_1      | –         | –     | 0       | –0.4 |

Supplement: Supplementary file 2 — Supplementary Material 2 [file 41598_2026_35990_MOESM2_ESM.zip › AllTheTPPData/D40vD86/Panobinostat_Vignette/Melting_Curves/meltCurve_Q0K615.pdf]

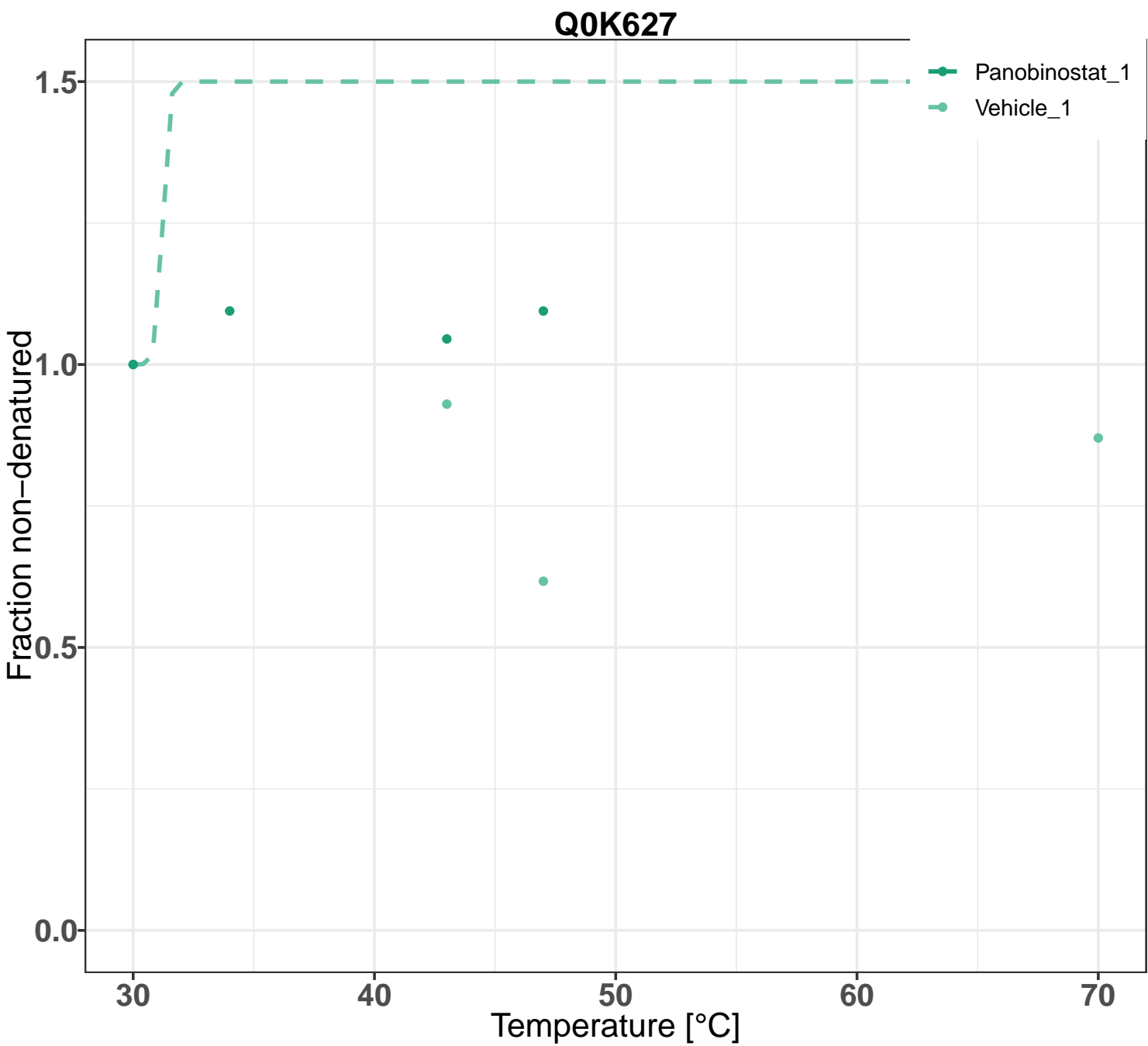

|                | meltPoint | slope | plateau | R2    |
|----------------|-----------|-------|---------|-------|
| Panobinostat_1 | –         | –     | –       | –     |
| Vehicle_1      | –         | 1     | 1.5     | –0.11 |

Supplement: Supplementary file 2 — Supplementary Material 2 [file 41598_2026_35990_MOESM2_ESM.zip › AllTheTPPData/D40vD86/Panobinostat_Vignette/Melting_Curves/meltCurve_Q0K627.pdf]

# Q0K628

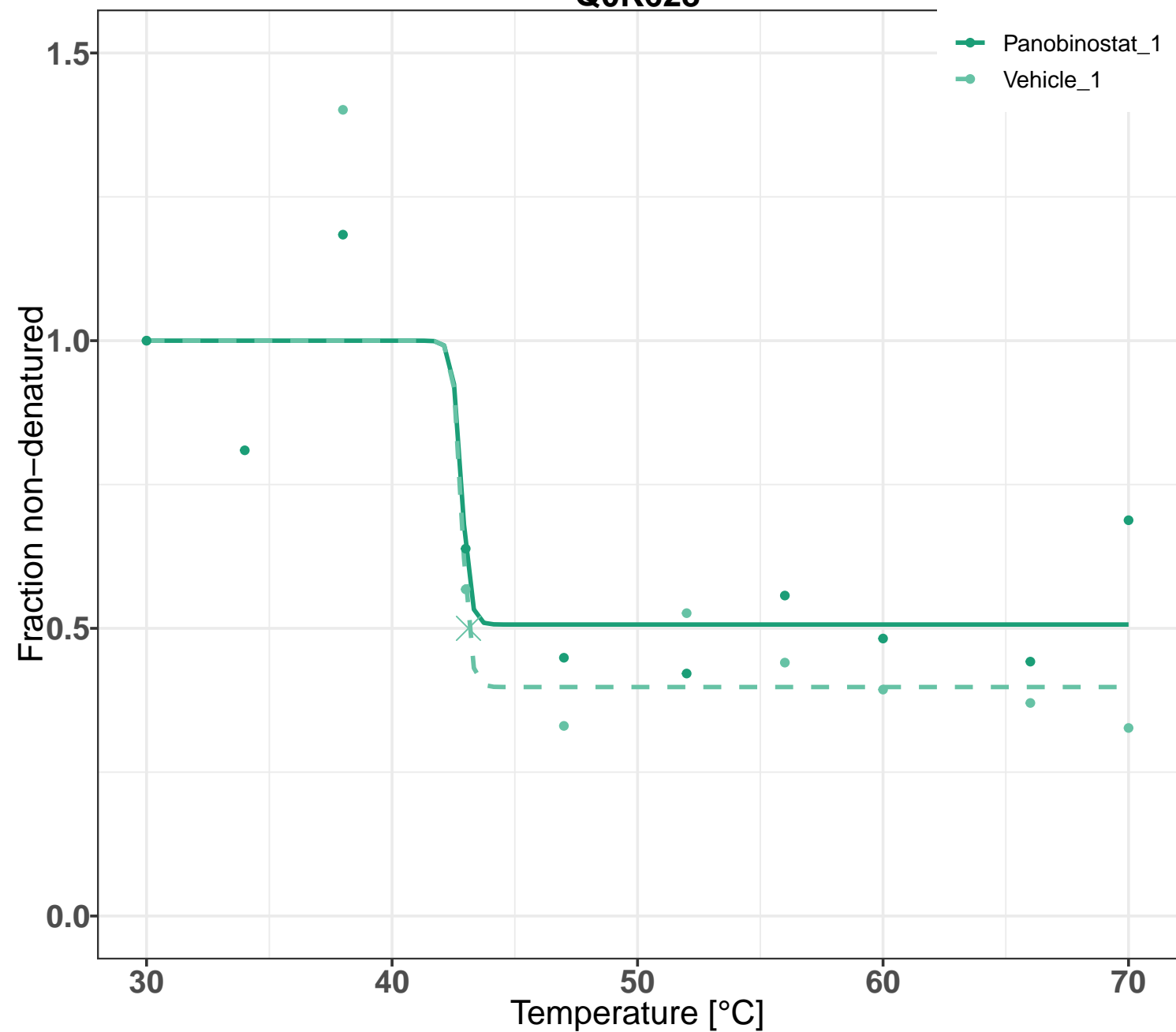

meltPoint

slope

plateau

R2

Panobinostat\_1

–

–0.7

0.51

0.8

Vehicle\_1

43.11

–0.88

0.4

0.73

Supplement: Supplementary file 2 — Supplementary Material 2 [file 41598_2026_35990_MOESM2_ESM.zip › AllTheTPPData/D40vD86/Panobinostat_Vignette/Melting_Curves/meltCurve_Q0K628.pdf]

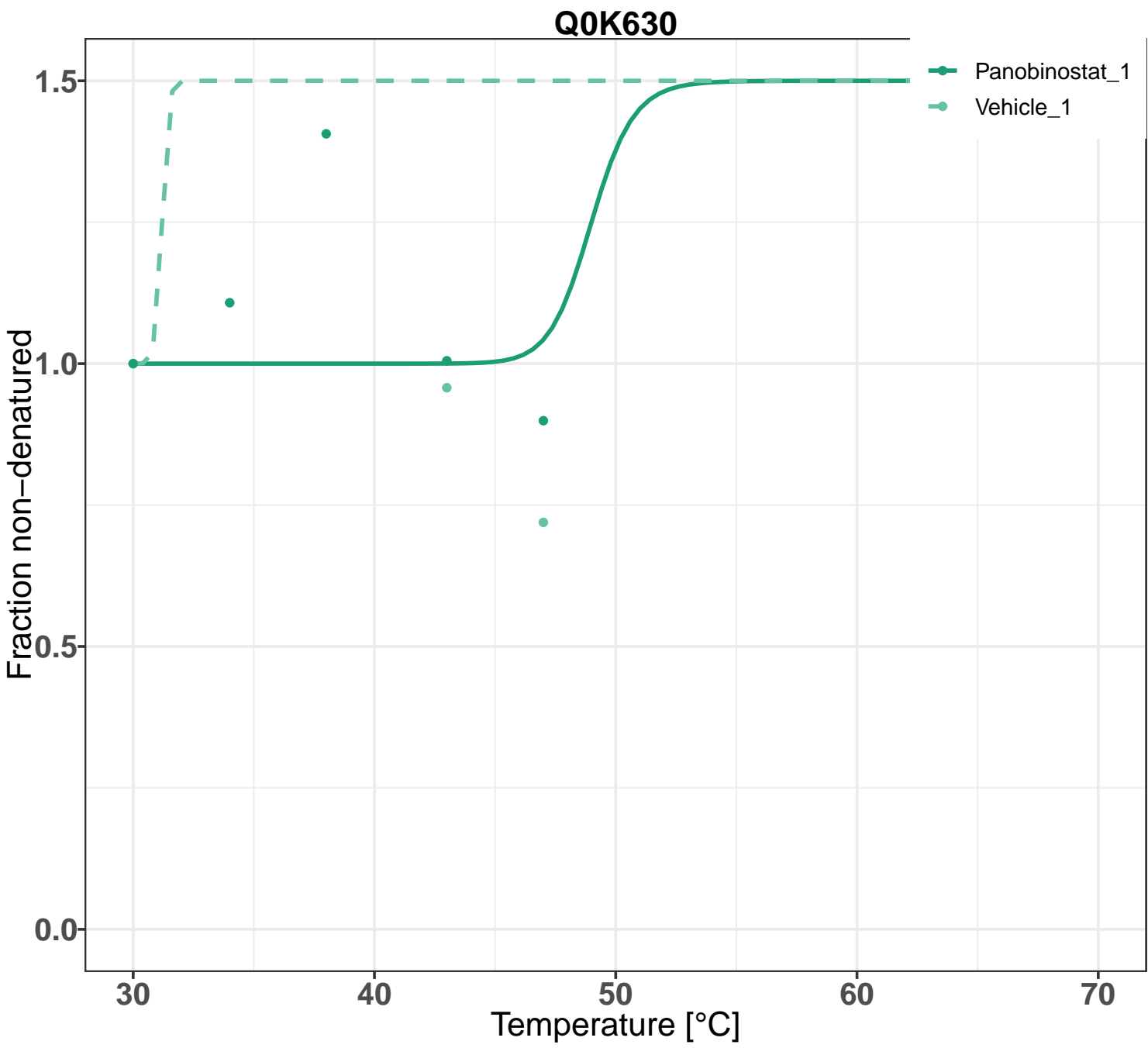

|                | meltPoint | slope | plateau | R2    |
|----------------|-----------|-------|---------|-------|
| Panobinostat_1 | –         | 0.14  | 1.5     | –0.15 |
| Vehicle_1      | –         | 1     | 1.5     | –0.14 |

Supplement: Supplementary file 2 — Supplementary Material 2 [file 41598_2026_35990_MOESM2_ESM.zip › AllTheTPPData/D40vD86/Panobinostat_Vignette/Melting_Curves/meltCurve_Q0K630.pdf]

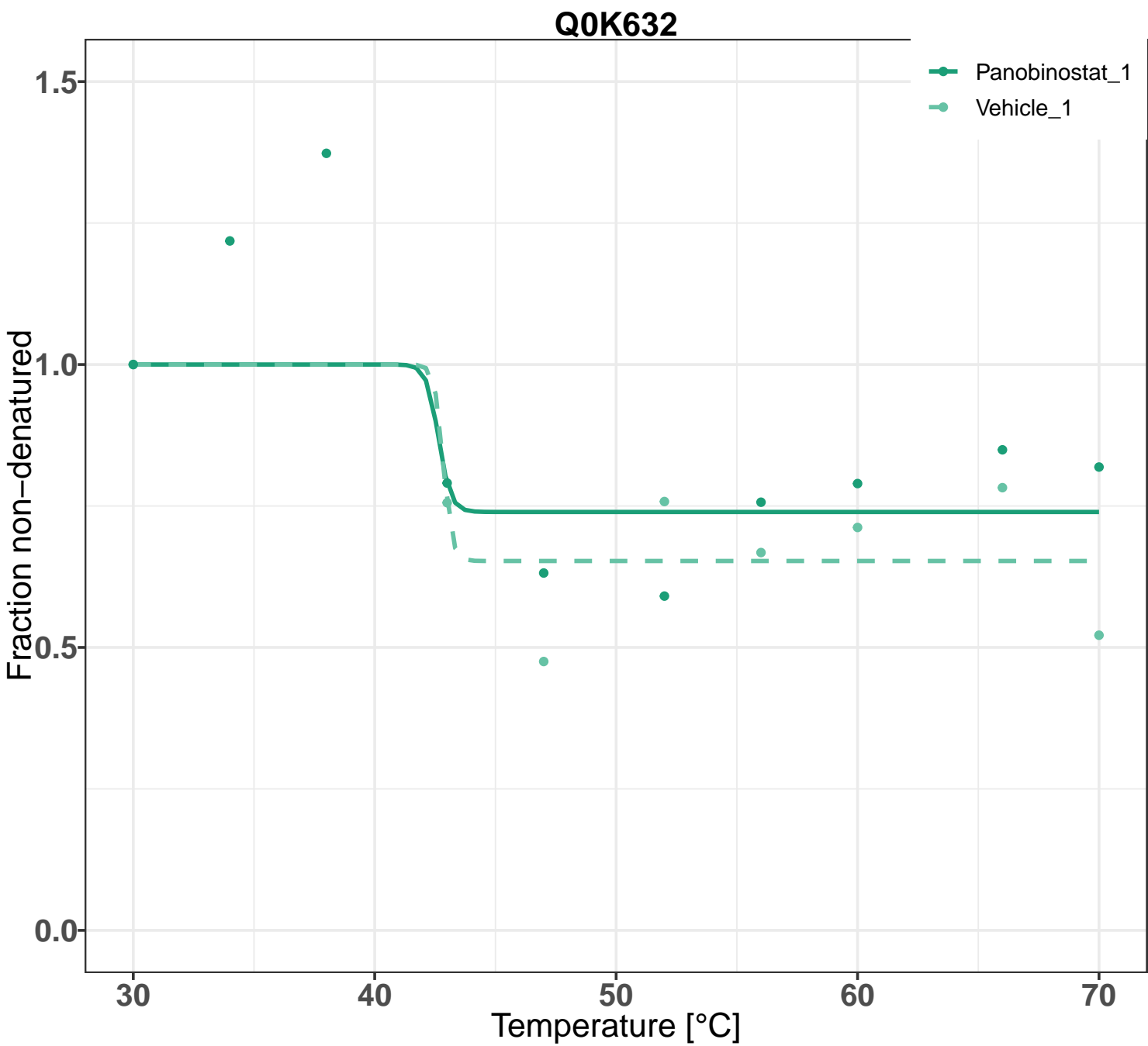

|                | meltPoint | slope | plateau | R2   |
|----------------|-----------|-------|---------|------|
| Panobinostat_1 | —         | -0.26 | 0.74    | 0.56 |
| Vehicle_1      | —         | -0.47 | 0.65    | 0.4  |

Supplement: Supplementary file 2 — Supplementary Material 2 [file 41598_2026_35990_MOESM2_ESM.zip › AllTheTPPData/D40vD86/Panobinostat_Vignette/Melting_Curves/meltCurve_Q0K632.pdf]

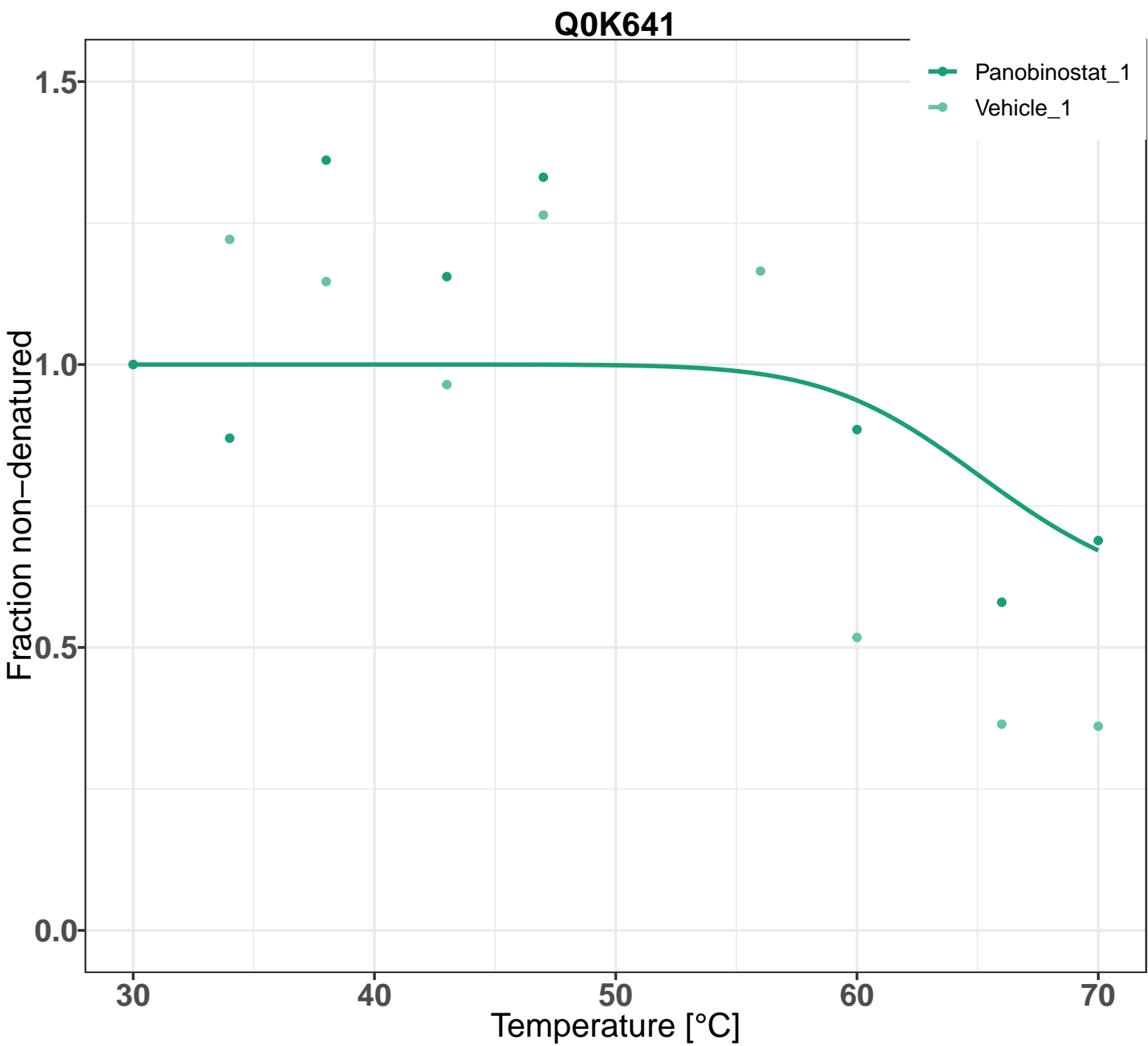

|                | meltPoint | slope  | plateau | R2   |
|----------------|-----------|--------|---------|------|
| Panobinostat_1 | –         | –0.031 | 0.56    | 0.11 |
| Vehicle_1      | –         | –      | –       | –    |

Supplement: Supplementary file 2 — Supplementary Material 2 [file 41598_2026_35990_MOESM2_ESM.zip › AllTheTPPData/D40vD86/Panobinostat_Vignette/Melting_Curves/meltCurve_Q0K641.pdf]

# Q0K644

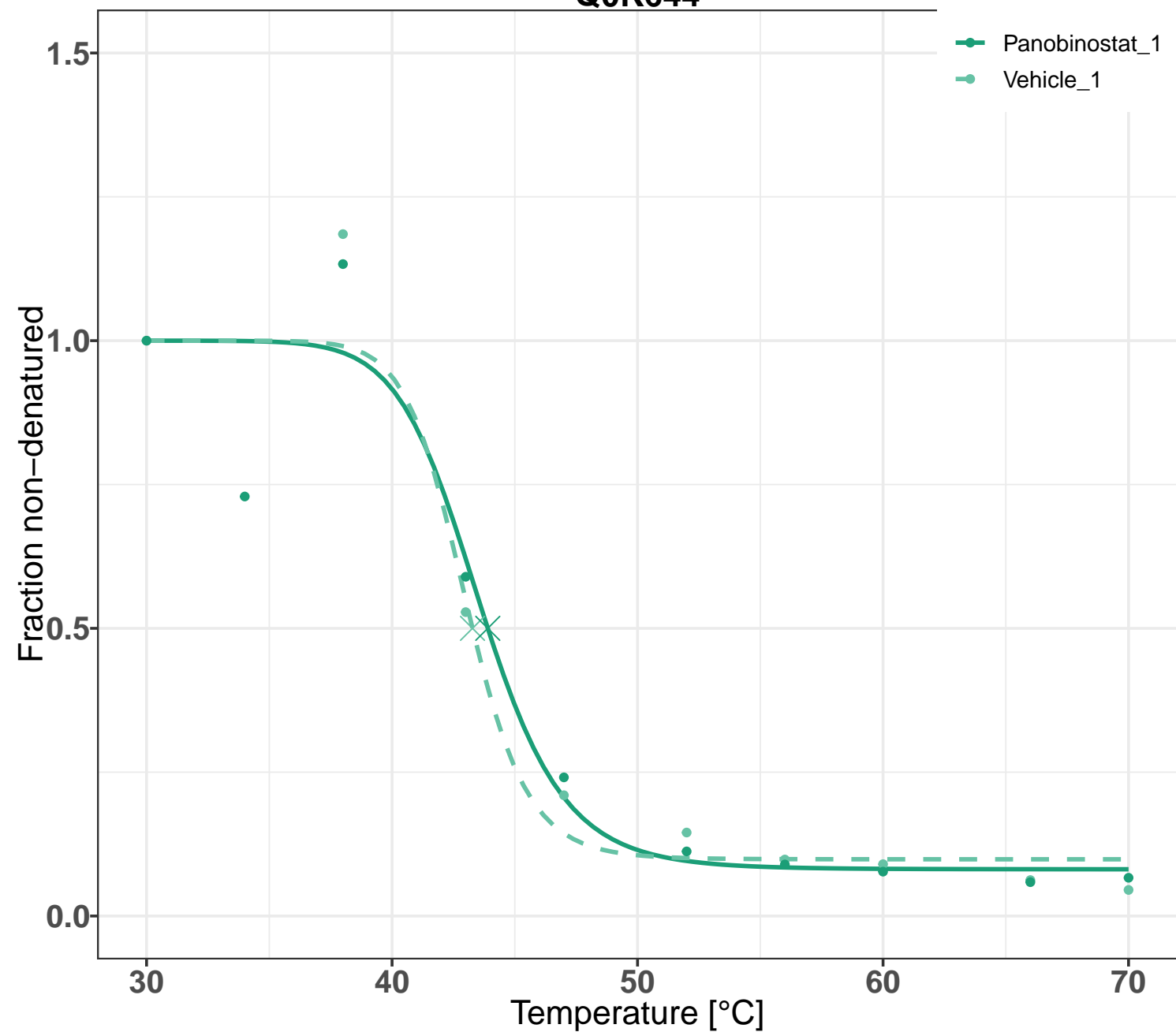

meltPoint

slope

plateau

R2

Panobinostat\_1

43.9

-0.14

0.08

0.94

Vehicle\_1

43.28

-0.18

0.1

0.78

Supplement: Supplementary file 2 — Supplementary Material 2 [file 41598_2026_35990_MOESM2_ESM.zip › AllTheTPPData/D40vD86/Panobinostat_Vignette/Melting_Curves/meltCurve_Q0K644.pdf]

# Q0K645

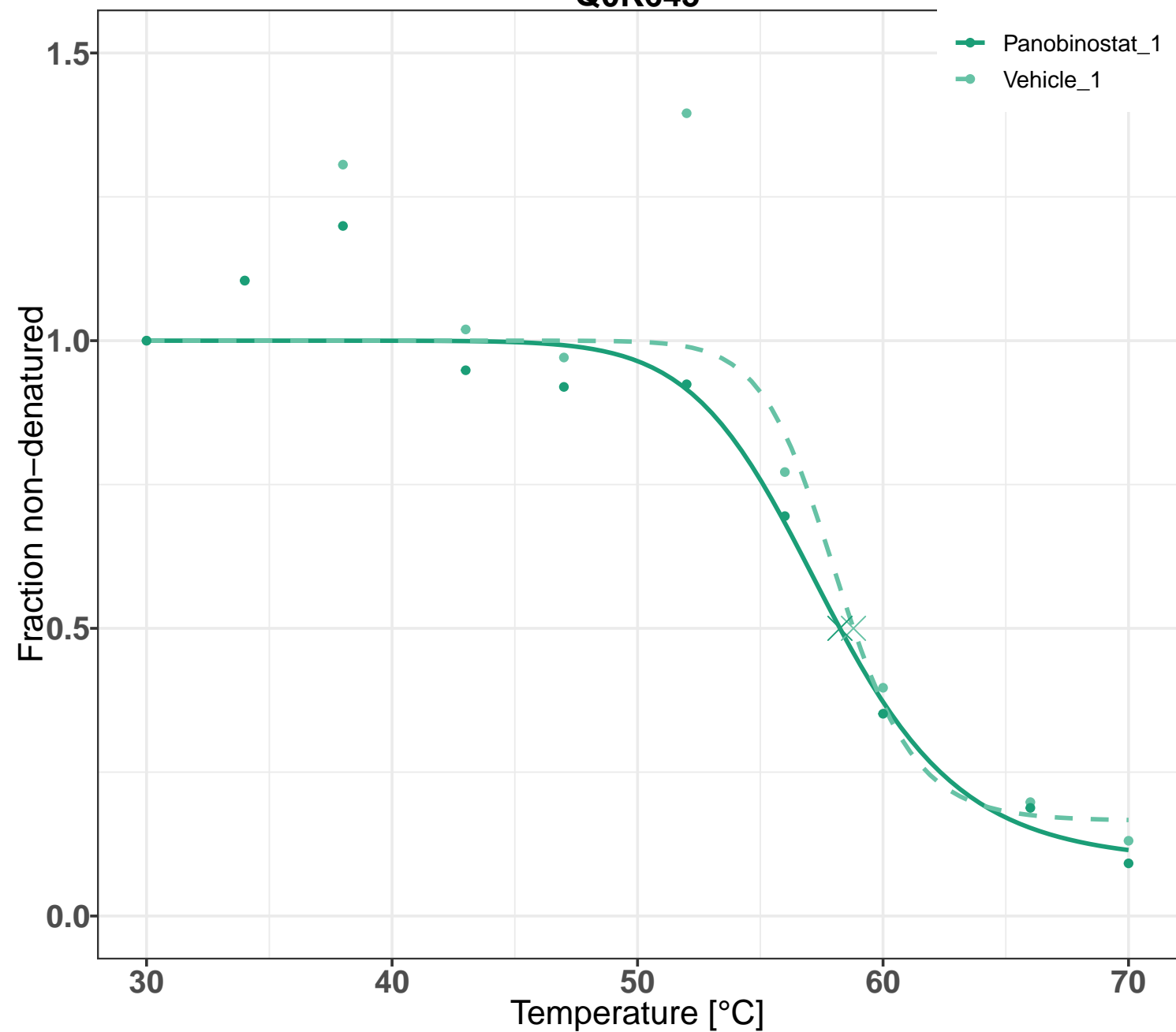

|                | meltPoint | slope  | plateau | R2   |
|----------------|-----------|--------|---------|------|
| Panobinostat_1 | 58.24     | -0.083 | 0.09    | 0.96 |
| Vehicle_1      | 58.79     | -0.13  | 0.17    | 0.84 |

Supplement: Supplementary file 2 — Supplementary Material 2 [file 41598_2026_35990_MOESM2_ESM.zip › AllTheTPPData/D40vD86/Panobinostat_Vignette/Melting_Curves/meltCurve_Q0K645.pdf]

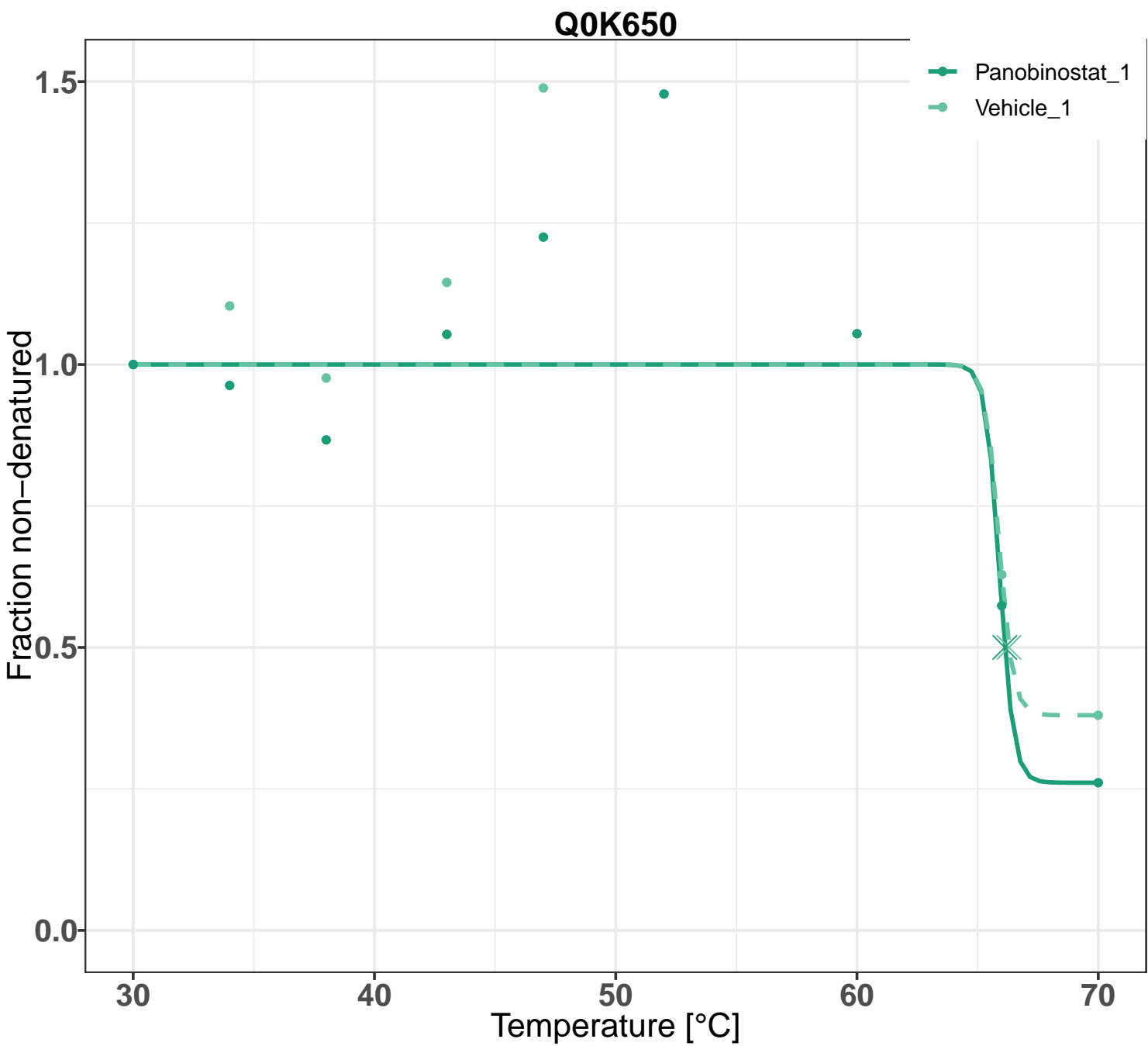

|                | meltPoint | slope | plateau | R2    |
|----------------|-----------|-------|---------|-------|
| Panobinostat_1 | 66.12     | -0.64 | 0.26    | 0.56  |
| Vehicle_1      | 66.3      | -0.54 | 0.38    | -0.03 |

Supplement: Supplementary file 2 — Supplementary Material 2 [file 41598_2026_35990_MOESM2_ESM.zip › AllTheTPPData/D40vD86/Panobinostat_Vignette/Melting_Curves/meltCurve_Q0K650.pdf]

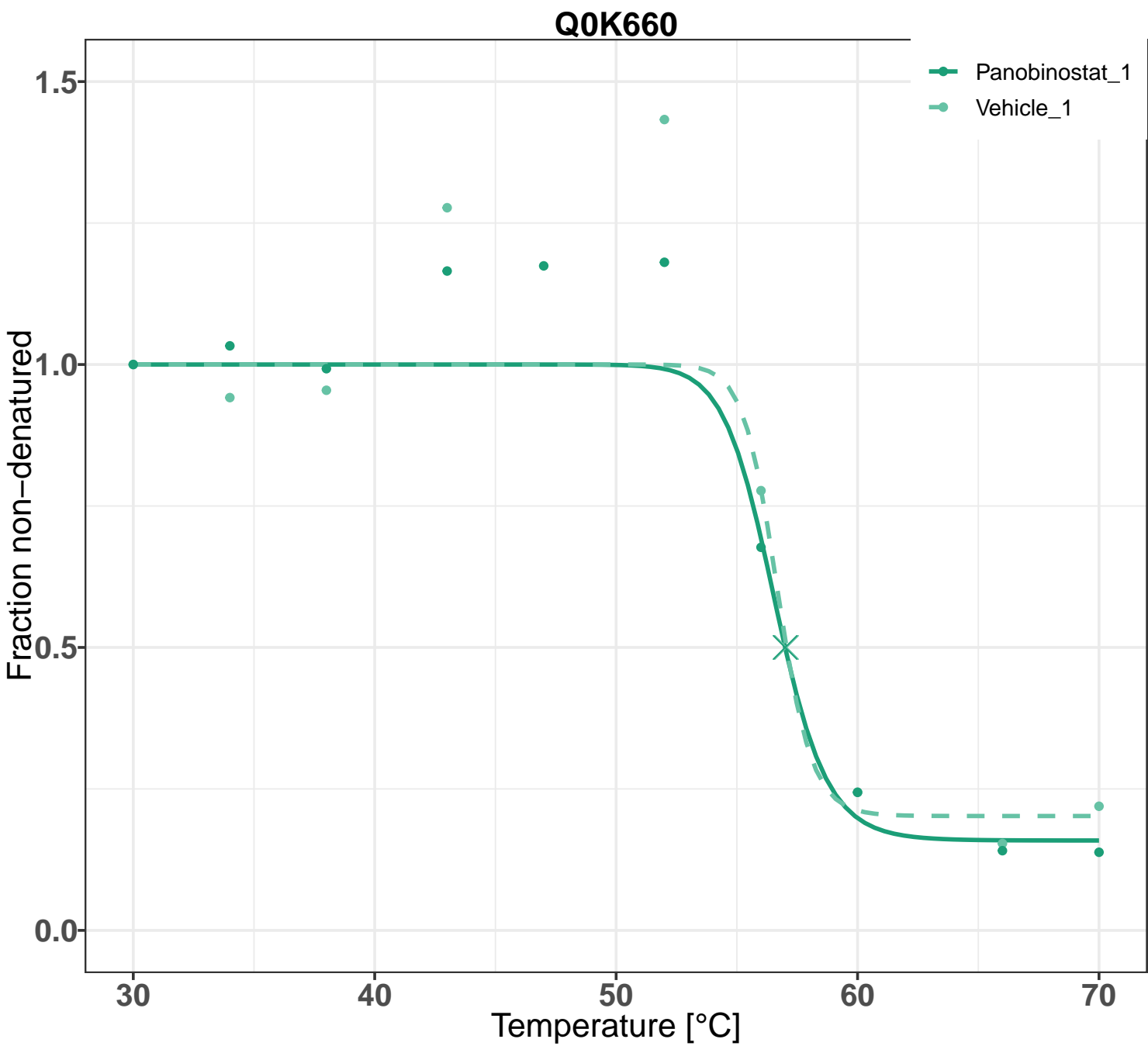

|                | meltPoint | slope | plateau | R2   |
|----------------|-----------|-------|---------|------|
| Panobinostat_1 | 57        | -0.2  | 0.16    | 0.94 |
| Vehicle_1      | 57.06     | -0.28 | 0.2     | 0.74 |

Supplement: Supplementary file 2 — Supplementary Material 2 [file 41598_2026_35990_MOESM2_ESM.zip › AllTheTPPData/D40vD86/Panobinostat_Vignette/Melting_Curves/meltCurve_Q0K660.pdf]

# Q0K672

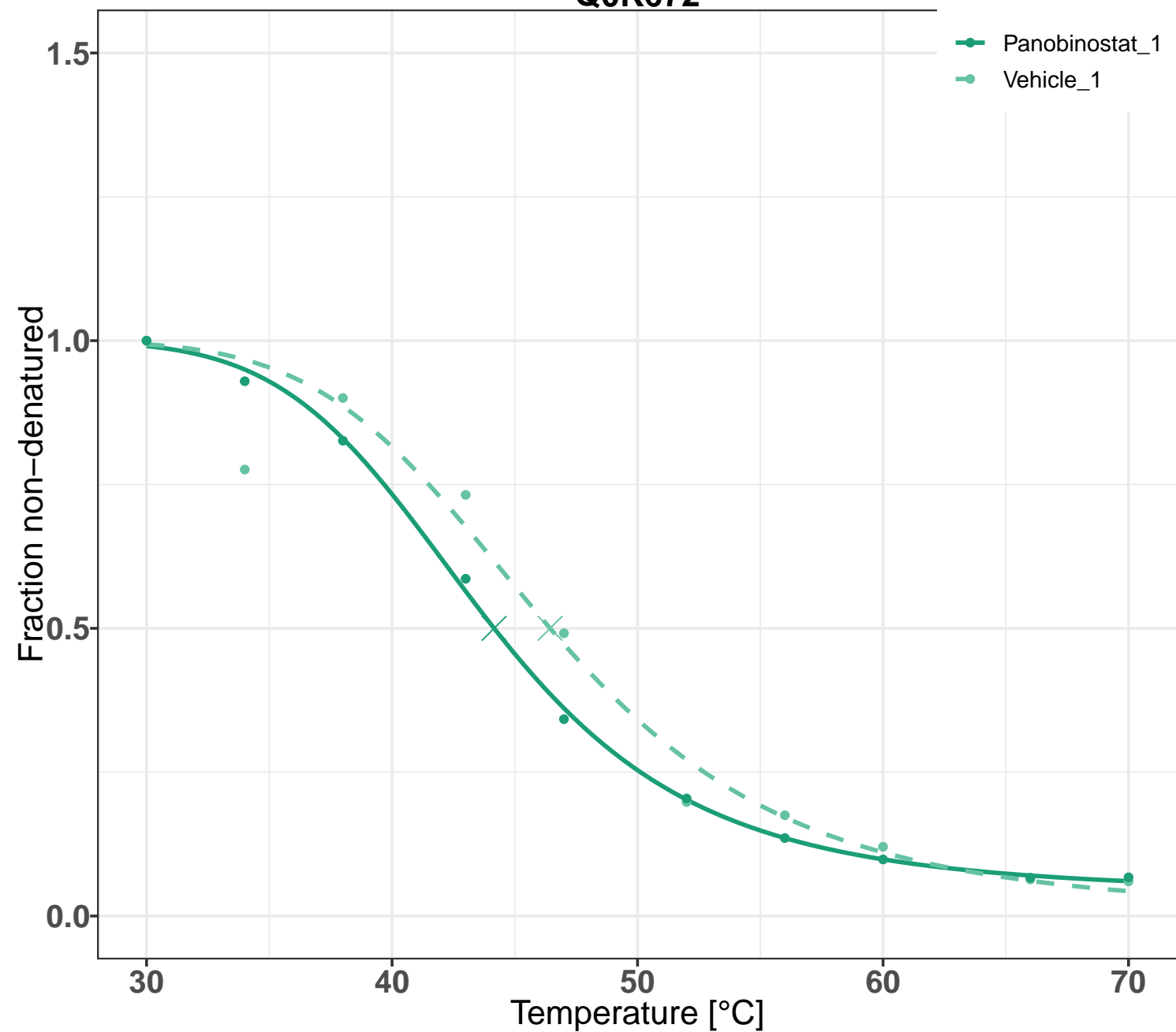

**meltPoint**

**slope**

**plateau**

**R2**

**Panobinostat\_1**

**44.16**

**-0.057**

**0.04**

**1**

**Vehicle\_1**

**46.44**

**-0.052**

**0**

**0.96**

Supplement: Supplementary file 2 — Supplementary Material 2 [file 41598_2026_35990_MOESM2_ESM.zip › AllTheTPPData/D40vD86/Panobinostat_Vignette/Melting_Curves/meltCurve_Q0K672.pdf]

# Q0K673

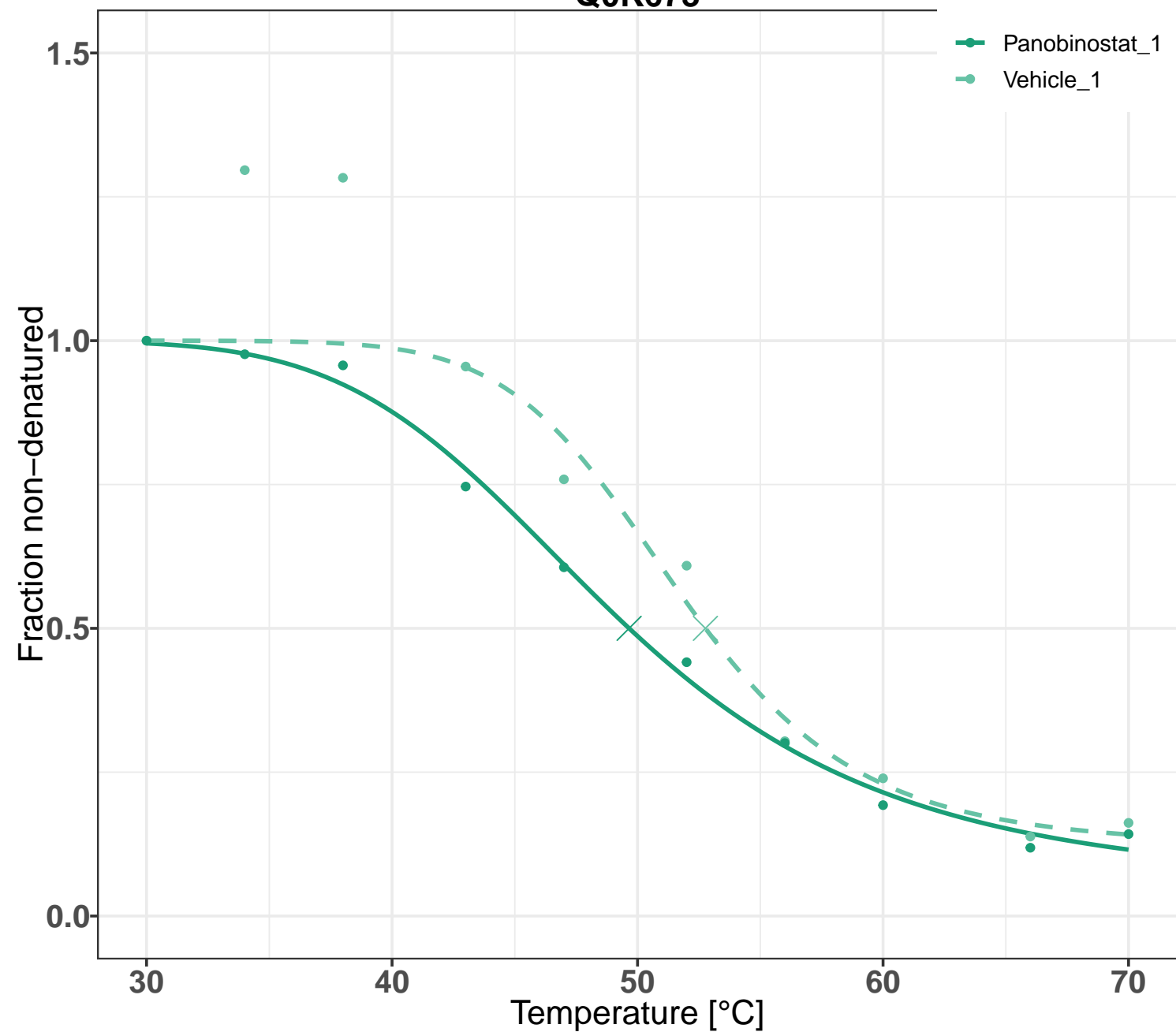

meltPoint

slope

plateau

R2

Panobinostat\_1

49.66

-0.043

0.04

1

Vehicle\_1

52.76

-0.062

0.12

0.9

Supplement: Supplementary file 2 — Supplementary Material 2 [file 41598_2026_35990_MOESM2_ESM.zip › AllTheTPPData/D40vD86/Panobinostat_Vignette/Melting_Curves/meltCurve_Q0K673.pdf]

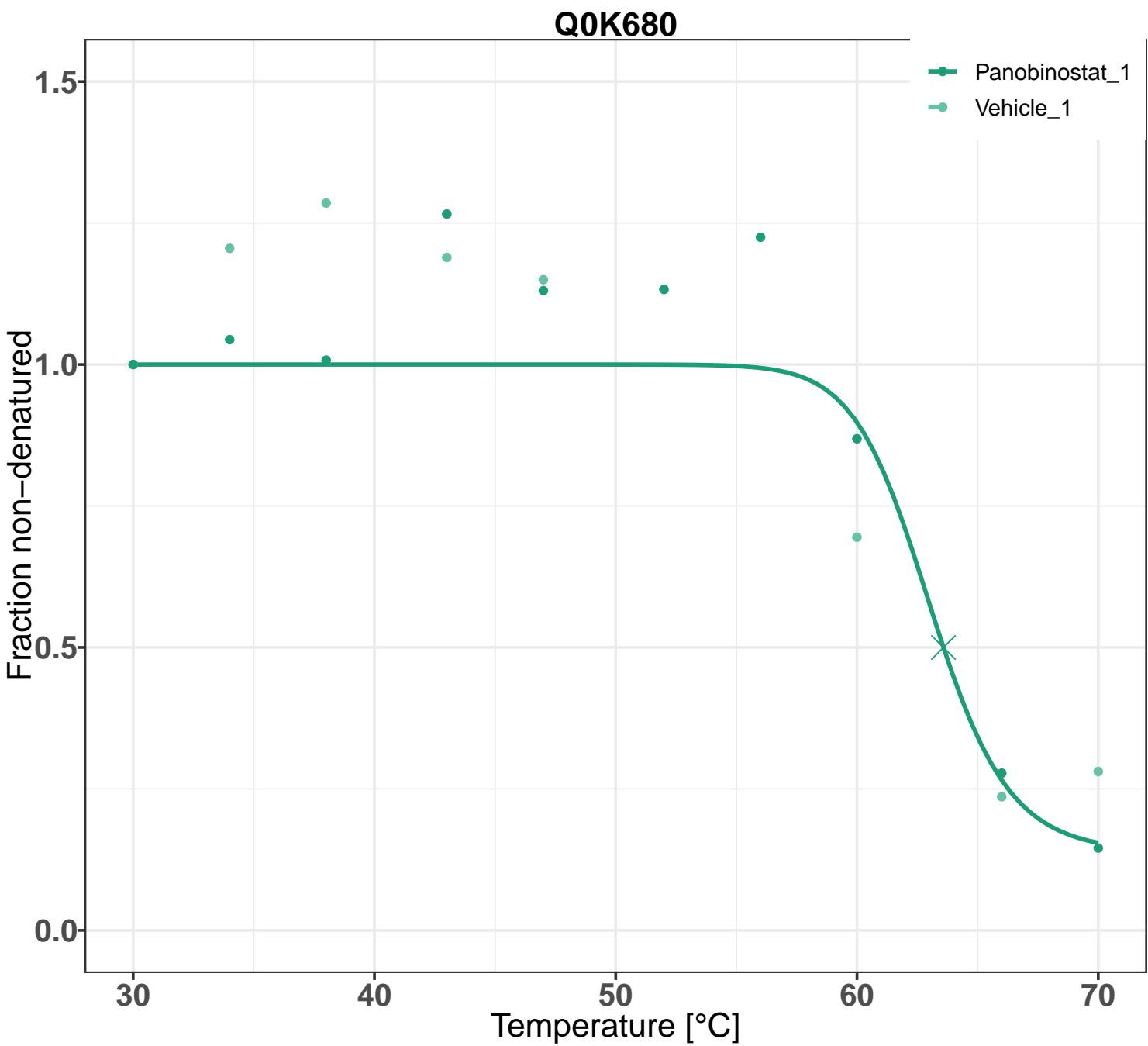

|                | meltPoint | slope | plateau | R2   |
|----------------|-----------|-------|---------|------|
| Panobinostat_1 | 63.58     | -0.13 | 0.14    | 0.88 |
| Vehicle_1      | -         | -     | -       | -    |

Supplement: Supplementary file 2 — Supplementary Material 2 [file 41598_2026_35990_MOESM2_ESM.zip › AllTheTPPData/D40vD86/Panobinostat_Vignette/Melting_Curves/meltCurve_Q0K680.pdf]

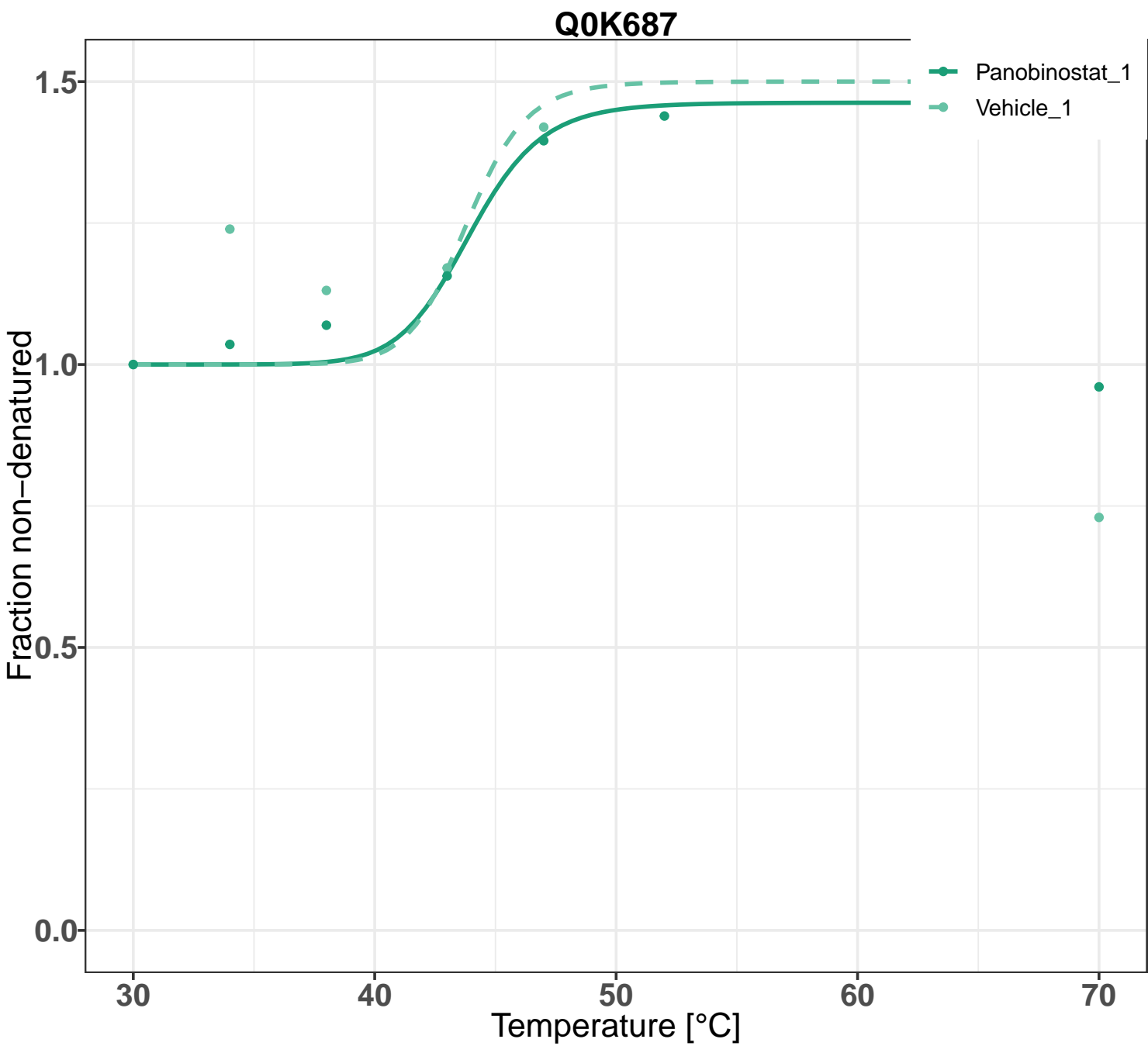

|                | meltPoint | slope | plateau | R2   |
|----------------|-----------|-------|---------|------|
| Panobinostat_1 | —         | 0.078 | 1.46    | 0.44 |
| Vehicle_1      | —         | 0.1   | 1.5     | 0.23 |

Supplement: Supplementary file 2 — Supplementary Material 2 [file 41598_2026_35990_MOESM2_ESM.zip › AllTheTPPData/D40vD86/Panobinostat_Vignette/Melting_Curves/meltCurve_Q0K687.pdf]

# Q0K688

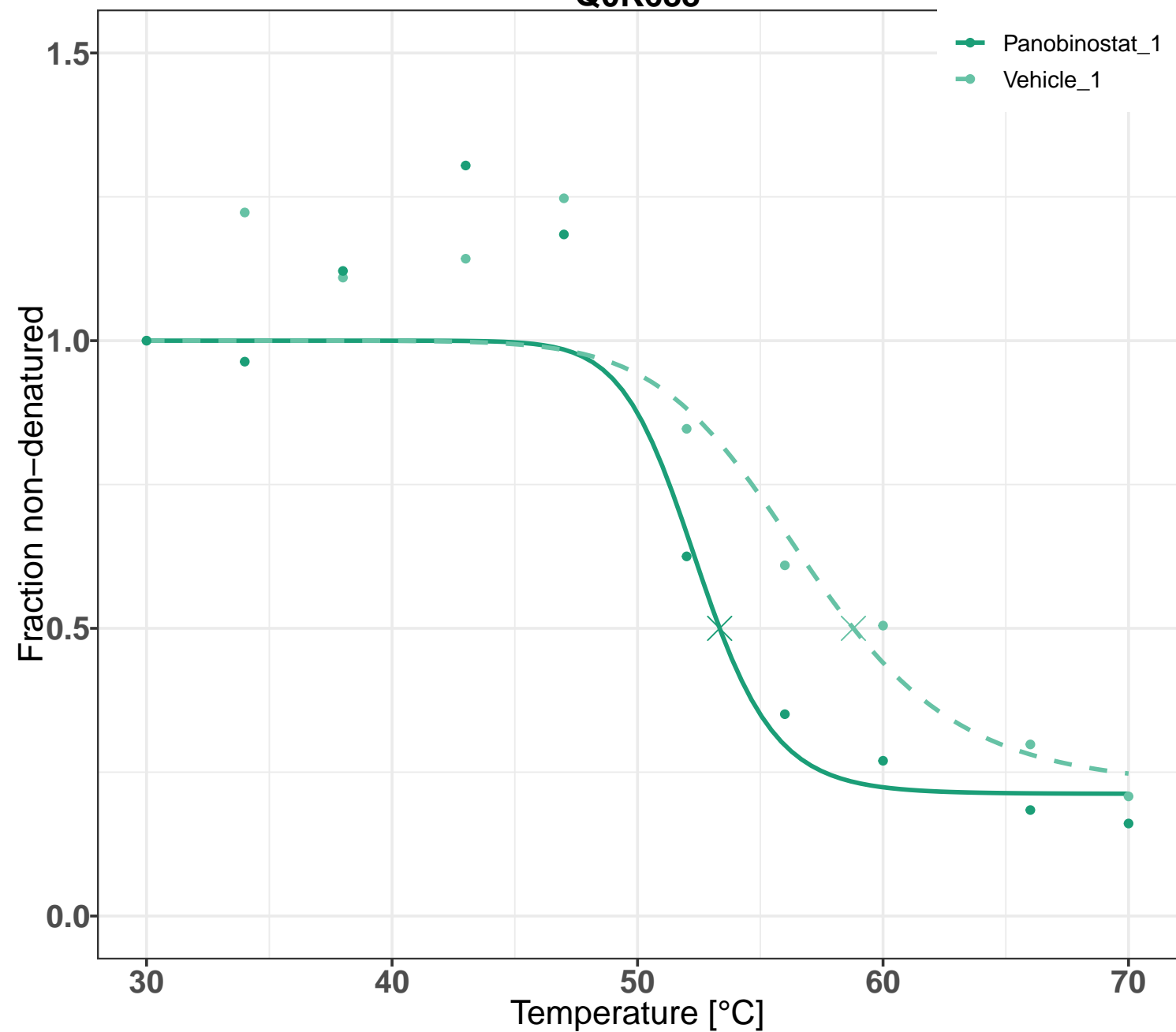

meltPoint

slope

plateau

R2

Panobinostat\_1

53.34

-0.13

0.21

0.91

Vehicle\_1

58.79

-0.063

0.22

0.88

Supplement: Supplementary file 2 — Supplementary Material 2 [file 41598_2026_35990_MOESM2_ESM.zip › AllTheTPPData/D40vD86/Panobinostat_Vignette/Melting_Curves/meltCurve_Q0K688.pdf]

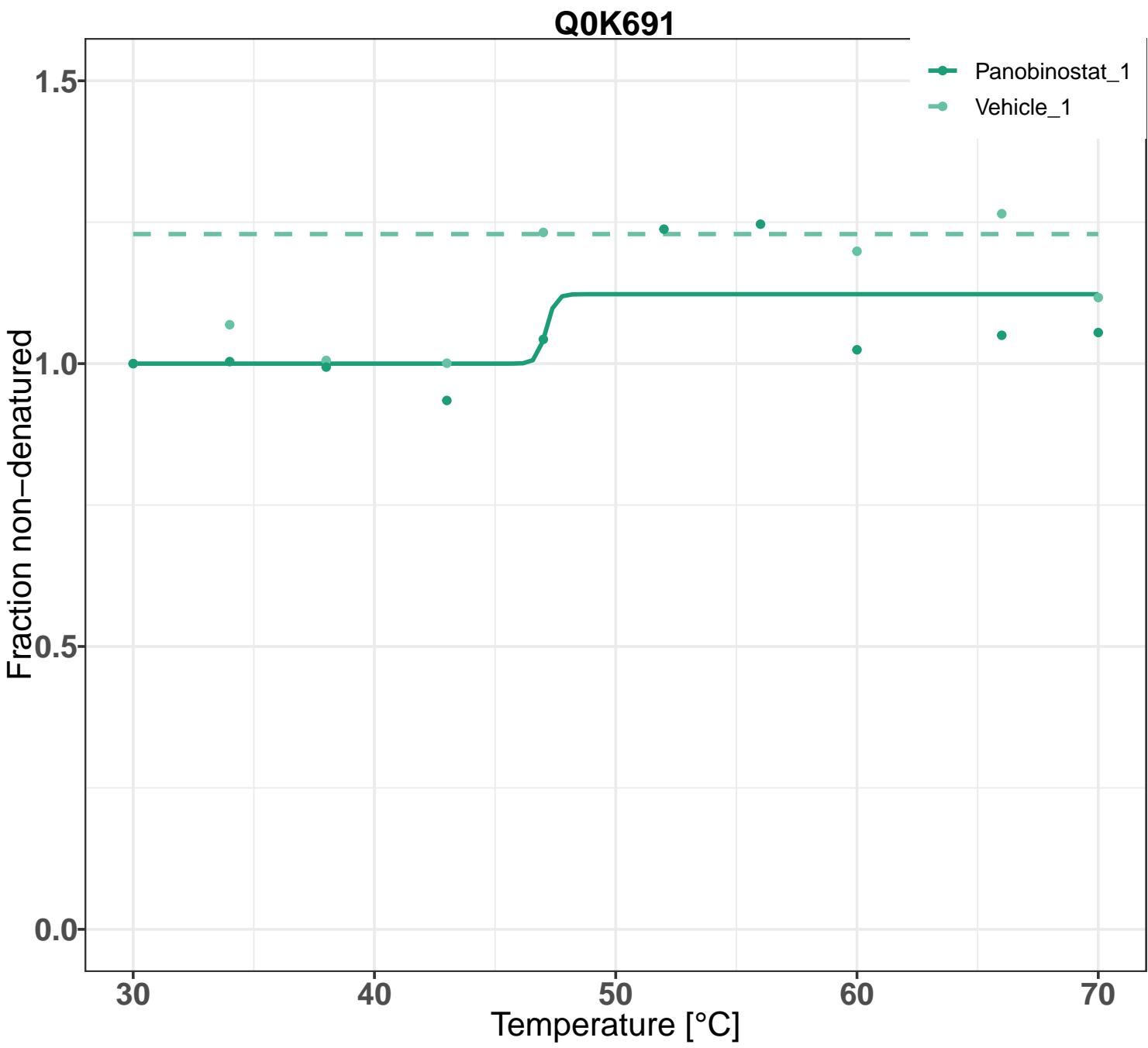

|                | meltPoint | slope | plateau | R2   |
|----------------|-----------|-------|---------|------|
| Panobinostat_1 | –         | 0.16  | 1.12    | 0.45 |
| Vehicle_1      | –         | –     | 1.35    | 0    |

Supplement: Supplementary file 2 — Supplementary Material 2 [file 41598_2026_35990_MOESM2_ESM.zip › AllTheTPPData/D40vD86/Panobinostat_Vignette/Melting_Curves/meltCurve_Q0K691.pdf]

# Q0K692

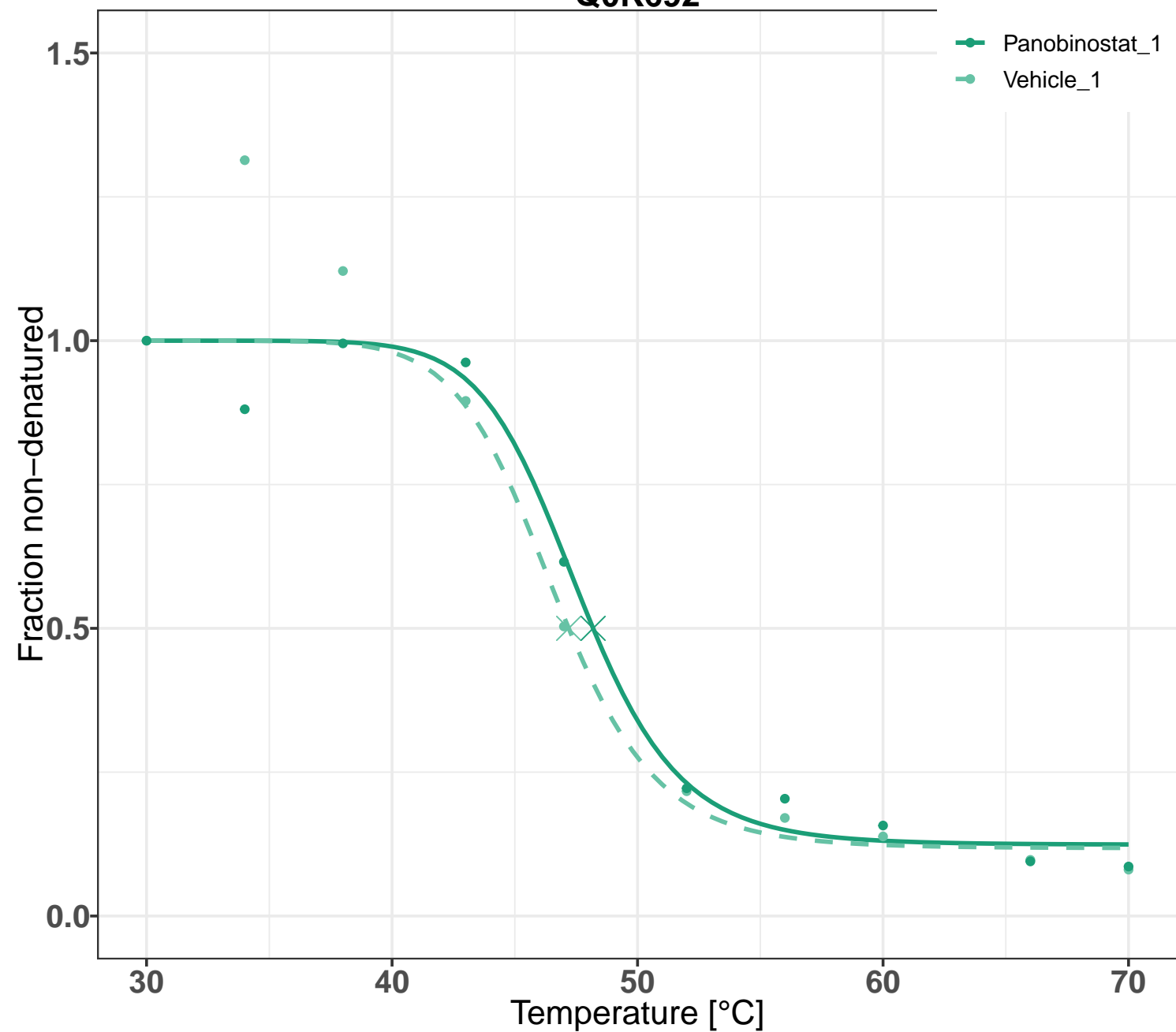

**meltPoint**

**slope**

**plateau**

**R2**

**Panobinostat\_1**

**48.19**

**-0.11**

**0.12**

**0.99**

**Vehicle\_1**

**47.2**

**-0.11**

**0.12**

**0.94**

Supplement: Supplementary file 2 — Supplementary Material 2 [file 41598_2026_35990_MOESM2_ESM.zip › AllTheTPPData/D40vD86/Panobinostat_Vignette/Melting_Curves/meltCurve_Q0K692.pdf]

# Q0K693

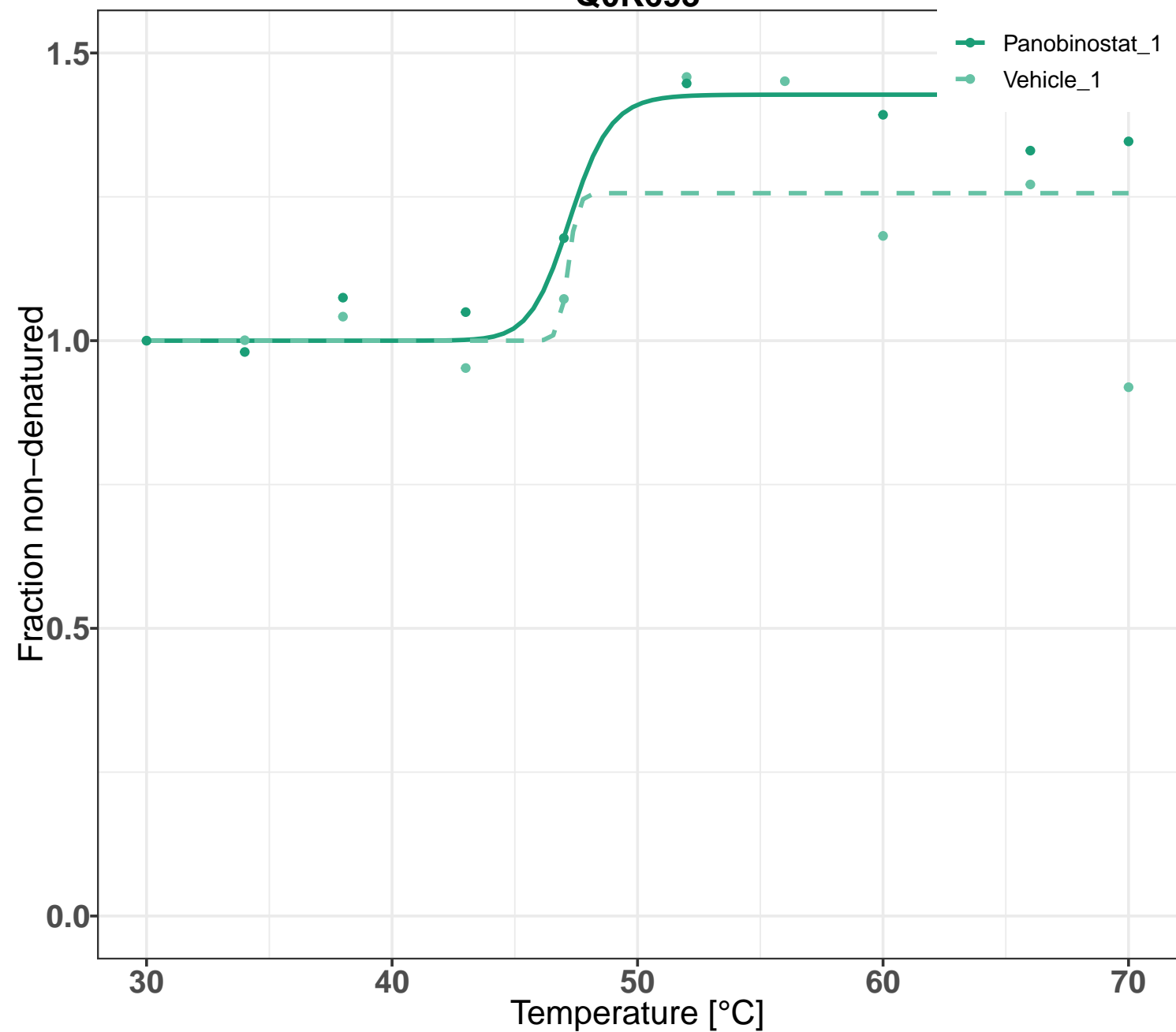

|                | meltPoint | slope | plateau | R2   |
|----------------|-----------|-------|---------|------|
| Panobinostat_1 | –         | 0.13  | 1.43    | 0.85 |
| Vehicle_1      | –         | 0.34  | 1.26    | 0.43 |

Supplement: Supplementary file 2 — Supplementary Material 2 [file 41598_2026_35990_MOESM2_ESM.zip › AllTheTPPData/D40vD86/Panobinostat_Vignette/Melting_Curves/meltCurve_Q0K693.pdf]

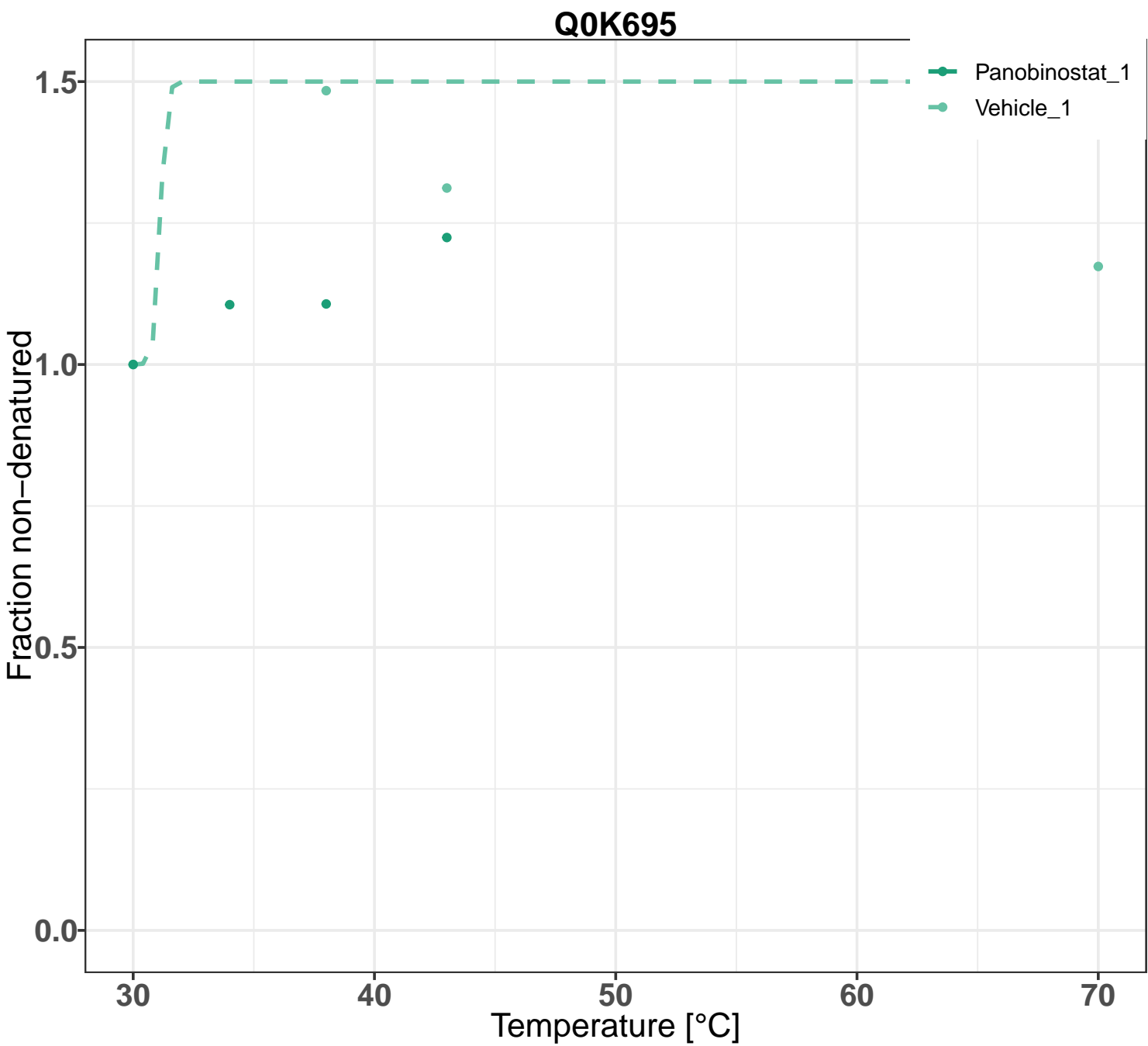

|                | meltPoint | slope | plateau | R2    |
|----------------|-----------|-------|---------|-------|
| Panobinostat_1 | –         | –     | –       | –     |
| Vehicle_1      | –         | 1     | 1.5     | –0.49 |

Supplement: Supplementary file 2 — Supplementary Material 2 [file 41598_2026_35990_MOESM2_ESM.zip › AllTheTPPData/D40vD86/Panobinostat_Vignette/Melting_Curves/meltCurve_Q0K695.pdf]

# Q0K6A5

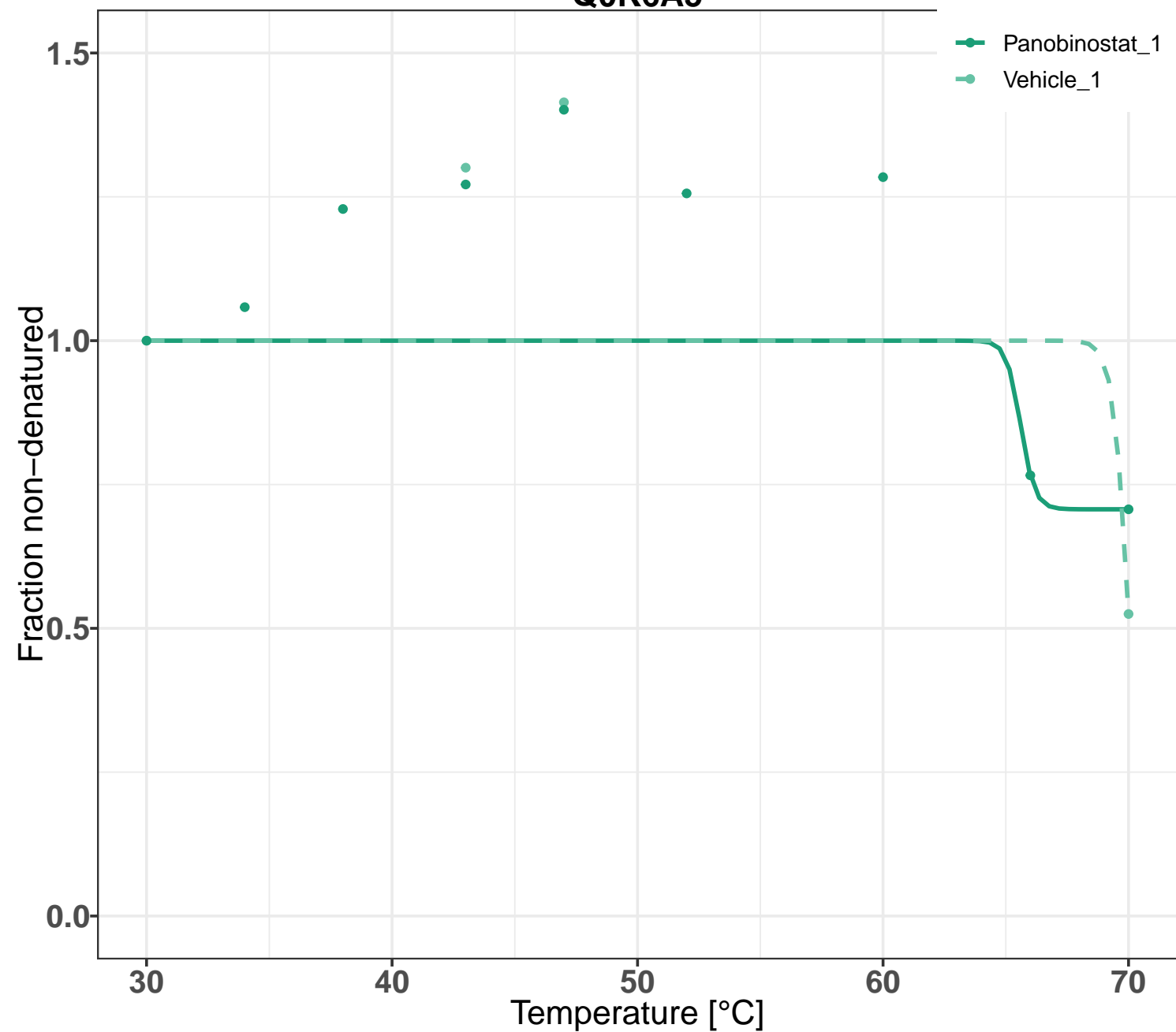

meltPoint

slope

plateau

R2

Panobinostat\_1

–

–0.26

0.71

–0.13

Vehicle\_1

–

–

0

–1.1

Supplement: Supplementary file 2 — Supplementary Material 2 [file 41598_2026_35990_MOESM2_ESM.zip › AllTheTPPData/D40vD86/Panobinostat_Vignette/Melting_Curves/meltCurve_Q0K6A5.pdf]

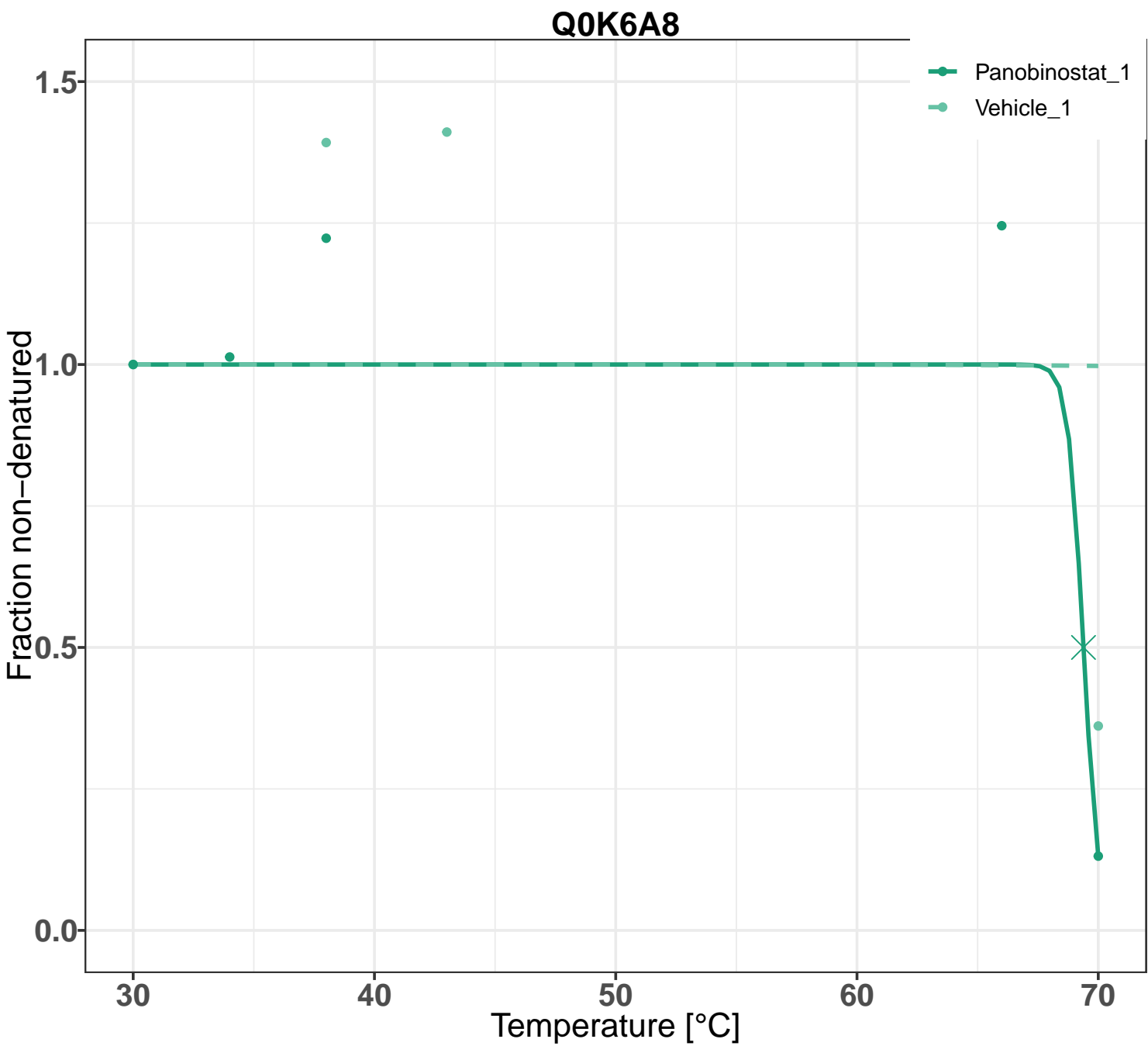

|                | meltPoint | slope | plateau | R2    |
|----------------|-----------|-------|---------|-------|
| Panobinostat_1 | 69.39     | -0.78 | 0       | -0.46 |
| Vehicle_1      | -         | -     | 0       | -0.83 |

Supplement: Supplementary file 2 — Supplementary Material 2 [file 41598_2026_35990_MOESM2_ESM.zip › AllTheTPPData/D40vD86/Panobinostat_Vignette/Melting_Curves/meltCurve_Q0K6A8.pdf]

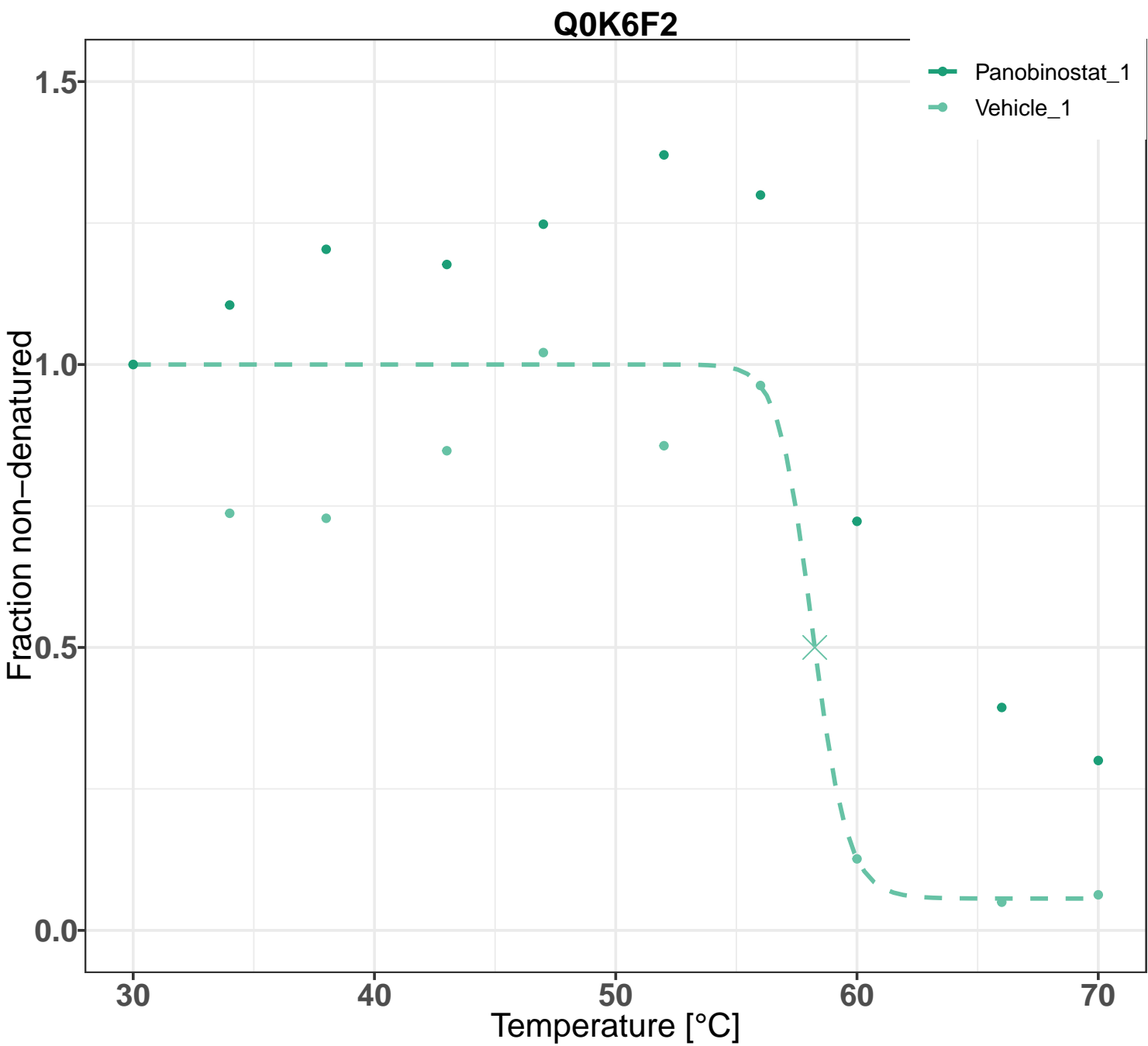

|                | meltPoint | slope | plateau | R2   |
|----------------|-----------|-------|---------|------|
| Panobinostat_1 | -         | -     | -       | -    |
| Vehicle_1      | 58.25     | -0.33 | 0.06    | 0.87 |

Supplement: Supplementary file 2 — Supplementary Material 2 [file 41598_2026_35990_MOESM2_ESM.zip › AllTheTPPData/D40vD86/Panobinostat_Vignette/Melting_Curves/meltCurve_Q0K6F2.pdf]

# Q0K6H2

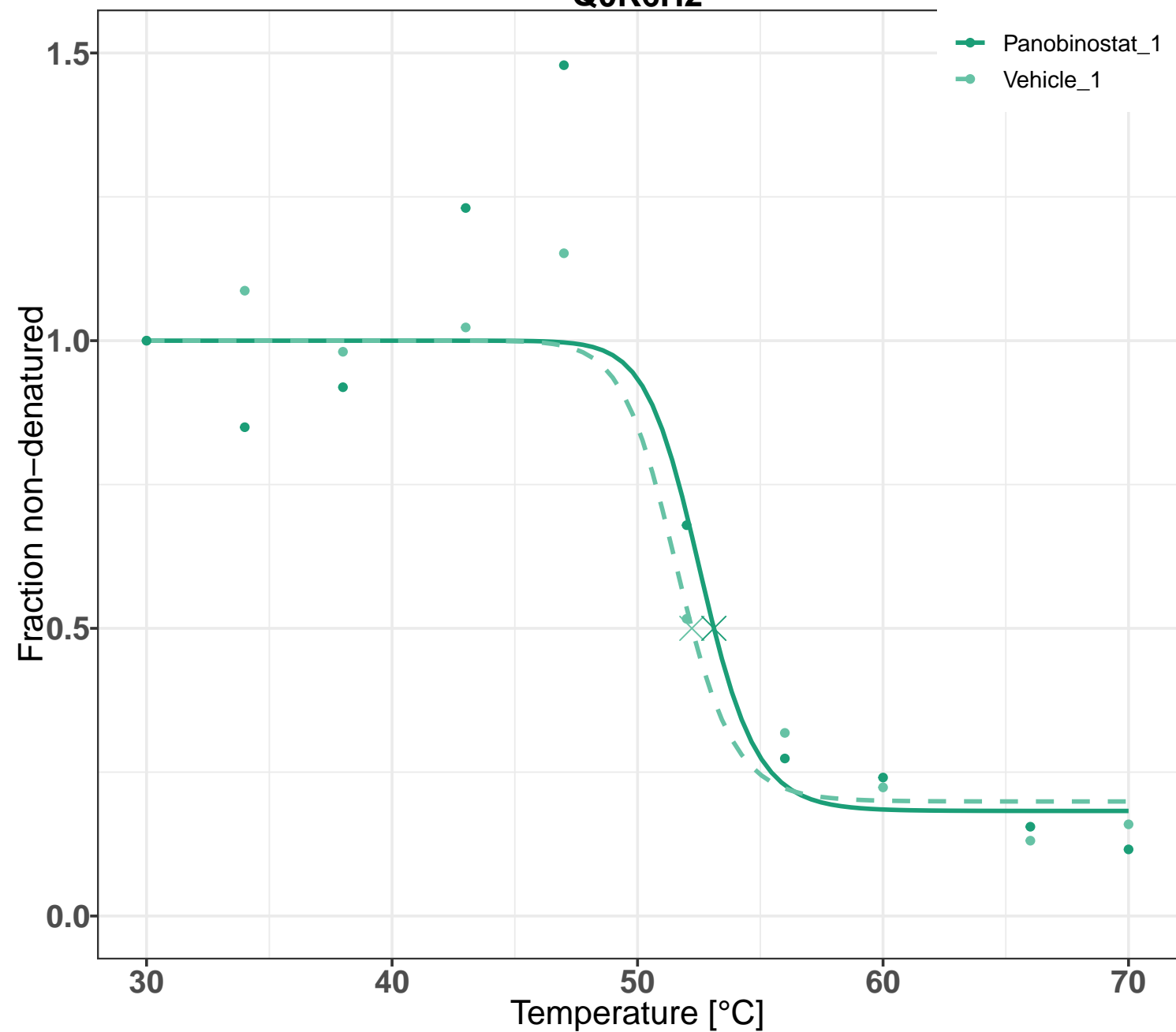

meltPoint

slope

plateau

R2

Panobinostat\_1

53.11

-0.18

0.18

0.84

Vehicle\_1

52.21

-0.18

0.2

0.97

Supplement: Supplementary file 2 — Supplementary Material 2 [file 41598_2026_35990_MOESM2_ESM.zip › AllTheTPPData/D40vD86/Panobinostat_Vignette/Melting_Curves/meltCurve_Q0K6H2.pdf]

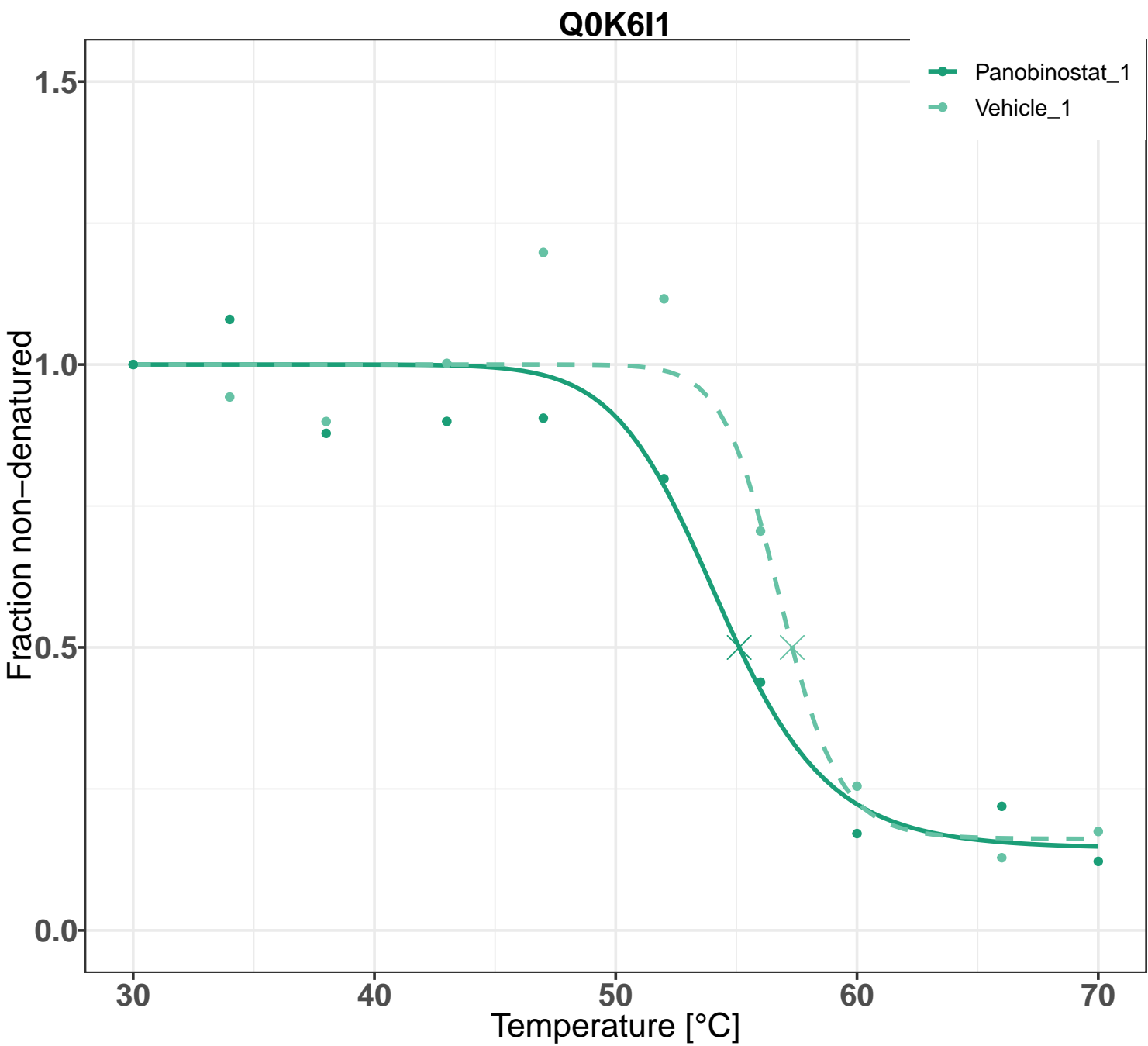

|                | meltPoint | slope  | plateau | R2   |
|----------------|-----------|--------|---------|------|
| Panobinostat_1 | 55.12     | -0.096 | 0.14    | 0.96 |
| Vehicle_1      | 57.31     | -0.17  | 0.16    | 0.95 |

Supplement: Supplementary file 2 — Supplementary Material 2 [file 41598_2026_35990_MOESM2_ESM.zip › AllTheTPPData/D40vD86/Panobinostat_Vignette/Melting_Curves/meltCurve_Q0K6I1.pdf]

# Q0K6L6

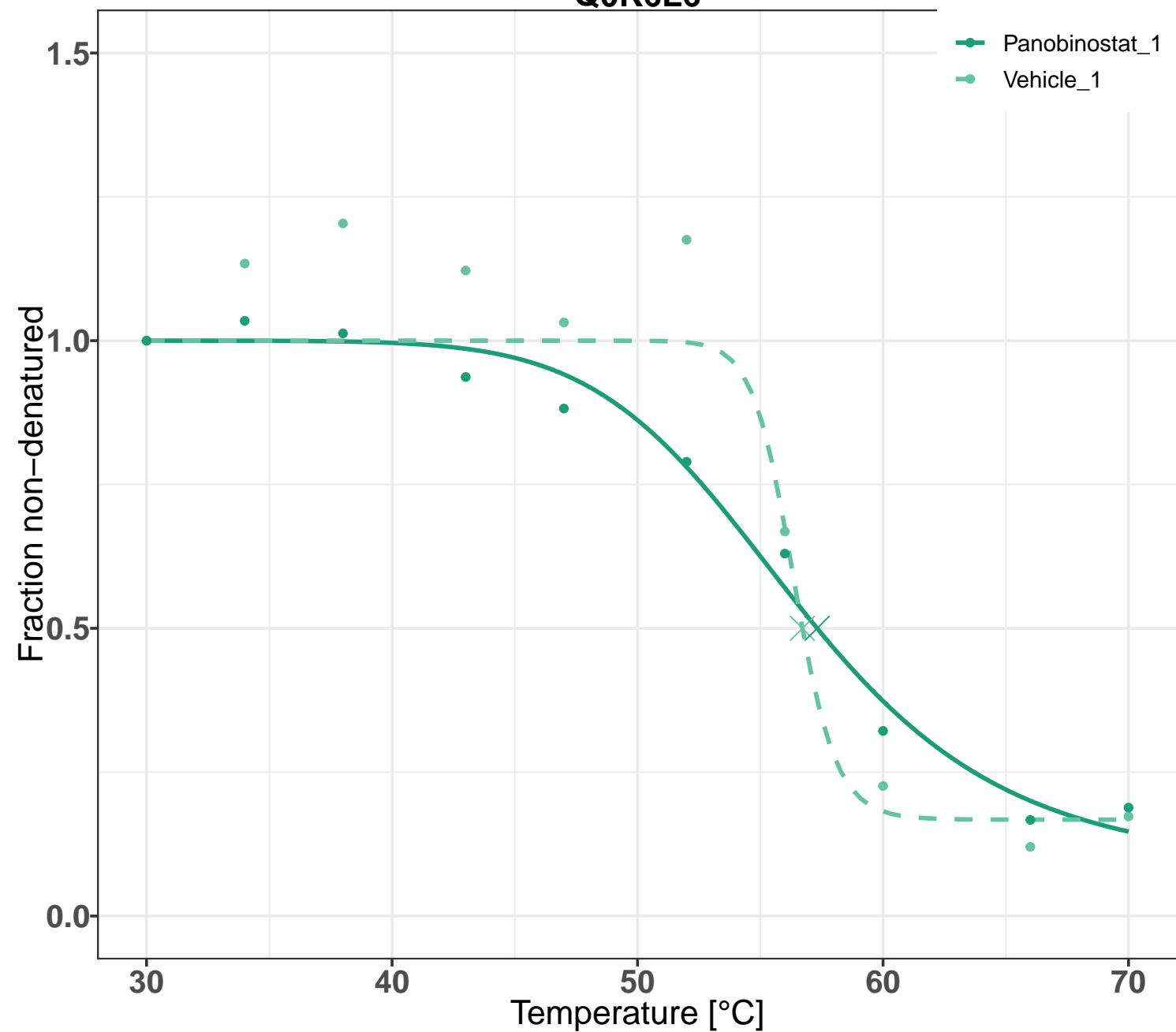

meltPoint

slope

plateau

R2

Panobinostat\_1

57.32

-0.055

0.08

0.98

Vehicle\_1

56.72

-0.25

0.17

0.94

Supplement: Supplementary file 2 — Supplementary Material 2 [file 41598_2026_35990_MOESM2_ESM.zip › AllTheTPPData/D40vD86/Panobinostat_Vignette/Melting_Curves/meltCurve_Q0K6L6.pdf]

# Q0K6M0

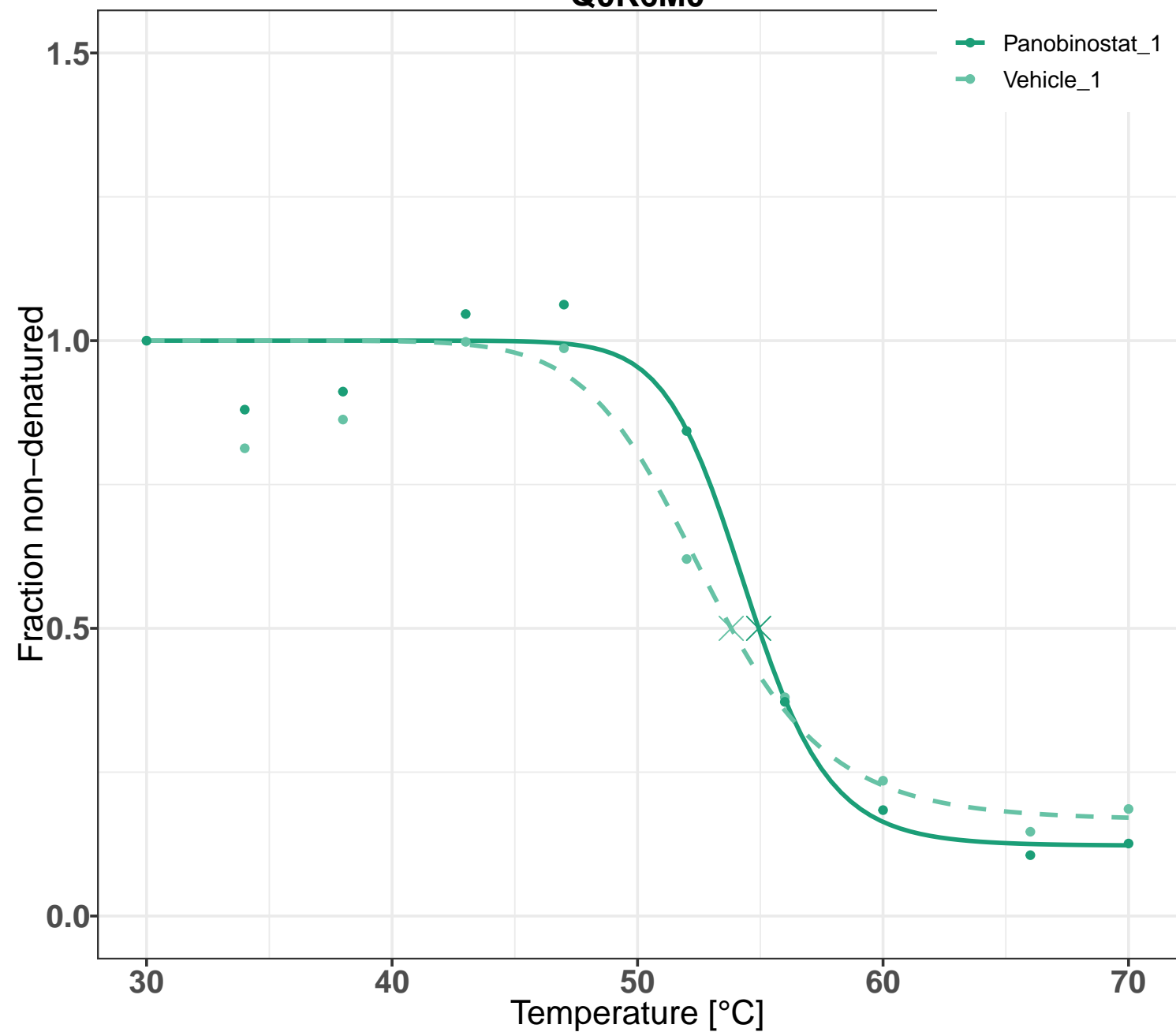

meltPoint

slope

plateau

R2

Panobinostat\_1

54.93

-0.13

0.12

0.98

Vehicle\_1

53.81

-0.084

0.17

0.95

Supplement: Supplementary file 2 — Supplementary Material 2 [file 41598_2026_35990_MOESM2_ESM.zip › AllTheTPPData/D40vD86/Panobinostat_Vignette/Melting_Curves/meltCurve_Q0K6M0.pdf]

# Q0K6M2

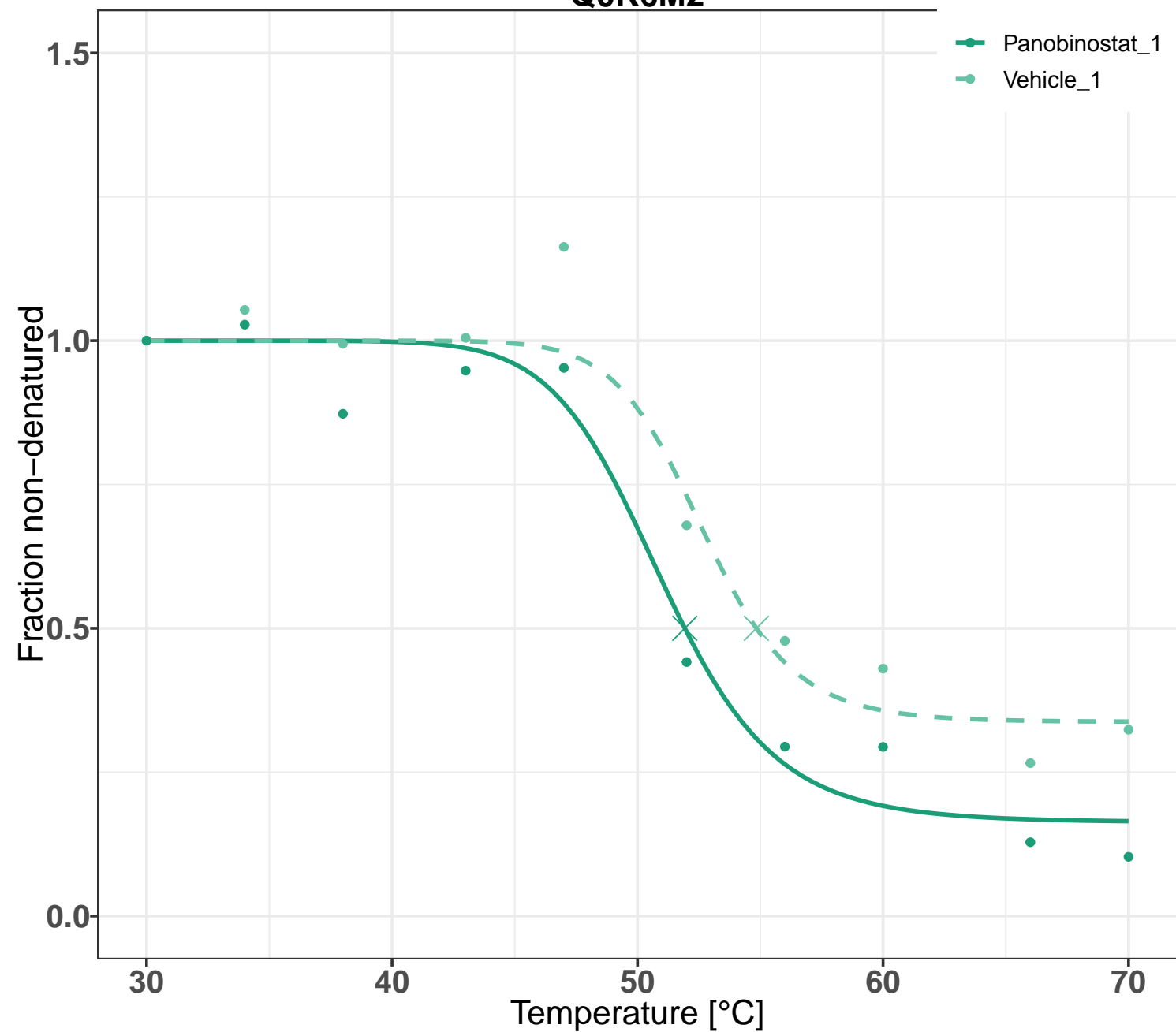

meltPoint

slope

plateau

R2

Panobinostat\_1

51.93

-0.092

0.16

0.97

Vehicle\_1

54.84

-0.09

0.34

0.95

Supplement: Supplementary file 2 — Supplementary Material 2 [file 41598_2026_35990_MOESM2_ESM.zip › AllTheTPPData/D40vD86/Panobinostat_Vignette/Melting_Curves/meltCurve_Q0K6M2.pdf]

# Q0K6M5

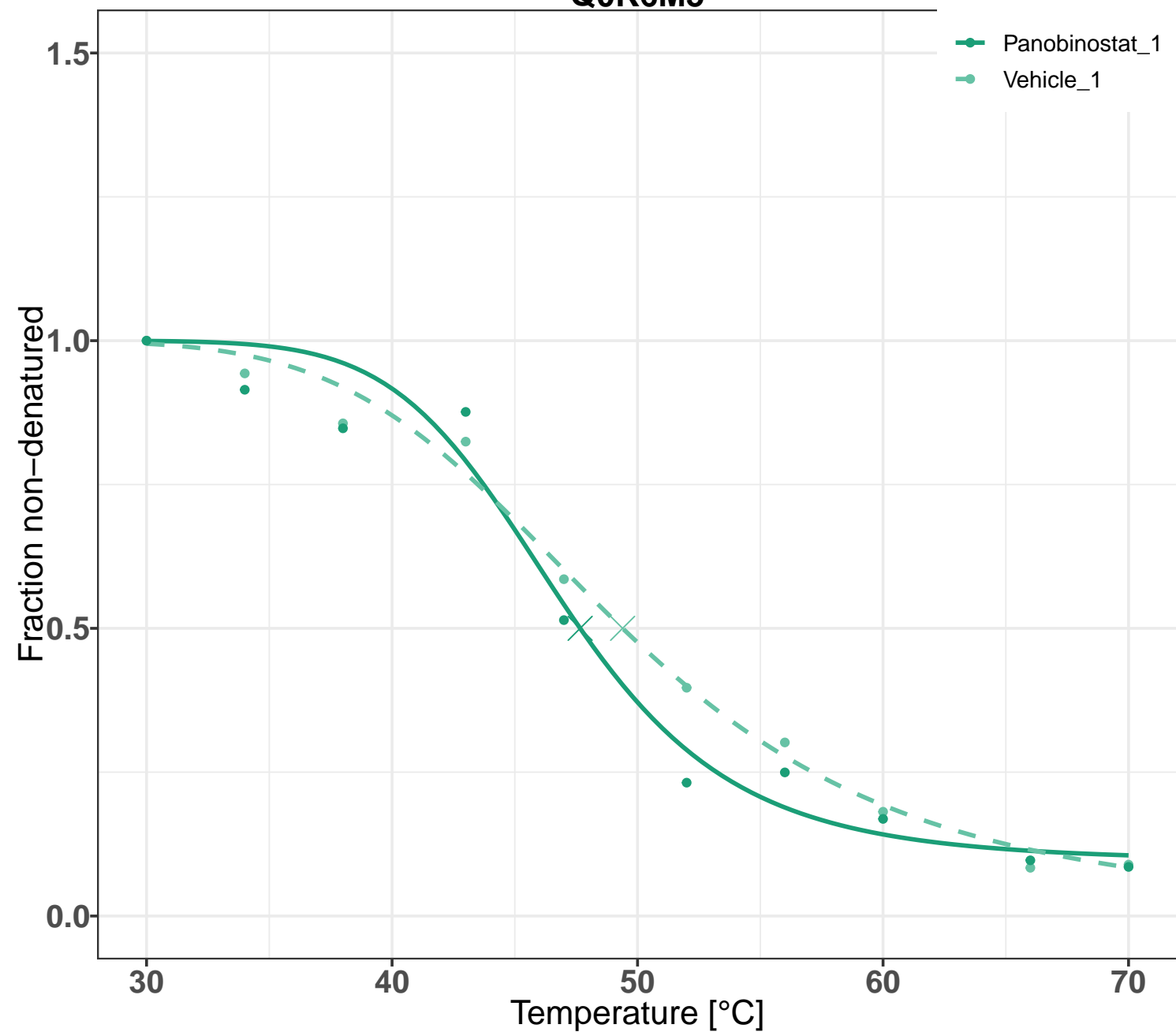

meltPoint

slope

plateau

R2

Panobinostat\_1

47.66

-0.065

0.09

0.97

Vehicle\_1

49.39

-0.043

0

0.99

Supplement: Supplementary file 2 — Supplementary Material 2 [file 41598_2026_35990_MOESM2_ESM.zip › AllTheTPPData/D40vD86/Panobinostat_Vignette/Melting_Curves/meltCurve_Q0K6M5.pdf]

# Q0K6M6

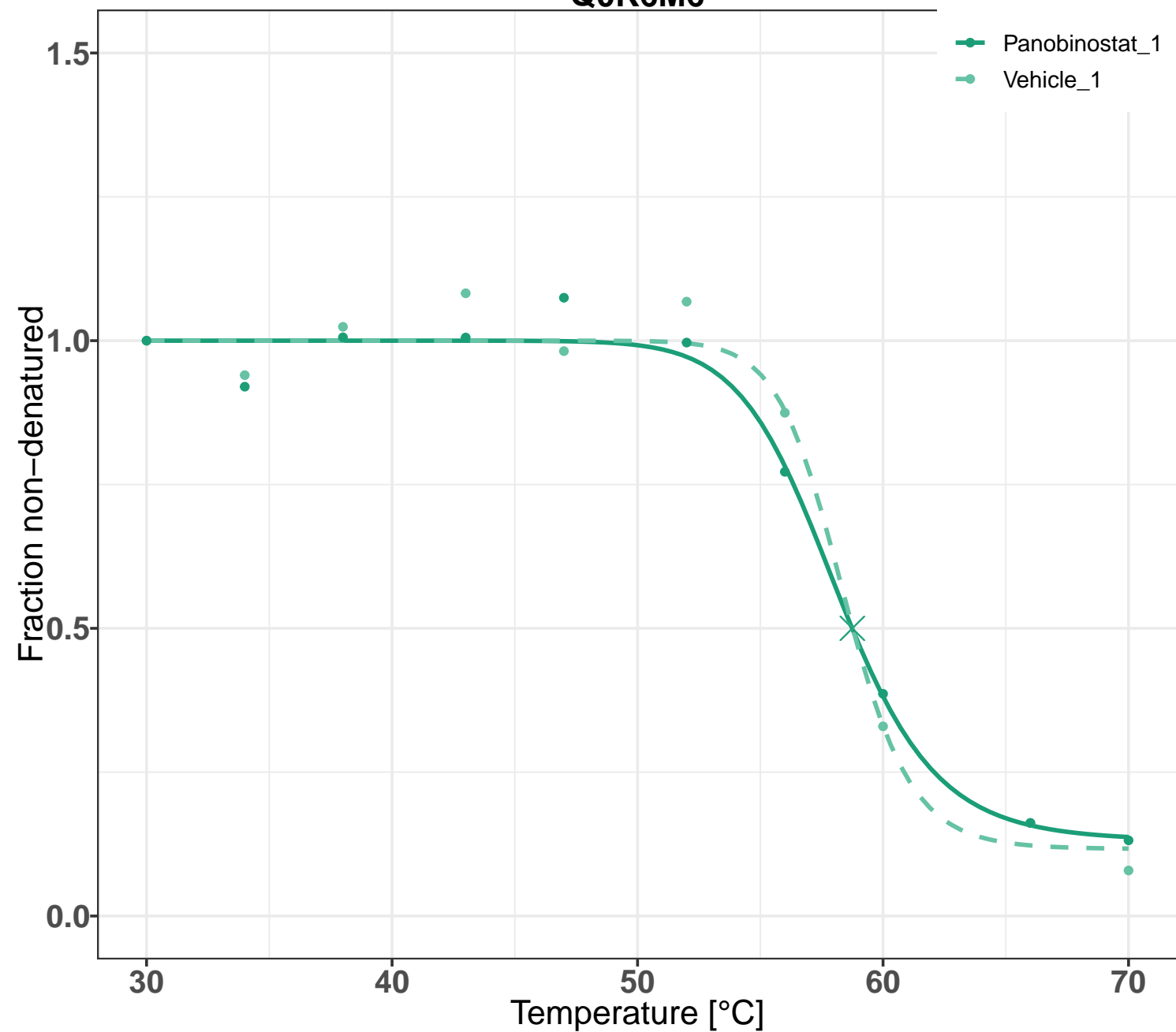

meltPoint

slope

plateau

R2

Panobinostat\_1

58.74

-0.11

0.13

0.99

Vehicle\_1

58.77

-0.16

0.12

0.99

Supplement: Supplementary file 2 — Supplementary Material 2 [file 41598_2026_35990_MOESM2_ESM.zip › AllTheTPPData/D40vD86/Panobinostat_Vignette/Melting_Curves/meltCurve_Q0K6M6.pdf]

# Q0K6M8

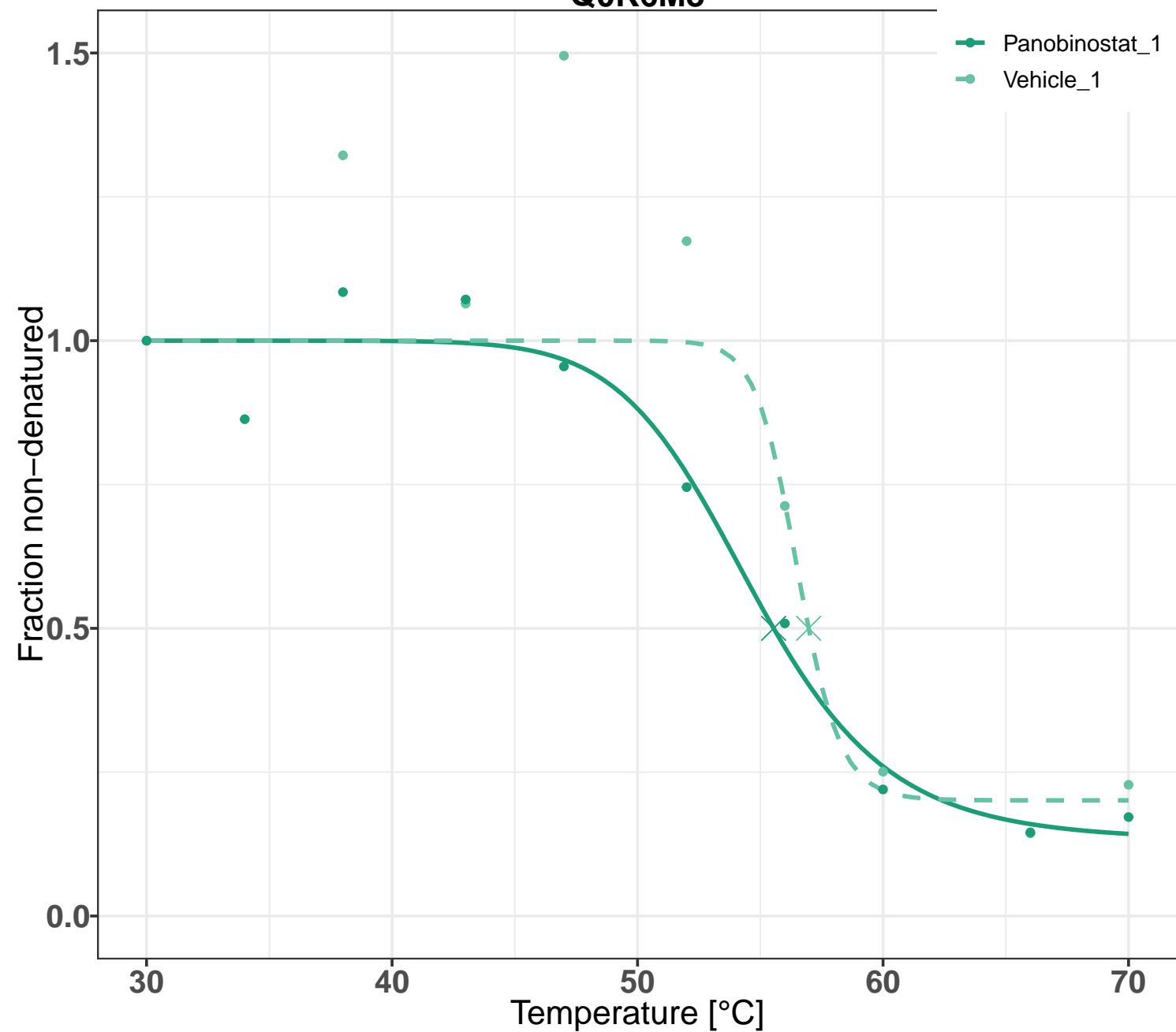

meltPoint

slope

plateau

R2

Panobinostat\_1

55.54

-0.079

0.13

0.97

Vehicle\_1

56.97

-0.23

0.2

0.74

Supplement: Supplementary file 2 — Supplementary Material 2 [file 41598_2026_35990_MOESM2_ESM.zip › AllTheTPPData/D40vD86/Panobinostat_Vignette/Melting_Curves/meltCurve_Q0K6M8.pdf]

# Q0K6N0

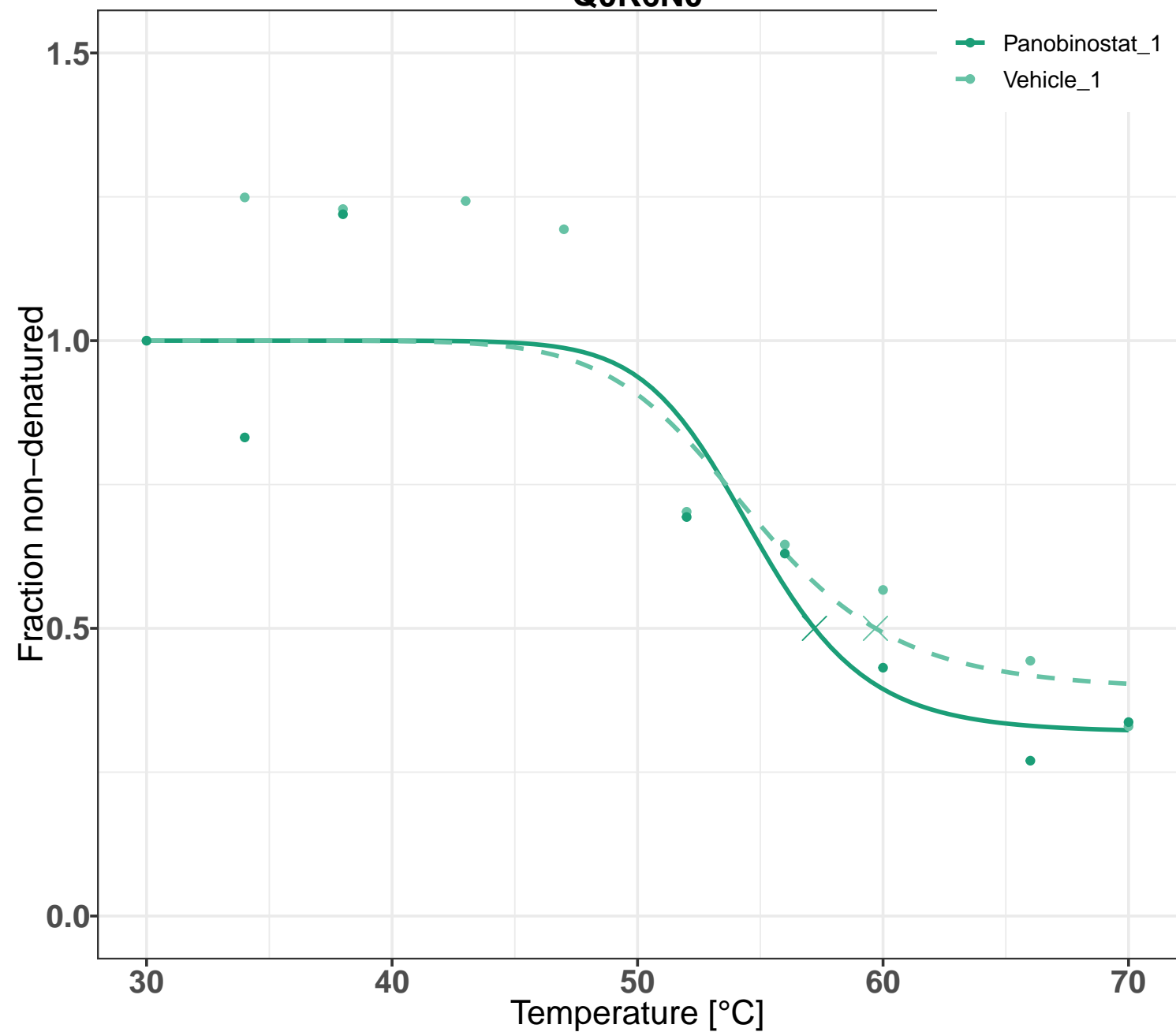

**meltPoint**

**slope**

**plateau**

**R2**

**Panobinostat\_1**

**57.2**

**-0.075**

**0.32**

**0.59**

**Vehicle\_1**

**59.69**

**-0.051**

**0.39**

**0.78**

Supplement: Supplementary file 2 — Supplementary Material 2 [file 41598_2026_35990_MOESM2_ESM.zip › AllTheTPPData/D40vD86/Panobinostat_Vignette/Melting_Curves/meltCurve_Q0K6N0.pdf]

# Q0K6N3

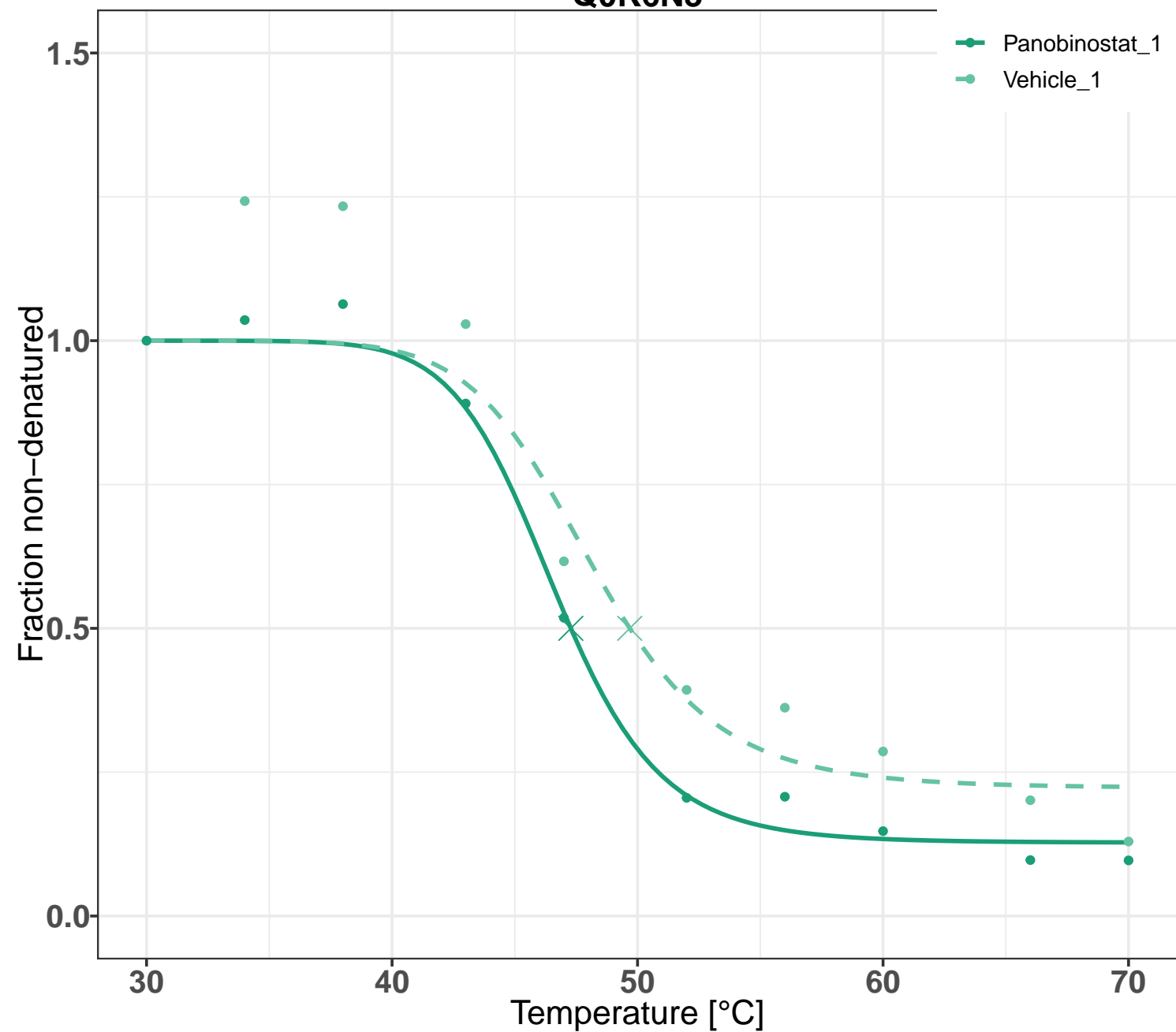

meltPoint

slope

plateau

R2

Panobinostat\_1

47.28

-0.1

0.13

0.99

Vehicle\_1

49.68

-0.077

0.22

0.91

Supplement: Supplementary file 2 — Supplementary Material 2 [file 41598_2026_35990_MOESM2_ESM.zip › AllTheTPPData/D40vD86/Panobinostat_Vignette/Melting_Curves/meltCurve_Q0K6N3.pdf]

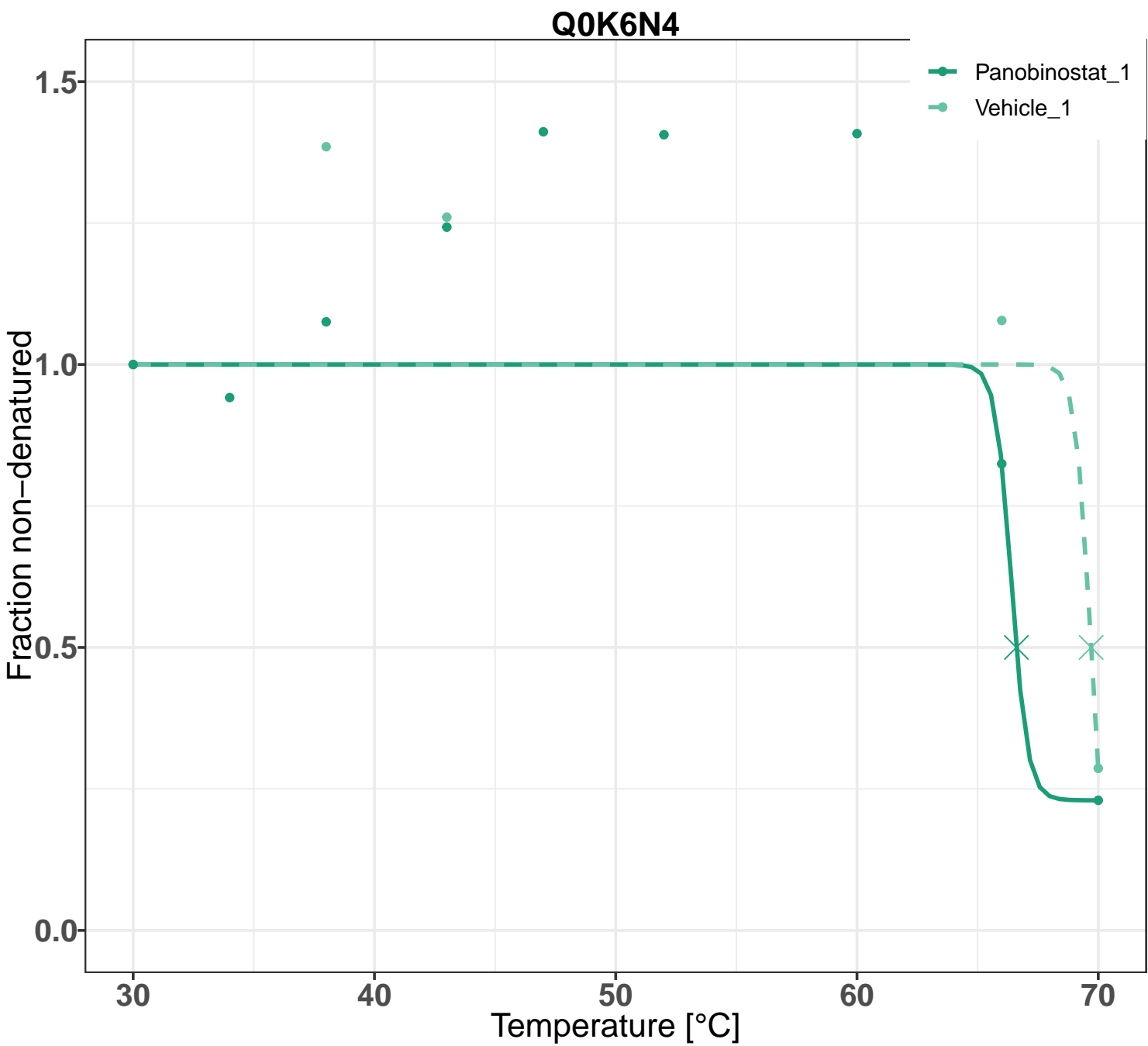

|                | meltPoint | slope | plateau | R2    |
|----------------|-----------|-------|---------|-------|
| Panobinostat_1 | 66.61     | -0.58 | 0.23    | 0.24  |
| Vehicle_1      | 69.7      | -0.77 | 0       | -0.56 |

Supplement: Supplementary file 2 — Supplementary Material 2 [file 41598_2026_35990_MOESM2_ESM.zip › AllTheTPPData/D40vD86/Panobinostat_Vignette/Melting_Curves/meltCurve_Q0K6N4.pdf]

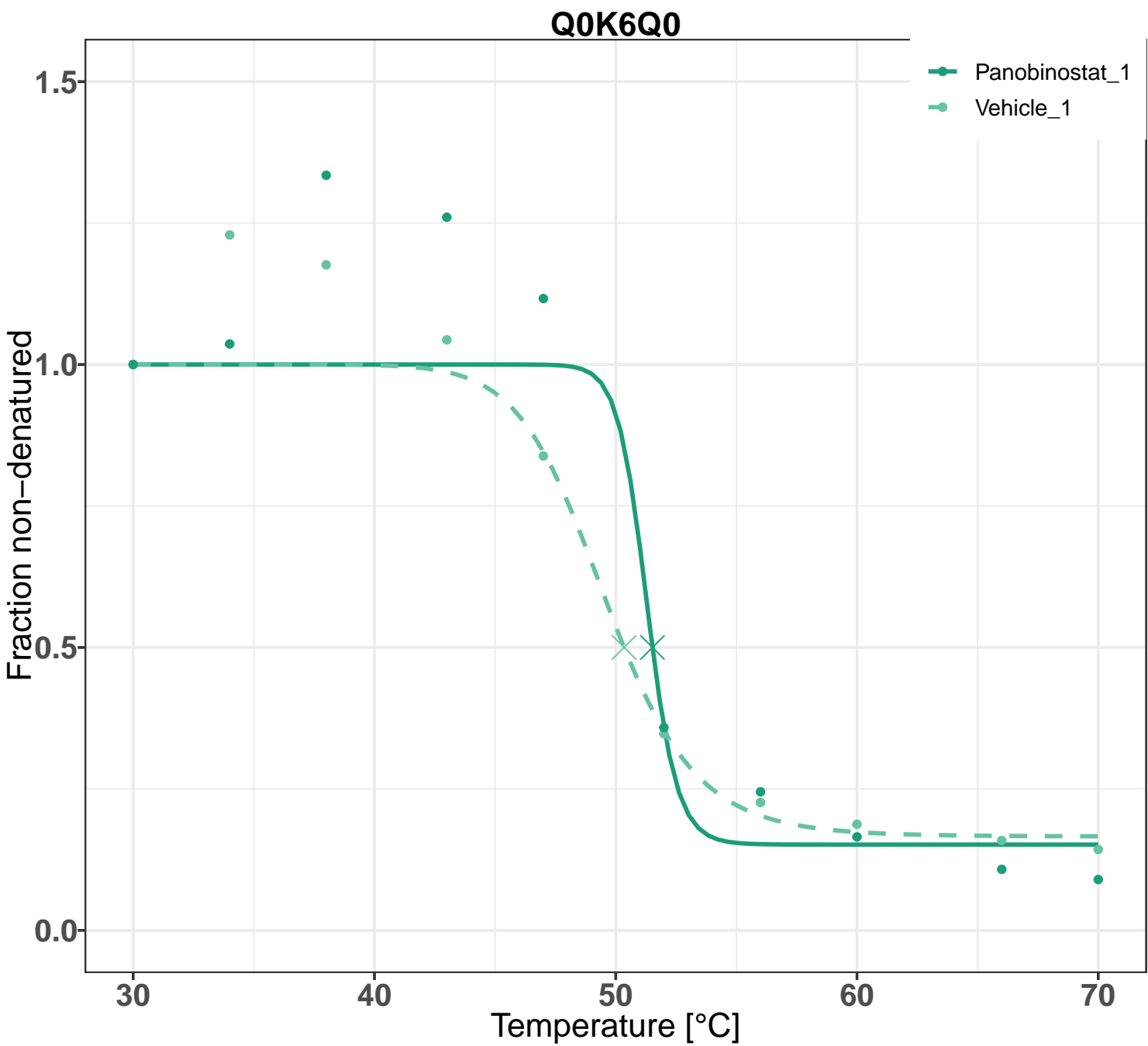

|                | meltPoint | slope | plateau | R2   |
|----------------|-----------|-------|---------|------|
| Panobinostat_1 | 51.52     | -0.35 | 0.15    | 0.91 |
| Vehicle_1      | 50.35     | -0.11 | 0.17    | 0.95 |

Supplement: Supplementary file 2 — Supplementary Material 2 [file 41598_2026_35990_MOESM2_ESM.zip › AllTheTPPData/D40vD86/Panobinostat_Vignette/Melting_Curves/meltCurve_Q0K6Q0.pdf]

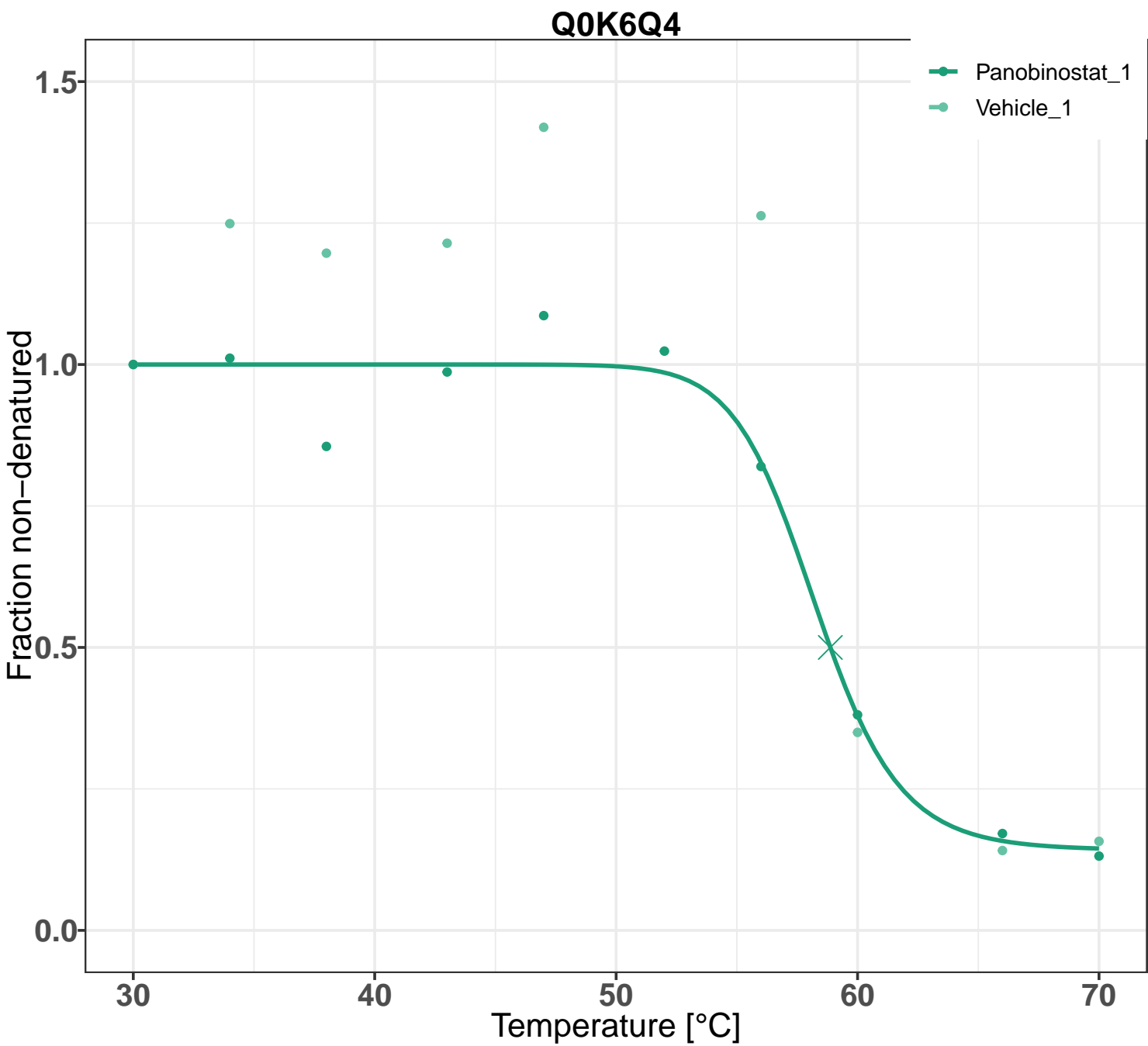

|                | meltPoint | slope | plateau | R2   |
|----------------|-----------|-------|---------|------|
| Panobinostat_1 | 58.87     | -0.12 | 0.14    | 0.98 |
| Vehicle_1      | -         | -     | -       | -    |

Supplement: Supplementary file 2 — Supplementary Material 2 [file 41598_2026_35990_MOESM2_ESM.zip › AllTheTPPData/D40vD86/Panobinostat_Vignette/Melting_Curves/meltCurve_Q0K6Q4.pdf]

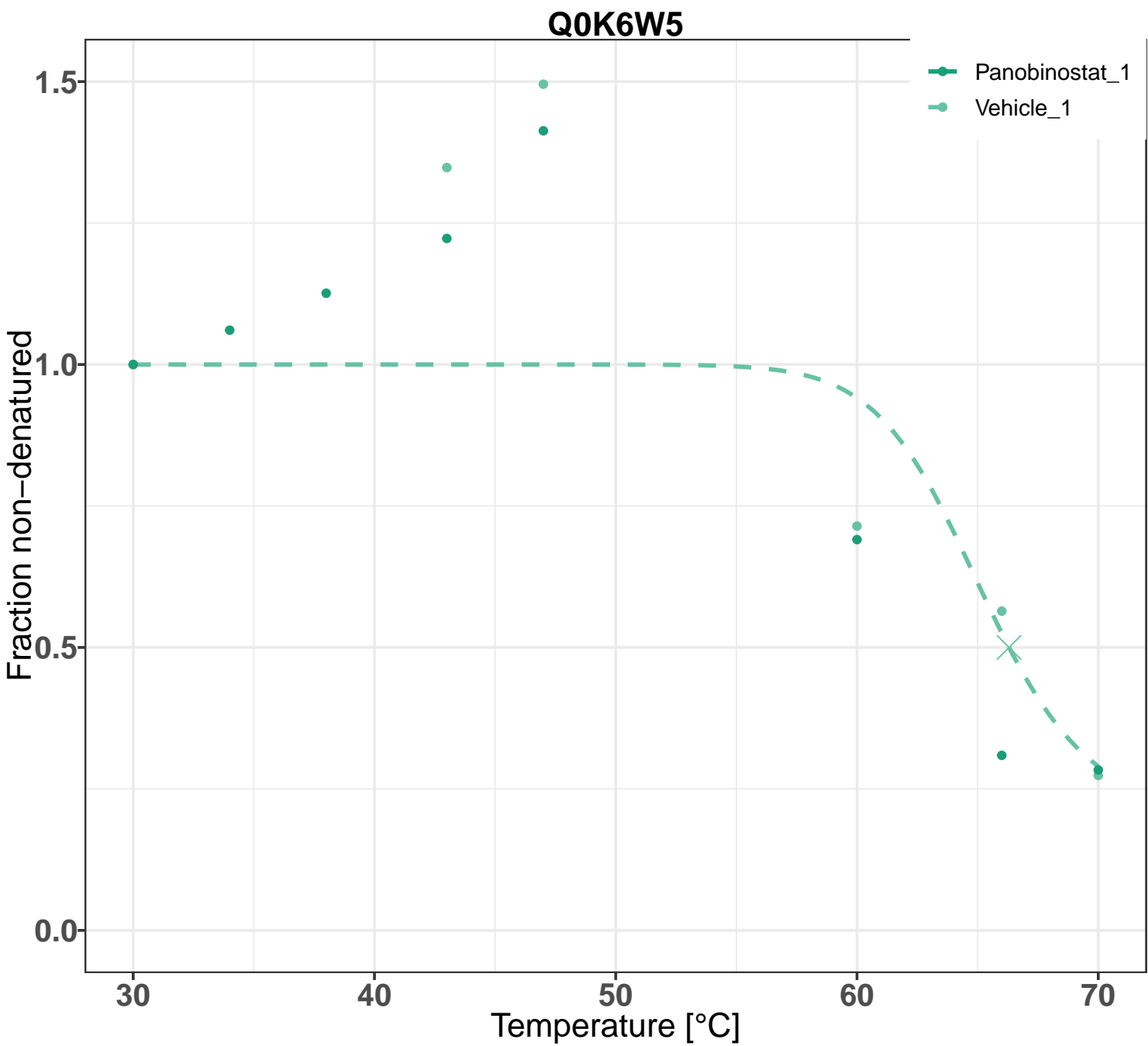

|                | meltPoint | slope  | plateau | R2    |
|----------------|-----------|--------|---------|-------|
| Panobinostat_1 | –         | –      | –       | –     |
| Vehicle_1      | 66.3      | -0.091 | 0.2     | -0.03 |

Supplement: Supplementary file 2 — Supplementary Material 2 [file 41598_2026_35990_MOESM2_ESM.zip › AllTheTPPData/D40vD86/Panobinostat_Vignette/Melting_Curves/meltCurve_Q0K6W5.pdf]

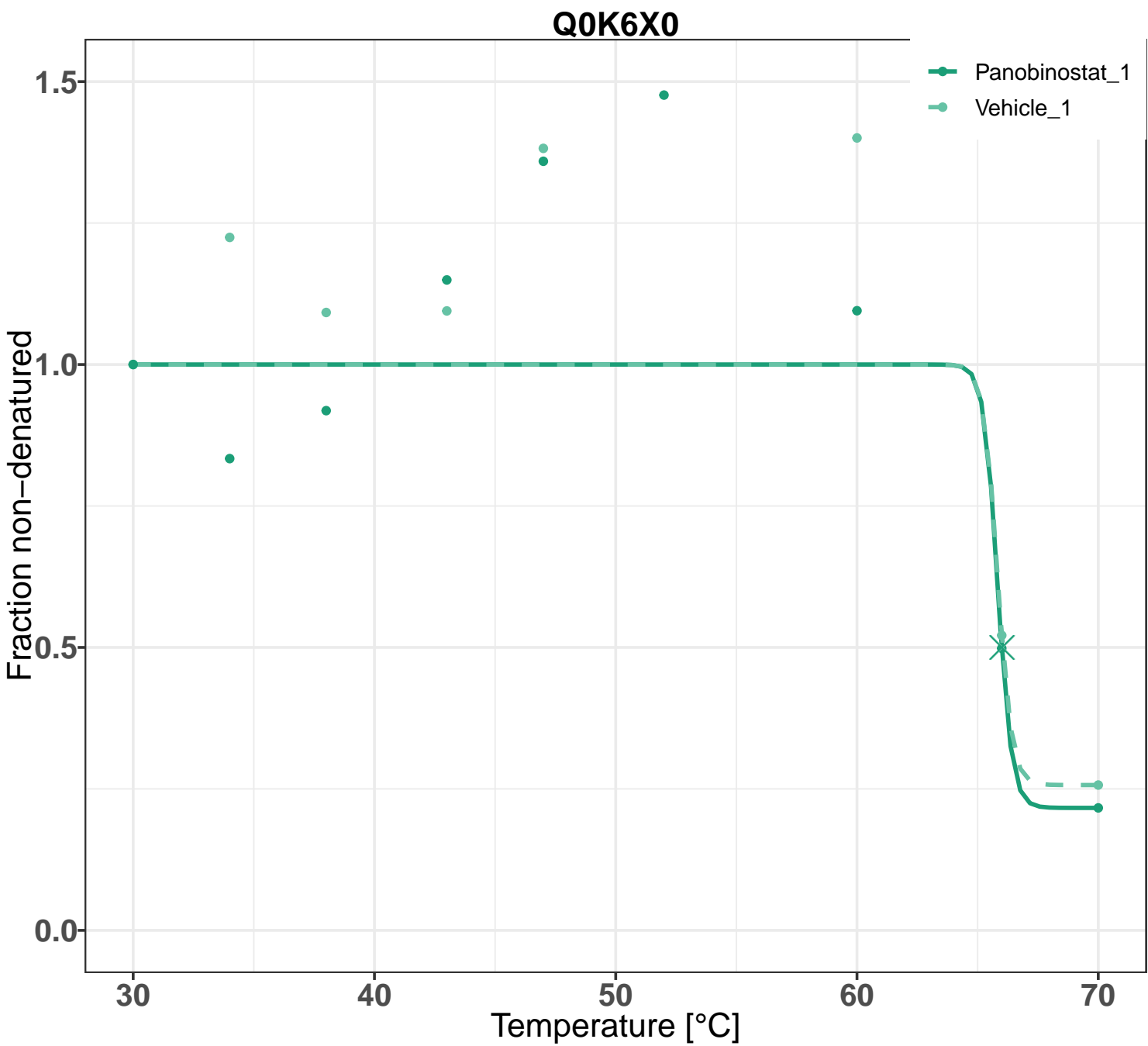

|                | meltPoint | slope | plateau | R2   |
|----------------|-----------|-------|---------|------|
| Panobinostat_1 | 66        | -0.68 | 0.22    | 0.55 |
| Vehicle_1      | 66.04     | -0.64 | 0.26    | 0.24 |

Supplement: Supplementary file 2 — Supplementary Material 2 [file 41598_2026_35990_MOESM2_ESM.zip › AllTheTPPData/D40vD86/Panobinostat_Vignette/Melting_Curves/meltCurve_Q0K6X0.pdf]

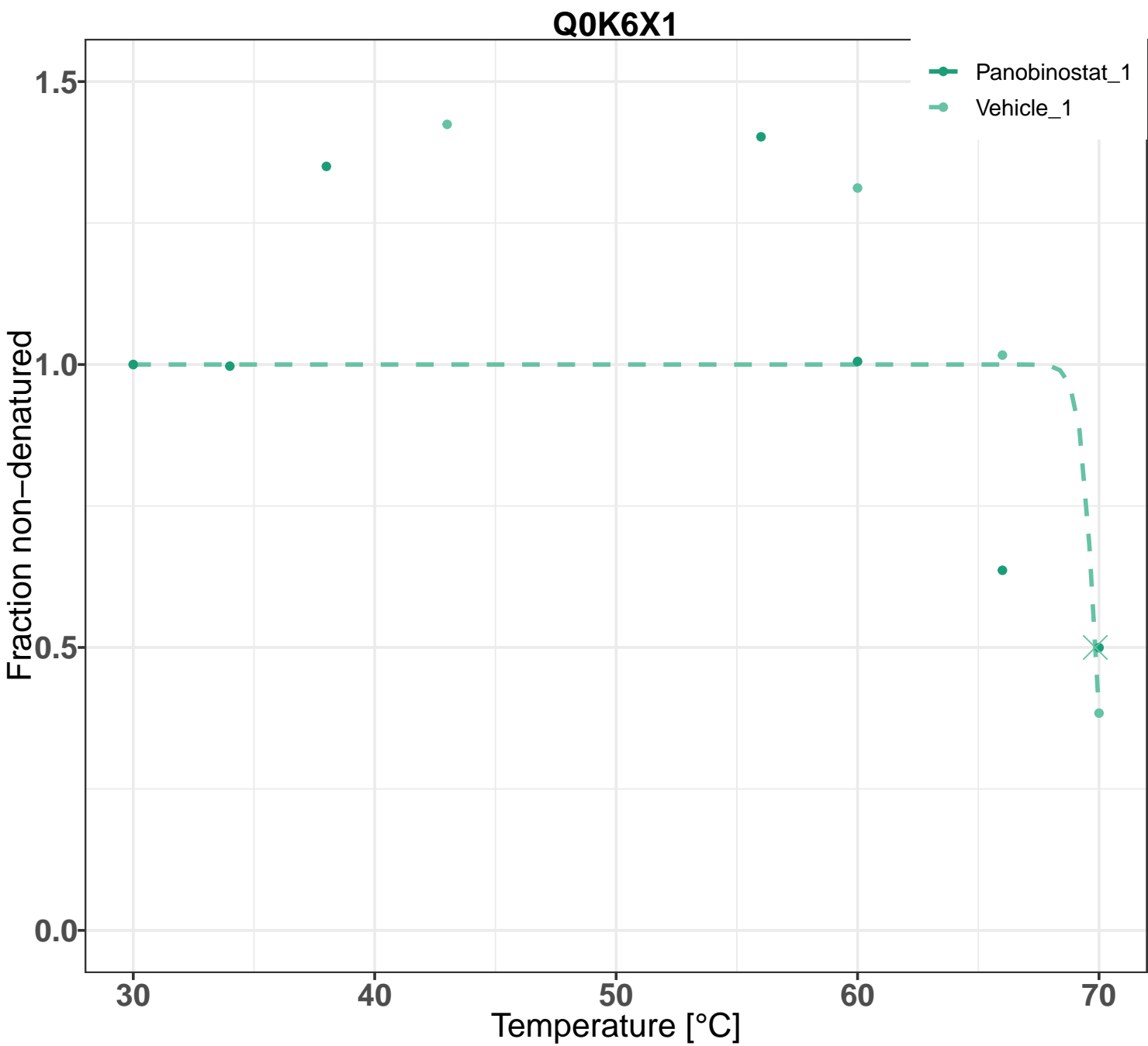

|                | meltPoint | slope | plateau | R2    |
|----------------|-----------|-------|---------|-------|
| Panobinostat_1 | –         | –     | –       | –     |
| Vehicle_1      | 69.85     | –0.77 | 0       | –0.62 |

Supplement: Supplementary file 2 — Supplementary Material 2 [file 41598_2026_35990_MOESM2_ESM.zip › AllTheTPPData/D40vD86/Panobinostat_Vignette/Melting_Curves/meltCurve_Q0K6X1.pdf]

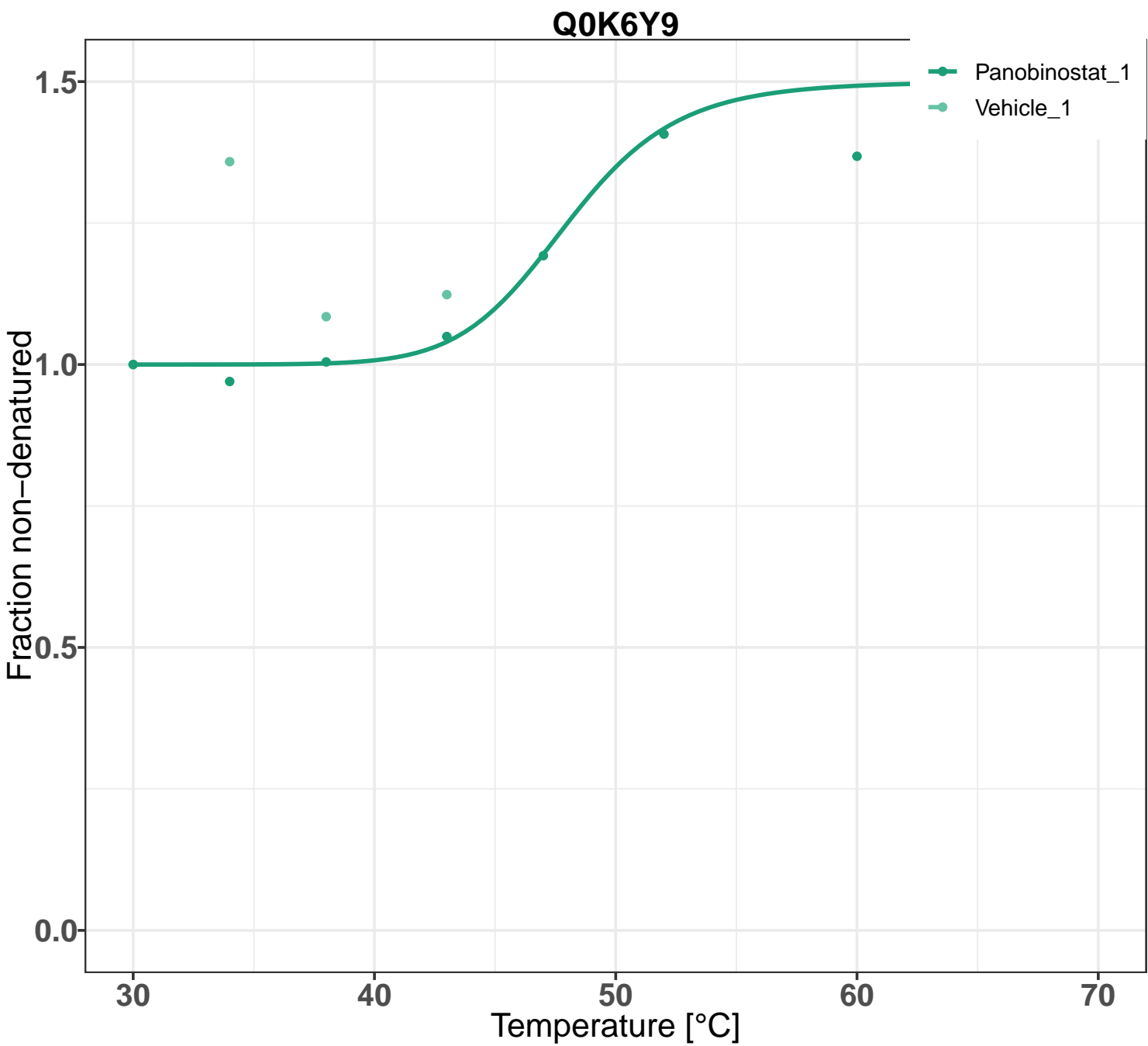

|                | meltPoint | slope | plateau | R2   |
|----------------|-----------|-------|---------|------|
| Panobinostat_1 | —         | 0.055 | 1.5     | 0.76 |
| Vehicle_1      | —         | —     | —       | —    |

Supplement: Supplementary file 2 — Supplementary Material 2 [file 41598_2026_35990_MOESM2_ESM.zip › AllTheTPPData/D40vD86/Panobinostat_Vignette/Melting_Curves/meltCurve_Q0K6Y9.pdf]

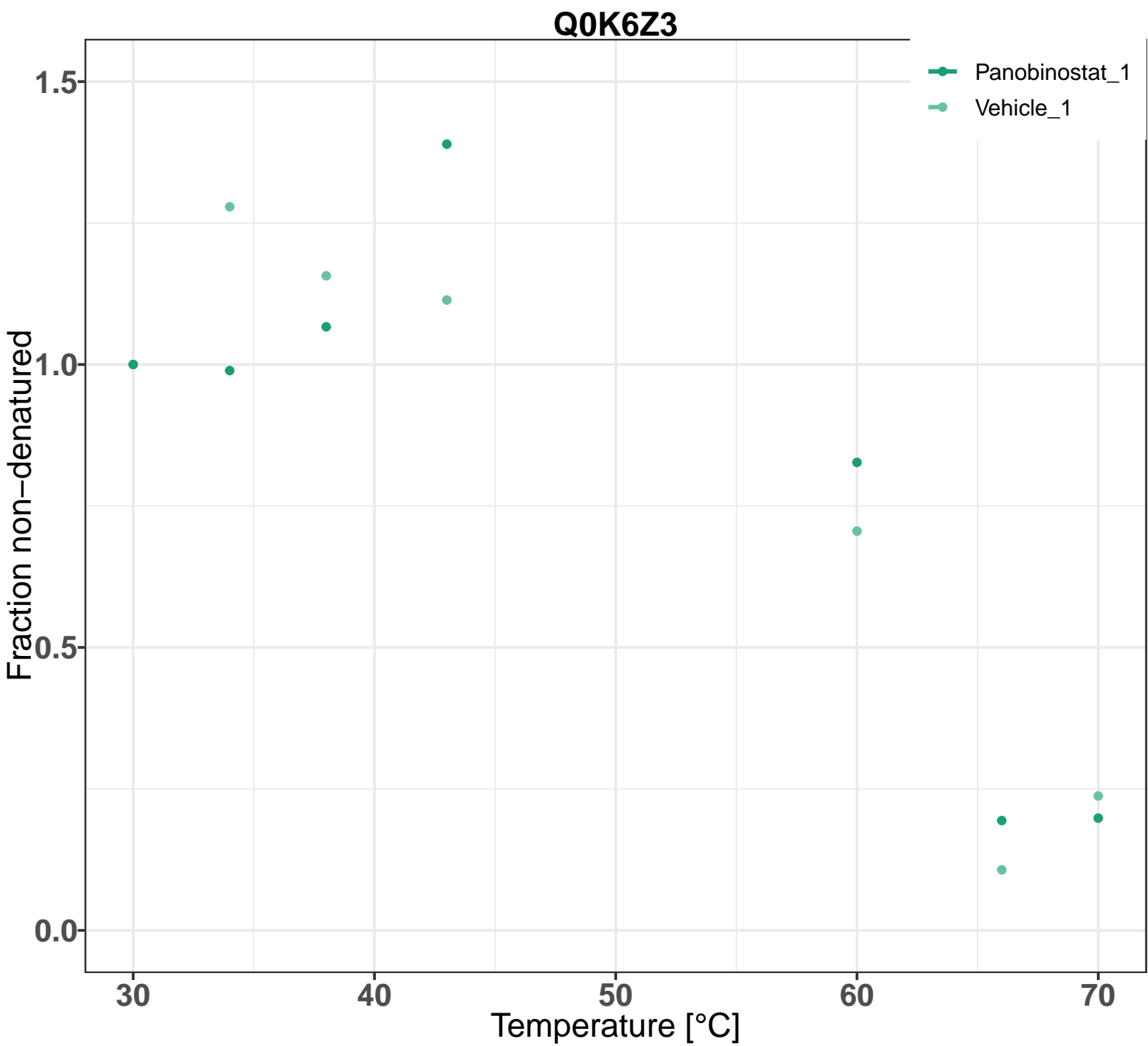

|                | meltPoint | slope | plateau | R2 |
|----------------|-----------|-------|---------|----|
| Panobinostat_1 | —         | —     | —       | —  |
| Vehicle_1      | —         | —     | —       | —  |

Supplement: Supplementary file 2 — Supplementary Material 2 [file 41598_2026_35990_MOESM2_ESM.zip › AllTheTPPData/D40vD86/Panobinostat_Vignette/Melting_Curves/meltCurve_Q0K6Z3.pdf]

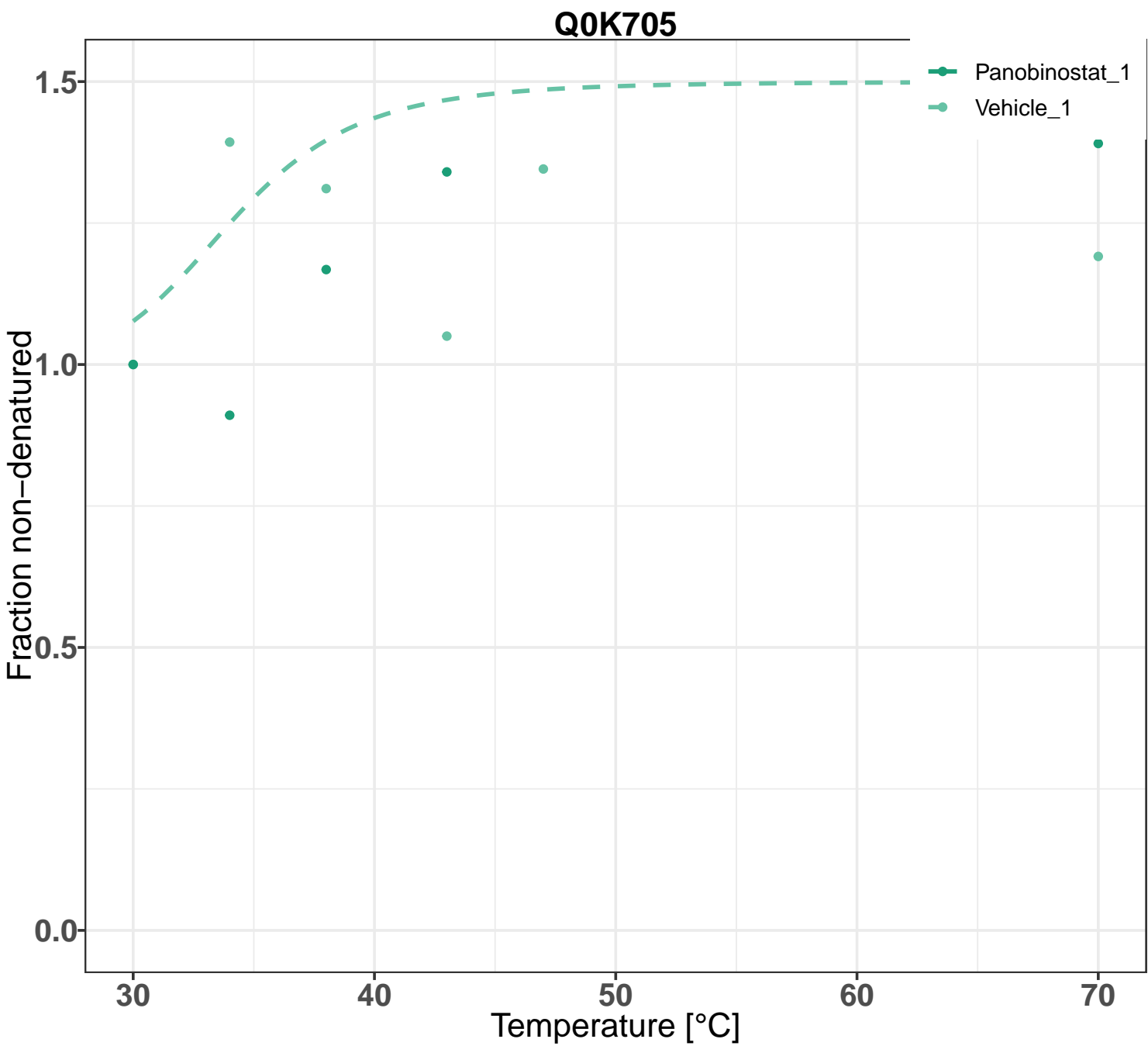

|                | meltPoint | slope | plateau | R2   |
|----------------|-----------|-------|---------|------|
| Panobinostat_1 | –         | –     | –       | –    |
| Vehicle_1      | –         | 0.048 | 1.5     | 0.06 |

Supplement: Supplementary file 2 — Supplementary Material 2 [file 41598_2026_35990_MOESM2_ESM.zip › AllTheTPPData/D40vD86/Panobinostat_Vignette/Melting_Curves/meltCurve_Q0K705.pdf]

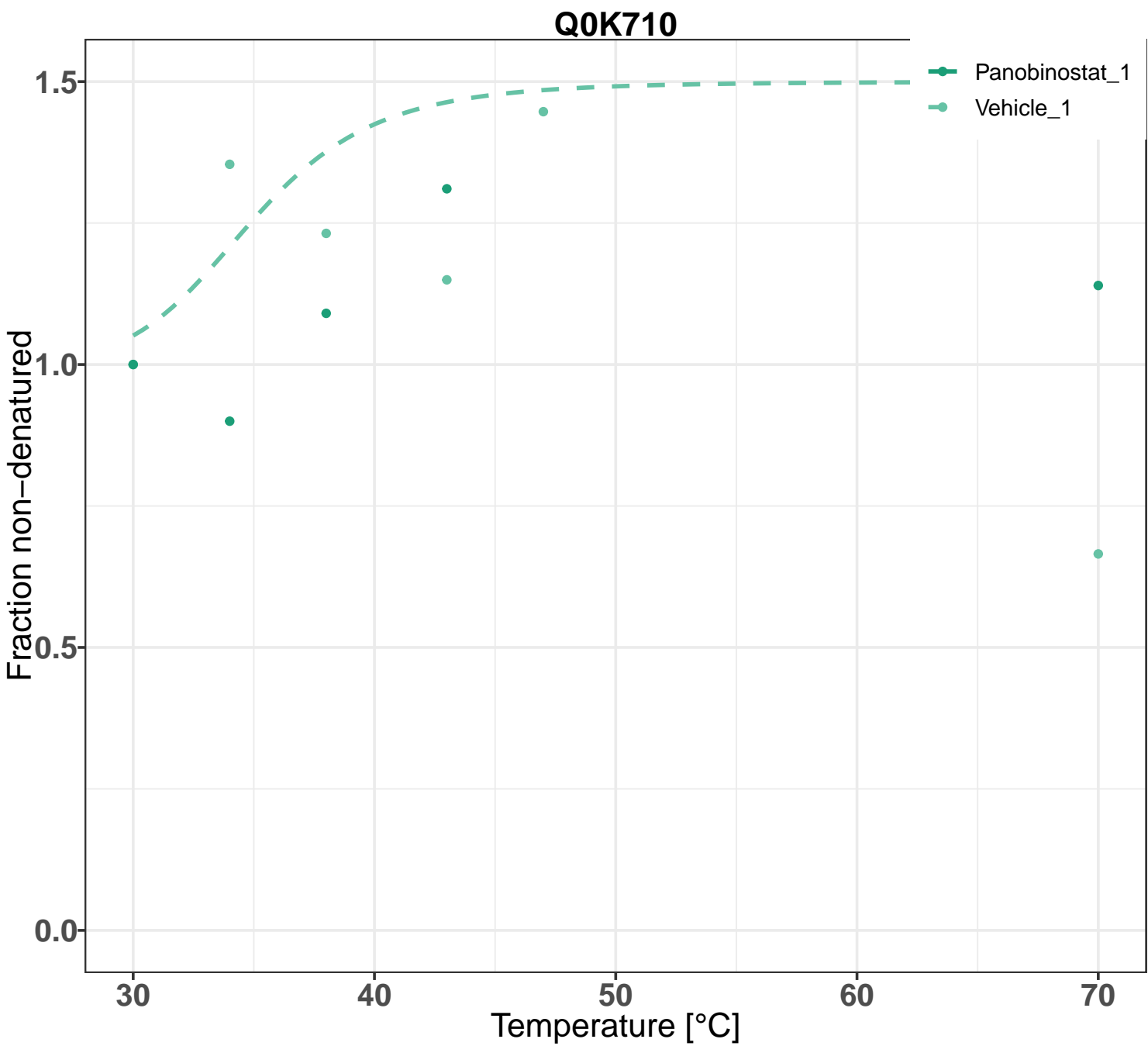

|                | meltPoint | slope | plateau | R2   |
|----------------|-----------|-------|---------|------|
| Panobinostat_1 | –         | –     | –       | –    |
| Vehicle_1      | –         | 0.049 | 1.5     | 0.05 |

Supplement: Supplementary file 2 — Supplementary Material 2 [file 41598_2026_35990_MOESM2_ESM.zip › AllTheTPPData/D40vD86/Panobinostat_Vignette/Melting_Curves/meltCurve_Q0K710.pdf]

# Q0K718

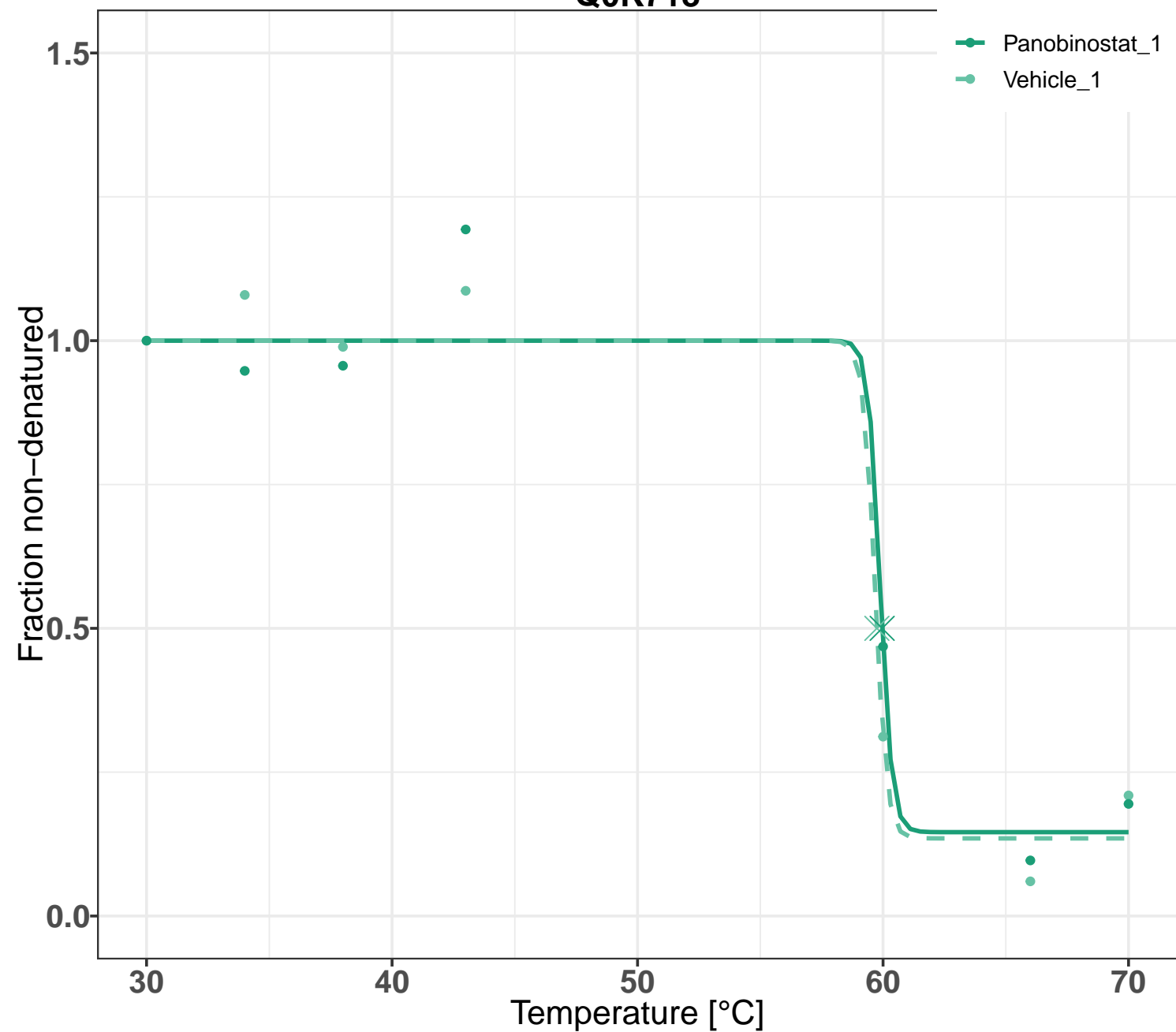

|                | meltPoint | slope | plateau | R2   |
|----------------|-----------|-------|---------|------|
| Panobinostat_1 | 59.96     | -0.89 | 0.15    | 0.61 |
| Vehicle_1      | 59.75     | -0.91 | 0.13    | 0.52 |

Supplement: Supplementary file 2 — Supplementary Material 2 [file 41598_2026_35990_MOESM2_ESM.zip › AllTheTPPData/D40vD86/Panobinostat_Vignette/Melting_Curves/meltCurve_Q0K718.pdf]

# Q0K721

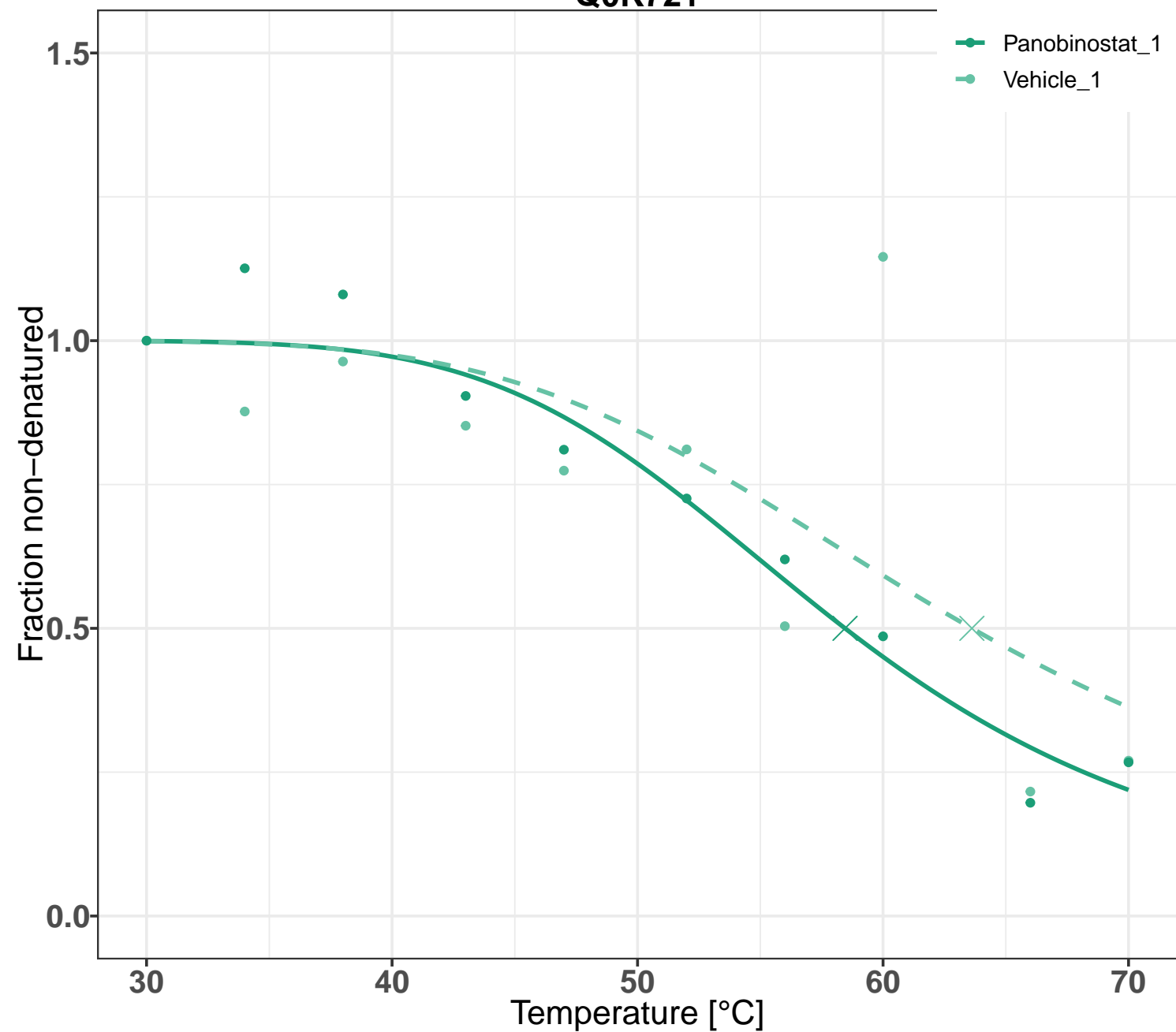

meltPoint

slope

plateau

R2

Panobinostat\_1

58.45

-0.035

0

0.95

Vehicle\_1

63.62

-0.027

0

0.49

Supplement: Supplementary file 2 — Supplementary Material 2 [file 41598_2026_35990_MOESM2_ESM.zip › AllTheTPPData/D40vD86/Panobinostat_Vignette/Melting_Curves/meltCurve_Q0K721.pdf]

# Q0K722

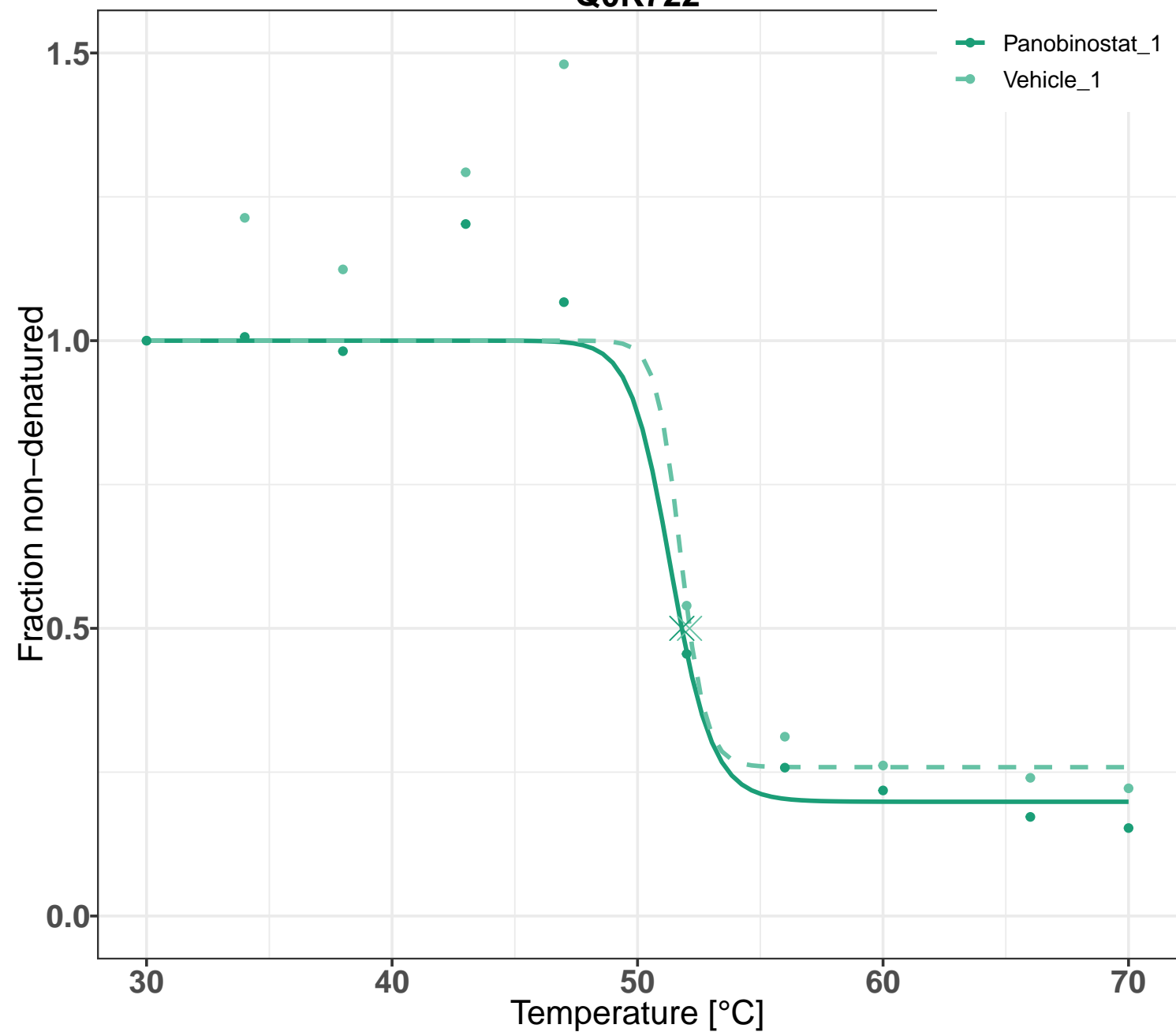

meltPoint

slope

plateau

R2

Panobinostat\_1

51.8

-0.24

0.2

0.97

Vehicle\_1

52.12

-0.37

0.26

0.83

Supplement: Supplementary file 2 — Supplementary Material 2 [file 41598_2026_35990_MOESM2_ESM.zip › AllTheTPPData/D40vD86/Panobinostat_Vignette/Melting_Curves/meltCurve_Q0K722.pdf]

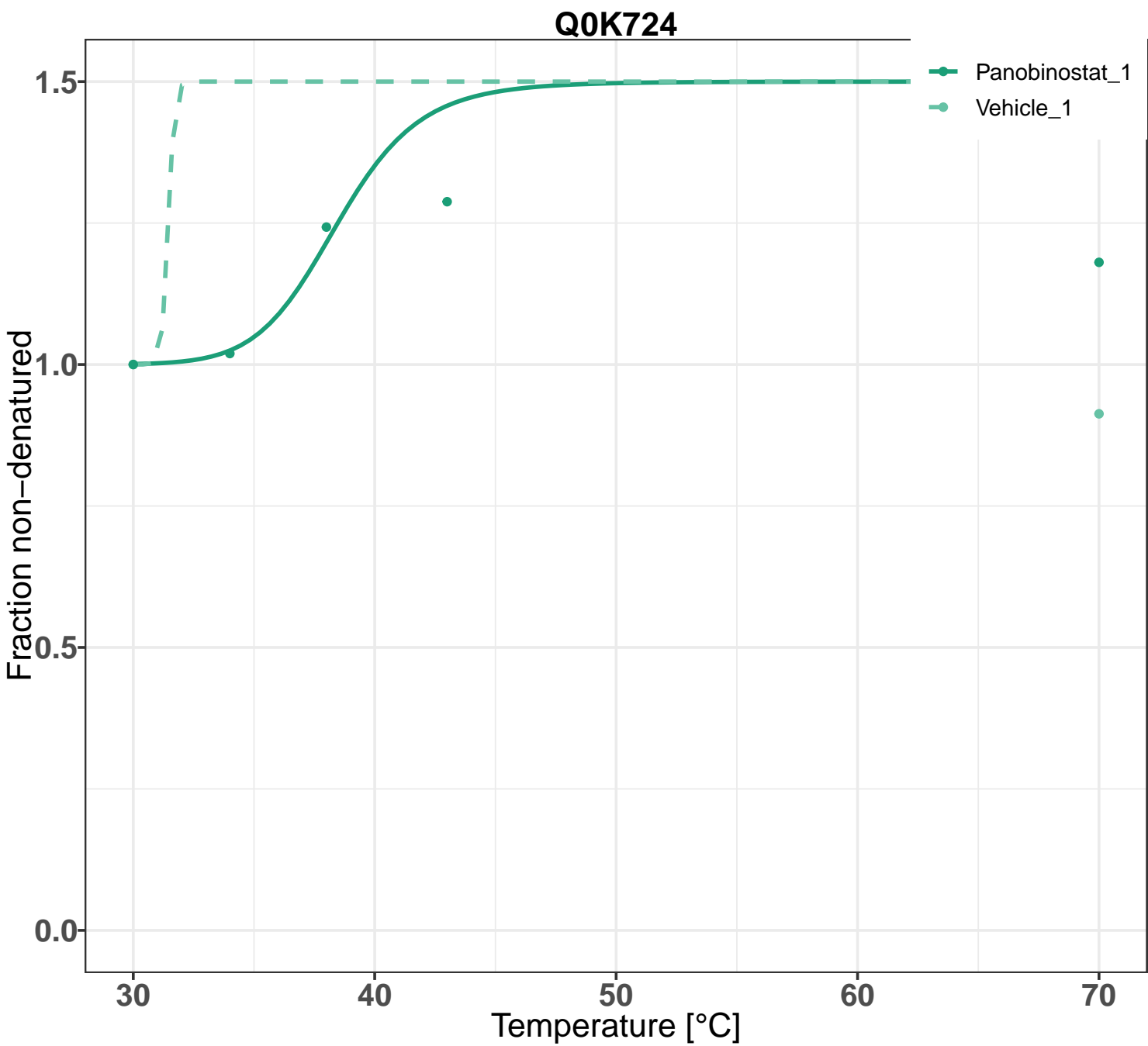

|                | meltPoint | slope | plateau | R2    |
|----------------|-----------|-------|---------|-------|
| Panobinostat_1 | —         | 0.074 | 1.5     | 0.24  |
| Vehicle_1      | —         | 0.99  | 1.5     | -0.52 |

Supplement: Supplementary file 2 — Supplementary Material 2 [file 41598_2026_35990_MOESM2_ESM.zip › AllTheTPPData/D40vD86/Panobinostat_Vignette/Melting_Curves/meltCurve_Q0K724.pdf]

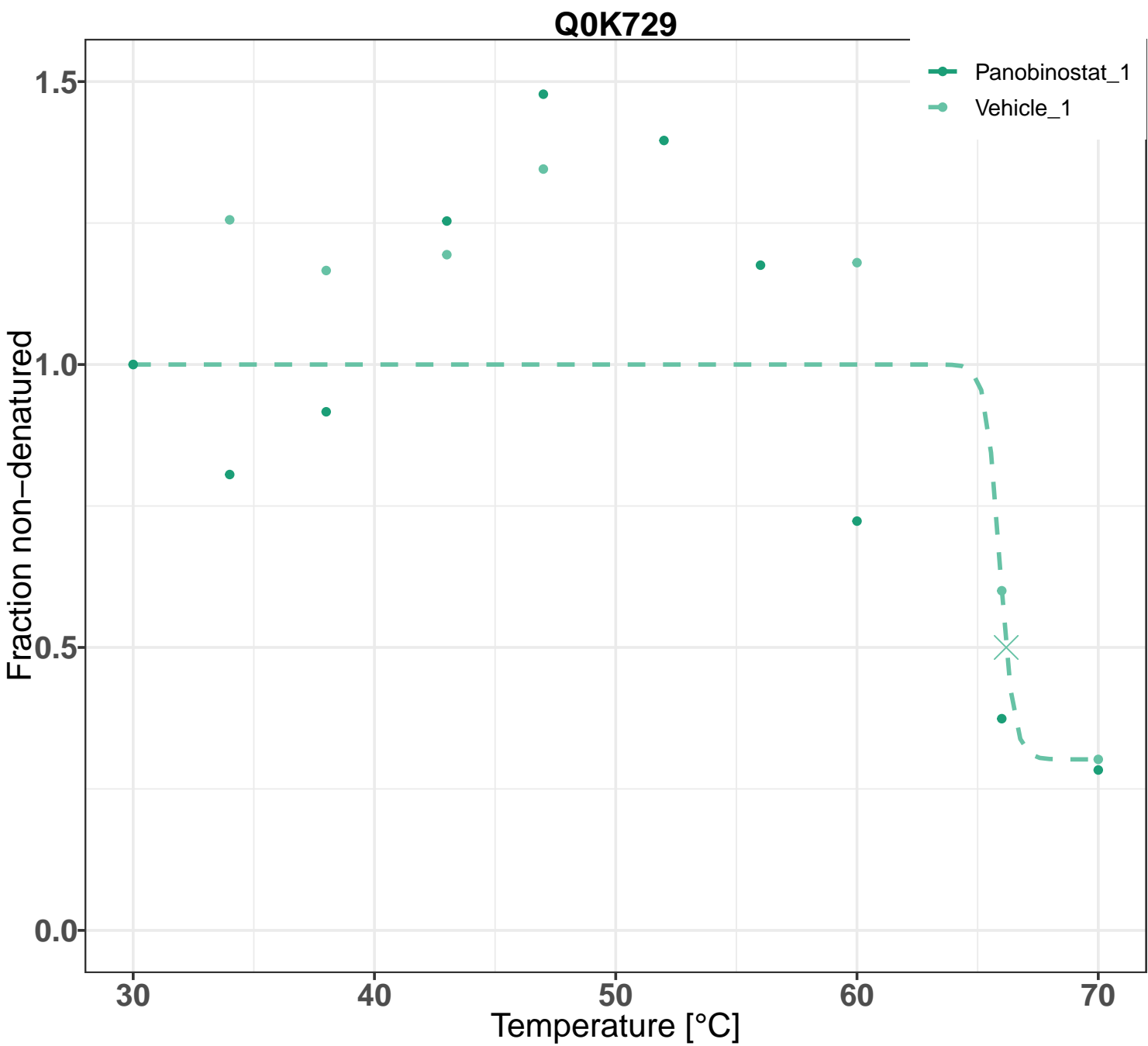

|                | meltPoint | slope | plateau | R2   |
|----------------|-----------|-------|---------|------|
| Panobinostat_1 | –         | –     | –       | –    |
| Vehicle_1      | 66.18     | –0.6  | 0.3     | 0.19 |

Supplement: Supplementary file 2 — Supplementary Material 2 [file 41598_2026_35990_MOESM2_ESM.zip › AllTheTPPData/D40vD86/Panobinostat_Vignette/Melting_Curves/meltCurve_Q0K729.pdf]

# Q0K740

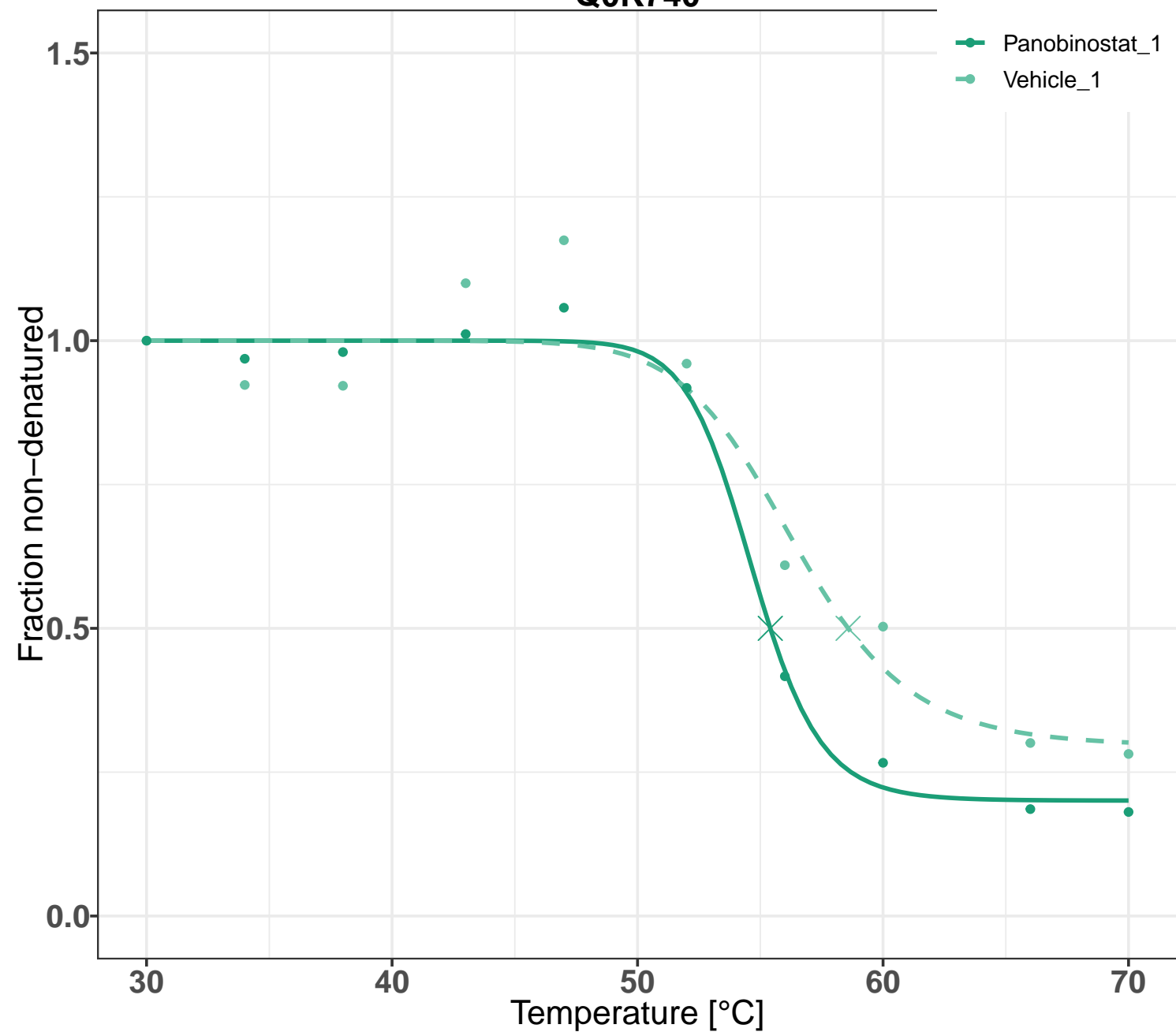

meltPoint

slope

plateau

R2

Panobinostat\_1

55.41

-0.15

0.2

0.99

Vehicle\_1

58.58

-0.075

0.29

0.93

Supplement: Supplementary file 2 — Supplementary Material 2 [file 41598_2026_35990_MOESM2_ESM.zip › AllTheTPPData/D40vD86/Panobinostat_Vignette/Melting_Curves/meltCurve_Q0K740.pdf]

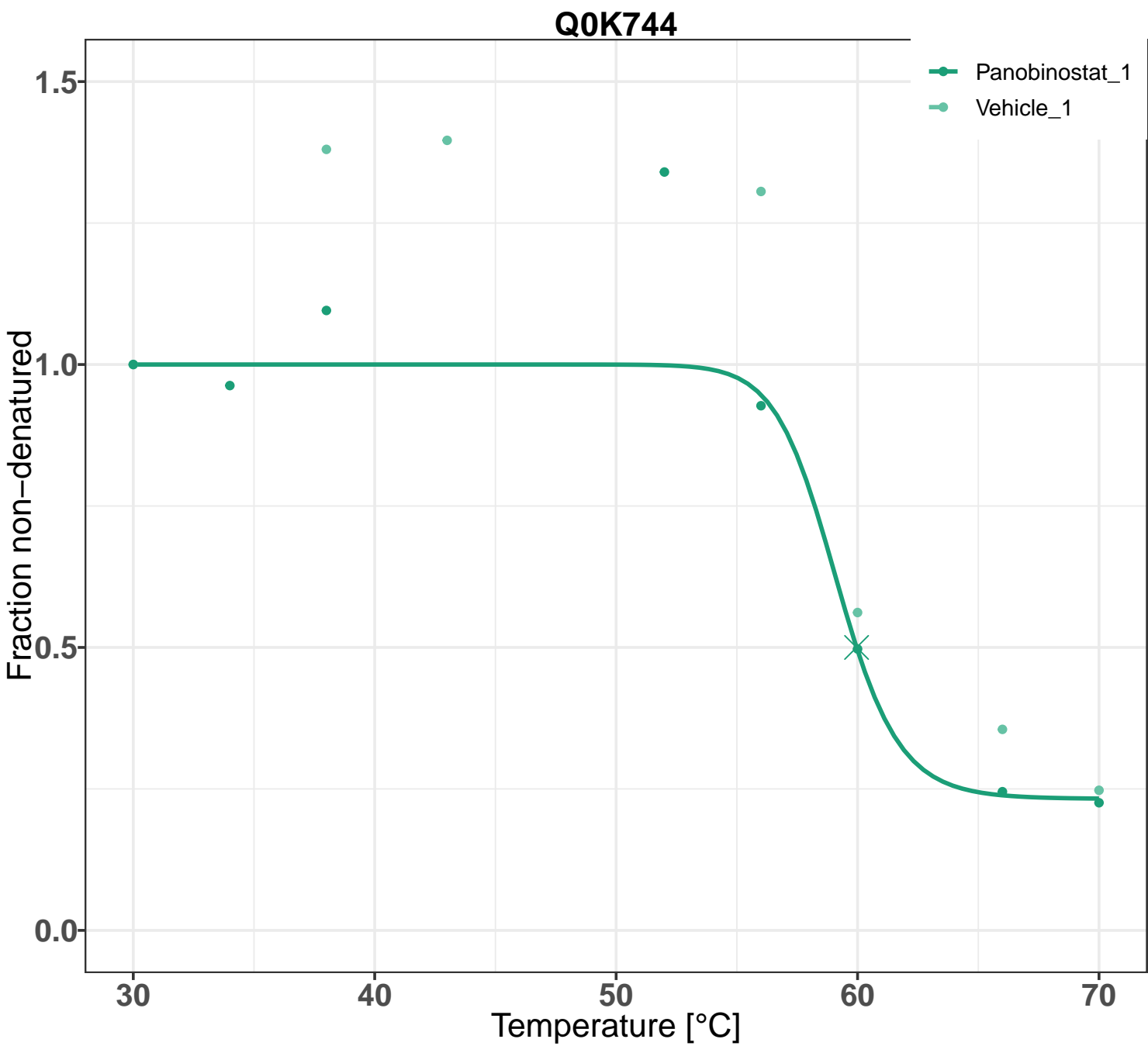

|                | meltPoint | slope | plateau | R2   |
|----------------|-----------|-------|---------|------|
| Panobinostat_1 | 59.96     | -0.15 | 0.23    | 0.56 |
| Vehicle_1      | —         | —     | —       | —    |

Supplement: Supplementary file 2 — Supplementary Material 2 [file 41598_2026_35990_MOESM2_ESM.zip › AllTheTPPData/D40vD86/Panobinostat_Vignette/Melting_Curves/meltCurve_Q0K744.pdf]

# Q0K755

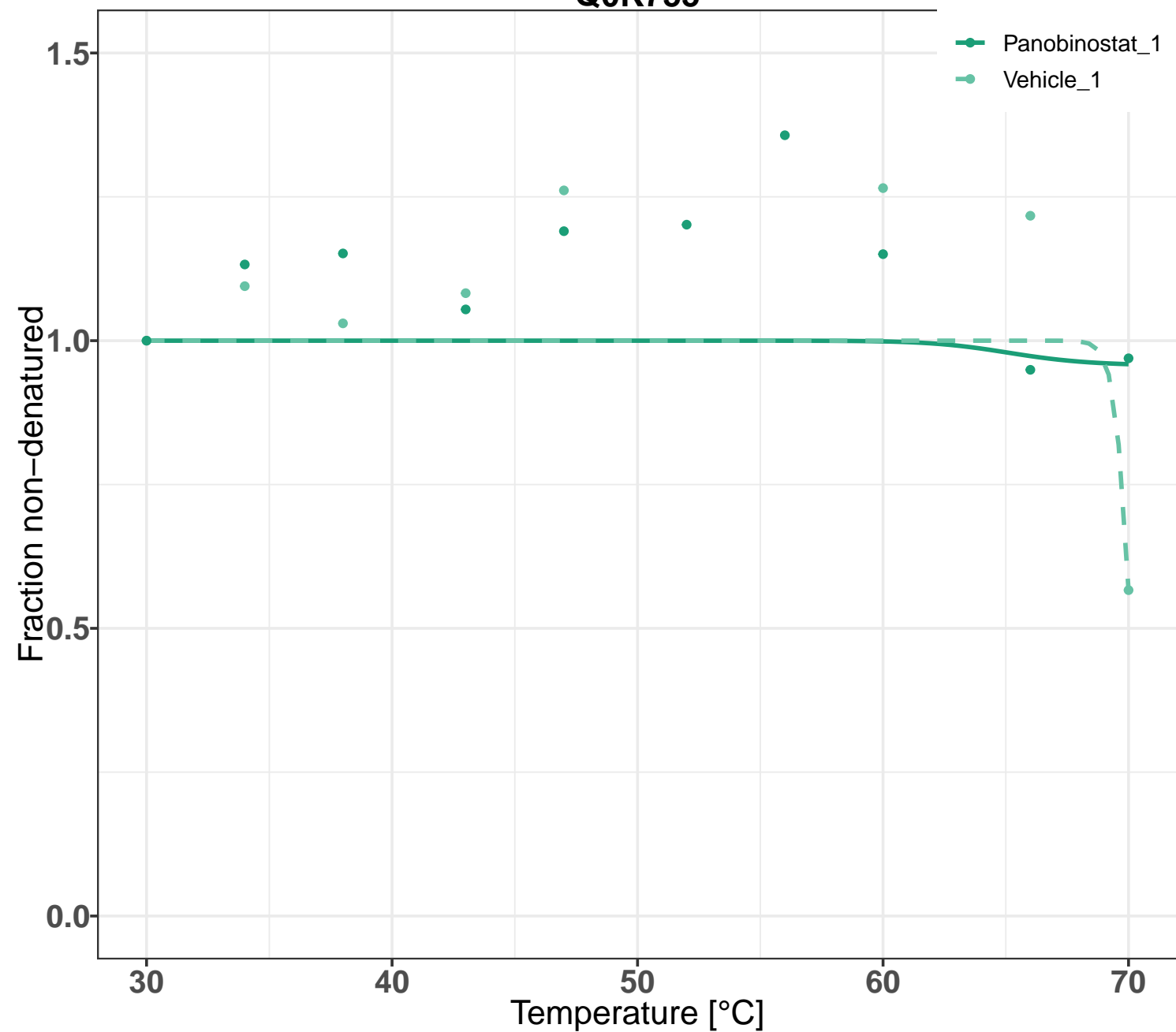

|                | meltPoint | slope   | plateau | R2    |
|----------------|-----------|---------|---------|-------|
| Panobinostat_1 | –         | –0.0067 | 0.96    | –0.94 |
| Vehicle_1      | –         | –       | 0       | –0.11 |

Supplement: Supplementary file 2 — Supplementary Material 2 [file 41598_2026_35990_MOESM2_ESM.zip › AllTheTPPData/D40vD86/Panobinostat_Vignette/Melting_Curves/meltCurve_Q0K755.pdf]

# Q0K757

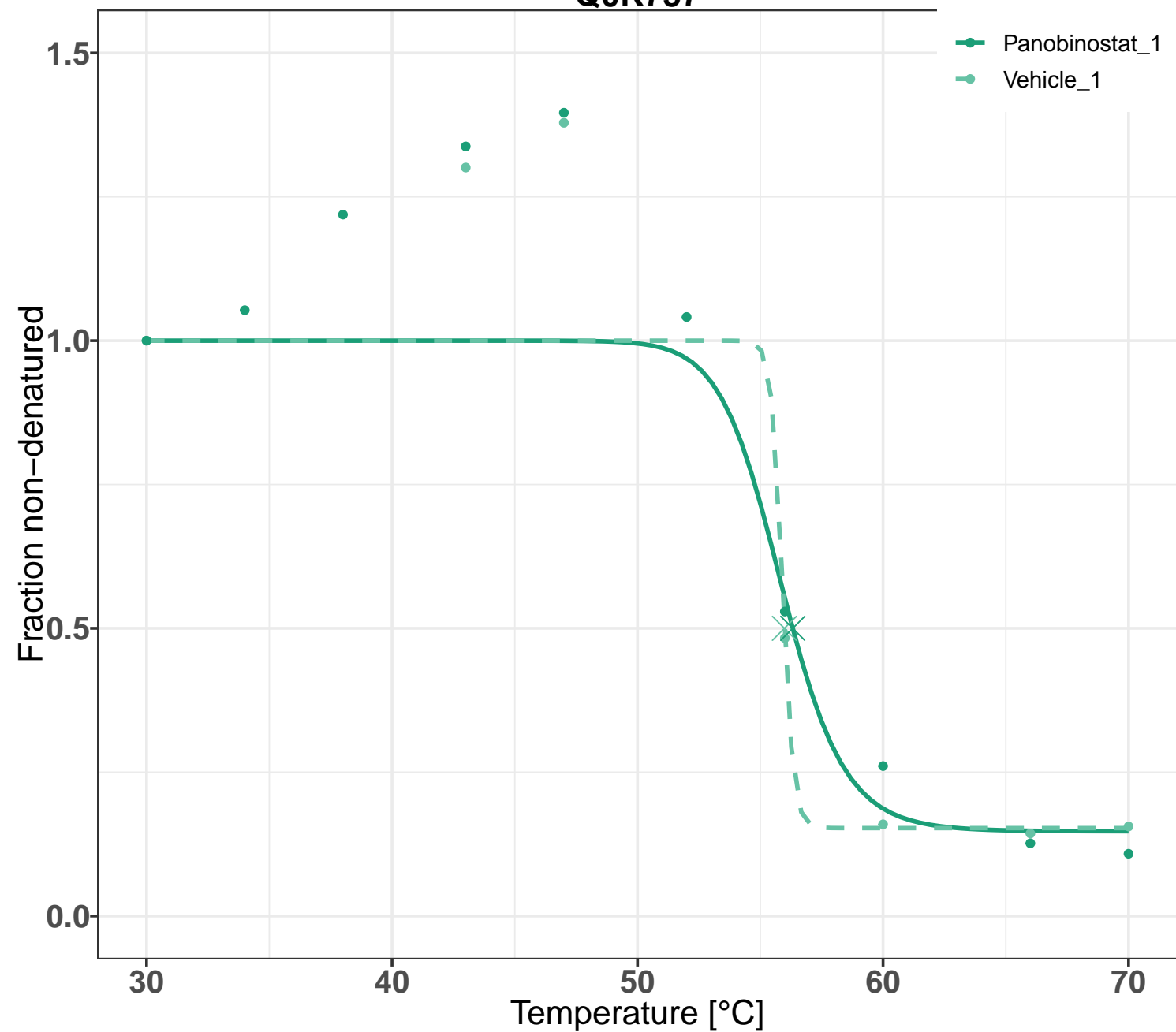

meltPoint

slope

plateau

R2

Panobinostat\_1

56.32

-0.17

0.15

0.85

Vehicle\_1

55.98

-0.95

0.15

0.58

Supplement: Supplementary file 2 — Supplementary Material 2 [file 41598_2026_35990_MOESM2_ESM.zip › AllTheTPPData/D40vD86/Panobinostat_Vignette/Melting_Curves/meltCurve_Q0K757.pdf]
