# Supplementary figures and images for: Molecular crowding effects on protein stability in a bacterial proteome (part 1 of 2)
Source: Sci Rep. 2026 Jan 21;16:5908. doi: 10.1038/s41598-026-35990-9 (PMC12894976; doi:10.1038/s41598-026-35990-9)

# A0AAE5ZAU9

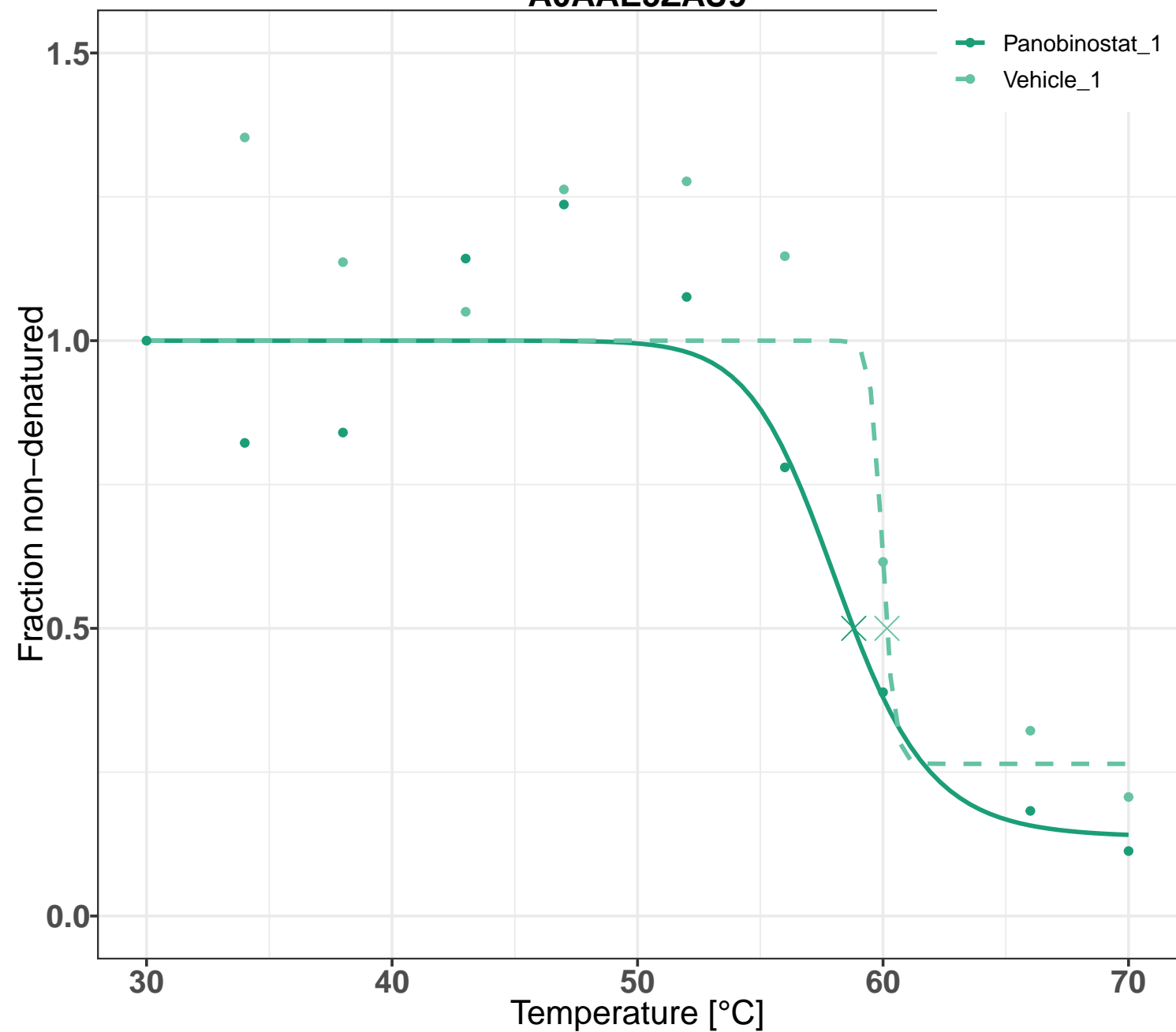

|                | meltPoint | slope | plateau | R2   |
|----------------|-----------|-------|---------|------|
| Panobinostat_1 | 58.81     | -0.12 | 0.14    | 0.9  |
| Vehicle_1      | 60.16     | -0.77 | 0.26    | 0.79 |

Supplement: Supplementary file 2 — Supplementary Material 2 [file 41598_2026_35990_MOESM2_ESM.zip › AllTheTPPData/D40vD86/Panobinostat_Vignette/Melting_Curves/meltCurve_A0AAE5ZAU9.pdf]

A0AAE5ZAZ1

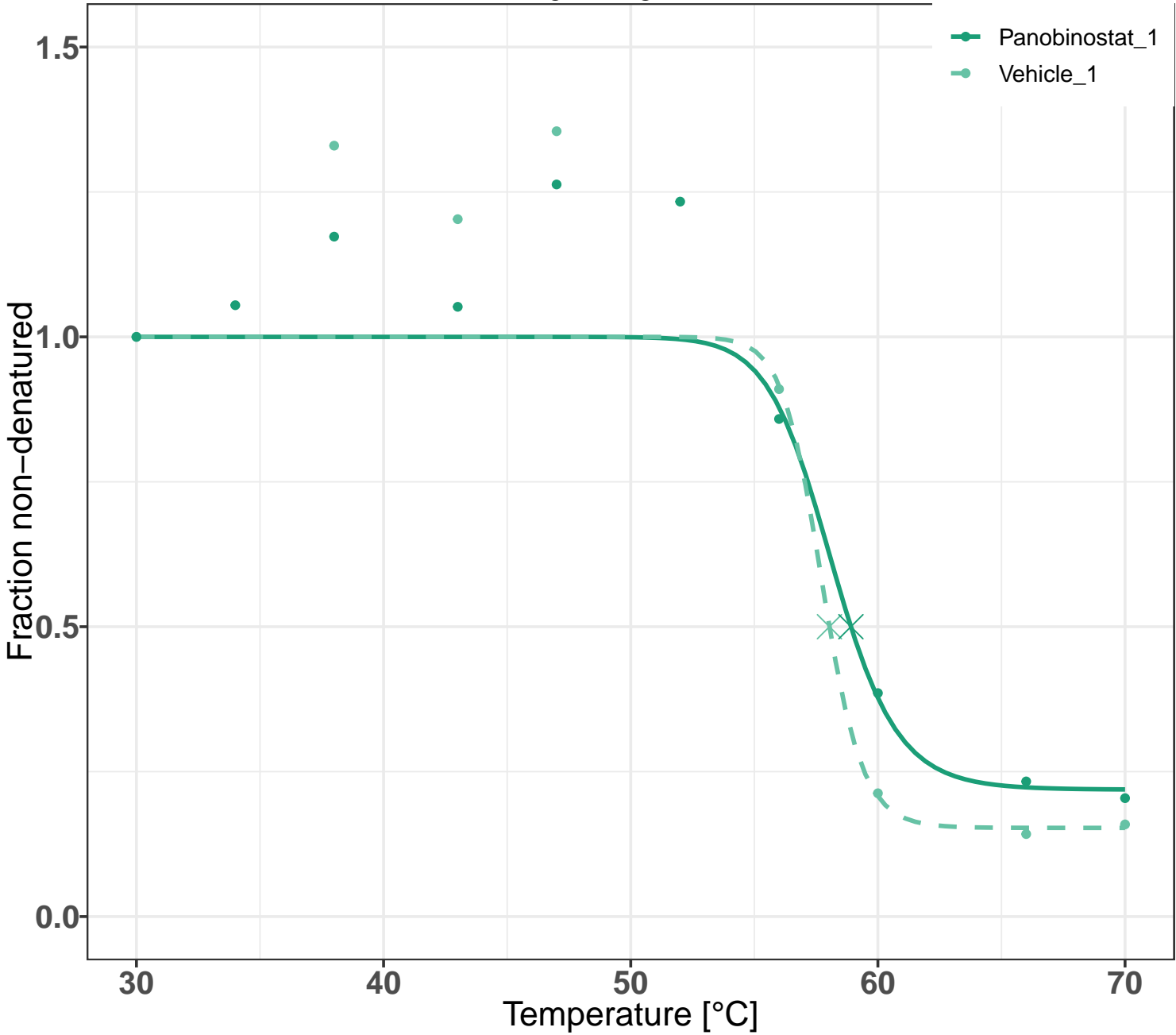

|                | meltPoint | slope | plateau | R2   |
|----------------|-----------|-------|---------|------|
| Panobinostat_1 | 58.91     | -0.15 | 0.22    | 0.89 |
| Vehicle_1      | 58.04     | -0.26 | 0.15    | 0.64 |

Supplement: Supplementary file 2 — Supplementary Material 2 [file 41598_2026_35990_MOESM2_ESM.zip › AllTheTPPData/D40vD86/Panobinostat_Vignette/Melting_Curves/meltCurve_A0AAE5ZAZ1.pdf]

# A0AAE5ZCH2

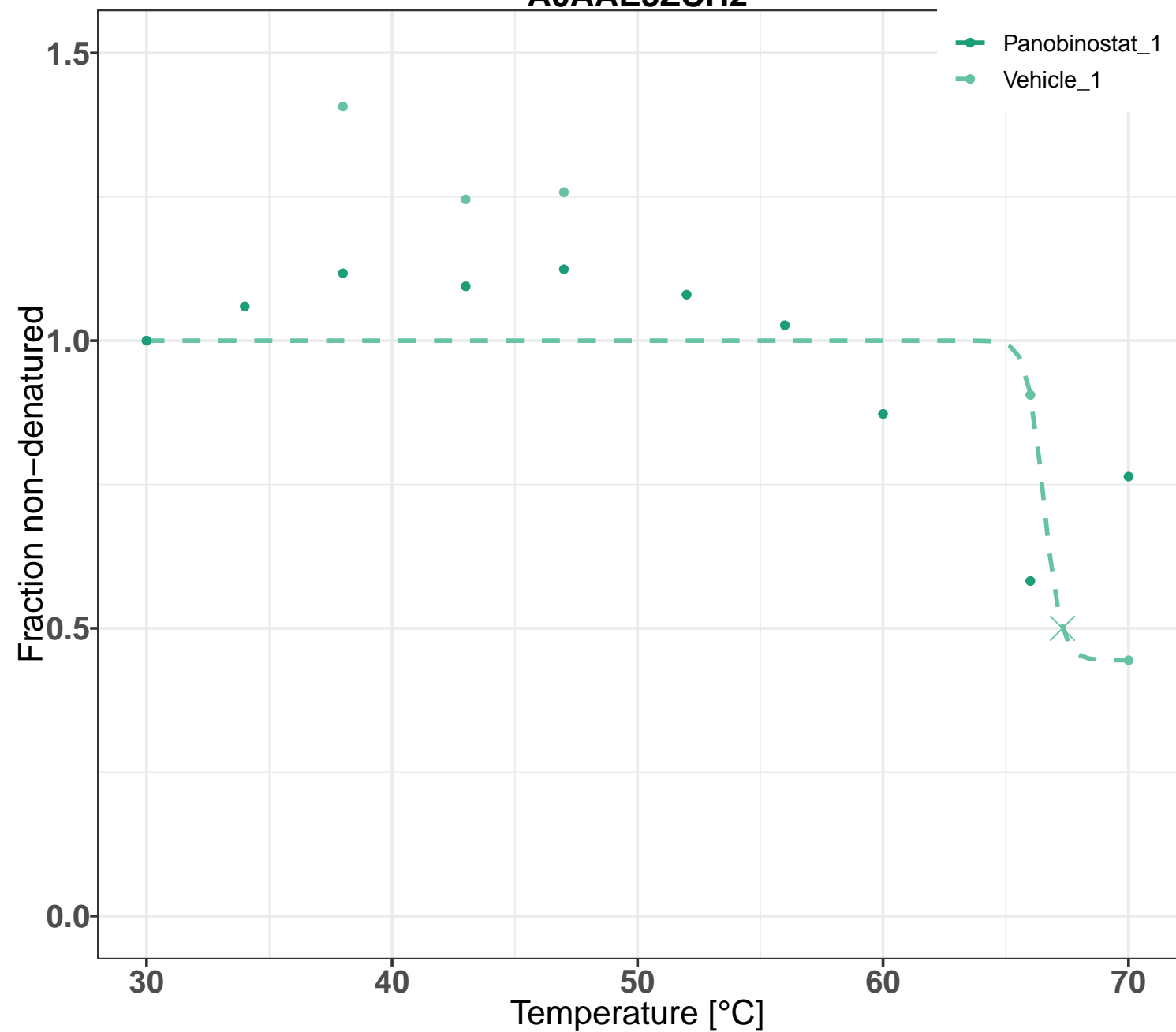

**meltPoint**

**slope**

**plateau**

**R2**

**Panobinostat\_1**

—

—

—

—

**Vehicle\_1**

**67.31**

**-0.4**

**0.44**

**-0.43**

Supplement: Supplementary file 2 — Supplementary Material 2 [file 41598_2026_35990_MOESM2_ESM.zip › AllTheTPPData/D40vD86/Panobinostat_Vignette/Melting_Curves/meltCurve_A0AAE5ZCH2.pdf]

A0AAE5ZCV1

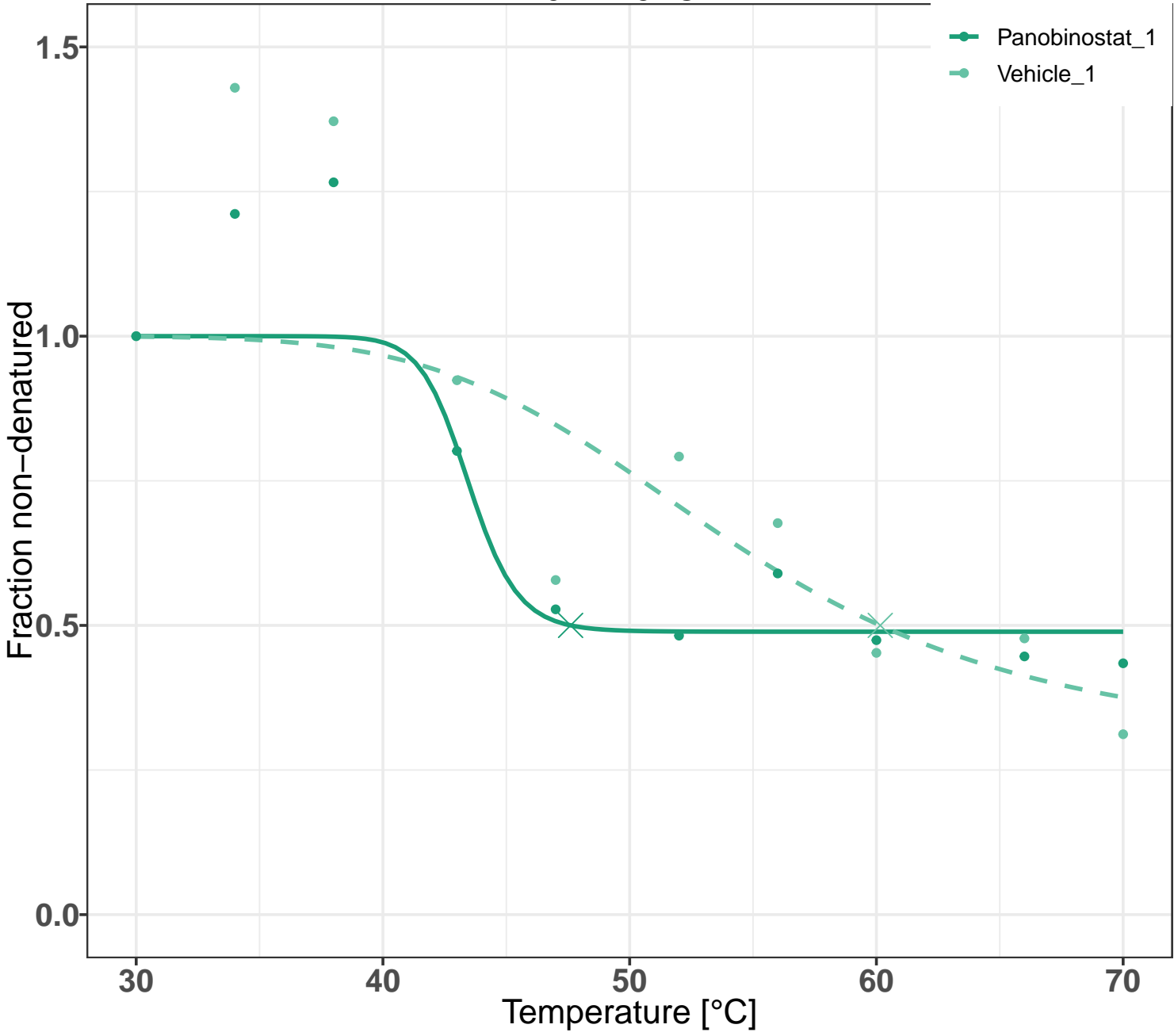

|                | meltPoint | slope | plateau | R2   |
|----------------|-----------|-------|---------|------|
| Panobinostat_1 | 47.61     | -0.13 | 0.49    | 0.86 |
| Vehicle_1      | 60.16     | -0.03 | 0.28    | 0.66 |

Supplement: Supplementary file 2 — Supplementary Material 2 [file 41598_2026_35990_MOESM2_ESM.zip › AllTheTPPData/D40vD86/Panobinostat_Vignette/Melting_Curves/meltCurve_A0AAE5ZCV1.pdf]

A0AAE5ZE15

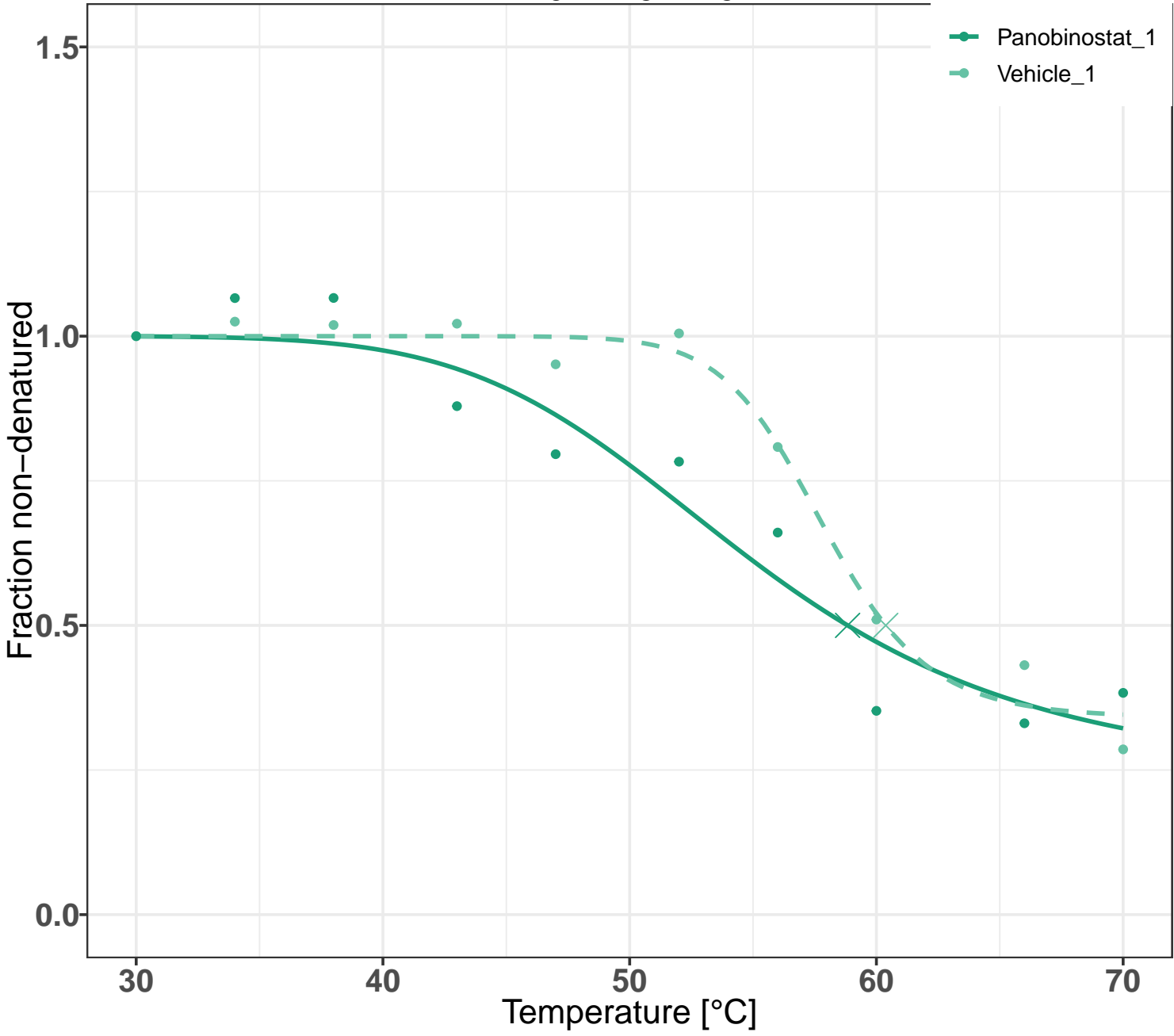

|                | meltPoint | slope  | plateau | R2   |
|----------------|-----------|--------|---------|------|
| Panobinostat_1 | 58.83     | -0.034 | 0.23    | 0.93 |
| Vehicle_1      | 60.38     | -0.079 | 0.34    | 0.98 |

Supplement: Supplementary file 2 — Supplementary Material 2 [file 41598_2026_35990_MOESM2_ESM.zip › AllTheTPPData/D40vD86/Panobinostat_Vignette/Melting_Curves/meltCurve_A0AAE5ZE15.pdf]

A0AAE5ZEY9

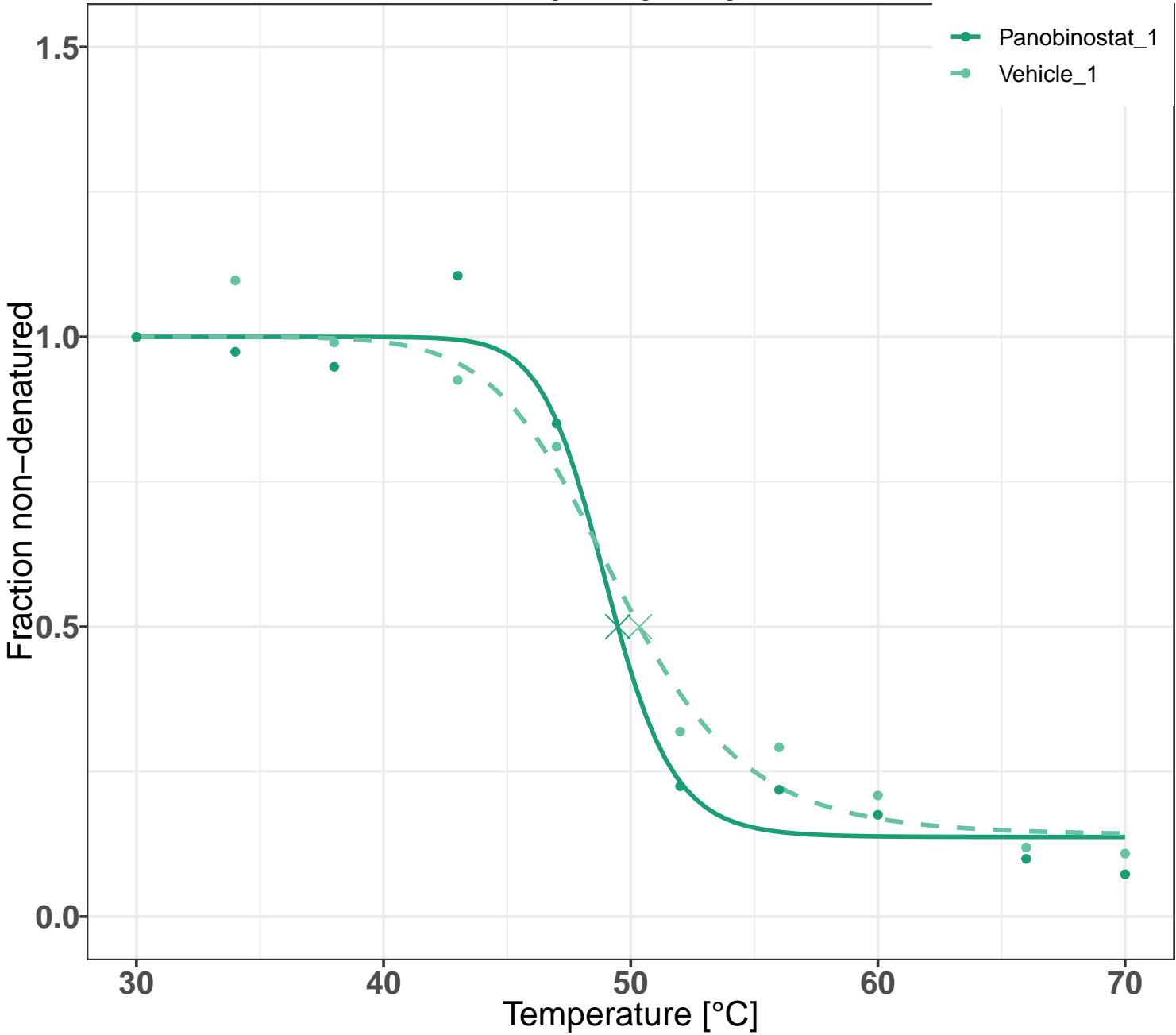

|                | meltPoint | slope  | plateau | R2   |
|----------------|-----------|--------|---------|------|
| Panobinostat_1 | 49.48     | -0.16  | 0.14    | 0.98 |
| Vehicle_1      | 50.35     | -0.084 | 0.14    | 0.98 |

Supplement: Supplementary file 2 — Supplementary Material 2 [file 41598_2026_35990_MOESM2_ESM.zip › AllTheTPPData/D40vD86/Panobinostat_Vignette/Melting_Curves/meltCurve_A0AAE5ZEY9.pdf]

# A0AAE5ZF35

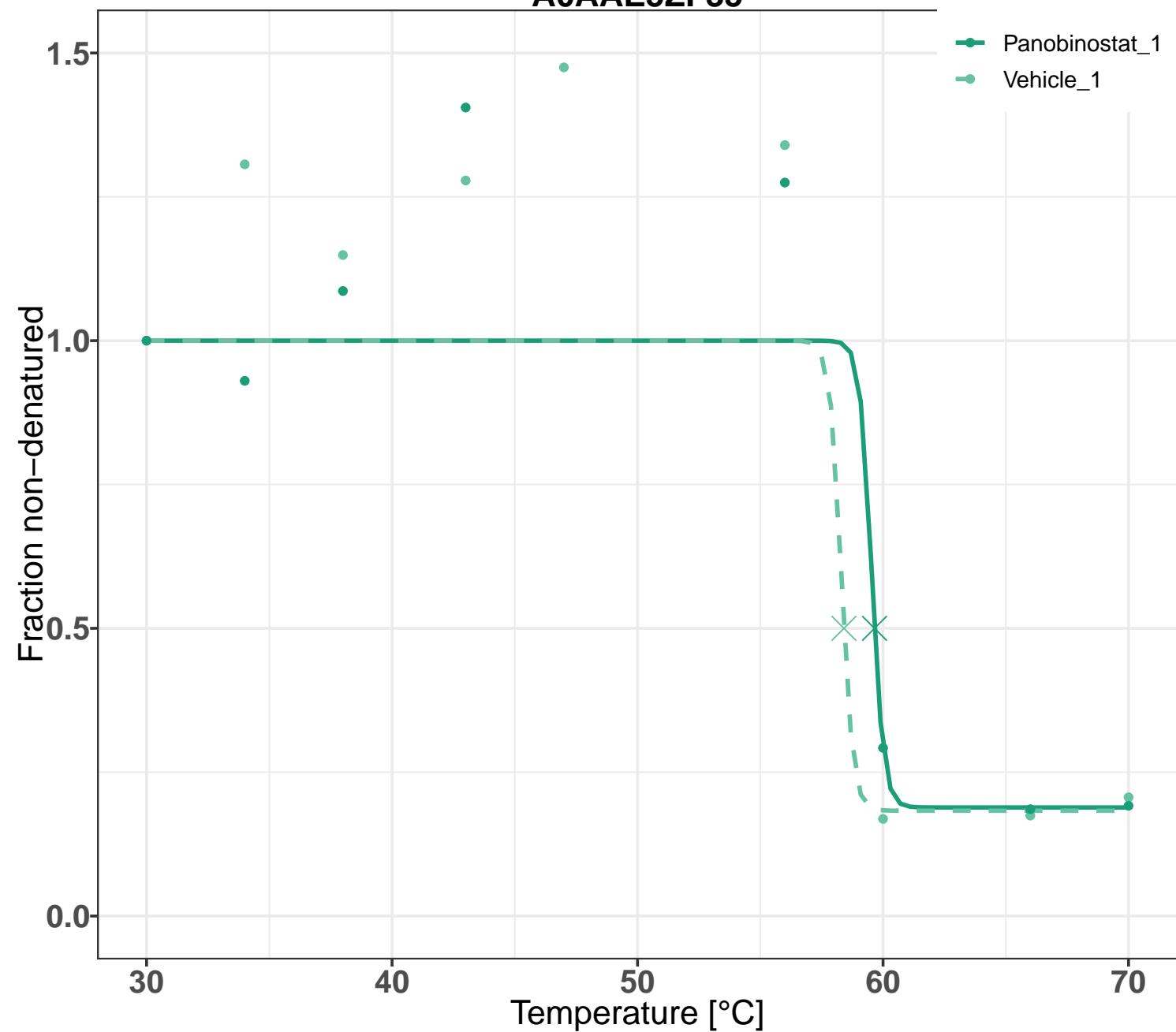

|                | meltPoint | slope | plateau | R2   |
|----------------|-----------|-------|---------|------|
| Panobinostat_1 | 59.65     | -0.85 | 0.19    | 0.63 |
| Vehicle_1      | 58.41     | -0.88 | 0.18    | 0.62 |

Supplement: Supplementary file 2 — Supplementary Material 2 [file 41598_2026_35990_MOESM2_ESM.zip › AllTheTPPData/D40vD86/Panobinostat_Vignette/Melting_Curves/meltCurve_A0AAE5ZF35.pdf]

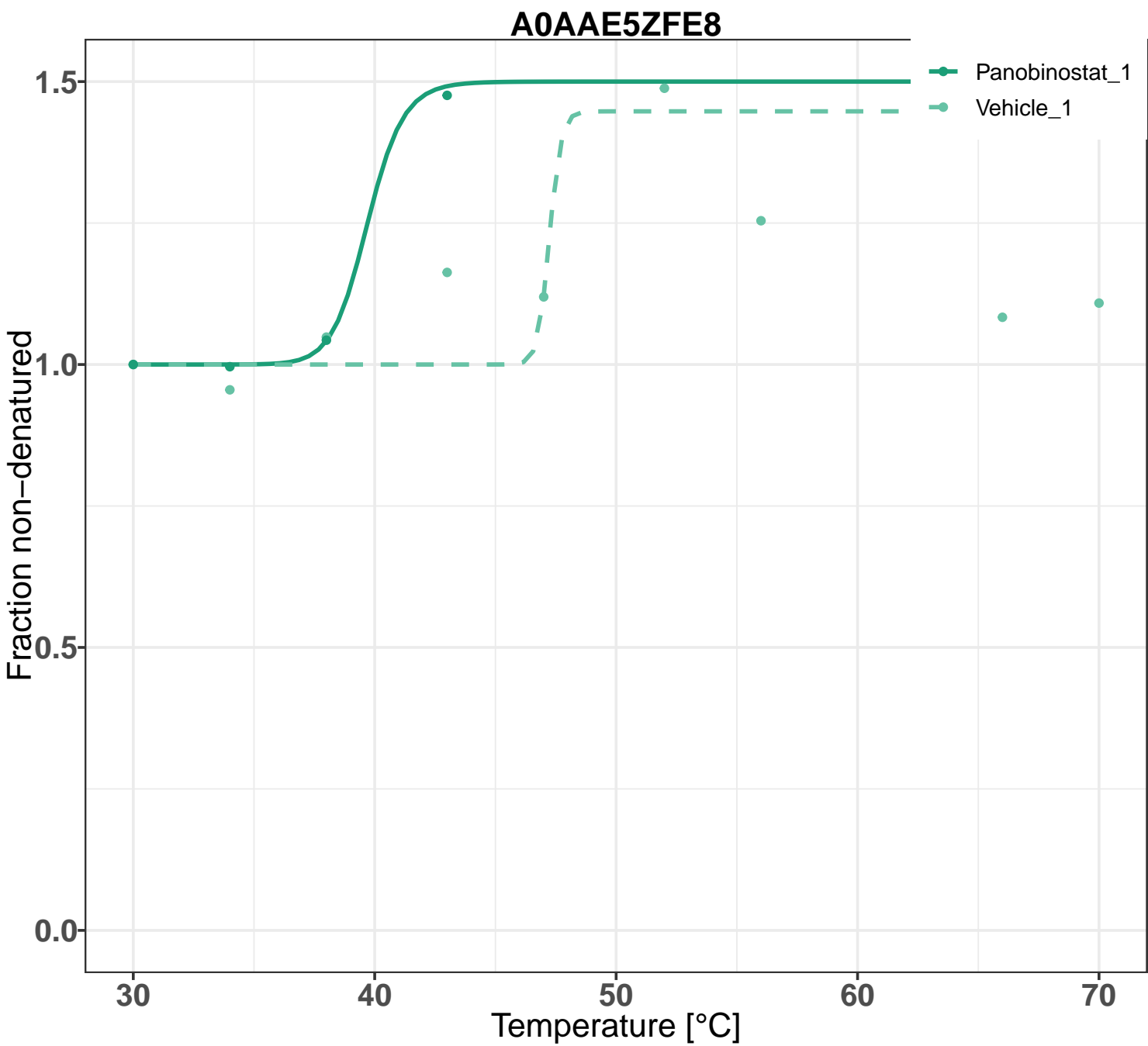

|                | meltPoint | slope | plateau | R2   |
|----------------|-----------|-------|---------|------|
| Panobinostat_1 | –         | 0.17  | 1.5     | –0.3 |
| Vehicle_1      | –         | 0.47  | 1.45    | 0.27 |

Supplement: Supplementary file 2 — Supplementary Material 2 [file 41598_2026_35990_MOESM2_ESM.zip › AllTheTPPData/D40vD86/Panobinostat_Vignette/Melting_Curves/meltCurve_A0AAE5ZFE8.pdf]

A0AAE5ZH50

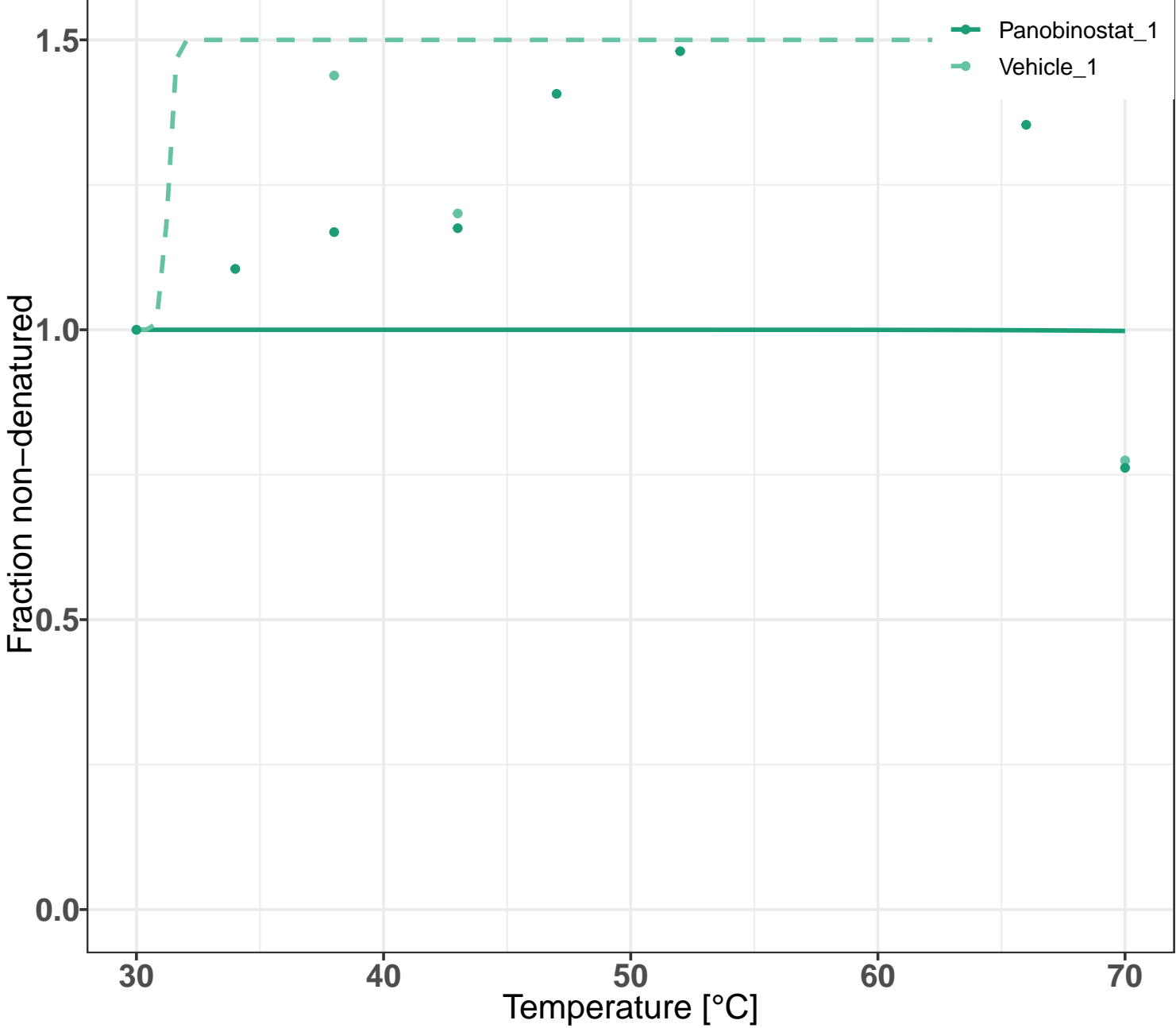

|                | meltPoint | slope | plateau | R2    |
|----------------|-----------|-------|---------|-------|
| Panobinostat_1 | -         | -     | 0       | -1.01 |
| Vehicle_1      | -         | 1     | 1.5     | -0.19 |

Supplement: Supplementary file 2 — Supplementary Material 2 [file 41598_2026_35990_MOESM2_ESM.zip › AllTheTPPData/D40vD86/Panobinostat_Vignette/Melting_Curves/meltCurve_A0AAE5ZH50.pdf]

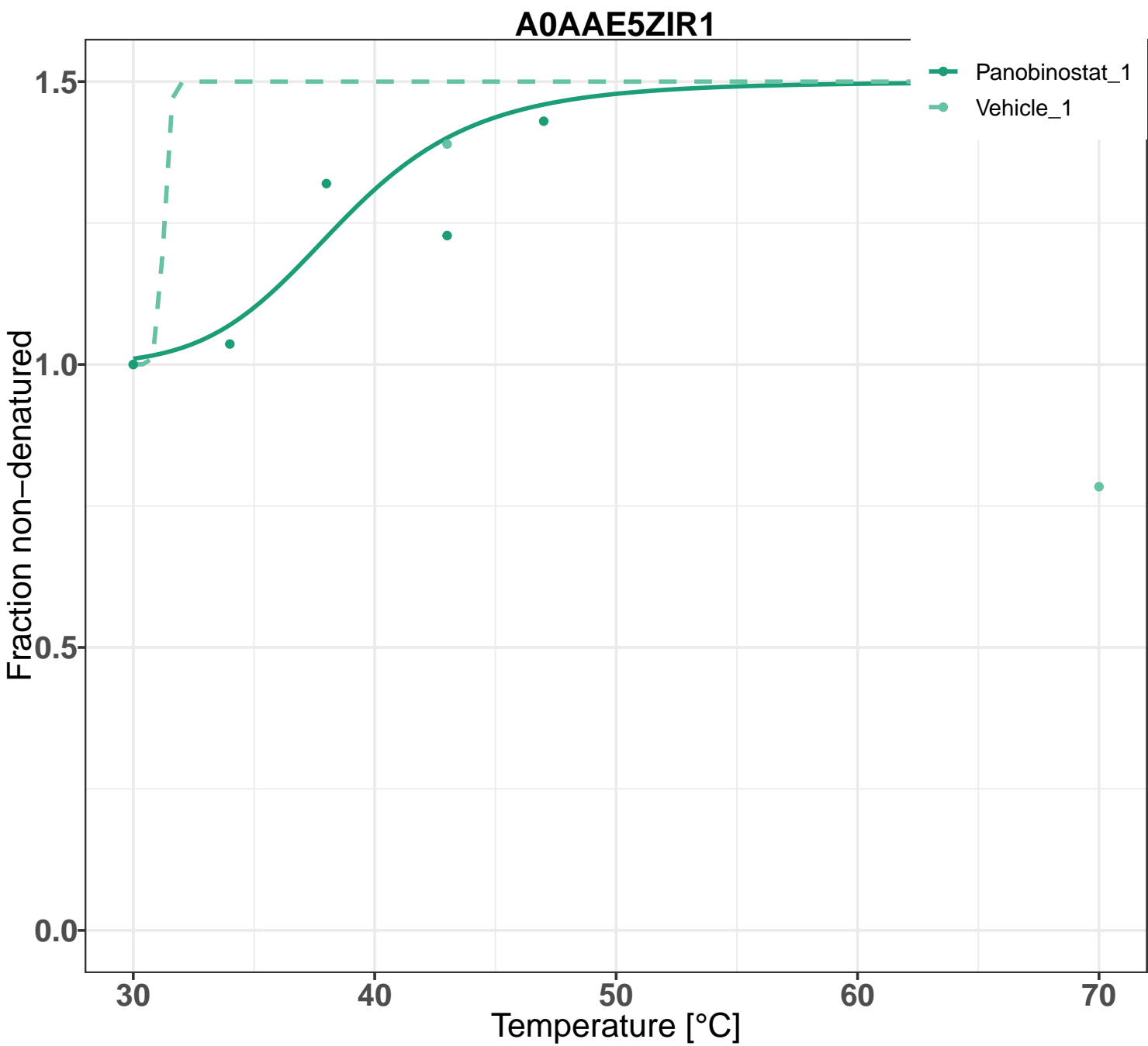

|                | meltPoint | slope | plateau | R2    |
|----------------|-----------|-------|---------|-------|
| Panobinostat_1 | –         | 0.045 | 1.5     | 0.68  |
| Vehicle_1      | –         | 1     | 1.5     | –0.22 |

Supplement: Supplementary file 2 — Supplementary Material 2 [file 41598_2026_35990_MOESM2_ESM.zip › AllTheTPPData/D40vD86/Panobinostat_Vignette/Melting_Curves/meltCurve_A0AAE5ZIR1.pdf]

A0AAE5ZLC1

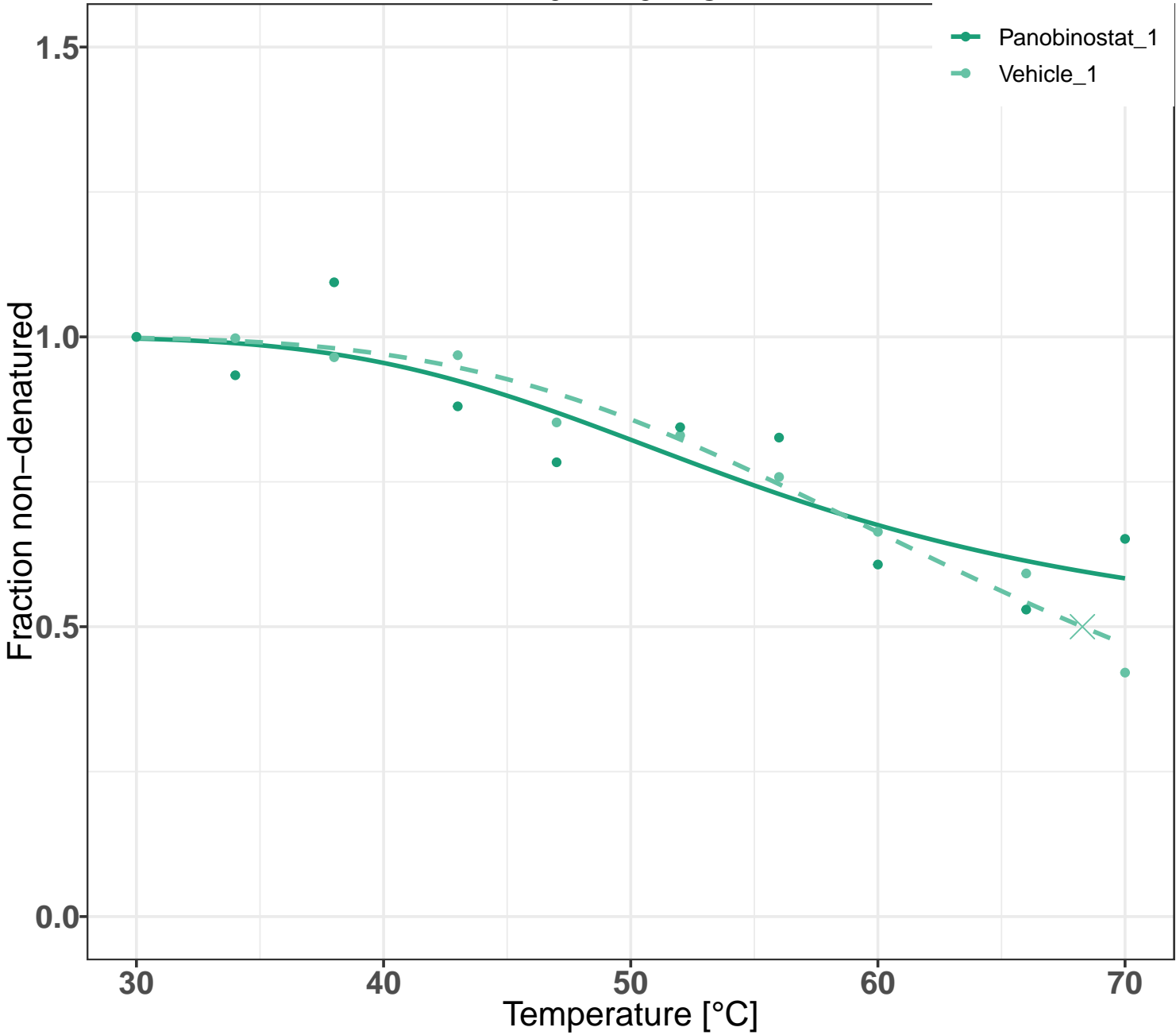

|                | meltPoint | slope  | plateau | R2   |
|----------------|-----------|--------|---------|------|
| Panobinostat_1 | -         | -0.016 | 0.46    | 0.8  |
| Vehicle_1      | 68.27     | -0.021 | 0       | 0.98 |

Supplement: Supplementary file 2 — Supplementary Material 2 [file 41598_2026_35990_MOESM2_ESM.zip › AllTheTPPData/D40vD86/Panobinostat_Vignette/Melting_Curves/meltCurve_A0AAE5ZLC1.pdf]

# A0AAE6DEN8

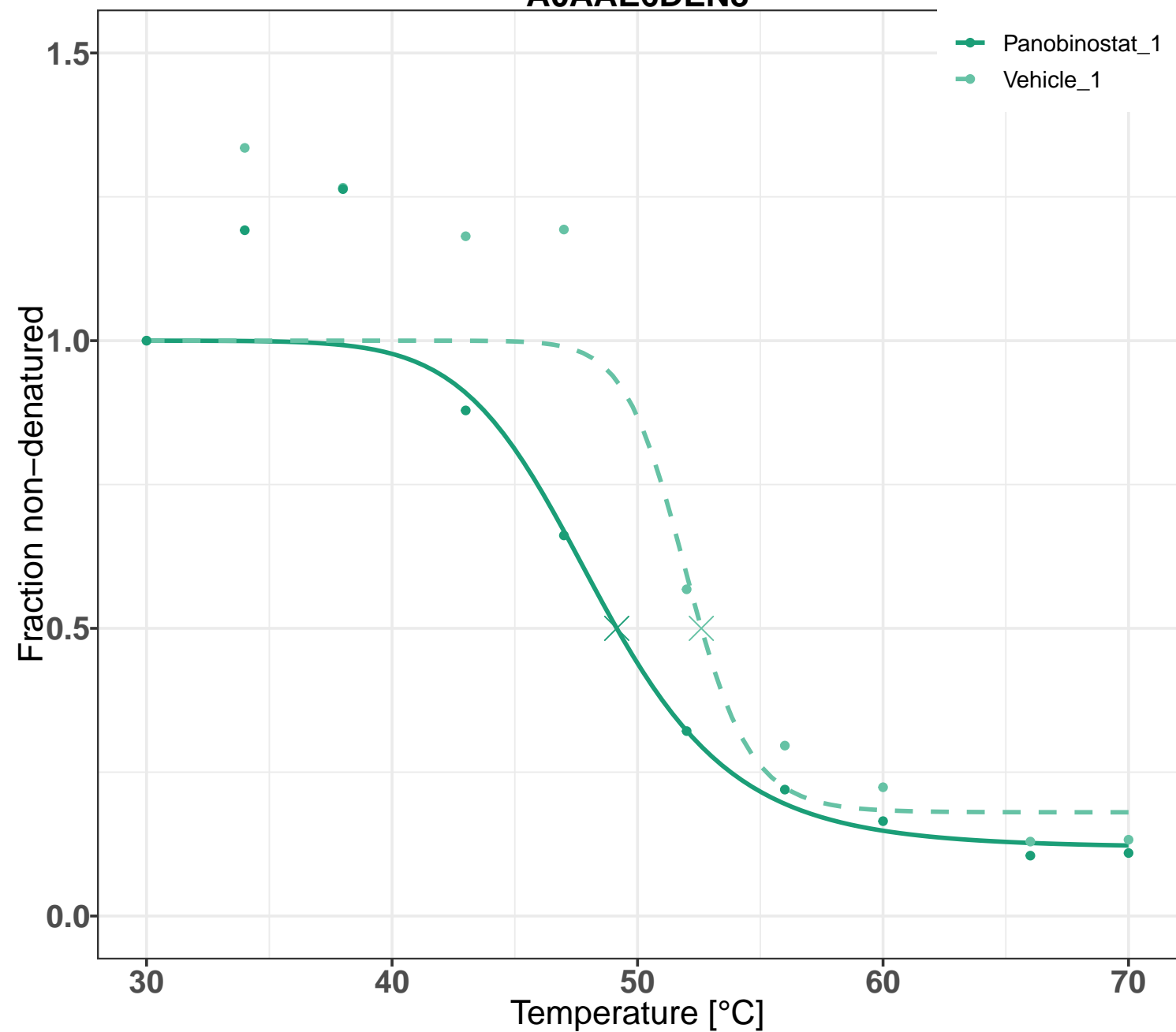

meltPoint

slope

plateau

R2

Panobinostat\_1

49.15

-0.08

0.12

0.94

Vehicle\_1

52.6

-0.16

0.18

0.88

Supplement: Supplementary file 2 — Supplementary Material 2 [file 41598_2026_35990_MOESM2_ESM.zip › AllTheTPPData/D40vD86/Panobinostat_Vignette/Melting_Curves/meltCurve_A0AAE6DEN8.pdf]

# A0AAE6DEV5

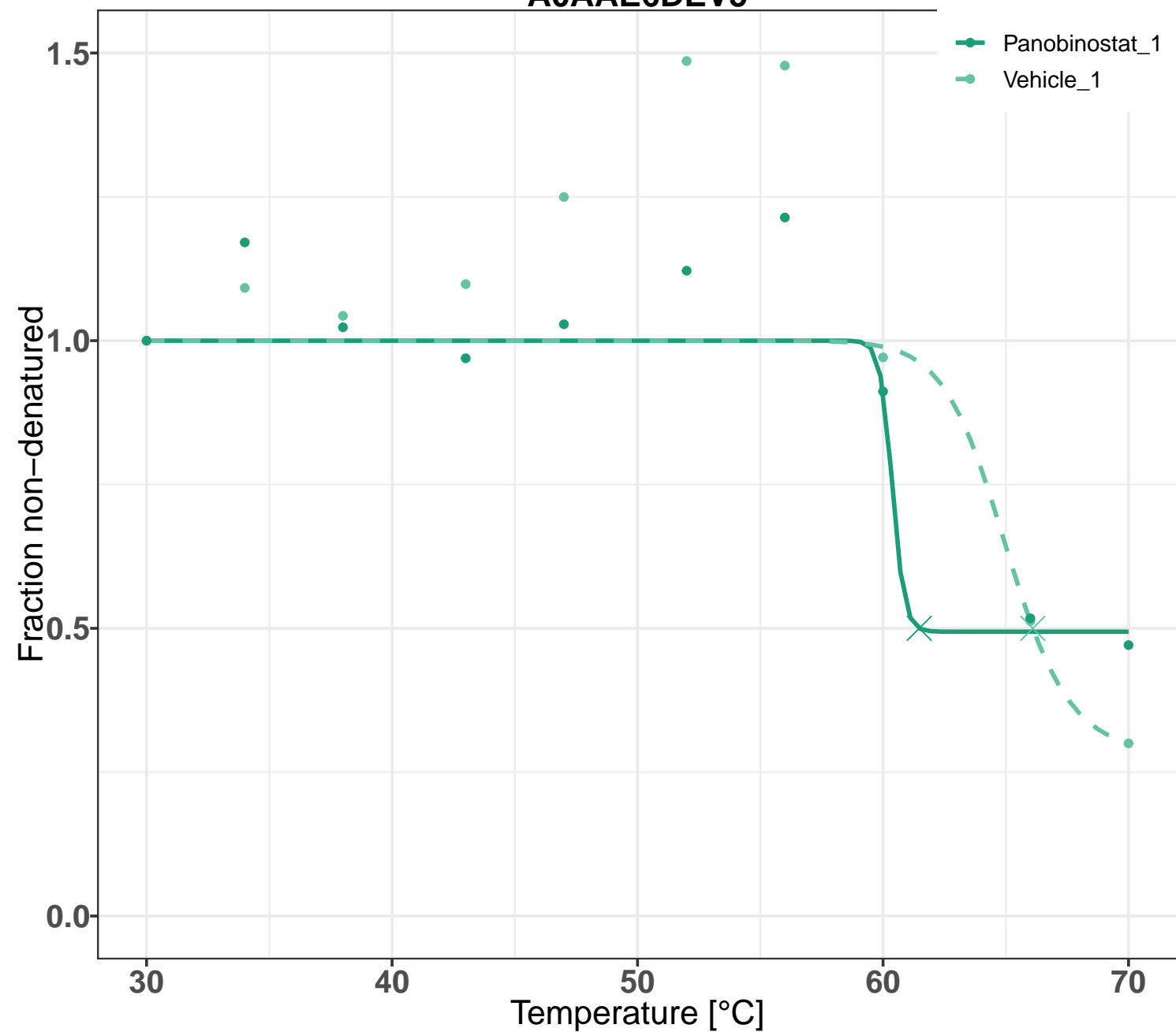

|                | meltPoint | slope | plateau | R2   |
|----------------|-----------|-------|---------|------|
| Panobinostat_1 | 61.47     | -0.52 | 0.49    | 0.84 |
| Vehicle_1      | 66.1      | -0.14 | 0.28    | 0.57 |

Supplement: Supplementary file 2 — Supplementary Material 2 [file 41598_2026_35990_MOESM2_ESM.zip › AllTheTPPData/D40vD86/Panobinostat_Vignette/Melting_Curves/meltCurve_A0AAE6DEV5.pdf]

A0AAE6DF06

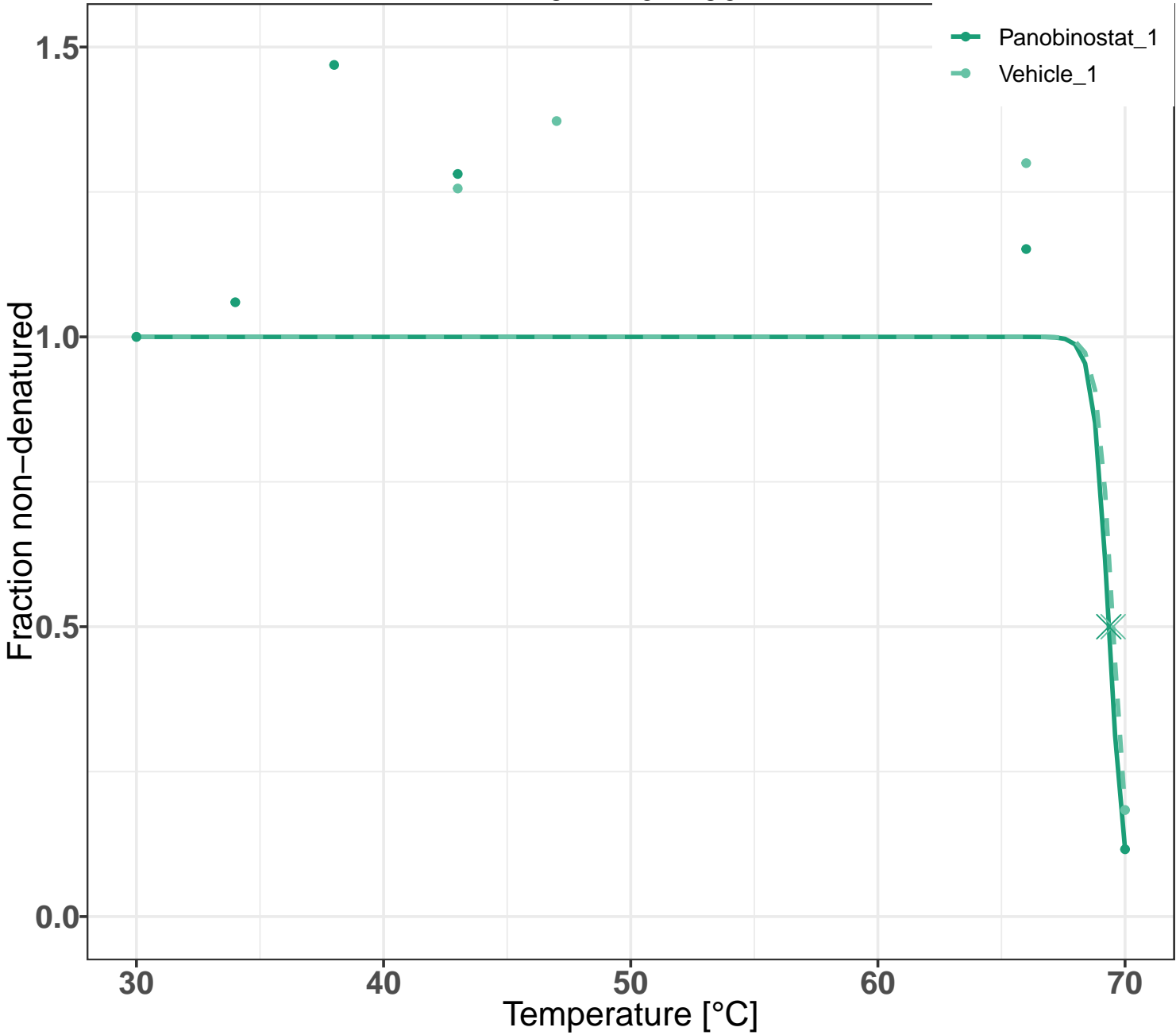

|                | meltPoint | slope | plateau | R2    |
|----------------|-----------|-------|---------|-------|
| Panobinostat_1 | 69.34     | -0.78 | 0       | -0.2  |
| Vehicle_1      | 69.52     | -0.78 | 0       | -0.58 |

Supplement: Supplementary file 2 — Supplementary Material 2 [file 41598_2026_35990_MOESM2_ESM.zip › AllTheTPPData/D40vD86/Panobinostat_Vignette/Melting_Curves/meltCurve_A0AAE6DF06.pdf]

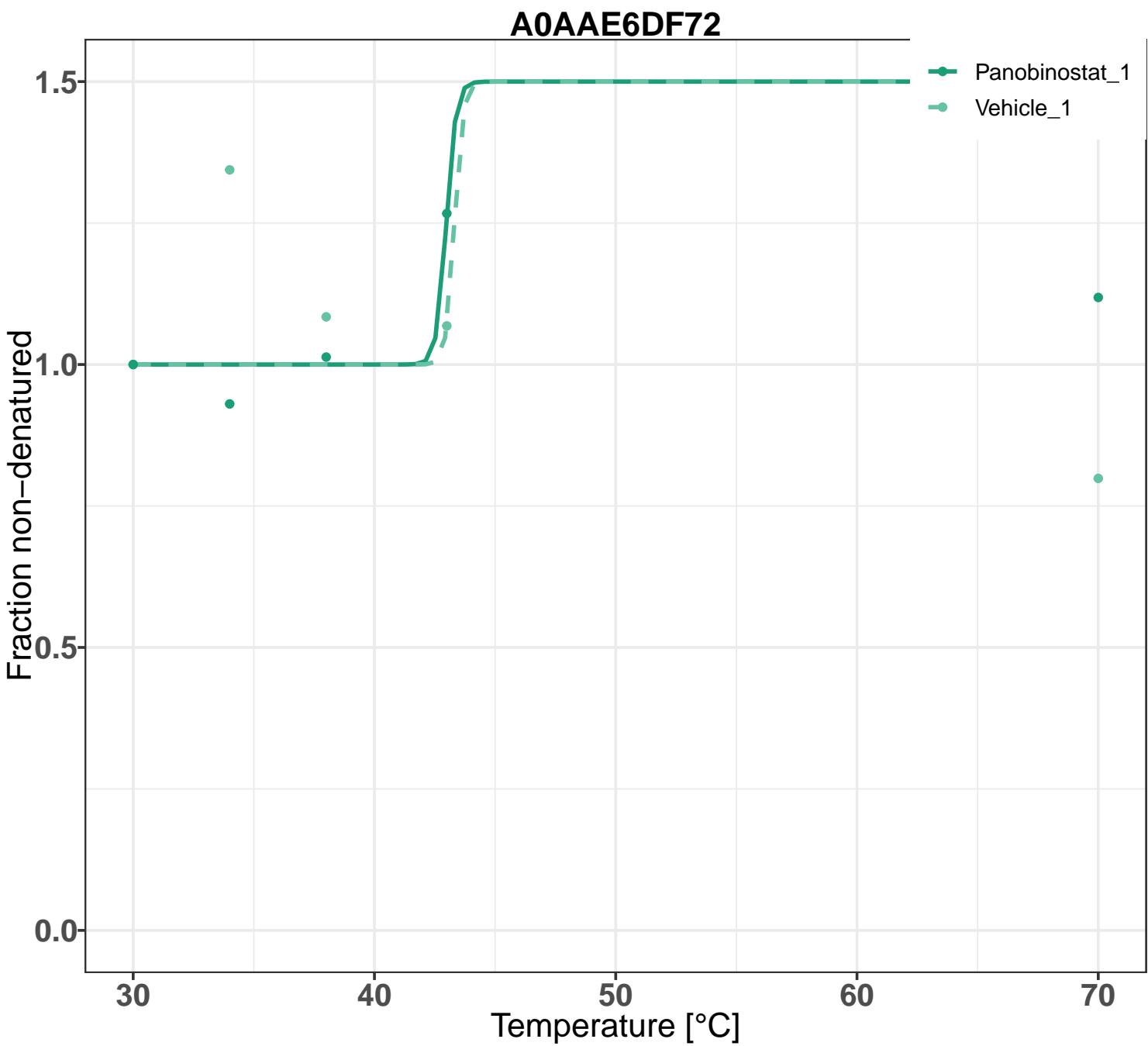

|                | meltPoint | slope | plateau | R2    |
|----------------|-----------|-------|---------|-------|
| Panobinostat_1 | –         | 0.63  | 1.5     | 0.36  |
| Vehicle_1      | –         | 0.72  | 1.5     | –0.12 |

Supplement: Supplementary file 2 — Supplementary Material 2 [file 41598_2026_35990_MOESM2_ESM.zip › AllTheTPPData/D40vD86/Panobinostat_Vignette/Melting_Curves/meltCurve_A0AAE6DF72.pdf]

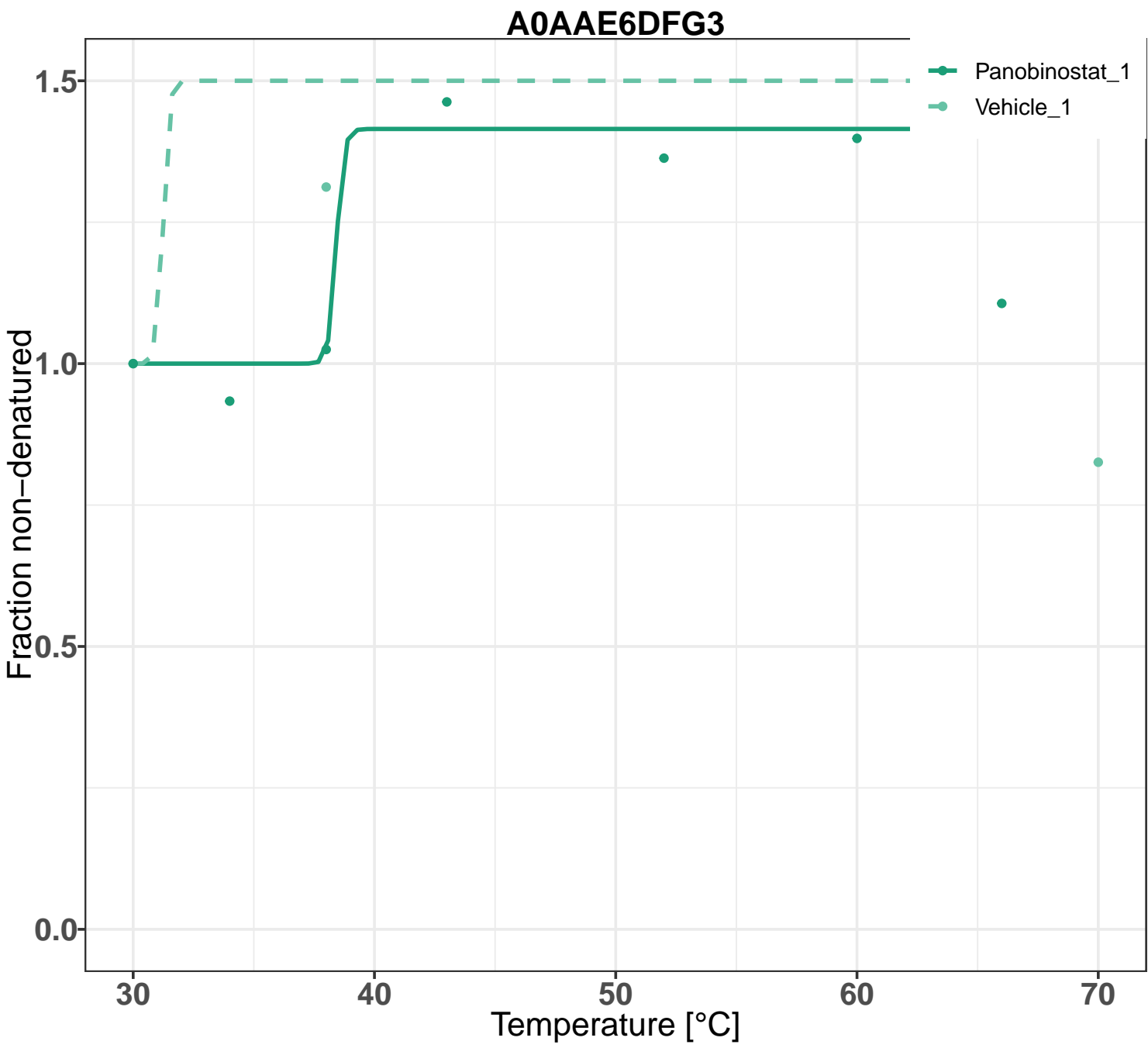

|                | meltPoint | slope | plateau | R2    |
|----------------|-----------|-------|---------|-------|
| Panobinostat_1 | –         | 0.67  | 1.41    | 0.73  |
| Vehicle_1      | –         | 1     | 1.5     | –0.24 |

Supplement: Supplementary file 2 — Supplementary Material 2 [file 41598_2026_35990_MOESM2_ESM.zip › AllTheTPPData/D40vD86/Panobinostat_Vignette/Melting_Curves/meltCurve_A0AAE6DFG3.pdf]

# A0AAE6DFK2

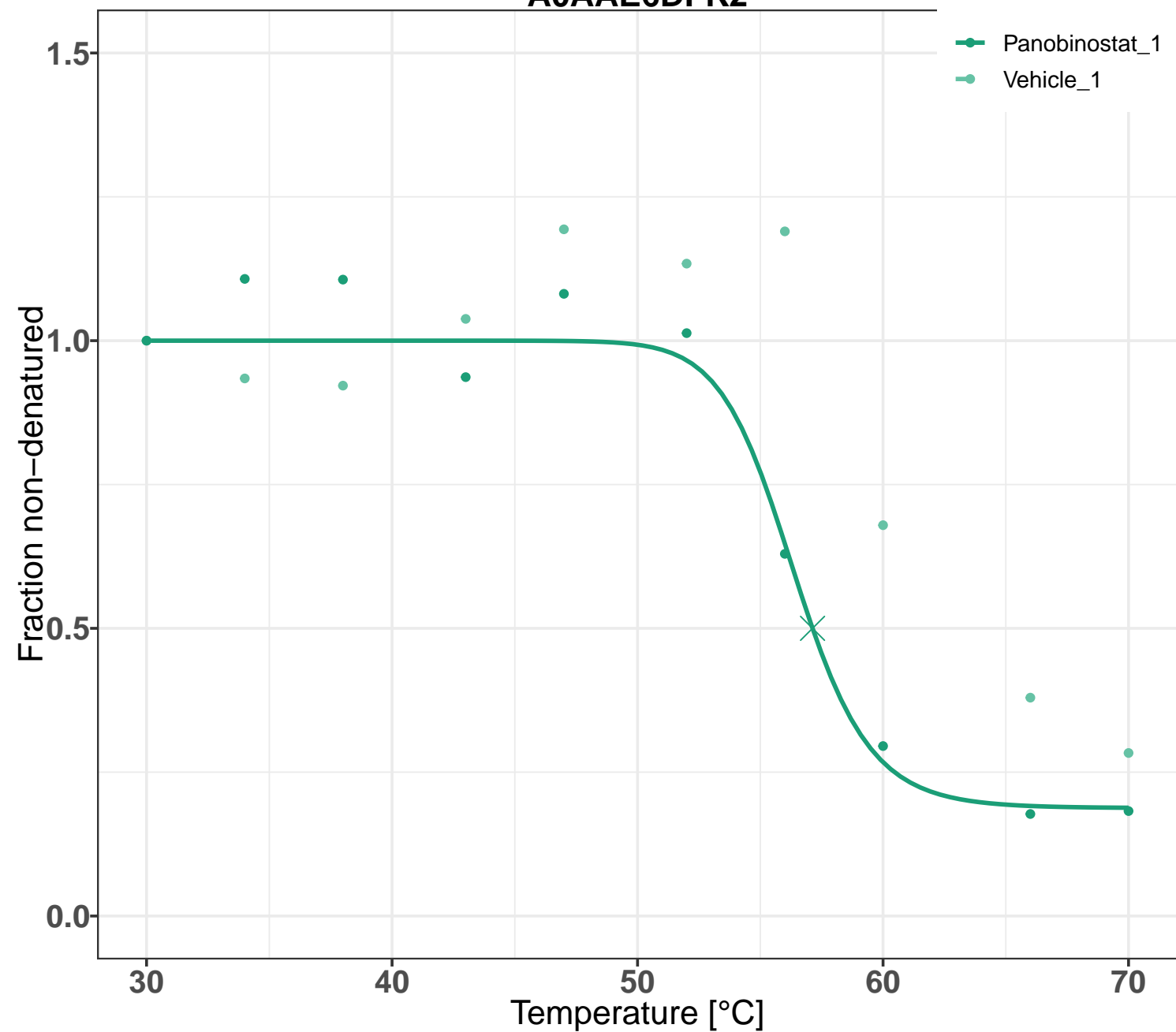

meltPoint

slope

plateau

R2

Panobinostat\_1

57.13

-0.13

0.19

0.97

Vehicle\_1

-

-

-

-

Supplement: Supplementary file 2 — Supplementary Material 2 [file 41598_2026_35990_MOESM2_ESM.zip › AllTheTPPData/D40vD86/Panobinostat_Vignette/Melting_Curves/meltCurve_A0AAE6DFK2.pdf]

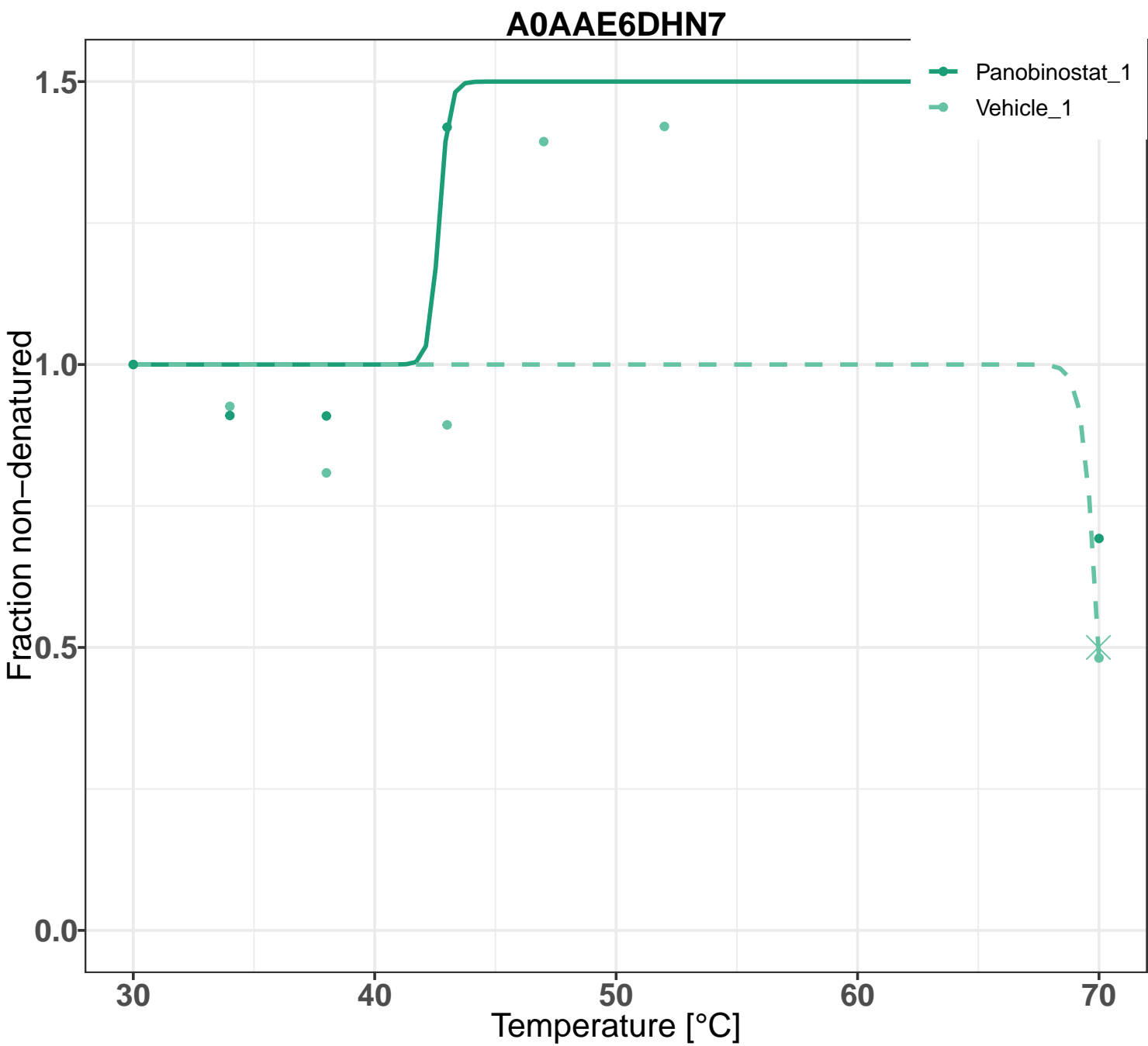

|                | meltPoint | slope | plateau | R2    |
|----------------|-----------|-------|---------|-------|
| Panobinostat_1 | –         | 0.61  | 1.5     | 0.41  |
| Vehicle_1      | 69.98     | –0.77 | 0       | –0.22 |

Supplement: Supplementary file 2 — Supplementary Material 2 [file 41598_2026_35990_MOESM2_ESM.zip › AllTheTPPData/D40vD86/Panobinostat_Vignette/Melting_Curves/meltCurve_A0AAE6DHN7.pdf]

# A0AAE6DHQ6

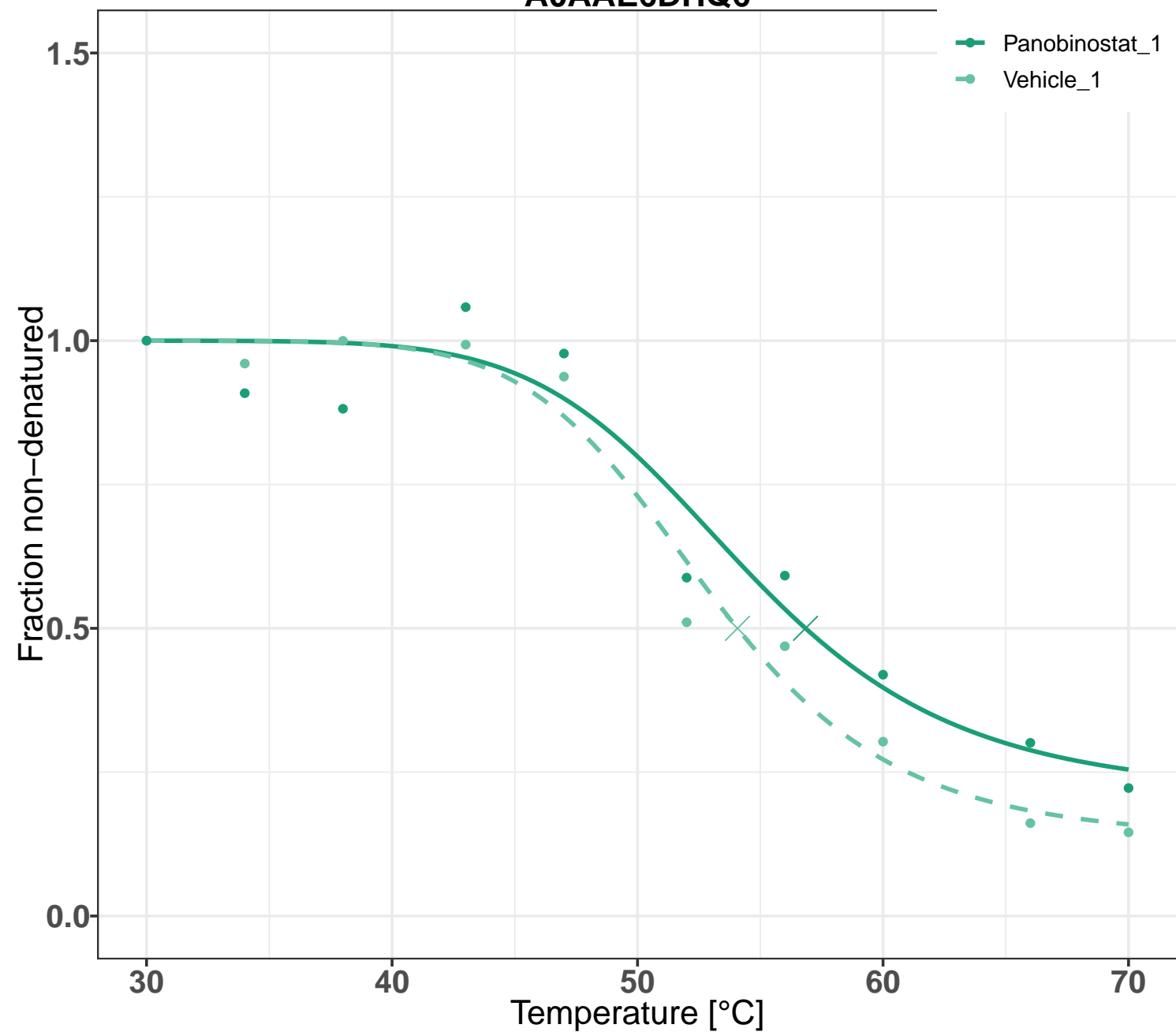

|                | meltPoint | slope  | plateau | R2   |
|----------------|-----------|--------|---------|------|
| Panobinostat_1 | 56.84     | -0.046 | 0.21    | 0.94 |
| Vehicle_1      | 54.07     | -0.058 | 0.13    | 0.98 |

Supplement: Supplementary file 2 — Supplementary Material 2 [file 41598_2026_35990_MOESM2_ESM.zip › AllTheTPPData/D40vD86/Panobinostat_Vignette/Melting_Curves/meltCurve_A0AAE6DHQ6.pdf]

A0AAF1D537

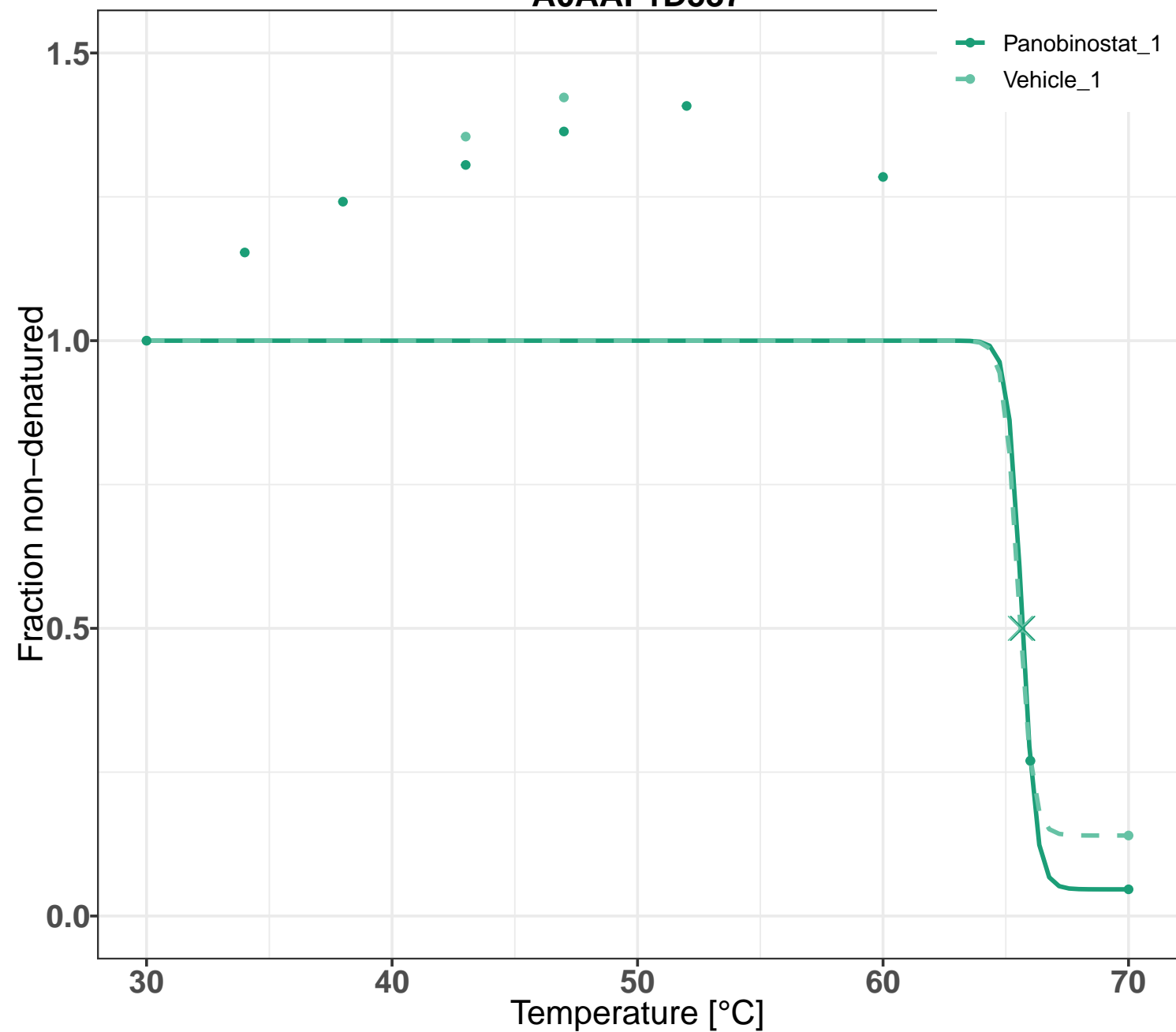

meltPoint

slope

plateau

R2

Panobinostat\_1

65.69

-0.83

0.05

0.58

Vehicle\_1

65.59

-0.75

0.14

-0.19

Supplement: Supplementary file 2 — Supplementary Material 2 [file 41598_2026_35990_MOESM2_ESM.zip › AllTheTPPData/D40vD86/Panobinostat_Vignette/Melting_Curves/meltCurve_A0AAF1D537.pdf]

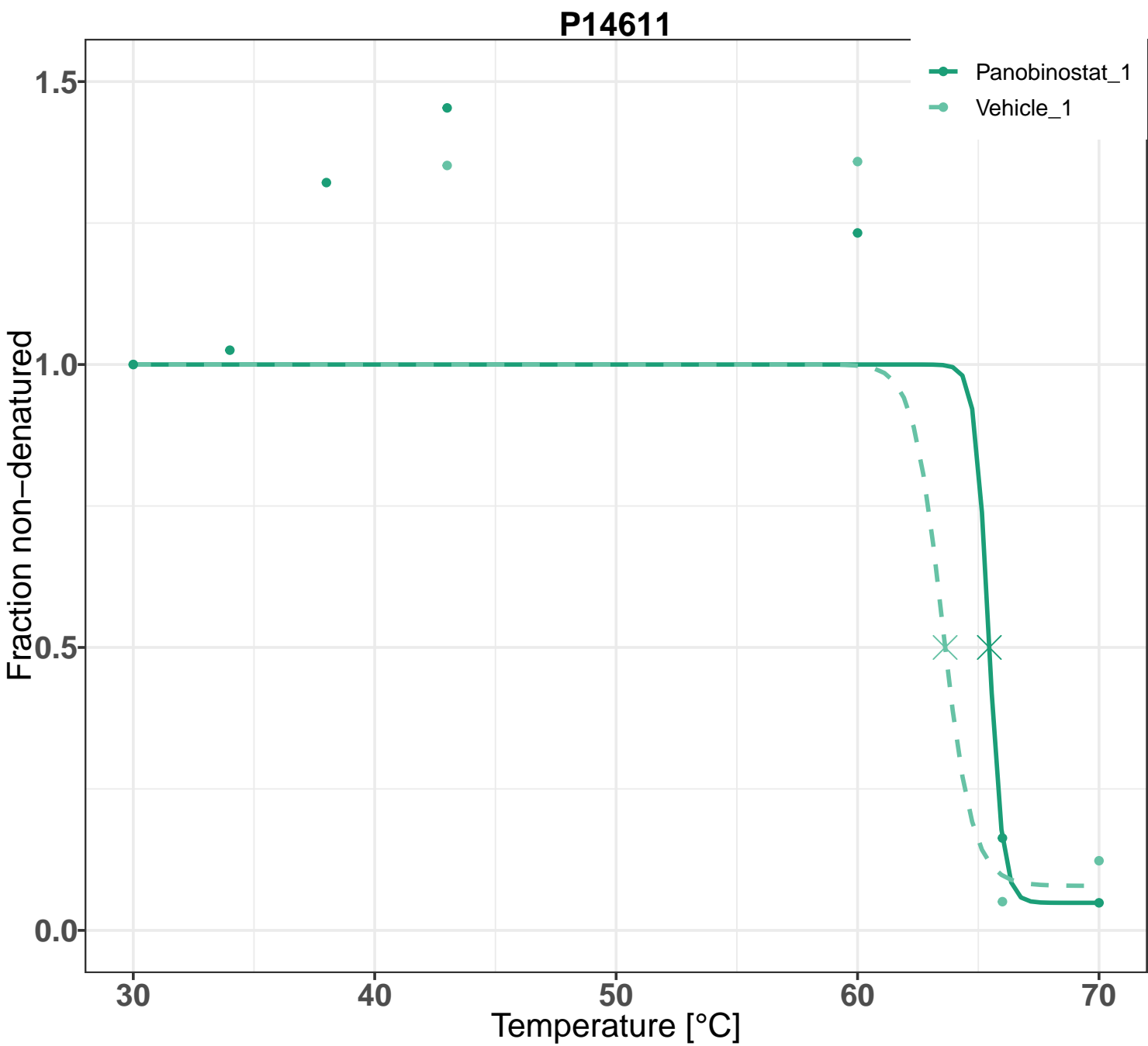

|                | meltPoint | slope | plateau | R2    |
|----------------|-----------|-------|---------|-------|
| Panobinostat_1 | 65.46     | -0.83 | 0.05    | 0.2   |
| Vehicle_1      | 63.63     | -0.38 | 0.08    | -0.15 |

Supplement: Supplementary file 2 — Supplementary Material 2 [file 41598_2026_35990_MOESM2_ESM.zip › AllTheTPPData/D40vD86/Panobinostat_Vignette/Melting_Curves/meltCurve_P14611.pdf]

# P14697

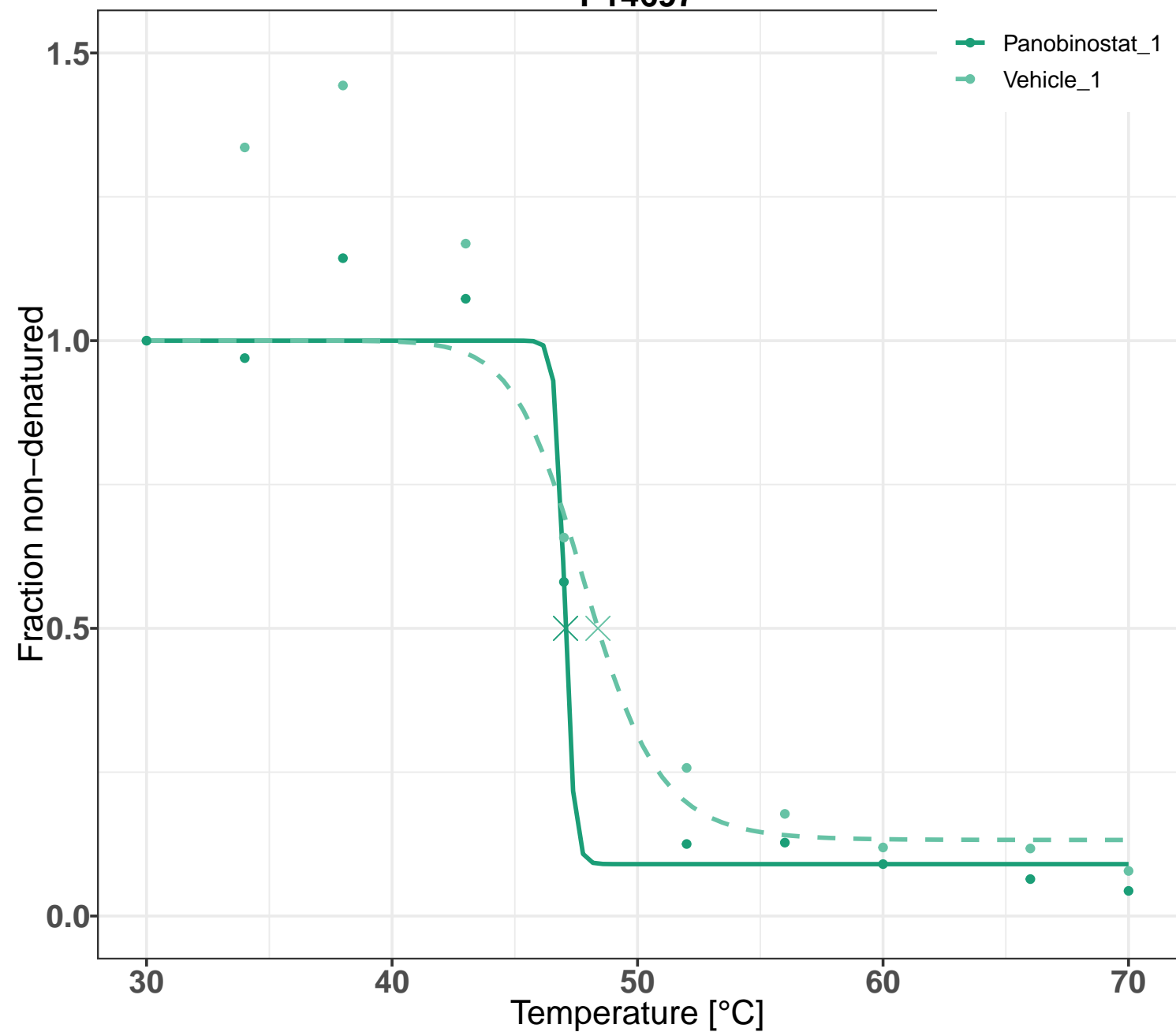

**meltPoint**

**slope**

**plateau**

**R2**

**Panobinostat\_1**

**47.07**

**-1.2**

**0.09**

**0.98**

**Vehicle\_1**

**48.39**

**-0.15**

**0.13**

**0.87**

Supplement: Supplementary file 2 — Supplementary Material 2 [file 41598_2026_35990_MOESM2_ESM.zip › AllTheTPPData/D40vD86/Panobinostat_Vignette/Melting_Curves/meltCurve_P14697.pdf]

# P23608

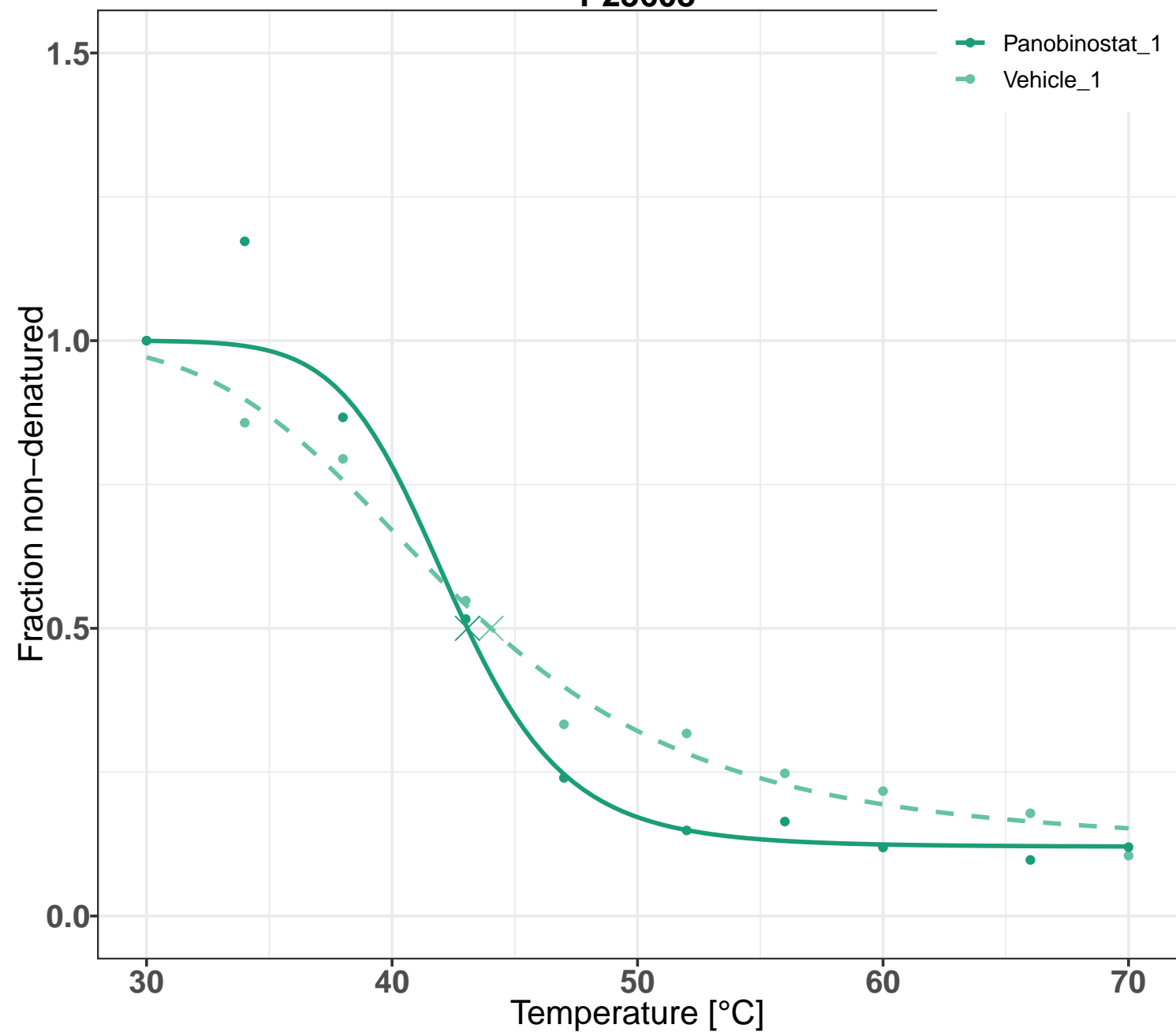

**meltPoint**

**slope**

**plateau**

**R2**

**Panobinostat\_1**

**43.07**

**-0.096**

**0.12**

**0.98**

**Vehicle\_1**

**44.03**

**-0.044**

**0.12**

**0.99**

Supplement: Supplementary file 2 — Supplementary Material 2 [file 41598_2026_35990_MOESM2_ESM.zip › AllTheTPPData/D40vD86/Panobinostat_Vignette/Melting_Curves/meltCurve_P23608.pdf]

# P27746

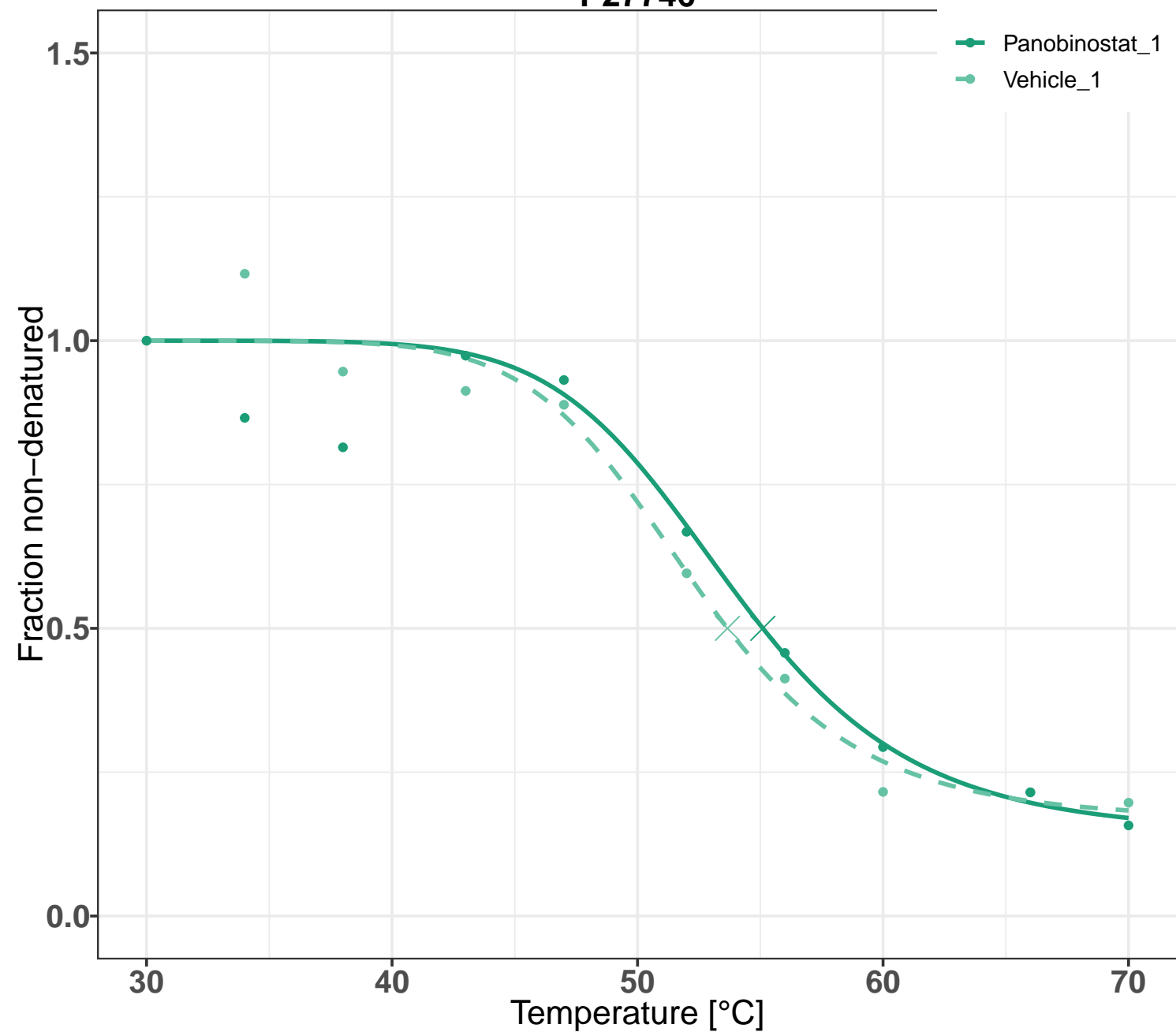

meltPoint

slope

plateau

R2

Panobinostat\_1

55.1

-0.059

0.14

0.95

Vehicle\_1

53.66

-0.062

0.17

0.98

Supplement: Supplementary file 2 — Supplementary Material 2 [file 41598_2026_35990_MOESM2_ESM.zip › AllTheTPPData/D40vD86/Panobinostat_Vignette/Melting_Curves/meltCurve_P27746.pdf]

# P28613

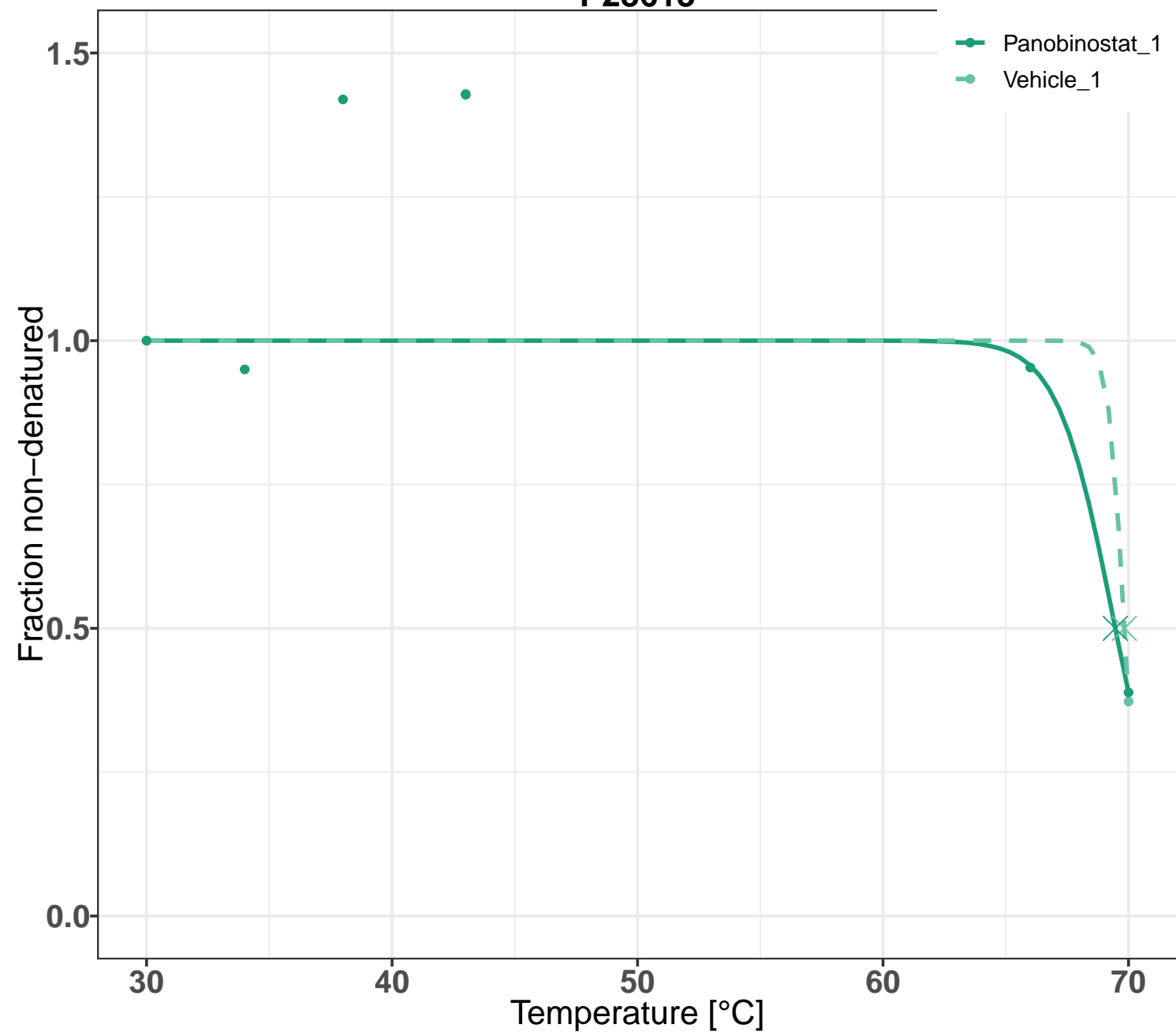

|                | meltPoint | slope | plateau | R2    |
|----------------|-----------|-------|---------|-------|
| Panobinostat_1 | 69.46     | -0.21 | 0       | -0.28 |
| Vehicle_1      | 69.83     | -0.77 | 0       | -0.94 |

Supplement: Supplementary file 2 — Supplementary Material 2 [file 41598_2026_35990_MOESM2_ESM.zip › AllTheTPPData/D40vD86/Panobinostat_Vignette/Melting_Curves/meltCurve_P28613.pdf]

# P31638

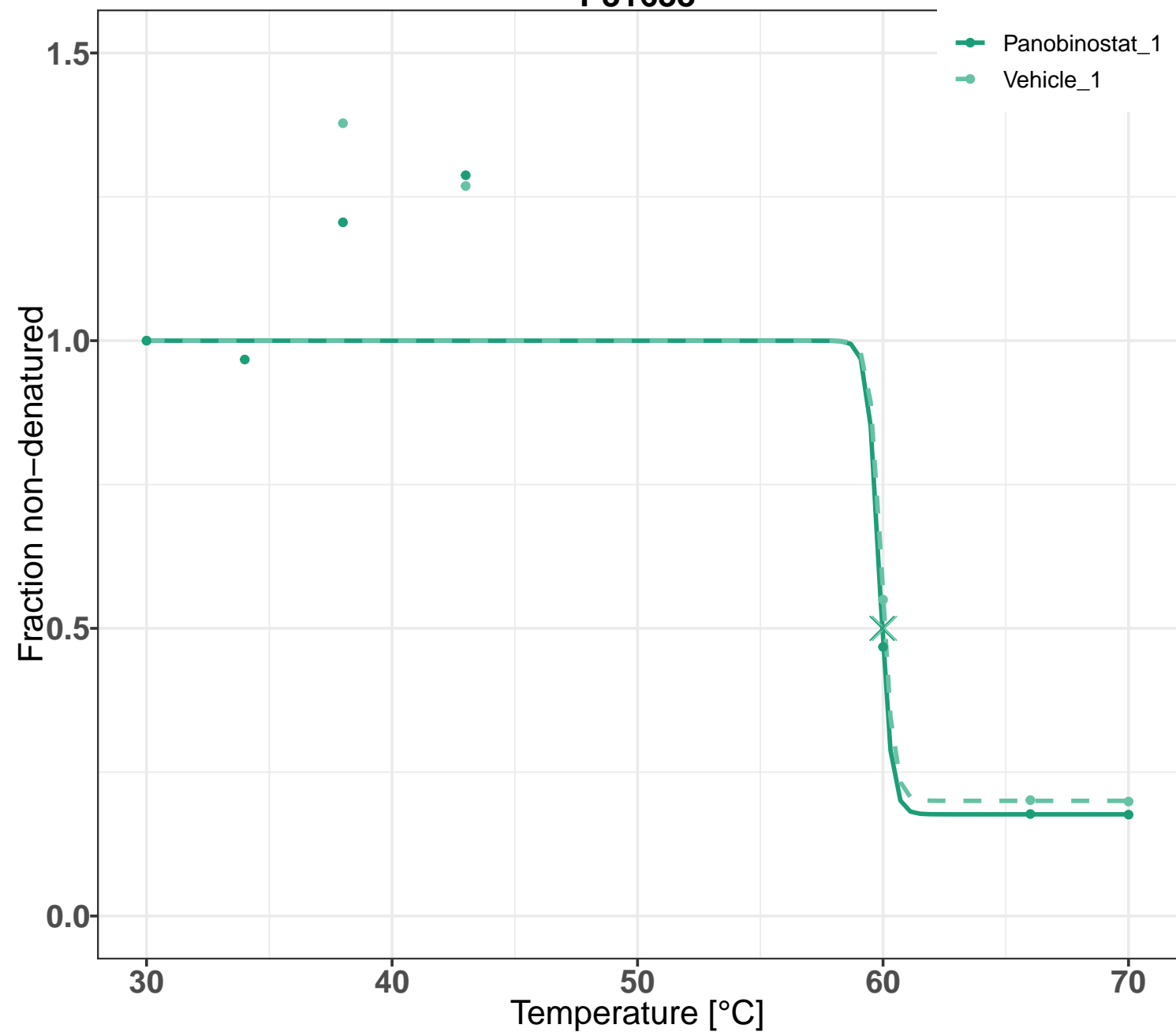

|                | meltPoint | slope | plateau | R2   |
|----------------|-----------|-------|---------|------|
| Panobinostat_1 | 59.96     | -0.86 | 0.18    | 0.51 |
| Vehicle_1      | 60.06     | -0.83 | 0.2     | 0.07 |

Supplement: Supplementary file 2 — Supplementary Material 2 [file 41598_2026_35990_MOESM2_ESM.zip › AllTheTPPData/D40vD86/Panobinostat_Vignette/Melting_Curves/meltCurve_P31638.pdf]

# P31891

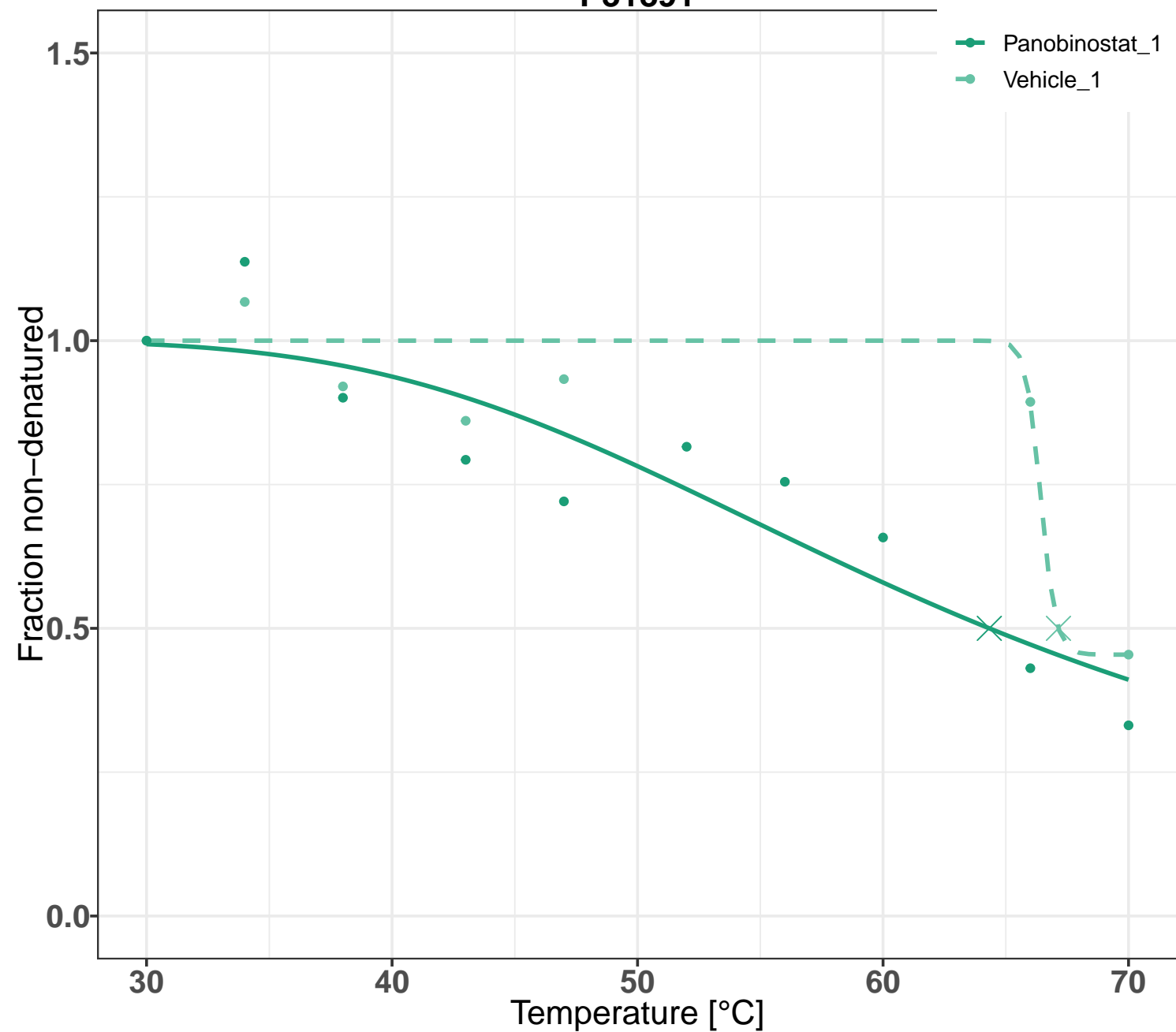

meltPoint

slope

plateau

R2

Panobinostat\_1

64.33

-0.021

0

0.85

Vehicle\_1

67.15

-0.46

0.45

0.03

Supplement: Supplementary file 2 — Supplementary Material 2 [file 41598_2026_35990_MOESM2_ESM.zip › AllTheTPPData/D40vD86/Panobinostat_Vignette/Melting_Curves/meltCurve_P31891.pdf]

# P39662

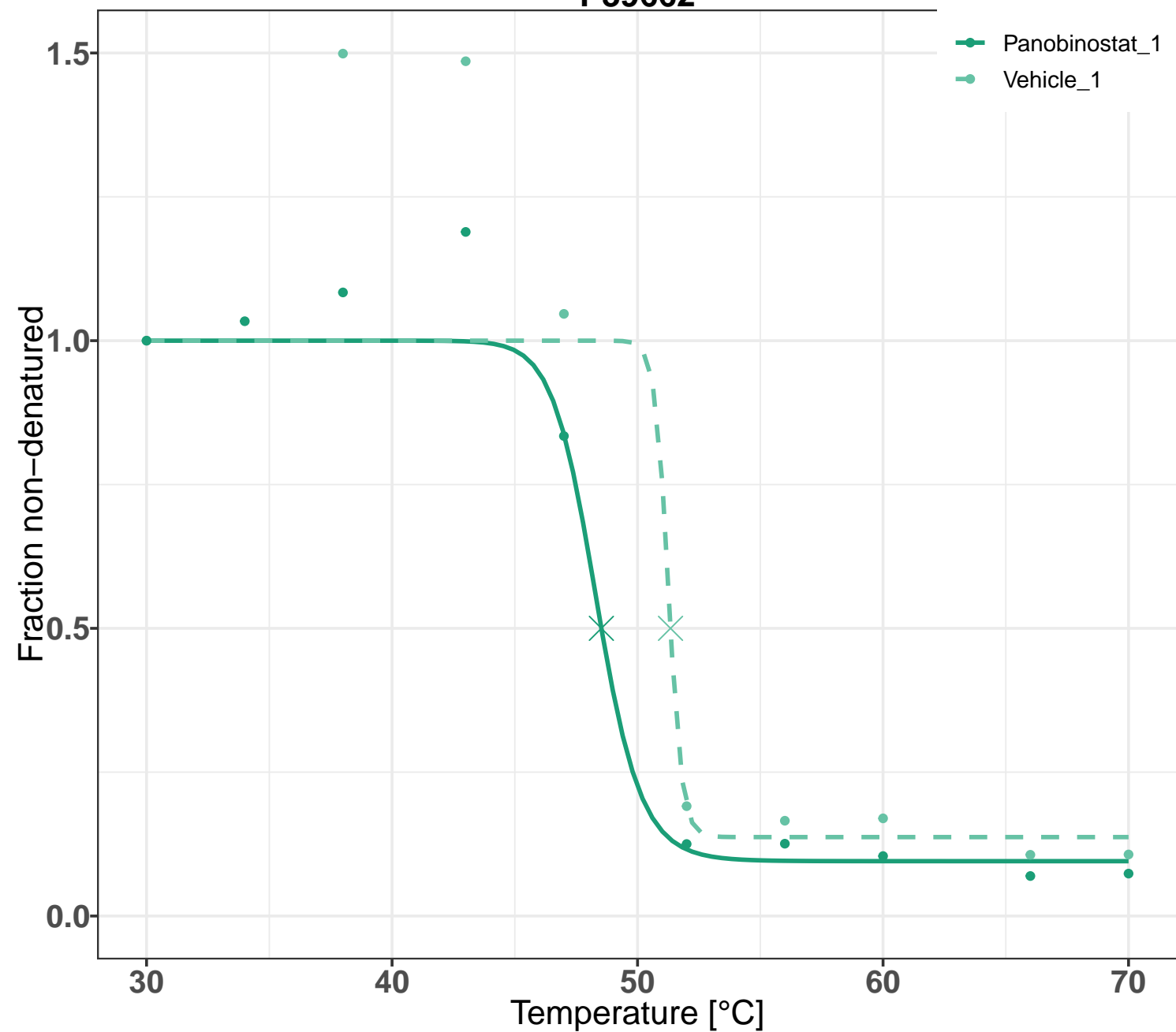

meltPoint

slope

plateau

R2

Panobinostat\_1

48.53

-0.25

0.1

0.98

Vehicle\_1

51.34

-0.8

0.14

0.74

Supplement: Supplementary file 2 — Supplementary Material 2 [file 41598_2026_35990_MOESM2_ESM.zip › AllTheTPPData/D40vD86/Panobinostat_Vignette/Melting_Curves/meltCurve_P39662.pdf]

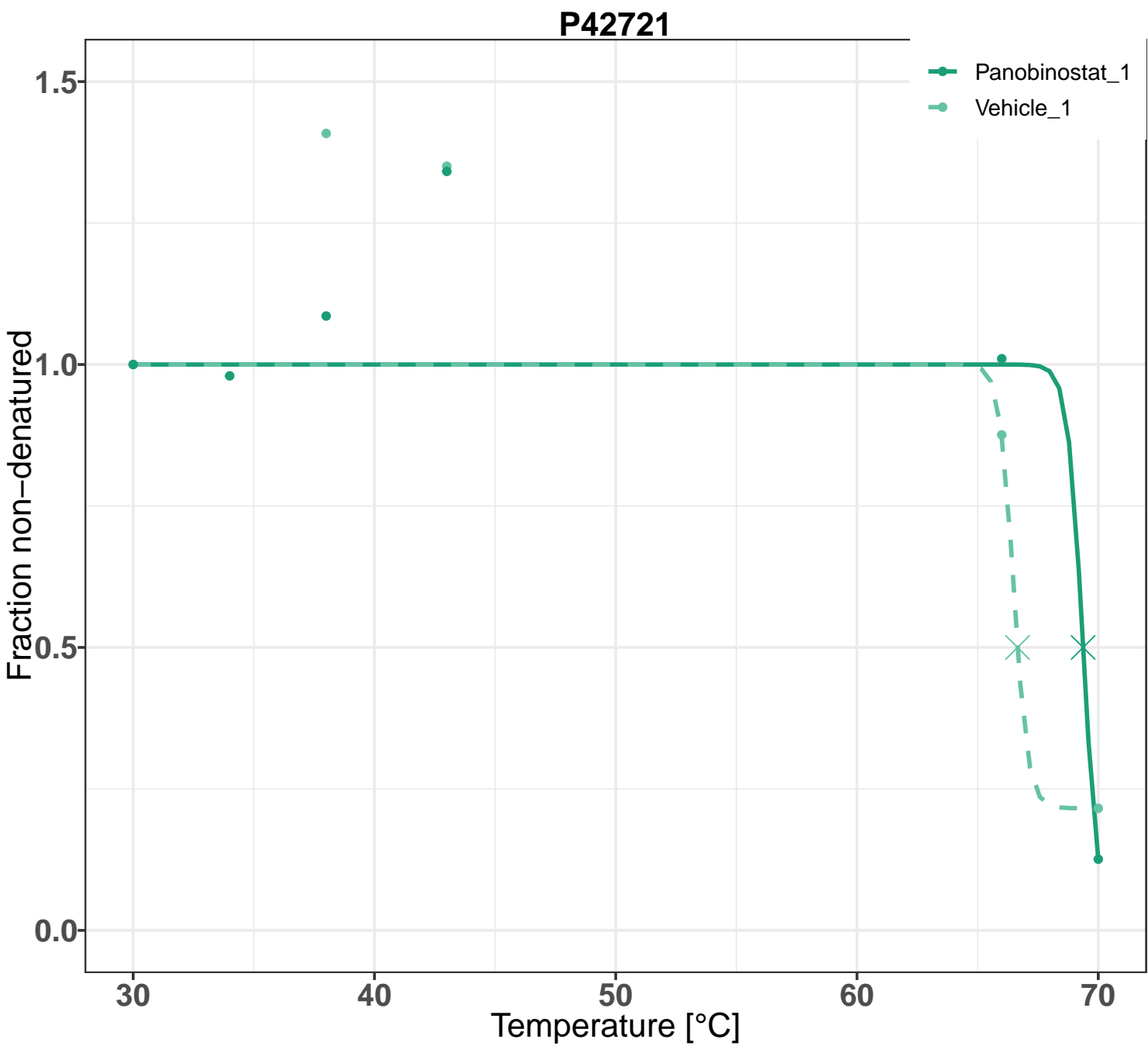

|                | meltPoint | slope | plateau | R2    |
|----------------|-----------|-------|---------|-------|
| Panobinostat_1 | 69.37     | -0.78 | 0       | -0.13 |
| Vehicle_1      | 66.66     | -0.67 | 0.22    | -0.52 |

Supplement: Supplementary file 2 — Supplementary Material 2 [file 41598_2026_35990_MOESM2_ESM.zip › AllTheTPPData/D40vD86/Panobinostat_Vignette/Melting_Curves/meltCurve_P42721.pdf]

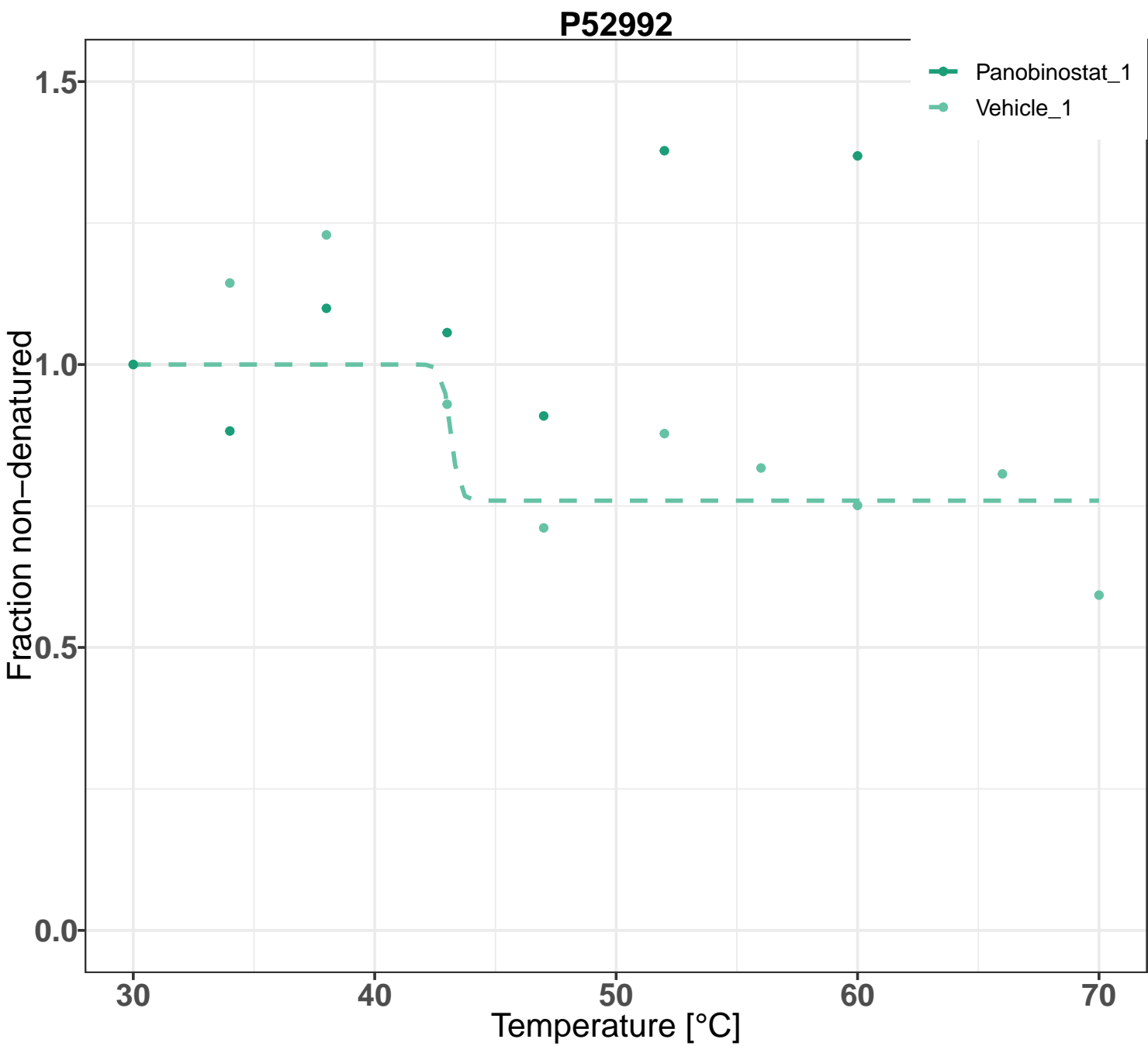

|                | meltPoint | slope | plateau | R2   |
|----------------|-----------|-------|---------|------|
| Panobinostat_1 | –         | –     | –       | –    |
| Vehicle_1      | –         | –0.35 | 0.76    | 0.64 |

Supplement: Supplementary file 2 — Supplementary Material 2 [file 41598_2026_35990_MOESM2_ESM.zip › AllTheTPPData/D40vD86/Panobinostat_Vignette/Melting_Curves/meltCurve_P52992.pdf]

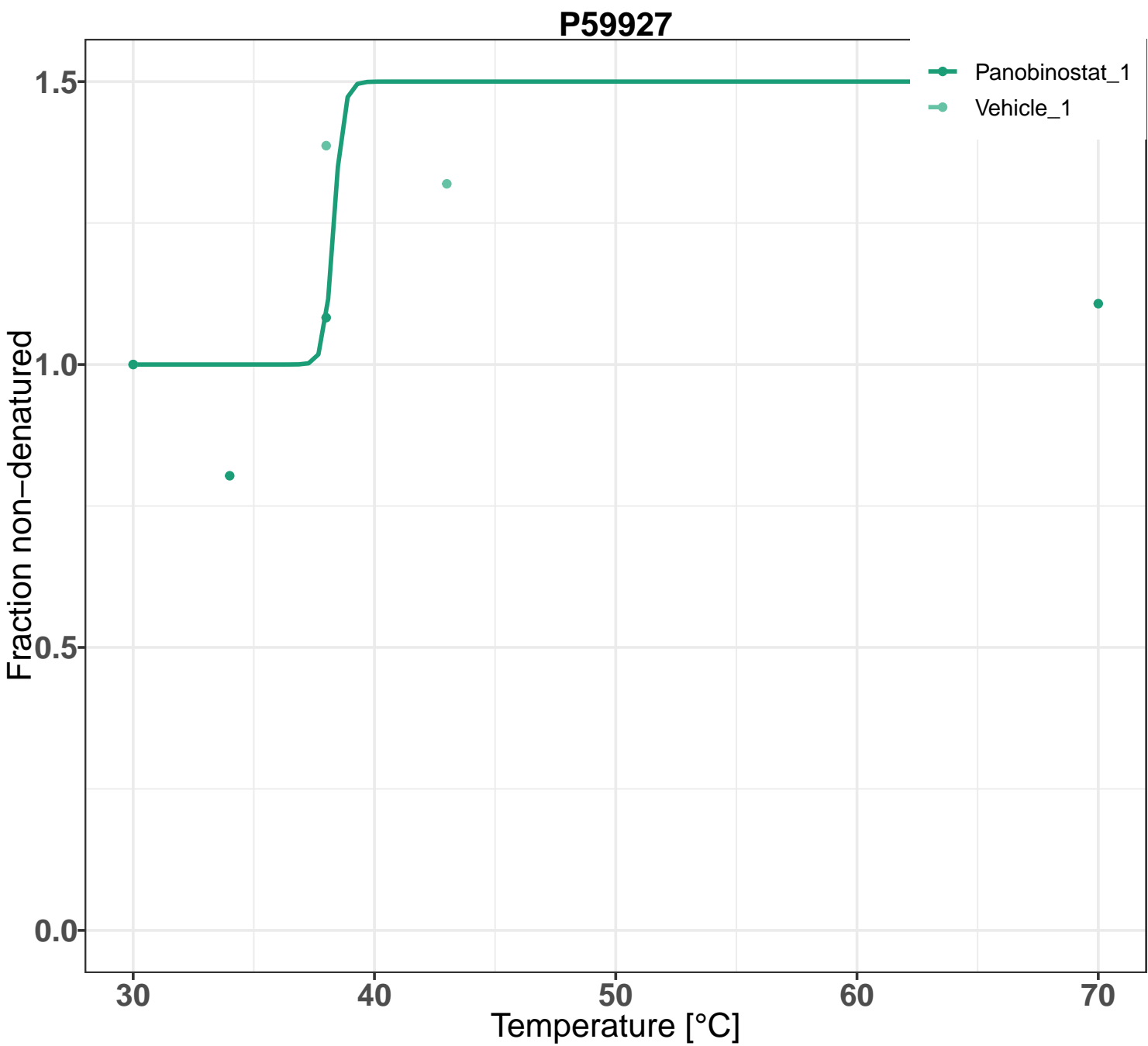

|                | meltPoint | slope | plateau | R2    |
|----------------|-----------|-------|---------|-------|
| Panobinostat_1 | —         | 0.63  | 1.5     | -0.06 |
| Vehicle_1      | —         | —     | —       | —     |

Supplement: Supplementary file 2 — Supplementary Material 2 [file 41598_2026_35990_MOESM2_ESM.zip › AllTheTPPData/D40vD86/Panobinostat_Vignette/Melting_Curves/meltCurve_P59927.pdf]

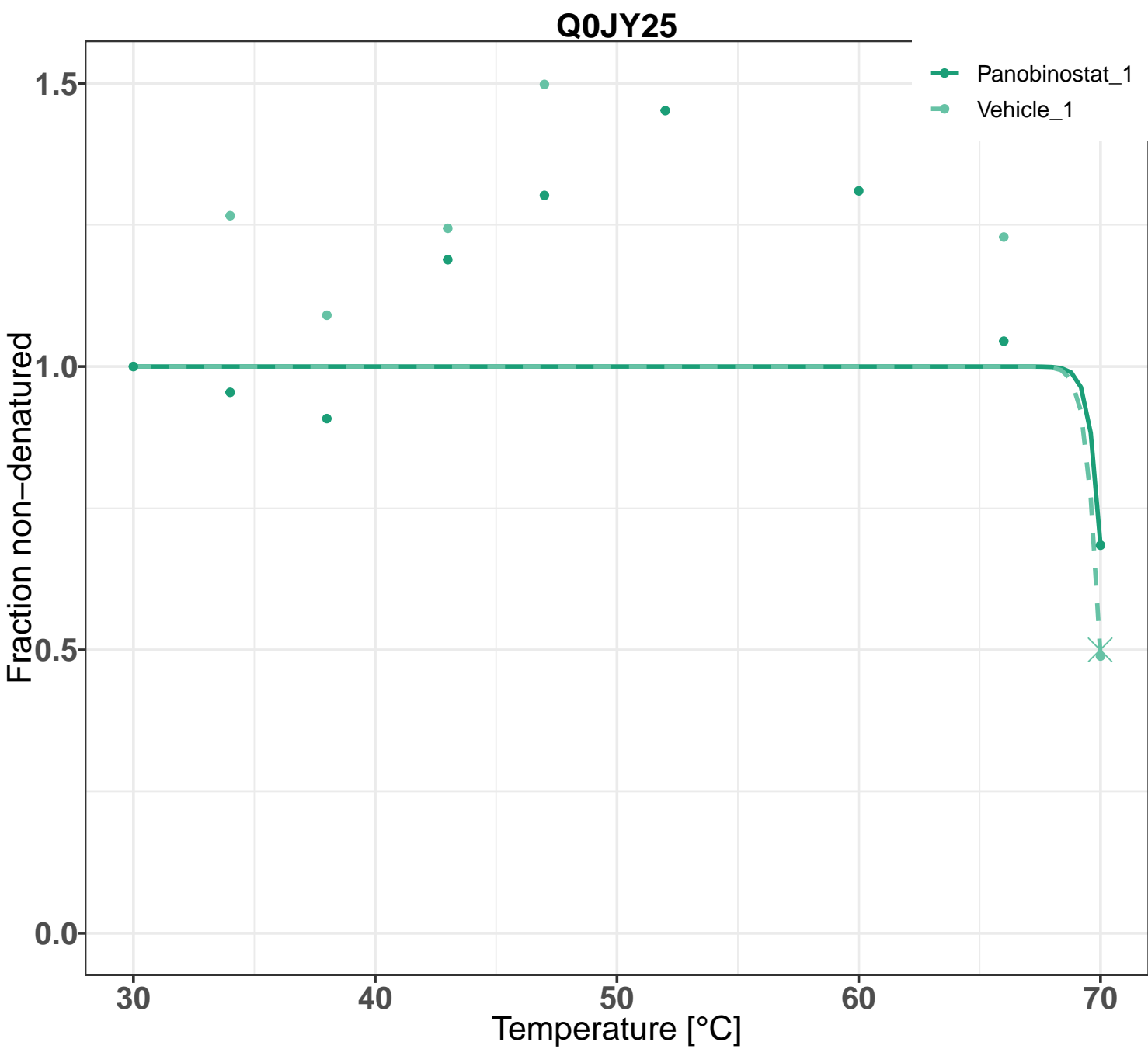

|                | meltPoint | slope | plateau | R2    |
|----------------|-----------|-------|---------|-------|
| Panobinostat_1 | –         | –     | 0       | –0.17 |
| Vehicle_1      | 69.99     | –0.77 | 0       | –0.52 |

Supplement: Supplementary file 2 — Supplementary Material 2 [file 41598_2026_35990_MOESM2_ESM.zip › AllTheTPPData/D40vD86/Panobinostat_Vignette/Melting_Curves/meltCurve_Q0JY25.pdf]

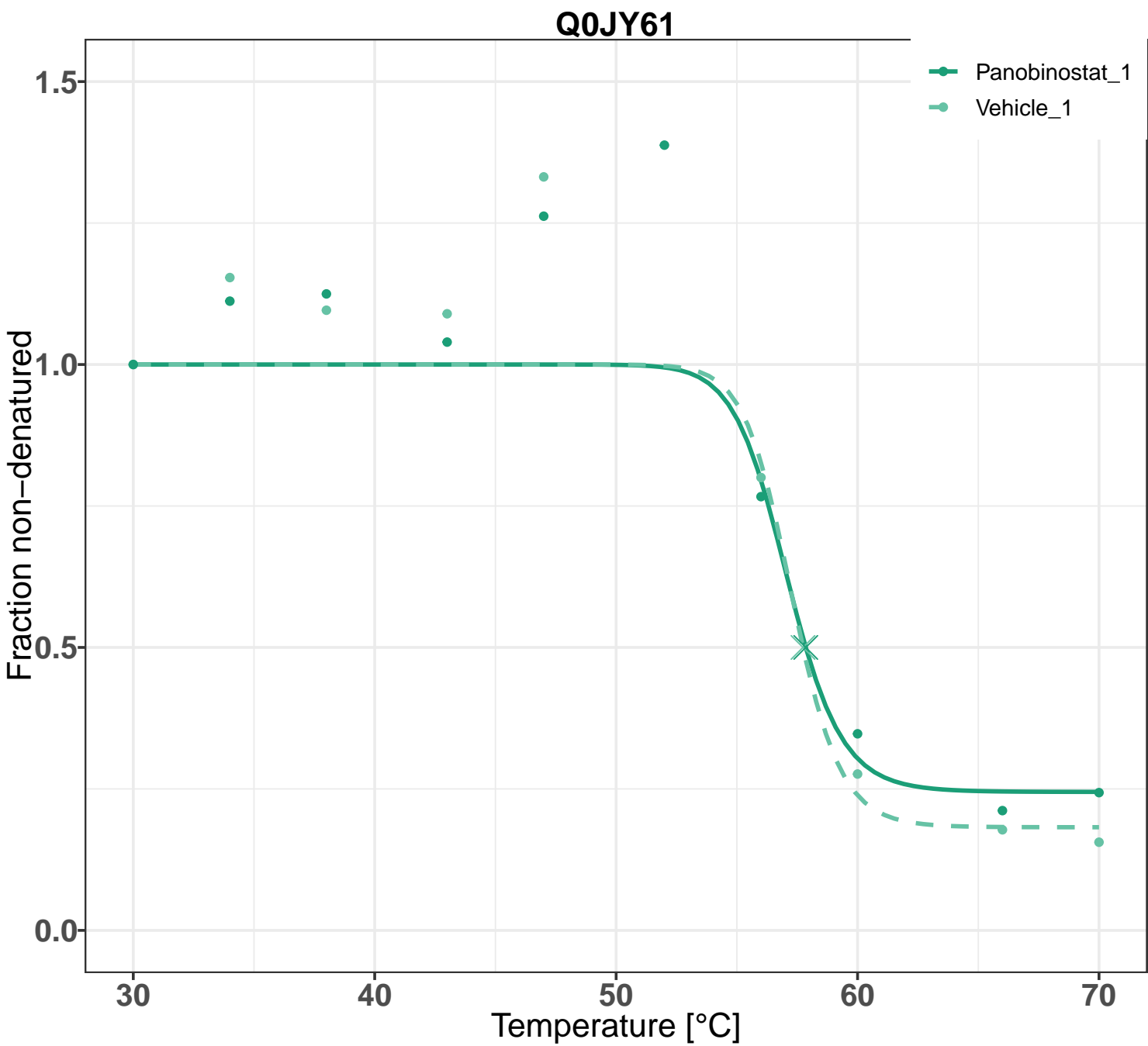

|                | meltPoint | slope | plateau | R2   |
|----------------|-----------|-------|---------|------|
| Panobinostat_1 | 57.85     | -0.17 | 0.24    | 0.85 |
| Vehicle_1      | 57.74     | -0.21 | 0.18    | 0.73 |

Supplement: Supplementary file 2 — Supplementary Material 2 [file 41598_2026_35990_MOESM2_ESM.zip › AllTheTPPData/D40vD86/Panobinostat_Vignette/Melting_Curves/meltCurve_Q0JY61.pdf]

Q0JY94

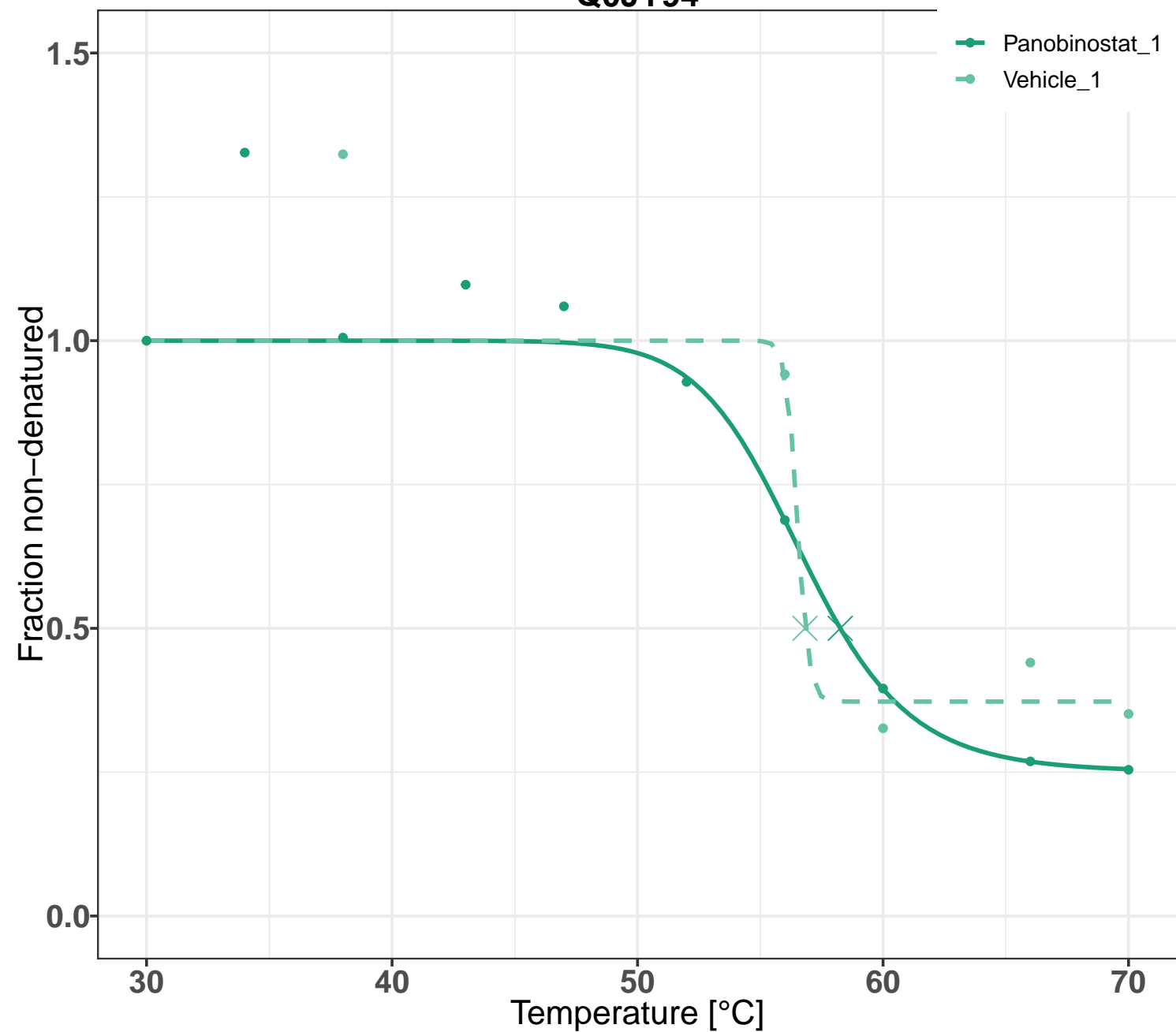

meltPoint

slope

plateau

R2

Panobinostat\_1

58.26

-0.087

0.25

0.91

Vehicle\_1

56.82

-0.69

0.37

0.44

Supplement: Supplementary file 2 — Supplementary Material 2 [file 41598_2026_35990_MOESM2_ESM.zip › AllTheTPPData/D40vD86/Panobinostat_Vignette/Melting_Curves/meltCurve_Q0JY94.pdf]

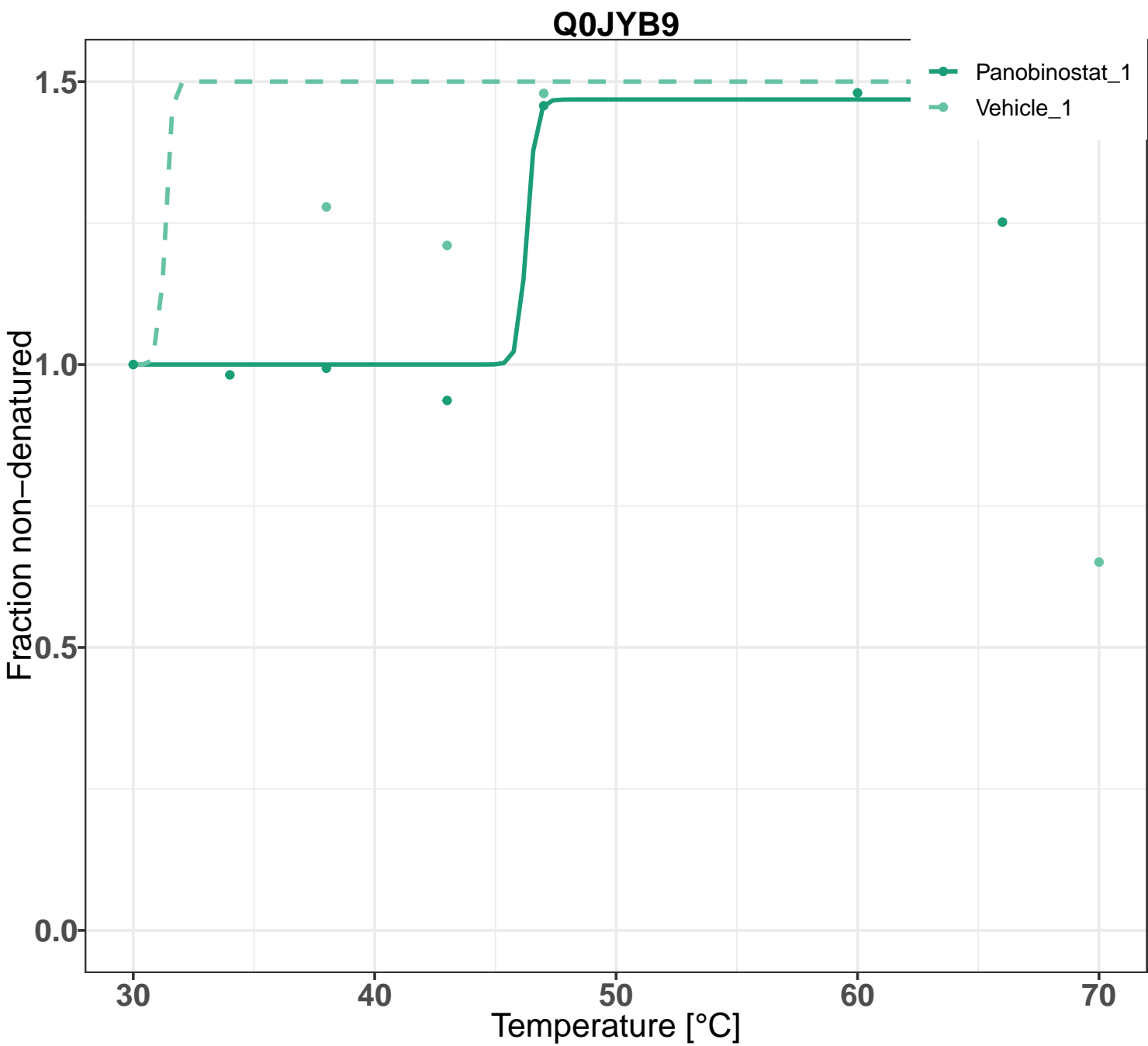

|                | meltPoint | slope | plateau | R2   |
|----------------|-----------|-------|---------|------|
| Panobinostat_1 | –         | 0.63  | 1.47    | 0.84 |
| Vehicle_1      | –         | 1     | 1.5     | 0.13 |

Supplement: Supplementary file 2 — Supplementary Material 2 [file 41598_2026_35990_MOESM2_ESM.zip › AllTheTPPData/D40vD86/Panobinostat_Vignette/Melting_Curves/meltCurve_Q0JYB9.pdf]

# Q0JYF3

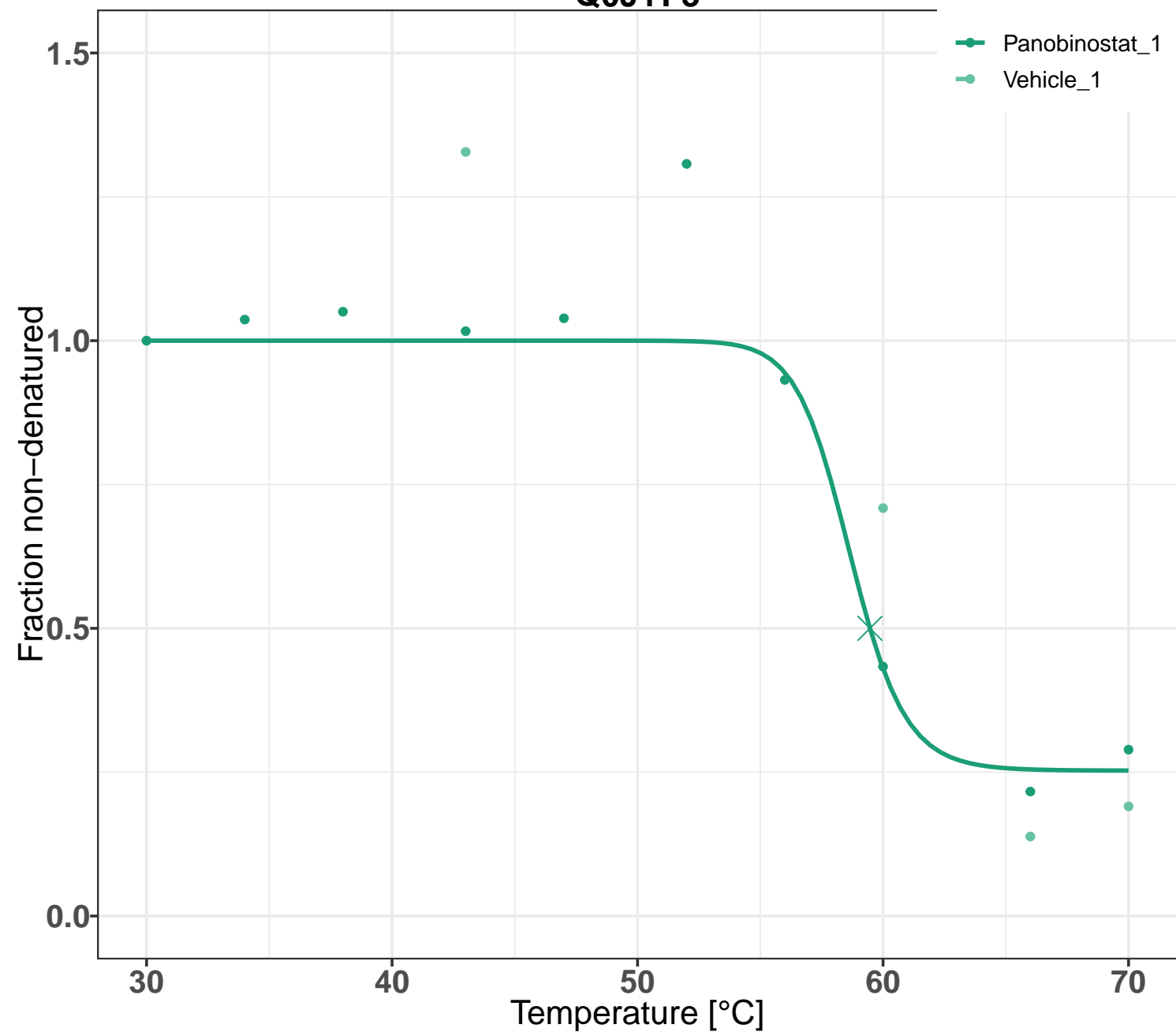

meltPoint

slope

plateau

R2

Panobinostat\_1

59.47

-0.17

0.25

0.92

Vehicle\_1

—

—

—

—

Supplement: Supplementary file 2 — Supplementary Material 2 [file 41598_2026_35990_MOESM2_ESM.zip › AllTheTPPData/D40vD86/Panobinostat_Vignette/Melting_Curves/meltCurve_Q0JYF3.pdf]

# Q0JYK2

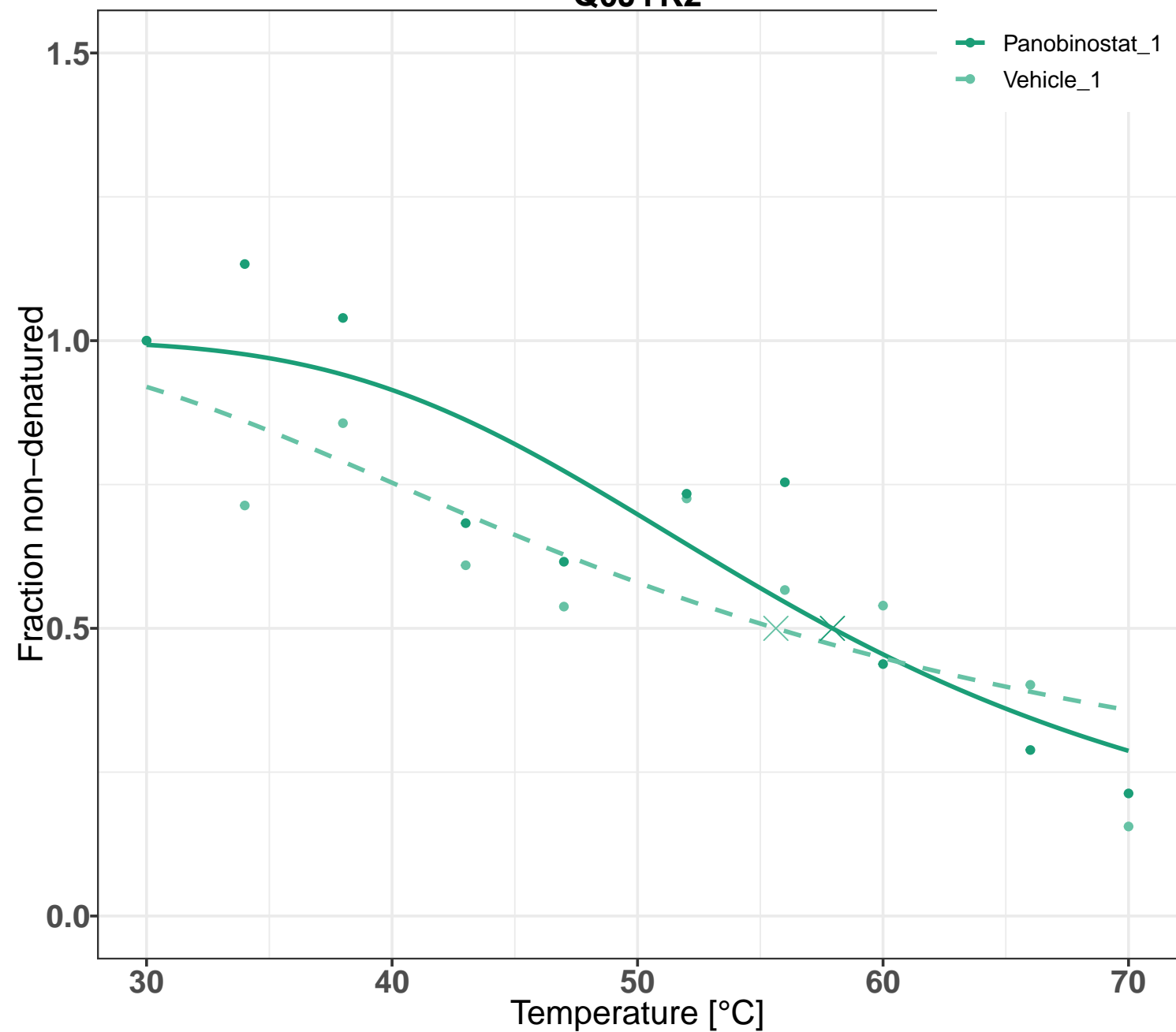

**meltPoint**

**slope**

**plateau**

**R2**

**Panobinostat\_1**

**57.94**

**-0.026**

**0**

**0.83**

**Vehicle\_1**

**55.63**

**-0.018**

**0**

**0.73**

Supplement: Supplementary file 2 — Supplementary Material 2 [file 41598_2026_35990_MOESM2_ESM.zip › AllTheTPPData/D40vD86/Panobinostat_Vignette/Melting_Curves/meltCurve_Q0JYK2.pdf]

# Q0JYZ2

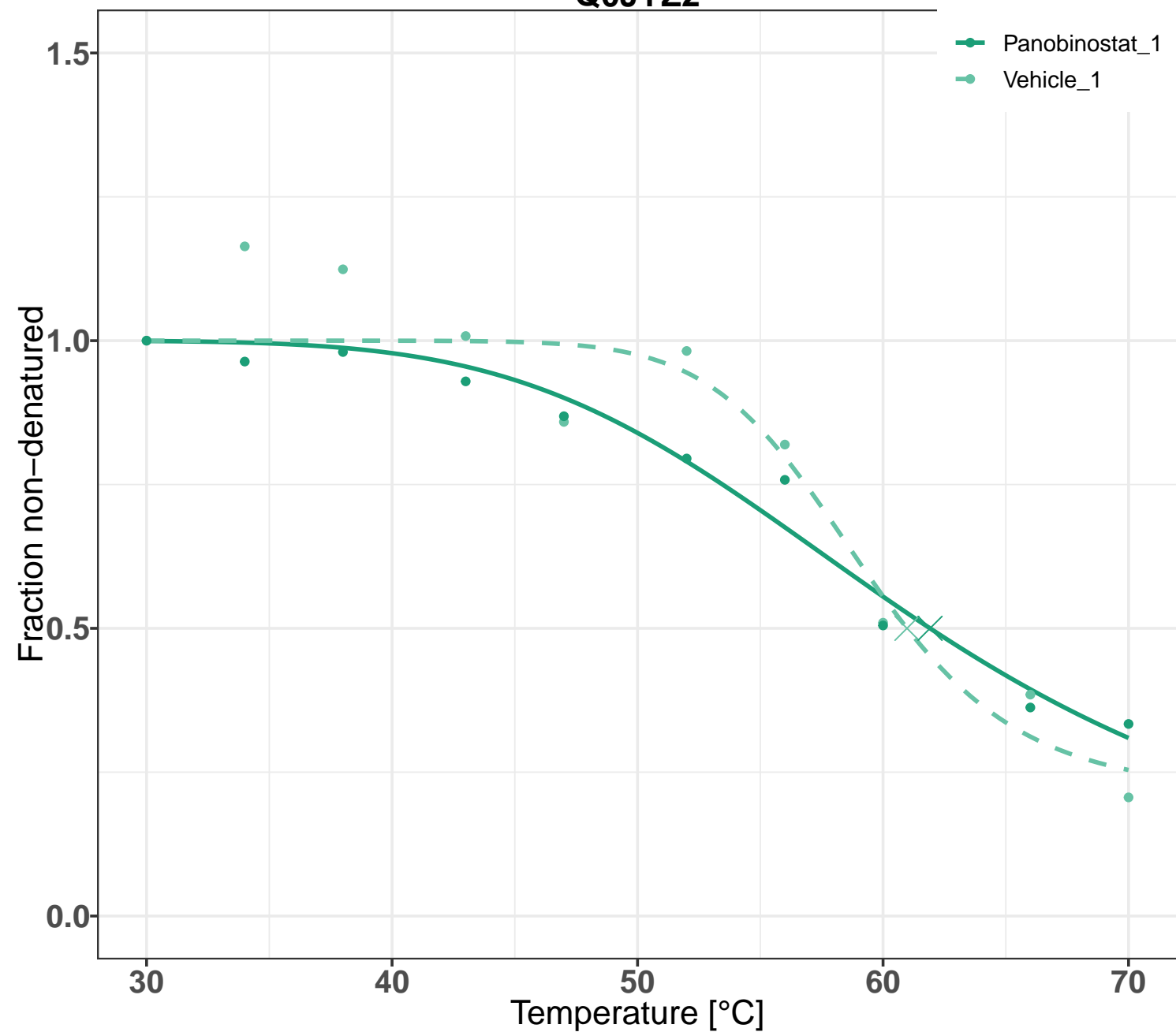

|                | meltPoint | slope  | plateau | R2   |
|----------------|-----------|--------|---------|------|
| Panobinostat_1 | 61.91     | -0.03  | 0       | 0.98 |
| Vehicle_1      | 60.97     | -0.063 | 0.21    | 0.93 |

Supplement: Supplementary file 2 — Supplementary Material 2 [file 41598_2026_35990_MOESM2_ESM.zip › AllTheTPPData/D40vD86/Panobinostat_Vignette/Melting_Curves/meltCurve_Q0JYZ2.pdf]

# Q0JZ03

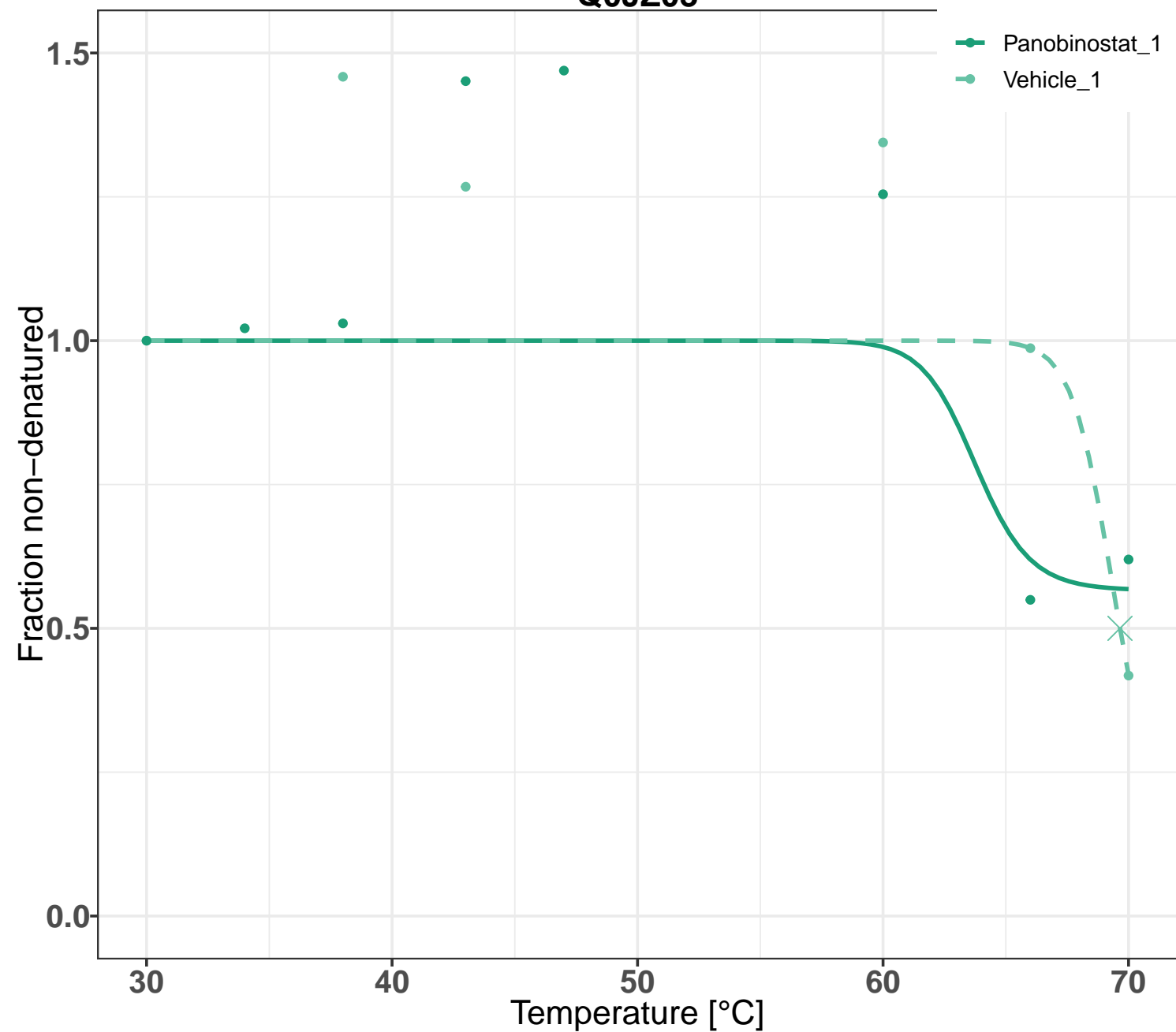

meltPoint

slope

plateau

R2

Panobinostat\_1

–

–0.099

0.57

0.09

Vehicle\_1

69.65

–0.25

0.15

–0.52

Supplement: Supplementary file 2 — Supplementary Material 2 [file 41598_2026_35990_MOESM2_ESM.zip › AllTheTPPData/D40vD86/Panobinostat_Vignette/Melting_Curves/meltCurve_Q0JZ03.pdf]

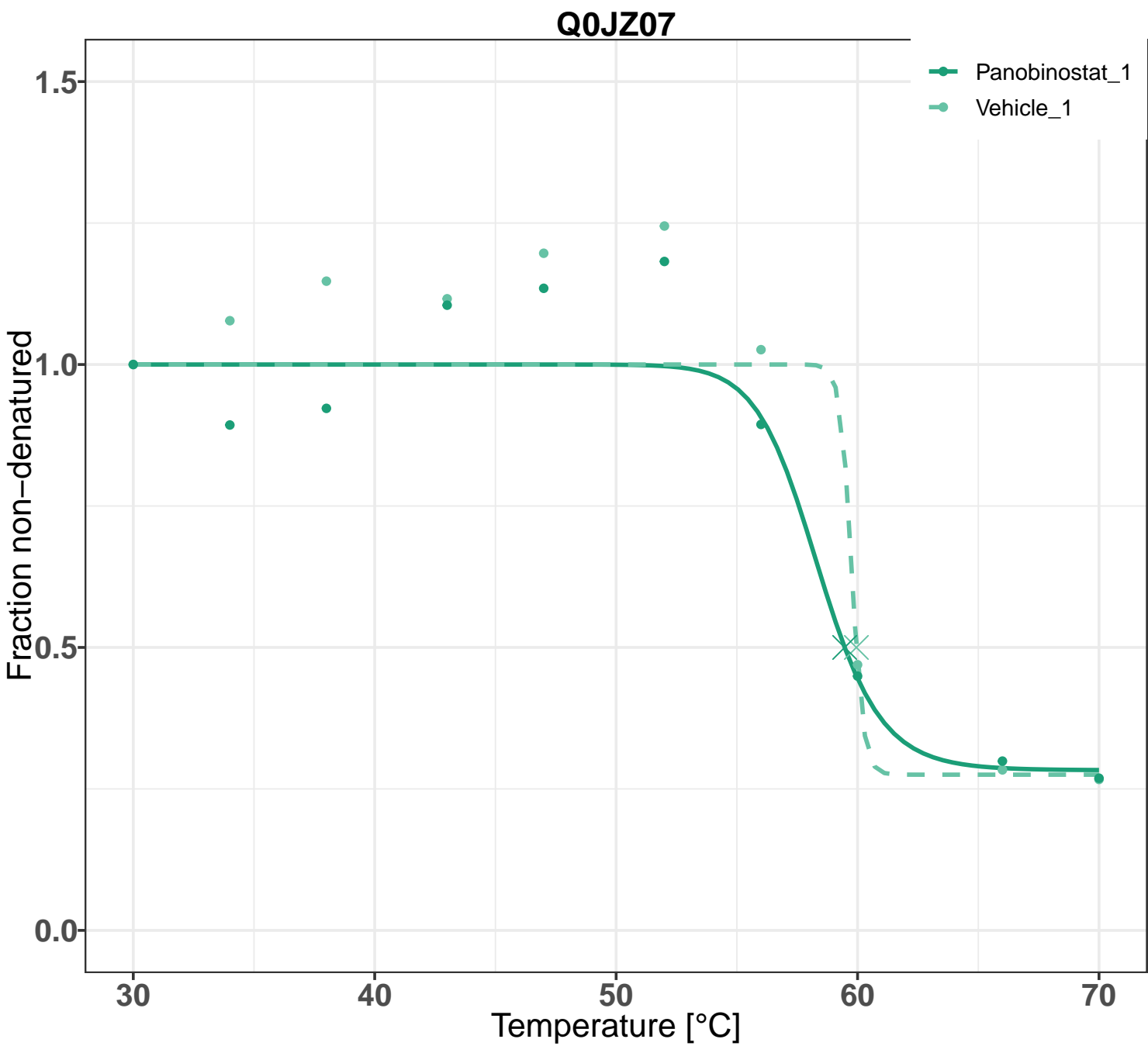

|                | meltpoint | slope | plateau | R2   |
|----------------|-----------|-------|---------|------|
| Panobinostat_1 | 59.47     | -0.14 | 0.28    | 0.92 |
| Vehicle_1      | 59.95     | -0.76 | 0.28    | 0.89 |

Supplement: Supplementary file 2 — Supplementary Material 2 [file 41598_2026_35990_MOESM2_ESM.zip › AllTheTPPData/D40vD86/Panobinostat_Vignette/Melting_Curves/meltCurve_Q0JZ07.pdf]

# Q0JZ09

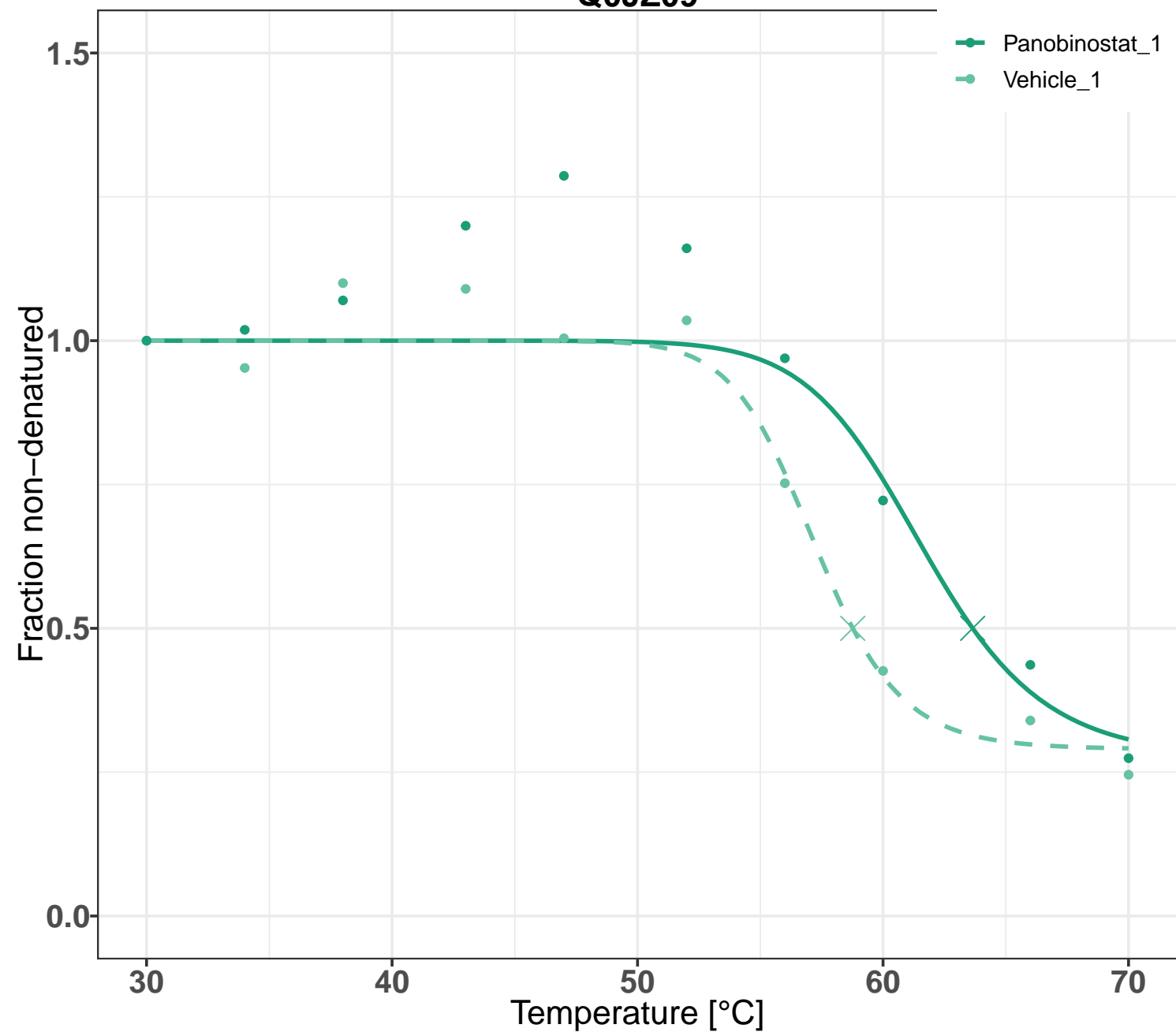

|                | meltPoint | slope  | plateau | R2   |
|----------------|-----------|--------|---------|------|
| Panobinostat_1 | 63.65     | -0.075 | 0.27    | 0.84 |
| Vehicle_1      | 58.77     | -0.1   | 0.29    | 0.97 |

Supplement: Supplementary file 2 — Supplementary Material 2 [file 41598_2026_35990_MOESM2_ESM.zip › AllTheTPPData/D40vD86/Panobinostat_Vignette/Melting_Curves/meltCurve_Q0JZ09.pdf]

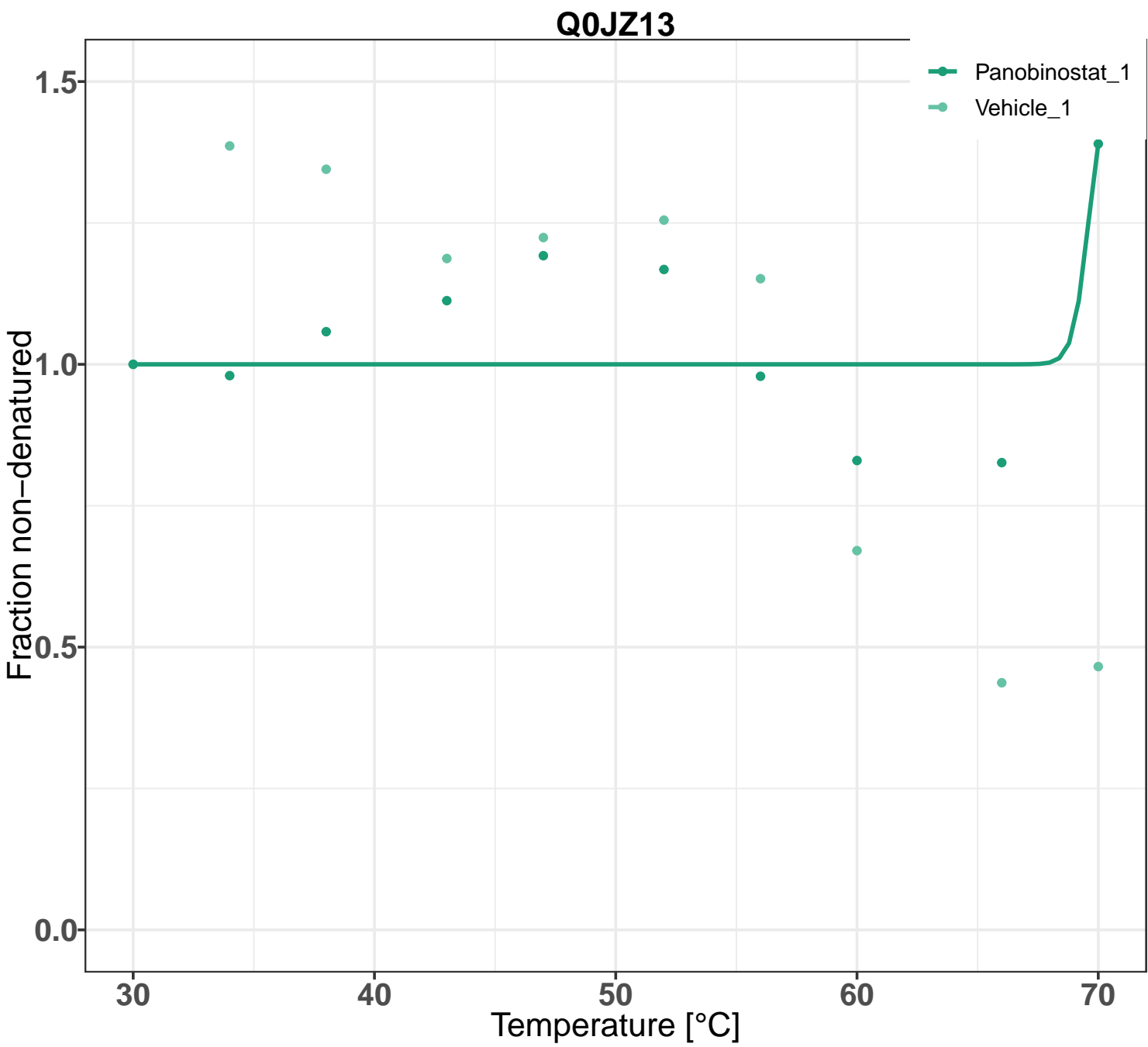

|                | meltPoint | slope | plateau | R2   |
|----------------|-----------|-------|---------|------|
| Panobinostat_1 | —         | 0.39  | 1.5     | 0.47 |
| Vehicle_1      | —         | —     | —       | —    |

Supplement: Supplementary file 2 — Supplementary Material 2 [file 41598_2026_35990_MOESM2_ESM.zip › AllTheTPPData/D40vD86/Panobinostat_Vignette/Melting_Curves/meltCurve_Q0JZ13.pdf]

# Q0JZG6

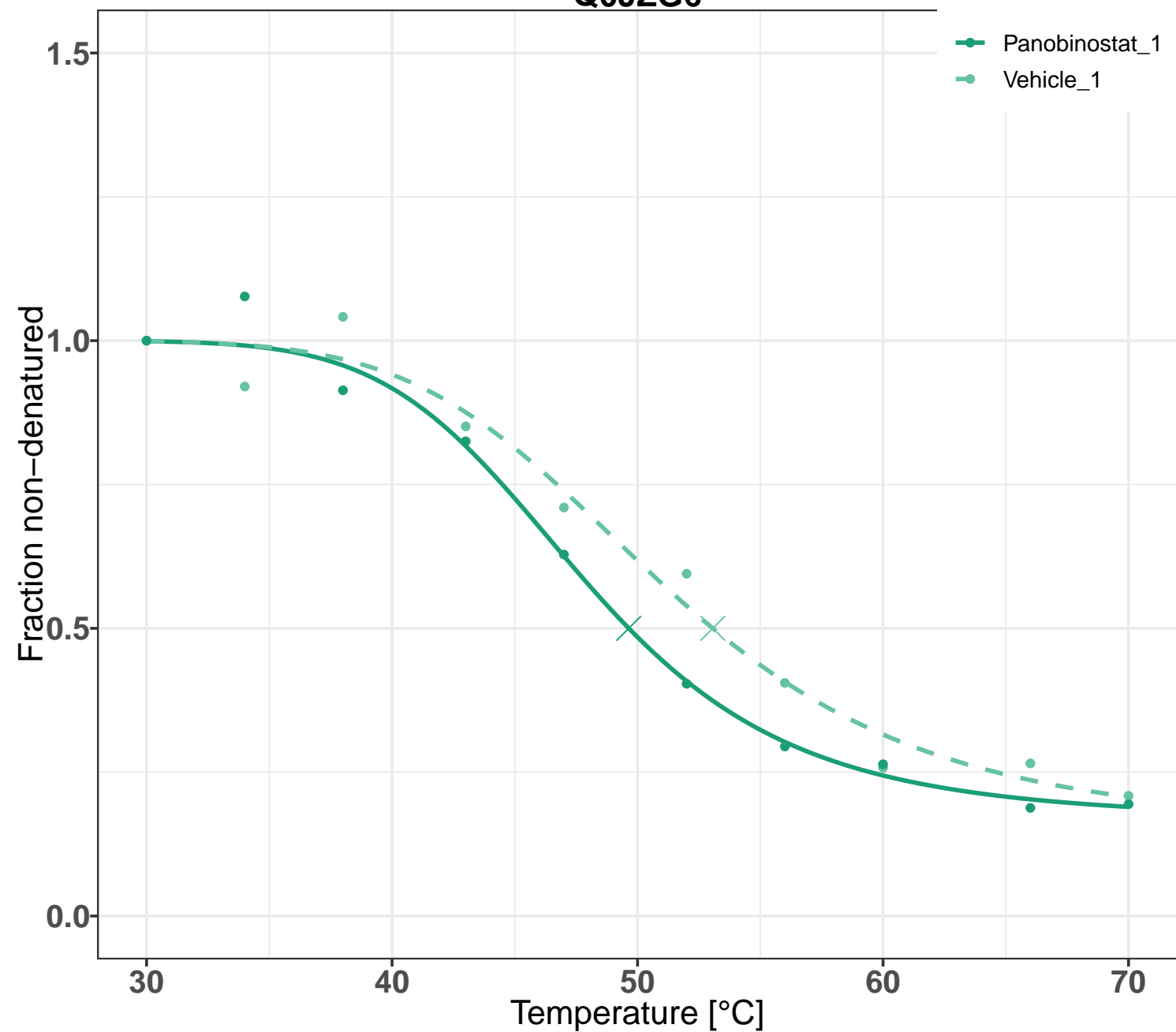

meltPoint

slope

plateau

R2

Panobinostat\_1

49.64

-0.05

0.17

0.99

Vehicle\_1

53.07

-0.041

0.14

0.98

Supplement: Supplementary file 2 — Supplementary Material 2 [file 41598_2026_35990_MOESM2_ESM.zip › AllTheTPPData/D40vD86/Panobinostat_Vignette/Melting_Curves/meltCurve_Q0JZG6.pdf]

Q0JZI5

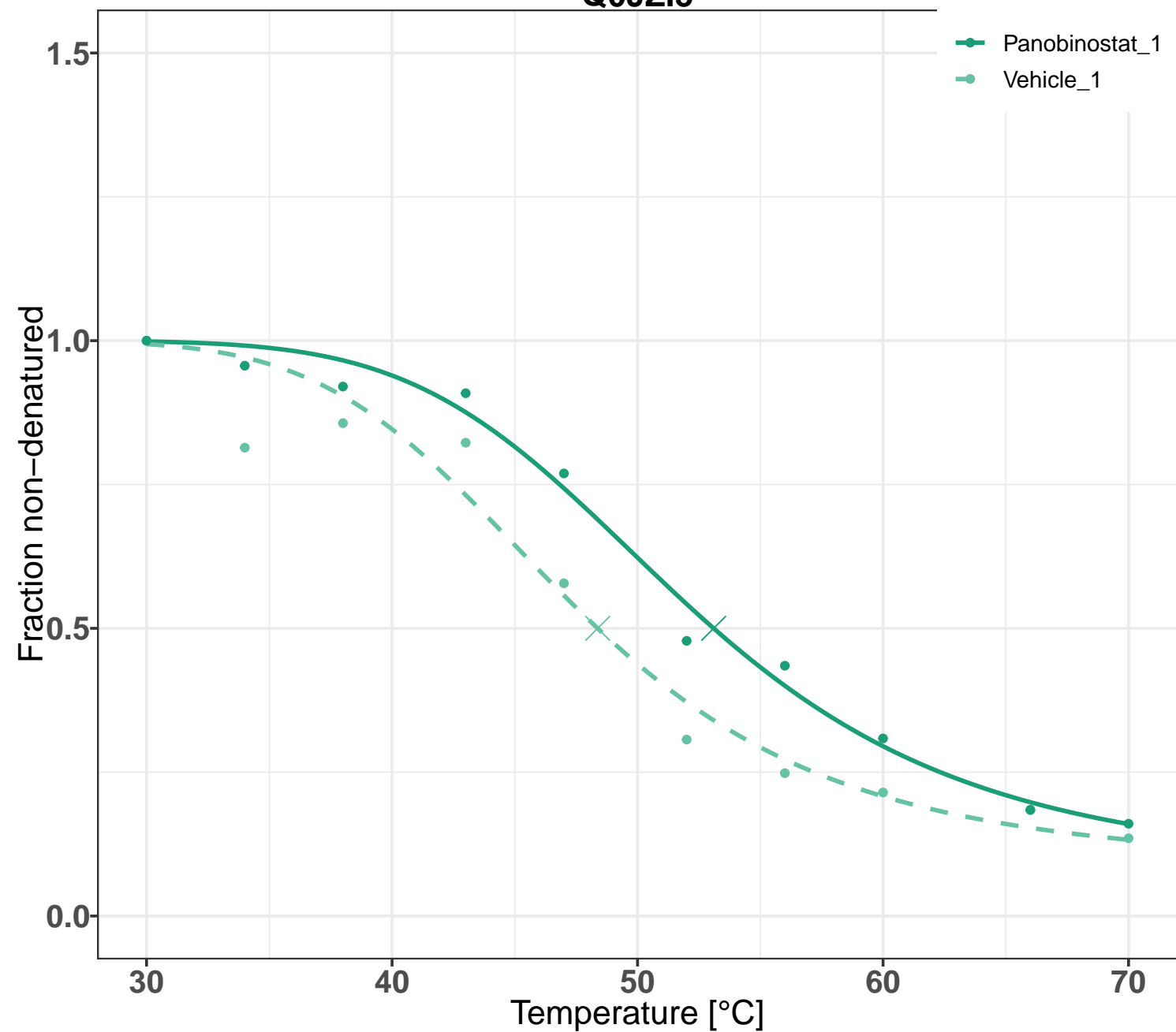

meltPoint

slope

plateau

R2

Panobinostat\_1

53.1

-0.041

0.07

0.99

Vehicle\_1

48.38

-0.044

0.08

0.96

Supplement: Supplementary file 2 — Supplementary Material 2 [file 41598_2026_35990_MOESM2_ESM.zip › AllTheTPPData/D40vD86/Panobinostat_Vignette/Melting_Curves/meltCurve_Q0JZI5.pdf]

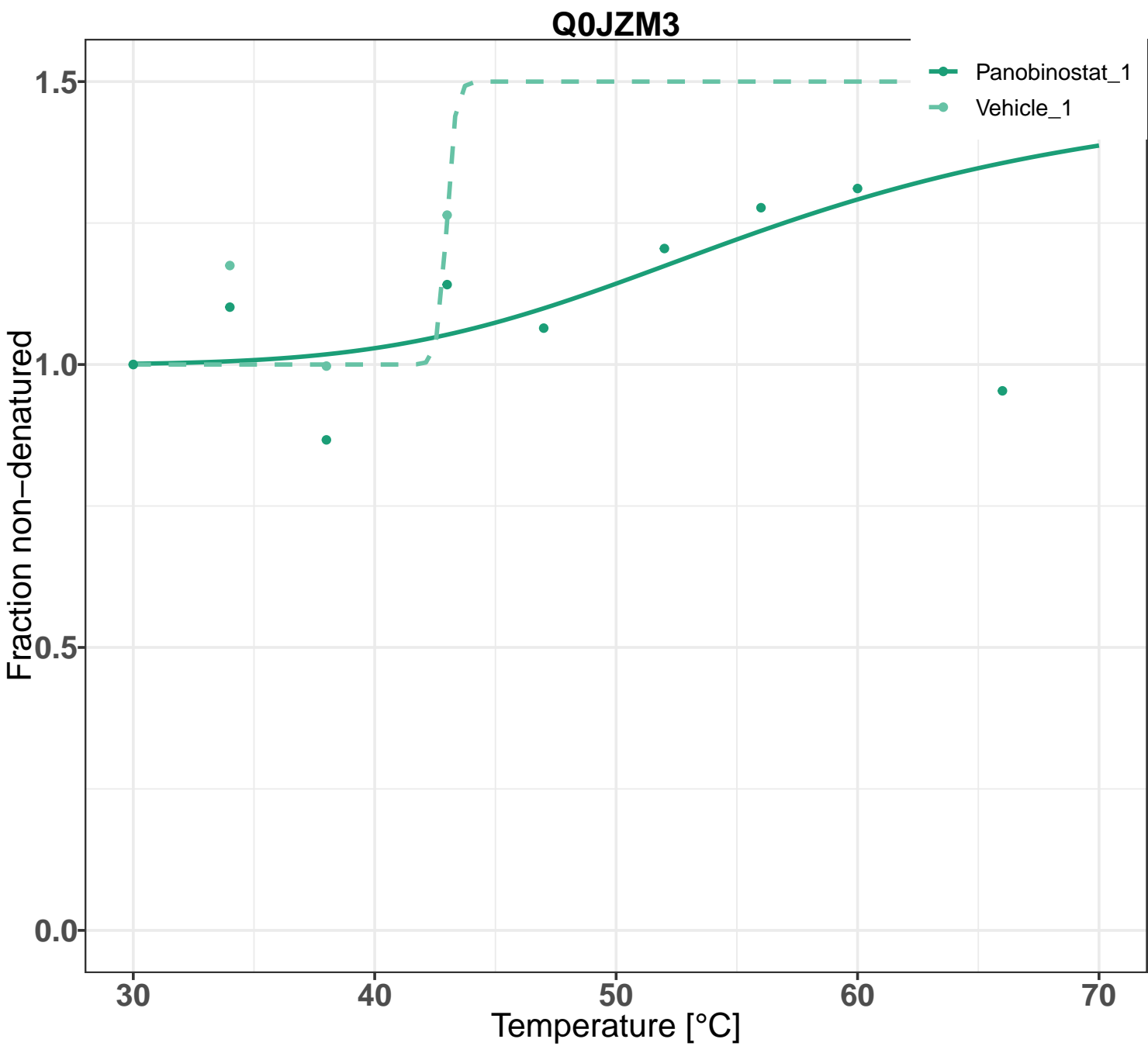

|                | meltPoint | slope | plateau | R2   |
|----------------|-----------|-------|---------|------|
| Panobinostat_1 | —         | 0.016 | 1.5     | 0.39 |
| Vehicle_1      | —         | 0.7   | 1.5     | 0.28 |

Supplement: Supplementary file 2 — Supplementary Material 2 [file 41598_2026_35990_MOESM2_ESM.zip › AllTheTPPData/D40vD86/Panobinostat_Vignette/Melting_Curves/meltCurve_Q0JZM3.pdf]

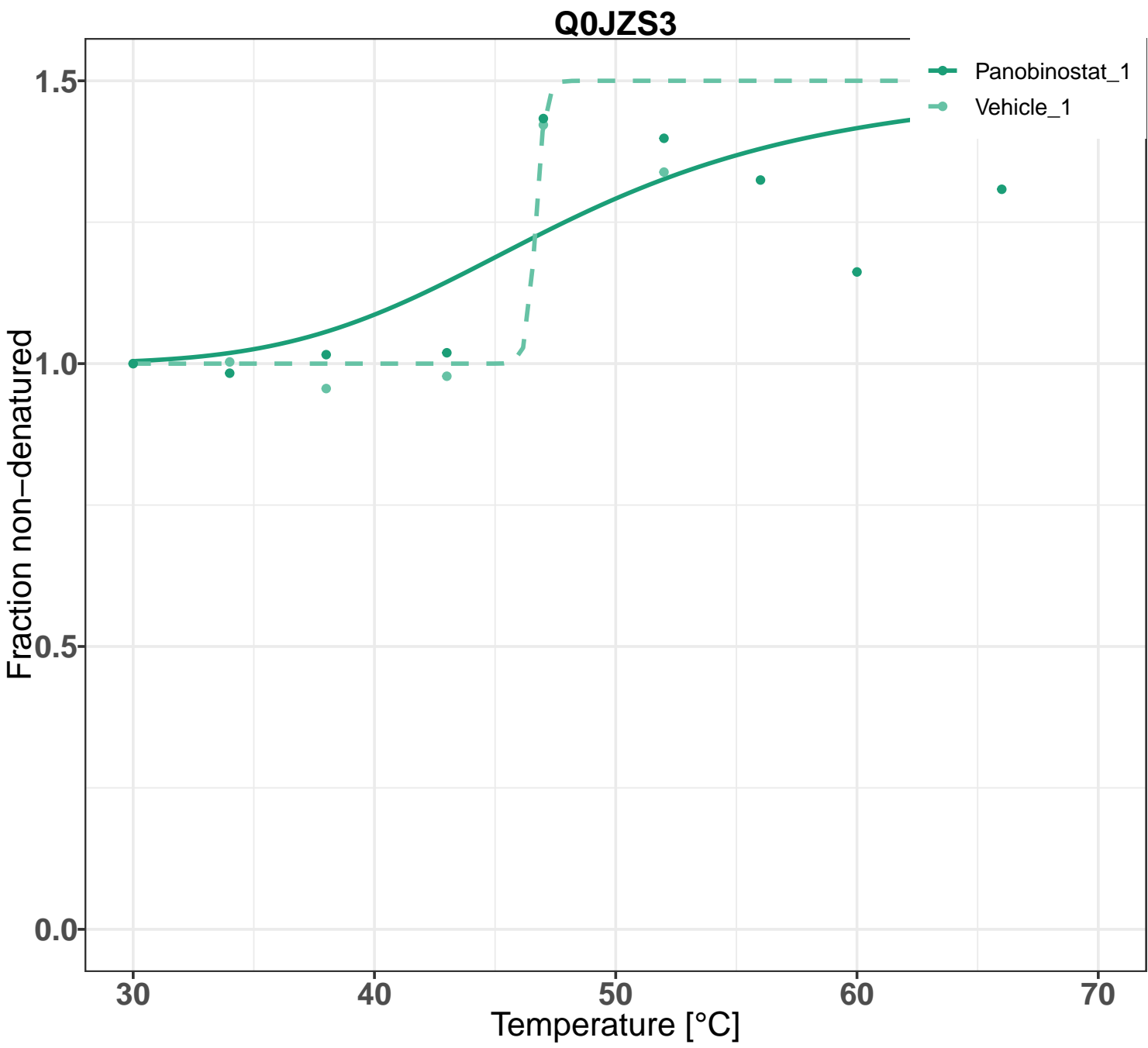

|                | meltPoint | slope | plateau | R2   |
|----------------|-----------|-------|---------|------|
| Panobinostat_1 | —         | 0.022 | 1.5     | 0.57 |
| Vehicle_1      | —         | 0.67  | 1.5     | 0.7  |

Supplement: Supplementary file 2 — Supplementary Material 2 [file 41598_2026_35990_MOESM2_ESM.zip › AllTheTPPData/D40vD86/Panobinostat_Vignette/Melting_Curves/meltCurve_Q0JZS3.pdf]

# Q0JZW1

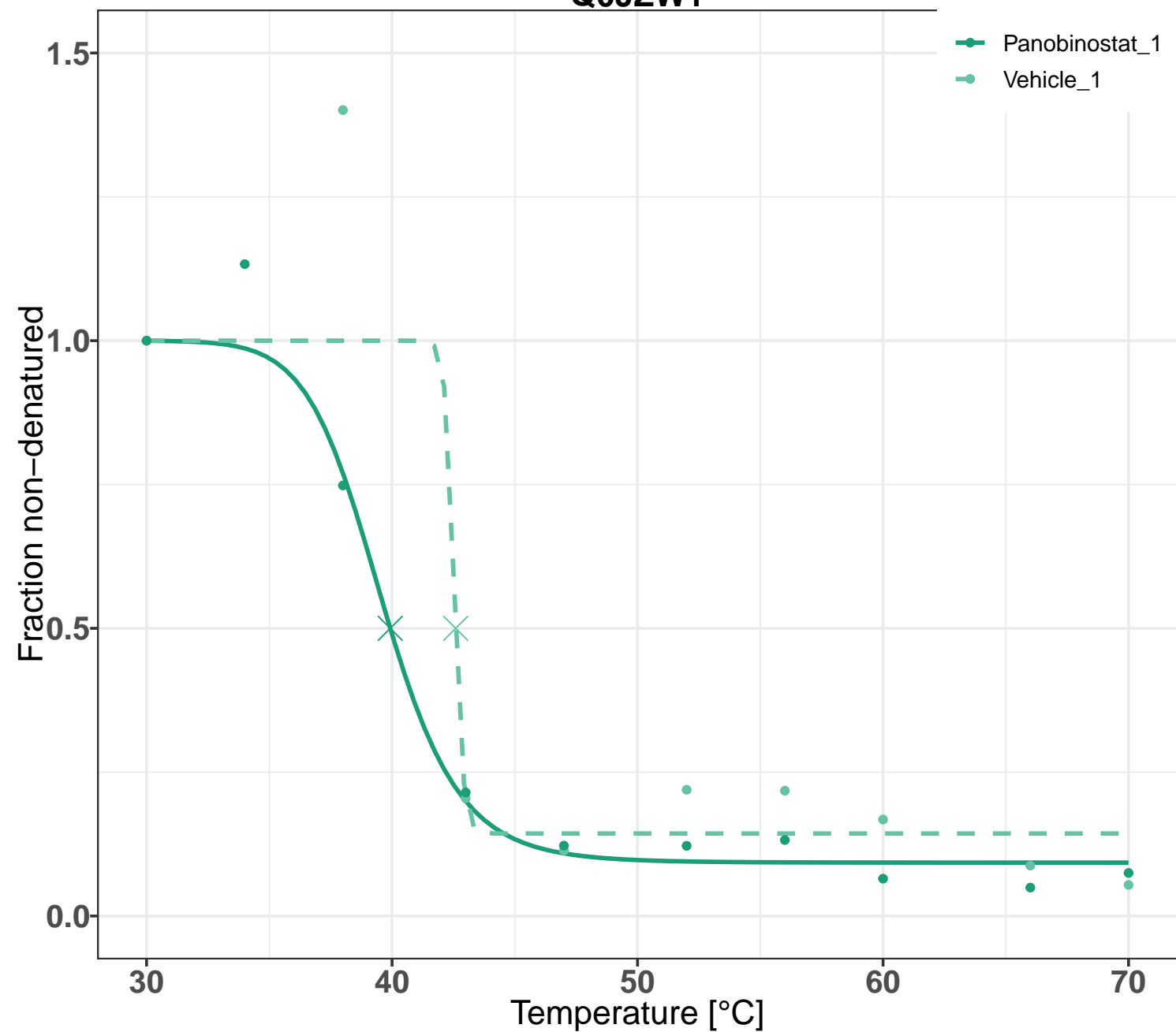

meltPoint

slope

plateau

R2

Panobinostat\_1

39.93

-0.15

0.09

0.98

Vehicle\_1

42.59

-1.2

0.14

0.79

Supplement: Supplementary file 2 — Supplementary Material 2 [file 41598_2026_35990_MOESM2_ESM.zip › AllTheTPPData/D40vD86/Panobinostat_Vignette/Melting_Curves/meltCurve_Q0JZW1.pdf]

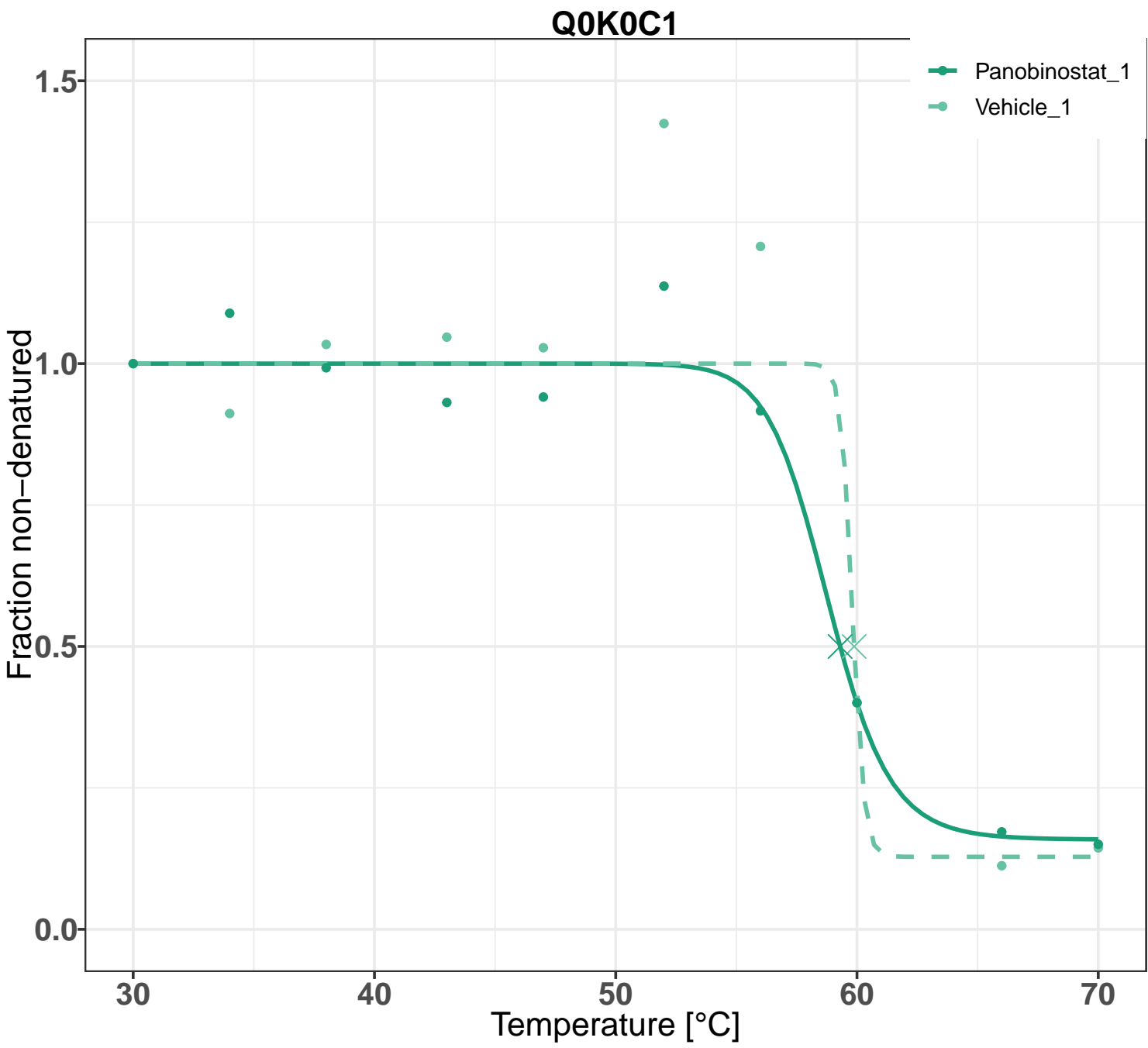

|                | meltPoint | slope | plateau | R2   |
|----------------|-----------|-------|---------|------|
| Panobinostat_1 | 59.3      | -0.17 | 0.16    | 0.97 |
| Vehicle_1      | 59.88     | -0.91 | 0.13    | 0.87 |

Supplement: Supplementary file 2 — Supplementary Material 2 [file 41598_2026_35990_MOESM2_ESM.zip › AllTheTPPData/D40vD86/Panobinostat_Vignette/Melting_Curves/meltCurve_Q0K0C1.pdf]

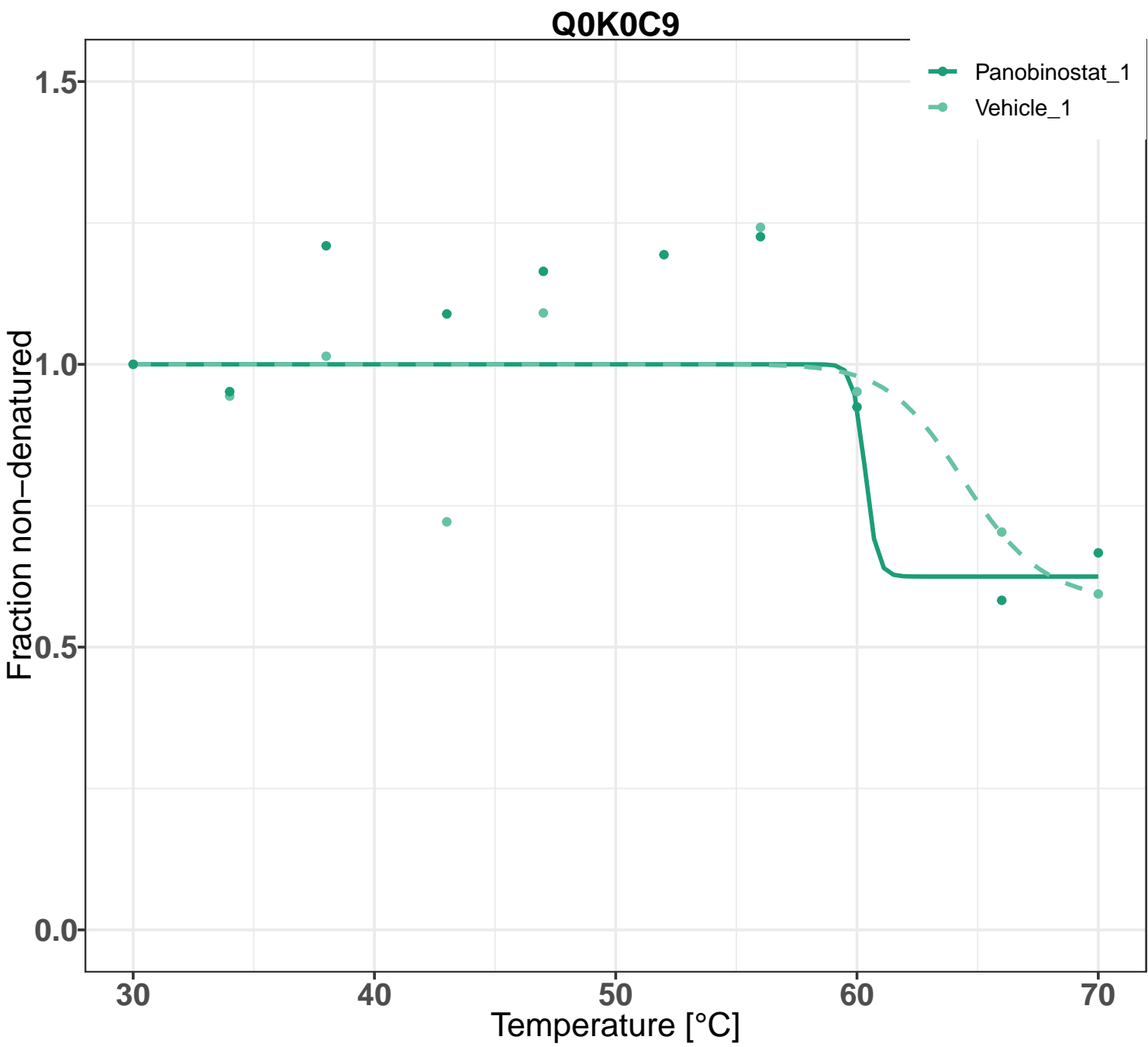

|                | meltPoint | slope  | plateau | R2   |
|----------------|-----------|--------|---------|------|
| Panobinostat_1 | —         | -0.39  | 0.62    | 0.62 |
| Vehicle_1      | —         | -0.065 | 0.58    | 0.19 |

Supplement: Supplementary file 2 — Supplementary Material 2 [file 41598_2026_35990_MOESM2_ESM.zip › AllTheTPPData/D40vD86/Panobinostat_Vignette/Melting_Curves/meltCurve_Q0K0C9.pdf]

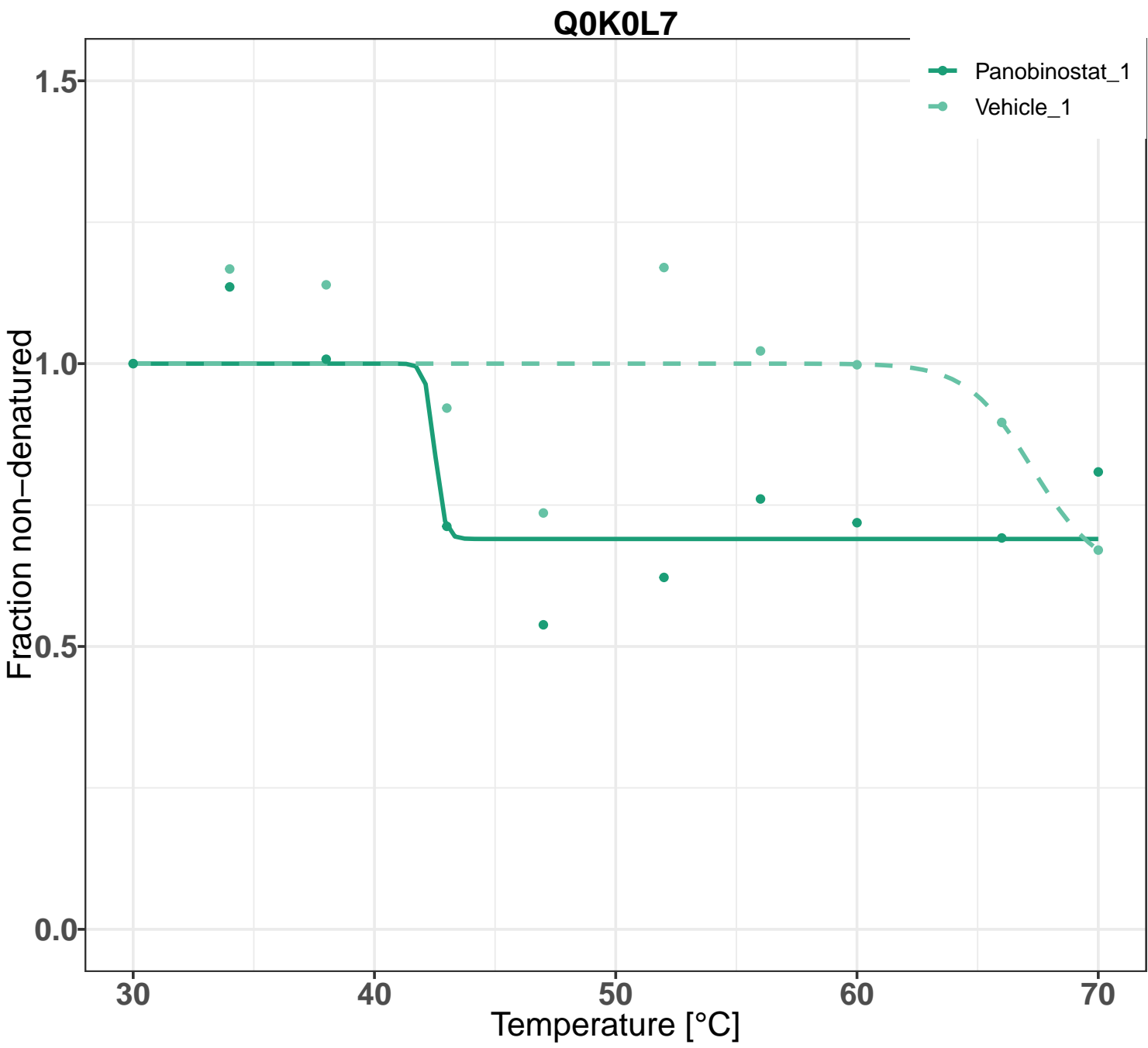

|                | meltPoint | slope  | plateau | R2   |
|----------------|-----------|--------|---------|------|
| Panobinostat_1 | —         | -0.4   | 0.69    | 0.8  |
| Vehicle_1      | —         | -0.068 | 0.62    | 0.42 |

Supplement: Supplementary file 2 — Supplementary Material 2 [file 41598_2026_35990_MOESM2_ESM.zip › AllTheTPPData/D40vD86/Panobinostat_Vignette/Melting_Curves/meltCurve_Q0K0L7.pdf]

# Q0K0N2

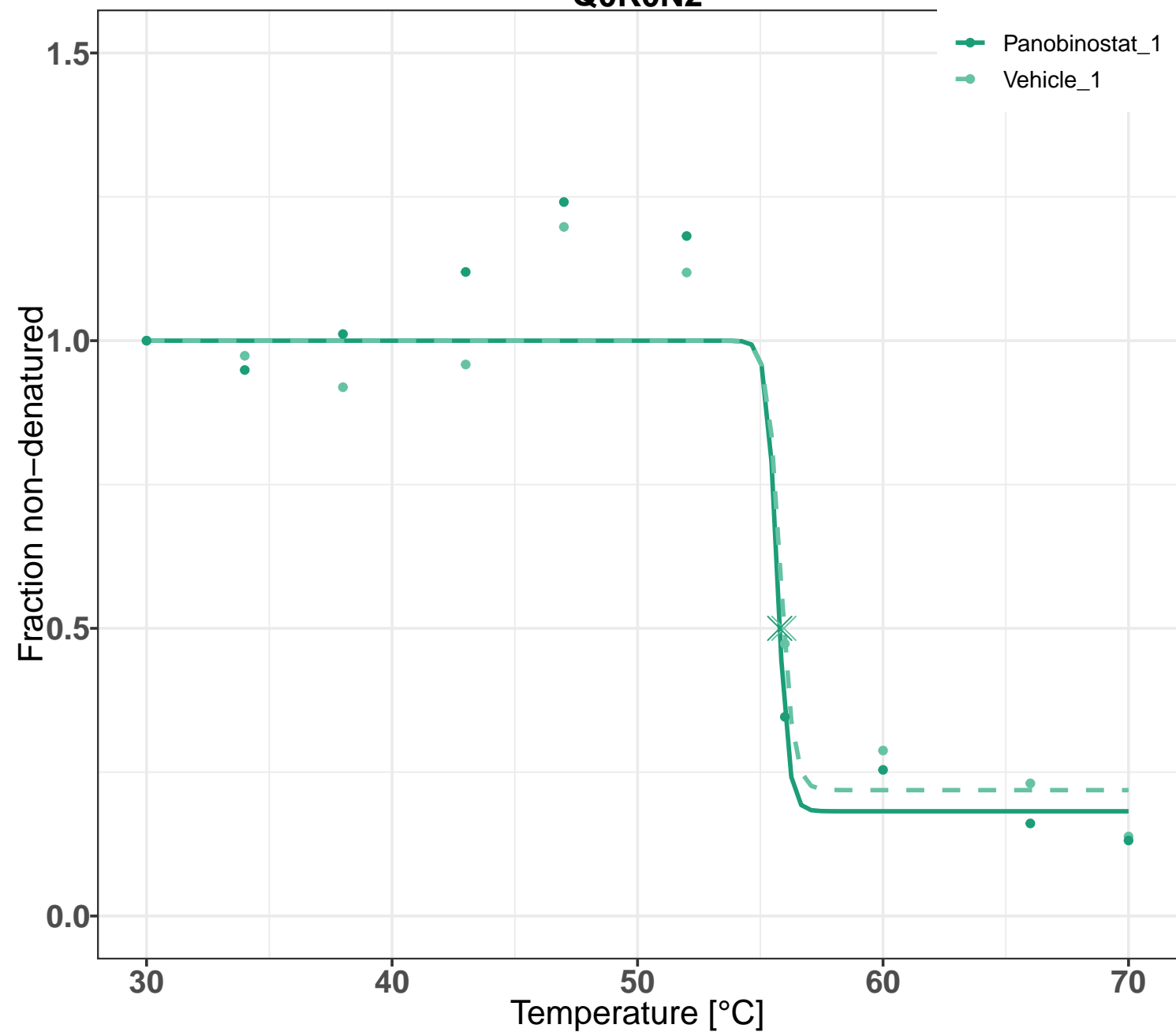

|                | meltPoint | slope | plateau | R2   |
|----------------|-----------|-------|---------|------|
| Panobinostat_1 | 55.79     | -0.92 | 0.18    | 0.94 |
| Vehicle_1      | 55.96     | -0.73 | 0.22    | 0.95 |

Supplement: Supplementary file 2 — Supplementary Material 2 [file 41598_2026_35990_MOESM2_ESM.zip › AllTheTPPData/D40vD86/Panobinostat_Vignette/Melting_Curves/meltCurve_Q0K0N2.pdf]

# Q0K0P2

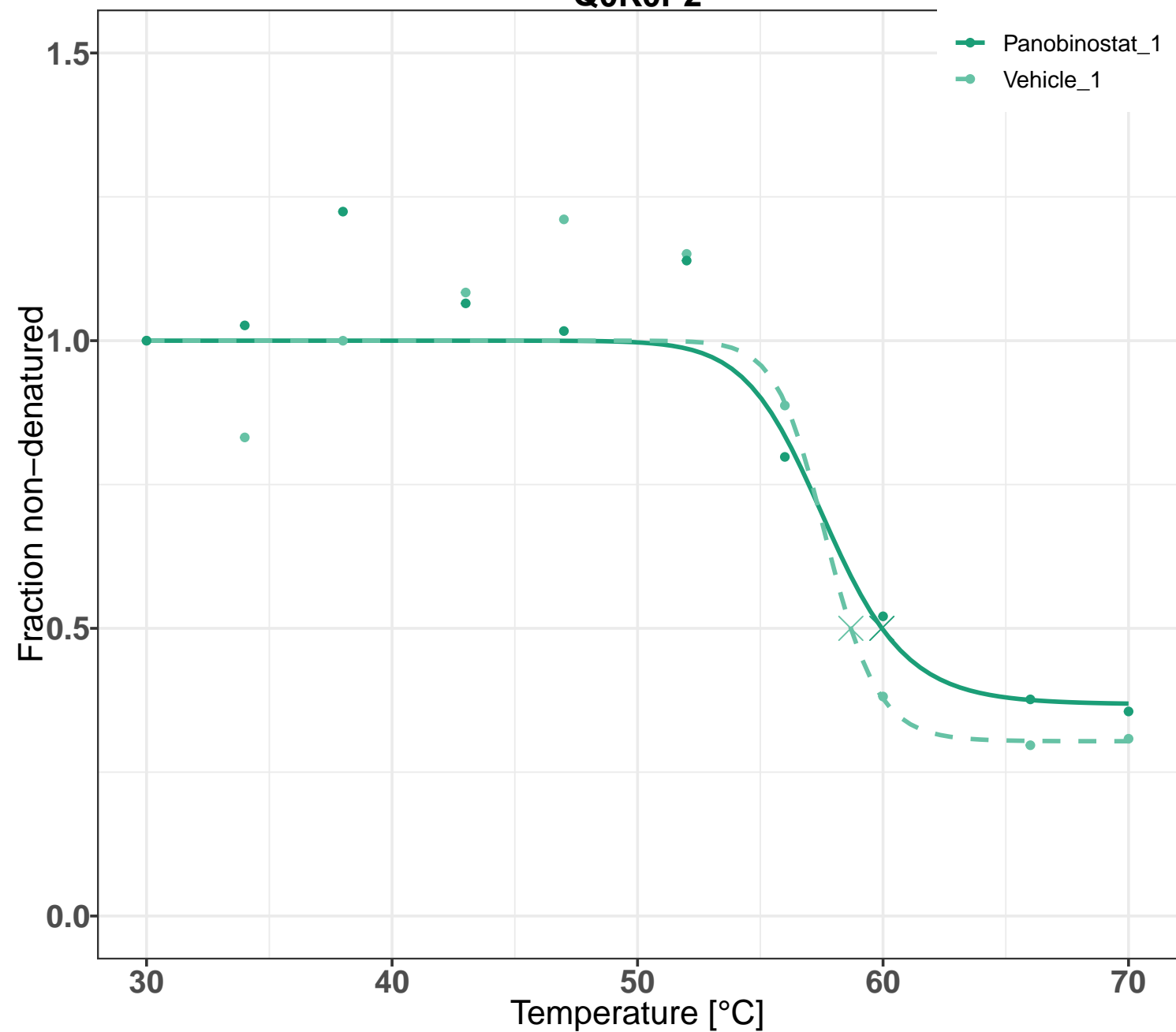

|                | meltPoint | slope  | plateau | R2   |
|----------------|-----------|--------|---------|------|
| Panobinostat_1 | 59.95     | -0.096 | 0.37    | 0.91 |
| Vehicle_1      | 58.69     | -0.17  | 0.3     | 0.91 |

Supplement: Supplementary file 2 — Supplementary Material 2 [file 41598_2026_35990_MOESM2_ESM.zip › AllTheTPPData/D40vD86/Panobinostat_Vignette/Melting_Curves/meltCurve_Q0K0P2.pdf]

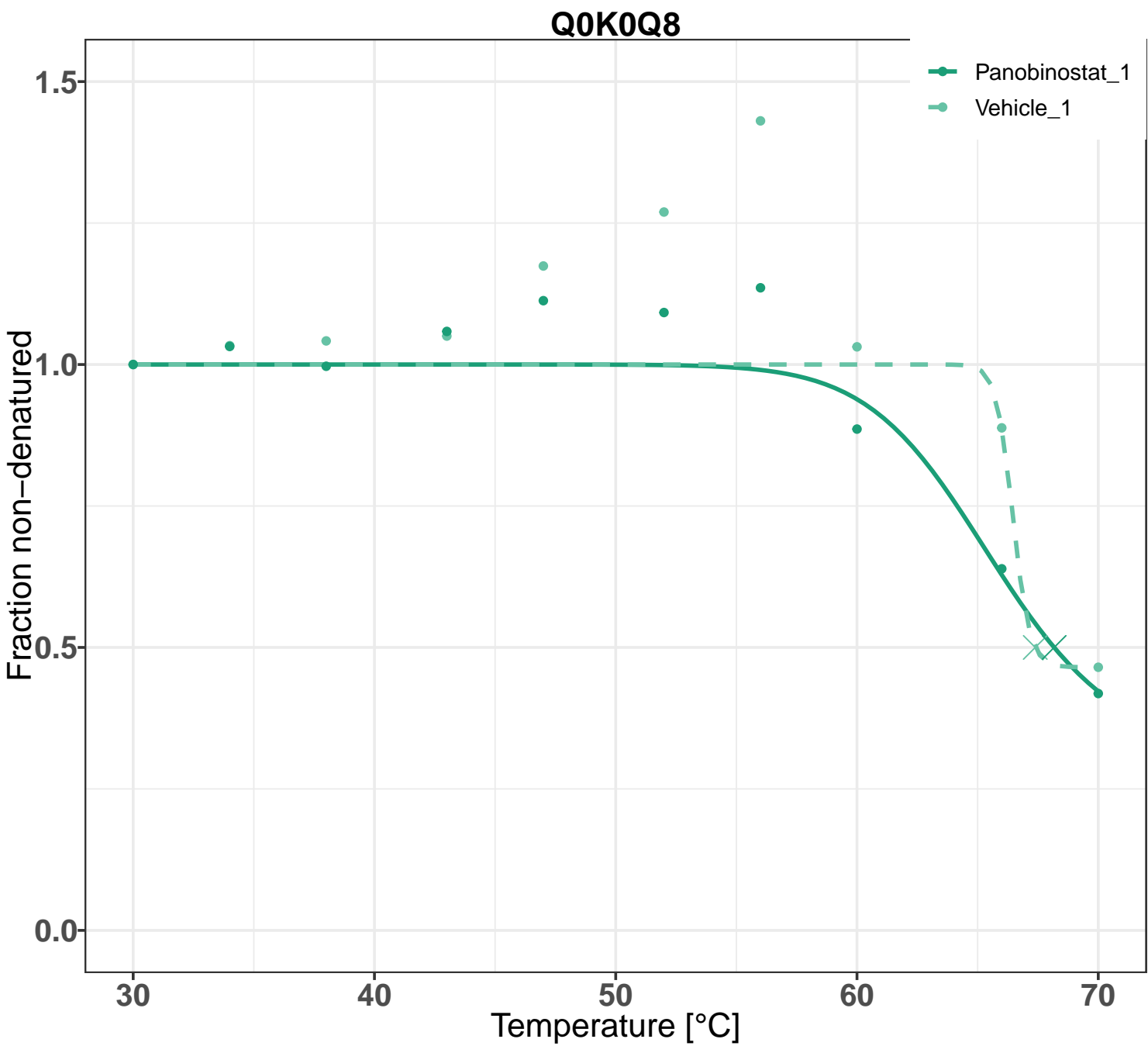

|                | meltPoint | slope  | plateau | R2   |
|----------------|-----------|--------|---------|------|
| Panobinostat_1 | 68.17     | -0.067 | 0.3     | 0.9  |
| Vehicle_1      | 67.39     | -0.39  | 0.46    | 0.49 |

Supplement: Supplementary file 2 — Supplementary Material 2 [file 41598_2026_35990_MOESM2_ESM.zip › AllTheTPPData/D40vD86/Panobinostat_Vignette/Melting_Curves/meltCurve_Q0K0Q8.pdf]

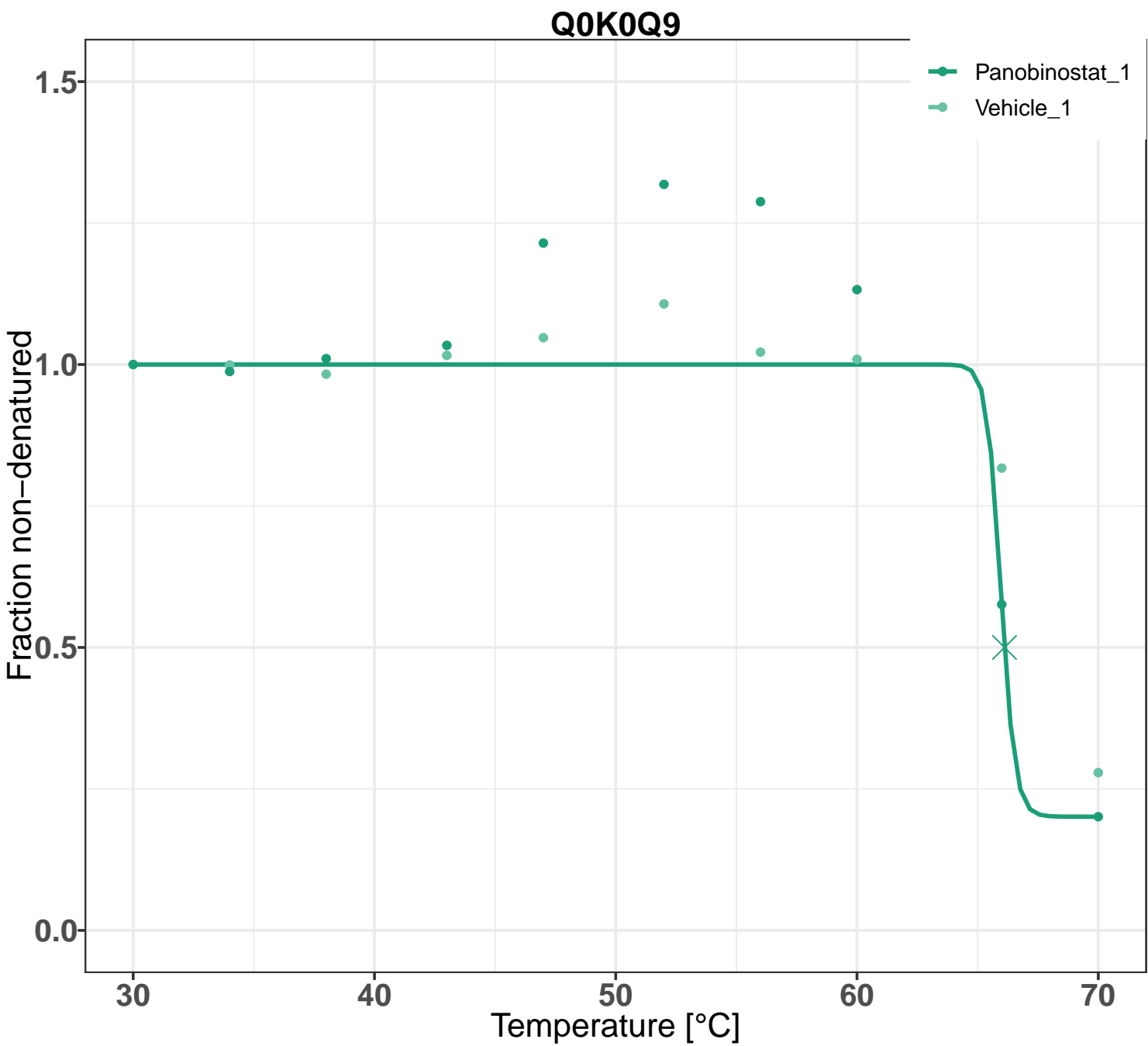

|                | meltPoint | slope | plateau | R2   |
|----------------|-----------|-------|---------|------|
| Panobinostat_1 | 66.11     | -0.69 | 0.2     | 0.77 |
| Vehicle_1      | -         | -     | -       | -    |

Supplement: Supplementary file 2 — Supplementary Material 2 [file 41598_2026_35990_MOESM2_ESM.zip › AllTheTPPData/D40vD86/Panobinostat_Vignette/Melting_Curves/meltCurve_Q0K0Q9.pdf]

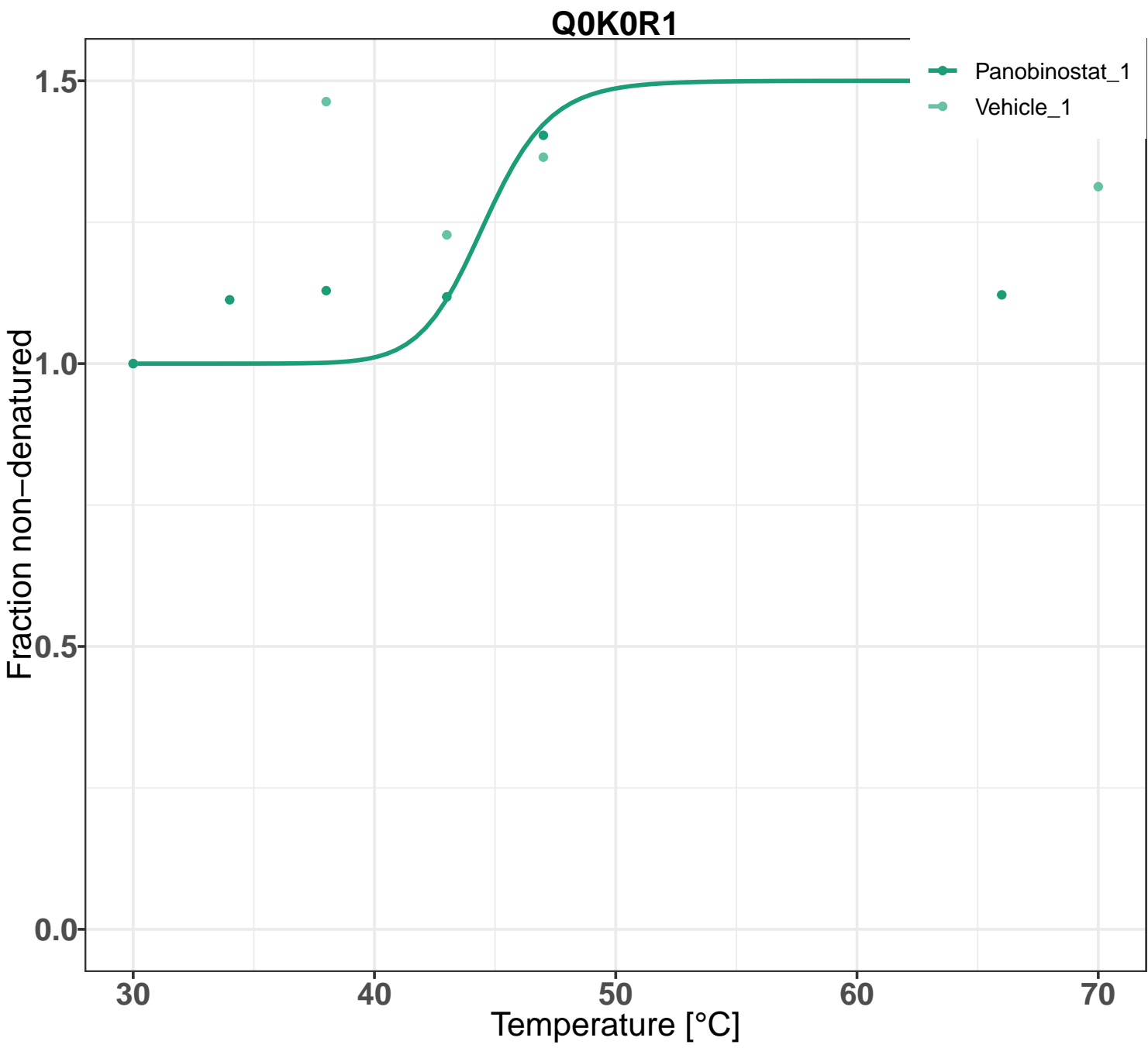

|                | meltPoint | slope | plateau | R2   |
|----------------|-----------|-------|---------|------|
| Panobinostat_1 | —         | 0.093 | 1.5     | 0.51 |
| Vehicle_1      | —         | —     | —       | —    |

Supplement: Supplementary file 2 — Supplementary Material 2 [file 41598_2026_35990_MOESM2_ESM.zip › AllTheTPPData/D40vD86/Panobinostat_Vignette/Melting_Curves/meltCurve_Q0K0R1.pdf]

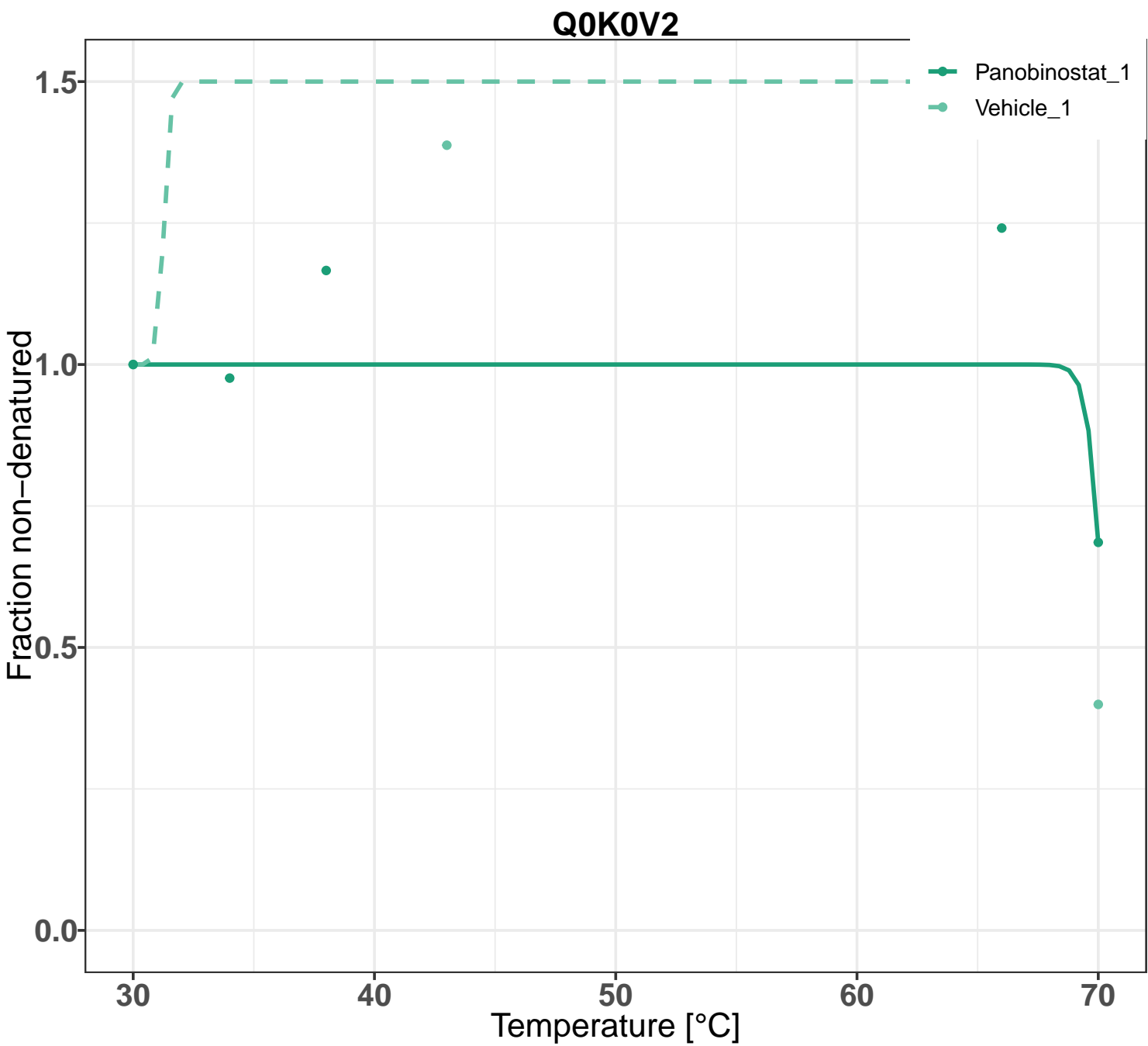

|                | meltPoint | slope | plateau | R2    |
|----------------|-----------|-------|---------|-------|
| Panobinostat_1 | –         | –     | 0       | –0.77 |
| Vehicle_1      | –         | 1     | 1.5     | –0.26 |

Supplement: Supplementary file 2 — Supplementary Material 2 [file 41598_2026_35990_MOESM2_ESM.zip › AllTheTPPData/D40vD86/Panobinostat_Vignette/Melting_Curves/meltCurve_Q0K0V2.pdf]

# Q0K0V6

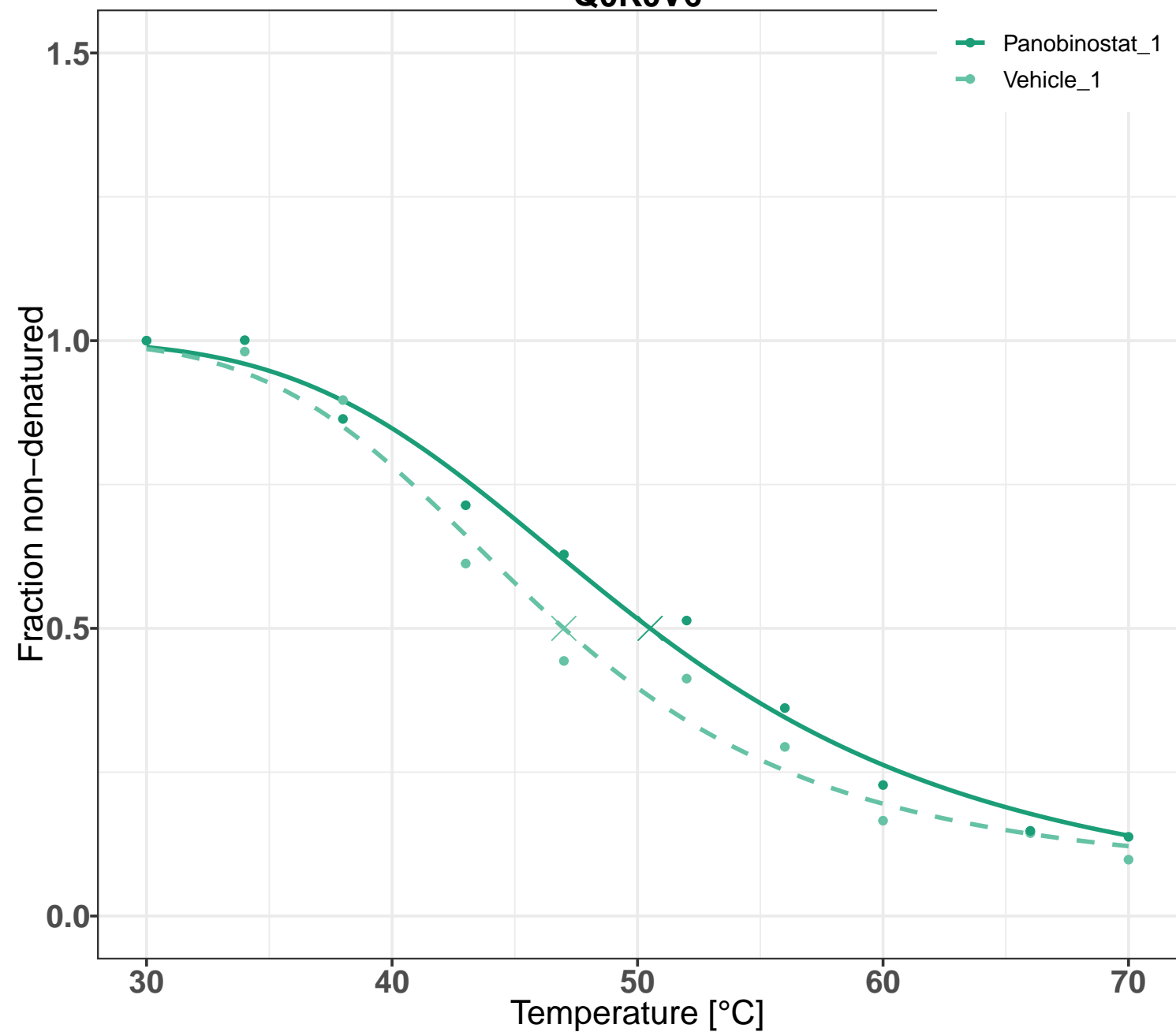

|                | meltPoint | slope  | plateau | R2   |
|----------------|-----------|--------|---------|------|
| Panobinostat_1 | 50.51     | -0.035 | 0       | 0.99 |
| Vehicle_1      | 47        | -0.042 | 0.06    | 0.98 |

Supplement: Supplementary file 2 — Supplementary Material 2 [file 41598_2026_35990_MOESM2_ESM.zip › AllTheTPPData/D40vD86/Panobinostat_Vignette/Melting_Curves/meltCurve_Q0K0V6.pdf]

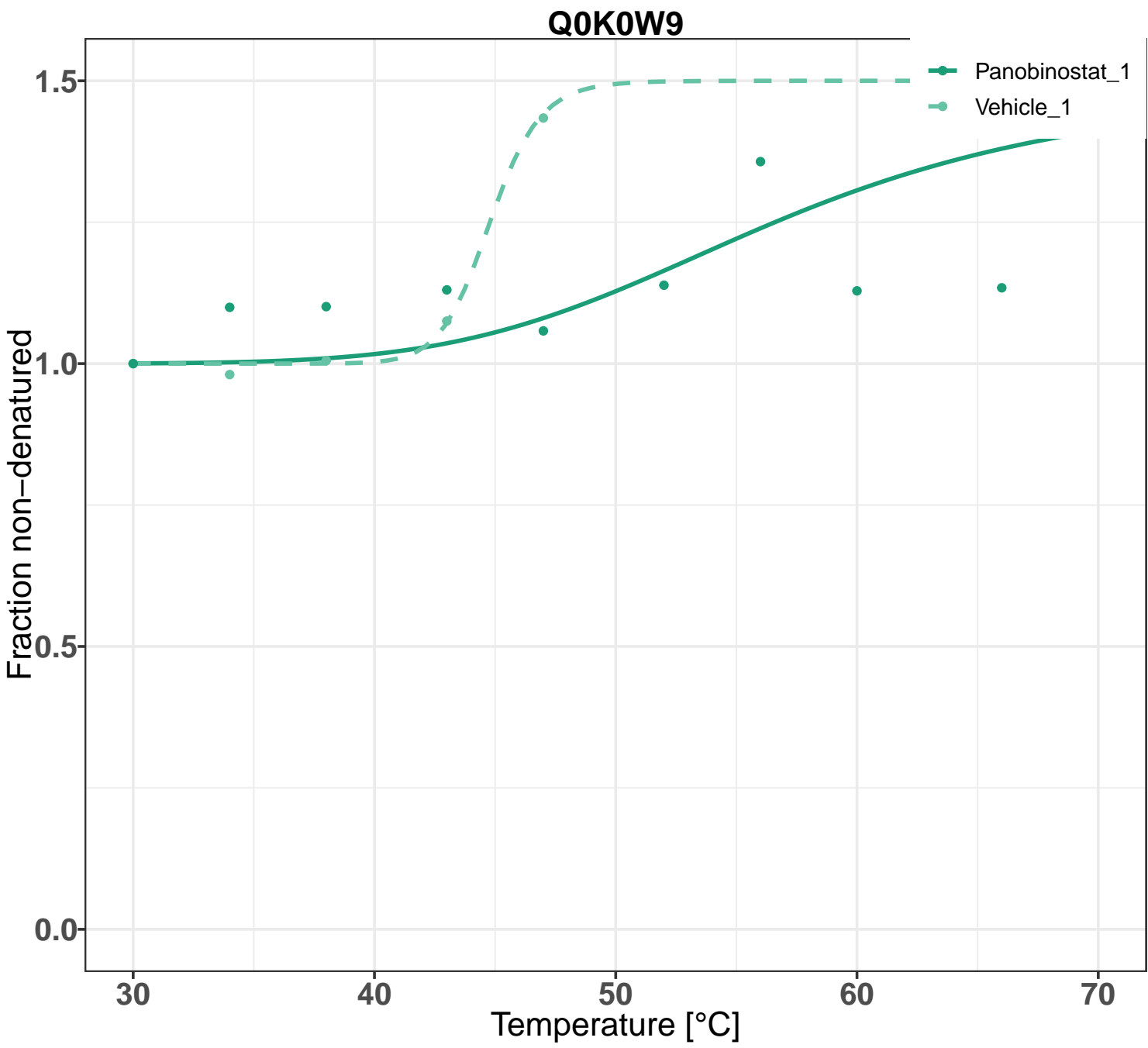

|                | meltPoint | slope | plateau | R2   |
|----------------|-----------|-------|---------|------|
| Panobinostat_1 | –         | 0.019 | 1.5     | 0.41 |
| Vehicle_1      | –         | 0.12  | 1.5     | 0.91 |

Supplement: Supplementary file 2 — Supplementary Material 2 [file 41598_2026_35990_MOESM2_ESM.zip › AllTheTPPData/D40vD86/Panobinostat_Vignette/Melting_Curves/meltCurve_Q0K0W9.pdf]

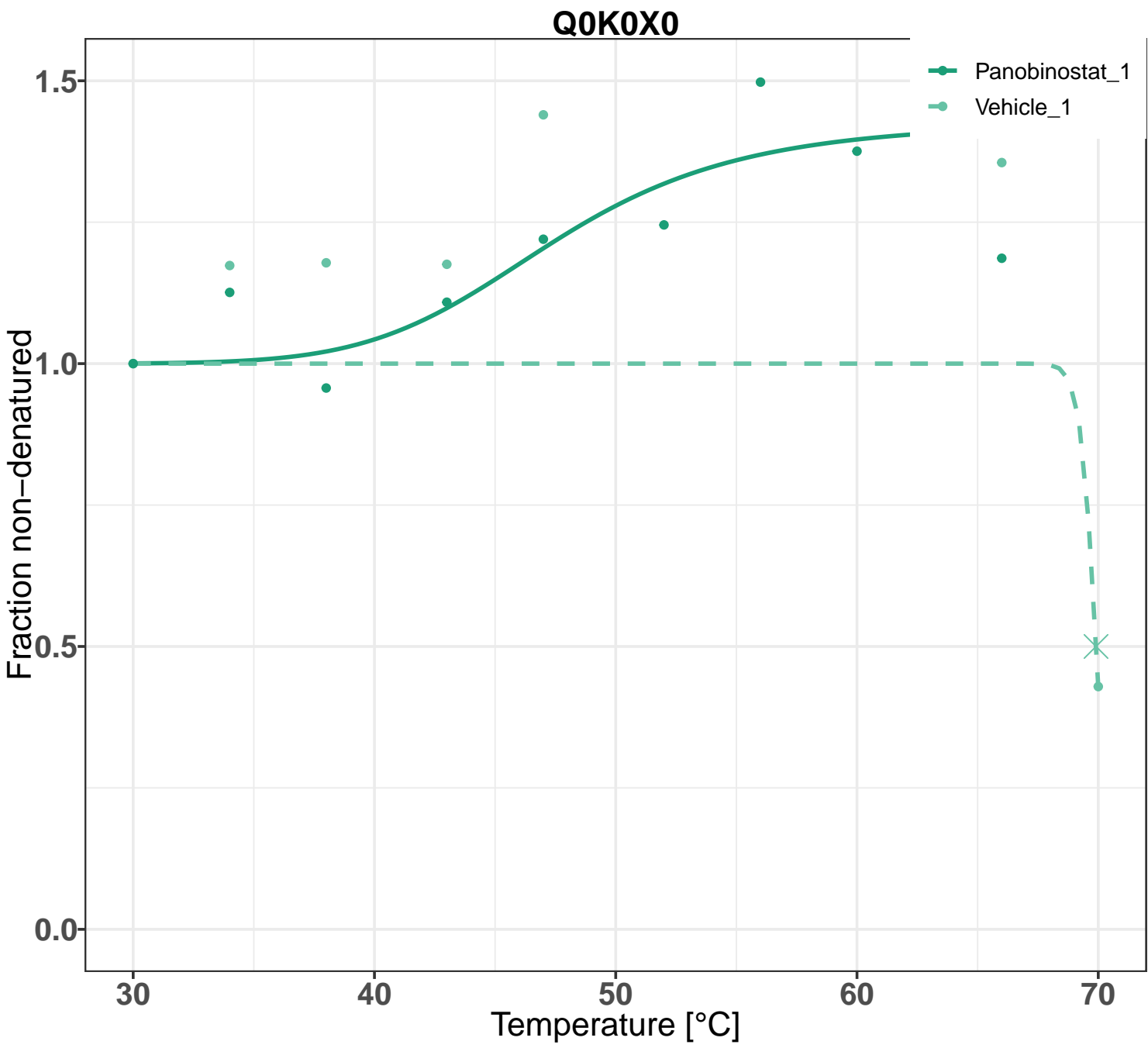

|                | meltPoint | slope | plateau | R2    |
|----------------|-----------|-------|---------|-------|
| Panobinostat_1 | –         | 0.028 | 1.43    | 0.67  |
| Vehicle_1      | 69.91     | -0.77 | 0       | -0.45 |

Supplement: Supplementary file 2 — Supplementary Material 2 [file 41598_2026_35990_MOESM2_ESM.zip › AllTheTPPData/D40vD86/Panobinostat_Vignette/Melting_Curves/meltCurve_Q0K0X0.pdf]

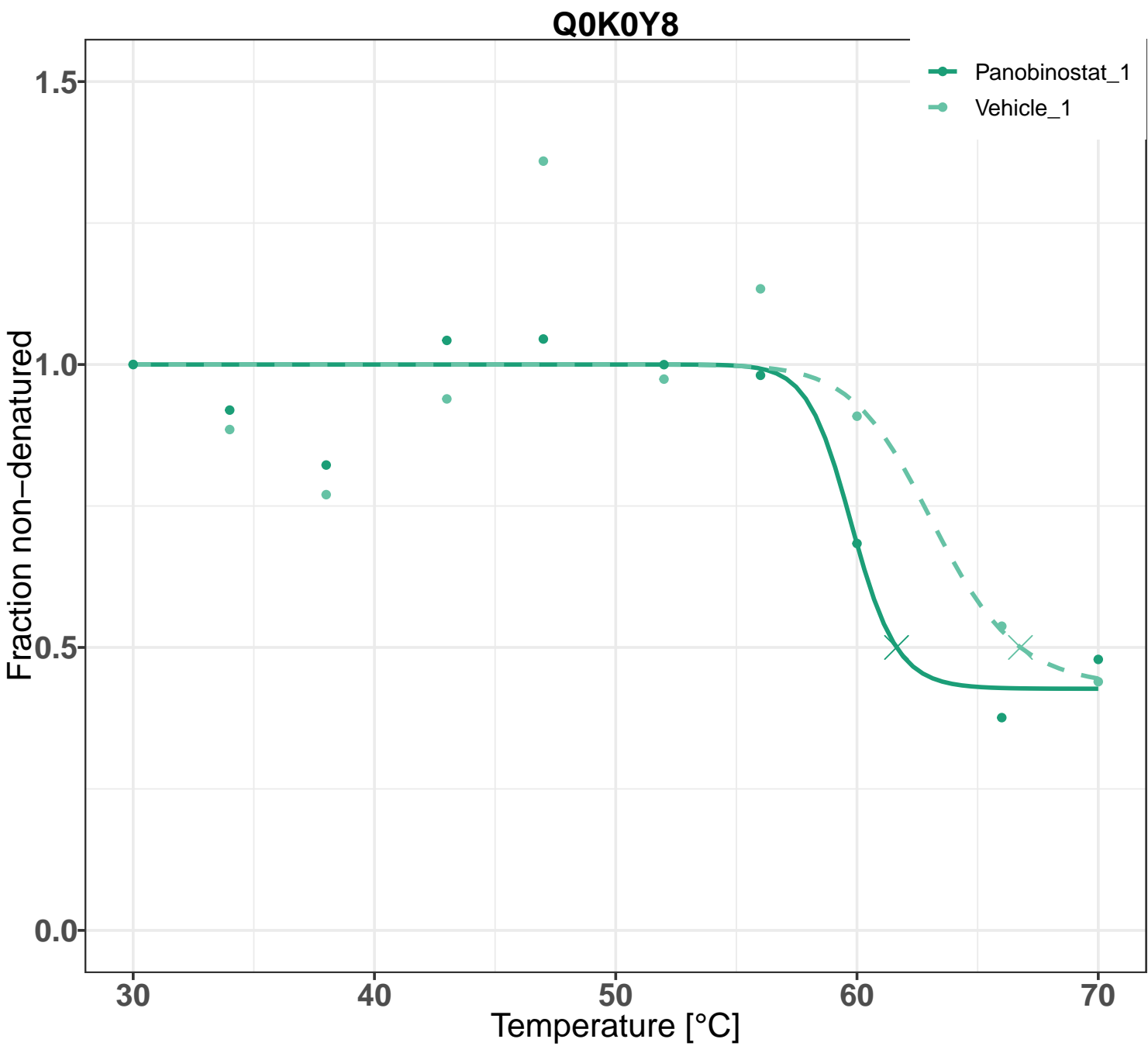

|                | meltPoint | slope  | plateau | R2   |
|----------------|-----------|--------|---------|------|
| Panobinostat_1 | 61.64     | -0.15  | 0.43    | 0.91 |
| Vehicle_1      | 66.77     | -0.083 | 0.43    | 0.66 |

Supplement: Supplementary file 2 — Supplementary Material 2 [file 41598_2026_35990_MOESM2_ESM.zip › AllTheTPPData/D40vD86/Panobinostat_Vignette/Melting_Curves/meltCurve_Q0K0Y8.pdf]

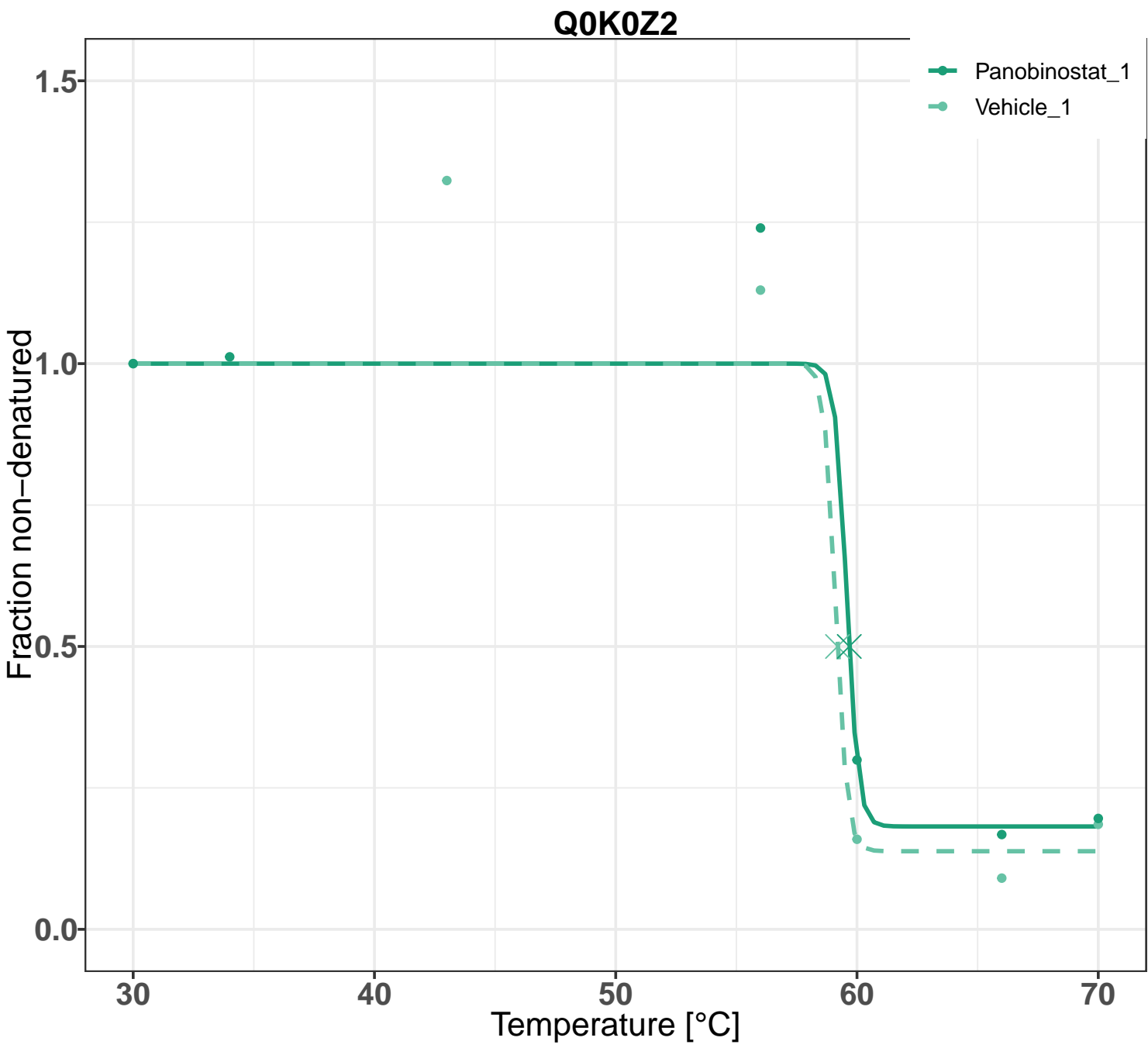

|                | meltPoint | slope | plateau | R2   |
|----------------|-----------|-------|---------|------|
| Panobinostat_1 | 59.68     | -0.86 | 0.18    | 0.44 |
| Vehicle_1      | 59.19     | -0.91 | 0.14    | 0.28 |

Supplement: Supplementary file 2 — Supplementary Material 2 [file 41598_2026_35990_MOESM2_ESM.zip › AllTheTPPData/D40vD86/Panobinostat_Vignette/Melting_Curves/meltCurve_Q0K0Z2.pdf]

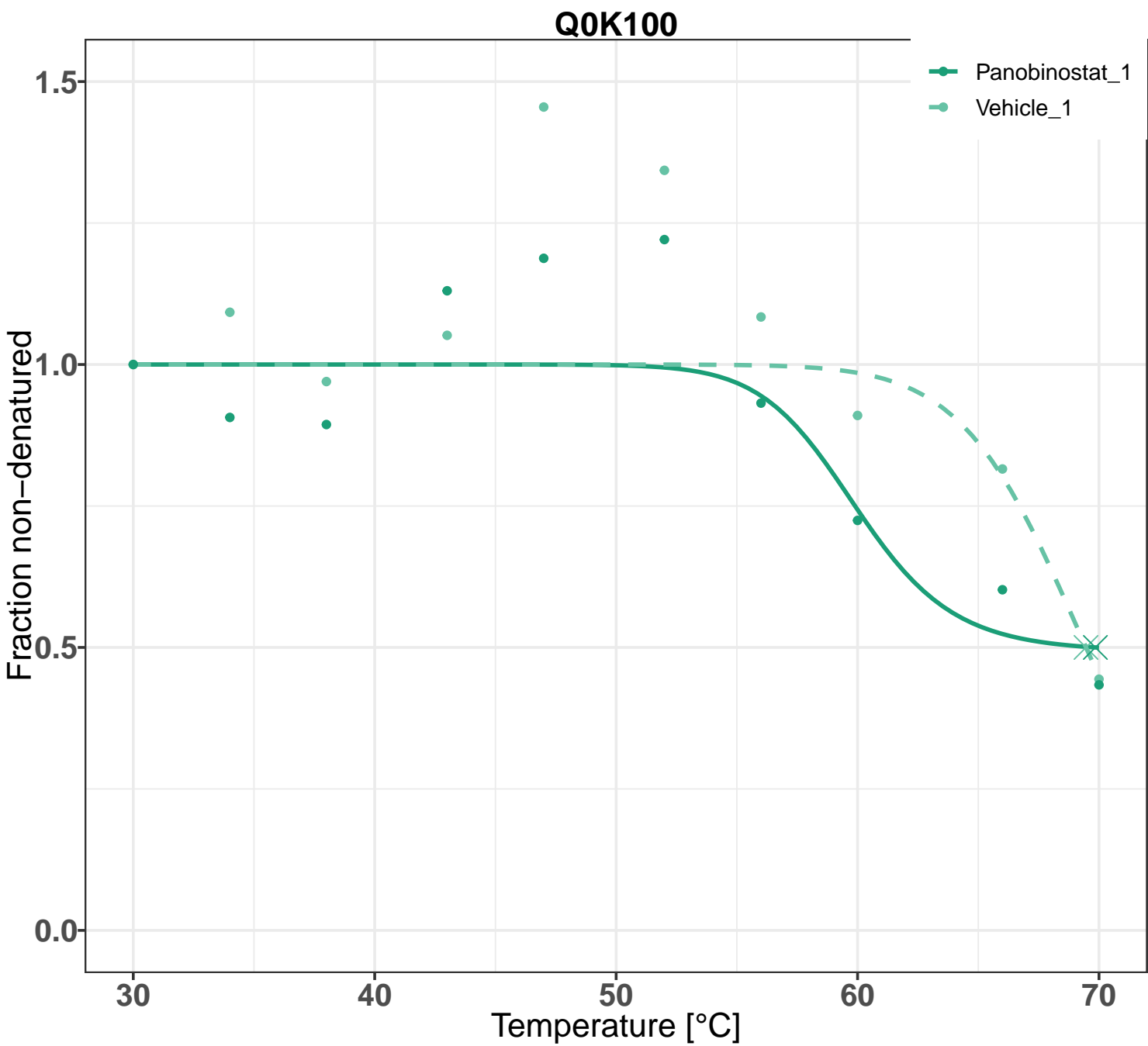

|                | meltPoint | slope  | plateau | R2   |
|----------------|-----------|--------|---------|------|
| Panobinostat_1 | 69.85     | -0.063 | 0.49    | 0.77 |
| Vehicle_1      | 69.46     | -0.096 | 0       | 0.49 |

Supplement: Supplementary file 2 — Supplementary Material 2 [file 41598_2026_35990_MOESM2_ESM.zip › AllTheTPPData/D40vD86/Panobinostat_Vignette/Melting_Curves/meltCurve_Q0K100.pdf]

# Q0K187

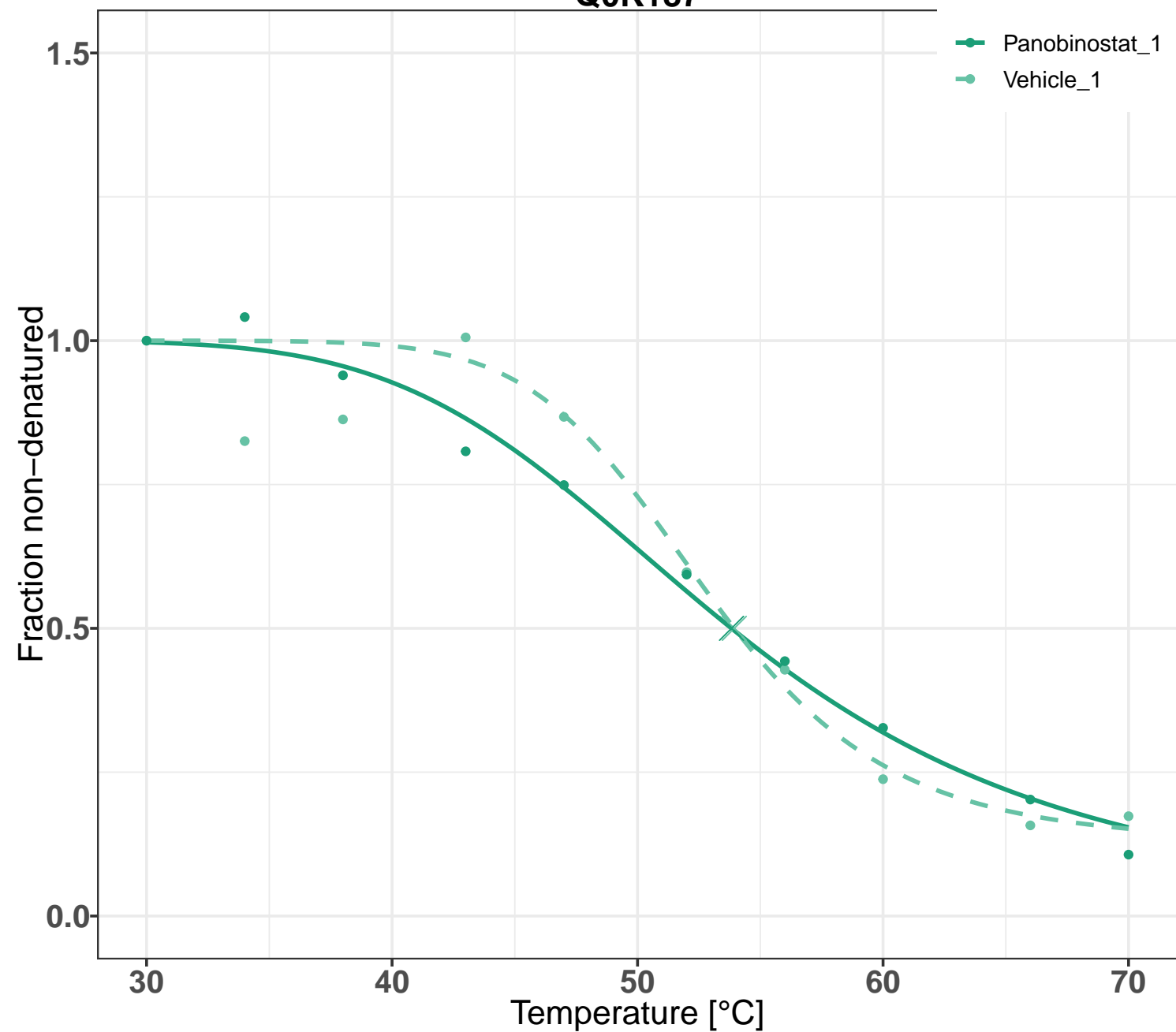

meltPoint

slope

plateau

R2

Panobinostat\_1

53.83

-0.037

0

0.99

Vehicle\_1

53.94

-0.06

0.13

0.95

Supplement: Supplementary file 2 — Supplementary Material 2 [file 41598_2026_35990_MOESM2_ESM.zip › AllTheTPPData/D40vD86/Panobinostat_Vignette/Melting_Curves/meltCurve_Q0K187.pdf]

# Q0K1C9

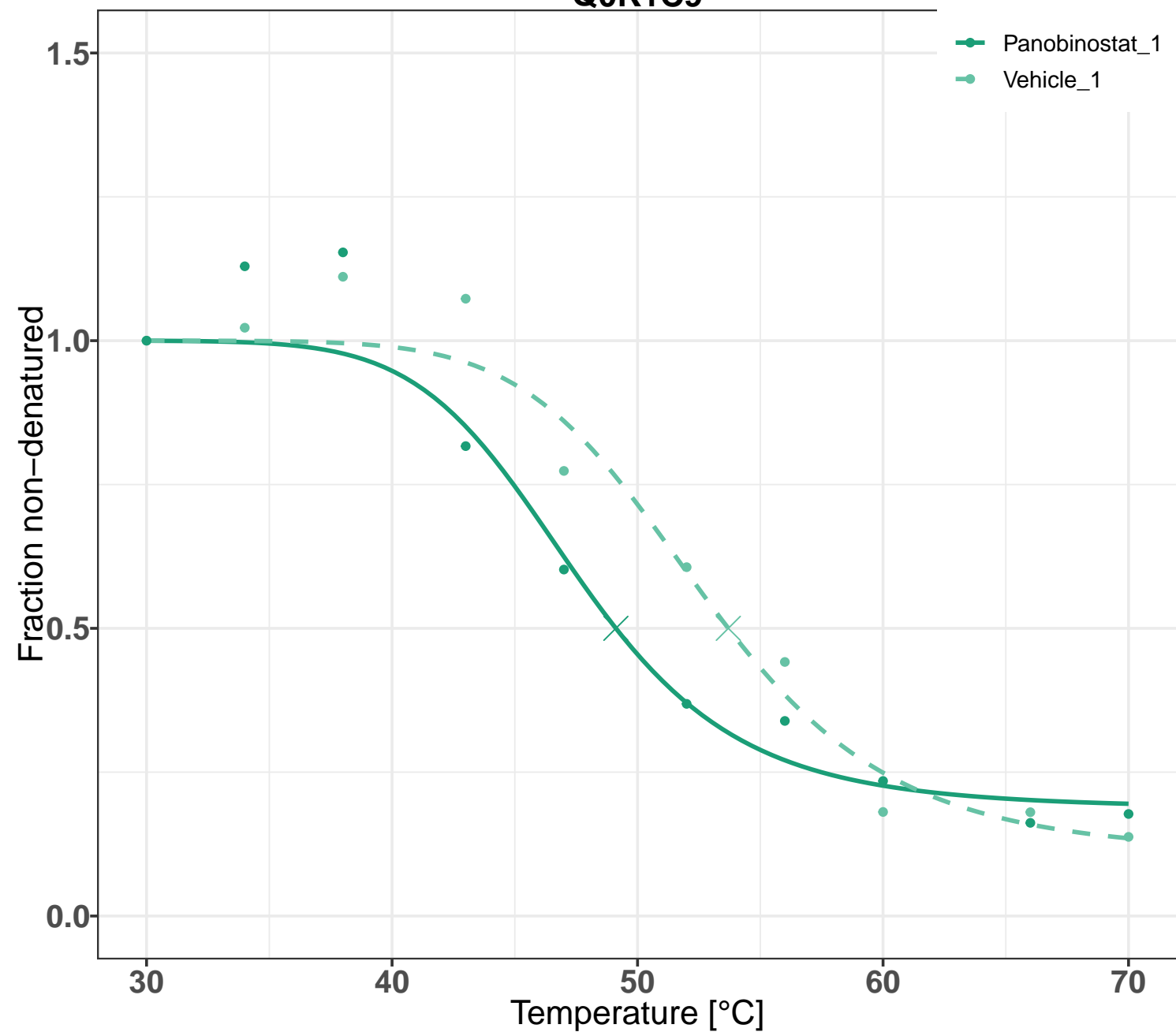

|                | meltPoint | slope  | plateau | R2   |
|----------------|-----------|--------|---------|------|
| Panobinostat_1 | 49.12     | -0.062 | 0.19    | 0.96 |
| Vehicle_1      | 53.7      | -0.059 | 0.11    | 0.97 |

Supplement: Supplementary file 2 — Supplementary Material 2 [file 41598_2026_35990_MOESM2_ESM.zip › AllTheTPPData/D40vD86/Panobinostat_Vignette/Melting_Curves/meltCurve_Q0K1C9.pdf]

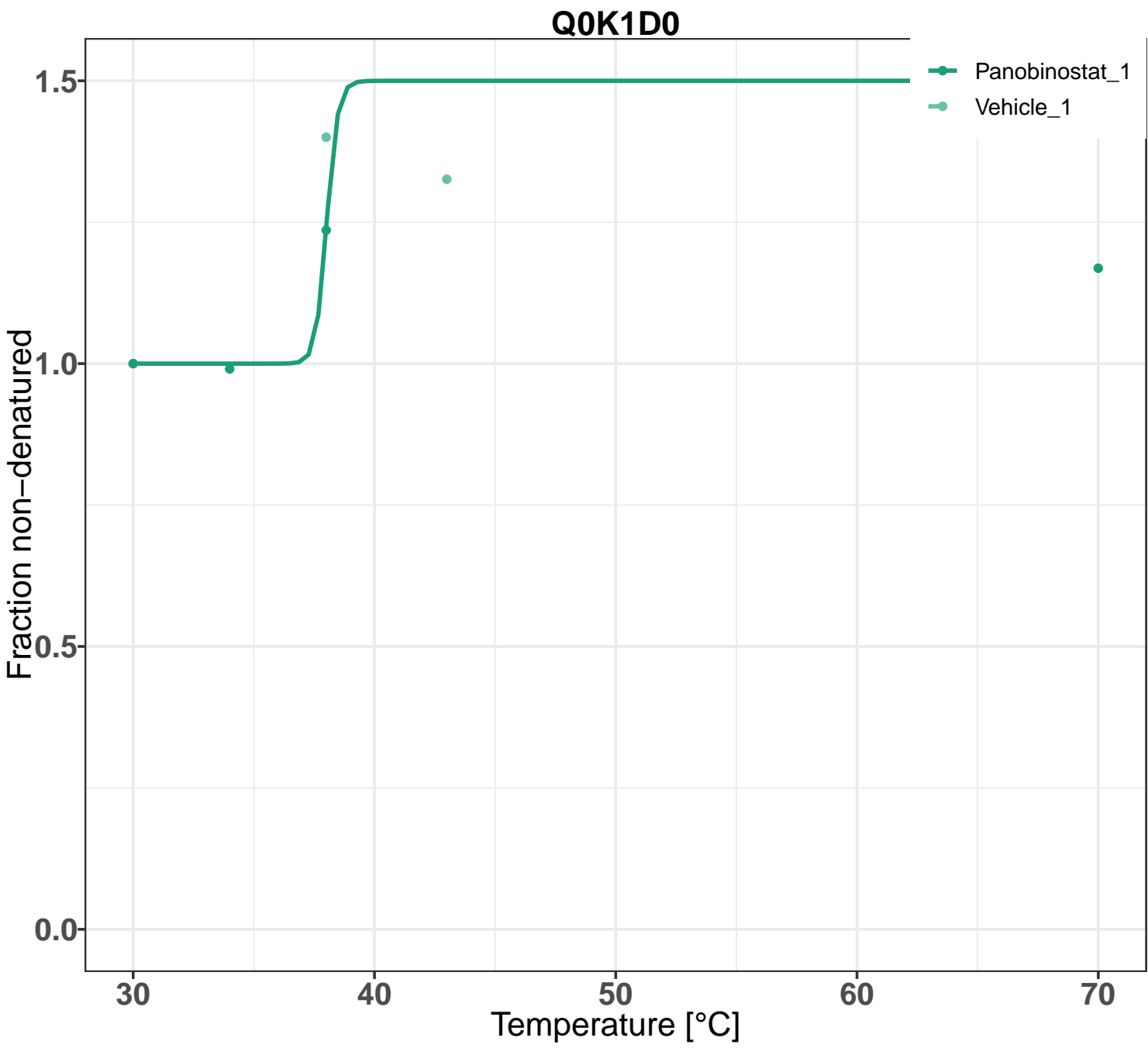

|                | meltPoint | slope | plateau | R2   |
|----------------|-----------|-------|---------|------|
| Panobinostat_1 | —         | 0.56  | 1.5     | 0.07 |
| Vehicle_1      | —         | —     | —       | —    |

Supplement: Supplementary file 2 — Supplementary Material 2 [file 41598_2026_35990_MOESM2_ESM.zip › AllTheTPPData/D40vD86/Panobinostat_Vignette/Melting_Curves/meltCurve_Q0K1D0.pdf]

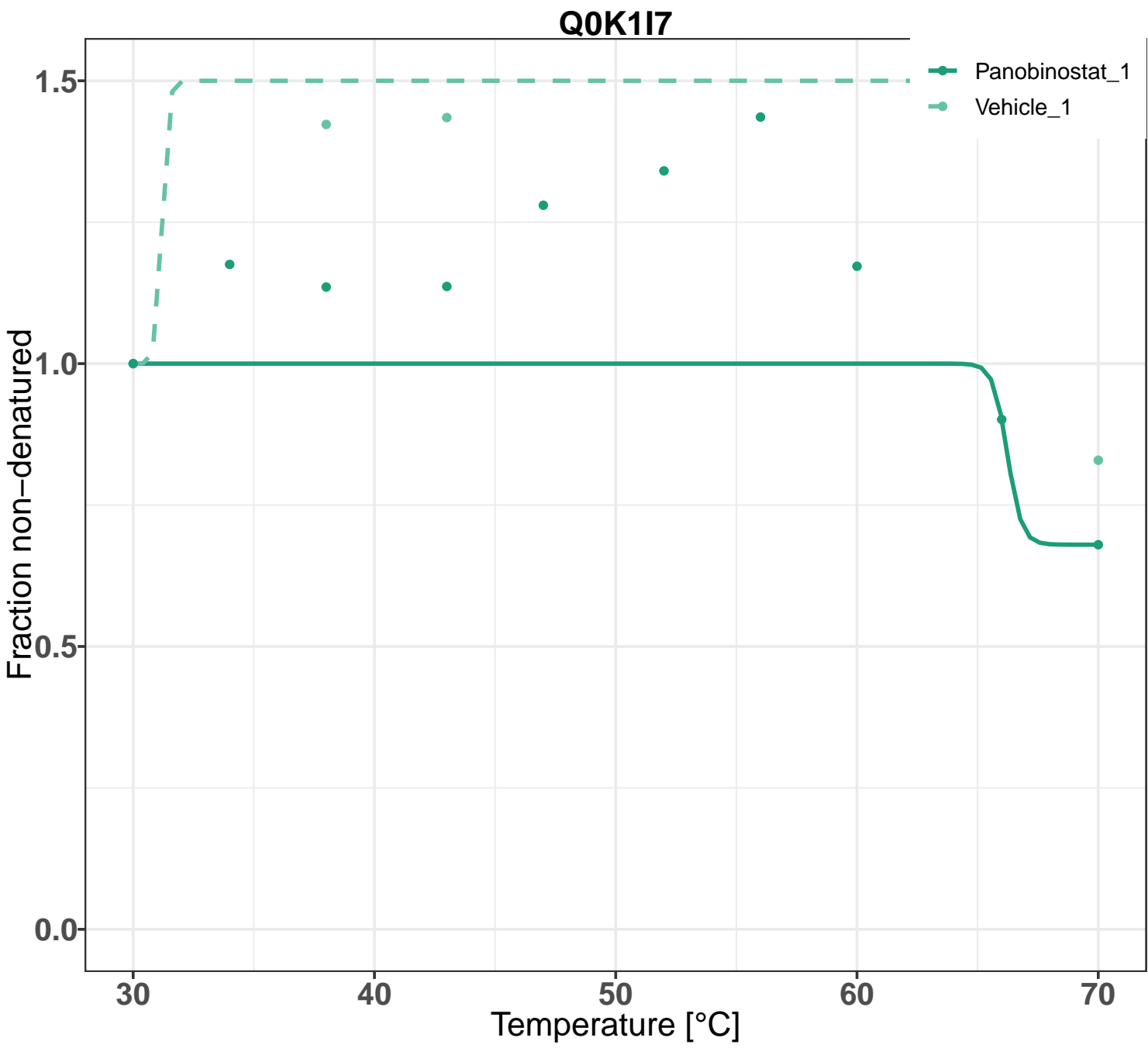

|                | meltPoint | slope | plateau | R2    |
|----------------|-----------|-------|---------|-------|
| Panobinostat_1 | –         | –0.27 | 0.68    | –0.1  |
| Vehicle_1      | –         | 1     | 1.5     | –0.16 |

Supplement: Supplementary file 2 — Supplementary Material 2 [file 41598_2026_35990_MOESM2_ESM.zip › AllTheTPPData/D40vD86/Panobinostat_Vignette/Melting_Curves/meltCurve_Q0K1I7.pdf]

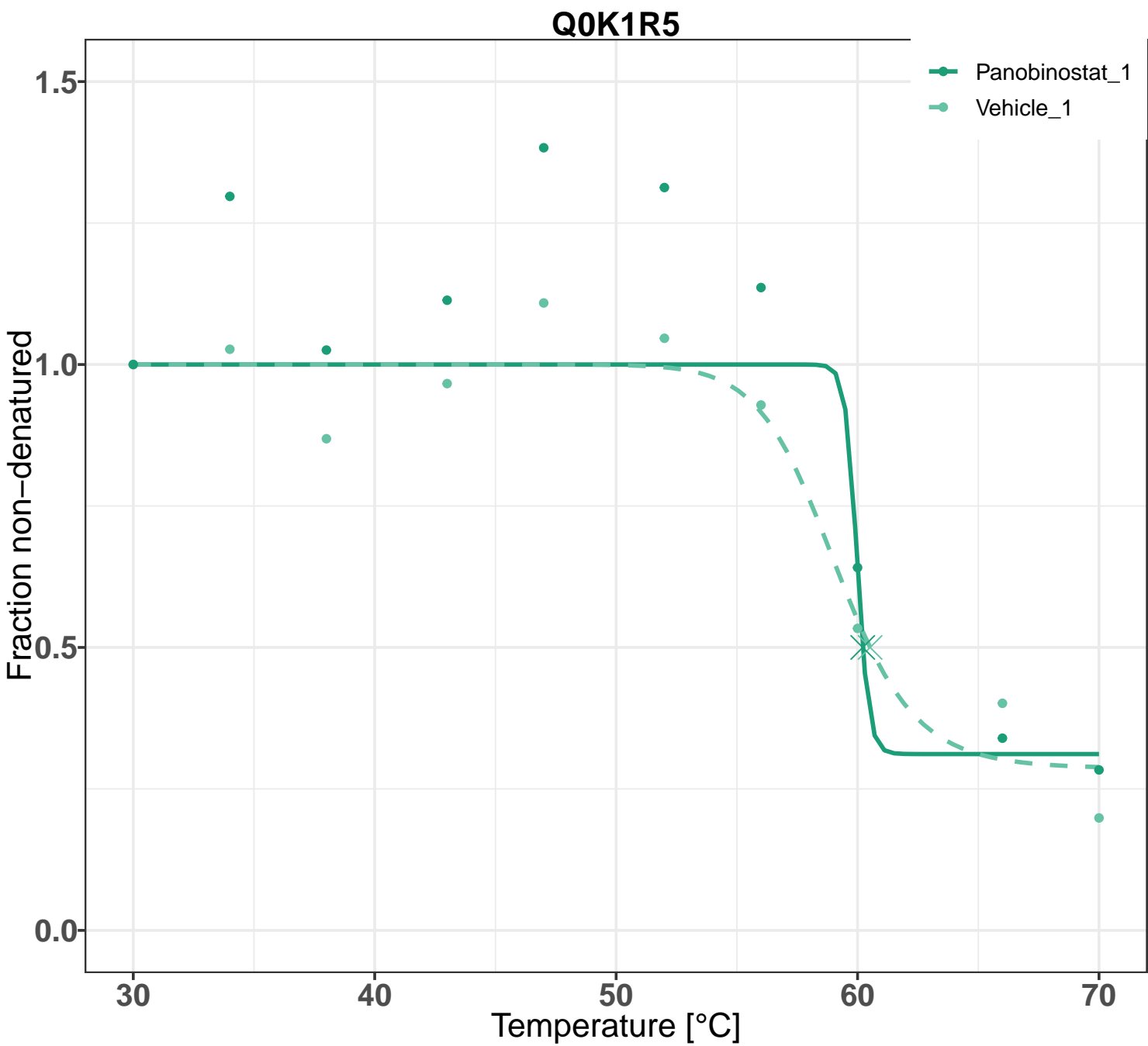

|                | meltPoint | slope | plateau | R2   |
|----------------|-----------|-------|---------|------|
| Panobinostat_1 | 60.21     | -0.72 | 0.31    | 0.74 |
| Vehicle_1      | 60.52     | -0.11 | 0.29    | 0.94 |

Supplement: Supplementary file 2 — Supplementary Material 2 [file 41598_2026_35990_MOESM2_ESM.zip › AllTheTPPData/D40vD86/Panobinostat_Vignette/Melting_Curves/meltCurve_Q0K1R5.pdf]

# Q0K1U9

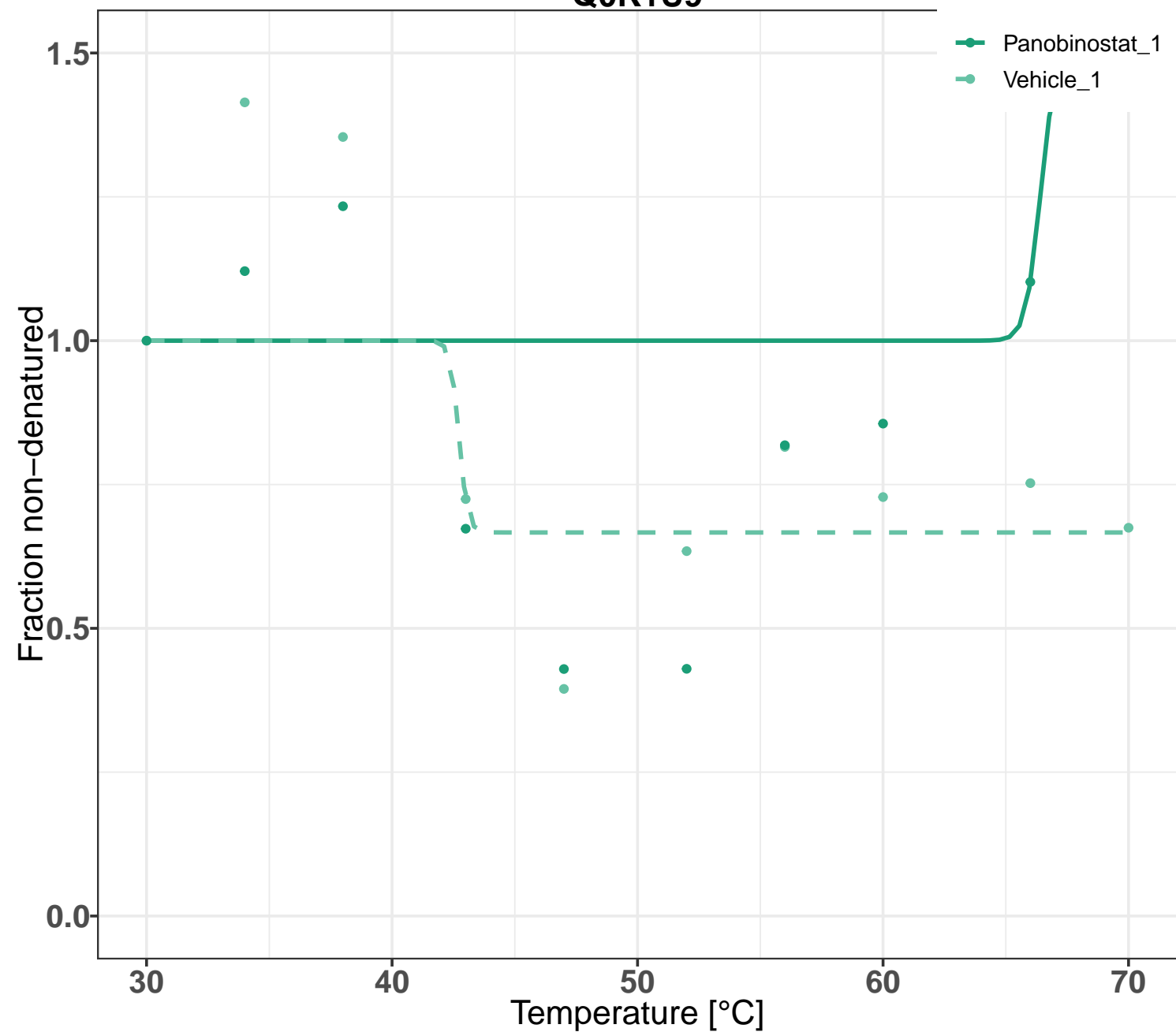

|                | meltPoint | slope | plateau | R2   |
|----------------|-----------|-------|---------|------|
| Panobinostat_1 | –         | 0.43  | 1.5     | 0.37 |
| Vehicle_1      | –         | -0.47 | 0.67    | 0.56 |

Supplement: Supplementary file 2 — Supplementary Material 2 [file 41598_2026_35990_MOESM2_ESM.zip › AllTheTPPData/D40vD86/Panobinostat_Vignette/Melting_Curves/meltCurve_Q0K1U9.pdf]

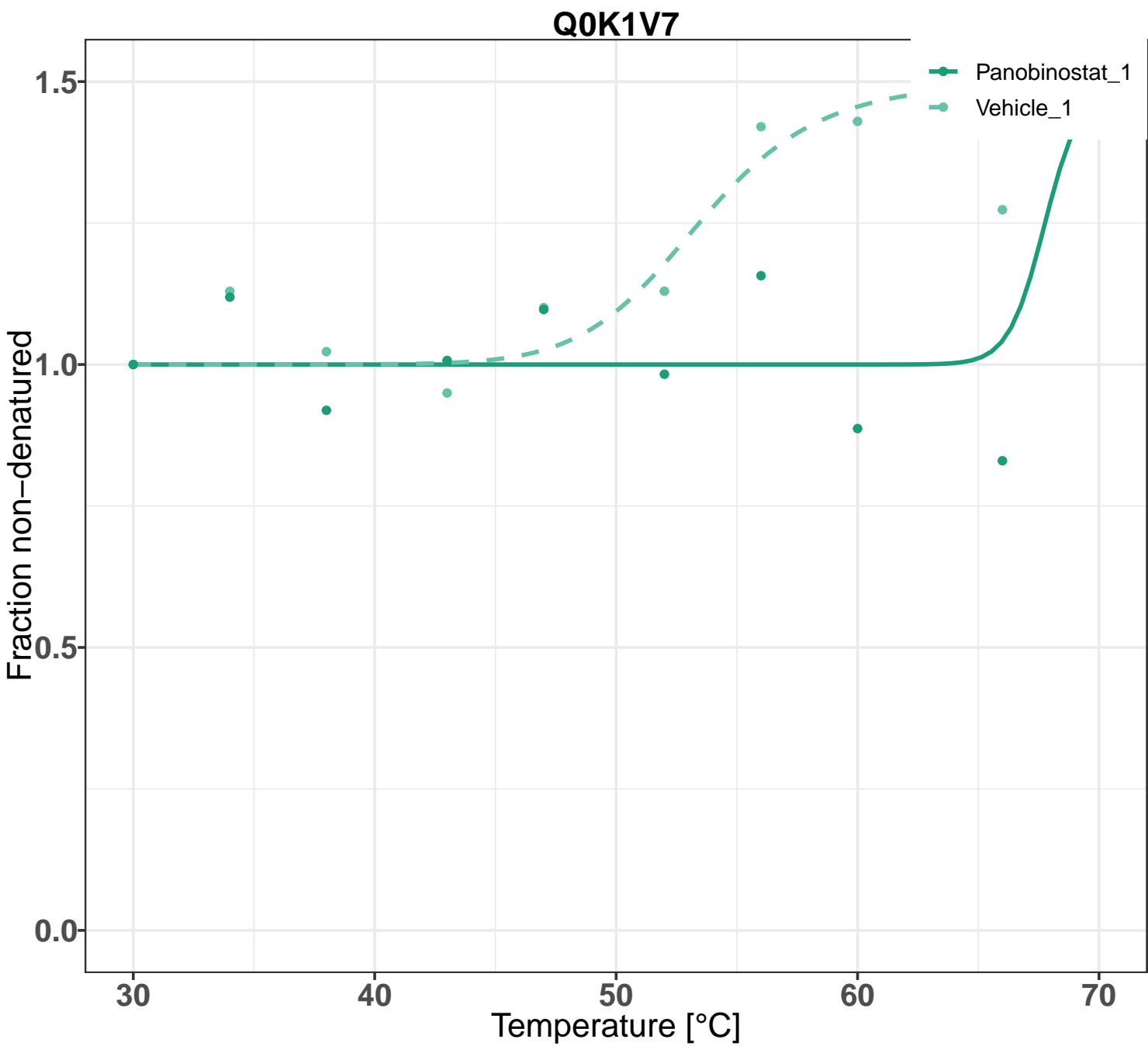

|                | meltPoint | slope | plateau | R2   |
|----------------|-----------|-------|---------|------|
| Panobinostat_1 | —         | 0.17  | 1.5     | 0.67 |
| Vehicle_1      | —         | 0.05  | 1.5     | 0.7  |

Supplement: Supplementary file 2 — Supplementary Material 2 [file 41598_2026_35990_MOESM2_ESM.zip › AllTheTPPData/D40vD86/Panobinostat_Vignette/Melting_Curves/meltCurve_Q0K1V7.pdf]

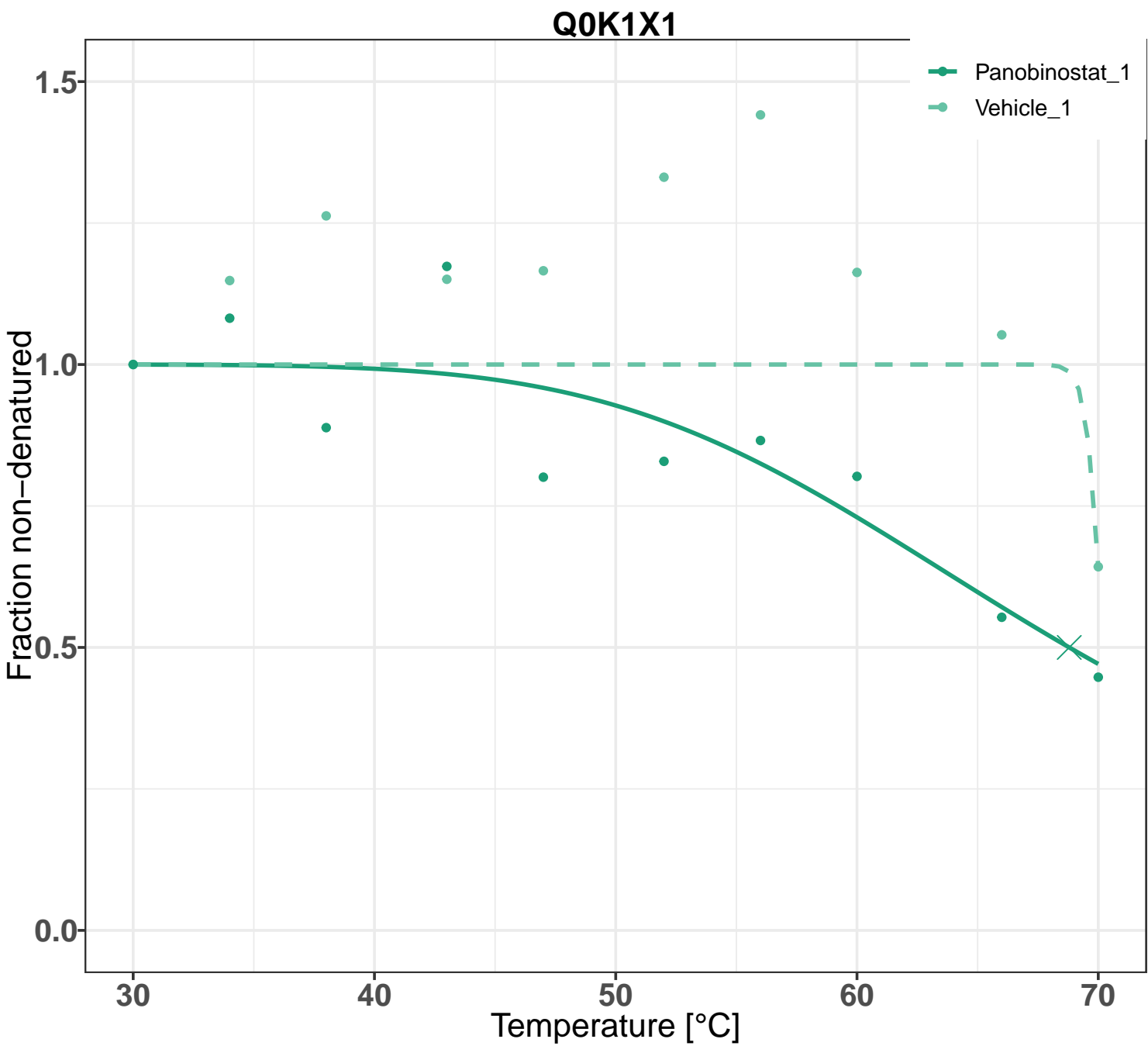

|                | meltPoint | slope  | plateau | R2    |
|----------------|-----------|--------|---------|-------|
| Panobinostat_1 | 68.8      | -0.027 | 0       | 0.79  |
| Vehicle_1      | -         | -      | 0       | -0.13 |

Supplement: Supplementary file 2 — Supplementary Material 2 [file 41598_2026_35990_MOESM2_ESM.zip › AllTheTPPData/D40vD86/Panobinostat_Vignette/Melting_Curves/meltCurve_Q0K1X1.pdf]

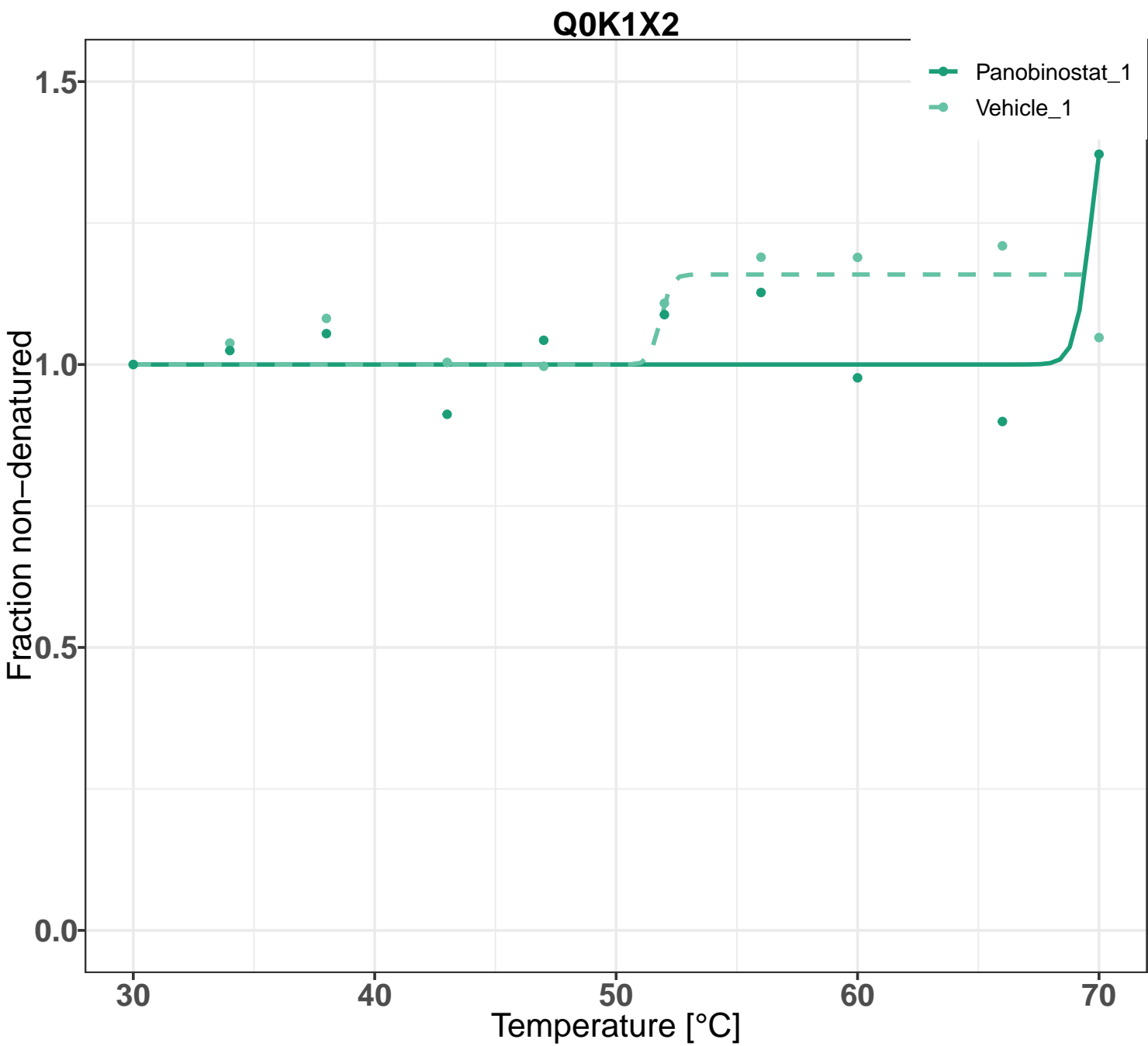

|                | meltPoint | slope | plateau | R2   |
|----------------|-----------|-------|---------|------|
| Panobinostat_1 | —         | 0.39  | 1.5     | 0.7  |
| Vehicle_1      | —         | 0.19  | 1.16    | 0.61 |

Supplement: Supplementary file 2 — Supplementary Material 2 [file 41598_2026_35990_MOESM2_ESM.zip › AllTheTPPData/D40vD86/Panobinostat_Vignette/Melting_Curves/meltCurve_Q0K1X2.pdf]

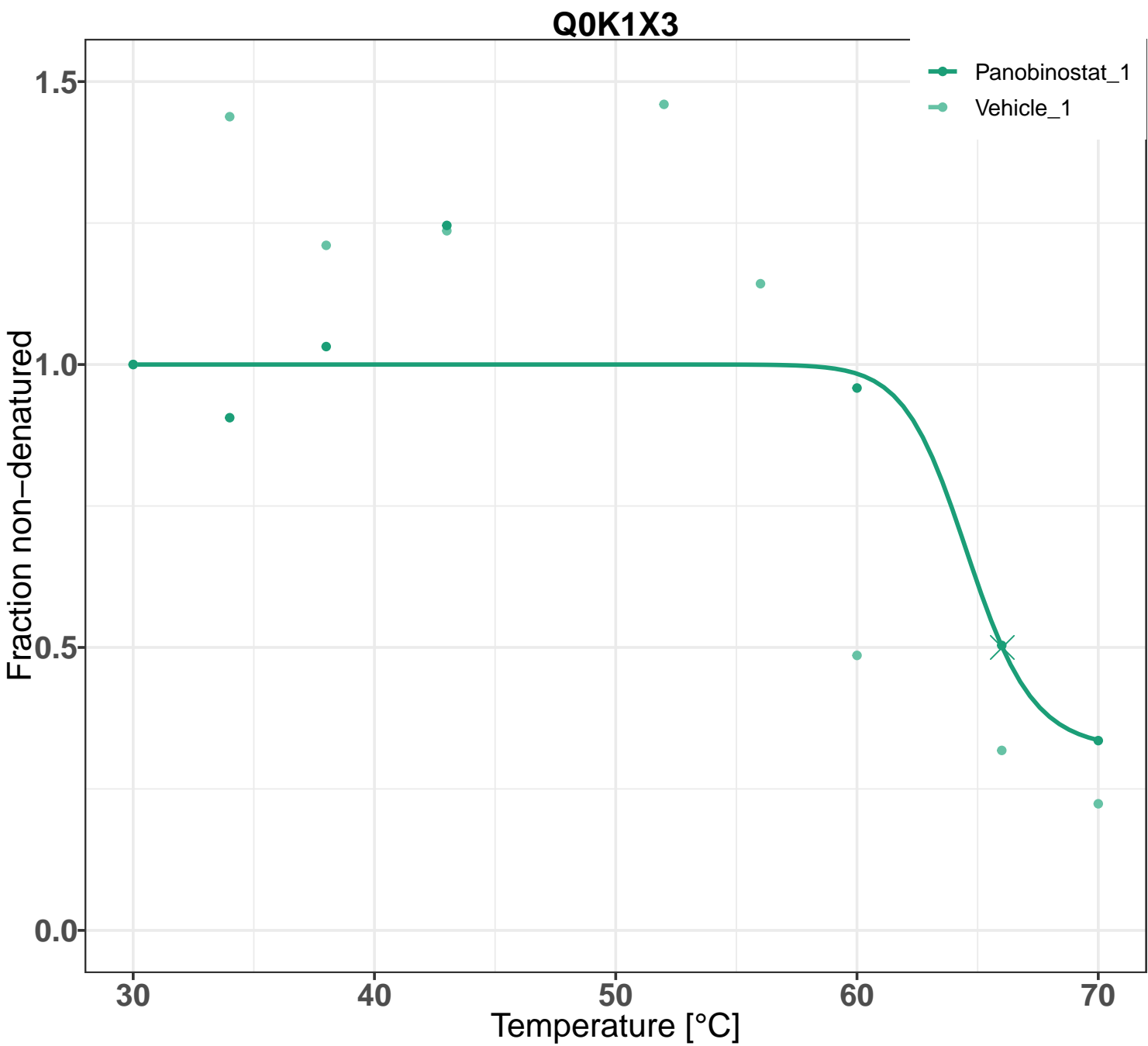

|                | meltPoint | slope | plateau | R2   |
|----------------|-----------|-------|---------|------|
| Panobinostat_1 | 66.02     | -0.13 | 0.32    | 0.43 |
| Vehicle_1      | —         | —     | —       | —    |

Supplement: Supplementary file 2 — Supplementary Material 2 [file 41598_2026_35990_MOESM2_ESM.zip › AllTheTPPData/D40vD86/Panobinostat_Vignette/Melting_Curves/meltCurve_Q0K1X3.pdf]

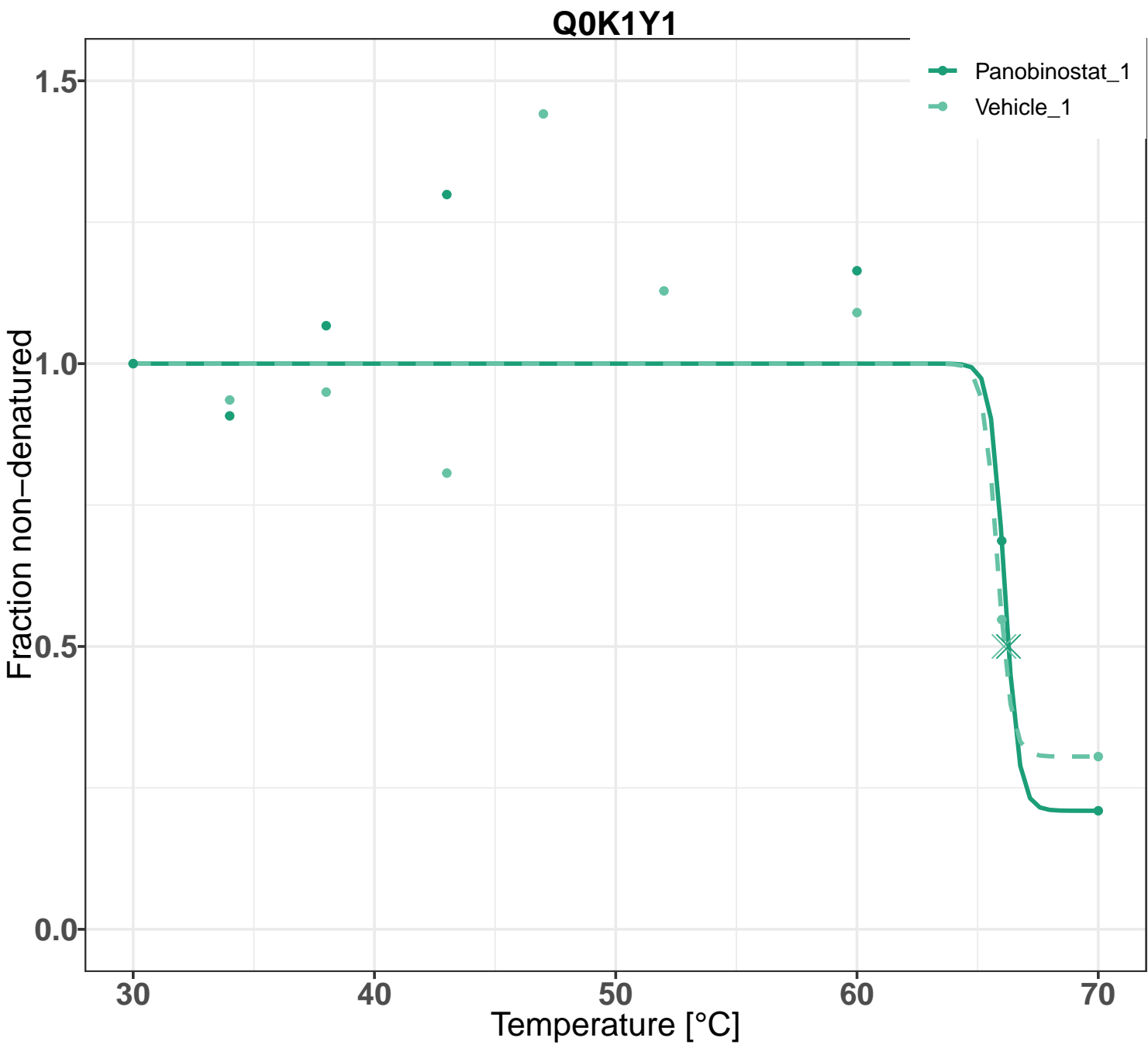

|                | meltPoint | slope | plateau | R2   |
|----------------|-----------|-------|---------|------|
| Panobinostat_1 | 66.28     | -0.68 | 0.21    | 0.25 |
| Vehicle_1      | 66.09     | -0.6  | 0.31    | 0.57 |

Supplement: Supplementary file 2 — Supplementary Material 2 [file 41598_2026_35990_MOESM2_ESM.zip › AllTheTPPData/D40vD86/Panobinostat_Vignette/Melting_Curves/meltCurve_Q0K1Y1.pdf]

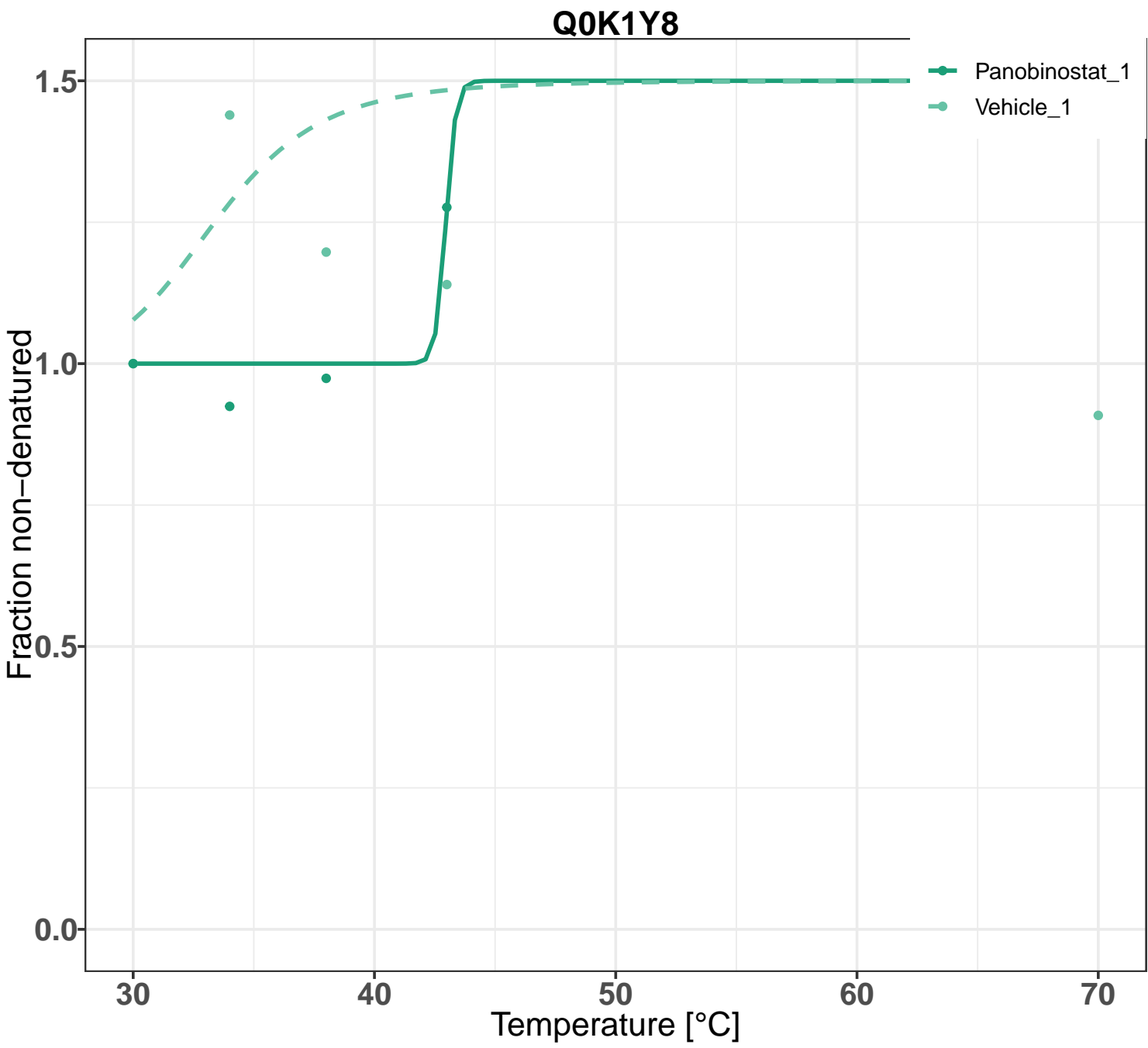

|                | meltPoint | slope | plateau | R2    |
|----------------|-----------|-------|---------|-------|
| Panobinostat_1 | —         | 0.61  | 1.5     | 0.36  |
| Vehicle_1      | —         | 0.057 | 1.5     | -0.09 |

Supplement: Supplementary file 2 — Supplementary Material 2 [file 41598_2026_35990_MOESM2_ESM.zip › AllTheTPPData/D40vD86/Panobinostat_Vignette/Melting_Curves/meltCurve_Q0K1Y8.pdf]

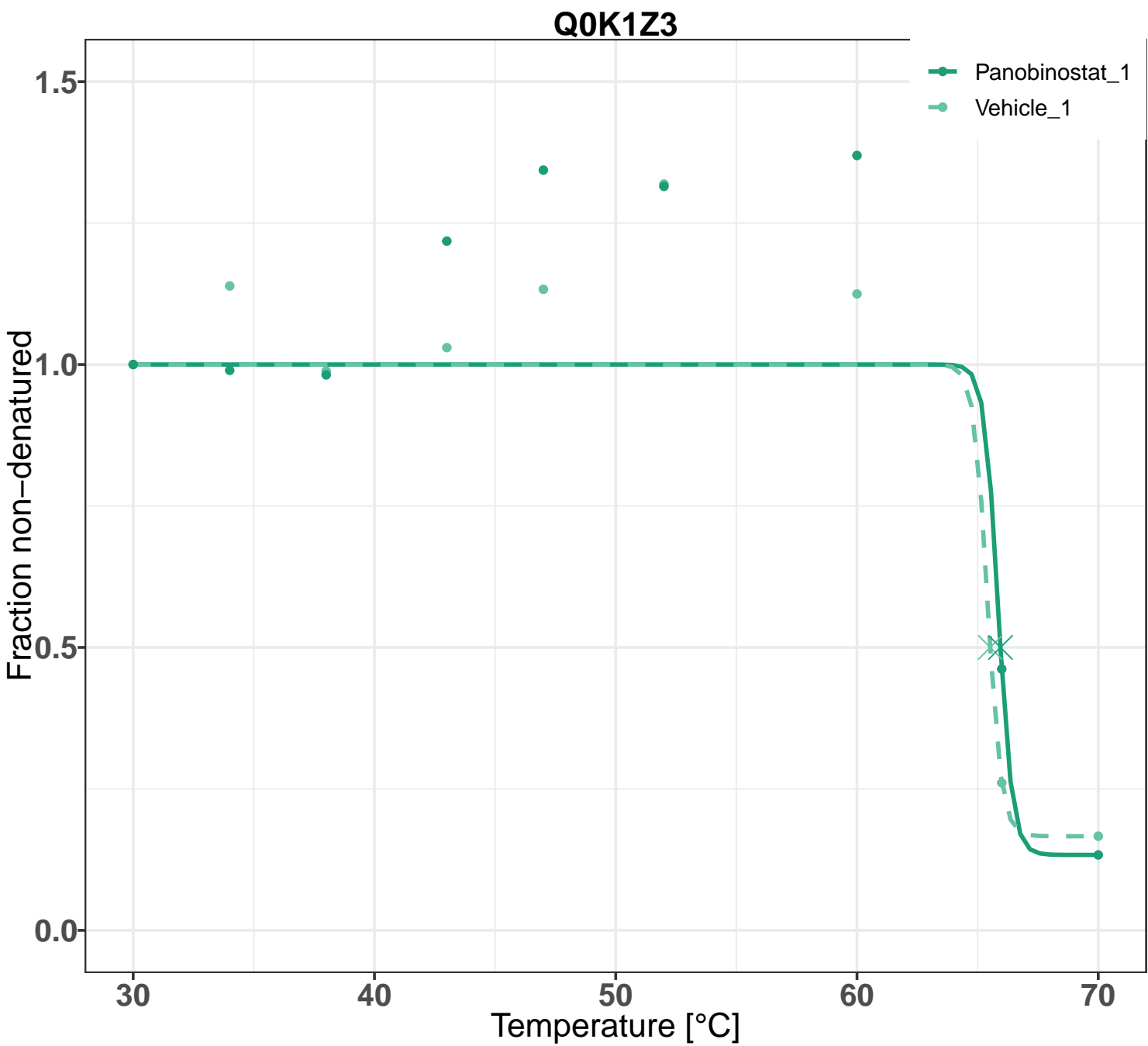

|                | meltPoint | slope | plateau | R2   |
|----------------|-----------|-------|---------|------|
| Panobinostat_1 | 65.95     | -0.75 | 0.13    | 0.59 |
| Vehicle_1      | 65.52     | -0.73 | 0.17    | 0.64 |

Supplement: Supplementary file 2 — Supplementary Material 2 [file 41598_2026_35990_MOESM2_ESM.zip › AllTheTPPData/D40vD86/Panobinostat_Vignette/Melting_Curves/meltCurve_Q0K1Z3.pdf]

# Q0K1Z4

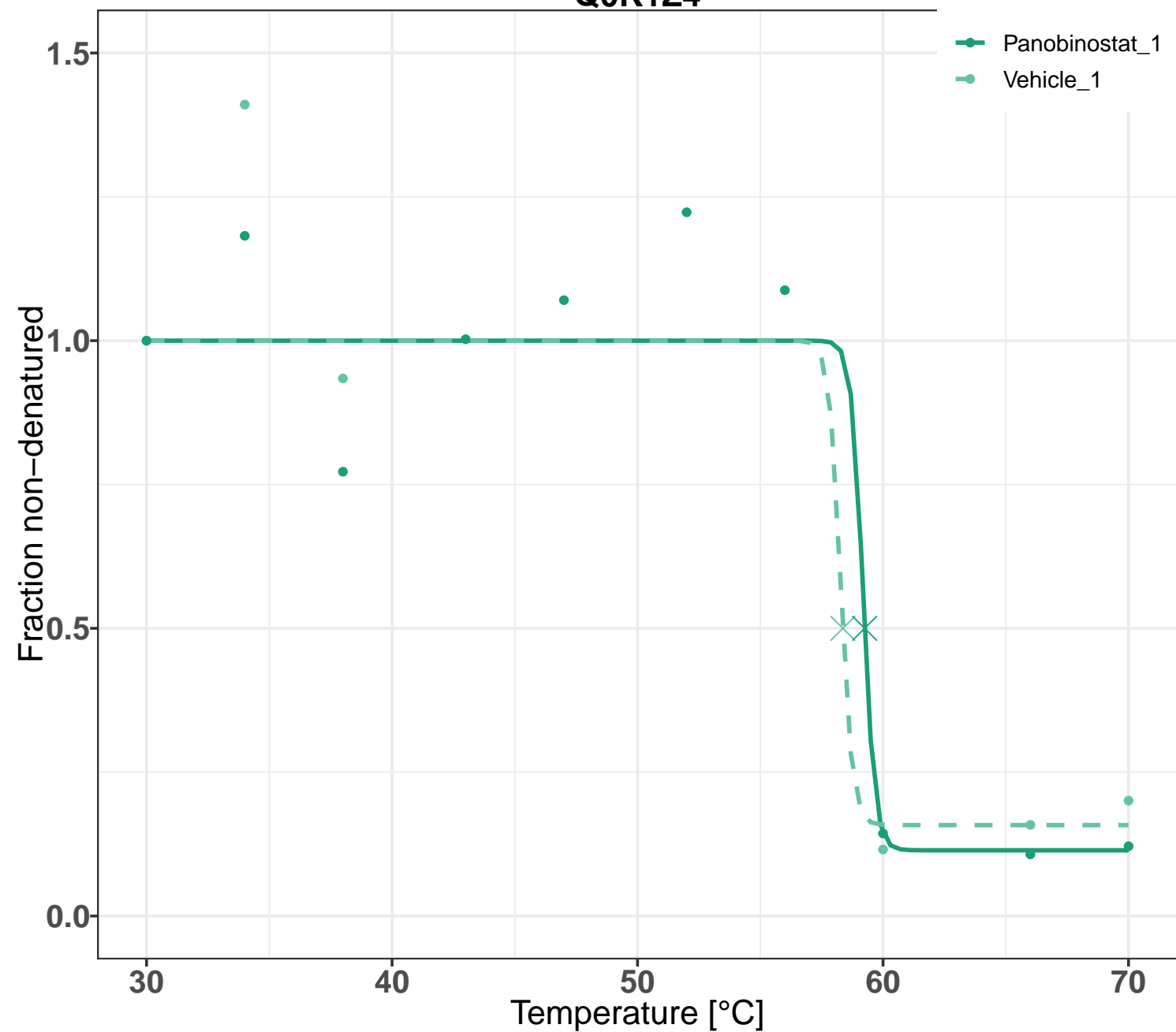

meltPoint

slope

plateau

R2

Panobinostat\_1

59.25

-0.94

0.11

0.92

Vehicle\_1

58.36

-0.9

0.16

0.38

Supplement: Supplementary file 2 — Supplementary Material 2 [file 41598_2026_35990_MOESM2_ESM.zip › AllTheTPPData/D40vD86/Panobinostat_Vignette/Melting_Curves/meltCurve_Q0K1Z4.pdf]

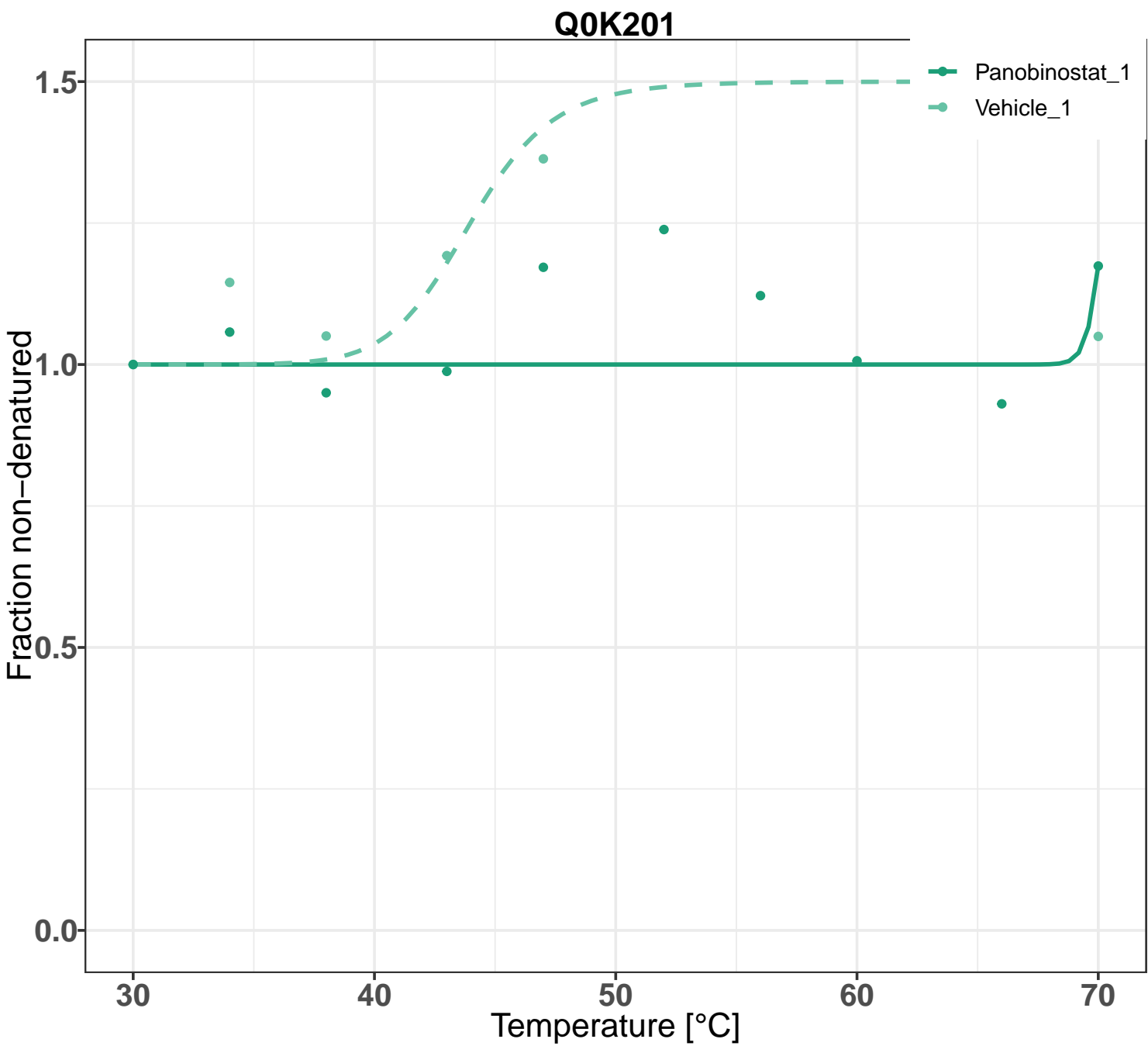

|                | meltPoint | slope | plateau | R2   |
|----------------|-----------|-------|---------|------|
| Panobinostat_1 | –         | –     | 1.5     | –0.1 |
| Vehicle_1      | –         | 0.073 | 1.5     | 0.49 |

Supplement: Supplementary file 2 — Supplementary Material 2 [file 41598_2026_35990_MOESM2_ESM.zip › AllTheTPPData/D40vD86/Panobinostat_Vignette/Melting_Curves/meltCurve_Q0K201.pdf]

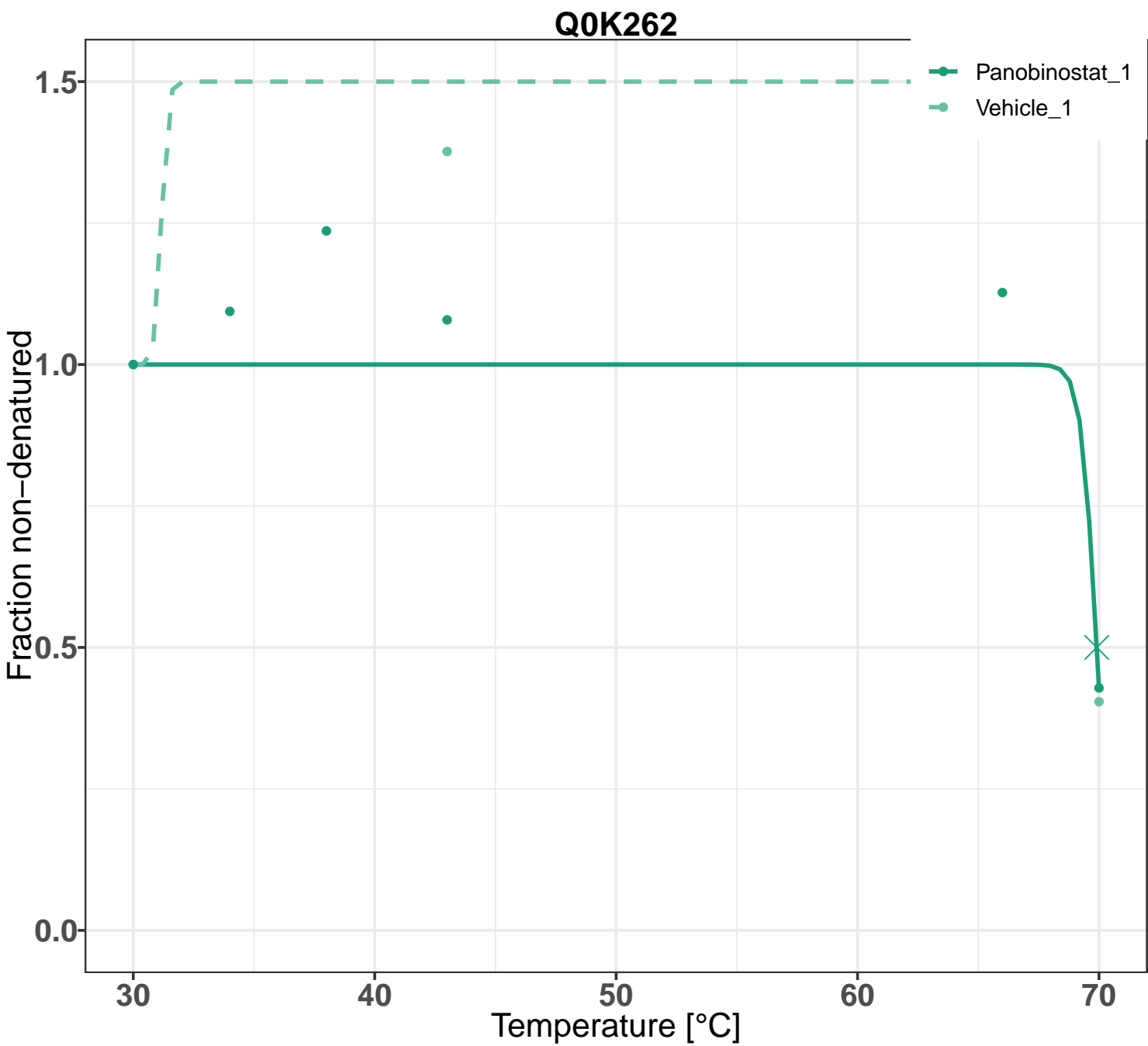

|                | meltPoint | slope | plateau | R2    |
|----------------|-----------|-------|---------|-------|
| Panobinostat_1 | 69.91     | -0.77 | 0       | -0.45 |
| Vehicle_1      | -         | 1     | 1.5     | -0.49 |

Supplement: Supplementary file 2 — Supplementary Material 2 [file 41598_2026_35990_MOESM2_ESM.zip › AllTheTPPData/D40vD86/Panobinostat_Vignette/Melting_Curves/meltCurve_Q0K262.pdf]

# Q0K280

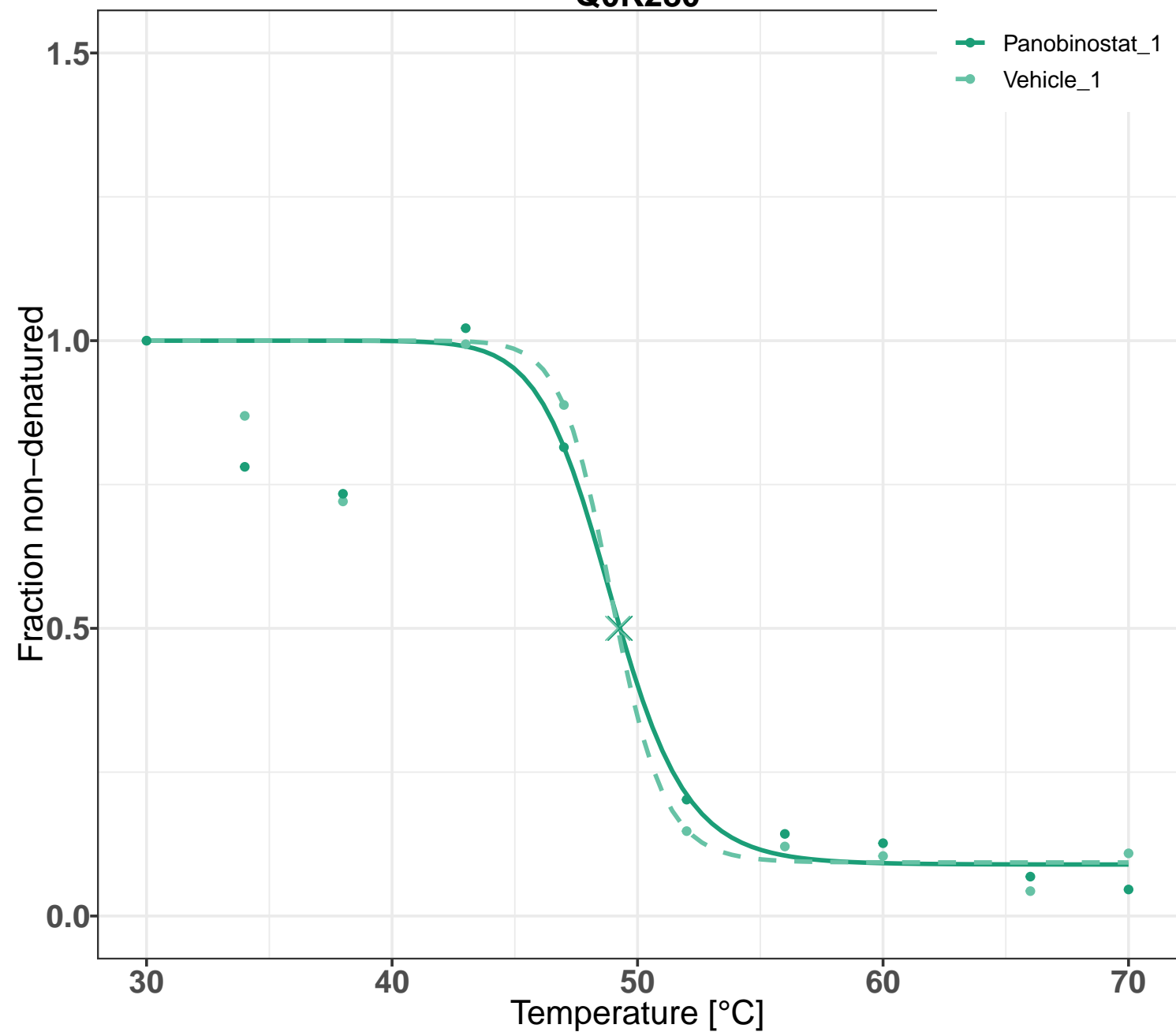

**meltPoint**

**slope**

**plateau**

**R2**

**Panobinostat\_1**

**49.29**

**-0.15**

**0.09**

**0.92**

**Vehicle\_1**

**49.19**

**-0.22**

**0.09**

**0.94**

Supplement: Supplementary file 2 — Supplementary Material 2 [file 41598_2026_35990_MOESM2_ESM.zip › AllTheTPPData/D40vD86/Panobinostat_Vignette/Melting_Curves/meltCurve_Q0K280.pdf]

# Q0K287

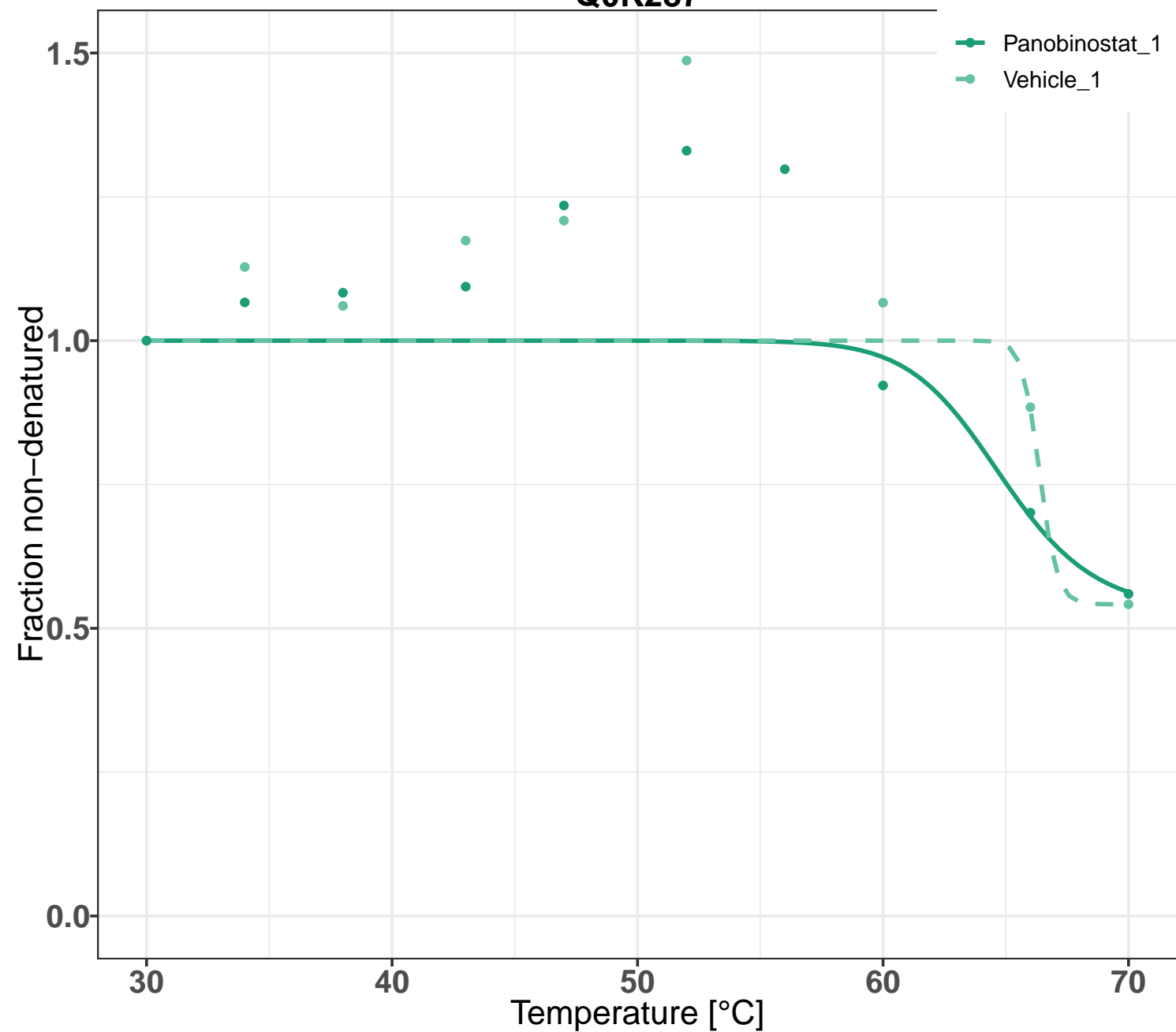

|                | meltPoint | slope  | plateau | R2   |
|----------------|-----------|--------|---------|------|
| Panobinostat_1 | –         | –0.062 | 0.53    | 0.5  |
| Vehicle_1      | –         | –0.33  | 0.54    | 0.16 |

Supplement: Supplementary file 2 — Supplementary Material 2 [file 41598_2026_35990_MOESM2_ESM.zip › AllTheTPPData/D40vD86/Panobinostat_Vignette/Melting_Curves/meltCurve_Q0K287.pdf]

# Q0K288

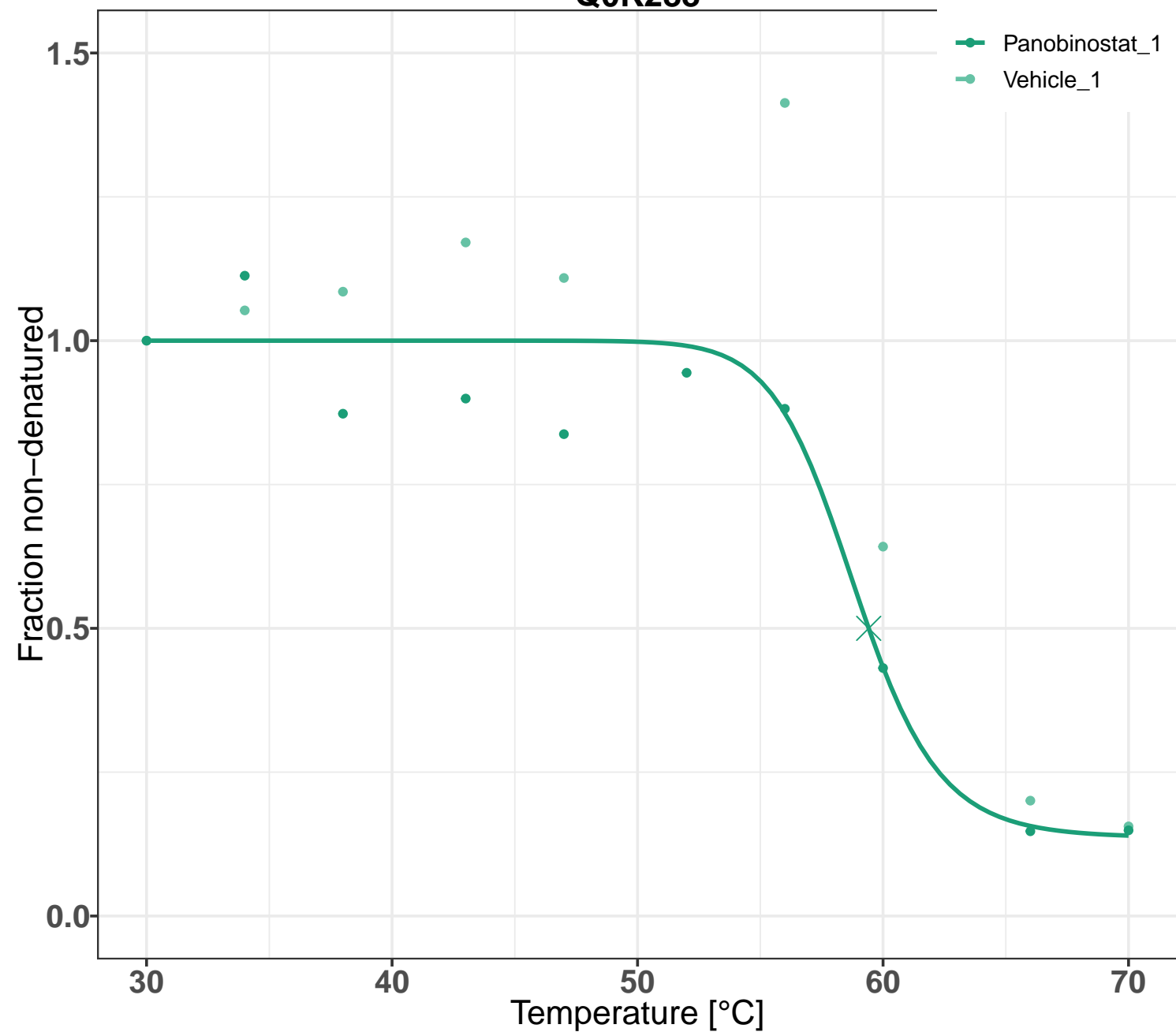

**meltPoint**

**slope**

**plateau**

**R2**

**Panobinostat\_1**

**59.42**

**-0.13**

**0.14**

**0.94**

**Vehicle\_1**

—

—

—

—

Supplement: Supplementary file 2 — Supplementary Material 2 [file 41598_2026_35990_MOESM2_ESM.zip › AllTheTPPData/D40vD86/Panobinostat_Vignette/Melting_Curves/meltCurve_Q0K288.pdf]

# Q0K2B4

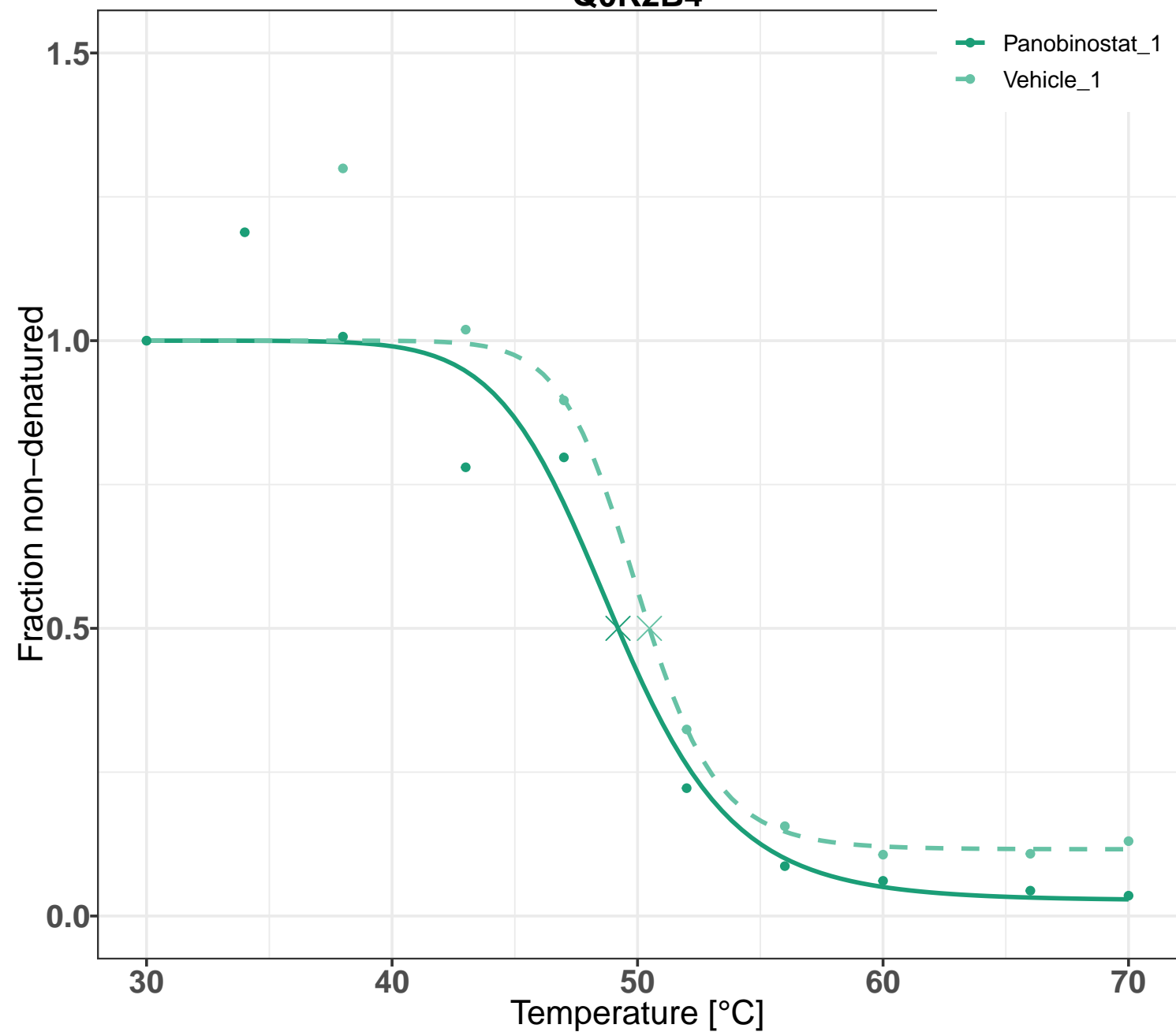

meltPoint

slope

plateau

R2

Panobinostat\_1

49.2

-0.1

0.03

0.96

Vehicle\_1

50.48

-0.14

0.12

0.82

Supplement: Supplementary file 2 — Supplementary Material 2 [file 41598_2026_35990_MOESM2_ESM.zip › AllTheTPPData/D40vD86/Panobinostat_Vignette/Melting_Curves/meltCurve_Q0K2B4.pdf]

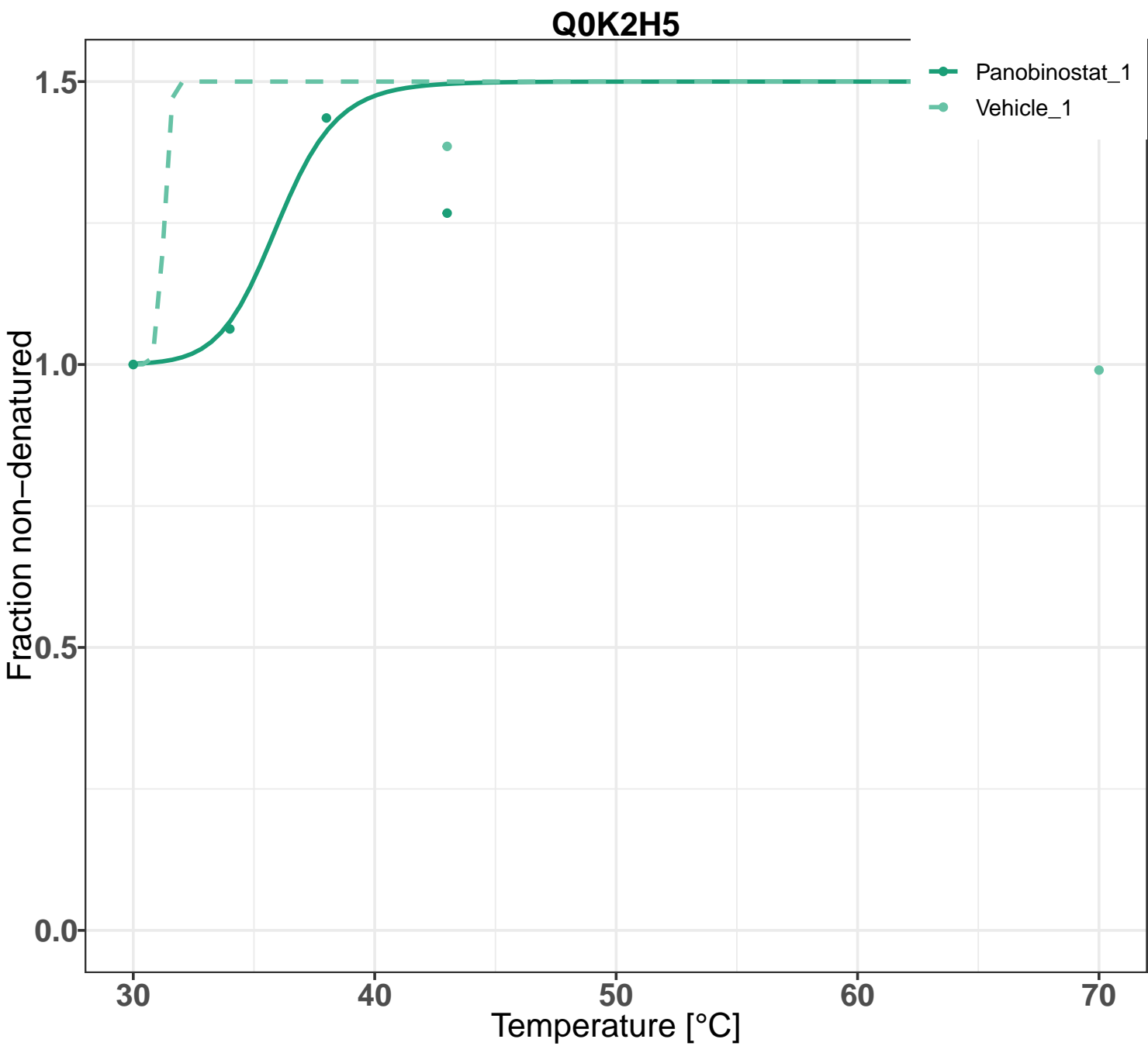

|                | meltPoint | slope | plateau | R2    |
|----------------|-----------|-------|---------|-------|
| Panobinostat_1 | –         | 0.1   | 1.5     | 0.22  |
| Vehicle_1      | –         | 1     | 1.5     | –0.36 |

Supplement: Supplementary file 2 — Supplementary Material 2 [file 41598_2026_35990_MOESM2_ESM.zip › AllTheTPPData/D40vD86/Panobinostat_Vignette/Melting_Curves/meltCurve_Q0K2H5.pdf]

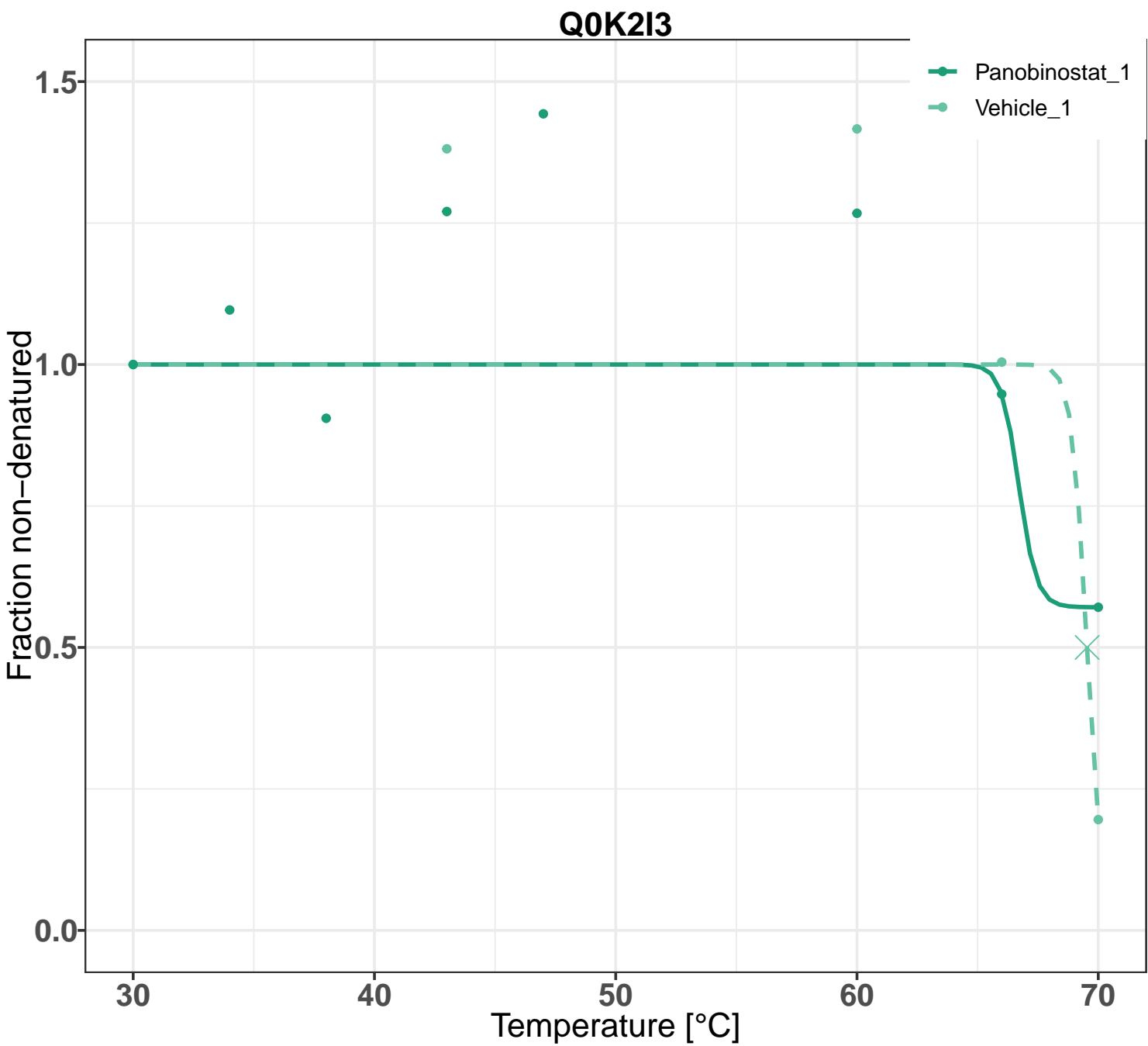

|                | meltPoint | slope | plateau | R2    |
|----------------|-----------|-------|---------|-------|
| Panobinostat_1 | –         | –0.29 | 0.57    | –0.11 |
| Vehicle_1      | 69.54     | –0.77 | 0       | –0.43 |

Supplement: Supplementary file 2 — Supplementary Material 2 [file 41598_2026_35990_MOESM2_ESM.zip › AllTheTPPData/D40vD86/Panobinostat_Vignette/Melting_Curves/meltCurve_Q0K2I3.pdf]

# Q0K2N8

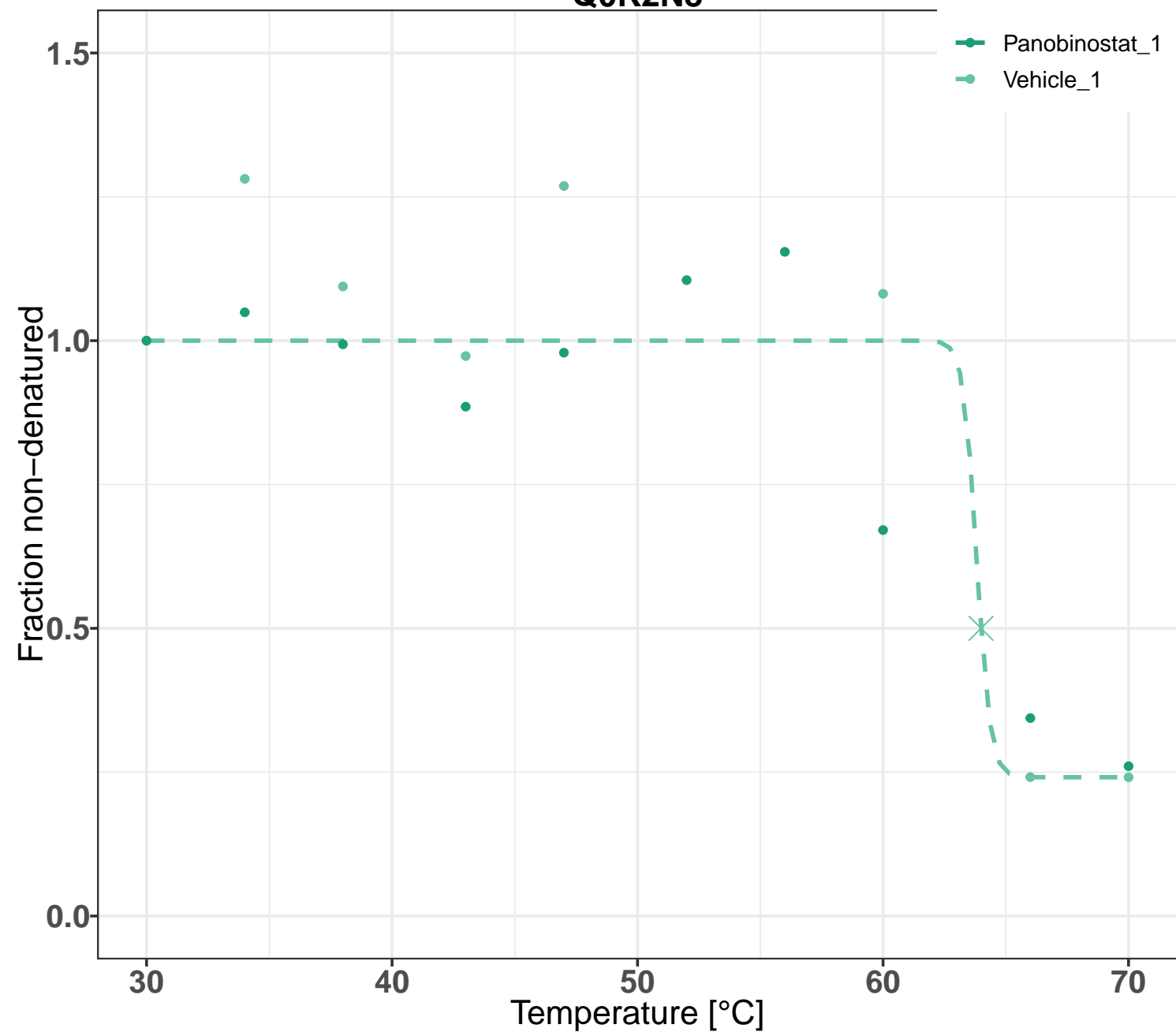

**meltPoint**

**slope**

**plateau**

**R2**

**Panobinostat\_1**

–

–

–

–

**Vehicle\_1**

**63.99**

**–0.7**

**0.24**

**0.42**

Supplement: Supplementary file 2 — Supplementary Material 2 [file 41598_2026_35990_MOESM2_ESM.zip › AllTheTPPData/D40vD86/Panobinostat_Vignette/Melting_Curves/meltCurve_Q0K2N8.pdf]

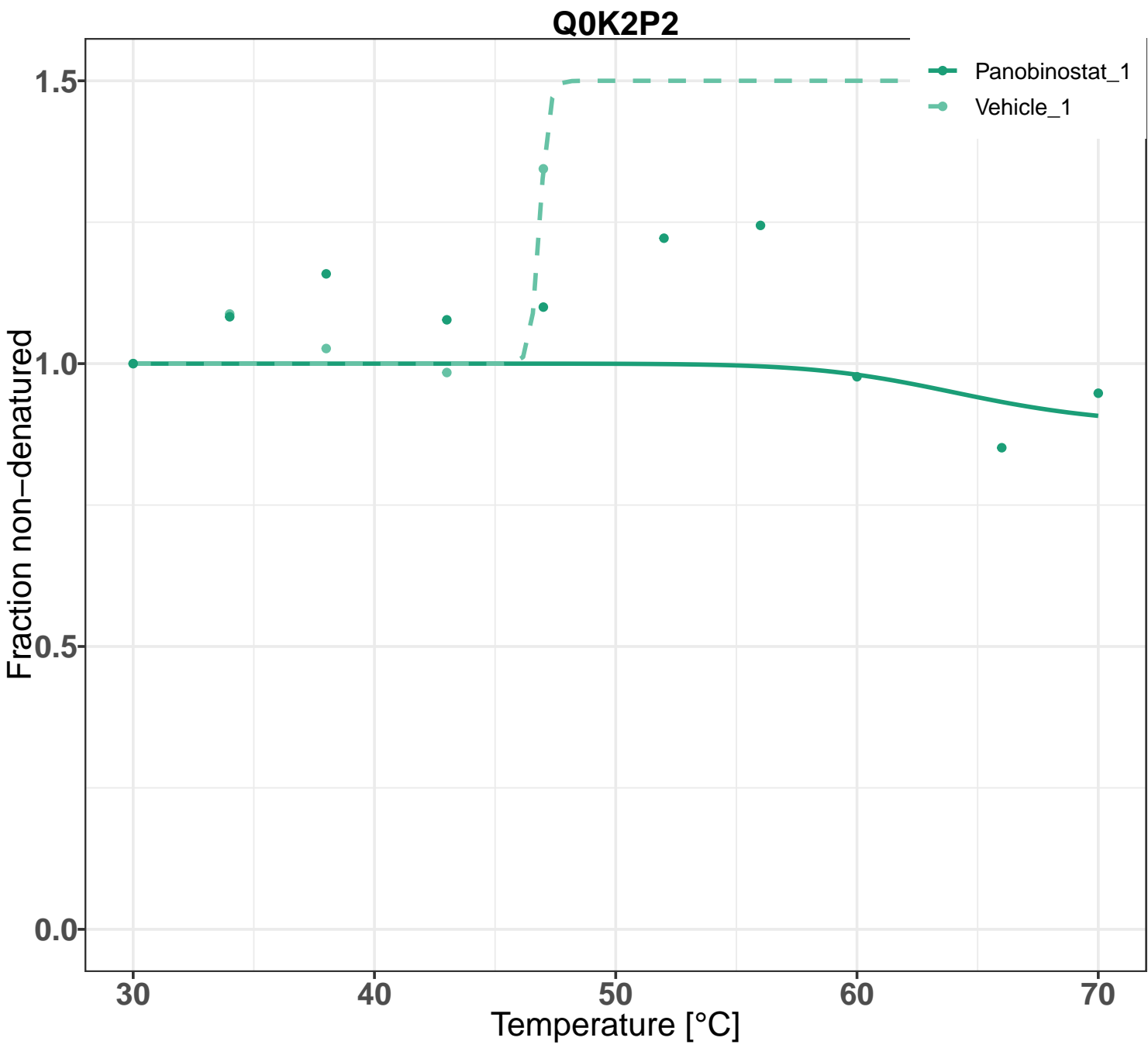

|                | meltPoint | slope   | plateau | R2    |
|----------------|-----------|---------|---------|-------|
| Panobinostat_1 | –         | –0.0088 | 0.89    | –0.21 |
| Vehicle_1      | –         | 0.67    | 1.5     | 0.63  |

Supplement: Supplementary file 2 — Supplementary Material 2 [file 41598_2026_35990_MOESM2_ESM.zip › AllTheTPPData/D40vD86/Panobinostat_Vignette/Melting_Curves/meltCurve_Q0K2P2.pdf]

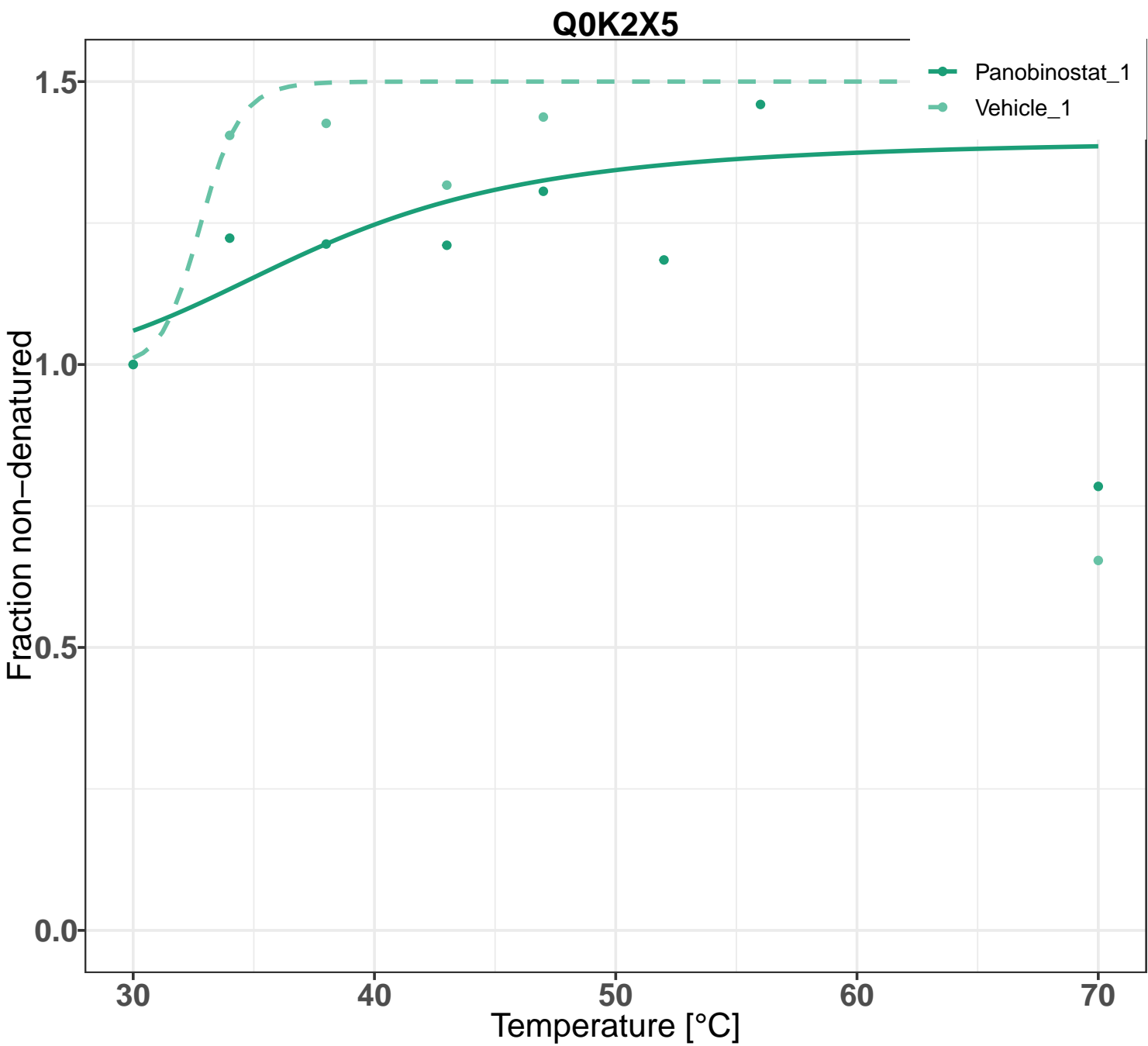

|                | meltPoint | slope | plateau | R2   |
|----------------|-----------|-------|---------|------|
| Panobinostat_1 | –         | 0.021 | 1.4     | 0.15 |
| Vehicle_1      | –         | 0.15  | 1.5     | 0.06 |

Supplement: Supplementary file 2 — Supplementary Material 2 [file 41598_2026_35990_MOESM2_ESM.zip › AllTheTPPData/D40vD86/Panobinostat_Vignette/Melting_Curves/meltCurve_Q0K2X5.pdf]

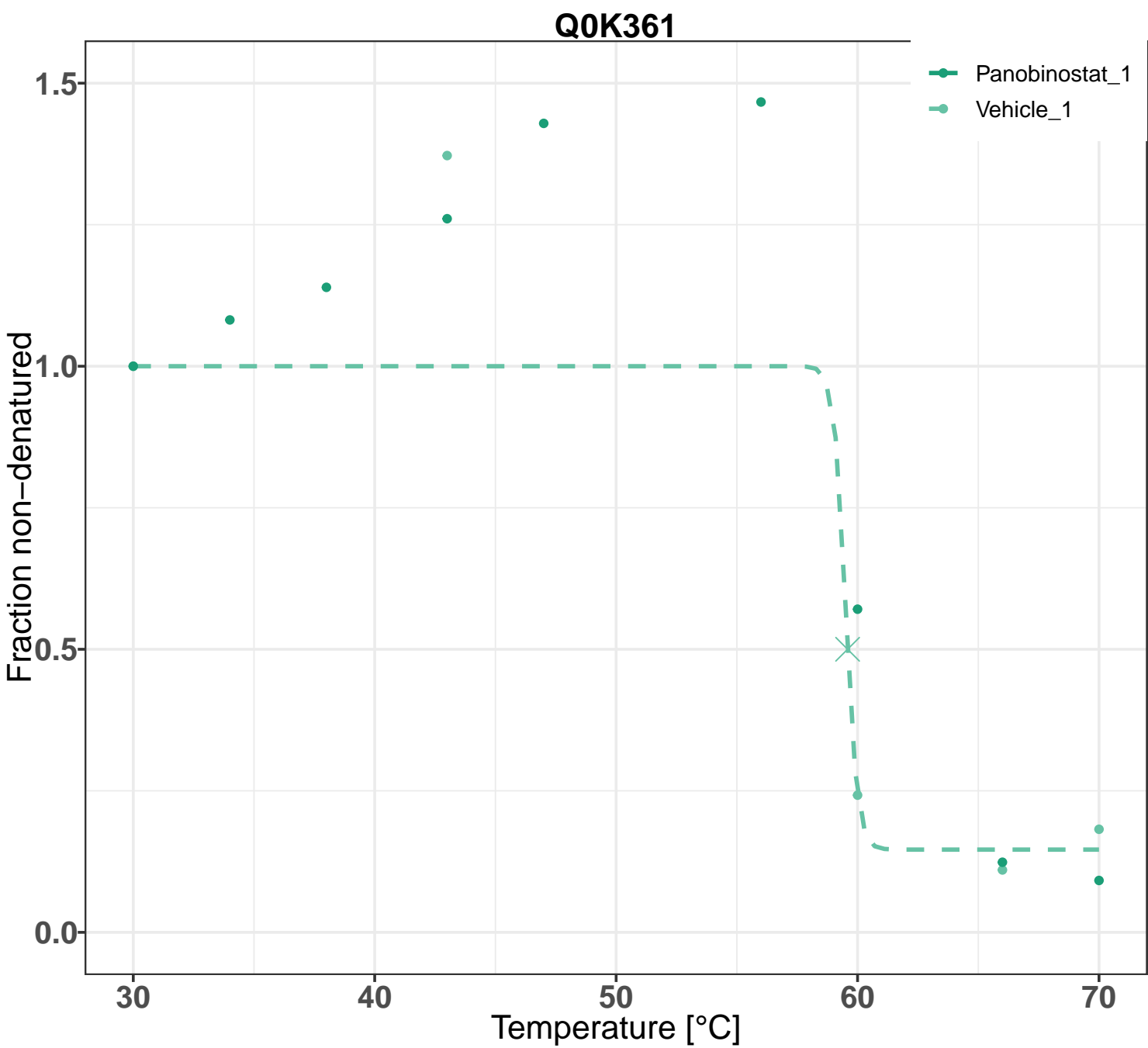

|                | meltPoint | slope | plateau | R2   |
|----------------|-----------|-------|---------|------|
| Panobinostat_1 | -         | -     | -       | -    |
| Vehicle_1      | 59.59     | -0.9  | 0.15    | 0.14 |

Supplement: Supplementary file 2 — Supplementary Material 2 [file 41598_2026_35990_MOESM2_ESM.zip › AllTheTPPData/D40vD86/Panobinostat_Vignette/Melting_Curves/meltCurve_Q0K361.pdf]

# Q0K3C7

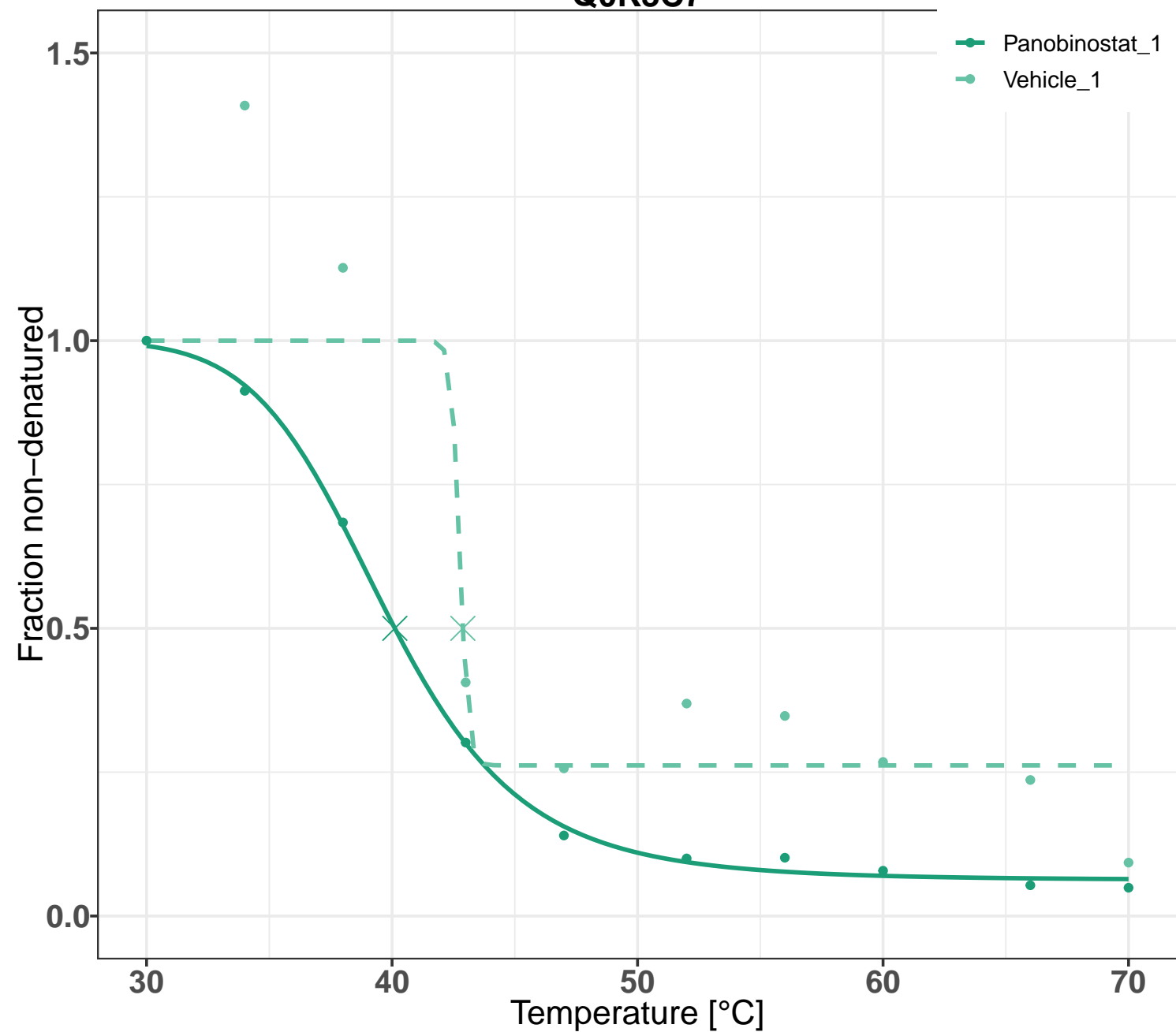

**meltPoint**

**slope**

**plateau**

**R2**

**Panobinostat\_1**

**40.11**

**-0.086**

**0.06**

**1**

**Vehicle\_1**

**42.88**

**-1.1**

**0.26**

**0.87**

Supplement: Supplementary file 2 — Supplementary Material 2 [file 41598_2026_35990_MOESM2_ESM.zip › AllTheTPPData/D40vD86/Panobinostat_Vignette/Melting_Curves/meltCurve_Q0K3C7.pdf]

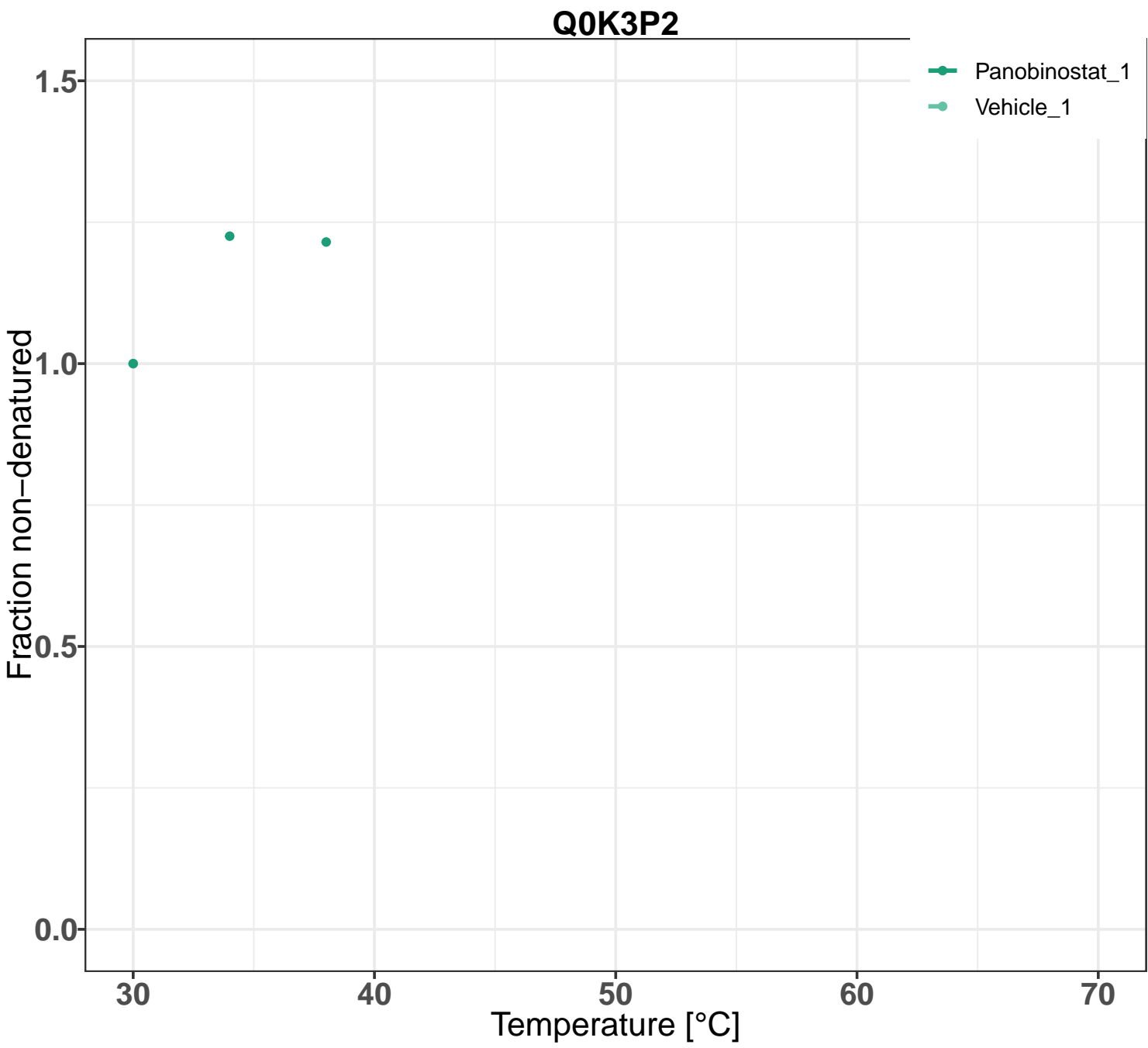

|                | meltPoint | slope | plateau | R2 |
|----------------|-----------|-------|---------|----|
| Panobinostat_1 | —         | —     | —       | —  |
| Vehicle_1      | —         | —     | —       | —  |

Supplement: Supplementary file 2 — Supplementary Material 2 [file 41598_2026_35990_MOESM2_ESM.zip › AllTheTPPData/D40vD86/Panobinostat_Vignette/Melting_Curves/meltCurve_Q0K3P2.pdf]

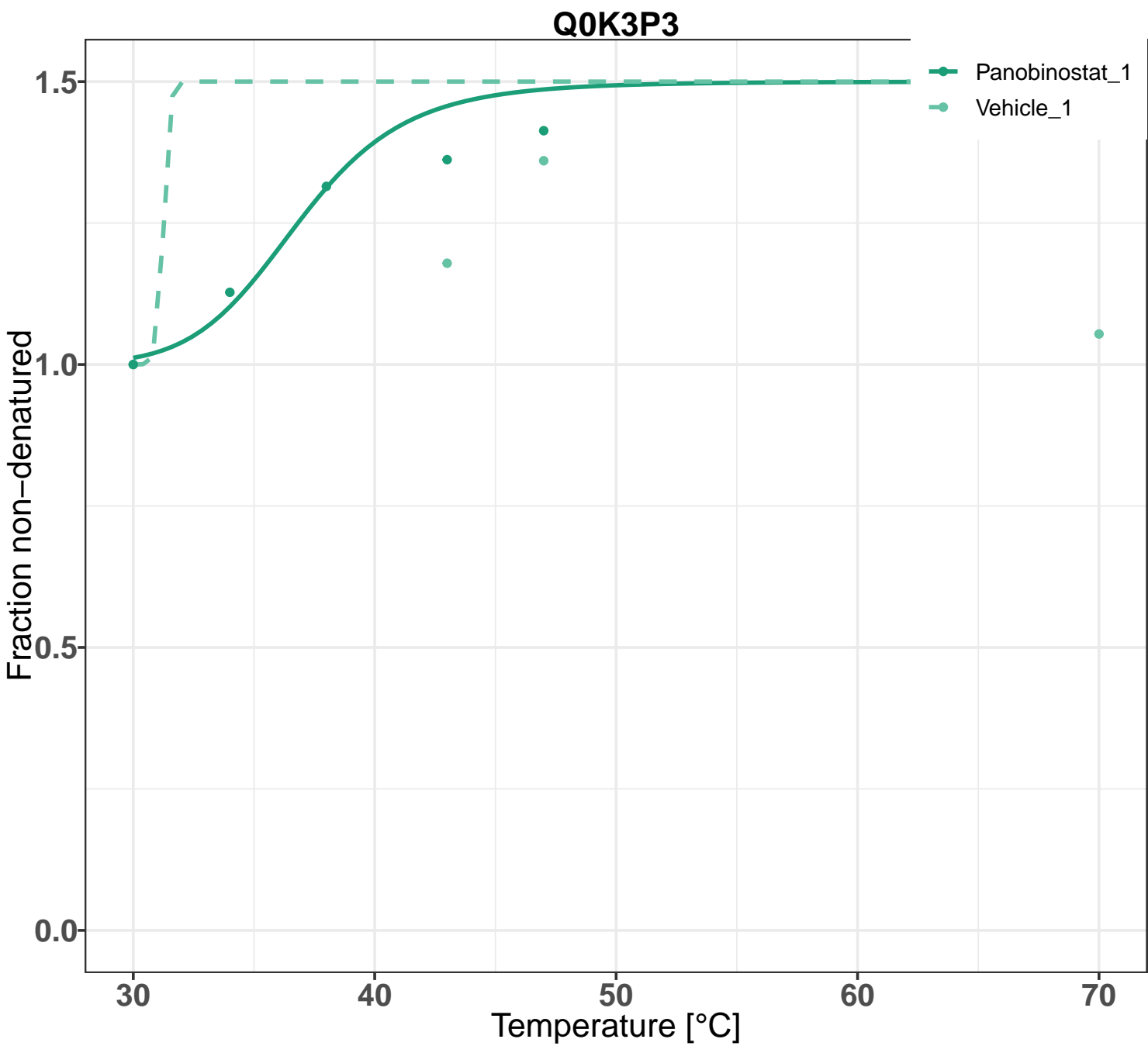

|                | meltPoint | slope | plateau | R2    |
|----------------|-----------|-------|---------|-------|
| Panobinostat_1 | –         | 0.057 | 1.5     | 0.22  |
| Vehicle_1      | –         | 1     | 1.5     | –0.21 |

Supplement: Supplementary file 2 — Supplementary Material 2 [file 41598_2026_35990_MOESM2_ESM.zip › AllTheTPPData/D40vD86/Panobinostat_Vignette/Melting_Curves/meltCurve_Q0K3P3.pdf]

# Q0K3Q9

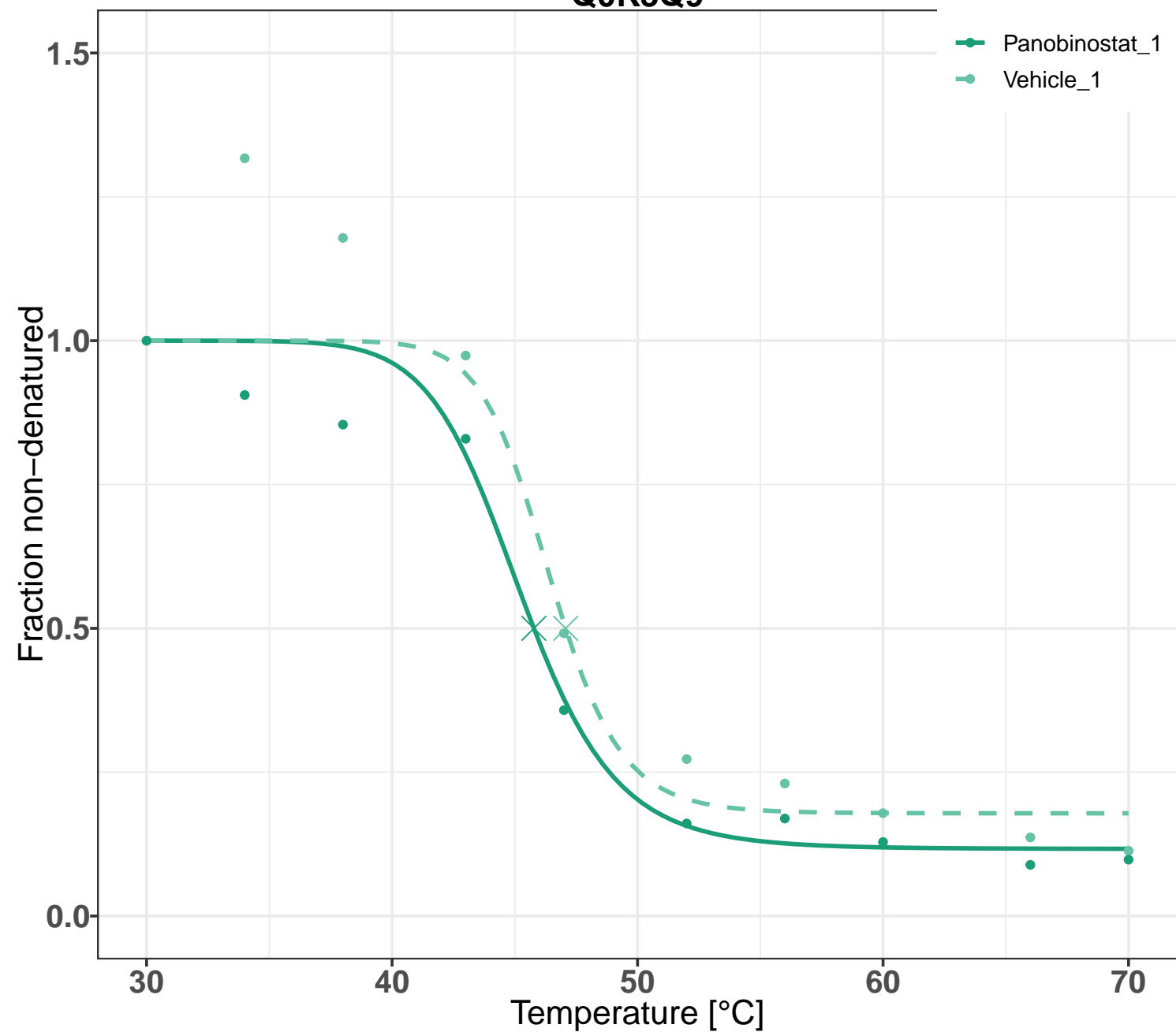

meltPoint

slope

plateau

R2

Panobinostat\_1

45.78

-0.12

0.12

0.98

Vehicle\_1

47.07

-0.14

0.18

0.93

Supplement: Supplementary file 2 — Supplementary Material 2 [file 41598_2026_35990_MOESM2_ESM.zip › AllTheTPPData/D40vD86/Panobinostat_Vignette/Melting_Curves/meltCurve_Q0K3Q9.pdf]

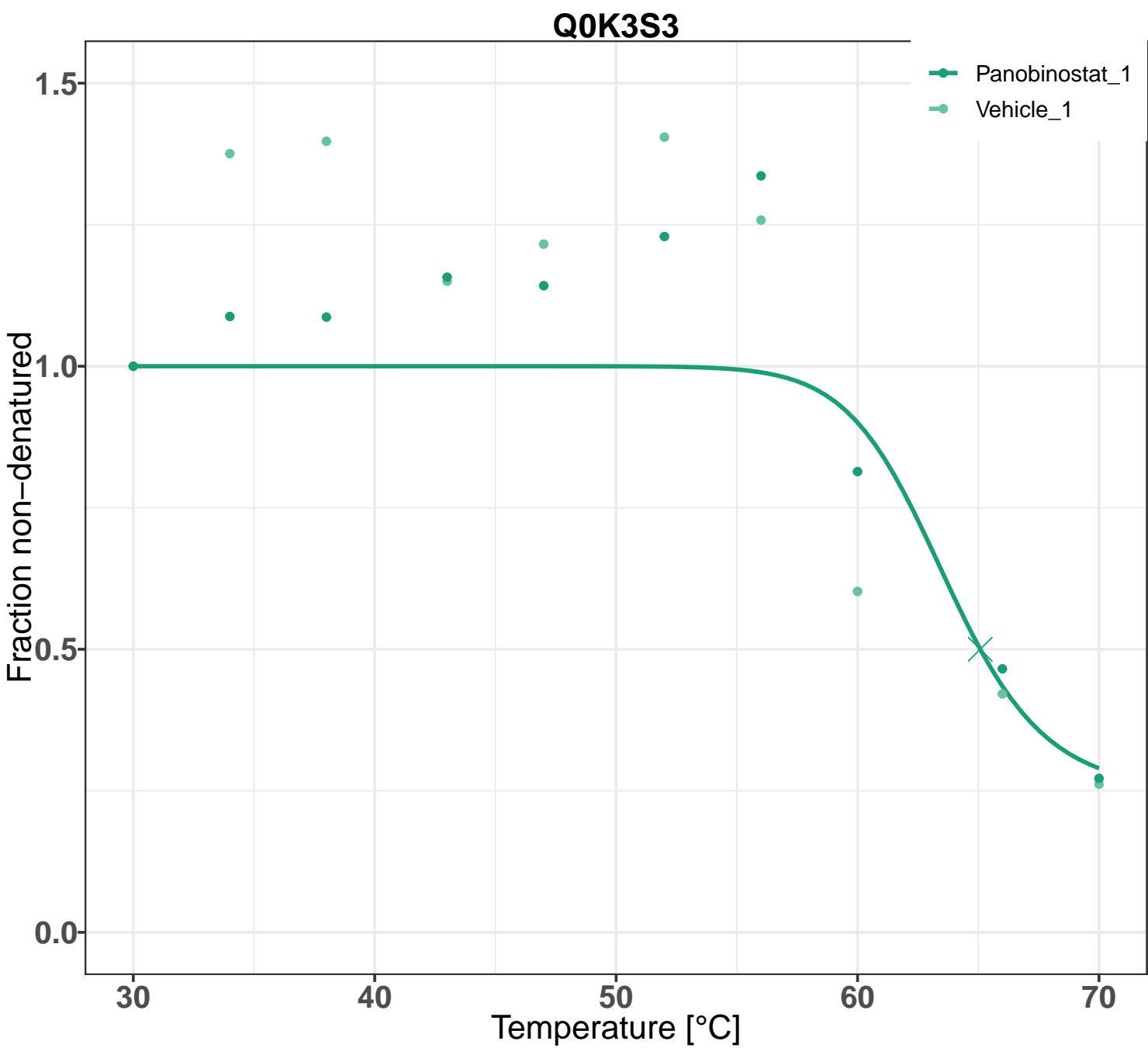

|                | meltPoint | slope  | plateau | R2   |
|----------------|-----------|--------|---------|------|
| Panobinostat_1 | 65.08     | -0.092 | 0.25    | 0.77 |
| Vehicle_1      | -         | -      | -       | -    |

Supplement: Supplementary file 2 — Supplementary Material 2 [file 41598_2026_35990_MOESM2_ESM.zip › AllTheTPPData/D40vD86/Panobinostat_Vignette/Melting_Curves/meltCurve_Q0K3S3.pdf]

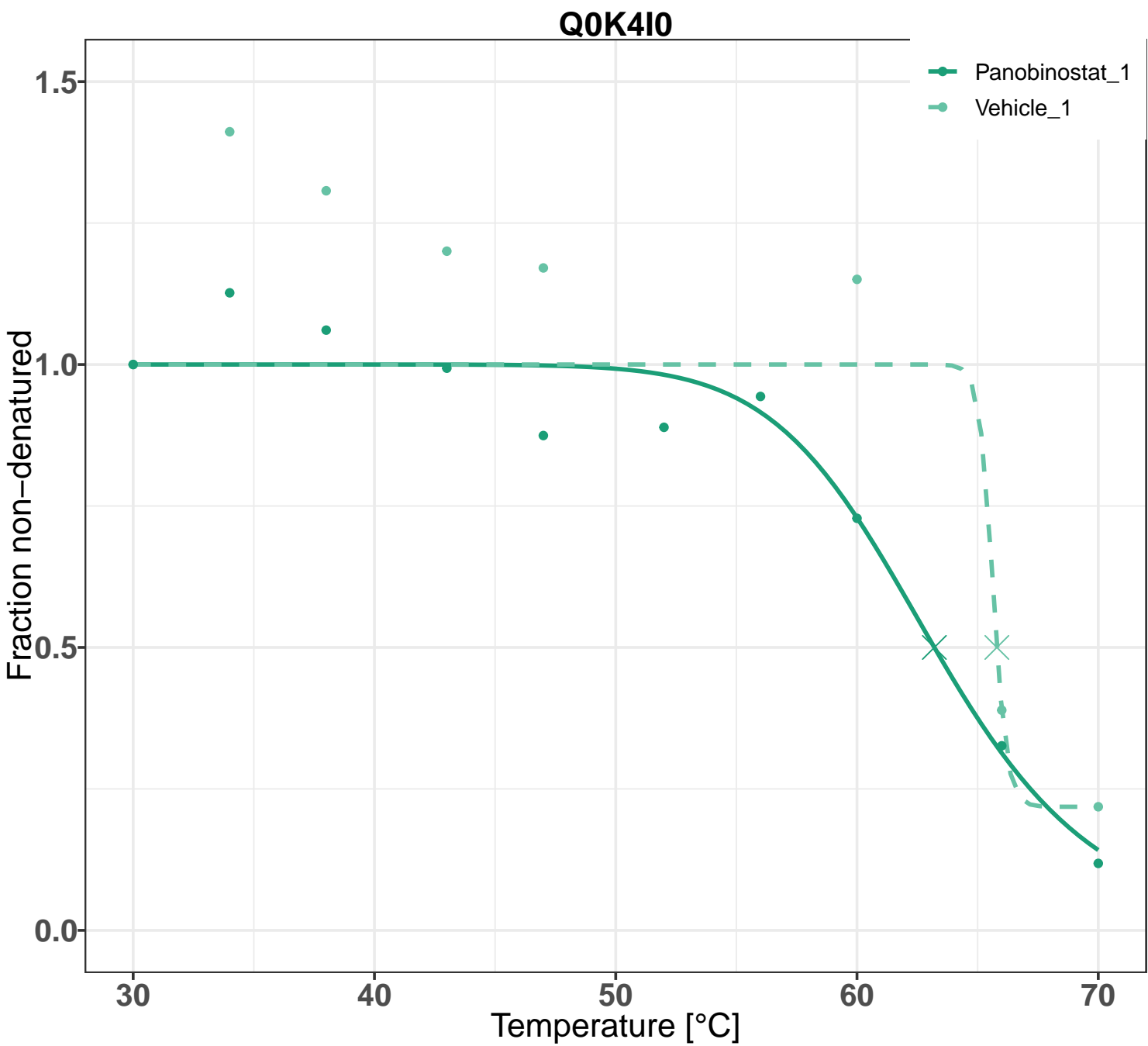

|                | meltPoint | slope  | plateau | R2   |
|----------------|-----------|--------|---------|------|
| Panobinostat_1 | 63.21     | -0.074 | 0       | 0.95 |
| Vehicle_1      | 65.8      | -0.68  | 0.22    | 0.26 |

Supplement: Supplementary file 2 — Supplementary Material 2 [file 41598_2026_35990_MOESM2_ESM.zip › AllTheTPPData/D40vD86/Panobinostat_Vignette/Melting_Curves/meltCurve_Q0K4I0.pdf]

Q0K4J3

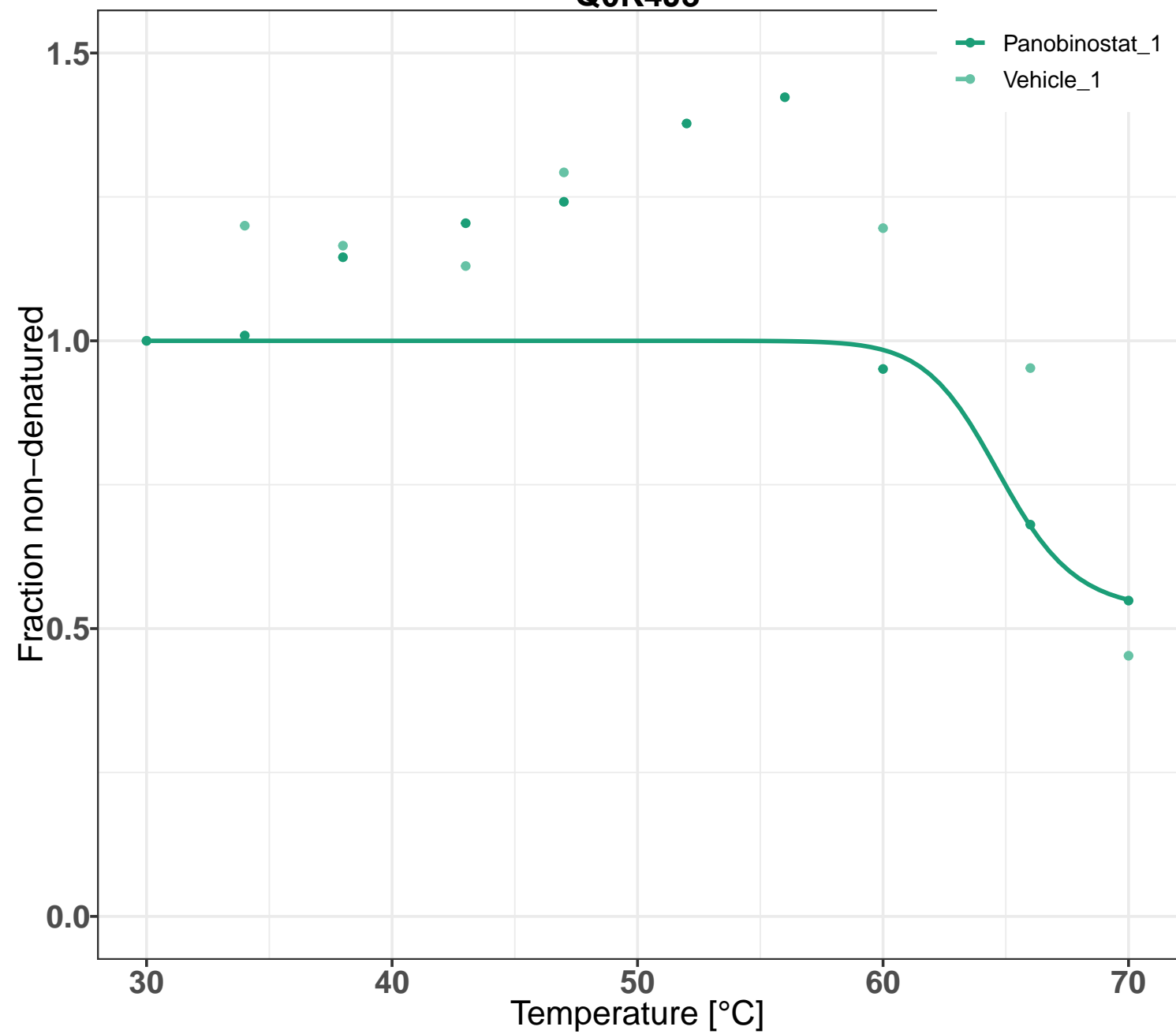

|                | meltPoint | slope  | plateau | R2   |
|----------------|-----------|--------|---------|------|
| Panobinostat_1 | –         | –0.077 | 0.53    | 0.38 |
| Vehicle_1      | –         | –      | –       | –    |

Supplement: Supplementary file 2 — Supplementary Material 2 [file 41598_2026_35990_MOESM2_ESM.zip › AllTheTPPData/D40vD86/Panobinostat_Vignette/Melting_Curves/meltCurve_Q0K4J3.pdf]

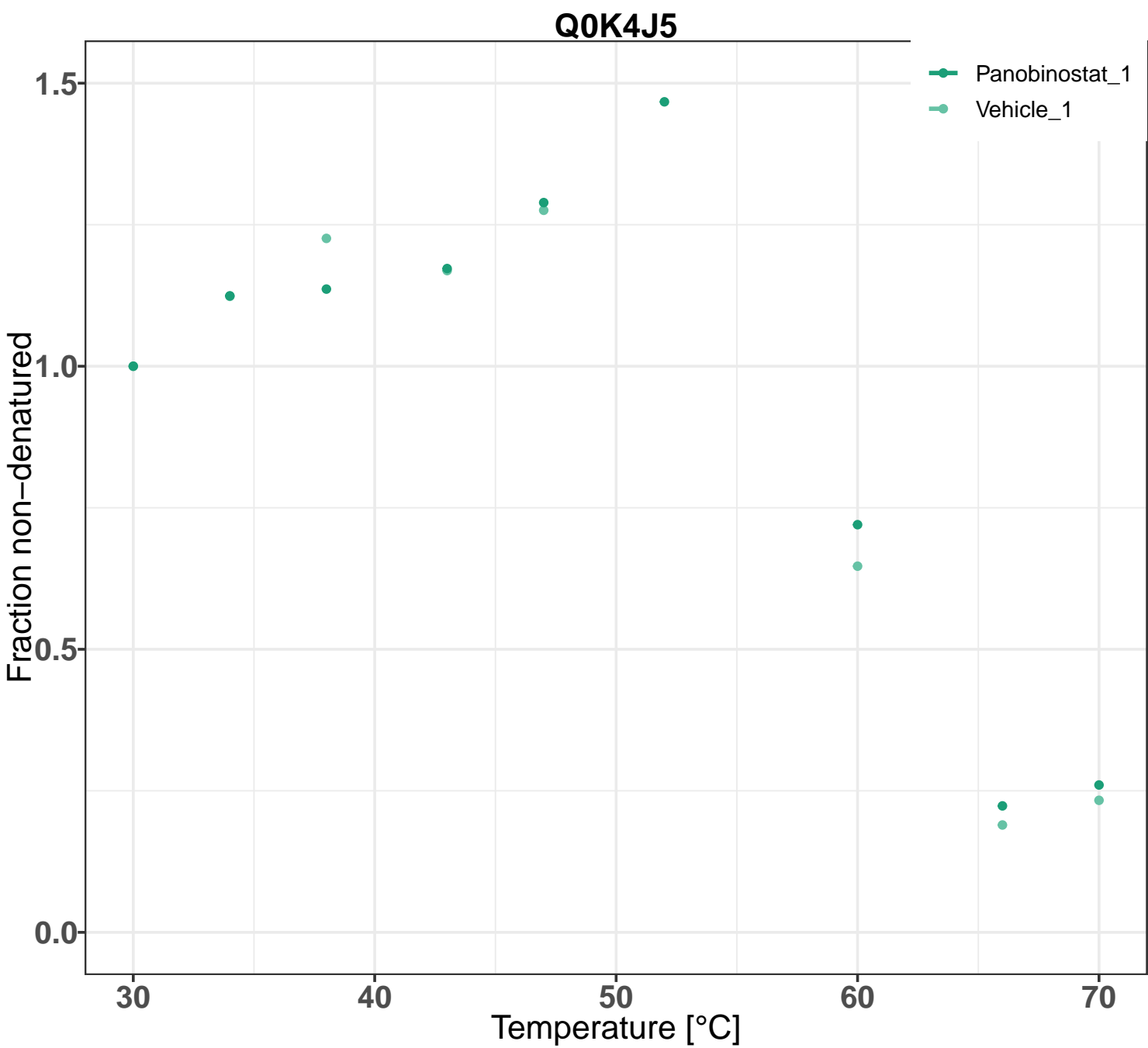

|                | meltPoint | slope | plateau | R2 |
|----------------|-----------|-------|---------|----|
| Panobinostat_1 | —         | —     | —       | —  |
| Vehicle_1      | —         | —     | —       | —  |

Supplement: Supplementary file 2 — Supplementary Material 2 [file 41598_2026_35990_MOESM2_ESM.zip › AllTheTPPData/D40vD86/Panobinostat_Vignette/Melting_Curves/meltCurve_Q0K4J5.pdf]

# Q0K4P3

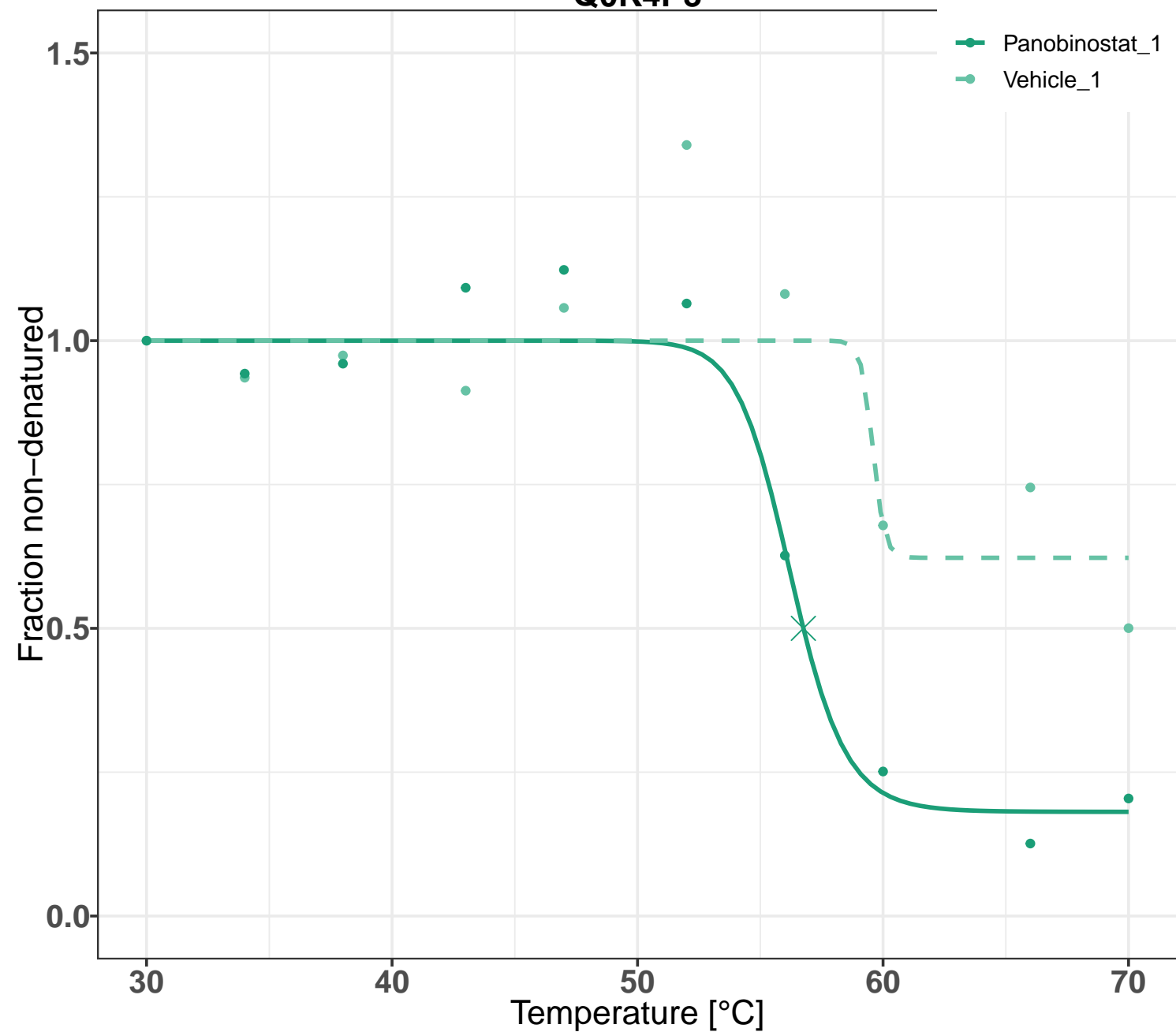

meltPoint

slope

plateau

R2

Panobinostat\_1

56.75

-0.19

0.18

0.97

Vehicle\_1

-

-0.4

0.62

0.66

Supplement: Supplementary file 2 — Supplementary Material 2 [file 41598_2026_35990_MOESM2_ESM.zip › AllTheTPPData/D40vD86/Panobinostat_Vignette/Melting_Curves/meltCurve_Q0K4P3.pdf]

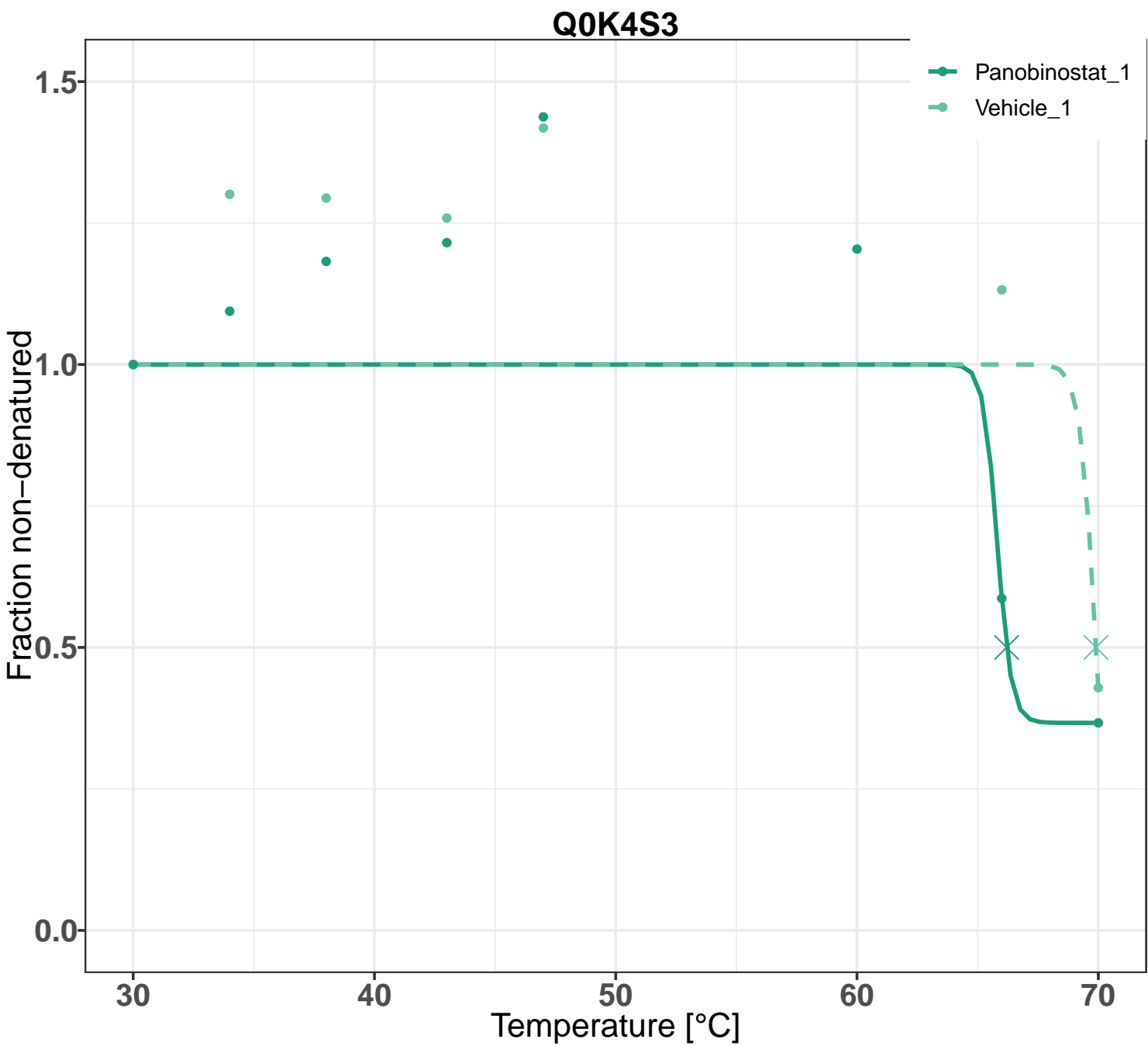

|                | meltPoint | slope | plateau | R2    |
|----------------|-----------|-------|---------|-------|
| Panobinostat_1 | 66.2      | -0.55 | 0.37    | 0.23  |
| Vehicle_1      | 69.91     | -0.77 | 0       | -0.46 |

Supplement: Supplementary file 2 — Supplementary Material 2 [file 41598_2026_35990_MOESM2_ESM.zip › AllTheTPPData/D40vD86/Panobinostat_Vignette/Melting_Curves/meltCurve_Q0K4S3.pdf]

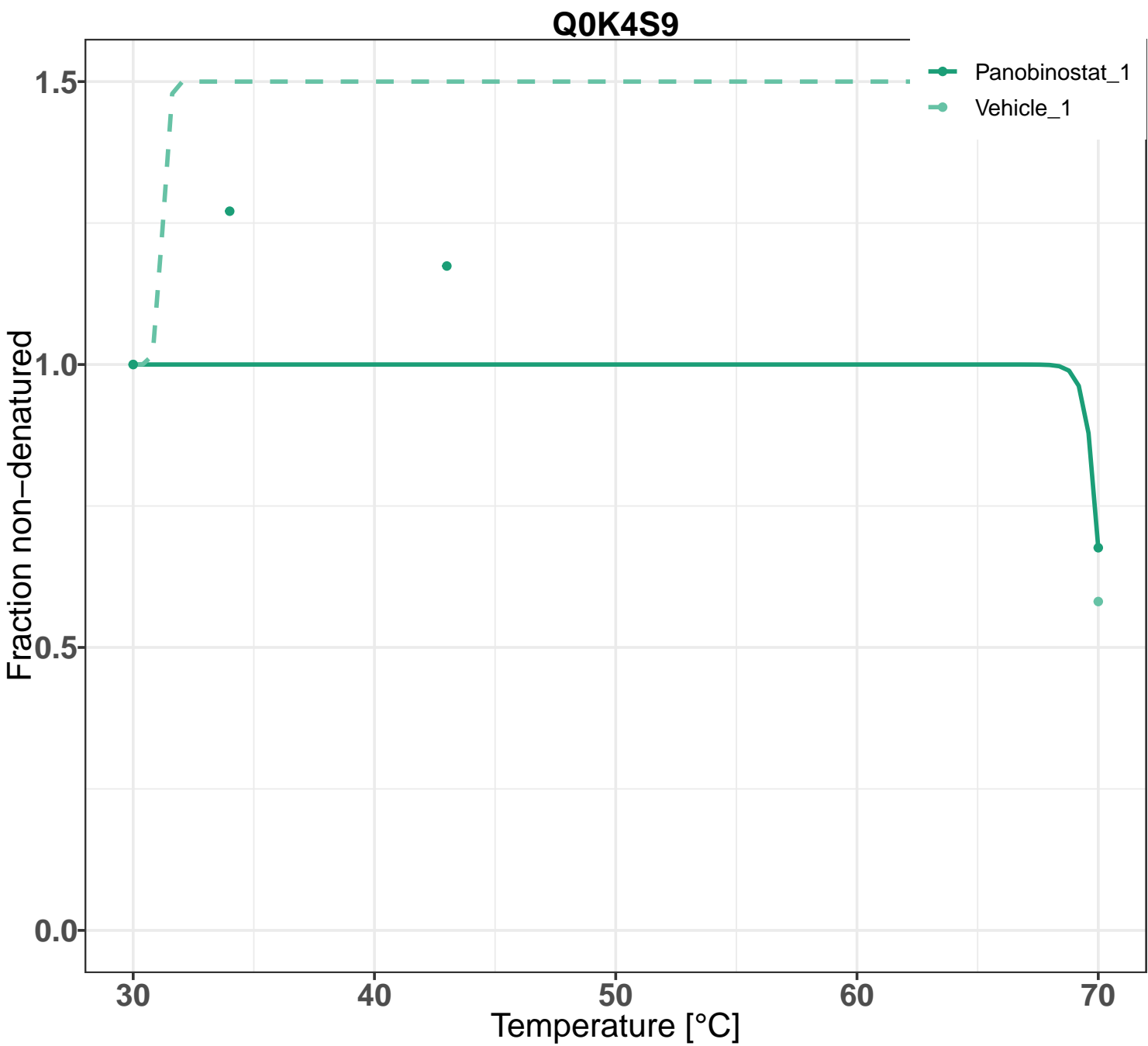

|                | meltPoint | slope | plateau | R2    |
|----------------|-----------|-------|---------|-------|
| Panobinostat_1 | –         | –     | 0       | –1.17 |
| Vehicle_1      | –         | 1     | 1.5     | –0.37 |

Supplement: Supplementary file 2 — Supplementary Material 2 [file 41598_2026_35990_MOESM2_ESM.zip › AllTheTPPData/D40vD86/Panobinostat_Vignette/Melting_Curves/meltCurve_Q0K4S9.pdf]

Q0K4U8

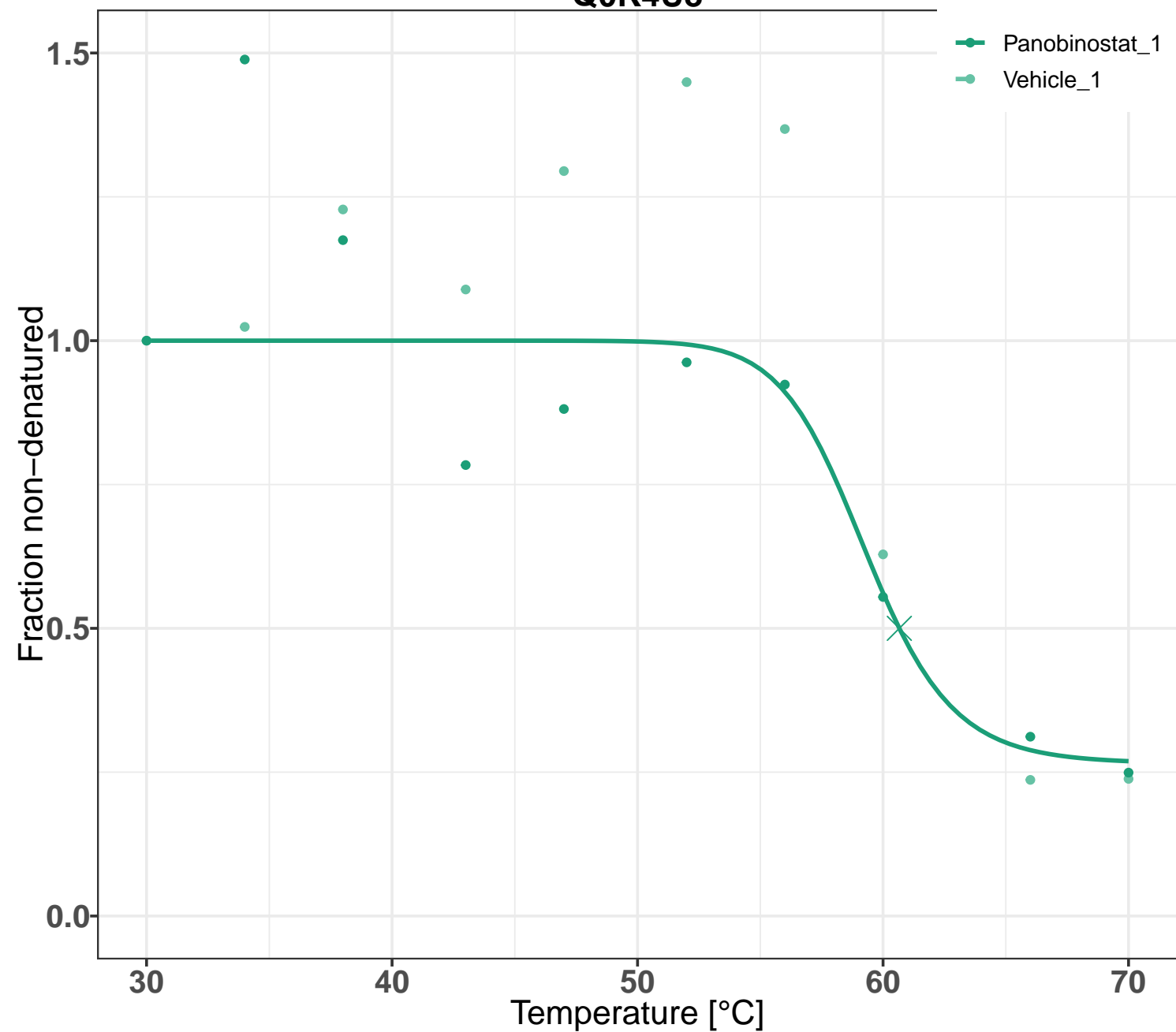

meltPoint

slope

plateau

R2

Panobinostat\_1

60.66

-0.1

0.26

0.74

Vehicle\_1

-

-

-

-

Supplement: Supplementary file 2 — Supplementary Material 2 [file 41598_2026_35990_MOESM2_ESM.zip › AllTheTPPData/D40vD86/Panobinostat_Vignette/Melting_Curves/meltCurve_Q0K4U8.pdf]
